# *MIG1* as a positive regulator for the histidine biosynthesis pathway and as a global regulator in thermotolerant yeast *Kluyveromyces marxianus*

**DOI:** 10.1038/s41598-019-46411-5

**Published:** 2019-07-09

**Authors:** Mochamad Nurcholis, Masayuki Murata, Savitree Limtong, Tomoyuki Kosaka, Mamoru Yamada

**Affiliations:** 1 Graduate School of Medicine, Yamaguchi University, Ube, 755-8505 Japan; 2 Department of Food Science and Technology, Faculty of Agricultural Technology, Brawijaya University, Malang, 65145 Indonesia; 3 Graduate School of Science and Technology for Innovation, Yamaguchi University, Yamaguchi, 753-8515 Japan; 4 Department of Microbiology, Faculty of Science, Kasetsart University, Bangkok, 10900 Thailand; 5 Department of Biological Chemistry, Faculty of Agriculture, Yamaguchi University, Yamaguchi, 753-8515 Japan; 6 Research Center for Thermotolerant Microbial Resources, Yamaguchi University, Yamaguchi, 753-8315 Japan

**Keywords:** Applied microbiology, Microbiology

## Abstract

*Kmmig1* as a disrupted mutant of *MIG1* encoding a regulator for glucose repression in *Kluyveromyces marxianus* exhibits a histidine-auxotrophic phenotype. Genome-wide expression analysis revealed that only *HIS4* in seven *HIS* genes for histidine biosynthesis was down-regulated in *Kmmig1*. Consistently, introduction of *HIS4* into *Kmmig1* suppressed the requirement of histidine. Considering the fact that His4 catalyzes four of ten steps in histidine biosynthesis, *K*. *marxianus* has evolved a novel and effective regulation mechanism via Mig1 for the control of histidine biosynthesis. Moreover, RNA-Seq analysis revealed that there were more than 1,000 differentially expressed genes in *Kmmig1*, suggesting that Mig1 is directly or indirectly involved in the regulation of their expression as a global regulator.

## Introduction

*Kluyveromyces marxianus*, a nonconventional yeast, has attractive characteristics including good thermotolerance, high ethanol productivity^1^, a broad spectrum in sugar assimilation^2,3^ and weak glucose repression on sucrose assimilation^4^. There have been several studies on sugar utilization and ethanol production by *K*. *marxianus* at high temperatures^1,2,4,5^ that were carried out with the aim of establishing high-temperature fermentation, which has advantages including reduction of cooling costs, prevention of contamination and reduction of enzymatic hydrolysis cost^5–7^. The regulation of some genes related to glucose repression in *K*. *marxianus* has also been investigated^4,8^, and such studies may provide crucial information for utilization of mixed sugars such as mixed sugars in general biomass.

One of the most important factors in the regulation of glucose repression in *K*. *marxianus* and its sister yeast species, *Saccharomyces cerevisiae*, is Mig1. ScMig1 has been shown to function as a regulator complex including ScHxk2 in glucose repression^9,10^ and to be involved in negative regulation of the expression of several genes including *GAL83*, *SUC2*, *MAL62*, *LAC4* and *LAC12* when glucose co-exists^11–15^. In *K*. *marxianus*, *MIG1* mutants have been shown to exhibit increased activities of β-galactosidase and inulinase^16,17^. KmMig1 with KmRag5, a orthologue of ScHxk2, is involved in negative regulation of the expression of *INU1* encoding inulinase and positive regulation of the expression of *RAG1* for a low-affinity glucose transporter, and, notably, a *MIG1*-disrupted mutant (*Kmmig1*), but not a *RAG5* mutant, exhibited a histidine-auxotrophic phenotype^8^.

The histidine biosynthesis pathway has been studied in detail in prokaryotes and lower eukaryotes^18,19^. The pathways in *Escherichia coli* and *Salmonella typhimurium* consist of 8 histidine genes^20^, whereas the pathway in *S*. *cerevisiae* has 7 genes including *HIS1*, *HIS2*, *HIS3*, *HIS4*, *HIS5*, *HIS6* and *HIS7*^21–26^. *K*. *marxianus* DMKU3-1042 also has seven *HIS* genes, the products of which are involved in ten steps of the histidine biosynthesis pathway^27^.

On the basis of a histidine-auxotrophic phenotype of *Kmmig1* in *K*. *marxianus*^8^, in order to understand the role of Mig1 for histidine biosynthesis, we performed genome-wide expression analysis with *Kmmig1* and complementation experiments with a candidate gene regulated by Mig1. The results suggested a novel regulation by Mig1, that is, *HIS4*, which encodes an enzyme catalyzing 4 steps of histidine biosynthesis, is positively regulated by Mig1. Additionally, the genome-wide expression analysis revealed that a defect of *MIG1* significantly affected the expression of 1,150 genes, in which 689 and 461 were up- and down-regulated, respectively. The results thus suggest that Mig1 is involved in the positive regulation and negative regulation of the expression of many genes in *K*. *marxianus*.

## Materials and Methods

### Materials

Oligonucleotide primers were purchased from Greiner Bio-one (Tokyo, Japan). A PCR purification kit, gel extraction kit, and RNeasy plus mini kit were from QIAGEN (Hilden, Germany). *Ex Taq* and primeSTAR DNA polymerases, In-fusion HD cloning kit, DNase treatment kit, and Yeastmaker^TM^ carrier DNA-Clontech were from Takara Bio (Shiga, Japan). A DNA sequencing kit was from Beckman Coulter (Deutschland, Germany). Zeomycin (Zeocin^TM^) was from Invitrogen-Thermo Fisher Scientific (Brookfield, USA). Yeast extract and zymolyase were from Nacalai Tesque (Kyoto, Japan). Peptone was from Kyokuto (Tokyo, Japan). D-glucose and RNase A were from SIGMA-ALDRICH (Tokyo, Japan). D-galactose was from Wako (Osaka, Japan). Yeast nitrogen base without amino acids was from DIFCO (Houston, USA). Other chemicals used in this study were of analytical grade.

### Strains, media and growth conditions

The yeast strains used in this study were *K*. *marxianus* DMKU3-1042^1^, *Kmmig1*, *Kmmig1 KmMIG1*^8^ and *Kmmig1 TDH3*-*HIS4*-*ble* (this study) and *S*. *cerevisiae* BY4741 (*MAT*a *his3Δ1 leu2Δ0 met15Δ0 ura3Δ0*)^28^. YP consists of 1% (*w/v*) yeast extract and 2% (*w/v*) peptone. The medium used to examine growth characteristics of yeast strains on agar plates was YP supplemented with 1.5% (*w/v*) agar and a carbon source, YPG (2% (*w/v*) galactose). The medium used to observe growth characteristics of yeast strains on minimal medium agar plates was 0.67% (*w/v*) yeast nitrogen base (YNB) without amino acids supplemented with 1.5% (*w/v*) agar and a carbon source, YNBD (2% (*w/v*) glucose) or YNBG (2% (*w/v*) galactose). If necessary, 0.01% (*w/v*) histidine was added. *E*. *coli DH5α* and SOC medium (Toyobo, Japan) were used for the In-fusion cloning method. LB (1% (*w/v*) tryptone (Nacalai Tesque, Japan), 0.5% (*w/v*) yeast extract (Nacalai Tesque, Japan), and 1% (*w/v*) NaCl (SIGMA-ALDRICH, Japan)) was used as a general medium for *E*. *coli*. If necessary, ampicillin (25 μg ml^−1^) (Wako, Japan), X-Gal (40 μg ml^−1^) (Nacalai Tesque, Japan), or IPTG (40 μg ml^−1^) (Nacalai Tesque, Japan) was added.

Cells were pre-cultured in 5 ml of YPG medium at 30 °C under a shaking condition at 160 rpm for 18 h. The pre-culture was inoculated into a 300-ml flask containing 100 ml of YNBG and 0.01% (*w/v*) histidine to adjust the initial optical density at 660 nm (OD_660_) to 0.1, followed by incubation at 30 °C for 24 h under a shaking condition at 160 rpm. Cell density was measured turbidimetrically at 660 nm on a spectrophotometer (U-2000A, Hitachi, Japan). To observe growth characteristics of yeast strains on agar plates, cells were streaked on YPG or YNBG and 0.01% (*w/v*) histidine and incubated at 30 °C for 24 h and 48 h.

### RNA preparation for RNA-Seq

Cells were pre-cultured in 5 ml of YPG at 30 °C under a shaking condition at 160 rpm for 18 h. The pre-culture was inoculated into a 300-ml flask containing 100 ml of YNBG and 0.01% (*w/v*) histidine at 30 °C under a shaking condition at 160 rpm for 12 h (in the case of wild type) and for 18 h (in the case of *Kmmig1*). At the mid-log phase, cells were harvested by centrifugation at 5,000 rpm for 5 min at 4 °C. The different pre-culture times were due to the fact that the growth of the latter was slower than that of the former. The cells were washed with YNBG and transferred to 100 ml of YNBG, followed by incubation at 30 °C for 1 h. The cells were harvested by centrifugation at 5,000 rpm for 5 min at 4 °C and subjected to an RNA preparation process. RNA was prepared by a modified procedure on the basis of the procedure reported previously^27^. The RNA samples then were subjected to RNase-free DNase treatment. All RNA samples were purified by using an RNeasy plus mini kit (QIAGEN) according to the protocol provided by supplier.

### RNA-Seq-based transcriptomic analysis

The purified RNA samples were analysed on an Illumina MiniSeq at the Research Center of Yamaguchi University. The detailed procedure for RNA-Seq has been described previously^29^. All these data were deposited under accession number DRA008595 in DDBJ Sequence Read Archive (https://www.ddbj.nig.ac.jp/dra/index-e.html). The sequencing results were analysed using CLC genomic workbench version 10.1.1. All mapped reads at exons were counted, and the numbers were converted to unique exon reads. The unique exon reads from three biological replicates of *Kmmig1* were compared to those of the parental strain.

Gene expression profiles of *Kmmig1* and the parental strain were compared to find differentially expressed genes (DEGs) based on unique exon read values from CLC genomic workbench outputs using DESeq2R package^30^. The resulting *P*-values were adjusted using Benjamin-Hochberg’s method for controlling the false discovery rate. Genes with adjusted *P* values less than 0.01 (*P*_adj_ < 0.01) and log_2_ (fold change) values greater than 1 or lower than −1 were assigned as significant DEGs. Kyoto Encyclopedia of Genes and Genomes (KEGG) pathway mapping with these significant DEGs was performed by KEGG web tools (http://www.genome.jp/keg/tool/map_pathway1.html). Gene ontology (GO) enrichment analysis of significant DEGs was performed using topGO R package^31^. GO terms with *P* values less than 0.01 were considered significantly enriched.

### Increased expression of *HIS4* in *Kmmig1*

For increased expression of *HIS4* in *Kmmig1*, a *TDH3*-*HIS4*-*ble* DNA fragment was constructed as follows. The *TDH3* promoter fragment was amplified by PCR using genomic DNA of *S*. *cerevisiae* BY4741 as a template and primers prTDH3-5′-F and prTDH3-3′-R (Table 1). The primers were designed to amplify the fragment corresponding to the region from the *TDH3* start codon to 993-bp upstream of the start codon. The *HIS4* fragment was amplified by PCR using genomic DNA of *K*. *marxianus* DMKU 3-1042 as a template and primers HIS4TD-5′-F and HIS4BL-3′-R. The primers were designed to amplify the fragment corresponding to the region from the start codon of *HIS4* (2,409 bp). The *ble* gene (zeomycin resistance gene) was amplified by PCR from pSH65 plasmid DNA as a template with primers BLE-5′-F and BLE-3′-R^32^. Linear pUC19 DNA (Takara Bio, Japan) was prepared by PCR amplification with primers pUC19-5′-F and pUC19-3′-R (Table 1). The four amplified fragments were purified using a QIAquick gel extraction kit, connected by the In-fusion cloning method (Takara Bio, Japan), introduced into *E*. *coli DH5α* by using the heat shock method^33^, and screened on LB plates containing ampicillin, IPTG and X-Gal. Transformants harboring pUC19 containing a *TDH3*-*HIS4*-*ble* fragment (3,905 bp) were confirmed by colony PCR. The *TDH3*-*HIS4*-*ble* fragment was amplified by PCR and directly introduced into *Kmmig1* by the lithium acetate method^34,35^. Transformants were obtained on YPD plates containing zeomycin (100 μg ml^−1^), and recombinants were then examined by PCR to check the existence of the *TDH3-HIS4-b1e* fragment, generating *Kmmig1 TDH3*-*HIS4*-*ble*. Physiological confirmation tests were carried out on YNBD or YNBG plates in the absence or presence of 0.01% (*w/v*) histidine.

**Table 1:** Primers used in this study.

No.	Primer name	Nucleotide sequences
1	pUC19-5′-F	5′-GATCCTCTAGAGTCGACCTG-3′
2	pUC19-3′-R	5′-GATCCCCGGGTACCGAGCTC-3′
3	prTDH3-5′-F	5′-CGACTCTAGAGGATCCGAGGACCTTGTCACCTTGAG-3′
4	prTDH3-3′-R	5′-TTTGTTTGTTTATGTGTGTT-3′
5	HIS4TD-5′-F	5′-ACATAAACAAACAAAATGTTACCTCTTGTGCCCTTA-3′
6	HIS4BL-3′-R	5′-TCGCCCTTAGATTAGTTATTCAAAATTAGGTGGTA-3′
7	BLE-5′-F	5′-CTAATCTAAGGGCGAGCTCG-3′
8	BLE-3′-R	5′-CGGTACCCGGGGATCTCCGTCGAGTGGGTGGTGA-3′

Underline indicates additional hanging 15 nucleotides for In-fusion cloning.

### Ethics statement

This article does not contain any studies with human participants or animals performed by any of the authors.

## Results

### Effect of *MIG1*-disrupted mutation on expression of genes for histidine biosynthesis

*K*. *marxianus* became histidine-auxotrophic when *MIG1* was disrupted^8^, but the possibility that *MIG1* is not directly involved in the regulation of histidine biosynthesis but that other genes are directly involved could not be excluded. We thus decided not to examine only genes for histidine biosynthesis but to perform genome-wide expression analysis by RNA-Seq. RNA-Seq analysis was performed with RNAs prepared from *Kmmig1* and parental cells that had been incubated for 1 h after shifting from a minimal medium in the presence of histidine to that in the absence of histidine. After sequencing and removing the adaptors and the low quality reads, more than 0.8 Gb clean data qualified for follow-up analysis were acquired from each sample, being equivalent to more than 75-fold genome coverage. Unique exon reads of each gene were determined as transcript abundance. The difference in expression of each gene in *Kmmig1* from that in the parental strain was shown as the ratio of unique exon reads in *Kmmig1* to that in the parental strain. To further explore the transcriptional changes in *Kmmig1* compared to those in the parental strain, we conducted analysis of DEGs based on the ratio of unique exon reads. Significant DEGs showed changes in the transcription level with log_2_ (fold change) > 1 and log_2_ (fold change) < −1 (*P*_adj_ < 0.01). *Kmmig1* was found to have 1,150 DEGs including 689 up-regulated and 461 down-regulated genes (Supplementary Information Fig. S1 and File S1).

**Figure 1:**
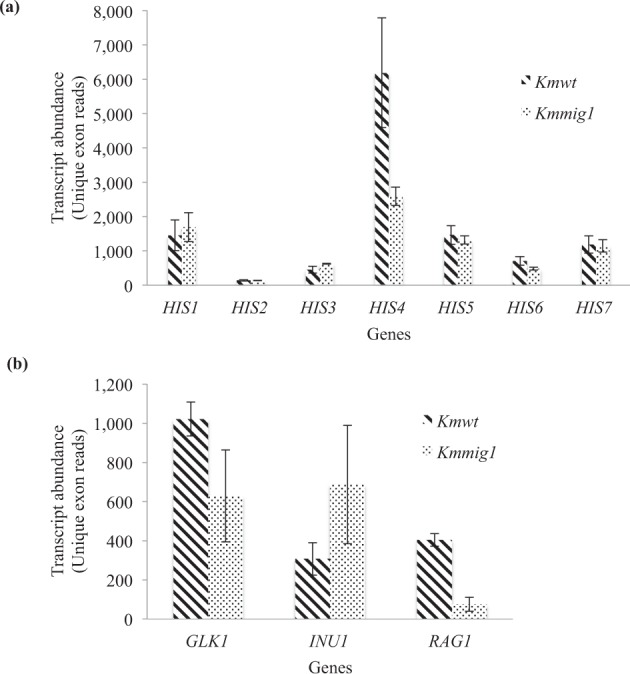
Effects of *MIG1*-disrupted mutation on transcription of several genes for histidine biosynthesis and of *GLK1*, *INU1* and *RAG1* in *K*. *marxianus*. RNA-Seq analysis was performed as described in Materials and methods. Transcript abundance in the form of unique exon reads of *Km*WT and *Kmmig1* was estimated for several genes for histidine biosynthesis (**a**) and *GLK1* for glucokinase, *INU1* for inulinase and *RAG1* for glucose transporter (**b**) in *K*. *marxianus*. Data presented are averages of triplicate independent experiments, and error bars indicate standard deviations.

**Figure 2:**
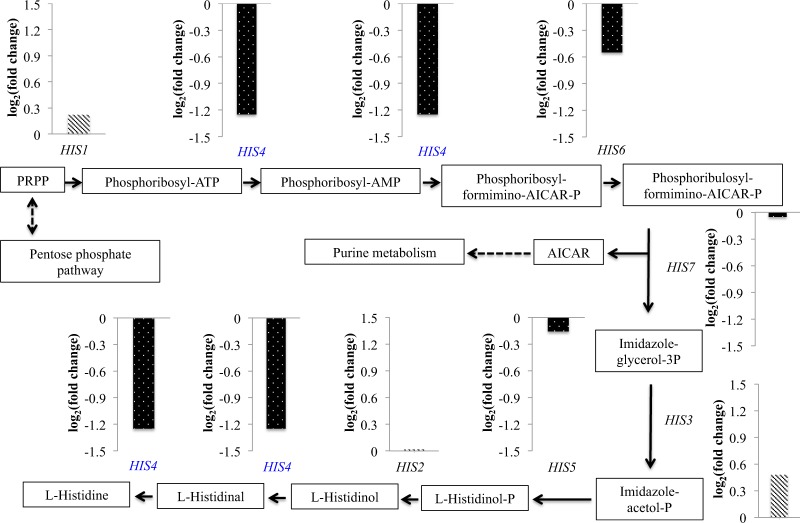
Schematic representation of *MIG1*-disruption effects on the expression of *HIS* genes for histidine biosynthesis in *K*. *marxianus*. The ratio of the transcriptional level of each gene in *Kmmig1* to that in the parental strain is presented by log_2_(fold change). The log_2_(fold change) values of the up-regulation are represented as backslash columns, while values of the down-regulation are represented as dotted columns. Further details are given in Supplementary Information File S4.

In order to explore the gene(s) responsible for a histidine-auxotrophic phenotype in *Kmmig1*, the unique exon reads of seven *HIS* genes for histidine biosynthesis were compared in *Kmmig1* and the parental strain (Fig. 1a). Analysis of DEGs indicated that the expression level of *HIS4* in *Kmmig1* was 2.4-times lower than that in the parental strain (Fig. 2). There was almost no difference between the expression levels of other *HIS* genes. Therefore, these findings indicated the possibility that the histidine-auxotrophic phenotype of *Kmmig1* was due to reduction in the expression of *HIS4*.

To confirm the significance of down-regulation of *HIS4*, the consistency of the RNA-Seq data and previous RT-PCR data for *INU1*, *RAG1* and *GLK1* was examined. A comparison of the RT-PCR data for *Kmmig1* and the parental strain^8^ revealed that the *MIG1*-disrupted mutation increased *INU1* expression by 3 fold, decreased *RAG1* expression by more than 2 fold and had almost no effect on *GLK1* expression. Consistently, the unique exon reads of *INU1* and *RAG1* in *Kmmig1* were 2.2-times higher and 5.3-times lower, respectively, than those in the parental strain, and the unique exon reads of *GLK1* in *Kmmig1* were not different from those in the parental strain (Fig. 1b). Therefore, the RNA-Seq data confirmed the previous conclusion that Mig1 is involved in the negative regulation of *INU1* and in the positive regulation of *RAG1*^8^ and suggested positive regulation of *HIS4* by Mig1. Notably, although the RNA samples were prepared from cells grown under different medium conditions, YPD for RT-PCR analysis and histidine-free YNBG for RNA-Seq analysis, data obtained from the different medium conditions showed good consistency in expression of the three genes. These facts may indicate that incubation in histidine-free YNBG for 1 h has almost no effect on cell metabolism and that the data therefore reflect only the effects of *MIG1*-disrupted mutation on the expression of genomic genes and that the influence of histidine-free YNBG is limited and is specific to some pathways, for example, histidine biosynthesis.

### Increased expression of *HIS4* in *Kmmig1*

**Figure 3:**
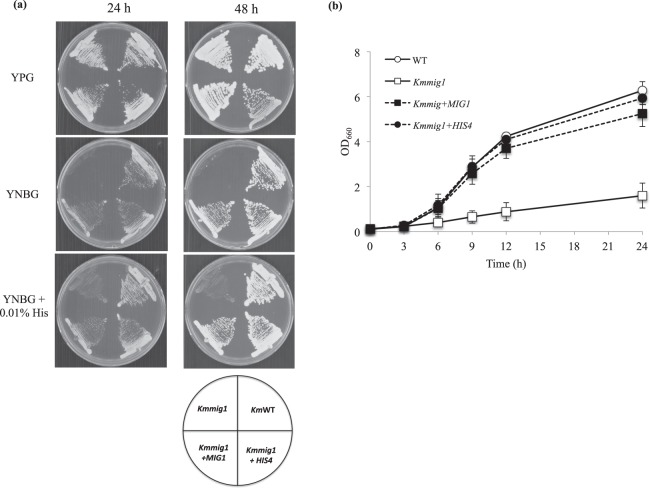
Complementation experiments by increased expression of *HIS4* under control of the *S*. *cerevisiae TDH3* promoter in *Kmmig1*. Cells were pre-cultured in 5 ml of YPG at 30 °C under a shaking condition at 160 rpm for 15–18 h. (**a**) The cells were streaked on plates of YNBG, YNBG supplemented with 0.01% (*w/v*) histidine and YPG as a control. The plates were incubated at 30 °C and photos were taken at 24 h and 48 h. (**b**) The pre-cultured cells were inoculated in 100 ml of YNBG and 0.01% (*w/v*) histidine at the final OD_660_ of 0.1 and cultivated at 30 °C under a shaking condition at 160 rpm for 24 h.

RNA-Seq analysis indicated the possibility that down-regulation of the expression of *HIS4* was responsible for the histidine-auxotrophic phenotype in *Kmmig1*. Interestingly, His4 is involved in 4 catalytic steps of the histidine biosynthesis pathway (Fig. 2) and down-regulation (58% reduction) at each step thus led to a large effect (97% reduction) on the entire histidine biosynthesis. Increased expression of *HIS4* in *Kmmig1* was thus tested (Fig. 3). A DNA fragment of *TDH3*-*HIS4*-*ble*, in which *HIS4* was under the control of the promoter of *TDH3* as one of the strong promoters from *S*. *cerevisiae*, was constructed and introduced into the genome of *Kmmig1*. The growth of the recombinant on YNBG without histidine was compared with that of *Kmmig1* (Fig. 3a). *Kmmig1* exhibited almost no growth as expected, but the recombinant grew well like the wild type. Similarly, the recombinant showed growth equivalent to that of the wild type in the liquid minimal medium, but *Kmmig1* showed greatly retarded growth even with the addition of 0.01% (*w/v*) histidine to the medium (Fig. 3b). These results and the down-regulation of *HIS4* in *Kmmig1* (*MIG1*-disruption mutation) suggest that the down-regulation of *HIS4* in *Kmmig1* caused the defect of growth in the minimal medium and that Mig1 positively regulates *HIS4* expression. However, we cannot exclude the possibility that Mig1 regulates *His4* expression via another regulator(s).

### Effects of *MIG1*-disrupted mutation on expression of genomic genes

Since there were many significant DEGs caused by the *MIG1*-disrupted mutation, suggesting its global influence on the genomic genes in *K*. *marxianus*, these DEGs were subjected to a GO term enrichment test (Supplementary Information File S2). In the 689 up-regulated DEGs, the enriched GO terms for biological processes were related to the lipid catabolic process, cellular lipid catabolic process, fatty acid catabolic process, fatty acid oxidation, lipid oxidation, fatty acid beta-oxidation, fatty acid metabolic process, organic acid catabolic process, carboxylic acid catabolic process, small molecule catabolic process, monocarboxylic acid catabolic process, lipid modification, antibiotic metabolic process, glutamate metabolic process, and other processes. The enriched GO terms for cellular components included an integral component of the membrane, intrinsic component of the membrane, membrane part, peroxisome organelle, integral component of the peroxisome, peroxisomal matrix and intrinsic component of the peroxisome, cell wall, and other cellular components. The GO terms for molecular functions included oxidoreductase activity, catalytic activity, transmembrane transporter activity, hydrolase activity, transporter activity, coenzyme binding, and other molecular functions (Supplementary Information File S2).

On the other hand, in the 461 down-regulated DEGs, the enriched GO terms for biological processes included ribosome biogenesis, rRNA processing, ribonucleoprotein complex biogenesis, rRNA metabolic process, ncRNA processing, ncRNA metabolic process, glycolytic process, ATP generation from ADP, pyruvate biosynthetic process, nucleoside diphosphate metabolic process, purine nucleoside diphosphate metabolic process, purine ribonucleoside diphosphate metabolic process, and pyridine nucleotide biosynthetic process. The enriched GO terms for cellular components included the preribosome, nucleolus, small-subunit processome, ribonucleoprotein complex, nucleolar part, 90S preribosome, preribosome, large subunit precursor, nuclear lumen, cytosolic ribosome, cytosolic large ribosomal subunit, nucleolus, and nucleus. The enriched GO terms for molecular functions included snoRNA binding, RNA binding, rRNA binding, oxidoreductase acitivity, oxidoreductase activity, organic cyclic compound binding, heterocyclic compound binding, iron ion binding, coenzyme binding, and nucleic acid binding (Supplementary Information File S2).

The up-regulated and down-regulated DEGs were also mapped to the terms in the KEGG database (Supplementary Information File S3). The mapping analysis revealed that pathways related to the 689 up-regulated DEGs included metabolic pathways, biosynthesis of secondary metabolites, biosynthesis of antibiotics, carbon metabolism, autophagy, MAPK signaling pathway, biosynthesis of amino acids, meiosis, peroxisome, glyoxylate and dicarboxylate metabolism, spliceosome, fatty acid degradation, glycolysis/gluconeogenesis, glycerolipid metabolism, endocytosis, arginine and proline metabolism, citrate cycle (TCA cycle), glycine, serine, and threonine metabolism, tyrosine metabolism, phenylalanine metabolism, autophagy, pyruvate metabolism, purine metabolism, and pyrimidine metabolism. Pathways related to the 461 down-regulated DEGs included metabolic pathways, biosynthesis of secondary metabolites, biosynthesis of antibiotics, ribosome biogenesis in eukaryotes, biosynthesis of amino acids, carbon metabolism, glycolysis/gluconeogenesis, ribosome, purine metabolism, RNA transport, methane metabolism, starch and sucrose metabolism, cysteine and methionine metabolism, MAPK signaling pathway, galactose metabolism, RNA polymerase, cell cycle, glycine, serine and threonine metabolism, steroid biosynthesis, amino sugar and nucleotide sugar metabolism, pentose phosphate pathway, pyruvate metabolism, fatty acid metabolism, and pyrimidine metabolism.

To further understand the possible downstream relationship from Mig1, we explored significant DEGs for transcription factors (TFs) that are orthologues to those of *S*. *cerevisiae*, from the lists in Supplementary Information File S1^36–47^. As a result, three down-regulated genes corresponding to *SFP1*, *RGT1*, and *MTH1* in *S*. *cerevisiae* and four up-regulated genes corresponding to *KAR4*, *ADR1*, *GSM1*, and *SIP4* in *S*. *cerevisiae* were found, and they were subjected to GO and KEGG analyses but no item in KEGG pathway was found for all TFs (Supplementary Information Table S1). Based on the physiological functions of these TFs in *S*. *cerevisiae* (Supplementary Information Table S2), it is assumed that KLMA_60316 (Rgt1) and KLMA_30237 (Mth1) function under a glucose-rich condition, whereas KLMA_60316 (Rgt1), KLMA_20117 (Adr1), KLMA_20140 (Gsm1), and KLMA_30166 (Sip4) function under a glucose-starved condition. Such glucose level-specific manners might indicate the link with Mig1. The remaining two, KLMA_40457 (Sfp1) and KLMA_10029 (Kar4), presumably regulate cognate genes under a condition unrelated to glucose level. These putative TFs could be involved in regulation of the expression of genes included in terms of GO (Supplementary Information Table S1). Notably, in *S*. *cerevisiae*, Sip4 expression is negatively regulated by Mig1 via Cat8^48^, Sip4 activates the expression of many genes for gluconeogenesis^49^, and the expression of 108 genes is significantly decreased in the absence of Adr1^50^. Taken together, the results indicate the possibility that Mig1 regulates many genes directly or indirectly via various TFs including the seven putative TFs described above.

### Effects of *MIG1*-disrupted mutation on central carbon metabolism

**Figure 4:**
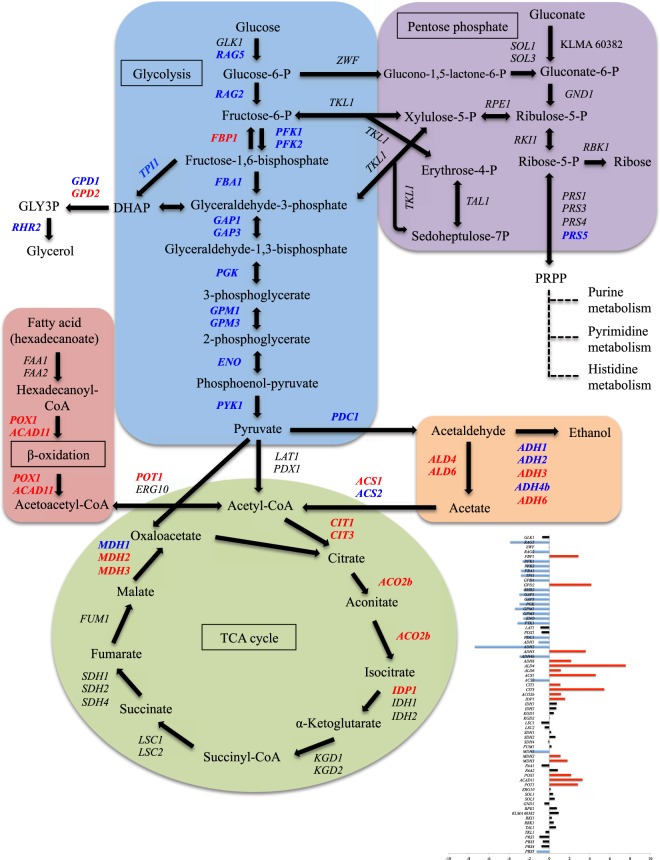
Expressional change of genes for the central carbon metabolic network in *Kmmig1*. The ratio of the transcriptional level of each gene in *Kmmig1* to that in the parental strain that is presented by log_2_(fold change) is shown at the right bottom side. Red-coloured bars: significantly up-regulated genes in *Kmmig1*; blue-coloured bars: significantly down-regulated genes; black-coloured bars: not significantly changed genes. In the central carbon metabolic network, significantly up-regulated and down-regulated genes in *Kmmig1* are represented in red and blue, respectively, and genes that were not significantly changed in *Kmmig1* are represented in black. Further details are given in Supplementary Information File S4.

Since Mig1 is known to be a regulator of glucose repression in *K*. *marxianus*^8,51^ as well as in *S*. *cerevisiae*^52,53^, the effects of *MIG1*-disrupted mutation on central carbon metabolism were focused on. The mutation caused changes in the transcriptional levels of most of the genes involved in central carbon metabolism (Fig. 4 and Supplementary Information File S1). Most of the genes for the glycolytic pathway including *RAG5* for hexokinase, *RAG2* for glucose-6-phosphate isomerase, *PFK1* and *PFK2* for phosphofructokinase, *FBA1* for fructose-bisphosphate aldolase, *GAP1* for glyceraldehyde-3-phosphate dehydrogenase 1, *GAP3* for glyceraldehyde-3-phosphate dehydrogenase 3, *PGK* for phosphoglycerate kinase, *GPM1* for phosphoglycerate mutase 1, *GPM3* for phosphoglycerate mutase 3, *ENO* for enolase and *PYK1* for pyruvate kinase were significantly down-regulated in *Kmmig1*. In addition, *PDC1* for pyruvate decarboxylase, *ADH1* for alcohol dehydrogenase 1 and *ADH2* for alcohol dehydrogenase 2, which are related to ethanol production^54,55^, and *GPD1* for glycerol-3-phosphate dehydrogenase and *RHR2* for glycerol-3-phosphatase 1, which are related to glycerol production^56,57^, were down-regulated. On the other hand, *FBP1* for fructose-1,6-bisphosphatase, which is involved in gluconeogenesis, was significantly up-regulated. Many genes for ethanol degradation, TCA cycle and fatty acid degradation, including *ADH3*, *ADH6*, *ACS1*, *CIT1*, *CIT3*, *ACO2b*, *IDP1*, *MDH2*, *MDH3*, *POX1*, *ACAD11* and *POT1* encoding alcohol dehydrogenase 3, alcohol dehydrogenase 6, acetyl-coenzyme A synthetase 1, citrate synthase 1, citrate synthase 3, aconitate hydratase, isocitrate dehydrogenase, malate dehydrogenase 2, malate dehydrogenase 3, acyl-coenzyme A oxidase, acyl-CoA dehydrogenase family member 11 and 3-ketoacyl-CoA thiolase, respectively, were also significantly up-regulated (Fig. 4 and Supplementary Information File S1).

## Discussion

Physiological analysis of the effect of *MIG1*-disrupted mutation indicated the possibility that Mig1 is required for histidine biosynthesis^8^. In this study, in order to understand the role of Mig1 in histidine biosynthesis, genome-wide expression analysis was performed. Among seven *HIS* genes for enzymes related to the histidine biosynthesis pathway, only the expression level of *HIS4* in the *MIG1*-disrupted mutant was significantly down-regulated compared to that in the parental strain. The level of reduction of *HIS4* expression was only 58%, but it is assumed that such an intermediate level of the effect becomes very strong in total to cause the defect of growth in a minimal medium because His4 catalyzes 4 steps in the histidine biosynthesis pathway. This assumption was examined by increased expression of *HIS4* in *Kmmig1*, resulting in the recovery of growth in the minimal medium without the addition of histidine (Fig. 3). Consequently, it is thought that Mig1 is a positive regulator for *HIS4* and thus for histidine biosynthesis. It is noteworthy that the regulation of histidine biosynthesis in *K*. *marxianus* by Mig1 creates a novel and effective mechanism targeting one gene, *HIS4*, of which the product is involved in 4 catalytic steps of histidine biosynthesis. Interestingly, *S*. *cerevisiae* has three His4-involved steps in the histidine biosynthesis pathway^24^, though its regulation by Mig1 remains to be investigated. On the other hand, Hua *et al*. reported that *Kmmig1* was isolated on a minimal medium (SD medium)^58^. It is possible that they took slowly formed colonies when *Kmmig1* was screened because we noticed that *Kmmig1* is able to grow on a minimal medium, though very slowly^8^ (Fig. 3 in this manuscript). Alternatively, their *Kmmig1* might have an additional suppressor mutation that allowed it to grow on the SD medium or the histidine-auxotrophic phenotype of *Kmmig1* might be strain-specific.

In *S*. *cerevisiae*, Mig1 has been extensively analysed and shown to be a key regulator as a complex with other proteins including Hxk2 for glucose repression^9,10^. Surprisingly, the present study indicated the possibility that Mig1 is a global regulator for genomic genes in *K*. *marxianus*. Transcriptome analysis of the *MIG1*-disrupted mutant and its parental strain was performed with RNAs prepared from cells that were cultivated in a minimal medium containing galactose as a sole carbon source (under a condition with no glucose repression). The analysis suggests that Mig1 acts as a positive regulator for most genes (except *GLK1* and *FBP1*) in glycolysis and as a negative regulator for many genes in the TCA cycle and fatty acid degradation (Fig. 4). In anabolic pathways, Mig1 may activate the expression of genes for biosynthesis of secondary metabolites, antibiotics and amino acids, ribosome biogenesis, rRNA processing, and purine and pyrimidine metabolism and inhibit the expression of genes for biosynthesis of secondary metabolites, antibiotics and amino acids, and for gluconeogenesis. Considering that the medium still contained a sufficient amount of galactose under the condition in which RNA was prepared for RNA-Seq and considering that Mig1 seems to activate genes for the ethanol synthesis pathway in addition to genes for glycolysis and to inhibit genes for the TCA cycle, it is likely that Mig1 is a crucial regulation factor to enhance ethanol production in *K*. *marxianus*.

In addition, as shown in KEGG analysis, the down-regulation of genes for ribosome biogenesis, biosynthesis of amino acids, carbon metabolism, ribosome, RNA transport, RNA polymerase and purine and pyrimidine metabolism in *Kmmig1* suggests that Mig1 promotes cell proliferation. There are some pathways that seem to be subjected to both positive regulation and negative regulation by Mig1; for example, biosynthesis of secondary metabolites, antibiotics, amino acids, purine and pyrimidine. It is assumed that such a dual regulation by Mig1 contributes to the fine tuning of these pathways or balanced metabolism in cells. Further analysis of the regulation of individual gene expression in these pathways may lead to an understanding of the physiological importance of Mig1-directed regulation in each pathway.

## Supplementary information

Supplementary Information of Nurcholis et al.

## Data Availability

Results of all data analyses performed in this study are included in this manuscript and its Supplementary Information files.
