# Author Correction: *MIG1* as a positive regulator for the histidine biosynthesis pathway and as a global regulator in thermotolerant yeast *Kluyveromyces marxianus*

**DOI:** 10.1038/s41598-020-60963-x

**Published:** 2020-03-04

**Authors:** Mochamad Nurcholis, Masayuki Murata, Savitree Limtong, Tomoyuki Kosaka, Mamoru Yamada

**Affiliations:** 1 Graduate School of Medicine, Yamaguchi University, Ube, 755-8505 Japan; 2 Department of Food Science and Technology, Faculty of Agricultural Technology, Brawijaya University, Malang, 65145 Indonesia; 3 Graduate School of Science and Technology for Innovation, Yamaguchi University, Yamaguchi, 753-8515 Japan; 4 Department of Microbiology, Faculty of Science, Kasetsart University, Bangkok, 10900 Thailand; 5 Department of Biological Chemistry, Faculty of Agriculture, Yamaguchi University, Yamaguchi, 753-8515 Japan; 6 Research Center for Thermotolerant Microbial Resources, Yamaguchi University, Yamaguchi, 753-8315 Japan

Correction to: *Scientific Reports* 10.1038/s41598-019-46411-5, published online 09 July 2019

The Supplementary Information file that accompanies this Article contains an error in Supplementary Table S2, where the reference numbers shown in the table are incorrect. The correct Table S2 appears below as Table 1.

**Table 1:** Transcription factors (TFs) of *S. cerevisiae*, of which orthologs in *K. marxianus* are presumably located downstream of Mig1.

TFs^a^	Description/Function	Reference
Sfp1	A stress- and nutrient-sensitive regulator of ribosomal protein (RP) gene expression and biogenesis genes; Novel heat shock TFs and regulates RP gene expression in response to heat shock.	36, 37
Rgt1	Glucose-responsive transcription factor; regulates expression of several glucose transporter (HXT) genes in response to glucose; bind to promoters and acts both as a transcriptional activator and repressor	38, 39
Mth1	Negative regulator of the glucose-sensing signal transduction pathway; required for repression of transcription by Rgt1; interacts with Rgt1 and the Snf3 and Rgt2 glucose sensors.	40, 41, 42
Kar4	Acting at a subset of Ste12-inducible genes in the pheromone-dependent expression; a karyogamy-specific component; required for the induction of *KAR3* and *CIK1*.	43
Adr1	A carbon source-responsive zinc-finger transcription factor; required for transcription of the glucose-repressed genes for ethanol, glycerol and fatty acid utilization.	44, 50
Gsm1	Putative zinc cluster protein of unknown function; proposed to be involved in the regulation of energy metabolism based on pattern of expression.	45
Sip4	C_6_ zinc cluster transcriptional activator; binds to the carbon source-responsive element (CSRE) of gluconeogenic genes; involved in the positive regulation of gluconeogenesis; regulated by Snf1 protein kinase.	46, 47, 49

^a^These TFs are shown in Table S1.

