# Supplementary material for: MIG1 as a positive regulator for the histidine biosynthesis pathway and as a global regulator in thermotolerant yeast Kluyveromyces marxianus
Source: Sci Rep. 2019 Jul 9;9:9926. doi: 10.1038/s41598-019-46411-5 (PMC6617469; doi:10.1038/s41598-019-46411-5)
Supplement: Supplementary file 1 — Supplementary Information of Nurcholis et al. [file 41598_2019_46411_MOESM1_ESM.pdf]

MIG1 as a positive regulator for the histidine biosynthesis pathway and as a global regulator in thermotolerant yeast *Kluyveromyces marxianus*

Mochamad Nurcholis[1,2], Masayuki Murata[3], Savitree Limtong[4], Tomoyuki Kosaka[3,5,6], Mamoru Yamada[1,3,5,6*]

[1]Graduate School of Medicine, Yamaguchi University, Ube 755-8505, Japan

[2]Department of Food Science and Technology, Faculty of Agricultural Technology, Brawijaya University, Malang 65145, Indonesia

[3]Graduate School of Science and Technology for Innovation, Yamaguchi University, Yamaguchi 753-8515, Japan

[4]Department of Microbiology, Faculty of Science, Kasetsart University, Bangkok 10900, Thailand

[5]Department of Biological Chemistry, Faculty of Agriculture, Yamaguchi University, Yamaguchi 753-8515, Japan

[6]Research Center for Thermotolerant Microbial Resources, Yamaguchi University, Yamaguchi 753-8315, Japan

[*]Corresponding authors: Mamoru Yamada (e-mail: m-yamada@yamaguchi-u.ac.jp)

Phone: +81 90-7595-5196

ORCID: 0000-0003-4354-7324

Supplementary Figure

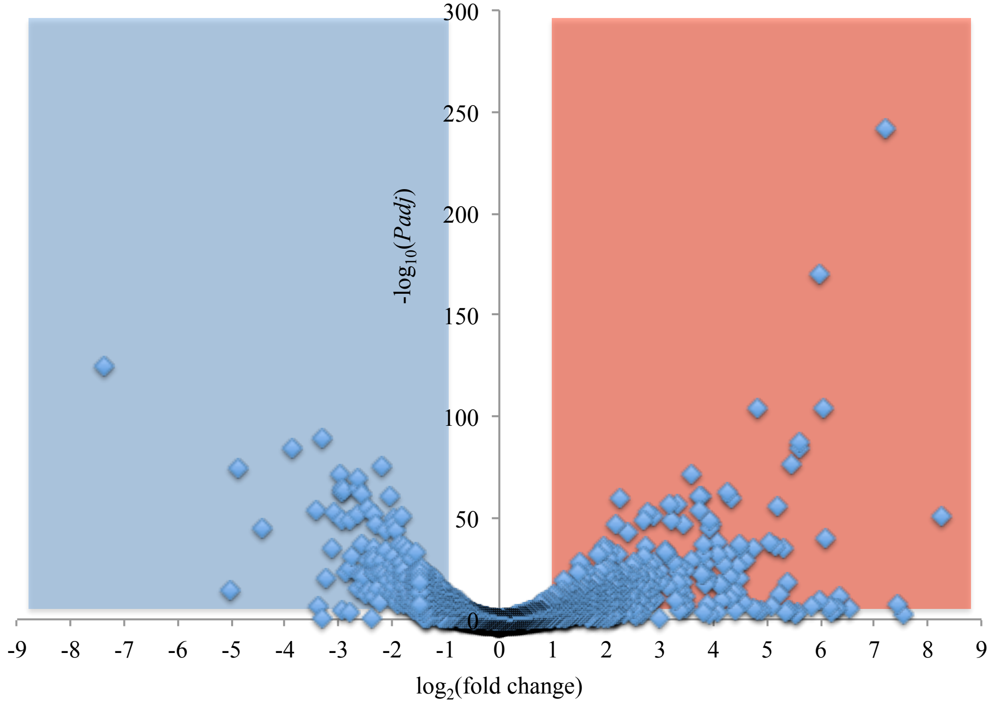

Supplementary Figure S1. Diagram plots of differentially expressed genes (DEGs) for *Kmmig1* and the parental strain of *K. marxianus*. Genes with adjusted *P* values (*Padj*) less than 0.01 and $\log_2$(fold change) values greater than 1 were assigned as differentially expressed. Red coloured bar: significantly up-regulated genes in *Kmmig1*; blue coloured bar: significantly down-regulated genes in *Kmmig1*.

Supplementary Tables

Supplementary Table S1. Transcription factors (TFs) that are assumed to be under the control of *MIG1* in *K. marxianus*

TFs		Query coverage [a] (%)	Identity [a] (%)	Analyses [b]	
S.cerevisiae	*K. marxianus*			DEGs [c]	GO terms
Sfp1	KLMA_40457 (Sfp1)	75	45	D	Organic cyclic compound binding, nucleic acid binding
Rgt1	KLMA_60316 (Rgt1)	56	33	D	Organic substance metabolic process, metabolic process, small molecule metabolic process, primary metabolic process, hexose metabolic process, monosaccharide metabolic process, carbohydrate metabolic process, glucose metabolic process, nucleus, organic cyclic compound binding, heterocyclic compound binding, nucleic acid binding, transition metal ion binding
Mth1	KLMA_30237 (Mth1)	99	50	D	-
Kar4	KLMA_10029 (Kar4)	89	70	U	Reproductive process, multi organism process, response to pheromone
Adr1	KLMA_20117 (Adr1)	70	32	U	-
Gsm1	KLMA_20140 (Gsm1)	99	37	U	Drug metabolic process, transition metal ion binding, zinc ion binding
Sip4	KLMA_30166 (Sip4)	80	31	U	Transition metal ion binding, zinc ion binding

[a] Query coverage and identity when TF in *S. cerevisiae* was compared with the corresponding ortholog (https://blast.ncbi.nlm.nih.gov/Blast.cgi) in *K. marxianus*.
[b] These data are from Supplementary Information File S1, File S2, and File S3.
[c] D, significantly down-regulated by *MIG1* disruption; U, significantly up-regulated by *MIG1* disruption in *K. marxianus*.
"-" mean that the transcription-related factors were not included any items in GO terms.

Supplementary Table S2. **Transcription factors (TFs) of *S. cerevisiae*, of which orthologs in *K. marxianus* are presumably located downstream of Mig1**

TFs [a]	Description/Function	Reference
Sfp1	A stress- and nutrient-sensitive regulator of ribosomal protein (RP) gene expression and biogenesis genes; Novel heat shock TFs and regulates RP gene expression in response to heat shock.	47, 48
Rgt1	Glucose-responsive transcription factor; regulates expression of several glucose transporter (HXT) genes in response to glucose; bind to promoters and acts both as a transcriptional activator and repressor	49, 50
Mth1	Negative regulator of the glucose-sensing signal transduction pathway; required for repression of transcription by Rgt1; interacts with Rgt1 and the Snf3 and Rgt2 glucose sensors.	51, 52, 53
Kar4	Acting at a subset of Ste12-inducible genes in the pheromone-dependent expression; a karyogamy-specific component; required for the induction of *KAR3* and *CIK1*.	54
Adr1	A carbon source-responsive zinc-finger transcription factor; required for transcription of the glucose-repressed genes for ethanol, glycerol and fatty acid utilization.	38, 55
Gsm1	Putative zinc cluster protein of unknown function; proposed to be involved in the regulation of energy metabolism based on pattern of expression.	56
Sip4	C_6 zinc cluster transcriptional activator; binds to the carbon source-responsive element (CSRE) of gluconeogenic genes; involved in the positive regulation of gluconeogenesis; regulated by Snf1 protein kinase.	37, 57, 58

[a] These TFs are shown in Table S1.

Supplementary Files

Supplementary File S1. Differentially expressed genes (DEGs) based on unique exon reads for *Kmmig1* and the parental strain of *K. marxianus*

Down-regulated DEGs

Locus_tag	UniProt _gene	Product	baseMean	log_2FoldChange	lfcSE	stat	pvalue	padj
KLMA_10031	SPB1	adoMet-dependent rRNA methyltransferase SPB1	894.1376186	-1.041340272	0.151229212	-6.885840745	5.74E-12	5.45E-11
KLMA_10035	APA2	5',5'''-P-1,P-4-tetraphosphate phosphorylase 2	2494.817491	-1.781132181	0.202374251	-8.801179868	1.35E-18	2.63E-17
KLMA_10050	GRH1	GRASP65 homolog protein 1	503.3257319	-1.190414472	0.171256722	-6.951052532	3.63E-12	3.53E-11
KLMA_10054	SRO9	RNA-binding protein SRO9	611.7488969	-1.114073206	0.21231716	-5.247212269	1.54E-07	7.80E-07
KLMA_10065	HIS4	histidine biosynthesis trifunctional protein	4388.328621	-1.255591334	0.218378988	-5.749597737	8.95E-09	5.49E-08
KLMA_10068		SH3 super family	262.6397174	-1.074486655	0.196817564	-5.459302696	4.78E-08	2.62E-07
KLMA_10078	LPP1	lipid phosphate phosphatase 1	129.9127625	-1.197386511	0.260387051	-4.598487158	4.26E-06	1.70E-05
KLMA_10079	SAM2	S-adenosylmethionine synthetase 2	11798.21897	-1.760611861	0.279709217	-6.294436356	3.09E-10	2.30E-09
KLMA_10083	RpL37a	ribosomal_L37e super family	2271.817758	-1.089999768	0.226536375	-4.811588288	1.50E-06	6.51E-06
KLMA_10100	CCN1	G1/S-specific cyclin CLN1	1269.053296	-1.427923225	0.168394807	-8.479615547	2.26E-17	3.90E-16
KLMA_10106	CAN1	arginine permease	5708.034862	-1.807486782	0.253320546	-7.135176405	9.67E-13	1.01E-11
KLMA_10107	LYP1	lysine-specific permease	5983.841715	-1.514040177	0.173318901	-8.735574526	2.42E-18	4.61E-17
KLMA_10113		uncharacterized membrane protein YGL140C	1808.983135	-1.291808264	0.163264792	-7.912350527	2.53E-15	3.53E-14
KLMA_10134	RPA49	DNA-directed RNA polymerase I subunit RPA49	1455.520381	-1.12743058	0.150066316	-7.512882392	5.78E-14	6.91E-13
KLMA_10146		Zn2/Cys6 DNA-binding domain	1023.450608	-1.203074301	0.21121543	-5.69595839	1.23E-08	7.35E-08
KLMA_10153	ACO2	probable aconitate hydratase 2	1449.860785	-1.0319743	0.255734799	-4.035329977	5.45E-05	0.000172253
KLMA_10176	PRS5	ribose-phosphate pyrophosphokinase 5	1254.392765	-1.260334383	0.140589109	-8.964665833	3.11E-19	6.39E-18
KLMA_10179	GPD1	glycerol-3-phosphate dehydrogenase	12492.90635	-1.949083761	0.192187305	-10.14158434	3.61E-24	1.14E-22

Locus_tag	UniProt_gene	Product	baseMean	log_2FoldChange	lfcSE	stat	pvalue	padj
		[NAD+] 1						
KLMA_10182	GPM3	phosphoglycerate mutase 3	1336.33172	-2.640043092	0.169630591	-15.56348464	1.29E-54	1.75E-52
KLMA_10199	NOP1	rRNA 2'-O-methyltransferase fibrillarin	3741.071188	-1.370254553	0.287188493	-4.77127247	1.83E-06	7.84E-06
KLMA_10225	GSP1	GTP-binding nuclear protein GSP1/Ran	2556.470636	-1.164741274	0.187860029	-6.200048414	5.64E-10	4.12E-09
KLMA_10238		hypothetical protein	1688.529958	-2.064856886	0.216897084	-9.519984526	1.73E-21	4.17E-20
KLMA_10244	OLE1	acyl-CoA desaturase 1	35818.34597	-1.217264125	0.2834003	-4.29521114	1.75E-05	6.16E-05
KLMA_10245	SDS23	protein SDS23	967.0167794	-1.149009072	0.150233322	-7.648163922	2.04E-14	2.53E-13
KLMA_10255	SOH1	mediator of RNA polymerase II transcription subunit 31	47.88991982	-1.116590043	0.33823059	-3.301268652	0.000962487	0.002289715
KLMA_10278	DSS1	exoribonuclease II	632.2214344	-1.171570409	0.155075124	-7.554857164	4.19E-14	5.09E-13
KLMA_10282	PNO1	pre-rRNA-processing protein PNO1	648.2827106	-1.00970132	0.153042454	-6.597524355	4.18E-11	3.54E-10
KLMA_10293	UTP5	U3 small nucleolar RNA-associated protein 5	804.9826495	-1.131332665	0.178744246	-6.329337522	2.46E-10	1.87E-09
KLMA_10294	HPT1	hypoxanthine-guanine phosphoribosyltransferase	1563.401708	-1.345243433	0.164324672	-8.186496998	2.69E-16	4.14E-15
KLMA_10305		probable phosphoglycerate mutase YOR283W	612.253329	-1.270593148	0.173451212	-7.325363331	2.38E-13	2.66E-12
KLMA_10317	GUA1	GMP synthase	7461.181859	-1.429034677	0.240991103	-5.929823373	3.03E-09	2.00E-08
KLMA_10320	GAS3	probable 1,3-beta-glucanosyltransferase GAS3	4180.83398	-1.082675114	0.175674372	-6.162965613	7.14E-10	5.17E-09
KLMA_10334	SDA1	protein SDA1	947.443798	-1.249471405	0.148609505	-8.407748891	4.18E-17	7.09E-16
KLMA_10341	PFK1	6-phosphofructokinase subunit alpha	15263.93002	-2.640809857	0.146797317	-17.9894968	2.35E-72	7.19E-70
KLMA_10348	TIF1	ATP-dependent RNA helicase eIF4A	13443.44369	-1.063102703	0.19713128	-5.392866628	6.93E-08	3.70E-07
KLMA_10356	BIO2	biotin synthase	1051.91972	-1.968773362	0.228226209	-8.626412243	6.33E-18	1.16E-16
KLMA_10376	RPL17B	ribosomal_L22	9005.104751	-1.091349165	0.215241096	-5.070356853	3.97E-07	1.87E-06
KLMA_10388	EBP2	rRNA-processing protein EBP2	707.6693472	-1.001755274	0.15198119	-6.591310915	4.36E-11	3.68E-10
KLMA_10404	ATX2	zinc/iron permease	451.0110051	-1.411507447	0.196855834	-7.170259682	7.49E-13	7.88E-12
KLMA_10418	RPF2	ribosome biogenesis protein RPF2	789.5340646	-1.194914251	0.167115114	-7.150246442	8.66E-13	9.06E-12
KLMA_10425	RSE1	pre-mRNA-splicing factor RSE1	839.5689713	-1.039923533	0.153244909	-6.786023366	1.15E-11	1.06E-10

Locus_tag	UniProt _gene	Product	baseMean	log_2FoldChange	lfcSE	stat	pvalue	padj
KLMA_10427	GAL80	galactose/lactose metabolism regulatory protein GAL80	7563.520855	-2.203361321	0.116997722	-18.83251466	4.09E-79	1.66E-76
KLMA_10458	KYE1	enoate reductase 1	11211.97802	-3.089059005	0.196356545	-15.73188717	9.14E-56	1.31E-53
KLMA_10462	ENO	enolase	197772.0979	-2.567456121	0.260257513	-9.865060527	5.90E-23	1.63E-21
KLMA_10475	TNA1	high-affinity nicotinic acid transporter	7522.923924	-2.212869687	0.365482442	-6.054653876	1.41E-09	9.72E-09
KLMA_10483	MES1	methionyl-tRNA synthetase	3012.908779	-1.187524774	0.219172921	-5.418209361	6.02E-08	3.26E-07
KLMA_10484	HAS1	ATP-dependent RNA helicase HAS1	1447.493641	-1.560360676	0.150763737	-10.34970814	4.20E-25	1.44E-23
KLMA_10487	TDA1	probable serine/threonine-protein kinase YMR291W	1275.696206	-2.527690544	0.251804151	-10.0383196	1.03E-23	3.08E-22
KLMA_10491	YTA7	TAT-binding homolog 7	2061.769883	-1.200762067	0.155863504	-7.703933508	1.32E-14	1.69E-13
KLMA_10500	LIP1	ceramide synthase subunit LIP1	319.1530522	-1.063545974	0.201249428	-5.2847155	1.26E-07	6.44E-07
KLMA_10513	FAU1	5-formyltetrahydrofolate cyclo-ligase	607.5176754	-1.11488885	0.356432484	-3.127910335	0.001760539	0.003961061
KLMA_10517		uncharacterized protein conserved in bacteria	341.3432005	-1.039624013	0.204117391	-5.093265246	3.52E-07	1.67E-06
KLMA_10532	TAT2	tryptophan permease	2532.393485	-1.641427223	0.245264041	-6.692490338	2.19E-11	1.93E-10
KLMA_10540	PGK	phosphoglycerate kinase	70718.63609	-2.93840867	0.19365807	-15.1731796	5.32E-52	6.19E-50
KLMA_10548	CWH43	protein CWH43	2190.992292	-1.535375958	0.198234778	-7.745240115	9.54E-15	1.26E-13
KLMA_10577	SCS7	inositolphosphorylceramide-B C-26 hydroxylase	3204.286643	-1.246707238	0.257166296	-4.847864038	1.25E-06	5.48E-06
KLMA_10578		uncharacterized protein YML108W	64.10315166	-1.474618504	0.3451787	-4.272043744	1.94E-05	6.79E-05
KLMA_10612	PRY2	protein PRY1	530.6555327	-1.362221242	0.23093505	-5.898720198	3.66E-09	2.41E-08
KLMA_10654	FUR4	uracil permease	773.4095232	-1.425127077	0.201635513	-7.067837669	1.57E-12	1.58E-11
KLMA_10660	SCO1	SCO	1108.363623	-1.497905229	0.187879282	-7.972700419	1.55E-15	2.24E-14
KLMA_10671	OCA5	uncharacterized protein YHL029C	1838.009208	-2.334900529	0.192646091	-12.12015526	8.26E-34	4.58E-32
KLMA_10677	MET17	protein MET17	14501.85954	-1.556866639	0.284175473	-5.478539793	4.29E-08	2.37E-07
KLMA_10692	MSW1	tryptophanyl-tRNA synthetase	341.7188264	-1.374654685	0.235742252	-5.831176524	5.50E-09	3.50E-08
KLMA_10704	bioA	uncharacterized aminotransferase C1771.03c	370.0329324	-1.333477257	0.275883841	-4.833473586	1.34E-06	5.85E-06
KLMA_10711	TAH11	hypothetical protein	277.7064567	-1.340055436	0.211288249	-6.342309354	2.26E-10	1.73E-09
KLMA_10735	FUR1	uracil phosphoribosyltransferase	1014.279239	-1.684360617	0.2655921	-6.34190783	2.27E-10	1.73E-09

Locus_tag	UniProt _gene	Product	baseMean	log_2FoldChange	lfcSE	stat	pvalue	padj
KLMA_10747	AAH1	adenosine deaminase	752.6387687	-1.529148165	0.169350465	-9.029489	1.72E-19	3.63E-18
KLMA_10758	GCR1	glycolytic genes transcriptional activator GCR1	2710.667128	-2.895389133	0.16785961	-17.24887324	1.14E-66	2.93E-64
KLMA_10763	RAG2	glucose-6-phosphate isomerase	29864.27498	-1.669829927	0.240537597	-6.942074536	3.86E-12	3.75E-11
KLMA_10768	RIM2	mitochondrial carrier protein RIM2	567.3929963	-1.090510705	0.161955789	-6.73338514	1.66E-11	1.49E-10
KLMA_10770		hypothetical protein	10549.00849	-1.036111586	0.257178933	-4.028757613	5.61E-05	0.000176341
KLMA_10791	NOG1	nucleolar GTP-binding protein 1	2408.612156	-1.242491891	0.125973229	-9.863142387	6.01E-23	1.65E-21
KLMA_10832	URA1	dihydroorotate dehydrogenase	2949.733803	-2.939976881	0.168742333	-17.42287682	5.53E-68	1.59E-65
KLMA_20023	TPS2	trehalose-phosphatase	8094.628903	-1.071475723	0.161166816	-6.648240316	2.97E-11	2.56E-10
KLMA_20052		uncharacterized membrane protein YGR149W	538.5182779	-1.336557248	0.307551506	-4.345799717	1.39E-05	4.99E-05
KLMA_20054	RPL24	60S ribosomal protein L24	7955.338496	-1.102117706	0.213931268	-5.151737357	2.58E-07	1.25E-06
KLMA_20074	TSL1	trehalose synthase complex regulatory subunit TPS3	3506.881249	-1.504207374	0.153457654	-9.802100662	1.10E-22	2.96E-21
KLMA_20090		ras-related protein rapC	242.6519106	-1.06919232	0.190190731	-5.62168468	1.89E-08	1.10E-07
KLMA_20098	GPM1	phosphoglycerate mutase 1	34135.68355	-3.402956455	0.214844014	-15.83919601	1.67E-56	2.55E-54
KLMA_20107		jmjC super family	669.2382984	-1.270327818	0.159901055	-7.944461769	1.95E-15	2.75E-14
KLMA_20126	UTP13	U3 small nucleolar RNA-associated protein 13	822.633694	-1.174086402	0.18342692	-6.40084019	1.55E-10	1.21E-09
KLMA_20138	RPC37	DNA-directed RNA polymerase III subunit rpc5	354.7554812	-1.090769118	0.178978361	-6.094418958	1.10E-09	7.73E-09
KLMA_20139	DBP7	ATP-dependent RNA helicase DBP7	535.6351908	-1.317599917	0.157425612	-8.369666804	5.78E-17	9.53E-16
KLMA_20147	UTP15	U3 small nucleolar RNA-associated protein 15	844.0264385	-1.2804268	0.17974385	-7.123619543	1.05E-12	1.08E-11
KLMA_20157	HSL1	probable serine/threonine-protein kinase HSL1	1173.692509	-1.001189911	0.166301853	-6.020317239	1.74E-09	1.18E-08
KLMA_20158	ADH4	alcohol dehydrogenase 4	2997.662752	-2.936023481	0.63087214	-4.653912092	3.26E-06	1.33E-05
KLMA_20159	UTP11	U3 small nucleolar RNA-associated protein 11	198.2393025	-1.07633911	0.203154343	-5.298134871	1.17E-07	6.02E-07
KLMA_20165	ADK1	adenylate kinase 1	2572.406413	-1.308084245	0.193339052	-6.765752861	1.33E-11	1.20E-10
KLMA_20173	ARX1	probable metalloprotease ARX1	822.220041	-1.823384132	0.170328951	-10.70507464	9.64E-27	3.89E-25
KLMA_20186		conserved hypothetical protein	846.7099628	-1.515573244	0.340859546	-4.446327707	8.74E-06	3.26E-05

Locus_tag	UniProt _gene	Product	baseMean	log_2FoldChange	lfcSE	stat	pvalue	padj
KLMA_20192	NOP53	ribosome biogenesis protein NOP53	716.284206	-1.001363292	0.209191493	-4.786826077	1.69E-06	7.30E-06
KLMA_20239	RMD9	protein RMD9	7714.160346	-1.822888312	0.118354418	-15.40194562	1.59E-53	1.99E-51
KLMA_20268	RPS2	40S ribosomal protein S2	17354.0792	-1.106138147	0.271643009	-4.072028766	4.66E-05	0.000149652
KLMA_20276	MET13	methylenetetrahydrofolate reductase 2	1161.753648	-1.848996539	0.242017053	-7.639943198	2.17E-14	2.69E-13
KLMA_20288	CYS4	cystathionine beta-synthase	3781.141055	-1.059017729	0.217273349	-4.874126239	1.09E-06	4.83E-06
KLMA_20305	SHM2	serine hydroxymethyltransferase	12779.74086	-1.40802017	0.318385505	-4.422375229	9.76E-06	3.62E-05
KLMA_20308	ERG3	c-5 sterol desaturase	3994.822075	-2.350025826	0.184404233	-12.74388218	3.37E-37	2.32E-35
KLMA_20320	NOP58	nucleolar protein 58	4632.938909	-1.112204874	0.189728491	-5.862086747	4.57E-09	2.94E-08
KLMA_20323	SLY41	uncharacterized transporter SLY41	504.3726713	-1.045702615	0.144484308	-7.237482274	4.57E-13	4.96E-12
KLMA_20326	MCH5	riboflavin transporter MCH5	766.2728748	-1.126854228	0.244961468	-4.600128491	4.22E-06	1.68E-05
KLMA_20331	GAL7	galactose-1-phosphate uridylyltransferase	18497.94383	-1.80911301	0.157175713	-11.51013077	1.17E-30	5.85E-29
KLMA_20332	GAL10	bifunctional protein GAL10	41677.65387	-2.64693837	0.1526667	-17.33802047	2.43E-67	6.59E-65
KLMA_20333	GAL1	galactokinase	58630.29623	-1.767183527	0.167155082	-10.57211967	4.01E-26	1.56E-24
KLMA_20341	FHL1	pre-rRNA-processing protein FHL1	642.671196	-1.08550499	0.142091016	-7.639504733	2.18E-14	2.70E-13
KLMA_20354	CYS3	cystathionine gamma-lyase	3530.015886	-1.702415507	0.225747458	-7.541238878	4.66E-14	5.64E-13
KLMA_20355		60S acidic ribosomal protein P2-alpha	3098.008596	-1.018838116	0.267552291	-3.807996238	0.000140097	0.000406557
KLMA_20359	NOP12	nucleolar protein 12	726.4948091	-1.152400867	0.15440536	-7.463477098	8.43E-14	9.85E-13
KLMA_20374	CCC2	copper-transporting ATPase	1570.859465	-1.127814871	0.146436487	-7.701733992	1.34E-14	1.71E-13
KLMA_20375		SERF-like protein YDL085C-A	42.39041633	-1.176082281	0.32142361	-3.658979127	0.000253222	0.000689374
KLMA_20392		delta(12) fatty acid desaturase	1779.345069	-1.299013656	0.200102479	-6.491741941	8.48E-11	6.87E-10
KLMA_20411	ENP1	essential nuclear protein 1	788.440287	-1.550890202	0.1612715	-9.616641523	6.80E-22	1.69E-20
KLMA_20417	CLN2	G1/S-specific cyclin CLN2	2318.131598	-1.561326802	0.162464822	-9.610245379	7.24E-22	1.79E-20
KLMA_20429	UBP3	ubiquitin carboxyl-terminal hydrolase 3	983.2670094	-1.051630983	0.136759878	-7.689616271	1.48E-14	1.87E-13
KLMA_20478	CBF5	centromere/microtubule-binding protein CBF5	2484.101399	-1.442347896	0.170367375	-8.466103879	2.54E-17	4.36E-16
KLMA_20481		RFX-like DNA-binding protein RFX1	382.6192262	-1.550990371	0.178886251	-8.670260362	4.31E-18	8.10E-17

Locus_tag	UniProt_gene	Product	baseMean	log_2FoldChange	lfcSE	stat	pvalue	padj
KLMA_20482	RLP7	ribosome biogenesis protein RLP7	960.2710059	-1.169461129	0.161215197	-7.254037763	4.05E-13	4.43E-12
KLMA_20495	RPL22A	60S ribosomal protein L22-A	5880.589439	-1.008483955	0.272518312	-3.700609875	0.000215082	0.000596852
KLMA_20508	UTP8	U3 small nucleolar RNA-associated protein 8	881.1440257	-1.15158208	0.170010562	-6.773591405	1.26E-11	1.14E-10
KLMA_20514	TPA1	PKHD-type hydroxylase TPA1	1664.224497	-1.840706513	0.14015262	-13.13358617	2.11E-39	1.67E-37
KLMA_20527	ERG28	ergosterol biosynthetic protein 28	817.0077082	-1.076349804	0.228112853	-4.718496967	2.38E-06	9.90E-06
KLMA_20551	CYP707A7	lanosterol 14-alpha demethylase	4390.081646	-2.248096093	0.26870506	-8.366407731	5.94E-17	9.77E-16
KLMA_20555	TDA3	UPF0673 membrane protein YHR009C	1691.317538	-1.234722031	0.156230636	-7.90320045	2.72E-15	3.76E-14
KLMA_20591	ORT1	mitochondrial ornithine carrier protein	383.6747024	-1.064601725	0.195270051	-5.451945755	4.98E-08	2.73E-07
KLMA_20598	ADE2	phosphoribosylaminoimidazole carboxylase	2437.790869	-1.011415122	0.192585145	-5.251781615	1.51E-07	7.62E-07
KLMA_20619	YPR1	putative reductase 1	6119.972547	-1.424835339	0.157836111	-9.027308985	1.76E-19	3.69E-18
KLMA_20640	PET494	COX3 mRNA-specific translational activator PET494	298.0614566	-1.346240916	0.193345258	-6.962885607	3.33E-12	3.26E-11
KLMA_20682	MPP10	U3 small nucleolar RNA-associated protein MPP10	732.8990518	-1.175456754	0.200541231	-5.861421862	4.59E-09	2.95E-08
KLMA_20704	GAC1	CBM_21 super family	2229.471895	-1.089697847	0.16035681	-6.795457231	1.08E-11	9.93E-11
KLMA_20722	ERG9	squalene synthetase	1524.730457	-1.073329592	0.164012743	-6.544184145	5.98E-11	4.95E-10
KLMA_20724		non-structural maintenance of chromosome element 3	289.6123453	-1.033096314	0.196951131	-5.245444942	1.56E-07	7.87E-07
KLMA_20726	DTD1	D-tyrosyl-tRNA(Tyr) deacylase	248.7927933	-1.620871315	0.234780532	-6.903772225	5.06E-12	4.86E-11
KLMA_20739	IPI3	pre-rRNA-processing protein IPI3	543.5442432	-1.230722159	0.187826538	-6.552440204	5.66E-11	4.70E-10
KLMA_20742	RPS3	40S ribosomal protein S3	18707.71784	-1.209348225	0.213389013	-5.66734064	1.45E-08	8.58E-08
KLMA_20800	RPL5	60S ribosomal protein L5	16607.49874	-1.143255863	0.203990231	-5.6044638	2.09E-08	1.20E-07
KLMA_20810	NAN1	NET1-associated nuclear protein 1	1125.590175	-1.0791989	0.14019185	-7.698014528	1.38E-14	1.76E-13
KLMA_20829	PHO3	repressible acid phosphatase	212.4177213	-2.072868964	0.251559797	-8.240064528	1.72E-16	2.71E-15
KLMA_20830	LAC12	lactose permease	2951.356361	-1.068546837	0.295515535	-3.61587365	0.000299336	0.000799885
KLMA_30010	LAC12	lactose permease	87973.73377	-1.531320189	0.215348717	-7.110886058	1.15E-12	1.18E-11
KLMA_30041	LIA1	deoxyhypusine hydroxylase	1686.151989	-1.791583841	0.13694094	-13.08289432	4.12E-39	3.15E-37

Locus_tag	UniProt _gene	Product	baseMean	log_2FoldChange	lfcSE	stat	pvalue	padj
KLMA_30042	HAM1	protein HAM1	117.4065179	-1.218970438	0.235257202	-5.181437289	2.20E-07	1.08E-06
KLMA_30060	MRT4	mRNA turnover protein 4	1076.365305	-1.276581439	0.200050155	-6.381306934	1.76E-10	1.37E-09
KLMA_30073	MUP1	high-affinity methionine permease	4850.640315	-1.48926886	0.284808485	-5.229018591	1.70E-07	8.53E-07
KLMA_30074	RSC2	chromatin structure-remodeling complex subunit RSC2	946.7547179	-1.079305519	0.206935367	-5.215664844	1.83E-07	9.11E-07
KLMA_30083	RRS1	regulator of ribosome biosynthesis	545.3308817	-1.082068847	0.200740114	-5.39039671	7.03E-08	3.74E-07
KLMA_30099	PGM2	phosphoglucomutase-2	5880.12941	-2.578455332	0.151530137	-17.01612218	6.24E-65	1.38E-62
KLMA_30102	PMU1	uncharacterized protein YKL128C	200.0704808	-1.266567857	0.252099191	-5.024085378	5.06E-07	2.34E-06
KLMA_30119	CMC1	COX assembly mitochondrial protein	84.42747653	-1.047180325	0.337827607	-3.099747632	0.001936856	0.004311579
KLMA_30153	TEF3	elongation factor 3	47110.32692	-1.427915699	0.243184485	-5.871738474	4.31E-09	2.79E-08
KLMA_30154	GSH1	glutamate--cysteine ligase	1874.157265	-1.166272134	0.201179629	-5.797168134	6.74E-09	4.24E-08
KLMA_30158	PHS1	protein PHS1	401.8401563	-1.000033591	0.190843164	-5.240080749	1.61E-07	8.08E-07
KLMA_30190	IPI1	pre-rRNA-processing protein IPI1	144.5546233	-1.748560311	0.260685159	-6.707556039	1.98E-11	1.76E-10
KLMA_30199	RNR2	ribonucleoside-diphosphate reductase small chain 1	4227.229987	-1.641727582	0.137570151	-11.93374849	7.89E-33	4.24E-31
KLMA_30203	ATF1	alcohol O-acetyltransferase 1	756.5288467	-1.178988769	0.2055632	-5.735407738	9.73E-09	5.92E-08
KLMA_30204	ERG1	squalene monooxygenase	2894.071572	-2.532186591	0.277529151	-9.12403825	7.24E-20	1.57E-18
KLMA_30222	DCAF13	protein SOF1	483.3480997	-1.135962316	0.15170933	-7.487755131	7.01E-14	8.27E-13
KLMA_30225	MEU1	multicopy enhancer of UAS2	1514.957488	-1.005165319	0.200683553	-5.008707998	5.48E-07	2.53E-06
KLMA_30226		cytochrome c oxidase copper chaperone	99.2770965	-1.186117924	0.259083721	-4.578126016	4.69E-06	1.86E-05
KLMA_30229	DRS1	ATP-dependent RNA helicase DRS1	741.640094	-1.074469018	0.176084271	-6.102015869	1.05E-09	7.46E-09
KLMA_30237	MTH1	protein MTH1	3687.119688	-1.413858274	0.174415588	-8.10626099	5.22E-16	7.84E-15
KLMA_30249	ETT1	uncharacterized protein YOR051C	1211.130745	-2.199689104	0.209591232	-10.49513897	9.09E-26	3.27E-24
KLMA_30254	VHS3	protein VHS3	716.6044525	-1.024628019	0.148972753	-6.877955859	6.07E-12	5.74E-11
KLMA_30255	NOB1	20S-pre-rRNA D-site endonuclease NOB1	318.8515328	-1.370526364	0.181965009	-7.531812696	5.00E-14	6.02E-13
KLMA_30263		uncharacterized protein YKR075C	3946.516419	-1.949886075	0.131494533	-14.82864751	9.56E-50	9.94E-48
KLMA_30264	RPL3	60S ribosomal protein L3	29512.4949	-1.259745403	0.206617585	-6.096990267	1.08E-09	7.63E-09
KLMA_30274		fumarate reductase	4706.01433	-2.017987336	0.177751432	-11.35286122	7.18E-30	3.34E-28

Locus_tag	UniProt _gene	Product	baseMean	log_2FoldChange	lfcSE	stat	pvalue	padj
KLMA_30299	PAB1	polyadenylate-binding protein	4014.179959	-1.037142845	0.188373632	-5.505775056	3.68E-08	2.05E-07
KLMA_30301	RLI1	translation initiation factor RLI1	3815.999557	-1.087977997	0.30089278	-3.615832851	0.000299384	0.000799885
KLMA_30308	MRPL15	54S ribosomal protein L15	753.7958276	-1.027668205	0.23353062	-4.400571568	1.08E-05	3.96E-05
KLMA_30317		hypothetical protein	194.7154789	-1.658654357	0.223164978	-7.432413344	1.07E-13	1.23E-12
KLMA_30320		glyco_transf_15 super family protein	1474.659223	-1.102956907	0.125386699	-8.796442615	1.41E-18	2.74E-17
KLMA_30326	PIB2	phosphatidylinositol-3-phosphate-binding protein 2	1450.37963	-1.000146419	0.151777524	-6.589555496	4.41E-11	3.71E-10
KLMA_30370	GSY2	glycogen [starch] synthase isoform 2	5494.268558	-1.800426946	0.15724387	-11.44990228	2.35E-30	1.15E-28
KLMA_30372	PDC2	protein PDC2	785.4351139	-1.452556056	0.133037834	-10.91836823	9.42E-28	4.03E-26
KLMA_30380	ALK2	serine/threonine-protein kinase Haspin homolog	645.3653109	-1.052260483	0.261393842	-4.025574881	5.68E-05	0.000178544
KLMA_30382	NSR1	nuclear localization sequence-binding protein	1943.958007	-1.937465975	0.135152663	-14.33538882	1.32E-46	1.19E-44
KLMA_30430	SUR2	sphingolipid C4-hydroxylase SUR2	3712.363003	-1.673043223	0.241755816	-6.920384592	4.50E-12	4.34E-11
KLMA_30432	BFR2	protein BFR2	552.761611	-1.336631053	0.148867766	-8.978646565	2.74E-19	5.67E-18
KLMA_30457	MEX67	mRNA export factor MEX67	841.0828889	-1.109745678	0.150322272	-7.382443477	1.55E-13	1.78E-12
KLMA_30477	SER3	D-3-phosphoglycerate dehydrogenase 1	5482.837952	-1.375391904	0.242944798	-5.661335055	1.50E-08	8.86E-08
KLMA_30493	PWP2	periodic tryptophan protein 2	1021.304607	-1.122810265	0.180940961	-6.205395713	5.46E-10	4.00E-09
KLMA_30495	YIH1	protein IMPACT homolog	283.4447524	-1.162596855	0.184354306	-6.306317861	2.86E-10	2.14E-09
KLMA_30509	HNM1	choline transport protein	3011.90997	-1.390899115	0.299624103	-4.642146941	3.45E-06	1.40E-05
KLMA_30511	DBP3	ATP-dependent RNA helicase DBP3	986.7386386	-1.299219512	0.176568378	-7.358166414	1.86E-13	2.11E-12
KLMA_30545	PAN5	2-dehydropantoate 2-reductase	506.4747077	-1.217637594	0.172228261	-7.069905882	1.55E-12	1.56E-11
KLMA_30548	RRP3	ATP-dependent rRNA helicase RRP3	551.0694452	-1.138539395	0.1881501	-6.051229295	1.44E-09	9.89E-09
KLMA_30549	SSF1	ribosome biogenesis protein SSF2	703.3910784	-1.031816114	0.188258313	-5.480852864	4.23E-08	2.34E-07
KLMA_30552	TIF32	eukaryotic translation initiation factor 3 subunit A	3923.256932	-1.354467046	0.185030317	-7.320243901	2.48E-13	2.75E-12
KLMA_30576	HAL9	halotolerance protein 9	2141.53969	-1.117462847	0.148886846	-7.505450475	6.12E-14	7.27E-13
KLMA_30587	VTS1	protein VTS1	1266.428536	-1.115326268	0.191641488	-5.819858092	5.89E-09	3.74E-08
KLMA_30608	SCW4	probable family 17 glucosidase SCW10	841.0897549	-1.177969609	0.198809601	-5.9251143	3.12E-09	2.06E-08

Locus_tag	UniProt _gene	Product	baseMean	log_2FoldChange	lfcSE	stat	pvalue	padj
KLMA_30614		hypothetical protein	2705.394871	-2.309608643	0.155990082	-14.80612493	1.34E-49	1.36E-47
KLMA_30615	RAD54	DNA repair and recombination protein RAD54	523.3188553	-1.113129404	0.158668099	-7.015458139	2.29E-12	2.26E-11
KLMA_30639	KSS1	mitogen-activated protein kinase KSS1	397.3341351	-1.265497808	0.164756414	-7.681023028	1.58E-14	1.99E-13
KLMA_30667	HKR1	herpes_gp2	1129.019862	-1.250665898	0.149171219	-8.384096525	5.11E-17	8.49E-16
KLMA_30671	ERG6	sterol 24-C-methyltransferase	1180.449648	-1.567822167	0.199873847	-7.844058604	4.36E-15	5.93E-14
KLMA_30687	SAH1	adenosylhomocysteinase	21362.46627	-1.085736862	0.307765516	-3.527805446	0.00041902	0.001078237
KLMA_30695	DUG1	cys-Gly metallodipeptidase DUG1	2219.749609	-1.428077942	0.158080635	-9.033857552	1.66E-19	3.52E-18
KLMA_30697	ADE5,7	bifunctional purine biosynthetic protein ADE5	8831.68422	-1.084240679	0.217316477	-4.989224434	6.06E-07	2.77E-06
KLMA_30698	MTO1	mitochondrial translation optimization protein 1	510.6959817	-1.010649563	0.151902426	-6.653281263	2.87E-11	2.48E-10
KLMA_30709	GUS1	glutamyl-tRNA synthetase	5844.806784	-1.222477478	0.166475288	-7.343296979	2.08E-13	2.34E-12
KLMA_30715		LCB5	1670.946983	-1.38565146	0.293371501	-4.723197235	2.32E-06	9.69E-06
KLMA_30717		uncharacterized protein YKR096W	3633.943524	-1.556861589	0.124530727	-12.50182687	7.30E-36	4.57E-34
KLMA_40014	BRE5	UBP3-associated protein BRE5	1660.215085	-1.22800495	0.157859186	-7.779116186	7.30E-15	9.69E-14
KLMA_40019	YCT1	uncharacterized transporter YLL055W	776.352917	-1.099552374	0.369693879	-2.974223907	0.002937307	0.006314175
KLMA_40064	RRB1	ribosome assembly protein RRB1	1126.08915	-1.389375542	0.18353406	-7.570123727	3.73E-14	4.55E-13
KLMA_40072	ECM16	probable ATP-dependent RNA helicase DHR1	465.1122678	-1.035697279	0.17073282	-6.06618739	1.31E-09	9.09E-09
KLMA_40080	RPL15B	60S ribosomal protein L15-B	7543.379505	-1.358425085	0.211284816	-6.429354987	1.28E-10	1.01E-09
KLMA_40102	ADH1	alcohol dehydrogenase 1	81946.03565	-1.065723927	0.20492703	-5.200504436	1.99E-07	9.81E-07
KLMA_40111	dsd1	dihydroceramide delta(4)-desaturase	790.6514724	-1.543497167	0.248915257	-6.20089417	5.61E-10	4.10E-09
KLMA_40115	HEM13	coproporphyrinogen-III oxidase	1507.255044	-1.976950314	0.248634657	-7.951225845	1.85E-15	2.62E-14
KLMA_40123	BAP3	valine amino-acid permease	3547.357009	-2.478077914	0.321375815	-7.710841316	1.25E-14	1.61E-13
KLMA_40125	TPI1	triosephosphate isomerase	20614.28544	-2.799656701	0.185124719	-15.12308414	1.14E-51	1.27E-49
KLMA_40132		transcription activator	931.4865252	-1.507910508	0.143332191	-10.52038974	6.96E-26	2.59E-24
KLMA_40147	SUL2	sulfate permease 2	9165.907466	-1.096250976	0.221883932	-4.940650583	7.79E-07	3.53E-06
KLMA_40149		hypothetical protein	4127.869111	-1.548204097	0.159455575	-9.70931307	2.75E-22	7.07E-21

Locus_tag	UniProt _gene	Product	baseMean	log_2FoldChange	lfcSE	stat	pvalue	padj
KLMA_40153	fmo1	thiol-specific monooxygenase	742.0892315	-1.560386616	0.172554643	-9.042855006	1.53E-19	3.26E-18
KLMA_40168	MDN1	midasin	2167.566691	-1.498092464	0.191989395	-7.802995899	6.05E-15	8.13E-14
KLMA_40194	LCB5	sphingoid long chain base kinase 5	1645.028382	-1.103232002	0.156505506	-7.049157738	1.80E-12	1.80E-11
KLMA_40209	GCR2	hypothetical protein	375.8710421	-1.578624279	0.176020271	-8.968423205	3.01E-19	6.20E-18
KLMA_40218	GAP1	glyceraldehyde-3-phosphate dehydrogenase 1	79994.31241	-2.957491564	0.162016095	-18.25430721	1.91E-74	6.23E-72
KLMA_40220	ADH2	alcohol dehydrogenase 2	50497.17099	-7.380279973	0.306526555	-24.07713081	4.34E-128	5.30E-125
KLMA_40225	RIB7	5-amino-6-(5-phosphoribosylamino)uracil reductase	6676.337237	-1.695942535	0.202613213	-8.370345185	5.74E-17	9.51E-16
KLMA_40226	SPP381	pre-mRNA-splicing factor SPP381	558.9371746	-1.974767274	0.16240069	-12.15984536	5.09E-34	2.85E-32
KLMA_40228	UTP10	U3 small nucleolar RNA-associated protein 10	2047.662215	-1.46033005	0.172942777	-8.444007199	3.07E-17	5.22E-16
KLMA_40240	GAR1	H/ACA ribonucleoprotein complex subunit 1	1296.711556	-1.169132179	0.259083889	-4.512562265	6.40E-06	2.47E-05
KLMA_40251	CIC1	proteasome-interacting protein CIC1	939.4831471	-1.05964129	0.195805476	-5.411704062	6.24E-08	3.37E-07
KLMA_40254		calcipressin-like protein	87.82736266	-1.124491215	0.238781582	-4.709287897	2.49E-06	1.04E-05
KLMA_40263	SFB3	SED5-binding protein 3	771.5881702	-1.309488478	0.208425109	-6.282776984	3.33E-10	2.46E-09
KLMA_40297	HAP1	hap1 transcriptional regulatory prottein	2425.739774	-2.470003276	0.15466934	-15.96957269	2.08E-57	3.28E-55
KLMA_40302	ERB1	ribosome biogenesis protein ERB1	1005.189181	-1.297714851	0.156867073	-8.27270392	1.31E-16	2.09E-15
KLMA_40304		hypothetical protein	55.99621093	-1.605992477	0.35214786	-4.560562928	5.10E-06	2.00E-05
KLMA_40305	NUP116	nucleoporin NUP116/NSP116	2618.183003	-1.089746463	0.145582404	-7.485427025	7.13E-14	8.39E-13
KLMA_40307	MCM1	pheromone receptor transcription factor	609.8824689	-1.294360272	0.151048126	-8.569191182	1.04E-17	1.85E-16
KLMA_40308		hypothetical protein	6525.206016	-1.456327909	0.255131432	-5.708147742	1.14E-08	6.90E-08
KLMA_40318	IMP2	mitochondrial inner membrane protease subunit 2	71.46757159	-1.163903123	0.276977287	-4.202160892	2.64E-05	8.99E-05
KLMA_40328	RRP12	ribosomal RNA-processing protein 12	1719.035853	-1.354612164	0.129336573	-10.47354302	1.14E-25	4.07E-24
KLMA_40374	MKS1	negative regulator of RAS-cAMP pathway	887.6796643	-1.129273505	0.175375797	-6.439163907	1.20E-10	9.54E-10

Locus_tag	UniProt _gene	Product	baseMean	log_2FoldChange	lfcSE	stat	pvalue	padj
KLMA_40375	IMP4	U3 small nucleolar ribonucleoprotein protein IMP4	216.8980256	-1.446357648	0.189742827	-7.622726353	2.48E-14	3.06E-13
KLMA_40378	FMS1	polyamine oxidase FMS1	431.6987648	-1.39488125	0.175379094	-7.953520667	1.81E-15	2.59E-14
KLMA_40388	FBA1	fructose-bisphosphate aldolase	91591.73868	-2.802818706	0.25675687	-10.91623645	9.64E-28	4.09E-26
KLMA_40390	TRM13	tRNA guanosine-2'-O-methyltransferase TRM13	328.0503889	-1.029068786	0.1754717	-5.864585492	4.50E-09	2.91E-08
KLMA_40404	ALD5	aldehyde dehydrogenase 5	1284.147401	-3.40219444	0.639350295	-5.321330834	1.03E-07	5.35E-07
KLMA_40407	MAM33	mitochondrial acidic protein MAM33	787.0926995	-1.165864507	0.174149862	-6.694604838	2.16E-11	1.91E-10
KLMA_40415	HRK1	serine/threonine-protein kinase HRK1	2406.995409	-1.598756978	0.16666065	-9.592888194	8.57E-22	2.09E-20
KLMA_40422	YTM1	ribosome biogenesis protein YTM1	1000.37013	-1.19423701	0.187499102	-6.369294546	1.90E-10	1.47E-09
KLMA_40431	NOP19	uncharacterized protein YGR251W	98.80625074	-1.008840348	0.265631942	-3.797887942	0.000145934	0.000421741
KLMA_40432	NMD3	60S ribosomal export protein NMD3	1146.606766	-1.298663145	0.13760862	-9.437367669	3.82E-21	8.93E-20
KLMA_40444	FPR3	FK506-binding protein 3	2211.456146	-1.0648629	0.169979345	-6.264660561	3.74E-10	2.76E-09
KLMA_40445	HMG1	3-hydroxy-3-methylglutaryl-coenzyme A reductase 1	4589.558944	-1.605187442	0.265961455	-6.035413819	1.59E-09	1.08E-08
KLMA_40457	SFP1	zinc finger protein SFP1	1234.045656	-1.980675465	0.12936137	-15.31118188	6.44E-53	7.67E-51
KLMA_40458	DUS3	tRNA-dihydrouridine synthase 3	374.9667423	-1.092107384	0.166199847	-6.57104927	5.00E-11	4.17E-10
KLMA_40471	DPM1	dolichol-phosphate mannosyltransferase	1709.667323	-1.022302422	0.184582757	-5.538450278	3.05E-08	1.73E-07
KLMA_40478	RPC82	DNA-directed RNA polymerase III subunit RPC3	722.192777	-1.455972716	0.176939092	-8.228666169	1.89E-16	2.97E-15
KLMA_40494	MET5	sulfite reductase [NADPH] subunit beta	10826.06949	-1.102703765	0.221612022	-4.97583007	6.50E-07	2.97E-06
KLMA_40500	RIX1	pre-rRNA-processing protein RIX1	1080.23942	-1.022740821	0.130866394	-7.815152442	5.49E-15	7.41E-14
KLMA_40501	SGM1	protein SGM1	756.9239091	-1.051266257	0.17477664	-6.01491285	1.80E-09	1.22E-08
KLMA_40502	XPT1	xanthine phosphoribosyltransferase 1	1025.066076	-1.407011214	0.171430505	-8.207472826	2.26E-16	3.50E-15
KLMA_40522	UTP21	U3 small nucleolar RNA-associated protein 21	1111.446385	-1.333177648	0.151422641	-8.804348118	1.32E-18	2.57E-17
KLMA_40526	FAS1	fatty acid synthase subunit beta	19115.91845	-1.249140895	0.254181053	-4.914374537	8.91E-07	3.99E-06
KLMA_40530	MTR2	mRNA transport regulator MTR2	132.0623085	-1.019663229	0.211585791	-4.819147946	1.44E-06	6.27E-06
KLMA_40531		uncharacterized protein YKL187C	16272.32995	-1.152051786	0.145633588	-7.9106187	2.56E-15	3.57E-14

Locus_tag	UniProt _gene	Product	baseMean	log_2FoldChange	lfcSE	stat	pvalue	padj
KLMA_40547	PUS1	tRNA pseudouridine synthase 1	622.409884	-1.043241684	0.144150949	-7.237147519	4.58E-13	4.96E-12
KLMA_40555	BMS1	ribosome biogenesis protein BMS1	1421.406084	-1.40248081	0.135534876	-10.34774849	4.28E-25	1.46E-23
KLMA_40558	RPL10A	60S ribosomal protein L10a	13500.34226	-1.040946855	0.241317147	-4.31360502	1.61E-05	5.71E-05
KLMA_40571	GUK1	guanylate kinase	706.2789182	-1.338869942	0.188059144	-7.11940889	1.08E-12	1.11E-11
KLMA_40579	UTP6	U3 small nucleolar RNA-associated protein 6	515.6879526	-1.025770094	0.15013078	-6.832510261	8.34E-12	7.76E-11
KLMA_40582		hypothetical protein	162.991639	-1.887893409	0.334415959	-5.645344844	1.65E-08	9.64E-08
KLMA_40583	DLD1	D-lactate dehydrogenase [cytochrome] 1	6837.124499	-4.866943488	0.261004985	-18.64693691	1.34E-77	5.02E-75
KLMA_40593		uncharacterized vacuolar membrane protein YML018C	1590.804891	-1.267526563	0.192406124	-6.587766216	4.46E-11	3.75E-10
KLMA_40619	PRP43	pre-mRNA-splicing factor ATP-dependent RNA helicase PRP43	1466.469287	-1.149542136	0.148608974	-7.735348019	1.03E-14	1.35E-13
KLMA_40626		flocculation protein FLO9	477.624138	-1.352354868	0.25208115	-5.364759987	8.11E-08	4.28E-07
KLMA_50044	MEI5	meiosis protein 5	23.51674743	-1.383361484	0.410668126	-3.368563067	0.000755611	0.001842439
KLMA_50045	NOC2	nucleolar complex protein 2	1229.578738	-1.283276784	0.160400617	-8.000447947	1.24E-15	1.81E-14
KLMA_50046	GEP3	mitochondrial protein FMP38	402.3799288	-1.021662346	0.168101626	-6.077647007	1.22E-09	8.50E-09
KLMA_50050	SHM1	serine hydroxymethyltransferase	2104.251634	-1.357562564	0.142648204	-9.516857016	1.78E-21	4.27E-20
KLMA_50057	MTR4	ATP-dependent RNA helicase DOB1	1306.473971	-1.120418857	0.130495244	-8.585898001	9.01E-18	1.63E-16
KLMA_50085	UTP18	U3 small nucleolar RNA-associated protein 18	599.6877603	-1.064039481	0.163698249	-6.500005251	8.03E-11	6.54E-10
KLMA_50101		SWIRM domain-containing protein YOR338W	3371.929078	-1.296985686	0.231354495	-5.60605355	2.07E-08	1.19E-07
KLMA_50120	LTE1	guanine nucleotide exchange factor LTE1	1115.212092	-1.013011182	0.140607653	-7.204523782	5.82E-13	6.21E-12
KLMA_50125	UPC2	sterol regulatory element-binding protein ECM22	1352.986883	-2.439527324	0.209409852	-11.64953466	2.31E-31	1.19E-29
KLMA_50140	ENP2	ribosome biogenesis protein ENP2	871.4643968	-1.075700895	0.137040935	-7.849485941	4.18E-15	5.70E-14
KLMA_50142	TIF4632	eukaryotic initiation factor 4F subunit p150	2382.853542	-1.19253123	0.154306705	-7.728317638	1.09E-14	1.42E-13
KLMA_50154	LEU1	3-isopropylmalate dehydratase	3740.246868	-1.322640396	0.231612543	-5.710573256	1.13E-08	6.81E-08
KLMA_50183	IMD4	inosine-5'-monophosphate dehydrogenase	15103.02929	-1.912303401	0.177359213	-10.78209226	4.18E-27	1.73E-25

Locus_tag	UniProt _gene	Product	baseMean	log_2FoldChange	lfcSE	stat	pvalue	padj
KLMA_50206	ERV1	mitochondrial FAD-linked sulfhydryl oxidase ERV1	285.0978943	-2.358489781	0.224372847	-10.51147592	7.65E-26	2.81E-24
KLMA_50214	RPA135	DNA-directed RNA polymerase I subunit RPA2	3413.501695	-1.420041402	0.153389408	-9.25775398	2.09E-20	4.70E-19
KLMA_50215	CWP1	cell wall protein CWP1	16353.96716	-1.253562573	0.216738359	-5.783759636	7.30E-09	4.56E-08
KLMA_50217	YJU3	serine hydrolase YJU3	1305.069786	-1.248466944	0.30519521	-4.090716048	4.30E-05	0.000139019
KLMA_50218		protein MBR1	2283.272964	-1.54705675	0.152495333	-10.14494492	3.49E-24	1.11E-22
KLMA_50222	PTK2	serine/threonine-protein kinase PTK2	2246.401425	-1.263268263	0.182986901	-6.90359942	5.07E-12	4.86E-11
KLMA_50241	PDX3	pyridoxamine 5'-phosphate oxidase	902.9108208	-1.34913441	0.248342749	-5.432550042	5.56E-08	3.03E-07
KLMA_50255	PIR3	cell wall mannoprotein HSP150	3724.245338	-1.002485454	0.260915605	-3.842182816	0.000121945	0.000359867
KLMA_50260	CCP1	cytochrome c peroxidase	5877.969183	-2.322097237	0.149542603	-15.52799802	2.24E-54	2.96E-52
KLMA_50264	RPS14	40S ribosomal protein S14	9285.488798	-1.106511556	0.245162498	-4.513380161	6.38E-06	2.46E-05
KLMA_50271	SUR4	elongation of fatty acids protein 3	2077.042137	-1.085853726	0.255355386	-4.252323572	2.12E-05	7.35E-05
KLMA_50278	VHT1	vitamin H transporter	2154.221029	-1.325611467	0.275038602	-4.819728794	1.44E-06	6.26E-06
KLMA_50283	ADE6	phosphoribosylformylglycinamidine synthase	5738.764521	-1.090986549	0.246549645	-4.425017728	9.64E-06	3.58E-05
KLMA_50284	ERG25	c-4 methylsterol oxidase	3103.621139	-1.742888257	0.241870073	-7.205886338	5.77E-13	6.16E-12
KLMA_50291	RRP5	rRNA biogenesis protein RRP5	2499.509234	-1.398351869	0.145531776	-9.608567347	7.36E-22	1.81E-20
KLMA_50309	PWP1	periodic tryptophan protein 1	872.7385649	-1.071654215	0.163806617	-6.542191246	6.06E-11	5.01E-10
KLMA_50311	NOP56	nucleolar protein 56	3558.350424	-1.262897163	0.176574901	-7.152189541	8.54E-13	8.95E-12
KLMA_50319	MSS51	protein MSS51	1359.558942	-1.70765213	0.134688962	-12.67848608	7.78E-37	5.21E-35
KLMA_50330	LAC9	lactose regulatory protein LAC9	2043.665205	-1.097489961	0.180037175	-6.095907465	1.09E-09	7.67E-09
KLMA_50332	SEO1	probable transporter SEO1	2417.144937	-1.498720882	0.260676022	-5.74936225	8.96E-09	5.49E-08
KLMA_50338		uncharacterized protein YPL245W	172.7661419	-2.10588339	0.20782874	-10.13278235	3.95E-24	1.22E-22
KLMA_50349	APE2	aminopeptidase 2	1360.596304	-1.133952433	0.299499218	-3.786161586	0.000152992	0.000439538
KLMA_50356	UTP4	U3 small nucleolar RNA-associated protein 4	1060.326827	-1.411058996	0.168786129	-8.36004121	6.27E-17	1.02E-15
KLMA_50360	KHT2	hexose transporter 2	10182.55276	-3.220038628	0.331270678	-9.720264542	2.47E-22	6.39E-21
KLMA_50361	KHT2	hexose transporter 2	15464.67737	-2.796062059	0.760807846	-3.675122536	0.000237735	0.000652303
KLMA_50362	KHT2	hexose transporter	24030.04473	-5.022981045	0.608670376	-8.25238297	1.55E-16	2.45E-15

Locus_tag	UniProt_gene	Product	baseMean	log_2FoldChange	lfcSE	stat	pvalue	padj
KLMA_50363	RAG1	low-affinity glucose transporter	240.6783052	-2.40408063	0.298403574	-8.056473974	7.85E-16	1.17E-14
KLMA_50378	NUG1	nuclear GTP-binding protein NUG1	642.4116782	-1.074590926	0.140082322	-7.671138721	1.70E-14	2.14E-13
KLMA_50397		gti1_Pac2 super family conserved domain	253.3403098	-1.722175672	0.231122354	-7.451359184	9.24E-14	1.07E-12
KLMA_50409	MUC1	flo11 super family	19168.46235	-4.430898567	0.305647358	-14.49676711	1.27E-47	1.17E-45
KLMA_50410		bZIP_1 super family conserved domain	125.1631431	-1.020489717	0.253564021	-4.02458406	5.71E-05	0.000179151
KLMA_50422	NNF2	protein NNF2	519.3261046	-1.068445224	0.158247602	-6.751730911	1.46E-11	1.32E-10
KLMA_50426	RPC40	DNA-directed RNA polymerases I and III subunit RPAC1	829.0243151	-1.544520579	0.254754552	-6.062779124	1.34E-09	9.27E-09
KLMA_50431	VAS1	valyl-tRNA synthetase	1963.149986	-1.449744141	0.206296823	-7.027467114	2.10E-12	2.08E-11
KLMA_50445	NOP7	pescadillo homolog	1649.95709	-1.384501287	0.146559399	-9.446690508	3.50E-21	8.21E-20
KLMA_50471	MET10	sulfite reductase [NADPH] flavoprotein component	7792.702245	-1.318452071	0.252144002	-5.228964641	1.70E-07	8.53E-07
KLMA_50473	RPL2	60S ribosomal protein L2	29029.77255	-1.045299017	0.248634797	-4.204154168	2.62E-05	8.93E-05
KLMA_50487	DIP2	U3 small nucleolar RNA-associated protein 12	1228.939622	-1.184270796	0.179443891	-6.599671837	4.12E-11	3.50E-10
KLMA_50502		DNA-directed RNA polymerase I subunit RPA14	484.9145812	-1.531957261	0.198758128	-7.707645849	1.28E-14	1.65E-13
KLMA_50506	NHA1	Na(+)/H(+) antiporter	2136.545811	-1.109078781	0.1266027	-8.760309093	1.95E-18	3.74E-17
KLMA_50531		uncharacterized transporter YLR152C	2677.814568	-1.550404412	0.147561122	-10.50686243	8.03E-26	2.93E-24
KLMA_50533	ACS2	acetyl-coenzyme A synthetase 2	7877.173596	-1.702106598	0.175345773	-9.707143624	2.81E-22	7.19E-21
KLMA_50566	MAP1	methionine aminopeptidase 1	1135.031558	-1.060938252	0.132152609	-8.028129471	9.90E-16	1.46E-14
KLMA_50596	BRE4	protein BRE4	2037.68916	-1.49747656	0.15199393	-9.852212925	6.71E-23	1.83E-21
KLMA_60007		flocculation protein FLO9	2449.368093	-2.76462293	0.228847343	-12.08064249	1.34E-33	7.25E-32
KLMA_60018	UTH1	protein UTH1	1050.848115	-1.345407366	0.287814096	-4.674570798	2.95E-06	1.21E-05
KLMA_60019	SHB17	uncharacterized protein YKR043C	440.2982211	-1.320130907	0.224204477	-5.88806667	3.91E-09	2.55E-08
KLMA_60025	ALB1	ribosome biogenesis protein ALB1	365.8017339	-1.061805093	0.153000678	-6.939871796	3.92E-12	3.80E-11
KLMA_60037	URA2	protein URA1	11253.01147	-1.548349037	0.284604292	-5.440357294	5.32E-08	2.90E-07
KLMA_60042	PEX21	peroxisomal membrane protein PEX21	513.2626469	-1.239972572	0.251640497	-4.927555722	8.33E-07	3.75E-06

Locus_tag	UniProt _gene	Product	baseMean	log_2FoldChange	lfcSE	stat	pvalue	padj
KLMA_60062	IMP3	U3 small nucleolar ribonucleoprotein protein IMP3	193.8961987	-1.046636306	0.189691696	-5.517565218	3.44E-08	1.93E-07
KLMA_60069		40S ribosomal protein S0	13481.65401	-1.116406235	0.276069861	-4.043926521	5.26E-05	0.000166701
KLMA_60075	PDC1	pyruvate decarboxylase	70134.64616	-2.354794995	0.22350894	-10.53557408	5.92E-26	2.24E-24
KLMA_60087	ADE3	c-1-tetrahydrofolate synthase	5190.121043	-1.560267173	0.203917101	-7.651477804	1.99E-14	2.48E-13
KLMA_60096	EXO1	exodeoxyribonuclease 1	284.8749108	-1.261528833	0.223919734	-5.633843934	1.76E-08	1.02E-07
KLMA_60097	HMS1	probable transcription factor HMS1	3721.520346	-1.375055343	0.138811884	-9.905890649	3.92E-23	1.10E-21
KLMA_60131	IPP1	inorganic pyrophosphatase	8932.030189	-1.387254156	0.176170095	-7.874515562	3.42E-15	4.71E-14
KLMA_60135	MDM20	N-terminal acetyltransferase B complex subunit MDM20	1102.137603	-1.488116959	0.161470405	-9.216035364	3.08E-20	6.91E-19
KLMA_60136	BRX1	ribosome biogenesis protein BRX1	607.6967641	-1.021315733	0.155473642	-6.569060339	5.06E-11	4.22E-10
KLMA_60153	URB1	nucleolar pre-ribosomal-associated protein 1	1057.301024	-1.067148704	0.141556763	-7.538662807	4.75E-14	5.74E-13
KLMA_60167	MDH1	malate dehydrogenase	6488.853334	-1.789701489	0.187723564	-9.533707198	1.52E-21	3.67E-20
KLMA_60173	TEF4	eukaryotic elongation factor 1Bgamma	3798.2559	-1.059360534	0.234518496	-4.517172644	6.27E-06	2.42E-05
KLMA_60179	DHR2	probable ATP-dependent RNA helicase DHR2	178.843765	-1.205987767	0.219068156	-5.505080201	3.69E-08	2.06E-07
KLMA_60187	FET3	iron transport multicopper oxidase FET3	12971.78769	-1.775864714	0.312558562	-5.681702352	1.33E-08	7.95E-08
KLMA_60209	RPA190	DNA-directed RNA polymerase I subunit RPA1	2986.544354	-1.495281641	0.141908828	-10.53691771	5.84E-26	2.23E-24
KLMA_60210	FUN12	eukaryotic translation initiation factor 5B	3047.897413	-1.067840469	0.162071885	-6.588684192	4.44E-11	3.73E-10
KLMA_60214		uncharacterized protein YOR342C	1558.912478	-3.325300092	0.163135959	-20.38361199	2.34E-92	1.63E-89
KLMA_60217	TYE7	hypothetical protein	1268.312248	-2.100294884	0.14733166	-14.25555705	4.14E-46	3.68E-44
KLMA_60218	REV1	DNA repair protein REV1	880.1147264	-1.791842912	0.178874779	-10.01730329	1.28E-23	3.76E-22
KLMA_60219	PYK1	pyruvate kinase	62631.949	-3.138032503	0.244289871	-12.8455285	9.11E-38	6.45E-36
KLMA_60226	LEU4	2-isopropylmalate synthase	2923.937904	-1.325423031	0.210411806	-6.299185672	2.99E-10	2.24E-09
KLMA_60229	YAF9	protein AF-9 homolog	599.9116869	-1.296188292	0.154457093	-8.391898788	4.78E-17	7.97E-16
KLMA_60230	TFC7	transcription factor tau 55 kDa subunit	2953.072485	-2.127709408	0.168702323	-12.61221172	1.81E-36	1.16E-34

Locus_tag	UniProt _gene	Product	baseMean	log_2FoldChange	lfcSE	stat	pvalue	padj
KLMA_60232	NOP15	ribosome biogenesis protein 15	561.2658671	-1.221348812	0.152733374	-7.996607298	1.28E-15	1.86E-14
KLMA_60233	CYB5	cytochrome b5	1758.768459	-1.253166482	0.264133266	-4.744447758	2.09E-06	8.86E-06
KLMA_60234	CEX1	uncharacterized protein YOR112W	581.2447081	-1.188779034	0.22013767	-5.400161795	6.66E-08	3.57E-07
KLMA_60237	AZF1	zf-C2H2 super family protein	1995.055303	-1.147186684	0.135714041	-8.452969786	2.84E-17	4.85E-16
KLMA_60265	KRE33	UPF0202 protein YNL132W	1184.74556	-1.368539653	0.138089501	-9.910526432	3.75E-23	1.06E-21
KLMA_60297	EPL1	enhancer of polycomb-like protein 1	1240.953437	-1.211387797	0.156890419	-7.721235018	1.15E-14	1.49E-13
KLMA_60303	FCF2	rRNA-processing protein FCF2	217.8000483	-1.021466876	0.222529223	-4.590259479	4.43E-06	1.76E-05
KLMA_60313		40S ribosomal protein S20	9926.39281	-1.255292212	0.235871493	-5.321932698	1.03E-07	5.34E-07
KLMA_60316	RGT1	uncharacterized transcriptional regulatory protein YKL038W	2884.430991	-1.384508985	0.14614266	-9.473681321	2.70E-21	6.40E-20
KLMA_60317	UGP1	UTP--glucose-1-phosphate uridylyltransferase	4721.539652	-1.827546577	0.16367633	-11.16561314	6.01E-29	2.69E-27
KLMA_60380	TRS23	transport protein particle 23 kDa subunit	212.5084734	-2.647890935	0.310241053	-8.534946975	1.40E-17	2.47E-16
KLMA_60381	SKS1	serine/threonine-protein kinase SKS1	5467.705939	-2.947323726	0.172485033	-17.08741724	1.84E-65	4.28E-63
KLMA_60396	NOP4	nucleolar protein 4	822.2946629	-1.098765106	0.194684367	-5.643828132	1.66E-08	9.72E-08
KLMA_60402	PDAT9	pisatin demethylase	499.3968993	-1.013227487	0.29857722	-3.393519066	0.000690008	0.001698587
KLMA_60412	RAG5	hexokinase	21510.28912	-3.871007849	0.194914453	-19.86003499	9.02E-88	4.41E-85
KLMA_60417	cyp524A1	cytochrome P450 61	5650.434244	-1.885231252	0.28385234	-6.641591366	3.10E-11	2.65E-10
KLMA_60433	JHD2	histone demethylase JHD2	796.5880846	-1.08935628	0.201196357	-5.414393675	6.15E-08	3.32E-07
KLMA_60441	LEU3	regulatory protein LEU3	829.867322	-1.78583097	0.175320008	-10.18612191	2.29E-24	7.35E-23
KLMA_60445	ERG13	hydroxymethylglutaryl-CoA synthase	2605.020566	-1.352999692	0.213144031	-6.347818823	2.18E-10	1.67E-09
KLMA_60461	PFK2	6-phosphofructokinase subunit beta	24633.73479	-2.319491955	0.228912093	-10.13267549	3.96E-24	1.22E-22
KLMA_60468	SMM1	tRNA-dihydrouridine synthase 2	424.1595733	-1.270160688	0.190108596	-6.681237522	2.37E-11	2.07E-10
KLMA_60512	RPB1	DNA-directed RNA polymerase II subunit RPB1	5712.972867	-1.092447659	0.168620536	-6.478734341	9.25E-11	7.44E-10
KLMA_70017	CDC60	leucyl-tRNA synthetase	5293.152312	-1.498952376	0.161738376	-9.267759529	1.90E-20	4.30E-19
KLMA_70048	PET309	pentatricopeptide repeat-containing protein PET309	904.8465916	-1.122732748	0.149002264	-7.535004632	4.88E-14	5.89E-13
KLMA_70066		conserved hypothetical membrane	93.99167192	-1.340977055	0.289685297	-4.629082214	3.67E-06	1.48E-05

Locus_tag	UniProt _gene	Product	baseMean	log_2FoldChange	lfcSE	stat	pvalue	padj
		protein						
KLMA_70073	IKI3	elongator complex protein 1	1116.034609	-1.009606656	0.175516982	-5.75218787	8.81E-09	5.42E-08
KLMA_70080		conserved hypothetical protein	1856.110577	-1.235649067	0.126529585	-9.765692889	1.58E-22	4.19E-21
KLMA_70083	FUI1	uridine permease	2053.661711	-2.235681048	0.212683193	-10.51178994	7.62E-26	2.81E-24
KLMA_70086	URA7	CTP synthase	2850.452886	-1.470130299	0.157501266	-9.334085607	1.02E-20	2.35E-19
KLMA_70105	CFL1	probable ferric reductase transmembrane component	1751.481023	-1.424830545	0.34276557	-4.156866007	3.23E-05	0.000107197
KLMA_70126	AIM24	uncharacterized protein YJR080C	833.1502164	-1.452409783	0.146943486	-9.884138627	4.88E-23	1.36E-21
KLMA_70148	ROX3	mediator of RNA polymerase II transcription subunit 19	784.8277553	-1.164196514	0.1329762	-8.75492389	2.04E-18	3.91E-17
KLMA_70170	RNR1	ribonucleoside-diphosphate reductase large chain 1	5553.962551	-1.612426074	0.18063386	-8.926488506	4.40E-19	8.95E-18
KLMA_70180	VHR1	transcription factor VHR1	600.9472937	-1.241325941	0.146246789	-8.487885115	2.10E-17	3.66E-16
KLMA_70183	RHR2	(DL)-glycerol-3-phosphatase 1	5769.616889	-2.547286043	0.193234519	-13.1823551	1.11E-39	8.88E-38
KLMA_70186	FCY2	purine-cytosine permease FCY2	7677.443189	-1.919161867	0.198095773	-9.688050549	3.39E-22	8.62E-21
KLMA_70187	PCL6	PHO85 cyclin-6	725.327599	-1.181389348	0.142806945	-8.272632321	1.31E-16	2.09E-15
KLMA_70197	RSA4	WD repeat-containing protein YCR072C	799.3583154	-1.101900468	0.187073642	-5.890196276	3.86E-09	2.52E-08
KLMA_70212	HSL7	protein arginine N-methyltransferase HSL7	366.0373977	-1.037150693	0.214077875	-4.844735555	1.27E-06	5.56E-06
KLMA_70219	FAS2	fatty acid synthase subunit alpha	9564.457747	-1.658129108	0.222476486	-7.453053312	9.12E-14	1.06E-12
KLMA_70224	SPT21	protein SPT21	263.023691	-1.059003272	0.208708323	-5.074082616	3.89E-07	1.84E-06
KLMA_70225	NEW1	[NU+] prion formation protein 1	2530.509267	-1.436821452	0.141013473	-10.18924946	2.21E-24	7.16E-23
KLMA_70235	NOP14	probable nucleolar complex protein 14	707.45551	-1.031132503	0.199493562	-5.168750772	2.36E-07	1.15E-06
KLMA_70265	TIF3	eukaryotic translation initiation factor 4B	1134.665915	-1.239831221	0.221292559	-5.602679222	2.11E-08	1.22E-07
KLMA_70271	MET16	phosphoadenosine phosphosulfate reductase	1515.467045	-1.3545099	0.252232349	-5.370087962	7.87E-08	4.16E-07
KLMA_70282	NOC4	nucleolar complex protein 4	844.4202511	-1.185759154	0.16629791	-7.130331061	1.00E-12	1.04E-11
KLMA_70290	RRP9	ribosomal RNA-processing protein 9	541.9616374	-1.09490812	0.193459884	-5.659613226	1.52E-08	8.93E-08
KLMA_70313	HEM14	protoporphyrinogen oxidase	593.7442584	-1.240942356	0.143509253	-8.647124371	5.28E-18	9.85E-17

Locus_tag	UniProt _gene	Product	baseMean	log_2FoldChange	lfcSE	stat	pvalue	padj
KLMA_70323		uncharacterized protein YOL036W	691.7469606	-1.030914922	0.142697488	-7.224478417	5.03E-13	5.40E-12
KLMA_70324		transcriptional activator of sulfur metabolism	513.970424	-1.043973004	0.188037746	-5.551933209	2.83E-08	1.61E-07
KLMA_70355		uncharacterized protein YBL029C-A	98.82020058	-1.009875706	0.272568832	-3.705030028	0.000211366	0.000587541
KLMA_70357		UPF0642 protein YBL028C	186.8878598	-1.143676388	0.191551729	-5.970587651	2.36E-09	1.58E-08
KLMA_70358	RPL19B	60S ribosomal protein L19	13982.16313	-1.038751897	0.22369752	-4.643555714	3.42E-06	1.39E-05
KLMA_70359	MIS1	c-1-tetrahydrofolate synthase	1031.851812	-1.48139576	0.148738451	-9.959736349	2.29E-23	6.57E-22
KLMA_70362	MAK21	ribosome biogenesis protein MAK21	902.7957044	-1.714055527	0.138910411	-12.33928775	5.56E-35	3.36E-33
KLMA_70371	DYS1	deoxyhypusine synthase	2722.953291	-1.300248704	0.226284634	-5.746076008	9.13E-09	5.58E-08
KLMA_70383	PTR2	peptide transporter PTR2	1709.39415	-1.666507139	0.272724318	-6.110592388	9.93E-10	7.08E-09
KLMA_70384	UGA4	GABA-specific permease	270.1909531	-2.01402386	0.263151793	-7.653468119	1.96E-14	2.44E-13
KLMA_70390	SRP40	suppressor protein SRP40	388.0140383	-1.821845364	0.18864566	-9.657499482	4.57E-22	1.14E-20
KLMA_70395	PUS4	tRNA pseudouridine synthase 4	243.652581	-1.35986326	0.181970259	-7.472997314	7.84E-14	9.20E-13
KLMA_70398	PCL1	PHO85 cyclin-1	1595.193433	-1.295032437	0.161539038	-8.016838856	1.09E-15	1.59E-14
KLMA_70408		zinc finger protein YPR022C	941.9306644	-1.536368889	0.16795165	-9.147685591	5.82E-20	1.27E-18
KLMA_70417	NOP9	pumilio domain-containing protein YJL010C	587.96101	-1.430665994	0.165696653	-8.634247984	5.91E-18	1.09E-16
KLMA_70419	LTV1	protein LTV1	339.6707018	-1.43615168	0.173725322	-8.266795344	1.38E-16	2.19E-15
KLMA_80006	UGA4	GABA-specific permease	161.138846	-1.037856143	0.210017367	-4.941763417	7.74E-07	3.51E-06
KLMA_80054	HIP1	histidine permease	1933.134107	-2.610815928	0.258543	-10.09818842	5.63E-24	1.72E-22
KLMA_80059	GAP3	glyceraldehyde-3-phosphate dehydrogenase 3	138843.9088	-2.331840323	0.316649401	-7.364107802	1.78E-13	2.03E-12
KLMA_80061	MET3	sulfate adenylyltransferase	3317.219457	-1.312126246	0.310330001	-4.228164351	2.36E-05	8.11E-05
KLMA_80063	MNN1	alpha-1,3-mannosyltransferase	596.1594134	-2.334800007	0.200668723	-11.63509674	2.73E-31	1.39E-29
KLMA_80064	MNN1	alpha-1,3-mannosyltransferase	1075.060207	-1.349491429	0.244505871	-5.519259824	3.40E-08	1.92E-07
KLMA_80081	RLP24	ribosome biogenesis protein RLP24	635.50128	-1.113803903	0.154622704	-7.203365818	5.87E-13	6.25E-12
KLMA_80090		uncharacterized protein YGL146C	467.4310244	-1.74275345	0.25615296	-6.803565546	1.02E-11	9.41E-11
KLMA_80091	RRP42	exosome complex component RRP42	578.2467804	-1.051281935	0.175537976	-5.988914512	2.11E-09	1.42E-08
KLMA_80112	PUB1	nuclear and cytoplasmic polyadenylated RNA-binding protein	1395.676942	-1.010988452	0.200510657	-5.04206842	4.61E-07	2.15E-06

Locus_tag	UniProt_gene	Product	baseMean	log_2FoldChange	lfcSE	stat	pvalue	padj
		PUB1						
KLMA_80126	UTP25	uncharacterized protein YIL091C	363.4660159	-1.044077679	0.184223464	-5.667452204	1.45E-08	8.58E-08
KLMA_80132	HDA1	histone deacetylase HDA1	759.5866174	-1.389904704	0.197192327	-7.048472555	1.81E-12	1.81E-11
KLMA_80170	NOP2	putative ribosomal RNA methyltransferase Nop2	1039.47978	-1.417909647	0.152719314	-9.284416038	1.63E-20	3.73E-19
KLMA_80171	TRM6	tRNA (adenine-N(1)-)-methyltransferase non-catalytic subunit TRM6	386.4151926	-1.02017771	0.190312651	-5.360535431	8.30E-08	4.37E-07
KLMA_80174	AQR1	probable transporter AQR1	2248.496291	-2.84732638	0.274459081	-10.37432017	3.25E-25	1.12E-23
KLMA_80175	QDR2	quinidine resistance protein 2	544.0663972	-1.237421581	0.210716577	-5.872445339	4.29E-09	2.79E-08
KLMA_80180		uncharacterized sulfatase PB10D8.02c	199.7677152	-1.046163433	0.276193718	-3.787788661	0.000151994	0.000436927
KLMA_80185	BGL2	glucan 1	6309.316048	-1.282940334	0.210319662	-6.099954333	1.06E-09	7.52E-09
KLMA_80186	NIP1	eukaryotic translation initiation factor 3 subunit C	3594.360517	-1.031404603	0.15870516	-6.498872528	8.09E-11	6.58E-10
KLMA_80217	ECM3	protein ECM3	931.5051079	-1.215600152	0.141386167	-8.597730441	8.13E-18	1.48E-16
KLMA_80224		nucleoporin NUP1	289.1798116	-1.004799843	0.244654761	-4.107011201	4.01E-05	0.000130679
KLMA_80236	MET4	transcriptional activator of sulfur metabolism MET4	269.3081775	-1.0170114	0.184707124	-5.506075669	3.67E-08	2.05E-07
KLMA_80241		jmjC domain-containing protein 4	1245.630376	-2.203965783	0.189479986	-11.63165477	2.85E-31	1.43E-29
KLMA_80242	GDS1	protein GDS1	1633.21274	-1.486329301	0.146631942	-10.1364633	3.81E-24	1.19E-22
KLMA_80243	CIR2	probable electron transfer flavoprotein-ubiquinone oxidoreductase	2210.66694	-1.198496774	0.205194108	-5.840795273	5.20E-09	3.30E-08
KLMA_80249		UPF0364 protein YMR027W	1706.248388	-1.142551435	0.162514295	-7.03046728	2.06E-12	2.04E-11
KLMA_80256		translationally-controlled tumor protein homolog	8876.546859	-1.090842128	0.192361113	-5.670803795	1.42E-08	8.43E-08
KLMA_80257	DEF1	uncharacterized protein YKL054C	2549.726423	-1.014921935	0.192856909	-5.262564559	1.42E-07	7.22E-07
KLMA_80272	PUF6	pumilio homology domain family member 6	1207.777655	-1.228052281	0.157647133	-7.789880189	6.71E-15	8.97E-14
KLMA_80284	VID24	vacuolar import and degradation protein 24	1267.386433	-2.336073336	0.20479501	-11.40688604	3.86E-30	1.85E-28
KLMA_80303		protein OPY2	1000.155967	-1.048726058	0.142195328	-7.37524978	1.64E-13	1.87E-12

Locus_tag	UniProt _gene	Product	baseMean	log$_2$FoldChange	lfcSE	stat	pvalue	padj
KLMA_80317	TPS1	trehalose-6-phosphate synthase	3741.522418	-2.057364518	0.122274923	-16.82572732	1.58E-63	3.09E-61
KLMA_80325	ROY1	uncharacterized protein YMR258C	909.1595721	-1.71413587	0.175609716	-9.761053696	1.65E-22	4.37E-21
KLMA_80338	PIN4	RNA-binding protein PIN4	1158.607375	-1.041707966	0.189843733	-5.487186475	4.08E-08	2.26E-07
KLMA_80341	DOT6	transcriptional regulatory protein DOT6	1669.285155	-1.004530395	0.128391707	-7.823950768	5.12E-15	6.93E-14
KLMA_80345	PTH2	peptidyl-tRNA hydrolase 2	547.9289814	-1.197349682	0.192759312	-6.211630824	5.24E-10	3.85E-09
KLMA_80348	MET6	5-methyltetrahydropteroyltriglutamate homocysteine methyltransferase	34841.92933	-1.509066135	0.292086419	-5.166505656	2.39E-07	1.16E-06
KLMA_80369	ILS1	isoleucyl-tRNA synthetase	3563.051555	-1.114724036	0.21800376	-5.113324822	3.17E-07	1.51E-06
KLMA_80380	KAP123	importin subunit beta-4	5121.453418	-1.062836044	0.204361068	-5.200775529	1.98E-07	9.81E-07
KLMA_80413	RPL8B	60S ribosomal protein L8-B	11122.54698	-1.181748106	0.234807504	-5.032837901	4.83E-07	2.25E-06

Up-regulated DEGs

Locus_tag	UniProt _gene	Product	baseMean	log$_2$FoldChange	lfcSE	stat	pvalue	padj
KLMA_10001	TY2B-GR2	RVT_2 super family	135.1219604	1.179543128	0.258634609	4.560654643	5.10E-06	2.00E-05
KLMA_10010	ARN2	siderophore iron transporter ARN2	587.9678966	1.111944794	0.330792751	3.361454539	0.000775331	0.001883747
KLMA_10012		uncharacterized protein C11D3.14c	145.674767	2.128284189	0.374716489	5.679718533	1.35E-08	8.04E-08
KLMA_10013		hypothetical protein	4.949355681	5.523381439	1.574300017	3.508468132	0.000450695	0.001149112
KLMA_10014	MATALPHA1	mating-type protein ALPHA1	116.0169163	6.191096258	1.626356452	3.806727763	0.000140818	0.000408405
KLMA_10015	HMLALPHA2	mating-type protein ALPHA2	134.8495506	5.388038136	1.243557331	4.332762151	1.47E-05	5.26E-05
KLMA_10016	HMLALPHA3	mating-type protein ALPHA3	415.2763966	6.173309106	1.117379849	5.52480798	3.30E-08	1.86E-07
KLMA_10018	CHA1	catabolic L-serine/threonine dehydratase	83.70306925	1.442253488	0.40139345	3.593116654	0.000326746	0.000865886
KLMA_10026		hypothetical protein	185.0524112	1.68621591	0.248756718	6.778574352	1.21E-11	1.11E-10

Locus_tag	UniProt _gene	Product	baseMean	log_2FoldChange	lfcSE	stat	pvalue	padj
KLMA_10029	KAR4	karyogamy protein KAR4	120.2627024	2.128584442	0.24838394	8.569734599	1.04E-17	1.85E-16
KLMA_10039		protein SNA2	997.3173102	3.867979941	0.342455669	11.29483402	1.39E-29	6.35E-28
KLMA_10044	SPS22	cell wall mannoprotein PST1	92.09984338	1.447410885	0.466614995	3.101938218	0.001922581	0.004287619
KLMA_10059	LSB5	LAS seventeen-binding protein 5	861.851194	1.115876078	0.156403044	7.134618687	9.71E-13	1.01E-11
KLMA_10060	MXR2	selR super family	149.5119675	1.504101865	0.237692647	6.327927626	2.48E-10	1.88E-09
KLMA_10080	VTA1	vacuolar protein sorting-associated protein VTA1	222.6843313	1.314448744	0.212865871	6.175009341	6.62E-10	4.82E-09
KLMA_10086	PRM1	plasma membrane fusion protein PRM1	36.33378322	2.182975613	0.528222078	4.132685292	3.59E-05	0.000118435
KLMA_10091	ATG26	sterol 3-beta-glucosyltransferase	1212.936963	1.811120583	0.248943263	7.275234374	3.46E-13	3.81E-12
KLMA_10110	IST1	vacuolar protein sorting-associated protein IST1	627.9422748	1.501591362	0.178786774	8.398783252	4.51E-17	7.57E-16
KLMA_10115	CWC23	pre-mRNA-splicing factor CWC23	171.7796805	1.029573538	0.216453868	4.756549505	1.97E-06	8.39E-06
KLMA_10120		uncharacterized ORAOV1 family protein YNL260C	123.7490443	1.354627863	0.322326744	4.202654254	2.64E-05	8.98E-05
KLMA_10151	ECM25	protein ECM25	400.2907156	1.06109618	0.188412382	5.631775206	1.78E-08	1.04E-07
KLMA_10162	VPS60	vacuolar protein-sorting-associated protein 60	303.9554338	1.072019023	0.226314329	4.736858813	2.17E-06	9.17E-06
KLMA_10170		SMC_prok_B	196.0533717	1.543934255	0.345281487	4.471523421	7.77E-06	2.93E-05
KLMA_10174		hypothetical protein	528.664432	1.847224552	0.270153881	6.837675415	8.05E-12	7.50E-11
KLMA_10186	YAT1	putative mitochondrial carnitine O-acetyltransferase	492.3689816	2.752429932	0.175682851	15.66703818	2.54E-55	3.55E-53
KLMA_10188	KIN3	serine/threonine-protein kinase KIN3	331.7400086	1.981242852	0.201610496	9.82708187	8.61E-23	2.34E-21
KLMA_10195	CDC7	cell division control protein 7	212.2628273	1.053476602	0.216226363	4.872100639	1.10E-06	4.88E-06
KLMA_10214	ECI1	3,2-trans-enoyl-CoA isomerase	109.2528317	1.678788977	0.265000039	6.335051813	2.37E-10	1.80E-09
KLMA_10228	YHC1	U1 small nuclear ribonucleoprotein C homolog	185.8747004	1.274923664	0.220177866	5.790426116	7.02E-09	4.40E-08
KLMA_10231	ECM38	gamma-glutamyltransferase	303.8355855	1.582756848	0.230479855	6.867224231	6.55E-12	6.16E-11
KLMA_10235		WW domain-containing protein YFL010C	647.2211903	1.329966871	0.18097695	7.348819124	2.00E-13	2.25E-12
KLMA_10239		hypothetical protein	49.66799514	5.971300472	0.898703751	6.644347999	3.05E-11	2.62E-10
KLMA_10246		hypothetical protein	100.8296517	1.316999695	0.337374208	3.903676282	9.47E-05	0.000285631

Locus_tag	UniProt _gene	Product	baseMean	log_2FoldChange	lfcSE	stat	pvalue	padj
KLMA_10251	ATG12	autophagy-related protein 12	38.80800482	1.448289815	0.418133401	3.463702764	0.000532795	0.001337189
KLMA_10257	HRD1	ERAD-associated E3 ubiquitin-protein ligase HRD1	872.4707991	1.290794688	0.202351877	6.378960792	1.78E-10	1.39E-09
KLMA_10258		OPA3-like protein	128.285885	1.701710664	0.235436446	7.227898198	4.91E-13	5.30E-12
KLMA_10260	PLB	lysophospholipase	5998.562713	1.380244887	0.18340058	7.525848007	5.24E-14	6.29E-13
KLMA_10268	MIC17	mitochondrial intermembrane space cysteine motif-containing protein MIC17	427.0223318	1.349774379	0.173444885	7.782151527	7.13E-15	9.49E-14
KLMA_10277		hypothetical protein	281.2374108	1.26914199	0.196677904	6.452895646	1.10E-10	8.77E-10
KLMA_10295	MCH2	probable transporter MCH2	786.9181775	2.978593796	0.277132731	10.74789609	6.06E-27	2.49E-25
KLMA_10297	URH1	uridine nucleosidase	656.917421	1.480318839	0.251461152	5.886868914	3.94E-09	2.57E-08
KLMA_10298	DIT2	cytochrome P450-DIT2	360.3743391	1.289716554	0.245006793	5.264003244	1.41E-07	7.18E-07
KLMA_10316	HEM4	uroporphyrinogen-III synthase	91.95607237	1.63730494	0.277027482	5.91026179	3.42E-09	2.25E-08
KLMA_10338	FMP43	UPF0041 protein FMP43	173.6415599	1.013212147	0.229238052	4.419912572	9.87E-06	3.65E-05
KLMA_10375	ATG27	autophagy-related protein 27	928.9080772	1.078773778	0.216291951	4.987581698	6.11E-07	2.80E-06
KLMA_10382	STE3	pheromone a factor receptor	297.2023461	2.03338532	0.276960867	7.341778422	2.11E-13	2.36E-12
KLMA_10393		lrgB super family	834.0380258	1.899629918	0.283629622	6.697572372	2.12E-11	1.87E-10
KLMA_10426	AIM32	hypothetical protein	76.03933196	1.616661449	0.275814685	5.861404547	4.59E-09	2.95E-08
KLMA_10430	PES4	RRM	285.8610061	3.032585753	0.311089532	9.748273221	1.88E-22	4.90E-21
KLMA_10432	YSD83	NADB_Rossmann super family	720.5700545	1.068118931	0.219332545	4.86986065	1.12E-06	4.93E-06
KLMA_10443		hypothetical protein	157.4447019	2.585267916	0.386869669	6.682529351	2.35E-11	2.06E-10
KLMA_10454	PMC1	calcium-transporting ATPase 2	4405.746308	1.992111481	0.234202121	8.505949797	1.80E-17	3.14E-16
KLMA_10457	PMA1	plasma membrane ATPase	70784.90236	1.842842517	0.150150534	12.27329979	1.26E-34	7.16E-33
KLMA_10469	SSP1	hypothetical protein	83.82636602	1.504676045	0.309397196	4.863250419	1.15E-06	5.09E-06
KLMA_10486		uncharacterized protein YGR266W	210.1218754	2.010652469	0.261540365	7.687732891	1.50E-14	1.89E-13
KLMA_10516	PGU1	polygalacturonase	205.0538828	1.400742471	0.400143773	3.500597952	0.000464216	0.001181464
KLMA_10518	INU1	inulinase	498.0170365	1.159534575	0.312901722	3.705746867	0.000210769	0.000586216
KLMA_10520		uncharacterized protein AN0679	144.5749667	2.660041101	0.327121135	8.131669933	4.23E-16	6.42E-15
KLMA_10522	ZTA1	probable quinone oxidoreductase	777.0712947	1.081415922	0.214734283	5.03606554	4.75E-07	2.21E-06

Locus_tag	UniProt _gene	Product	baseMean	log_2FoldChange	lfcSE	stat	pvalue	padj
KLMA_10523		hypothetical protein	116.3779047	1.342579443	0.287649986	4.667406593	3.05E-06	1.25E-05
KLMA_10525		atg31 super family	74.51048502	1.07057498	0.295694609	3.620542772	0.000293986	0.000788915
KLMA_10545	HGT1	high-affinity glucose transporter	776.7843966	3.158288303	0.356832457	8.850899751	8.68E-19	1.71E-17
KLMA_10551		N-terminal acetyltransferase C complex subunit MAK31	32.47682521	1.698254159	0.43555709	3.899039181	9.66E-05	0.000290977
KLMA_10552	MAK32	protein MAK32	330.3431807	1.084176792	0.181358648	5.978081584	2.26E-09	1.51E-08
KLMA_10560		glutathione transferase 3	1008.148504	1.877496112	0.175141274	10.71989523	8.21E-27	3.34E-25
KLMA_10567		hypothetical protein	257.0683753	1.787810243	0.25906729	6.900949338	5.17E-12	4.94E-11
KLMA_10570		SF3b10 super family	35.03736657	1.275003238	0.452027374	2.820632798	0.004792903	0.00988119
KLMA_10583	CTK3	CTD kinase subunit gamma	404.0603607	1.431641132	0.290393379	4.930006107	8.22E-07	3.70E-06
KLMA_10592	KYE1	enoate reductase 1	797.2244774	1.441860974	0.270696547	5.326484538	1.00E-07	5.22E-07
KLMA_10596		hemocyanin	26.14010686	3.365500226	0.798002867	4.217403679	2.47E-05	8.46E-05
KLMA_10598	RRT8	DUF540	41.23520499	2.197402915	0.430248217	5.107291156	3.27E-07	1.56E-06
KLMA_10603	IRS4	increased rDNA silencing protein 4	159.1381163	1.010216354	0.291763131	3.462453771	0.000535274	0.00134189
KLMA_10605		RING finger protein YKR017C	482.8750539	1.197825987	0.184344535	6.497756951	8.15E-11	6.61E-10
KLMA_10613		uncharacterized protein KLLA0D02464g	119.7056861	2.27231658	0.308530816	7.364958244	1.77E-13	2.02E-12
KLMA_10622	YKT6	synaptobrevin homolog YKT6	589.5436992	1.2435548	0.14807427	8.398182891	4.53E-17	7.58E-16
KLMA_10624	SPP382	pre-mRNA-splicing factor SPP382	230.5146956	1.68681779	0.292463769	5.767612841	8.04E-09	5.00E-08
KLMA_10625	ATG17	autophagy-related protein 17	220.6734963	2.208982693	0.270339019	8.17115748	3.05E-16	4.69E-15
KLMA_10635	DAL5	allantoate permease	390.941102	1.178567761	0.239984168	4.911022966	9.06E-07	4.05E-06
KLMA_10637		hypothetical protein	59.95557766	3.378472744	0.512588571	6.591002873	4.37E-11	3.69E-10
KLMA_10647		hypothetical protein	461.6861575	2.068812412	0.163987503	12.61567112	1.73E-36	1.13E-34
KLMA_10667	SMD1	small nuclear ribonucleoprotein Sm D1	208.3527117	1.531700683	0.198195418	7.728234592	1.09E-14	1.42E-13
KLMA_10674	ENT4	epsin-4	201.9343045	1.843066552	0.208226755	8.851247527	8.65E-19	1.71E-17
KLMA_10679	RAN1	putative copper-transporting ATPase 3	4137.270928	2.308352284	0.263712029	8.753306762	2.07E-18	3.95E-17
KLMA_10688	BIG1	protein BIG1	149.5932511	1.102308622	0.230628244	4.779590744	1.76E-06	7.55E-06
KLMA_10689	PEP12	syntaxin PEP12	373.8036757	1.491701995	0.241931223	6.165810182	7.01E-10	5.10E-09

Locus_tag	UniProt _gene	Product	baseMean	log_2FoldChange	lfcSE	stat	pvalue	padj
KLMA_10715	SOU2	sorbose reductase homolog SOU2	1924.10284	5.188854139	0.321982759	16.11531674	1.99E-58	3.24E-56
KLMA_10722	HUL4	probable E3 ubiquitin-protein ligase HUL4	246.0094938	1.502928629	0.423982412	3.544790031	0.000392926	0.001018713
KLMA_10725	RAD26	DNA repair and recombination protein RAD26	676.4161987	2.46493218	0.36120844	6.824126748	8.85E-12	8.18E-11
KLMA_10743	COS111	F-box protein COS111	1130.008392	1.073788052	0.296753333	3.618453219	0.000296369	0.000794002
KLMA_10749		uncharacterized protein YPL067C	358.451379	1.146700463	0.169925682	6.748246958	1.50E-11	1.35E-10
KLMA_10754		uncharacterized protein YPL071C	75.31638723	2.001911485	0.31910017	6.273614596	3.53E-10	2.61E-09
KLMA_10756	UBP16	hypothetical protein	597.0030858	1.402961032	0.222323437	6.310450444	2.78E-10	2.09E-09
KLMA_10757	YTA6	probable 26S protease subunit YTA6	234.6443378	2.106431212	0.266740931	7.896917819	2.86E-15	3.94E-14
KLMA_10774	GDT1	UPF0016 membrane protein YBR187W	1194.496652	1.752261765	0.191514689	9.149490191	5.72E-20	1.25E-18
KLMA_10775	PCH2	pachytene checkpoint protein 2	168.4662498	1.844415501	0.23436171	7.869952386	3.55E-15	4.87E-14
KLMA_10781	GLY1	low specificity L-threonine aldolase	241.8618811	1.604501933	0.21659914	7.407702217	1.29E-13	1.48E-12
KLMA_10782	YDC1	alkaline ceramidase YDC1	1933.886018	2.179408546	0.358579907	6.077888092	1.22E-09	8.50E-09
KLMA_10783	SOU1	sorbose reductase SOU1	1972.091823	3.954532465	0.267438334	14.78670765	1.78E-49	1.78E-47
KLMA_10796	PNG1	peptide-N(4)-(N-acetyl-beta-glucosaminyl)asparagine amidase	249.5895478	1.153575148	0.220300057	5.236381534	1.64E-07	8.21E-07
KLMA_10804	ECM31	3-methyl-2-oxobutanoate hydroxymethyltransferase	192.5479121	1.017617337	0.22276683	4.56808285	4.92E-06	1.94E-05
KLMA_10805		uncharacterized abhydrolase domain-containing protein YGR015C	435.4059795	4.273516875	0.347010356	12.31524304	7.50E-35	4.41E-33
KLMA_10806		hypothetical protein	28.69197955	1.851504958	0.453668379	4.081185829	4.48E-05	0.00014435
KLMA_10814		oxidored-like super family	238.2390819	1.168555631	0.227909947	5.127269112	2.94E-07	1.41E-06
KLMA_10818	DAL3	ureidoglycolate hydrolase	115.7907542	1.769524885	0.278750649	6.348056548	2.18E-10	1.67E-09
KLMA_10830	Nqo2	ribosyldihydronicotinamide dehydrogenase [quinone]	1432.562977	1.379877548	0.18597687	7.41961917	1.17E-13	1.35E-12
KLMA_10836	FCY2	purine-cytosine permease FCY2	80.82266089	4.372459182	0.561315867	7.789658972	6.72E-15	8.97E-14
KLMA_20003		conserved hypothetical membrane protein	19.30093819	2.179065617	0.540139657	4.034263343	5.48E-05	0.000172925
KLMA_20007	yxeK	putative monooxygenase yxeK	140.776473	1.045818822	0.280242584	3.731834059	0.000190091	0.000534486
KLMA_20008	FRE4	ferric reductase transmembrane	510.0486481	1.377534857	0.39435097	3.493169697	0.000477323	0.001211037

Locus_tag	UniProt _gene	Product	baseMean	log_2FoldChange	lfcSE	stat	pvalue	padj
		component 4						
KLMA_20009	ADY2	hypothetical protein	973.5749856	5.055265245	0.380980512	13.26909143	3.50E-40	2.90E-38
KLMA_20010		protein crtK	62.45035844	1.713544307	0.32835637	5.218550531	1.80E-07	8.98E-07
KLMA_20012	yciC	ras_like_GTPase super family	521.8543153	2.613848432	0.72399871	3.610294321	0.00030585	0.000815377
KLMA_20014		hypothetical protein	269.2869929	1.37867165	0.190817214	7.225090538	5.01E-13	5.39E-12
KLMA_20015	YUH1	ubiquitin carboxyl-terminal hydrolase YUH1	226.2714781	1.549550879	0.224184938	6.911931268	4.78E-12	4.60E-11
KLMA_20018	AIM25	altered inheritance rate of mitochondria protein 25	401.3976183	1.088491573	0.193644121	5.621092789	1.90E-08	1.10E-07
KLMA_20020		protein PEA2	505.9184859	1.07080115	0.252548676	4.239979266	2.24E-05	7.73E-05
KLMA_20027		hypothetical protein	93.34323512	4.891834387	1.041833132	4.695410652	2.66E-06	1.10E-05
KLMA_20032	MAG1	DNA-3-methyladenine glycosylase	148.9368	1.382079055	0.292230242	4.729418301	2.25E-06	9.45E-06
KLMA_20037	RFT1	oligosaccharide translocation protein RFT1	764.2834556	1.137040961	0.162842311	6.982466394	2.90E-12	2.85E-11
KLMA_20041	FUS3	mitogen-activated protein kinase FUS3	126.7124933	4.311361955	0.436855033	9.869090731	5.67E-23	1.57E-21
KLMA_20050	MIOX5	inositol oxygenase 1	118.160789	1.082384394	0.237066183	4.56574777	4.98E-06	1.96E-05
KLMA_20057		uricase	52.79982582	1.188525291	0.350502452	3.390918617	0.000696588	0.001712197
KLMA_20064		hypothetical conserved protein	68.54658907	1.818088635	0.32126928	5.659080238	1.52E-08	8.94E-08
KLMA_20069	SAP30	transcriptional regulatory protein SAP30	368.2727	1.776556652	0.309776863	5.734955908	9.75E-09	5.93E-08
KLMA_20070		metallo-dependent_hydrolases	108.8756023	2.41877036	0.304845157	7.934422781	2.11E-15	2.97E-14
KLMA_20072		hypothetical conserved protein	366.5317699	1.374159213	0.273644455	5.021695804	5.12E-07	2.37E-06
KLMA_20076	NIPA2	magnesium transporter NIPA2	195.00354	1.770913051	0.21186976	8.358498413	6.35E-17	1.03E-15
KLMA_20101		sterol-sensing super family	1654.865426	1.332116775	0.306562856	4.345330002	1.39E-05	4.99E-05
KLMA_20105	CIT1	citrate synthase	12244.51674	1.089419556	0.157908457	6.8990577	5.23E-12	4.98E-11
KLMA_20111	RTT109	histone acetyltransferase RTT109	212.9501928	1.207433	0.20516639	5.885140361	3.98E-09	2.59E-08
KLMA_20117	ADR1	regulatory protein ADR1	971.8228571	3.750399573	0.222783414	16.83428538	1.37E-63	2.78E-61
KLMA_20130	ECM4	glutathione S-transferase omega-like 2	2419.741019	3.936429688	0.271365832	14.50598868	1.11E-47	1.04E-45
KLMA_20131		Golgi apparatus membrane protein	1853.089021	1.828624647	0.267415201	6.838147731	8.02E-12	7.49E-11

Locus_tag	UniProt _gene	Product	baseMean	log_2FoldChange	lfcSE	stat	pvalue	padj
		TVP15						
KLMA_20140	GSM1	glucose starvation modulator protein 1	204.8465582	1.65318214	0.29705993	5.565146873	2.62E-08	1.50E-07
KLMA_20164		hypothetical protein	120.7863109	1.475328268	0.286122395	5.156283794	2.52E-07	1.22E-06
KLMA_20174	DMC1	meiotic recombination protein DMC1	75.33177002	1.085756422	0.313309963	3.465438545	0.000529367	0.001329954
KLMA_20176		actin superfamily	94.10225983	1.075760407	0.244095988	4.40712039	1.05E-05	3.86E-05
KLMA_20193	PXA1	peroxisomal long-chain fatty acid import protein 2	181.7155576	3.331772079	0.319473883	10.42893414	1.83E-25	6.47E-24
KLMA_20194		UPF0613 protein PB24D3.06c	523.9225658	1.720381433	0.279536958	6.154397068	7.54E-10	5.44E-09
KLMA_20195	ppr1	acetyltransferases	241.7148361	1.579388938	0.180258368	8.761806466	1.92E-18	3.71E-17
KLMA_20200		SGT1 super family	112.9460357	1.092397368	0.325260254	3.358533222	0.000783573	0.001902073
KLMA_20203	SSP2	sporulation-specific protein 2	64.21992969	6.339799428	0.874986954	7.24559309	4.31E-13	4.70E-12
KLMA_20212	HST2	NAD-dependent deacetylase HST2	90.66735386	1.005913607	0.30051939	3.347250263	0.000816175	0.001974343
KLMA_20220	POT1	3-ketoacyl-CoA thiolase	153.0895362	2.814214995	0.32807027	8.578086013	9.65E-18	1.74E-16
KLMA_20221	THI72	thiamine transporter	218.7279271	1.641600696	0.228770882	7.175741443	7.19E-13	7.60E-12
KLMA_20223	DSD1	D-serine dehydratase	491.7746084	1.129170303	0.166516716	6.781122837	1.19E-11	1.09E-10
KLMA_20241	ERT1	uncharacterized transcriptional regulatory protein YBR239C	476.9056834	1.328156524	0.318142744	4.174718887	2.98E-05	0.000100149
KLMA_20248	CSM1	monopolin complex subunit CSM1	100.5858473	1.191211376	0.226744938	5.253530177	1.49E-07	7.56E-07
KLMA_20258	JEN1	putative sialic acid transporter	234.3247852	2.895917741	0.251954227	11.49382481	1.42E-30	6.99E-29
KLMA_20261		hypothetical protein	50.6852782	1.54724904	0.369654621	4.185661297	2.84E-05	9.60E-05
KLMA_20263	POX1	acyl-coenzyme A oxidase	362.2944928	2.145403376	0.213884326	10.03067131	1.12E-23	3.31E-22
KLMA_20269		mitochondrial outer membrane protein OM14	1462.791683	3.471927784	0.582639608	5.958962859	2.54E-09	1.69E-08
KLMA_20273	SLX1	structure-specific endonuclease subunit SLX1	306.5188488	3.154955458	0.256608459	12.29482253	9.66E-35	5.61E-33
KLMA_20292		uncharacterized protein YEL137C	160.2186623	1.638812129	0.250574846	6.540210065	6.14E-11	5.07E-10
KLMA_20296	GPD2	glyceraldehyde-3-phosphate dehydrogenase 2	65.64796201	4.149256117	0.477471807	8.690054695	3.62E-18	6.86E-17
KLMA_20298	IME4	N6-adenosine-methyltransferase IME4	166.972017	2.786545368	0.43196793	6.45081538	1.11E-10	8.88E-10

Locus_tag	UniProt _gene	Product	baseMean	log_2FoldChange	lfcSE	stat	pvalue	padj
KLMA_20301	RIM15	serine/threonine-protein kinase RIM15	1634.850192	1.327082225	0.222815957	5.955956839	2.59E-09	1.72E-08
KLMA_20303		basic-leucine zipper (bZIP) transcription factor	22658.11645	1.082331172	0.220443586	4.909787547	9.12E-07	4.07E-06
KLMA_20304	AGX1	alanine--glyoxylate aminotransferase 1	912.5706414	3.210109506	0.212513887	15.10541056	1.49E-51	1.62E-49
KLMA_20317	SPS4	sporulation-specific protein 4	95.92074163	1.489349374	0.365766948	4.071853355	4.66E-05	0.000149666
KLMA_20324	YHM2	mitochondrial DNA replication protein YHM2	1661.947786	1.070450563	0.214468119	4.991187355	6.00E-07	2.75E-06
KLMA_20327	RRG7	uncharacterized protein YOR305W	70.83302543	1.027191166	0.294609263	3.486622092	0.000489162	0.0012385
KLMA_20329	CUS1	protein CUS1	238.4940638	1.218919974	0.188452918	6.468034503	9.93E-11	7.98E-10
KLMA_20334		hypothetical protein	37.78137545	1.635866505	0.436171249	3.750514294	0.000176472	0.000500517
KLMA_20345		protein FUN14	850.9703904	2.757756831	0.275607051	10.00611856	1.43E-23	4.19E-22
KLMA_20368		uncharacterized membrane protein YAL018C	6.305924971	3.922408551	1.291702348	3.036619509	0.002392472	0.005246895
KLMA_20380		hypothetical protein	103.7894433	3.825251332	0.935482225	4.089068963	4.33E-05	0.000139808
KLMA_20381	OSW5	hypothetical protein	16.74067895	5.400034187	1.160385291	4.653656184	3.26E-06	1.33E-05
KLMA_20383	IMP1	mitochondrial inner membrane protease subunit 1	54.8515523	1.089772379	0.298158982	3.655004358	0.000257178	0.000697825
KLMA_20384		hypothetical protein	13.7410609	4.056579205	0.988679439	4.103027779	4.08E-05	0.000132331
KLMA_20389	RAM1	protein farnesyltransferase subunit beta	184.2868112	1.199898397	0.208150868	5.764561102	8.19E-09	5.07E-08
KLMA_20426	RAD55	p-loop NTPase super family	453.7824541	1.316187607	0.243328708	5.409092989	6.33E-08	3.41E-07
KLMA_20427		hypothetical protein	17930.03697	2.195052392	0.717247062	3.060385339	0.002210524	0.004869733
KLMA_20428	MUC1	flocculation protein FLO11	448.5524221	1.166211339	0.201340917	5.792222256	6.95E-09	4.35E-08
KLMA_20431		uncharacterized protein YER152C	328.0191474	1.308065739	0.236349969	5.534444292	3.12E-08	1.76E-07
KLMA_20435		KH domain-containing protein YLL032C	586.2676888	1.244845826	0.146201518	8.514588953	1.67E-17	2.93E-16
KLMA_20442	UBI4	ubiquitin	7738.090765	1.355329856	0.233802235	5.796907188	6.75E-09	4.24E-08
KLMA_20457	POM33	UPF0121 membrane protein YLL023C	1400.186072	1.163419105	0.190383188	6.110934044	9.90E-10	7.07E-09
KLMA_20462	uapC	purine permease	681.5739341	2.205409339	0.179664619	12.27514552	1.23E-34	7.08E-33

Locus_tag	UniProt_gene	Product	baseMean	log_2FoldChange	lfcSE	stat	pvalue	padj
KLMA_20473	FAB1	1-phosphatidylinositol-3-phosphate 5-kinase FAB1	1906.983516	1.526975133	0.155420718	9.824784962	8.81E-23	2.38E-21
KLMA_20499	AOS1	DNA damage tolerance protein RHC31	262.1606128	1.117087136	0.177401517	6.296942394	3.04E-10	2.27E-09
KLMA_20504		NAP1-binding protein	173.0073955	1.079716344	0.223213064	4.837155687	1.32E-06	5.75E-06
KLMA_20509	SYF2	pre-mRNA-splicing factor SYF2	112.5429639	1.049783567	0.238430459	4.402892027	1.07E-05	3.93E-05
KLMA_20523		hypothetical protein	95.99023749	1.51795182	0.286980966	5.289381522	1.23E-07	6.29E-07
KLMA_20536		yjgF_YER057c_UK114_family	387.1525869	1.401620152	0.219854043	6.375230268	1.83E-10	1.42E-09
KLMA_20542	PIN3	[PSI+] inducibility protein 3	957.744179	2.164736897	0.224999424	9.621077503	6.51E-22	1.62E-20
KLMA_20546	VPS62	vacuolar protein sorting-associated protein 62	830.8364889	1.956692314	0.180381051	10.84754916	2.05E-27	8.62E-26
KLMA_20547		protein BTN2	197.1536396	1.513752723	0.246707136	6.135828677	8.47E-10	6.09E-09
KLMA_20581	MUS81	crossover junction endonuclease MUS81	201.0318506	1.141245875	0.252626187	4.517528014	6.26E-06	2.42E-05
KLMA_20586	ATO2	hypothetical protein	3811.784197	1.079339657	0.346425782	3.115644711	0.001835433	0.00411393
KLMA_20597	ARO10	transaminated amino acid decarboxylase	480.4351197	1.29959802	0.221894883	5.856818348	4.72E-09	3.02E-08
KLMA_20604	IAH1	isoamyl acetate-hydrolyzing esterase	274.305299	4.479833965	0.350547827	12.77952284	2.13E-37	1.49E-35
KLMA_20607		YTH super family	188.6633963	5.299209629	0.411196102	12.88730512	5.31E-38	3.81E-36
KLMA_20609	FRQ1	calcium-binding protein NCS-1	534.0676391	1.049123213	0.196517785	5.338566253	9.37E-08	4.90E-07
KLMA_20612	CTS2	sporulation-specific chitinase 2	246.3059911	2.303690181	0.803697092	2.866366201	0.004152136	0.008666254
KLMA_20621	SNG1	nitrosoguanidine resistance protein SNG1	489.3150796	1.277859768	0.18447985	6.926825699	4.30E-12	4.16E-11
KLMA_20634	PEX11	peroxisomal membrane protein PMP27	188.0454326	1.357801523	0.298429424	4.549824558	5.37E-06	2.10E-05
KLMA_20654	SNU23	U4/U6.U5 small nuclear ribonucleoprotein component	95.64155853	1.44225754	0.279411555	5.161767702	2.45E-07	1.19E-06
KLMA_20658		uncharacterized membrane protein YMR155W	626.8152388	1.967314099	0.192918925	10.19762111	2.03E-24	6.62E-23
KLMA_20659	MSC1	meiotic sister chromatid recombination protein 1	2177.629594	3.888270744	0.662325109	5.870637684	4.34E-09	2.81E-08
KLMA_20660		UPF0549 protein C1D4.09c	224.0987323	1.628075524	0.313780604	5.188579232	2.12E-07	1.04E-06
KLMA_20673	ALD2	aldehyde dehydrogenase [NAD(P)+]	506.3715929	1.304185113	0.255589301	5.102659254	3.35E-07	1.60E-06

**Locus_tag**	**UniProt _gene**	**Product**	**baseMean**	**log$_2$FoldChange**	**lfcSE**	**stat**	**pvalue**	**padj**
		1						
KLMA_20675	MLH1	DNA mismatch repair protein MLH1	172.9476537	1.098051164	0.196329624	5.592895987	2.23E-08	1.28E-07
KLMA_20685		uncharacterized protein YJR003C	307.5585631	1.13674584	0.209429537	5.427820042	5.70E-08	3.10E-07
KLMA_20689	FYV8	protein FYV8	328.621875	1.398007356	0.18250535	7.660089722	1.86E-14	2.33E-13
KLMA_20709	ATG14	autophagy-related protein 14	211.146902	2.870562391	0.445993505	6.436332271	1.22E-10	9.70E-10
KLMA_20730		hypothetical protein	147.6403959	1.914567815	0.332597541	5.756410019	8.59E-09	5.31E-08
KLMA_20734	GDH2	NAD-specific glutamate dehydrogenase	2593.153872	2.402657443	0.169407389	14.18271933	1.17E-45	1.02E-43
KLMA_20757		uncharacterized protein YNL165W	709.9704412	1.312705284	0.218164413	6.017045884	1.78E-09	1.20E-08
KLMA_20766	MEP2	ammonium transporter MEP2	389.3130207	2.735485493	0.209486073	13.05807803	5.72E-39	4.29E-37
KLMA_20771	HSP12	12 kDa heat shock protein	1483.443403	6.269192017	1.241956183	5.047836713	4.47E-07	2.09E-06
KLMA_20780		chaperone_DMP super family	50.98069963	1.14883461	0.314633906	3.651337599	0.000260878	0.000706671
KLMA_20781	YUC8	trkA	489.3694883	4.092210698	0.305733578	13.38489125	7.41E-41	6.24E-39
KLMA_20791	ISU1	iron sulfur cluster assembly protein 1	850.7194042	1.09772464	0.169888415	6.46144493	1.04E-10	8.32E-10
KLMA_20793		MTP18 super family	869.8342261	1.484777154	0.171244482	8.670510919	4.30E-18	8.10E-17
KLMA_20795	ODC2	mitochondrial 2-oxodicarboxylate carrier 2	3416.066541	1.020746788	0.327171072	3.119917606	0.001809016	0.004064046
KLMA_20797	RDS2	regulator of drug sensitivity 2	347.8440585	1.012360121	0.193617346	5.228664393	1.71E-07	8.54E-07
KLMA_20806	STE4	guanine nucleotide-binding protein subunit beta	620.2074151	1.446588743	0.201240699	7.188350819	6.56E-13	6.95E-12
KLMA_20813	PTP2	tyrosine-protein phosphatase 2	810.851954	1.642629411	0.315245708	5.210632119	1.88E-07	9.34E-07
KLMA_20815	RNY1	ribonuclease T2-like	323.2263949	2.552075718	0.287043914	8.890889475	6.06E-19	1.21E-17
KLMA_20816	RNY1	ribonuclease T2-like	356.6415712	2.645372773	0.306389243	8.634026275	5.92E-18	1.09E-16
KLMA_20819		hypothetical protein	229.6539206	5.169572214	0.396161623	13.04914942	6.43E-39	4.76E-37
KLMA_20823		uncharacterized transporter YBR287W	2292.590749	1.431853914	0.320081325	4.473406612	7.70E-06	2.91E-05
KLMA_20825	KHA1	K(+)/H(+) antiporter 1	1792.297338	1.06021564	0.178103661	5.952800936	2.64E-09	1.75E-08
KLMA_20826	FEN2	pantothenate transporter FEN2	253.6651656	1.909576131	0.221896431	8.605709091	7.58E-18	1.38E-16
KLMA_20834	amdS	acetamidase	54.44747654	2.326278401	0.513181129	4.533055231	5.81E-06	2.26E-05
KLMA_20837		FMN_red super family	223.0102796	1.466085771	0.387789949	3.780618283	0.000156439	0.000446813

Locus_tag	UniProt _gene	Product	baseMean	log_2FoldChange	lfcSE	stat	pvalue	padj
KLMA_30011		beta-glucosidase	4366.573528	1.429097352	0.209343352	6.826571463	8.70E-12	8.06E-11
KLMA_30013	CYB2	cytochrome b2	83.88934111	1.566667939	0.353981382	4.42584842	9.61E-06	3.57E-05
KLMA_30014		conserved hypothetical transmembrane protein	169.9859108	1.394524632	0.309363451	4.507722646	6.55E-06	2.52E-05
KLMA_30015	bioA	uncharacterized aminotransferase C1771.03c	279.3998338	2.490550055	0.254524114	9.785124154	1.30E-22	3.48E-21
KLMA_30016	YPR1	putative reductase 1	931.5779752	2.880781785	0.186857999	15.41695727	1.26E-53	1.62E-51
KLMA_30017	PUT4	probable proline-specific permease put4	536.1977841	3.317044136	0.36083073	9.192798352	3.83E-20	8.50E-19
KLMA_30018	ARI1	putative uncharacterized oxidoreductase YGL157W	167.3680953	1.370515385	0.367280719	3.731520098	0.000190328	0.000534845
KLMA_30019	fmo1	thiol-specific monooxygenase	292.8724967	2.094024224	0.331073037	6.324961527	2.53E-10	1.92E-09
KLMA_30037	mug157	meiotically up-regulated gene 157 protein	527.1059178	1.809902359	0.23464809	7.713262694	1.23E-14	1.58E-13
KLMA_30056		ubiquitin-binding protein CUE2	30.53808261	1.132426481	0.375153179	3.018570931	0.002539699	0.005537452
KLMA_30079		centromere DNA-binding protein complex CBF3 subunit	145.6581766	1.349292022	0.314908225	4.284715082	1.83E-05	6.43E-05
KLMA_30082	FUS2	nuclear fusion protein FUS2	15.20699002	1.937596595	0.583085428	3.323006378	0.000890529	0.00213832
KLMA_30086	mug14	meiotically up-regulated gene 14 protein	285.680689	2.159344378	0.258418748	8.355989632	6.49E-17	1.05E-15
KLMA_30087	RNH1	ribonuclease H	34.44237925	1.756384919	0.473219896	3.711561861	0.000205984	0.000574217
KLMA_30095		signal recognition particle subunit SRP21	314.6930668	1.502261583	0.19822891	7.57841823	3.50E-14	4.28E-13
KLMA_30100	YKU80	ATP-dependent DNA helicase II subunit 2	194.0653444	1.47926363	0.261751859	5.651396848	1.59E-08	9.32E-08
KLMA_30101	SPG4	uncharacterized protein YMR107W	635.3724629	5.596596728	0.27721779	20.18844722	1.24E-90	7.55E-88
KLMA_30112		uncharacterized protein YMR114C	246.6882976	2.123774099	0.299800446	7.083959114	1.40E-12	1.42E-11
KLMA_30124		succinate dehydrogenase [ubiquinone] cytochrome b subunit	4043.444797	1.018807738	0.177078683	5.753418322	8.75E-09	5.39E-08
KLMA_30129		hypothetical protein	831.6314501	2.839148636	0.331218008	8.571842615	1.02E-17	1.83E-16
KLMA_30133	RRG1	uncharacterized protein YDR065W	268.8951464	1.307507759	0.264838508	4.937000178	7.93E-07	3.58E-06
KLMA_30134	RTR1	RNA polymerase II-associated protein 2 homolog	182.3174693	1.802671723	0.247675275	7.278367717	3.38E-13	3.73E-12

Locus_tag	UniProt _gene	Product	baseMean	log$_2$FoldChange	lfcSE	stat	pvalue	padj
KLMA_30137	mlo2	protein mlo2	639.3390379	2.124470387	0.228906884	9.280937088	1.68E-20	3.83E-19
KLMA_30151	SSP120	protein SSP120	281.6091493	1.336332366	0.209813404	6.369146776	1.90E-10	1.47E-09
KLMA_30152	SPO1	putative meiotic phospholipase SPO1	61.83229519	2.80575277	0.41311635	6.791676901	1.11E-11	1.02E-10
KLMA_30161	TOK1	outward-rectifier potassium channel TOK1	376.9623613	1.648552304	0.246697572	6.682482889	2.35E-11	2.06E-10
KLMA_30163	SRS2	ATP-dependent DNA helicase SRS2	257.8171535	1.003477998	0.211526039	4.743992766	2.10E-06	8.88E-06
KLMA_30166	SIP4	protein SIP4	703.8982253	2.441905476	0.336352615	7.259956868	3.87E-13	4.25E-12
KLMA_30183	RTG1	retrograde regulation protein 1	50.6167148	1.15260692	0.370976478	3.106954185	0.001890257	0.004221314
KLMA_30191	NAM8	protein NAM8	1063.204489	1.003817872	0.329571085	3.045831133	0.002320381	0.005100244
KLMA_30192		protein PET130	197.4121661	1.63836736	0.197677835	8.288068096	1.15E-16	1.86E-15
KLMA_30200	OKP1	central kinetochore subunit OKP1	207.8774319	1.019181795	0.200401786	5.085692177	3.66E-07	1.74E-06
KLMA_30231	MMM1	maintenance of mitochondrial morphology protein 1	158.0517389	1.033397238	0.225435604	4.584001902	4.56E-06	1.81E-05
KLMA_30242	CUE5	ubiquitin-binding protein CUE5	653.4153458	1.057930296	0.231338959	4.573074509	4.81E-06	1.90E-05
KLMA_30243	GLO4	hydroxyacylglutathione hydrolase	969.1779845	2.471063738	0.222588608	11.10148341	1.23E-28	5.48E-27
KLMA_30244		WD40 super family protein	360.4243561	2.089180221	0.281316012	7.426453284	1.12E-13	1.29E-12
KLMA_30245		protein midA homolog	184.8125607	1.965205734	0.239154287	8.217313427	2.08E-16	3.25E-15
KLMA_30258	ASE1	anaphase spindle elongation protein	393.0480176	1.15982633	0.182981521	6.338488838	2.32E-10	1.77E-09
KLMA_30260		putative lipase YOR059C	553.2972238	1.068072956	0.155287916	6.87801721	6.07E-12	5.74E-11
KLMA_30272		DUF676 super family[cl10636]	279.9073774	1.295487051	0.208915716	6.201003338	5.61E-10	4.10E-09
KLMA_30282		NAD(+) kinase	449.294741	1.149948719	0.168419273	6.827892658	8.62E-12	8.00E-11
KLMA_30312	ACO2	aconitate hydratase	19972.30461	1.149186381	0.265759499	4.324159196	1.53E-05	5.45E-05
KLMA_30316	UBC12	NEDD8-conjugating enzyme UBC12	49.93655073	1.246106908	0.334469455	3.725622438	0.000194834	0.000546251
KLMA_30321	ATG1	serine/threonine-protein kinase ATG1	1303.272335	1.071755996	0.173963958	6.16079335	7.24E-10	5.24E-09
KLMA_30322	MND1	meiotic nuclear division protein 1	13.17406897	4.426757143	0.982687388	4.504746062	6.65E-06	2.55E-05
KLMA_30323		zinc finger protein YER130C	2118.329391	2.115381103	0.308660091	6.853432504	7.21E-12	6.76E-11
KLMA_30331		hypothetical protein	79.61131714	2.275559764	0.370272163	6.145640938	7.96E-10	5.74E-09
KLMA_30332		sporulation protein 23	839.546106	3.322236212	0.204709713	16.22901111	3.14E-59	5.30E-57
KLMA_30336	VTI1	t-SNARE VTI1	786.5702055	1.024489762	0.15295909	6.697802393	2.12E-11	1.87E-10

Locus_tag	UniProt _gene	Product	baseMean	log_2FoldChange	lfcSE	stat	pvalue	padj
KLMA_30343		central kinetochore subunit MCM21	357.1900685	1.866346689	0.205472265	9.083204921	1.05E-19	2.26E-18
KLMA_30344		protein HIM1	1362.741455	3.438406864	0.232776498	14.77128013	2.24E-49	2.19E-47
KLMA_30354	LEE1	YTH1[COG5084]	900.8554218	2.084734728	0.194776293	10.70322621	9.83E-27	3.94E-25
KLMA_30363	PDR12	ATP-dependent permease PDR12	1941.947745	2.067693351	0.196899053	10.50128641	8.52E-26	3.08E-24
KLMA_30364	EXO5	defects in morphology protein 1	176.8825277	1.378469987	0.254198622	5.422806686	5.87E-08	3.18E-07
KLMA_30365		putative pyridoxal reductase	959.6556111	2.700993618	0.179150085	15.07670853	2.30E-51	2.45E-49
KLMA_30369		hypothetical protein	2243.343337	1.087041042	0.235353364	4.618761429	3.86E-06	1.55E-05
KLMA_30371	CMK2	calcium/calmodulin-dependent protein kinase II	920.5136217	1.381843962	0.235863805	5.858652051	4.67E-09	2.99E-08
KLMA_30395		ZZ_NBR1_like protein	606.2873295	1.074766149	0.236148248	4.551234901	5.33E-06	2.08E-05
KLMA_30396	PHM7	phosphate metabolism protein 7	1874.616193	3.463461277	0.325384845	10.64419972	1.86E-26	7.31E-25
KLMA_30399		conserved hypothetical membrane protein	26.03912406	2.171532311	0.624074307	3.479605371	0.000502153	0.001266795
KLMA_30405	TAD2	tRNA-specific adenosine deaminase subunit TAD2	79.85094363	1.317839346	0.282387676	4.666773579	3.06E-06	1.25E-05
KLMA_30406	SNX4	sorting nexin-4	366.371942	1.321689994	0.239991791	5.507230021	3.65E-08	2.05E-07
KLMA_30410	ZIP1	synaptonemal complex protein ZIP1	222.5970575	1.634635673	0.2292642	7.129921183	1.00E-12	1.04E-11
KLMA_30411	YIM1	uncharacterized protein YMR152W	2843.871327	1.197699314	0.126037771	9.502701457	2.05E-21	4.87E-20
KLMA_30412	DPP1	diacylglycerol pyrophosphate phosphatase 1	1858.495359	1.332091607	0.239361009	5.565198835	2.62E-08	1.50E-07
KLMA_30426	DPL1	sphingosine-1-phosphate lyase	1957.856656	2.324473844	0.255325832	9.10395094	8.71E-20	1.88E-18
KLMA_30428		uncharacterized protein YHR035W	432.3789557	1.446246576	0.177190272	8.162110489	3.29E-16	5.02E-15
KLMA_30434	PIH1	protein interacting with Hsp90 1	186.0959243	1.118812726	0.192427925	5.814191074	6.09E-09	3.85E-08
KLMA_30444		vacuolar membrane protein YPL162C	240.5935458	1.388137003	0.221870683	6.256513852	3.94E-10	2.90E-09
KLMA_30446	MLH3	DNA mismatch repair protein MLH3	317.0666202	1.215233056	0.263562318	4.610799703	4.01E-06	1.61E-05
KLMA_30452	REV3	DNA polymerase zeta catalytic subunit	246.3242622	1.244289164	0.261512053	4.758056664	1.95E-06	8.33E-06
KLMA_30462	NIP100	protein NIP100	253.1540416	1.058374242	0.195532663	5.412774663	6.21E-08	3.35E-07
KLMA_30482		glyoxalase super family protein	1614.900021	2.164268537	0.146554061	14.76771458	2.37E-49	2.27E-47
KLMA_30518		uncharacterized protein YDR239C	274.7289725	1.402745645	0.265945646	5.274557664	1.33E-07	6.79E-07

Locus_tag	UniProt _gene	Product	baseMean	log_2FoldChange	lfcSE	stat	pvalue	padj
KLMA_30524		putative cation exchanger YDL206W	512.7289997	1.455403676	0.231589764	6.284404159	3.29E-10	2.44E-09
KLMA_30533		4,5-DOPA dioxygenase extradiol-like protein	377.2173351	1.804269972	0.213185297	8.463388414	2.60E-17	4.45E-16
KLMA_30538		hypothetical protein	205.3992384	2.050587867	0.362509247	5.656649815	1.54E-08	9.06E-08
KLMA_30550	PIB1	E3 ubiquitin-protein ligase PIB1	255.1185711	1.314251402	0.212606573	6.181612286	6.35E-10	4.63E-09
KLMA_30555		conserved hypothetical transmembrane protein	484.7814036	5.067269813	0.931051577	5.442523202	5.25E-08	2.87E-07
KLMA_30556	GAD1	glutamate decarboxylase	3467.503444	1.175980069	0.16629716	7.071558358	1.53E-12	1.55E-11
KLMA_30563	PRR1	probable serine/threonine-protein kinase YKL116C	652.3221481	2.021677923	0.250764124	8.062070003	7.50E-16	1.12E-14
KLMA_30564	APN1	DNA-(apurinic or apyrimidinic site) lyase 1	188.4454669	1.103067627	0.220305956	5.006980498	5.53E-07	2.55E-06
KLMA_30572	IME2	meiosis induction protein kinase IME2/SME1	140.7087514	1.206086633	0.282422662	4.270502313	1.95E-05	6.83E-05
KLMA_30573		conserved hypothetical transmembrane protein	2857.23106	2.759254336	0.265625961	10.38774344	2.82E-25	9.84E-24
KLMA_30577	ARA1	D-arabinose dehydrogenase [NAD(P)+] heavy chain	323.1826635	2.14100021	0.206053076	10.39052777	2.74E-25	9.62E-24
KLMA_30597	OAF1	peroxisome proliferation transcriptional regulator	528.1383281	1.092309874	0.164946585	6.622203622	3.54E-11	3.01E-10
KLMA_30598	OAF1	oleate-activated transcription factor 1	257.252577	1.38600887	0.208667443	6.642190308	3.09E-11	2.65E-10
KLMA_30601		probable metabolite transport protein C1271.09	166.6553464	1.143196387	0.284610554	4.016704124	5.90E-05	0.000184534
KLMA_30602	ACS1	acetyl-coenzyme A synthetase 1	2646.297416	4.58474723	0.389158693	11.78117645	4.88E-32	2.56E-30
KLMA_30604		probable 2-nitropropane dioxygenase	332.504821	1.793399591	0.213396797	8.404060472	4.31E-17	7.26E-16
KLMA_30607	STL1	sugar transporter STL1	1427.785343	2.632552507	0.269993543	9.750427658	1.84E-22	4.82E-21
KLMA_30619	REC8	meiotic recombination protein REC8	135.8765806	1.082080091	0.238550109	4.53607042	5.73E-06	2.23E-05
KLMA_30623	mug70	meiotically up-regulated gene 70 protein	308.9018338	1.012850683	0.249370352	4.061632332	4.87E-05	0.000155862
KLMA_30624		conserved hypothetical transmembrane protein	87.77147143	1.738614013	0.371861046	4.675440006	2.93E-06	1.20E-05
KLMA_30629	FMP48	calcium/calmodulin-dependent protein kinase type 1D	4358.048289	1.679145066	0.248120728	6.767451795	1.31E-11	1.19E-10
KLMA_30633	RME1	conserved hypothetical membrane	672.5736643	1.82987836	0.218880998	8.360151753	6.26E-17	1.02E-15

Locus_tag	UniProt _gene	Product	baseMean	log_2FoldChange	lfcSE	stat	pvalue	padj
		protein						
KLMA_30642		conserved hypothetical transmembrane protein	189.9347656	1.180887384	0.329318579	3.585851086	0.000335981	0.000887949
KLMA_30644	PNS1	protein PNS1	1163.083967	2.204111764	0.232771177	9.469006389	2.83E-21	6.67E-20
KLMA_30654	CAT2	carnitine O-acetyltransferase	429.0585324	2.651457374	0.35295648	7.512136833	5.82E-14	6.93E-13
KLMA_30672		probable metabolite transport protein C1271.09	1311.023465	3.820914106	0.288294348	13.25351721	4.31E-40	3.50E-38
KLMA_30673		vacuolar morphogenesis protein 7	317.7691255	1.344458339	0.179553908	7.487769855	7.01E-14	8.27E-13
KLMA_30679	SDT1	protein SSM1	457.0114827	2.615785959	0.270425752	9.672843413	3.93E-22	9.95E-21
KLMA_30710	ATG32	protein ECM37	654.0120583	1.215597329	0.198280845	6.130684622	8.75E-10	6.27E-09
KLMA_30728	LAC12	lactose permease	406.3984377	2.43779149	0.372284034	6.548203157	5.82E-11	4.83E-10
KLMA_40003		probable acid phosphatase	599.7285525	8.262336911	0.537324639	15.37680632	2.34E-53	2.86E-51
KLMA_40004	THI13	pyrimidine precursor biosynthesis enzyme THI13	20.86457075	3.442345456	0.630349347	5.461012174	4.73E-08	2.60E-07
KLMA_40005		UPF0317 protein C5H10.01	59.90718468	1.537630517	0.400335175	3.840857894	0.000122605	0.000361378
KLMA_40006		uncharacterized transporter C1683.12	207.1629797	1.458628205	0.187117392	7.795257223	6.43E-15	8.62E-14
KLMA_40007	OXP1	uncharacterized protein YKL215C	215.789259	1.708172226	0.210149532	8.128365593	4.35E-16	6.58E-15
KLMA_40010		uncharacterized protein YMR315W	1535.021268	1.292530154	0.203937318	6.337879536	2.33E-10	1.77E-09
KLMA_40011	ESF2	pre-rRNA-processing protein ESF2	359.802767	1.374868012	0.209756543	6.554589395	5.58E-11	4.64E-10
KLMA_40016	LYS9	saccharopine dehydrogenase [NADP+	5841.631212	1.082113109	0.273248299	3.960182422	7.49E-05	0.000230773
KLMA_40031	EST2	telomerase reverse transcriptase	422.1515767	1.072248537	0.202536877	5.294090406	1.20E-07	6.14E-07
KLMA_40040	TFG2	transcription initiation factor IIF subunit beta	814.3767172	1.018240331	0.201835607	5.044899384	4.54E-07	2.12E-06
KLMA_40041		pre-mRNA-splicing factor 18	64.21661998	1.220433615	0.304970974	4.001802524	6.29E-05	0.000196052
KLMA_40042		uncharacterized membrane protein YLR326W	134.2907549	1.387221318	0.277412249	5.000577026	5.72E-07	2.63E-06
KLMA_40049	MSB2	hansenula MRAKII killer toxin-resistant protein	2803.634644	1.49956631	0.131628308	11.39243021	4.56E-30	2.14E-28
KLMA_40050		uncharacterized abhydrolase domain-containing protein YGR015C	566.9396959	2.029491367	0.188166571	10.78561063	4.03E-27	1.68E-25
KLMA_40052		meiotic recombination protein	196.4816087	1.238175687	0.211128893	5.86454877	4.50E-09	2.91E-08

Locus_tag	UniProt _gene	Product	baseMean	log_2FoldChange	lfcSE	stat	pvalue	padj
		REC102						
KLMA_40055	MDH3	malate dehydrogenase	950.8141094	1.797938294	0.207638841	8.658969045	4.76E-18	8.91E-17
KLMA_40057	PSO2	interstrand crosslink repair protein	498.0169871	1.439145414	0.220481417	6.52728668	6.70E-11	5.50E-10
KLMA_40058	GAT2	hypothetical protein	393.6871854	1.091593666	0.19743768	5.528801108	3.22E-08	1.82E-07
KLMA_40059	GID8	glucose-induced degradation protein 8	243.566174	1.315262973	0.206798632	6.360114464	2.02E-10	1.55E-09
KLMA_40061		cell wall protein YLR040C	171.9520924	4.729713681	0.371315208	12.73773222	3.65E-37	2.47E-35
KLMA_40062		meiotic recombination protein	10.2993785	2.495706727	0.801337812	3.114425265	0.001843036	0.004129078
KLMA_40068		DNA mismatch repair protein MLH2	132.2281772	3.877969746	0.411790174	9.417344043	4.63E-21	1.08E-19
KLMA_40098	ISA1	iron sulfur assembly-related protein	646.5715374	1.428260353	0.234557923	6.089158417	1.14E-09	7.96E-09
KLMA_40105		uncharacterized glycosyl hydrolase YBR056W	446.8236855	2.169050036	0.242695862	8.937317753	3.99E-19	8.15E-18
KLMA_40106		uncharacterized protein YDR210W	75.80536615	1.52424988	0.299824743	5.083802834	3.70E-07	1.75E-06
KLMA_40117	NRG1	transcriptional regulator NRG1	231.9858597	1.225904739	0.218336629	5.614746104	1.97E-08	1.14E-07
KLMA_40128	HSP26	heat shock protein 26	756.1213352	2.462879127	0.238715523	10.31721396	5.89E-25	2.00E-23
KLMA_40133		uncharacterized sugar kinase YDR109C	1982.729631	1.698564063	0.309282504	5.491950055	3.98E-08	2.21E-07
KLMA_40145	TMA64	translation machinery-associated protein 64	652.314623	1.291149855	0.215715392	5.985432201	2.16E-09	1.45E-08
KLMA_40148		putative aryl-alcohol dehydrogenase YPL088W	493.7320749	1.631912874	0.238333103	6.847193504	7.53E-12	7.05E-11
KLMA_40152	NYV1	vacuolar v-SNARE NYV1	364.2452045	1.161709886	0.182851347	6.353302308	2.11E-10	1.62E-09
KLMA_40172		uncharacterized protein YDR132C	683.9139554	1.555578497	0.141642854	10.98240014	4.64E-28	2.01E-26
KLMA_40173	AHP1	peroxiredoxin type-2	5236.618332	1.973801893	0.276630237	7.135163222	9.67E-13	1.01E-11
KLMA_40181		hypothetical protein	165.1462997	1.043176228	0.24210782	4.308725871	1.64E-05	5.83E-05
KLMA_40198	UGA1	4-aminobutyrate aminotransferase	1650.097234	3.360683204	0.303137097	11.08634753	1.46E-28	6.43E-27
KLMA_40202	ENA5	sodium transport ATPase 5	495.6445977	2.627482203	0.264196974	9.945163895	2.65E-23	7.56E-22
KLMA_40212		solute carrier family 2	206.597566	4.169176996	0.368172114	11.32398906	9.98E-30	4.60E-28
KLMA_40213		MOG interacting and ectopic P-granules protein	475.7809636	2.767336426	0.356306792	7.766723748	8.05E-15	1.06E-13
KLMA_40215	CDC13	cell division control protein 13	443.3896016	1.092553108	0.232332838	4.702534173	2.57E-06	1.07E-05

Locus_tag	UniProt _gene	Product	baseMean	log_2FoldChange	lfcSE	stat	pvalue	padj
KLMA_40216		uncharacterized protein YNL193W	455.2188191	3.006078706	0.32195122	9.337062623	9.90E-21	2.29E-19
KLMA_40217	CHS1	chitin synthase 1	1824.862783	1.258720888	0.237514217	5.299560186	1.16E-07	5.99E-07
KLMA_40230		hypothetical protein	75.65480635	1.778679779	0.345816444	5.143421628	2.70E-07	1.30E-06
KLMA_40231	SPO14	phospholipase D1	182.3936214	1.063473061	0.293069885	3.628735386	0.000284813	0.000765562
KLMA_40244	PDR5	ATP-dependent permease PDR15	543.784625	2.719842044	0.299017971	9.095915	9.38E-20	2.02E-18
KLMA_40246		hypothetical protein	264.9013118	1.649409962	0.208536673	7.90944796	2.59E-15	3.60E-14
KLMA_40247	RTC3	SDO1-like protein YHR087W	243.0724462	4.474415421	0.341240261	13.11221429	2.80E-39	2.17E-37
KLMA_40260		hypothetical protein	5.08612249	5.562896157	1.50597452	3.693884646	0.000220854	0.000610788
KLMA_40262	YPS7	aspartic proteinase yapsin-7	722.4430116	1.641552597	0.195326179	8.40416071	4.31E-17	7.26E-16
KLMA_40271		zinc finger protein	18.6647369	7.438410921	1.284882895	5.789174213	7.07E-09	4.42E-08
KLMA_40272		v-type proton ATPase catalytic subunit A	258.3423796	1.881732123	0.319477095	5.890037671	3.86E-09	2.52E-08
KLMA_40276	SAE2	hypothetical protein	106.0539711	1.041876181	0.273921184	3.803561905	0.00014263	0.000413171
KLMA_40282		uncharacterized protein YJL016W/YJL017W	1535.108056	2.260015952	0.134839622	16.76077042	4.72E-63	8.88E-61
KLMA_40288		hypothetical protein	66.63328198	2.075879628	0.322693699	6.432972305	1.25E-10	9.89E-10
KLMA_40293		leucine-rich repeat-containing protein 6	54.59444021	2.147048336	0.476508975	4.505787823	6.61E-06	2.54E-05
KLMA_40316	ELM1	probable myosin light chain kinase DDB_G0271550	67.3873092	1.551324397	0.270857295	5.727460279	1.02E-08	6.19E-08
KLMA_40333		uncharacterized protein YCR075W-A	43.51401298	1.845896508	0.398187062	4.635752096	3.56E-06	1.44E-05
KLMA_40335		pyruvate dehydrogenase kinase	237.4506302	1.671600334	0.235743683	7.090753456	1.33E-12	1.36E-11
KLMA_40351		hypothetical protein	433.4302173	3.572341831	0.305324366	11.70015313	1.27E-31	6.61E-30
KLMA_40358		protein ASI2	156.3056201	1.08017323	0.208906554	5.1706048	2.33E-07	1.14E-06
KLMA_40359		putative agmatinase 2	513.5803992	2.317251418	0.228077512	10.15992937	2.99E-24	9.55E-23
KLMA_40368	LYS1	saccharopine dehydrogenase [NAD+	1851.503167	1.062769419	0.215206958	4.938359936	7.88E-07	3.56E-06
KLMA_40373	OM45	hypothetical protein	714.8877424	2.296844832	0.37709656	6.09086657	1.12E-09	7.89E-09
KLMA_40383	BLI1	hypothetical protein	46.52111069	1.035645481	0.320445671	3.231891007	0.00122974	0.002853745
KLMA_40391	MDH2	malate dehydrogenase	6581.989059	1.100173757	0.181744712	6.053401743	1.42E-09	9.78E-09
KLMA_40399	GAS4	glycolipid-anchored surface protein 4	149.4225897	1.070703329	0.2321707	4.611707375	3.99E-06	1.60E-05

Locus_tag	UniProt _gene	Product	baseMean	log_2FoldChange	lfcSE	stat	pvalue	padj
		precursor						
KLMA_40403	VTC1	vacuolar transporter chaperone 1	928.1374545	1.291254267	0.273168757	4.72694712	2.28E-06	9.55E-06
KLMA_40411	HOP1	meiosis-specific protein HOP1	99.17097402	1.864071956	0.370899478	5.025814445	5.01E-07	2.33E-06
KLMA_40446		conserved hypothetical membrane protein	21.48334092	2.668329267	0.752174755	3.547485805	0.000388927	0.001011017
KLMA_40459	YET3	endoplasmic reticulum transmembrane protein 3	417.1839625	1.119791133	0.240483195	4.656421554	3.22E-06	1.31E-05
KLMA_40469	CST9	hypothetical protein	140.4759977	2.203221042	0.309097212	7.127922728	1.02E-12	1.05E-11
KLMA_40470	IDP1	isocitrate dehydrogenase [NADP]	2360.886824	1.55482984	0.19658828	7.909066803	2.59E-15	3.60E-14
KLMA_40476		myosin light chain 2	225.1314067	1.139873821	0.198291258	5.748482481	9.00E-09	5.51E-08
KLMA_40484		hypothetical protein	36.13765357	4.1312402	0.637518197	6.480191808	9.16E-11	7.38E-10
KLMA_40532	PUN1	cell membrane protein YLR414C	1299.611205	3.879160965	0.38582347	10.05423793	8.80E-24	2.65E-22
KLMA_40533	PXA2	peroxisomal long-chain fatty acid import protein 1	241.9771316	3.102700729	0.249518897	12.43473248	1.69E-35	1.05E-33
KLMA_40536	NPY1	NADH pyrophosphatase	181.2651603	1.168302147	0.25619928	4.560130486	5.11E-06	2.00E-05
KLMA_40551	LEA1	U2 small nuclear ribonucleoprotein A'	123.2363397	1.043979738	0.259841524	4.017755599	5.88E-05	0.000183831
KLMA_40564		hypothetical protein	27.4342946	4.746767048	0.971509407	4.885971267	1.03E-06	4.56E-06
KLMA_40565	FMP40	UPF0061 protein FMP40	486.3474155	1.081867456	0.23912021	4.524366449	6.06E-06	2.35E-05
KLMA_40605	RAD33	DNA repair protein RAD33	205.2599083	1.182108692	0.201943545	5.853659213	4.81E-09	3.07E-08
KLMA_40621		hypothetical protein	38.24699185	2.577190303	0.626651942	4.112634353	3.91E-05	0.00012822
KLMA_40624	adh	alcohol dehydrogenase	4239.399453	2.035434392	0.171579959	11.86289124	1.84E-32	9.79E-31
KLMA_40625	THI4	thiazole biosynthetic enzyme	302.2836399	1.100776108	0.179822098	6.121472933	9.27E-10	6.63E-09
KLMA_40627	GEX1	siderophore iron transporter ARN1	1064.804808	1.989158546	0.610972407	3.255725664	0.00113103	0.002641774
KLMA_40628		NADPH-dependent methylglyoxal reductase GRE2	89.44986712	1.306193005	0.342883916	3.809432124	0.000139286	0.000404444
KLMA_40629		transposon Ty1-H Gag-Pol polyprotein	55.32656103	1.141518298	0.321970077	3.545417357	0.000391992	0.001017803
KLMA_40630	TY2B-GR2	transposon Ty2-F/Ty2-GR2 Gag-Pol polyprotein	14.86226405	2.126483954	0.692060172	3.072686511	0.002121412	0.004690347
KLMA_50012	ALD4	potassium-activated aldehyde dehydrogenase	9788.172554	7.554556679	0.178401552	42.34580132	0	0

Locus_tag	UniProt _gene	Product	baseMean	log_2FoldChange	lfcSE	stat	pvalue	padj
KLMA_50017	BDH2	(2R,3R)-2,3-butanediol dehydrogenase	2434.139407	1.902884193	0.239289995	7.952209599	1.83E-15	2.61E-14
KLMA_50021		zinc-type alcohol dehydrogenase-like protein C2E1P3.01	347.4903697	1.027613732	0.26761223	3.839935607	0.000123067	0.000362519
KLMA_50022	GDE1	glycerophosphodiester phosphodiesterase GDE1	780.0108984	1.272822896	0.202359878	6.289897529	3.18E-10	2.37E-09
KLMA_50023		hypothetical protein	671.7423865	4.808087417	0.217980577	22.05741209	8.11E-108	6.70E-105
KLMA_50024	CAR1	arginase	254.2661282	2.199468257	0.239379831	9.188193693	3.99E-20	8.83E-19
KLMA_50025	PEX25	peroxisomal membrane protein PEX25	436.5878513	1.207940161	0.169656324	7.119924171	1.08E-12	1.11E-11
KLMA_50032	HGT1	high-affinity glucose transporter	3772.842969	5.61229215	0.282575735	19.86119633	8.82E-88	4.41E-85
KLMA_50049	THI72	thiamine transporter THI72	204.0180411	1.001974515	0.252002405	3.976051398	7.01E-05	0.000216456
KLMA_50051	VPS21	vacuolar protein sorting-associated protein 21	294.0847304	1.035630429	0.221522161	4.675064666	2.94E-06	1.21E-05
KLMA_50065	BTN1	protein BTN1	314.6710397	1.226978745	0.168868157	7.265897638	3.71E-13	4.08E-12
KLMA_50078	DPB3	DNA polymerase epsilon subunit C	80.70589921	1.591975524	0.422238988	3.770318631	0.000163039	0.000463765
KLMA_50091		COG5647 (Cullin	373.2377103	1.750745319	0.248495102	7.045391647	1.85E-12	1.84E-11
KLMA_50092		hypothetical protein	336.4860254	1.224694937	0.243333808	5.032983075	4.83E-07	2.25E-06
KLMA_50093		COMPASS component SHG1	89.50588294	1.054288209	0.319285436	3.30202411	0.000959898	0.002285841
KLMA_50094	POP4	RNases MRP/P 32.9 kDa subunit	317.4171388	1.338870498	0.252115764	5.310538604	1.09E-07	5.65E-07
KLMA_50106	PRP45	pre-mRNA-processing protein 45	258.544702	1.028869299	0.210285817	4.892718457	9.95E-07	4.41E-06
KLMA_50115	SAW1	single-strand annealing weakened protein 1	144.3701192	1.203154484	0.226584943	5.309948973	1.10E-07	5.67E-07
KLMA_50123		uncharacterized CDP-alcohol phosphatidyltransferase class-I family protein with HAD_like super family	2106.284556	4.343235633	0.259166977	16.7584454	4.91E-63	8.89E-61
KLMA_50149	KAP122	pleiotropic drug resistance regulatory protein 6	332.4917937	1.187527674	0.175125486	6.781010007	1.19E-11	1.09E-10
KLMA_50151	ARO8	aromatic amino acid aminotransferase 1	372.6393015	1.701952998	0.261746853	6.502286392	7.91E-11	6.45E-10
KLMA_50156		uncharacterized endoplasmic reticulum membrane protein YGL010W	517.6336347	1.342068666	0.33368273	4.021990182	5.77E-05	0.00018102

Locus_tag	UniProt _gene	Product	baseMean	log_2FoldChange	lfcSE	stat	pvalue	padj
KLMA_50160		BAR super family and SH3	49.72899554	2.493574299	0.503014194	4.957264284	7.15E-07	3.25E-06
KLMA_50161	DAK1	dihydroxyacetone kinase 1	3528.330863	2.676425951	0.221491072	12.08367421	1.29E-33	7.07E-32
KLMA_50163	ITT1	translation termination inhibitor protein ITT1	426.1004133	1.107891075	0.237998904	4.655025952	3.24E-06	1.32E-05
KLMA_50164		putative transcriptional activator YLR445W	21.80824309	1.710938516	0.483531319	3.538423367	0.000402524	0.001041275
KLMA_50178	CAR2	ornithine aminotransferase	250.8427874	2.847704436	0.297614526	9.568432276	1.09E-21	2.64E-20
KLMA_50198		nested antisense gene NAG1	293.5288316	1.435482613	0.318413337	4.508236454	6.54E-06	2.51E-05
KLMA_50199		hypothetical protein	57.32926229	1.875169479	0.469858819	3.990921106	6.58E-05	0.000204226
KLMA_50202		DUF3445 super family conserved domain	282.2504522	1.458105253	0.177705845	8.20516204	2.30E-16	3.56E-15
KLMA_50225	APS2	AP-2 complex subunit sigma	141.8474223	2.108822829	0.246736494	8.546862256	1.26E-17	2.24E-16
KLMA_50228	ECO1	N-acetyltransferase ECO1	89.39262822	1.23612298	0.323712521	3.818582538	0.000134221	0.000391597
KLMA_50233	QDR3	uncharacterized transporter YBR043C	325.8077077	1.334712093	0.200175708	6.667702618	2.60E-11	2.27E-10
KLMA_50236	FIG1	factor-induced gene 1 protein	107.2377919	6.546218147	1.332449648	4.912919717	8.97E-07	4.01E-06
KLMA_50249		uncharacterized membrane protein YJL163C	1738.094332	1.940451327	0.154079236	12.59385354	2.28E-36	1.45E-34
KLMA_50251		J protein JJJ2	350.0782455	1.93711026	0.39545541	4.898429031	9.66E-07	4.30E-06
KLMA_50252		mitochondrial membrane protein FMP33	70.92057303	2.151082178	0.286663048	7.503869776	6.20E-14	7.35E-13
KLMA_50253	PIR1	cell wall mannoprotein HSP150	12771.61535	1.166779585	0.156257254	7.467042702	8.20E-14	9.61E-13
KLMA_50265	SOP4	protein SOP4	348.2121304	1.098179227	0.169408898	6.482417619	9.03E-11	7.29E-10
KLMA_50268		hypothetical protein	63.0170567	4.832888141	1.093756323	4.418615043	9.93E-06	3.67E-05
KLMA_50296	sdh	uncharacterized oxidoreductase YMR226C	652.1200418	1.62073309	0.182083591	8.901038715	5.53E-19	1.11E-17
KLMA_50300		hypothetical protein	877.9531853	3.779046086	0.366690239	10.3058268	6.63E-25	2.23E-23
KLMA_50304	GLT1	glutamate synthase [NADH]	5801.108729	1.231919778	0.257931009	4.776160046	1.79E-06	7.66E-06
KLMA_50310		protein UGX2	640.0783637	1.24072677	0.259591564	4.779534252	1.76E-06	7.55E-06
KLMA_50315	SFA1	S-(hydroxymethyl)glutathione dehydrogenase	3388.669075	1.50229903	0.166356396	9.030605787	1.71E-19	3.61E-18
KLMA_50327		uncharacterized MFS-type transporter	122.5164798	1.902528233	0.328645453	5.788999107	7.08E-09	4.42E-08

Locus_tag	UniProt _gene	Product	baseMean	log_2FoldChange	lfcSE	stat	pvalue	padj
		C1271.10c						
KLMA_50329		GAL4-like Zn2Cys6 binuclear cluster DNA-binding conserved domain	216.9238898	2.624964544	0.248598133	10.55906782	4.61E-26	1.77E-24
KLMA_50339	HUT1	UDP-galactose transporter homolog 1	316.6059337	1.156151755	0.191118616	6.049393725	1.45E-09	9.99E-09
KLMA_50343		tubulin-specific chaperone C	69.38482064	1.872758556	0.306979572	6.100596672	1.06E-09	7.51E-09
KLMA_50364	RAG1	low-affinity glucose transporter	314.49797	2.216356265	0.413944575	5.354234355	8.59E-08	4.52E-07
KLMA_50369	IST3	U2 snRNP component IST3	67.16242001	1.375959295	0.311302074	4.420013266	9.87E-06	3.65E-05
KLMA_50379	HAK1	high affinity potassium transporter	29992.82726	7.19044832	0.214562838	33.51208619	3.21E-246	7.85E-243
KLMA_50383		dynactin subunit 5	37.02605452	1.188864053	0.345629707	3.439704487	0.00058235	0.001451861
KLMA_50392	VAB2	protein VAB2	398.3474666	1.025151806	0.21672753	4.730141143	2.24E-06	9.43E-06
KLMA_50415	AIM17	uncharacterized oxidoreductase YHL021C	229.8689876	2.637971494	0.26453869	9.971968524	2.02E-23	5.84E-22
KLMA_50419	CTT1	catalase T	1035.374366	3.240695668	0.284181026	11.4036314	4.01E-30	1.90E-28
KLMA_50437		NADH-dependent flavin oxidoreductase (predicted); FlaRed super family conserved domain	74.94469985	1.251256084	0.358048353	3.494656723	0.000474672	0.001206109
KLMA_50440	TEL2	telomere length regulation protein TEL2	264.9920132	1.1937198	0.266805078	4.474126984	7.67E-06	2.90E-05
KLMA_50450	OPT2	oligopeptide transporter 2	516.4656471	1.328124258	0.184616736	7.193953739	6.29E-13	6.68E-12
KLMA_50451		acyl-coenzyme A:6-aminopenicillanic-acid-acyltransferase 40 kDa form	28.77476762	4.577056334	0.74237968	6.165384721	7.03E-10	5.10E-09
KLMA_50456		hypothetical protein	32.60904014	2.641333269	0.465072639	5.679399405	1.35E-08	8.04E-08
KLMA_50457	AXL1	putative protease AXL1	108.3155563	1.482023049	0.293075156	5.056802054	4.26E-07	2.00E-06
KLMA_50458		uncharacterized protein YGR111W	215.1139374	2.410730669	0.270843892	8.900812394	5.54E-19	1.11E-17
KLMA_50462	TRS20	transport protein particle 20 kDa subunit	55.16275582	1.144324703	0.310709429	3.682941673	0.000230558	0.000634034
KLMA_50463	MTC4	conserved hypothetical membrane protein	275.883025	1.171550891	0.206445569	5.674865766	1.39E-08	8.25E-08
KLMA_50468	BAR1	aspartic proteinase yapsin-3	171.6170954	1.073743985	0.272089089	3.946295643	7.94E-05	0.000242579
KLMA_50482	NCE102	non-classical export protein 2	1206.299814	1.7729939	0.234621676	7.556820542	4.13E-14	5.03E-13
KLMA_50484	APC2	anaphase-promoting complex subunit	195.818692	1.221540984	0.21823426	5.597384144	2.18E-08	1.25E-07

Locus_tag	UniProt _gene	Product	baseMean	log$_2$FoldChange	lfcSE	stat	pvalue	padj
		2						
KLMA_50485	DCN1	defective in cullin neddylation protein 1	129.215156	1.318495682	0.29564009	4.45980003	8.20E-06	3.08E-05
KLMA_50488	INO1	inositol-3-phosphate synthase	6465.302614	2.748356613	0.341886286	8.038803317	9.07E-16	1.34E-14
KLMA_50496	VPS70	vacuolar protein sorting-associated protein 70	1318.249977	1.243147106	0.18700253	6.647755535	2.98E-11	2.56E-10
KLMA_50504	RKM5	uncharacterized protein YLR137W	67.37089533	1.214251612	0.324238726	3.744930861	0.000180443	0.000511187
KLMA_50513	PUT1	proline dehydrogenase	1015.919942	1.305923027	0.185652012	7.034251936	2.00E-12	1.99E-11
KLMA_50528	STB3	protein STB3	392.2216966	1.027731172	0.211386837	4.861850362	1.16E-06	5.12E-06
KLMA_50530	PCD1	peroxisomal coenzyme A diphosphatase 1	158.4496338	1.050517494	0.199772682	5.25856429	1.45E-07	7.37E-07
KLMA_50535	URC1	putative GTP cyclohydrolase URC1	155.7634185	2.174740114	0.273116007	7.96269737	1.68E-15	2.42E-14
KLMA_50587	SPS19	peroxisomal 2	258.7293391	3.822914719	0.309434081	12.35453675	4.60E-35	2.81E-33
KLMA_50589	dpp1	probable diacylglycerol pyrophosphate phosphatase 1	88.84598235	1.744975321	0.357369519	4.882831999	1.05E-06	4.63E-06
KLMA_50601		peroxisomal membrane protein PEX17	346.2726392	1.693435607	0.21979755	7.704524499	1.31E-14	1.68E-13
KLMA_50613	GUD1	probable guanine deaminase	553.4344942	2.44760069	0.318584872	7.682727278	1.56E-14	1.97E-13
KLMA_50615	ATG4	probable cysteine protease ATG4	687.0642127	1.425817318	0.183571721	7.76708584	8.03E-15	1.06E-13
KLMA_60003	yxeK	putative monooxygenase yxeK	417.7077066	1.657819701	0.24126915	6.871246079	6.36E-12	6.00E-11
KLMA_60005		UNC93-like protein	121.27343	1.272696868	0.315677685	4.031633931	5.54E-05	0.000174517
KLMA_60012	KIN28	serine/threonine-protein kinase KIN28	485.9200049	1.667733578	0.209884289	7.945966719	1.93E-15	2.72E-14
KLMA_60013	MRF1	peptide chain release factor 1	315.3250006	1.708863702	0.218125013	7.834331695	4.71E-15	6.39E-14
KLMA_60027	FMP46	putative redox protein FMP46	214.5492087	1.680018447	0.205652849	8.16919606	3.10E-16	4.75E-15
KLMA_60029	ACAD11	acyl-CoA dehydrogenase family member 11	135.5400385	3.276766101	0.490411186	6.681670804	2.36E-11	2.07E-10
KLMA_60030		hypothetical protein	775.9766048	1.343532911	0.251028442	5.352114296	8.69E-08	4.57E-07
KLMA_60032	NIT2	probable hydrolase NIT2	542.2568403	1.181284278	0.203198764	5.813442248	6.12E-09	3.86E-08
KLMA_60039		uncharacterized protein YJL132W	644.8477331	2.091646364	0.203375823	10.28463629	8.27E-25	2.76E-23
KLMA_60049		hypothetical protein	4.371601739	5.345064621	1.455719053	3.671769364	0.000240877	0.000659072

Locus_tag	UniProt _gene	Product	baseMean	log_2FoldChange	lfcSE	stat	pvalue	padj
KLMA_60051		probable 26S proteasome regulatory subunit p28	225.3193884	1.227941565	0.253589261	4.842245922	1.28E-06	5.62E-06
KLMA_60053		spo12 super family protein	37.35116111	1.095293271	0.37216678	2.943017297	0.003250303	0.006929061
KLMA_60059	HSV2	SVP1-like protein 2	193.3066412	1.448713671	0.217724577	6.653882135	2.85E-11	2.48E-10
KLMA_60074	DIP5	dicarboxylic amino acid permease	278.2154943	1.507510051	0.320595983	4.702211295	2.57E-06	1.07E-05
KLMA_60085	TDA10	uncharacterized kinase YGR205W	183.4732898	1.326212999	0.251980587	5.263155453	1.42E-07	7.20E-07
KLMA_60086	TES1	peroxisomal acyl-coenzyme A thioester hydrolase 1	368.8381457	1.485259169	0.214549587	6.922684807	4.43E-12	4.28E-11
KLMA_60089	YCH1	uncharacterized protein YGR203W	379.3907602	2.513930609	0.220168107	11.41823239	3.39E-30	1.64E-28
KLMA_60101	HSP78	heat shock protein 78	1053.795514	1.192030543	0.257118672	4.636110367	3.55E-06	1.44E-05
KLMA_60105		hypothetical protein	50.72216832	1.746449979	0.407722319	4.28342992	1.84E-05	6.47E-05
KLMA_60120	RMD1	sporulation protein RMD1	311.4973524	1.157805288	0.179647366	6.444877605	1.16E-10	9.20E-10
KLMA_60123		RCR super family protein	436.4946765	1.454959082	0.281652675	5.165791815	2.39E-07	1.17E-06
KLMA_60124		RCR super family protein	121.6783051	3.40304502	0.419955404	8.103348565	5.35E-16	8.01E-15
KLMA_60125	gabD	succinate-semialdehyde dehydrogenase [NADP+]	675.995075	3.829405806	0.374475037	10.2260643	1.52E-24	4.97E-23
KLMA_60132	APC11	anaphase-promoting complex subunit 11	58.51862821	1.48631785	0.299833504	4.957143988	7.15E-07	3.25E-06
KLMA_60161		hypothetical protein	8.639724054	6.326817224	1.323881905	4.778989123	1.76E-06	7.56E-06
KLMA_60165	GPA1	guanine nucleotide-binding protein alpha-1 subunit	44.13910922	4.432487995	0.563811587	7.861647572	3.79E-15	5.19E-14
KLMA_60176	JLP1	alpha-ketoglutarate-dependent sulfonate dioxygenase	208.2326175	2.189417631	0.302346163	7.241426876	4.44E-13	4.83E-12
KLMA_60180	STL1	sugar transporter STL1	286.9688094	2.121138078	0.683142482	3.104971707	0.001902972	0.004245828
KLMA_60195		tRNA_bindingDomain super family protein	38.90658186	1.220033254	0.400375806	3.047220224	0.002309684	0.005081305
KLMA_60206		probable 5-hydroxyisourate hydrolase	120.7368482	1.273366018	0.247207104	5.151008995	2.59E-07	1.26E-06
KLMA_60208	VCX1	vacuolar calcium ion transporter	183.8798491	2.613761462	0.392721736	6.655504967	2.82E-11	2.45E-10
KLMA_60221	PUT4	proline-specific permease	1072.829003	2.105423976	0.256300872	8.214657887	2.13E-16	3.31E-15
KLMA_60241		uncharacterized vacuolar membrane protein SCY_4679	1004.375441	1.314770738	0.198504225	6.623389197	3.51E-11	2.99E-10
KLMA_60268		uncharacterized protein YNL134C	2752.893253	2.574786951	0.25173117	10.22831998	1.48E-24	4.89E-23

Locus_tag	UniProt _gene	Product	baseMean	log_2FoldChange	lfcSE	stat	pvalue	padj
KLMA_60271	ETR1	probable trans-2-enoyl-CoA reductase	1255.102962	1.471637644	0.255342345	5.763390497	8.24E-09	5.10E-08
KLMA_60273		hypothetical protein	36.25356841	1.207720042	0.389524171	3.100500893	0.001931936	0.004302589
KLMA_60293		LPLAT super family protein	58.38953679	1.79748793	0.390299445	4.605407344	4.12E-06	1.65E-05
KLMA_60312	URC4	uracil catabolism protein 4	458.6027232	1.015176904	0.22545624	4.502766941	6.71E-06	2.57E-05
KLMA_60321	ETP1	RING finger protein ETP1	336.0676858	1.229684567	0.236739486	5.19425207	2.06E-07	1.01E-06
KLMA_60323		uncharacterized transporter YHL008C	293.9063765	1.363798439	0.237464613	5.743164933	9.29E-09	5.67E-08
KLMA_60327	AO-I	copper amine oxidase 1	324.7303144	1.655080786	0.226131951	7.319093029	2.50E-13	2.77E-12
KLMA_60355		ULP1-interacting protein 4	697.6293447	1.985307945	0.26395219	7.521467986	5.42E-14	6.48E-13
KLMA_60356	MF(ALPHA)1	mating factor alpha-1	101.6657033	4.494196437	0.465231494	9.660129413	4.45E-22	1.12E-20
KLMA_60362	SCY1	protein kinase-like protein SCY1	605.2154789	1.470472919	0.347361058	4.233269344	2.30E-05	7.95E-05
KLMA_60366		uncharacterized protein YGL082W	1264.40348	1.014133305	0.216121825	4.692415048	2.70E-06	1.11E-05
KLMA_60367		hypothetical membrane protein	964.3397269	2.087140684	0.245918203	8.487133776	2.12E-17	3.67E-16
KLMA_60368		hypothetical protein	115.0938994	2.407593664	0.445447833	5.404883544	6.49E-08	3.48E-07
KLMA_60369	RSA1	ribosome assembly 1 protein	148.3034138	1.209481498	0.33234139	3.639274353	0.000273407	0.000738155
KLMA_60370	FMP37	UPF0041 protein FMP37	499.8417217	1.094302115	0.186269943	5.874818554	4.23E-09	2.75E-08
KLMA_60377	RAD1	DNA repair protein RAD1	278.7579774	1.181492012	0.247922972	4.765560862	1.88E-06	8.05E-06
KLMA_60387	PHO85	negative regulator of the PHO system	971.3791286	1.31400472	0.148649076	8.839642715	9.60E-19	1.88E-17
KLMA_60404		hypothetical protein	261.0644312	1.1949176	0.202133548	5.911525378	3.39E-09	2.23E-08
KLMA_60405		37S ribosomal protein YMR-31	278.5154782	1.113255162	0.200377516	5.555788813	2.76E-08	1.57E-07
KLMA_60406	PDE1	low-affinity cyclic AMP phosphodiesterase	380.7269714	2.423339706	0.240678036	10.06880289	7.59E-24	2.30E-22
KLMA_60409	ZIP2	protein ZIP2	308.5841076	6.088535001	0.447486278	13.60608203	3.68E-42	3.16E-40
KLMA_60413		uncharacterized esterase/lipase C417.12	314.0601026	1.679557012	0.19722602	8.51589974	1.65E-17	2.90E-16
KLMA_60414	DAL5	allantoate permease	344.8058134	5.220350291	0.686902408	7.599842758	2.96E-14	3.65E-13
KLMA_60415		uncharacterized esterase/lipase C417.12	222.9096169	1.682090941	0.217575905	7.731053406	1.07E-14	1.39E-13
KLMA_60423	ARO9	aromatic amino acid	429.8102448	1.483246193	0.203518526	7.288015597	3.15E-13	3.48E-12

Locus_tag	UniProt _gene	Product	baseMean	$log_2FoldChange$	lfcSE	stat	pvalue	padj
		aminotransferase 2						
KLMA_60425	GRE2	NADPH-dependent methylglyoxal reductase GRE2	1669.566386	1.170883988	0.141718276	8.262053594	1.43E-16	2.27E-15
KLMA_60426		putative lipase YJR107W	10.71199019	2.160730976	0.711329299	3.037595918	0.002384735	0.005232276
KLMA_60443	SST2	protein SST2	610.2201999	2.082805595	0.209881184	9.923736656	3.28E-23	9.32E-22
KLMA_60450		platinum sensitivity protein 3	242.1795528	1.413432638	0.265566488	5.322330572	1.02E-07	5.33E-07
KLMA_60452	FBP1	fructose-1,6-bisphosphatas	265.6374308	2.87567171	0.284688061	10.10113211	5.46E-24	1.68E-22
KLMA_60454	ATG3	autophagy-related protein 3	486.8621491	1.932224469	0.233322469	8.281347614	1.22E-16	1.96E-15
KLMA_60460	INP1	inheritance of peroxisomes protein 1	350.0937195	1.136382351	0.211177195	5.3811793	7.40E-08	3.93E-07
KLMA_60475		uncharacterized protein YIL077C	330.0619501	1.191658337	0.233411644	5.105393711	3.30E-07	1.58E-06
KLMA_60487	VCX1	vacuolar calcium ion transporter	2714.56491	1.636416311	0.204489603	8.00244259	1.22E-15	1.78E-14
KLMA_60495	SRF1	hypothetical membrane protein	313.1370028	1.34323083	0.238973494	5.620836047	1.90E-08	1.10E-07
KLMA_60496		uncharacterized protein YDL183C	440.0985563	1.686570853	0.212124837	7.950841027	1.85E-15	2.62E-14
KLMA_60498		uncharacterized protein YDL186W	124.9888369	3.490084252	0.483325666	7.220978519	5.16E-13	5.53E-12
KLMA_60500		probable allantoinase 1	164.768399	2.040174428	0.253064518	8.061874682	7.51E-16	1.12E-14
KLMA_60522	CRR1	probable glycosidase CRR1	254.3207073	1.879668907	0.333338567	5.63891819	1.71E-08	9.97E-08
KLMA_60551		uncharacterized protein YBR053C	843.6709614	1.059337812	0.212882695	4.976157467	6.49E-07	2.96E-06
KLMA_60552	ERC1	uncharacterized transporter C4B3.13	585.6484161	1.289057844	0.143122824	9.006654648	2.12E-19	4.42E-18
KLMA_60558		aminotriazole resistance protein	398.4774814	1.806939594	0.244162326	7.400566758	1.36E-13	1.55E-12
KLMA_70001		aminotriazole resistance protein	206.5461504	1.279673078	0.292802327	4.370433429	1.24E-05	4.50E-05
KLMA_70002		arylsulfatase	217.3405221	1.412618752	0.365498945	3.864905137	0.000111132	0.000331358
KLMA_70003	HXT15	hexose transporter HXT9	227.694327	2.930841636	0.4214497	6.954190829	3.55E-12	3.46E-11
KLMA_70004		conserved hypothetical protein	501.4625286	1.825976019	0.367910262	4.96310162	6.94E-07	3.16E-06
KLMA_70011		uncharacterized protein YJL171C	3252.703975	1.160358568	0.225312677	5.149992378	2.60E-07	1.26E-06
KLMA_70012		1-aminocyclopropane-1-carboxylate oxidase	514.7806564	1.817668084	0.220912509	8.227999833	1.90E-16	2.98E-15
KLMA_70023	PRM4	pheromone-regulated membrane protein 4	644.968071	1.665463495	0.246672451	6.751720718	1.46E-11	1.32E-10
KLMA_70024	KIP2	kinesin-like protein KIP2	541.5999095	1.799076975	0.223814739	8.038241733	9.11E-16	1.34E-14

Locus_tag	UniProt _gene	Product	baseMean	log_2FoldChange	lfcSE	stat	pvalue	padj
KLMA_70030		protein SIC1	261.0828951	1.031165618	0.203964497	5.055613282	4.29E-07	2.01E-06
KLMA_70033	FMP32	protein FMP32	142.9045615	1.130672012	0.246247109	4.591615374	4.40E-06	1.75E-05
KLMA_70040	OTU1	ubiquitin thioesterase OTU1	168.0898734	1.406537274	0.236914532	5.936897423	2.90E-09	1.92E-08
KLMA_70044	SOR1	sorbitol dehydrogenase 1	8515.325102	3.766594126	0.223676663	16.83945959	1.25E-63	2.66E-61
KLMA_70053		transmembrane protein 208 homolog	484.5961906	1.585578829	0.183547635	8.638514062	5.69E-18	1.06E-16
KLMA_70055	PER33	UPF0121 membrane protein YLR064W	768.9335377	1.094808302	0.179465611	6.100379325	1.06E-09	7.51E-09
KLMA_70089		conserved hypothetical membrane protein	17.99417167	2.149715938	0.611703103	3.514312628	0.000440894	0.001126805
KLMA_70094		hypothetical protein	234.9838094	2.78774122	0.248472468	11.21951757	3.27E-29	1.48E-27
KLMA_70102	YMR1	phosphoinositide 3-phosphatase	550.515876	1.032150855	0.194729803	5.300425708	1.16E-07	5.96E-07
KLMA_70103	MOH1	protein yippee-like MOH1	746.907493	1.993272001	0.346512803	5.752376194	8.80E-09	5.42E-08
KLMA_70107		hypothetical protein	419.0820662	2.295677149	0.282029231	8.139855371	3.96E-16	6.02E-15
KLMA_70117	ERV2	FAD-linked sulfhydryl oxidase ERV2	343.5785608	1.181034637	0.240221659	4.916436929	8.81E-07	3.95E-06
KLMA_70118		guanine nucleotide-binding protein subunit gamma	40.29053379	4.301905414	0.623768761	6.896634916	5.32E-12	5.06E-11
KLMA_70132	NTO1	nuA3 HAT complex component NTO1	535.5766334	1.085127445	0.201018493	5.398147342	6.73E-08	3.61E-07
KLMA_70139	ATH1	vacuolar acid trehalase	801.1242488	1.123472682	0.292160846	3.845390976	0.00012036	0.000356051
KLMA_70145	ywtG	conserved hypothetical membrane protein	317.3002229	3.48861536	0.678883034	5.138757616	2.77E-07	1.33E-06
KLMA_70146		uncharacterized protein YBL095W	337.8929403	1.06612706	0.195098947	5.464545447	4.64E-08	2.55E-07
KLMA_70154	TEL1	serine/threonine-protein kinase TEL1	680.809854	1.11592769	0.248126283	4.497418324	6.88E-06	2.62E-05
KLMA_70156		conserved hypothetical protein	84.43110807	1.295534615	0.411408153	3.149025135	0.001638161	0.003704064
KLMA_70172	SEE1	uncharacterized methyltransferase YIL064W	277.9121662	1.015450116	0.183199167	5.542875172	2.98E-08	1.69E-07
KLMA_70179	ICL1	isocitrate lyase	88.97440535	2.107149473	0.53932291	3.907027562	9.34E-05	0.000282572
KLMA_70189	MMF1	protein MMF1	632.7361859	1.107964765	0.173485082	6.386513197	1.70E-10	1.33E-09
KLMA_70199	ALG12	dolichyl-P-Man:Man(7)GlcNAc(2)-PP-dolichyl-alpha-1	2032.947333	1.011477228	0.133254103	7.590589744	3.18E-14	3.91E-13
KLMA_70200		uncharacterized protein YNR029C	387.2829207	1.183821813	0.185006292	6.398819186	1.57E-10	1.23E-09

Locus_tag	UniProt _gene	Product	baseMean	log_2FoldChange	lfcSE	stat	pvalue	padj
KLMA_70209		uncharacterized PH domain-containing protein YPR091C	377.800751	2.080719786	0.233503802	8.910860403	5.06E-19	1.02E-17
KLMA_70214	AGP2	general amino acid permease AGP2	1209.00893	1.543164826	0.180069226	8.569842035	1.04E-17	1.85E-16
KLMA_70220	USV1	conserved hypothetical protein	371.6169436	1.001610528	0.244085198	4.103528345	4.07E-05	0.000132133
KLMA_70230		hypothetical protein	3782.328386	6.052276523	0.274395532	22.05676046	8.23E-108	6.70E-105
KLMA_70233		uncharacterized protein YLR211C	283.3690025	1.100470956	0.193381462	5.690674506	1.27E-08	7.57E-08
KLMA_70236	ATG9	autophagy-related protein 9	409.8842218	1.185435278	0.228938929	5.177954145	2.24E-07	1.10E-06
KLMA_70242		conserved hypothetical protein	139.5148873	2.059344879	0.341182138	6.03591059	1.58E-09	1.08E-08
KLMA_70258	PHO84	inorganic phosphate transporter	9008.079883	5.362272787	0.584561722	9.173150723	4.59E-20	1.01E-18
KLMA_70266	FMP27	protein FMP27	1238.005922	1.152922887	0.261708006	4.405378754	1.06E-05	3.89E-05
KLMA_70270		uncharacterized protein C1F7.10	107.5800892	1.361781078	0.246318847	5.528529767	3.23E-08	1.82E-07
KLMA_70301	ANT1	peroxisomal adenine nucleotide transporter 1	586.7903661	2.276035432	0.213160054	10.67758893	1.30E-26	5.15E-25
KLMA_70303		probable 6-phosphofructo-2-kinase/fructose-2,6-biphosphatase	219.2793861	1.782376479	0.24601875	7.24488063	4.33E-13	4.72E-12
KLMA_70317		uncharacterized glycosyl hydrolase YIR007W	437.1371806	1.927131806	0.261818144	7.360573921	1.83E-13	2.08E-12
KLMA_70348		uncharacterized protein YBR085C-A	149.2835605	1.854302643	0.275521873	6.730146779	1.69E-11	1.51E-10
KLMA_70361	TEC1	conserved hypothetical protein	139.0558023	2.268054227	0.263110139	8.620170387	6.69E-18	1.22E-16
KLMA_70381	PTR2	peptide transporter PTR2	150.2193158	2.402237504	0.337087056	7.126460243	1.03E-12	1.06E-11
KLMA_70392	LPX1	peroxisomal membrane protein LPX1	369.7485438	2.260965875	0.219916498	10.2810198	8.58E-25	2.85E-23
KLMA_70404	FAD1	FAD synthetase	507.0035503	1.439664337	0.26169608	5.501283535	3.77E-08	2.10E-07
KLMA_70416	VFA1	uncharacterized protein YER128W	76.36547475	1.143705371	0.287594244	3.976802018	6.98E-05	0.000215911
KLMA_70426	FOX2	peroxisomal hydratase-dehydrogenase-epimerase	314.3608921	2.10326663	0.210764689	9.979217309	1.88E-23	5.46E-22
KLMA_70428		conserved hypothetical protein	134.4534576	1.357128777	0.315893381	4.296160861	1.74E-05	6.14E-05
KLMA_70429	ICL2	mitochondrial 2-methylisocitrate lyase	732.3865228	4.072975856	0.330538073	12.32225932	6.87E-35	4.09E-33
KLMA_70430		hypothetical protein	254.4437536	1.602538121	0.341454827	4.693265385	2.69E-06	1.11E-05
KLMA_70431		hypothetical protein	1133.53665	2.230347415	0.288349985	7.734862262	1.04E-14	1.36E-13
KLMA_70434		uncharacterized protein YDL144C	31.26571882	1.586119695	0.435406444	3.642848467	0.000269638	0.000729186

Locus_tag	UniProt _gene	Product	baseMean	log_2FoldChange	lfcSE	stat	pvalue	padj
KLMA_70439	SAD1	pre-mRNA-splicing factor SAD1	214.3339286	1.221739236	0.223331442	5.470520526	4.49E-08	2.47E-07
KLMA_70443	PDH1	probable 2-methylcitrate dehydratase	1056.555518	3.726465537	0.235455433	15.82662792	2.04E-56	3.02E-54
KLMA_70444	CIT3	citrate synthase 3	423.7431707	5.440632367	0.287025591	18.95521705	4.00E-80	1.78E-77
KLMA_70447	SNF8	vacuolar-sorting protein SNF8	221.8084295	1.985389089	0.248957413	7.974814111	1.53E-15	2.21E-14
KLMA_70450	ULA1	NEDD8-activating enzyme E1 regulatory subunit	382.0463406	1.097309175	0.190110996	5.771939553	7.84E-09	4.88E-08
KLMA_70455	ADY2	hypothetical protein	830.7702585	1.735978417	0.188642594	9.202473214	3.50E-20	7.80E-19
KLMA_70458	ywnB	uncharacterized protein ywnB	591.0143929	1.808064319	0.185850449	9.728598071	2.28E-22	5.92E-21
KLMA_70459	MCH2	probable transporter MCH2	213.6977078	1.13267586	0.256449688	4.416756632	1.00E-05	3.70E-05
KLMA_70462	FCY2	purine-cytosine permease FCY2	130.4332947	4.09305162	0.458804126	8.921130795	4.62E-19	9.35E-18
KLMA_80004		putative uncharacterized oxidoreductase YGL039W	227.4544403	1.228026269	0.252759834	4.858470777	1.18E-06	5.20E-06
KLMA_80005	LAC12	high-affinity glucose transporter	171.8440177	2.281399818	0.392031486	5.819430074	5.90E-09	3.74E-08
KLMA_80008		conserved hypothetical protein	495.7343798	3.170532668	0.194978329	16.26094901	1.87E-59	3.26E-57
KLMA_80011	PHR1	deoxyribodipyrimidine photo-lyase	225.912537	1.476838484	0.212405416	6.952922921	3.58E-12	3.49E-11
KLMA_80014	hyuA	conserved hypothetical protein	344.5582131	1.1545994	0.19017659	6.071196251	1.27E-09	8.82E-09
KLMA_80038	Pir	pirin-like protein	685.1033002	1.723723593	0.24981258	6.900067205	5.20E-12	4.96E-11
KLMA_80053		DNA-directed RNA polymerase II subunit RPB1	64.1981807	3.320013064	0.408610636	8.125126402	4.47E-16	6.74E-15
KLMA_80056	tsc2	conserved hypothetical protein	214.1774324	1.207556917	0.232591465	5.191750777	2.08E-07	1.02E-06
KLMA_80058		UPF0103 protein YJR008W	440.164588	2.068987597	0.32453104	6.375314969	1.83E-10	1.42E-09
KLMA_80068	mcfL	mitochondrial substrate carrier family protein L	264.3277	1.721908833	0.255872965	6.729545771	1.70E-11	1.52E-10
KLMA_80078	SSK1	conserved hypothetical protein	209.8403369	1.180611881	0.204766674	5.765644677	8.13E-09	5.04E-08
KLMA_80089	TMA17	translation machinery-associated protein 17	199.253766	1.421147315	0.215518756	6.594077192	4.28E-11	3.62E-10
KLMA_80093	ATG20	autophagy-related protein 20	648.0735943	1.359609938	0.270242327	5.031076929	4.88E-07	2.27E-06
KLMA_80094		uncharacterized oxidoreductase SSP0419	45.36253108	2.714918435	0.54061313	5.021924706	5.12E-07	2.37E-06
KLMA_80101	ywtG	conserved hypothetical protein containing the Major Facilitator	1164.256818	5.984991118	0.212643608	28.14564313	2.71E-174	4.41E-171

Locus_tag	UniProt _gene	Product	baseMean	log_2FoldChange	lfcSE	stat	pvalue	padj
		Superfamily (MFS) domain						
KLMA_80105	LYS5	L-aminoadipate-semialdehyde dehydrogenase-phosphopantetheinyl transferase	74.65588446	1.357342675	0.298811933	4.54246476	5.56E-06	2.17E-05
KLMA_80108		protease B inhibitors 2 and 1	883.3465306	3.443229404	0.371352354	9.272135661	1.82E-20	4.14E-19
KLMA_80110		uncharacterized protein YDL121C	172.2274437	1.235879065	0.291190617	4.244226954	2.19E-05	7.60E-05
KLMA_80117	KTR5	probable mannosyltransferase KTR5	148.6874522	1.519451105	0.251090314	6.051412664	1.44E-09	9.89E-09
KLMA_80120	AIM19	loss of respiratory capacity protein 2	173.0965387	2.204543091	0.265592876	8.300460173	1.04E-16	1.68E-15
KLMA_80121	SIW14	tyrosine-protein phosphatase SIW14	371.4547031	1.508816772	0.284993689	5.294211168	1.20E-07	6.14E-07
KLMA_80122	AVT7	vacuolar amino acid transporter 7	1144.089024	1.35634636	0.226719067	5.982497969	2.20E-09	1.47E-08
KLMA_80123	lag1	sphingosine N-acyltransferase lac1	338.3760087	2.065296391	0.229301219	9.006914128	2.12E-19	4.42E-18
KLMA_80136		conserved hypothetical protein containing PIG-H super family	25.00748895	1.261124623	0.444568965	2.836735628	0.004557733	0.009428196
KLMA_80141	SGA1	glucoamylase	447.1482702	2.382864387	0.237301127	10.04152157	1.00E-23	3.00E-22
KLMA_80142	NCE103	carbonic anhydrase	1866.057042	3.109567932	0.24615317	12.63265443	1.39E-36	9.21E-35
KLMA_80161	MSG5	tyrosine-protein phosphatase MSG5	1614.669065	1.070417415	0.231677092	4.620298911	3.83E-06	1.54E-05
KLMA_80176		uncharacterized oxidoreductase YJR096W	339.2055372	4.251626582	0.247589744	17.17206262	4.30E-66	1.05E-63
KLMA_80177	SFC1	succinate/fumarate mitochondrial transporter	1284.809239	1.25828326	0.254753983	4.939209363	7.84E-07	3.55E-06
KLMA_80191	mug70	meiotically up-regulated gene 70 protein	74.10500993	1.250904525	0.284372987	4.398816283	1.09E-05	3.99E-05
KLMA_80192		conserved hypothetical membrane brotien	44.30851123	1.509277758	0.366944239	4.113098391	3.90E-05	0.000128091
KLMA_80226	CRC1	mitochondrial carnitine carrier	206.6167149	4.453528584	0.405038302	10.99532701	4.02E-28	1.75E-26
KLMA_80231		[PSI+] induction protein 2	1824.733822	1.465339124	0.139118125	10.53305686	6.08E-26	2.29E-24
KLMA_80235	VAM3	syntaxin VAM3	147.3544295	1.173435614	0.211624891	5.544884656	2.94E-08	1.67E-07
KLMA_80260	SFK1	protein SFK1	320.2569617	1.346099146	0.1927887	6.982251257	2.90E-12	2.85E-11
KLMA_80262	HOF1	cytokinesis protein 2	432.5166044	1.152338542	0.177697824	6.4848208	8.88E-11	7.18E-10
KLMA_80286	UBA3	NEDD8-activating enzyme E1 catalytic subunit	43.86081784	1.808737015	0.34540807	5.236522173	1.64E-07	8.21E-07

Locus_tag	UniProt _gene	Product	baseMean	$log_2FoldChange$	lfcSE	stat	pvalue	padj
KLMA_80288		hypothetical protein	246.6154645	1.724921704	0.245333958	7.030912962	2.05E-12	2.04E-11
KLMA_80306	ADH3	alcohol dehydrogenase 3	4011.530088	3.596547636	0.196704685	18.28399584	1.11E-74	3.87E-72
KLMA_80318	amdS	putative amidase C550.07	346.4716158	1.081295707	0.189655759	5.701359739	1.19E-08	7.15E-08
KLMA_80332	ARO7	chorismate mutase	449.8324325	1.609601175	0.152180912	10.57689268	3.81E-26	1.49E-24
KLMA_80339	ADH6	NADP-dependent alcohol dehydrogenase 6	903.6790303	2.148704847	0.329852454	6.514139346	7.31E-11	5.99E-10
KLMA_80340	SAS3	histone acetyltransferase SAS3	185.9394351	1.065665172	0.203386514	5.23960588	1.61E-07	8.09E-07
KLMA_80372	ATG8	autophagy-related protein 8	284.305815	2.238524109	0.280273483	7.986927922	1.38E-15	2.00E-14
KLMA_80374		conserved hypothetical protein	22.63143625	5.828412445	1.111367839	5.244359465	1.57E-07	7.90E-07
KLMA_80389	HXT14	hexose transporter HXT14	1359.565003	3.917602092	0.258313977	15.1660477	5.93E-52	6.74E-50
KLMA_80412	GUT1	glycerol kinase	806.7726206	1.963952768	0.151386704	12.97308626	1.74E-38	1.27E-36
KLMA_80427	FCY2	purine-cytosine permease FCY2	406.5200701	3.838064583	0.432191112	8.880480131	6.66E-19	1.32E-17

Supplementary File S2. GO enrichment of differentially expressed genes for *Kmmig1* and the parental strain of *K. marxianus*

GO terms enriched in significantly down-regulated genes (biological process)

GO.ID	Term	Annotated gene	Significant	Expected	P-value	Genes
GO:0042254	ribosome biogenesis	256	94	25.76	< 1e-30	SPB1,UTP5,SDA1,EBP2,RPF2,HAS1,NOG1,UTP13,DBP7,UTP15,UTP11,ARX1,NOP53,RPS2,NOP58,NOP12,ENP1,CBF5,RLP7,UTP8,MPP10,IPI3,RPS3,RPL5,NAN1,MRT4,RRS1,IPI1,DCAF13,DRS1,NOB1,RPL3,RLI1,BFR2,MEX67,PWP2,DBP3,RRP3,RRB1,ECM16,MDN1,UTP10,GAR1,CIC1,ERB1,RRP12,IMP4,YTM1,NOP19,NMD3,RIX1,UTP21,MTR2,BMS1,UTP6,PRP43,NOC2,GEP3,MTR4,UTP18,ENP2,RRP5,PWP1,NOP56,UTP4,NUG1,NOP7,DIP2,ALB1,IMP3,KLMA_60069,BRX1,URB1,DHR2,FUN12,NOP15,KRE33,FCF2,KLMA_60313,NOP4,RSA4,NEW1,NOP14,NOC4,RRP9,MAK21,NOP9,LTV1,RLP24,RRP42,UTP25,NOP2,PUF6,RPL8B
GO:0006364	rRNA processing	183	74	18.41	1.40E-29	SPB1,UTP5,EBP2,RPF2,HAS1,NOG1,UTP13,DBP7,UTP15,UTP11,NOP53,NOP58,NOP12,ENP1,CBF5,RLP7,UTP8,MPP10,IPI3,NAN1,MRT4,RRS1,IPI1,DCAF13,DRS1,NOB1,RPL3,BFR2,PWP2,DBP3,RRP3,ECM16,MDN1,UTP10,GAR1,CIC1,ERB1,RRP12,IMP4,YTM1,NOP19,RIX1,UTP21,BMS1,UTP6,PRP43,GEP3,MTR4,UTP18,ENP2,RRP5,PWP1,NOP56,UTP4,NUG1,NOP7,DIP2,IMP3,BRX1,URB1,DHR2,FUN12,NOP15,KRE33,FCF2,KLMA_60313,NOP4,NOP14,NOC4,RRP9,NOP9,RRP42,UTP25,NOP2
GO:0022613	ribonucleoprotein complex biogenesis	319	99	32.1	7.40E-29	SPB1,UTP5,SDA1,EBP2,RPF2,RSE1,HAS1,NOG1,UTP13,DBP7,UTP15,UTP11,ARX1,NOP53,RPS2,NOP58,NOP12,ENP1,CBF5,RLP7,UTP8,MPP10,IPI3,RPS3,RPL5,NAN1,MRT4,RRS1,IPI1,DCAF13,DRS1,NOB1,RPL3,RLI1,BFR2,MEX67,PWP2,DBP3,RRP3,TIF32,RRB1,ECM16,MDN1,UTP10,GAR1,CIC1,ERB1,RRP12,IMP4,YTM1,NOP19,NMD3,RIX1,UTP21,MTR2,BMS1,UTP6,PRP43,NOC2,GEP3,MTR4,UTP18,ENP2,RRP5,PWP1,NOP56,UTP4,NUG1,NOP7,DIP2,ALB1,IMP3,KLMA_60069,BRX1,URB1,DHR2,FUN12,NOP15,KRE33,FCF2,KLMA_60313,NOP4,RSA4,NEW1,NOP14,TIF3,NOC4,RRP9,MAK21,NOP9,LTV1,RLP24,RRP42,PUB1,UTP25,NOP2,NIP1,PUF6,RPL8B

GO.ID	Term	Annotated gene	Significant	Expected	P-value	Genes
GO:0016072	rRNA metabolic process	207	77	20.83	6.50E-28	SPB1,RPA49,UTP5,EBP2,RPF2,HAS1,NOG1,UTP13,DBP7,UTP15,UTP11,NOP53,NOP58,NOP12,ENP1,CBF5,RLP7,UTP8,MPP10,IPI3,NAN1,MRT4,RRS1,IPI1,DCAF13,DRS1,NOB1,RPL3,BFR2,PWP2,DBP3,RRP3,ECM16,MDN1,UTP10,GAR1,CIC1,ERB1,RRP12,IMP4,YTM1,NOP19,RIX1,UTP21,BMS1,UTP6,PRP43,GEP3,MTR4,UTP18,ENP2,RPA135,RRP5,PWP1,NOP56,UTP4,NUG1,NOP7,DIP2,IMP3,BRX1,URB1,DHR2,RPA190,FUN12,NOP15,KRE33,FCF2,KLMA_60313,NOP4,NOP14,NOC4,RRP9,NOP9,RRP42,UTP25,NOP2
GO:0034470	ncRNA processing	254	78	25.56	5.10E-22	SPB1,NOP1,UTP5,EBP2,RPF2,HAS1,NOG1,UTP13,DBP7,UTP15,UTP11,NOP53,NOP58,NOP12,ENP1,CBF5,RLP7,UTP8,MPP10,IPI3,NAN1,MRT4,RRS1,IPI1,DCAF13,DRS1,NOB1,RPL3,BFR2,PWP2,DBP3,RRP3,MTO1,ECM16,MDN1,UTP10,GAR1,CIC1,ERB1,RRP12,IMP4,YTM1,NOP19,RIX1,UTP21,PUS1,BMS1,UTP6,PRP43,GEP3,MTR4,UTP18,ENP2,RRP5,PWP1,NOP56,UTP4,NUG1,NOP7,DIP2,IMP3,BRX1,URB1,DHR2,FUN12,NOP15,KRE33,FCF2,KLMA_60313,NOP4,IKI3,NOP14,NOC4,RRP9,NOP9,RRP42,UTP25,NOP2
GO:0034660	ncRNA metabolic process	339	91	34.11	3.30E-21	SPB1,RPA49,NOP1,UTP5,EBP2,RPF2,MES1,HAS1,MSW1,NOG1,UTP13,RPC37,DBP7,UTP15,UTP11,NOP53,NOP58,NOP12,ENP1,CBF5,RLP7,UTP8,MPP10,IPI3,NAN1,MRT4,RRS1,IPI1,DCAF13,DRS1,NOB1,RPL3,BFR2,PWP2,DBP3,RRP3,MTO1,GUS1,ECM16,MDN1,UTP10,GAR1,CIC1,ERB1,RRP12,IMP4,YTM1,NOP19,RPC82,RIX1,UTP21,PUS1,BMS1,UTP6,PRP43,GEP3,MTR4,UTP18,ENP2,RPA135,RRP5,PWP1,NOP56,UTP4,NUG1,RPC40,VAS1,NOP7,DIP2,IMP3,BRX1,URB1,DHR2,RPA190,FUN12,NOP15,KRE33,FCF2,KLMA_60313,NOP4,JHD2,CDC60,IKI3,NOP14,NOC4,RRP9,NOP9,RRP42,UTP25,NOP2,ILS1
GO:0042274	ribosomal small subunit biogenesis	94	44	9.46	1.20E-20	UTP5,HAS1,UTP13,UTP15,UTP11,NOP58,ENP1,UTP8,MPP10,NAN1,RRS1,DCAF13,NOB1,BFR2,PWP2,RRP3,ECM16,UTP10,RRP12,IMP4,NOP19,BMS1,UTP6,PRP43,GEP3,UTP18,ENP2,RRP5,UTP4,NOP7,DIP2,IMP3,KLMA_60069,DHR2,FUN12,KRE33,FCF2,KLMA_60313,NEW1,NOP14,NOC4,NOP9,LTV1,UTP25

GO.ID	Term	Annotated gene	Significant	Expected	P-value	Genes
GO:0000462	maturation of SSU-rRNA from tricistronic...	72	37	7.24	3.20E-19	UTP5,HAS1,UTP13,UTP15,UTP11,NOP58,ENP1,UTP8,MPP10,NAN1,RRS1,DCAF13,NOB1,BFR2,PWP2,RRP3,ECM16,UTP10,RRP12,NOP19,BMS1,UTP6,PRP43,UTP18,ENP2,RRP5,UTP4,NOP7,DIP2,DHR2,FUN12,FCF2,KLMA_60313,NOP14,NOC4,NOP9,UTP25
GO:0042273	ribosomal large subunit biogenesis	76	38	7.65	3.50E-19	SPB1,SDA1,RPF2,HAS1,NOG1,DBP7,NOP53,NOP12,RLP7,IPI3,RPL5,MRT4,RRS1,IPI1,DRS1,RPL3,RLI1,DBP3,MDN1,CIC1,ERB1,YTM1,RIX1,PRP43,NOC2,RRP5,NUG1,NOP7,ALB1,BRX1,URB1,NOP15,NOP4,RSA4,MAK21,RLP24,NOP2,PUF6
GO:0030490	maturation of SSU-rRNA	80	38	8.05	3.40E-18	UTP5,HAS1,UTP13,UTP15,UTP11,NOP58,ENP1,UTP8,MPP10,NAN1,RRS1,DCAF13,NOB1,BFR2,PWP2,RRP3,ECM16,UTP10,RRP12,NOP19,BMS1,UTP6,PRP43,GEP3,UTP18,ENP2,RRP5,UTP4,NOP7,DIP2,DHR2,FUN12,FCF2,KLMA_60313,NOP14,NOC4,NOP9,UTP25
GO:0006396	RNA processing	366	81	36.82	2.20E-13	SPB1,NOP1,UTP5,EBP2,RPF2,RSE1,HAS1,NOG1,UTP13,DBP7,UTP15,UTP11,NOP53,NOP58,NOP12,ENP1,CBF5,RLP7,UTP8,MPP10,IPI3,NAN1,MRT4,RRS1,IPI1,DCAF13,DRS1,NOB1,RPL3,MRPL15,BFR2,PWP2,DBP3,RRP3,MTO1,ECM16,MDN1,UTP10,GAR1,CIC1,ERB1,RRP12,IMP4,YTM1,NOP19,RIX1,UTP21,PUS1,BMS1,UTP6,PRP43,GEP3,MTR4,UTP18,ENP2,RRP5,PWP1,NOP56,UTP4,NUG1,NOP7,DIP2,IMP3,BRX1,URB1,DHR2,FUN12,NOP15,KRE33,FCF2,KLMA_60313,NOP4,IKI3,NOP14,NOC4,RRP9,PUS4,NOP9,RRP42,UTP25,NOP2
GO:0000463	maturation of LSU-rRNA from tricistronic...	35	21	3.52	3.90E-13	SPB1,RPF2,HAS1,DBP7,NOP53,NOP12,RLP7,IPI1,DBP3,CIC1,ERB1,YTM1,PRP43,RRP5,NUG1,NOP7,BRX1,URB1,NOP15,NOP4,NOP2
GO:0000460	maturation of 5.8S rRNA	73	30	7.34	1.70E-12	SPB1,RPF2,UTP13,UTP11,NOP58,ENP1,RLP7,MPP10,RRS1,PWP2,DBP3,UTP10,CIC1,ERB1,YTM1,NOP19,UTP6,PRP43,MTR4,UTP18,RRP5,NOP7,DIP2,BRX1,URB1,FCF2,NOP14,NOC4,NOP9,RRP42
GO:0000466	maturation of 5.8S rRNA from tricistroni...	73	30	7.34	1.70E-12	SPB1,RPF2,UTP13,UTP11,NOP58,ENP1,RLP7,MPP10,RRS1,PWP2,DBP3,UTP10,CIC1,ERB1,YTM1,NOP19,UTP6,PRP43,MTR4,UTP18,RRP5,NOP7,DIP2,BRX1,URB1,FCF2,NOP14,NOC4,NOP9,RRP42

GO.ID	Term	Annotated gene	Significant	Expected	P-value	Genes
GO:0000470	maturation of LSU-rRNA	37	21	3.72	1.80E-12	SPB1,RPF2,HAS1,DBP7,NOP53,NOP12,RLP7,IPI1,DBP3,CIC1,ERB1,YTM1,PRP43,RRP5,NUG1,NOP7,BRX1,URB1,NOP15,NOP4,NOP2
GO:0009185	ribonucleoside diphosphate metabolic pro...	23	16	2.31	1.00E-11	GPM3,ENO,PGK,RAG2,GPM1,ADK1,RNR2,PDC2,TPI1,GAP1,FBA1,GUK1,PYK1,RAG5,RNR1,GAP3
GO:0000967	rRNA 5'-end processing	31	18	3.12	4.40E-11	UTP13,UTP11,NOP58,RLP7,MPP10,PWP2,UTP10,NOP19,BMS1,UTP6,UTP18,RRP5,DIP2,BRX1,FCF2,NOP14,NOC4,NOP9
GO:0009132	nucleoside diphosphate metabolic process	25	16	2.52	6.90E-11	GPM3,ENO,PGK,RAG2,GPM1,ADK1,RNR2,PDC2,TPI1,GAP1,FBA1,GUK1,PYK1,RAG5,RNR1,GAP3
GO:0044283	small molecule biosynthetic process	254	58	25.56	3.30E-10	HIS4,GPM3,OLE1,HPT1,GUA1,BIO2,ENO,FAU1,PGK,SCS7,RAG2,URA1,GPM1,ADH4,MET13,CYS4,SHM2,ERG3,ERG28,CYP707A7,ORT1,ERG9,HAM1,PHS1,ERG1,MEU1,PDC2,SER3,PAN5,ERG6,dsd1,TPI1,GAP1,RIB7,FMS1,FBA1,ALD5,MET5,FAS1,GUK1,SHM1,LEU1,PDX3,SUR4,ERG25,PDC1,PYK1,LEU4,RAG5,cyp524A1,LEU3,ERG13,URA7,FAS2,GAP3,MET3,VID24,MET6
GO:0000966	RNA 5'-end processing	34	18	3.42	3.50E-10	UTP13,UTP11,NOP58,RLP7,MPP10,PWP2,UTP10,NOP19,BMS1,UTP6,UTP18,RRP5,DIP2,BRX1,FCF2,NOP14,NOC4,NOP9
GO:0034471	ncRNA 5'-end processing	34	18	3.42	3.50E-10	UTP13,UTP11,NOP58,RLP7,MPP10,PWP2,UTP10,NOP19,BMS1,UTP6,UTP18,RRP5,DIP2,BRX1,FCF2,NOP14,NOC4,NOP9
GO:0000478	endonucleolytic cleavage involved in rRN...	42	20	4.23	4.20E-10	UTP13,UTP11,NOP58,ENP1,MPP10,RRS1,PWP2,DBP3,UTP10,NOP19,BMS1,UTP6,UTP18,RRP5,DIP2,BRX1,FCF2,NOP14,NOC4,NOP9
GO:0000479	endonucleolytic cleavage of tricistronic...	42	20	4.23	4.20E-10	UTP13,UTP11,NOP58,ENP1,MPP10,RRS1,PWP2,DBP3,UTP10,NOP19,BMS1,UTP6,UTP18,RRP5,DIP2,BRX1,FCF2,NOP14,NOC4,NOP9
GO:0090502	RNA phosphodiester bond hydrolysis, endo...	42	20	4.23	4.20E-10	UTP13,UTP11,NOP58,ENP1,MPP10,RRS1,PWP2,DBP3,UTP10,NOP19,BMS1,UTP6,UTP18,RRP5,DIP2,BRX1,FCF2,NOP14,NOC4,NOP9

GO.ID	Term	Annotated gene	Significant	Expected	P-value	Genes
GO:0009135	purine nucleoside diphosphate metabolic ...	21	14	2.11	5.10E-10	GPM3,ENO,PGK,RAG2,GPM1,ADK1,PDC2,TPI1,GAP1,FBA1,GUK1,PYK1,RAG5,GAP3
GO:0009179	purine ribonucleoside diphosphate metabo...	21	14	2.11	5.10E-10	GPM3,ENO,PGK,RAG2,GPM1,ADK1,PDC2,TPI1,GAP1,FBA1,GUK1,PYK1,RAG5,GAP3
GO:0000469	cleavage involved in rRNA processing	61	24	6.14	9.50E-10	UTP13,UTP11,NOP58,ENP1,RLP7,MPP10,RRS1,NOB1,PWP2,DBP3,UTP10,NOP19,BMS1,UTP6,MTR4,UTP18,RRP5,DIP2,BRX1,FCF2,NOP14,NOC4,NOP9,RRP42
GO:0034404	nucleobase-containing small molecule bio...	36	18	3.62	1.20E-09	GPM3,HPT1,GUA1,ENO,PGK,RAG2,URA1,GPM1,HAM1,MEU1,TPI1,GAP1,FBA1,GUK1,PYK1,RAG5,URA7,GAP3
GO:0000472	endonucleolytic cleavage to generate mat...	29	16	2.92	1.60E-09	UTP13,UTP11,NOP58,MPP10,PWP2,UTP10,NOP19,BMS1,UTP6,UTP18,RRP5,DIP2,FCF2,NOP14,NOC4,NOP9
GO:0000480	endonucleolytic cleavage in 5'-ETS of tr...	30	16	3.02	3.00E-09	UTP13,UTP11,NOP58,MPP10,PWP2,UTP10,NOP19,BMS1,UTP6,UTP18,RRP5,DIP2,FCF2,NOP14,NOC4,NOP9
GO:0046031	ADP metabolic process	20	13	2.01	3.50E-09	GPM3,ENO,PGK,RAG2,GPM1,ADK1,PDC2,TPI1,GAP1,FBA1,PYK1,RAG5,GAP3
GO:0034641	cellular nitrogen compound metabolic pro...	1503	201	151.22	4.60E-09	SPB1,APA2,HIS4,RpL37a,RPA49,PRS5,GPM3,NOP1,SOH1,DSS1,UTP5,HPT1,GUA1,BIO2,RPL17B,EBP2,RPF2,RSE1,GAL80,ENO,MES1,HAS1,YTA7,LIP1,FAU1,PGK,MSW1,FUR1,AAH1,RAG2,NOG1,URA1,GPM1,UTP13,RPC37,DBP7,UTP15,ADH4,UTP11,ADK1,NOP53,RPS2,MET13,SHM2,NOP58,FHL1,KLMA_20355,NOP12,ENP1,CBF5,KLMA_20481,RLP7,RPL22A,UTP8,TPA1,ADE2,MPP10,IPI3,RPS3,RPL5,NAN1,HAM1,MRT4,RRS1,GSH1,IPI1,RNR2,DCAF13,MEU1,DRS1,ETT1,NOB1,RPL3,PAB1,RLI1,MRPL15,PDC2,BFR2,PWP2,YIH1,DBP3,PAN5,RRP3,TIF32,HAL9,VTS1,RAD54,KSS1,SAH1,DUG1,ADE5,7,MTO1,GUS1,ECM16,RPL15B,dsd1,HEM13,TPI1,MDN1,GAP1,RIB7,UTP10,GAR1,CIC1,HAP1

GO.ID	Term	Annotated gene	Significant	Expected	P-value	Genes
						,ERB1,MCM1,IMP2,RRP12,IMP4,FMS1,FBA1,MAM33,YTM1,NOP19,HMG1,RPC82,RIX1,XPT1,UTP21,PUS1,BMS1,RPL10A,GUK1,UTP6,PRP43,GEP3,SHM1,MTR4,UTP18,ENP2,RPA135,PDX3,RPS14,ADE6,RRP5,PWP1,NOP56,MSS51,LAC9,UTP4,NUG1,RPC40,VAS1,NOP7,RPL2,DIP2,ACS2,URA2,IMP3,KLMA_60069,PDC1,EXO1,BRX1,URB1,DHR2,RPA190,FUN12,REV1,PYK1,YAF9,NOP15,KRE33,EPL1,FCF2,KLMA_60313,UGP1,NOP4,RAG5,JHD2,LEU3,ERG13,RPB1,CDC60,PET309,IKI3,URA7,ROX3,RNR1,SPT21,NOP14,TIF3,MET16,NOC4,RRP9,HEM14,RPL19B,PUS4,KLMA_70408,NOP9,GAP3,RRP42,PUB1,UTP25,HDA1,NOP2,NIP1,KLMA_80256,DEF1,MET6,ILS1
GO:0000447	endonucleolytic cleavage in ITS1 to sepa...	35	17	3.52	6.20E-09	UTP13,UTP11,NOP58,ENP1,MPP10,RRS1,PWP2,UTP10,NOP19,UTP6,UTP18,RRP5,DIP2,FCF2,NOP14,NOC4,NOP9
GO:0000027	ribosomal large subunit assembly	24	14	2.41	6.40E-09	RPF2,NOP53,IPI3,RPL5,MRT4,IPI1,DRS1,RPL3,MDN1,RIX1,RRP5,BRX1,RSA4,MAK21
GO:0071704	organic substance metabolic process	2233	267	224.67	2.60E-08	SPB1,APA2,HIS4,LPP1,SAM2,RpL37a,RPA49,ACO2,PRS5,GPD1,GPM3,NOP1,OLE1,SOH1,DSS1,UTP5,HPT1,GUA1,PFK1,BIO2,RPL17B,EBP2,RPF2,RSE1,GAL80,ENO,MES1,HAS1,TDA1,YTA7,LIP1,FAU1,PGK,CWH43,SCS7,MSW1,FUR1,AAH1,RAG2,NOG1,URA1,TPS2,KLMA_20052,TSL1,GPM1,UTP13,RPC37,DBP7,UTP15,HSL1,ADH4,UTP11,ADK1,NOP53,RPS2,MET13,CYS4,SHM2,ERG3,NOP58,GAL7,GAL10,GAL1,FHL1,KLMA_20355,NOP12,KLMA_20392,ENP1,CLN2,UBP3,CBF5,KLMA_20481,RLP7,RPL22A,UTP8,TPA1,ERG28,CYP707A7,ORT1,ADE2,MPP10,ERG9,DTD1,IPI3,RPS3,RPL5,NAN1,LIA1,HAM1,MRT4,RRS1,PGM2,GSH1,PHS1,IPI1,RNR2,ERG1,DCAF13,MEU1,DRS1,ETT1,NOB1,RPL3,PAB1,RLI1,MRPL15,KLMA_30320,GSY2,PDC2,SUR2,BFR2,MEX67,SER3,PWP2,YIH1,DBP3,PAN5,RRP3,TIF32,HAL9,VTS1,SCW4,RAD54,KSS1,ERG6,SAH1,DUG1,ADE5,7,MTO1,GUS1,ECM16,RPL15B,dsd1,HEM13,TPI1,MDN1,GAP1,RIB7,UTP10,GAR1,CIC1,HAP1,ERB1,MCM1,IMP2,RRP12,IMP4,FMS1,FBA1,ALD5,MAM33,YTM1,NOP19,HMG1,DPM1,RPC82,MET5,RIX1,XPT1,UTP21,FAS1,MTR2,PUS1,BMS1,RPL10A,GUK1,UTP6,PRP43,GEP3,SHM1,MTR4,UTP18,ENP2,LEU1,RPA135,YJU3,PDX3,RPS14,SUR4,ADE6,ERG25,RRP5,PWP1,NOP56,MSS51,LAC9,UTP4,NUG1,

GO.ID	Term	Annotated gene	Significant	Expected	P-value	Genes
						RPC40,VAS1,NOP7,RPL2,DIP2,ACS2,MAP1,SHB17,URA2,IMP3,KLMA_60069,PDC1,EXO1,MDM20,BRX1,URB1,MDH1,DHR2,RPA190,FUN12,REV1,PYK1,LEU4,YAF9,NOP15,KRE33,EPL1,FCF2,KLMA_60313,RGT1,UGP1,NOP4,RAG5,cyp524A1,JHD2,LEU3,ERG13,PFK2,RPB1,CDC60,PET309,IKI3,URA7,ROX3,RNR1,PCL6,HSL7,FAS2,SPT21,NEW1,NOP14,TIF3,MET16,NOC4,RRP9,HEM14,RPL19B,DYS1,PUS4,KLMA_70408,NOP9,GAP3,MET3,MNN1,RRP42,PUB1,UTP25,HDA1,NOP2,BGL2,NIP1,KLMA_80256,DEF1,VID24,TPS1,PTH2,MET6,ILS1
GO:1901360	organic cyclic compound metabolic proces...	1310	178	131.8	4.00E-08	SPB1,APA2,HIS4,RPA49,PRS5,GPM3,NOP1,SOH1,DSS1,UTP5,HPT1,GUA1,BIO2,EBP2,RPF2,RSE1,GAL80,ENO,MES1,HAS1,YTA7,FAU1,PGK,MSW1,FUR1,AAH1,RAG2,NOG1,URA1,GPM1,UTP13,RPC37,DBP7,UTP15,ADH4,UTP11,ADK1,NOP53,MET13,SHM2,ERG3,NOP58,FHL1,NOP12,ENP1,CBF5,KLMA_20481,RLP7,UTP8,TPA1,ERG28,CYP707A7,ADE2,MPP10,ERG9,DTD1,IPI3,NAN1,HAM1,MRT4,RRS1,IPI1,RNR2,ERG1,DCAF13,MEU1,DRS1,NOB1,RPL3,PAB1,MRPL15,PDC2,BFR2,PWP2,DBP3,RRP3,HAL9,VTS1,RAD54,KSS1,ERG6,SAH1,ADE5,7,MTO1,GUS1,ECM16,HEM13,TPI1,MDN1,GAP1,RIB7,UTP10,GAR1,CIC1,HAP1,ERB1,MCM1,RRP12,IMP4,FBA1,YTM1,NOP19,HMG1,RPC82,RIX1,XPT1,UTP21,PUS1,BMS1,GUK1,UTP6,PRP43,GEP3,SHM1,MTR4,UTP18,ENP2,RPA135,PDX3,ADE6,ERG25,RRP5,PWP1,NOP56,LAC9,UTP4,NUG1,RPC40,VAS1,NOP7,DIP2,ACS2,URA2,IMP3,PDC1,EXO1,BRX1,URB1,DHR2,RPA190,FUN12,REV1,PYK1,YAF9,NOP15,KRE33,EPL1,FCF2,KLMA_60313,UGP1,NOP4,RAG5,cyp524A1,JHD2,ERG13,RPB1,CDC60,IKI3,URA7,ROX3,RNR1,SPT21,NOP14,NOC4,RRP9,HEM14,PUS4,KLMA_70408,NOP9,GAP3,RRP42,PUB1,UTP25,HDA1,NOP2,DEF1,MET6,ILS1
GO:0046390	ribose phosphate biosynthetic process	72	24	7.24	4.50E-08	PRS5,GPM3,HPT1,GUA1,ENO,PGK,RAG2,URA1,GPM1,ADK1,ADE2,ADE5,7,TPI1,GAP1,FBA1,XPT1,GUK1,ADE6,ACS2,SHB17,PYK1,RAG5,URA7,GAP3
GO:0006096	glycolytic process	17	11	1.71	6.50E-08	GPM3,ENO,PGK,RAG2,GPM1,TPI1,GAP1,FBA1,PYK1,RAG5,GAP3

GO.ID	Term	Annotated gene	Significant	Expected	P-value	Genes
GO:0006757	ATP generation from ADP	17	11	1.71	6.50E-08	GPM3,ENO,PGK,RAG2,GPM1,TPI1,GAP1,FBA1,PYK1,RAG5,GAP3
GO:0042866	pyruvate biosynthetic process	17	11	1.71	6.50E-08	GPM3,ENO,PGK,RAG2,GPM1,TPI1,GAP1,FBA1,PYK1,RAG5,GAP3
GO:0042255	ribosome assembly	36	16	3.62	8.70E-08	RPF2,NOP53,IPI3,RPL5,MRT4,IPI1,DRS1,RPL3,MDN1,RIX1,RRP5,KLMA_60069,BRX1,FUN12,RSA4,MAK21
GO:0009156	ribonucleoside monophosphate biosyntheti...	59	21	5.94	8.90E-08	PRS5,GPM3,HPT1,GUA1,ENO,PGK,AAH1,RAG2,URA1,GPM1,ADK1,ADE2,ADE5,7,TPI1,GAP1,FBA1,XPT1,ADE6,PYK1,RAG5,GAP3
GO:0072330	monocarboxylic acid biosynthetic process	59	21	5.94	8.90E-08	GPM3,OLE1,BIO2,ENO,PGK,SCS7,RAG2,GPM1,PHS1,PAN5,TPI1,GAP1,FMS1,FBA1,ALD5,FAS1,SUR4,PYK1,RAG5,FAS2,GAP3
GO:0009127	purine nucleoside monophosphate biosynth...	50	19	5.03	1.10E-07	GPM3,HPT1,GUA1,ENO,PGK,AAH1,RAG2,GPM1,ADK1,ADE2,ADE5,7,TPI1,GAP1,FBA1,XPT1,ADE6,PYK1,RAG5,GAP3
GO:0009168	purine ribonucleoside monophosphate bios...	50	19	5.03	1.10E-07	GPM3,HPT1,GUA1,ENO,PGK,AAH1,RAG2,GPM1,ADK1,ADE2,ADE5,7,TPI1,GAP1,FBA1,XPT1,ADE6,PYK1,RAG5,GAP3
GO:0006165	nucleoside diphosphate phosphorylation	18	11	1.81	1.50E-07	GPM3,ENO,PGK,RAG2,GPM1,TPI1,GAP1,FBA1,PYK1,RAG5,GAP3
GO:0046939	nucleotide phosphorylation	18	11	1.81	1.50E-07	GPM3,ENO,PGK,RAG2,GPM1,TPI1,GAP1,FBA1,PYK1,RAG5,GAP3
GO:0009124	nucleoside monophosphate biosynthetic pr...	61	21	6.14	1.70E-07	PRS5,GPM3,HPT1,GUA1,ENO,PGK,AAH1,RAG2,URA1,GPM1,ADK1,ADE2,ADE5,7,TPI1,GAP1,FBA1,XPT1,ADE6,PYK1,RAG5,GAP3
GO:0008152	metabolic process	2320	272	233.42	1.80E-07	SPB1,APA2,HIS4,LPP1,SAM2,RpL37a,RPA49,ACO2,PRS5,GPD1,GPM3,NOP1,OLE1,SOH1,DSS1,UTP5,HPT1,GUA1,PFK1,BIO2,R

GO.ID	Term	Annotated gene	Significant	Expected	P-value	Genes
						PL17B,EBP2,RPF2,RSE1,GAL80,ENO,MES1,HAS1,TDA1,YTA7,LIP1,FAU1,PGK,CWH43,SCS7,MSW1,FUR1,AAH1,RAG2,NOG1,URA1,TPS2,KLMA_20052,TSL1,GPM1,UTP13,RPC37,DBP7,UTP15,HSL1,ADH4,UTP11,ADK1,NOP53,RPS2,MET13,CYS4,SHM2,ERG3,NOP58,GAL7,GAL10,GAL1,FHL1,KLMA_20355,NOP12,KLMA_20392,ENP1,CLN2,UBP3,CBF5,KLMA_20481,RLP7,RPL22A,UTP8,TPA1,ERG28,CYP707A7,ORT1,ADE2,MPP10,ERG9,DTD1,IPI3,RPS3,RPL5,NAN1,LIA1,HAM1,MRT4,RRS1,PGM2,GSH1,PHS1,IPI1,RNR2,ERG1,DCAF13,MEU1,DRS1,ETT1,NOB1,RPL3,PAB1,RLI1,MRPL15,KLMA_30320,GSY2,PDC2,SUR2,BFR2,MEX67,SER3,PWP2,YIH1,DBP3,PAN5,RRP3,TIF32,HAL9,VTS1,SCW4,RAD54,KSS1,ERG6,SAH1,DUG1,ADE5,7,MTO1,GUS1,ECM16,RPL15B,dsd1,HEM13,TPI1,MDN1,GAP1,RIB7,UTP10,GAR1,CIC1,HAP1,ERB1,MCM1,IMP2,RRP12,IMP4,FMS1,FBA1,ALD5,MAM33,YTM1,NOP19,HMG1,DPM1,RPC82,MET5,RIX1,XPT1,UTP21,FAS1,MTR2,PUS1,BMS1,RPL10A,GUK1,UTP6,PRP43,GEP3,SHM1,MTR4,UTP18,ENP2,LEU1,RPA135,YJU3,PDX3,RPS14,SUR4,ADE6,ERG25,RRP5,PWP1,NOP56,MSS51,LAC9,UTP4,NUG1,RPC40,VAS1,NOP7,MET10,RPL2,DIP2,ACS2,MAP1,UTH1,SHB17,URA2,IMP3,KLMA_60069,PDC1,EXO1,IPP1,MDM20,BRX1,URB1,MDH1,DHR2,RPA190,FUN12,REV1,PYK1,LEU4,YAF9,NOP15,KRE33,EPL1,FCF2,KLMA_60313,RGT1,UGP1,NOP4,RAG5,cyp524A1,JHD2,LEU3,ERG13,PFK2,RPB1,CDC60,PET309,IKI3,URA7,ROX3,RNR1,PCL6,HSL7,FAS2,SPT21,NEW1,NOP14,TIF3,MET16,NOC4,RRP9,HEM14,RPL19B,DYS1,PUS4,PCL1,KLMA_70408,NOP9,GAP3,MET3,MNN1,RRP42,PUB1,UTP25,HDA1,NOP2,BGL2,NIP1,GDS1,KLMA_80256,DEF1,VID24,TPS1,PTH2,MET6,ILS1
GO:0044281	small molecule metabolic process	540	89	54.33	2.20E-07	APA2,HIS4,SAM2,ACO2,PRS5,GPM3,OLE1,HPT1,GUA1,BIO2,GAL80,ENO,MES1,FAU1,PGK,SCS7,MSW1,FUR1,AAH1,RAG2,URA1,GPM1,ADH4,ADK1,MET13,CYS4,SHM2,ERG3,GAL7,GAL10,GAL1,ERG28,CYP707A7,ORT1,ADE2,ERG9,DTD1,HAM1,PHS1,RNR2,ERG1,MEU1,PDC2,SER3,PAN5,ERG6,SAH1,ADE5,7,GUS1,dsd1,TPI1,GAP1,RIB7,FMS1,FBA1,ALD5,HMG1,MET5,XPT1,FAS1,GUK1,SHM1,LEU1,PDX3,SUR4,ADE6,ERG25,VAS1,ACS2,URA2,PDC1,MDH1,PYK1,LEU4,RGT1,UGP1,RAG5,cyp524A1,LEU3,ERG13,CDC60,URA7,RNR1,FAS2,GAP3,MET3,VID24,MET

GO.ID	Term	Annotated gene	Significant	Expected	P-value	Genes
						6,ILS1
GO:1901292	nucleoside phosphate catabolic process	22	12	2.21	2.30E-07	GPM3,ENO,PGK,RAG2,GPM1,HAM1,TPI1,GAP1,FBA1,PYK1,RAG5,GAP3
GO:0072522	purine-containing compound biosynthetic ...	73	23	7.34	2.80E-07	GPM3,HPT1,GUA1,ENO,PGK,AAH1,RAG2,GPM1,ADK1,ADE2,MEU1,ADE5,7,TPI1,GAP1,FBA1,XPT1,GUK1,ADE6,ACS2,PYK1,RAG5,GAP3,MET6
GO:0009260	ribonucleotide biosynthetic process	68	22	6.84	3.00E-07	GPM3,HPT1,GUA1,ENO,PGK,RAG2,URA1,GPM1,ADK1,ADE2,ADE5,7,TPI1,GAP1,FBA1,XPT1,GUK1,ADE6,ACS2,PYK1,RAG5,URA7,GAP3
GO:0090501	RNA phosphodiester bond hydrolysis	79	24	7.95	3.30E-07	UTP13,UTP11,NOP58,ENP1,RLP7,MPP10,RRS1,NOB1,PWP2,DBP3,UTP10,NOP19,BMS1,UTP6,MTR4,UTP18,RRP5,DIP2,BRX1,FCF2,NOP14,NOC4,NOP9,RRP42
GO:0016053	organic acid biosynthetic process	151	36	15.19	3.50E-07	HIS4,GPM3,OLE1,BIO2,ENO,FAU1,PGK,SCS7,RAG2,GPM1,MET13,CYS4,SHM2,ORT1,PHS1,MEU1,SER3,PAN5,TPI1,GAP1,FMS1,FBA1,ALD5,MET5,FAS1,SHM1,LEU1,SUR4,PYK1,LEU4,RAG5,LEU3,FAS2,GAP3,MET3,MET6
GO:0046394	carboxylic acid biosynthetic process	151	36	15.19	3.50E-07	HIS4,GPM3,OLE1,BIO2,ENO,FAU1,PGK,SCS7,RAG2,GPM1,MET13,CYS4,SHM2,ORT1,PHS1,MEU1,SER3,PAN5,TPI1,GAP1,FMS1,FBA1,ALD5,MET5,FAS1,SHM1,LEU1,SUR4,PYK1,LEU4,RAG5,LEU3,FAS2,GAP3,MET3,MET6

GO.ID	Term	Annotated gene	Significant	Expected	P-value	Genes
GO:0006725	cellular aromatic compound metabolic pro...	1260	168	126.77	7.70E-07	SPB1,APA2,HIS4,RPA49,PRS5,GPM3,NOP1,SOH1,DSS1,UTP5,HPT1,GUA1,EBP2,RPF2,RSE1,GAL80,ENO,MES1,HAS1,YTA7,FAU1,PGK,MSW1,FUR1,AAH1,RAG2,NOG1,URA1,GPM1,UTP13,RPC37,DBP7,UTP15,ADH4,UTP11,ADK1,NOP53,MET13,SHM2,NOP58,FHL1,NOP12,ENP1,CBF5,KLMA_20481,RLP7,UTP8,TPA1,ADE2,MPP10,DTD1,IPI3,NAN1,HAM1,MRT4,RRS1,IPI1,RNR2,DCAF13,MEU1,DRS1,NOB1,RPL3,PAB1,MRPL15,PDC2,BFR2,PWP2,DBP3,RRP3,HAL9,VTS1,RAD54,KSS1,SAH1,ADE5,7,MTO1,GUS1,ECM16,HEM13,TPI1,MDN1,GAP1,UTP10,GAR1,CIC1,HAP1,ERB1,MCM1,RRP12,IMP4,FBA1,YTM1,NOP19,HMG1,RPC82,RIX1,XPT1,UTP21,PUS1,BMS1,GUK1,UTP6,PRP43,GEP3,SHM1,MTR4,UTP18,ENP2,RPA135,PDX3,ADE6,RRP5,PWP1,NOP56,LAC9,UTP4,NUG1,RPC40,VAS1,NOP7,DIP2,ACS2,URA2,IMP3,PDC1,EXO1,BRX1,URB1,DHR2,RPA190,FUN12,REV1,PYK1,YAF9,NOP15,KRE33,EPL1,FCF2,KLMA_60313,UGP1,NOP4,RAG5,JHD2,ERG13,RPB1,CDC60,IKI3,URA7,ROX3,RNR1,SPT21,NOP14,NOC4,RRP9,HEM14,PUS4,KLMA_70408,NOP9,GAP3,RRP42,PUB1,UTP25,HDA1,NOP2,DEF1,MET6,ILS1
GO:0046483	heterocycle metabolic process	1270	169	127.78	7.90E-07	SPB1,APA2,HIS4,RPA49,PRS5,GPM3,NOP1,SOH1,DSS1,UTP5,HPT1,GUA1,BIO2,EBP2,RPF2,RSE1,GAL80,ENO,MES1,HAS1,YTA7,FAU1,PGK,MSW1,FUR1,AAH1,RAG2,NOG1,URA1,GPM1,UTP13,RPC37,DBP7,UTP15,ADH4,UTP11,ADK1,NOP53,MET13,SHM2,NOP58,FHL1,NOP12,ENP1,CBF5,KLMA_20481,RLP7,UTP8,TPA1,ADE2,MPP10,IPI3,NAN1,HAM1,MRT4,RRS1,IPI1,RNR2,DCAF13,MEU1,DRS1,NOB1,RPL3,PAB1,MRPL15,PDC2,BFR2,PWP2,DBP3,RRP3,HAL9,VTS1,RAD54,KSS1,SAH1,ADE5,7,MTO1,GUS1,ECM16,HEM13,TPI1,MDN1,GAP1,RIB7,UTP10,GAR1,CIC1,HAP1,ERB1,MCM1,RRP12,IMP4,FBA1,YTM1,NOP19,HMG1,RPC82,RIX1,XPT1,UTP21,PUS1,BMS1,GUK1,UTP6,PRP43,GEP3,SHM1,MTR4,UTP18,ENP2,RPA135,PDX3,ADE6,RRP5,PWP1,NOP56,LAC9,UTP4,NUG1,RPC40,VAS1,NOP7,DIP2,ACS2,URA2,IMP3,PDC1,EXO1,BRX1,URB1,DHR2,RPA190,FUN12,REV1,PYK1,YAF9,NOP15,KRE33,EPL1,FCF2,KLMA_60313,UGP1,NOP4,RAG5,JHD2,ERG13,RPB1,CDC60,IKI3,URA7,ROX3,RNR1,SPT21,NOP14,NOC4,RRP9,HEM14,PUS4,KLMA_70408,NOP9,GAP3,RRP42,PUB1,UTP25,HDA1,NOP2,DEF1,MET6,ILS1

GO.ID	Term	Annotated gene	Significant	Expected	P-value	Genes
GO:1901293	nucleoside phosphate biosynthetic proces...	94	26	9.46	8.20E-07	PRS5,GPM3,HPT1,GUA1,ENO,PGK,AAH1,RAG2,URA1,GPM1,ADK1,ADE2,RNR2,ADE5,7,TPI1,GAP1,FBA1,XPT1,GUK1,ADE6,ACS2,PYK1,RAG5,URA7,RNR1,GAP3
GO:1901566	organonitrogen compound biosynthetic pro...	639	99	64.29	8.40E-07	HIS4,RpL37a,GPM3,HPT1,GUA1,BIO2,RPL17B,ENO,MES1,LIP1,FAU1,PGK,CWH43,MSW1,FUR1,AAH1,RAG2,URA1,KLMA_20052,GPM1,UTP11,ADK1,RPS2,MET13,CYS4,SHM2,KLMA_20355,RPL22A,TPA1,ORT1,ADE2,RPS3,RPL5,GSH1,PHS1,RNR2,MEU1,ETT1,RPL3,PAB1,RLI1,KLMA_30320,PDC2,SUR2,SER3,YIH1,PAN5,TIF32,SAH1,ADE5,7,GUS1,RPL15B,dsd1,HEM13,TPI1,GAP1,RIB7,FMS1,FBA1,MAM33,DPM1,MET5,XPT1,RPL10A,GUK1,SHM1,LEU1,PDX3,RPS14,ADE6,MSS51,VAS1,RPL2,ACS2,URA2,KLMA_60069,FUN12,PYK1,LEU4,KLMA_60313,RAG5,LEU3,CDC60,PET309,IKI3,URA7,RNR1,TIF3,MET16,HEM14,RPL19B,GAP3,MET3,MNN1,NIP1,KLMA_80256,MET6,ILS1
GO:0006164	purine nucleotide biosynthetic process	62	20	6.24	1.10E-06	GPM3,HPT1,GUA1,ENO,PGK,RAG2,GPM1,ADK1,ADE2,ADE5,7,TPI1,GAP1,FBA1,XPT1,GUK1,ADE6,ACS2,PYK1,RAG5,GAP3
GO:0009152	purine ribonucleotide biosynthetic proce...	62	20	6.24	1.10E-06	GPM3,HPT1,GUA1,ENO,PGK,RAG2,GPM1,ADK1,ADE2,ADE5,7,TPI1,GAP1,FBA1,XPT1,GUK1,ADE6,ACS2,PYK1,RAG5,GAP3
GO:0009166	nucleotide catabolic process	21	11	2.11	1.30E-06	GPM3,ENO,PGK,RAG2,GPM1,TPI1,GAP1,FBA1,PYK1,RAG5,GAP3
GO:0046434	organophosphate catabolic process	29	13	2.92	1.30E-06	GPD1,GPM3,ENO,PGK,RAG2,GPM1,HAM1,TPI1,GAP1,FBA1,PYK1,RAG5,GAP3
GO:0006090	pyruvate metabolic process	25	12	2.52	1.40E-06	GPM3,ENO,PGK,RAG2,GPM1,PDC2,TPI1,GAP1,FBA1,PYK1,RAG5,GAP3

GO.ID	Term	Annotated gene	Significant	Expected	P-value	Genes
GO:0019752	carboxylic acid metabolic process	294	55	29.58	1.60E-06	HIS4,SAM2,ACO2,GPM3,OLE1,GUA1,BIO2,ENO,MES1,FAU1,PGK,SCS7,MSW1,RAG2,GPM1,ADH4,MET13,CYS4,SHM2,ORT1,DTD1,PHS1,MEU1,PDC2,SER3,PAN5,SAH1,GUS1,TPI1,GAP1,FMS1,FBA1,ALD5,MET5,FAS1,SHM1,LEU1,SUR4,ADE6,VAS1,ACS2,URA2,PDC1,MDH1,PYK1,LEU4,RAG5,LEU3,CDC60,URA7,FAS2,GAP3,MET3,MET6,ILS1
GO:0044237	cellular metabolic process	2232	261	224.57	1.80E-06	SPB1,APA2,HIS4,LPP1,SAM2,RpL37a,RPA49,ACO2,PRS5,GPD1,GPM3,NOP1,OLE1,SOH1,DSS1,UTP5,HPT1,GUA1,PFK1,BIO2,RPL17B,EBP2,RPF2,RSE1,GAL80,ENO,MES1,HAS1,TDA1,YTA7,LIP1,FAU1,PGK,CWH43,SCS7,MSW1,FUR1,AAH1,RAG2,NOG1,URA1,TPS2,KLMA_20052,TSL1,GPM1,UTP13,RPC37,DBP7,UTP15,HSL1,ADH4,UTP11,ADK1,NOP53,RPS2,MET13,CYS4,SHM2,ERG3,NOP58,FHL1,KLMA_20355,NOP12,ENP1,CLN2,UBP3,CBF5,KLMA_20481,RLP7,RPL22A,UTP8,TPA1,ERG28,CYP707A7,ORT1,ADE2,MPP10,ERG9,DTD1,IPI3,RPS3,RPL5,NAN1,LIA1,HAM1,MRT4,RRS1,GSH1,PHS1,IPI1,RNR2,ERG1,DCAF13,MEU1,DRS1,ETT1,NOB1,RPL3,PAB1,RLI1,MRPL15,KLMA_30320,GSY2,PDC2,SUR2,BFR2,SER3,PWP2,YIH1,DBP3,PAN5,RRP3,TIF32,HAL9,VTS1,RAD54,KSS1,ERG6,SAH1,DUG1,ADE5,7,MTO1,GUS1,ECM16,RPL15B,dsd1,HEM13,TPI1,MDN1,GAP1,RIB7,UTP10,GAR1,CIC1,HAP1,ERB1,MCM1,IMP2,RRP12,IMP4,FMS1,FBA1,ALD5,MAM33,YTM1,NOP19,HMG1,DPM1,RPC82,MET5,RIX1,XPT1,UTP21,FAS1,PUS1,BMS1,RPL10A,GUK1,UTP6,PRP43,GEP3,SHM1,MTR4,UTP18,ENP2,LEU1,RPA135,YJU3,PDX3,RPS14,SUR4,ADE6,ERG25,RRP5,PWP1,NOP56,MSS51,LAC9,UTP4,NUG1,RPC40,VAS1,NOP7,MET10,RPL2,DIP2,ACS2,MAP1,UTH1,SHB17,URA2,IMP3,KLMA_60069,PDC1,EXO1,IPP1,MDM20,BRX1,URB1,MDH1,DHR2,RPA190,FUN12,REV1,PYK1,LEU4,YAF9,NOP15,KRE33,EPL1,FCF2,KLMA_60313,UGP1,NOP4,RAG5,cyp524A1,JHD2,LEU3,ERG13,PFK2,RPB1,CDC60,PET309,IKI3,URA7,ROX3,RNR1,PCL6,HSL7,FAS2,SPT21,NOP14,TIF3,MET16,NOC4,RRP9,HEM14,RPL19B,DYS1,PUS4,PCL1,KLMA_70408,NOP9,GAP3,MET3,MNN1,RRP42,PUB1,UTP25,HDA1,NOP2,NIP1,GDS1,KLMA_80256,DEF1,VID24,TPS1,PTH2,MET6,ILS1
GO:0009165	nucleotide biosynthetic process	92	25	9.26	1.90E-06	PRS5,GPM3,HPT1,GUA1,ENO,PGK,RAG2,URA1,GPM1,ADK1,ADE2,RNR2,ADE5,7,TPI1,GAP1,FBA1,XPT1,GUK1,ADE6,ACS2,PYK1,RAG5,URA7,RNR1,GAP3

GO.ID	Term	Annotated gene	Significant	Expected	P-value	Genes
GO:0019693	ribose phosphate metabolic process	117	29	11.77	2.30E-06	PRS5,GPM3,HPT1,GUA1,ENO,PGK,RAG2,URA1,GPM1,ADK1,ADE2,RNR2,PDC2,ADE5,7,TPI1,GAP1,FBA1,HMG1,XPT1,GUK1,ADE6,ACS2,SHB17,PYK1,RAG5,ERG13,URA7,RNR1,GAP3
GO:0009259	ribonucleotide metabolic process	105	27	10.56	2.40E-06	GPM3,HPT1,GUA1,ENO,PGK,RAG2,URA1,GPM1,ADK1,ADE2,RNR2,PDC2,ADE5,7,TPI1,GAP1,FBA1,HMG1,XPT1,GUK1,ADE6,ACS2,PYK1,RAG5,ERG13,URA7,RNR1,GAP3
GO:0044238	primary metabolic process	2108	249	212.09	2.90E-06	SPB1,APA2,HIS4,LPP1,SAM2,RpL37a,RPA49,ACO2,PRS5,GPD1,GPM3,NOP1,OLE1,SOH1,DSS1,UTP5,HPT1,GUA1,RPL17B,EBP2,RPF2,RSE1,GAL80,ENO,MES1,HAS1,TDA1,YTA7,LIP1,PGK,CWH43,SCS7,MSW1,FUR1,AAH1,RAG2,NOG1,URA1,TPS2,KLMA_20052,TSL1,GPM1,UTP13,RPC37,DBP7,UTP15,HSL1,ADH4,UTP11,ADK1,NOP53,RPS2,MET13,CYS4,SHM2,ERG3,NOP58,GAL7,GAL10,GAL1,FHL1,KLMA_20355,NOP12,KLMA_20392,ENP1,CLN2,UBP3,CBF5,KLMA_20481,RLP7,RPL22A,UTP8,TPA1,ERG28,CYP707A7,ORT1,ADE2,MPP10,ERG9,DTD1,IPI3,RPS3,RPL5,NAN1,LIA1,HAM1,MRT4,RRS1,PGM2,PHS1,IPI1,RNR2,ERG1,DCAF13,MEU1,DRS1,ETT1,NOB1,RPL3,PAB1,RLI1,MRPL15,KLMA_30320,GSY2,PDC2,SUR2,BFR2,SER3,PWP2,YIH1,DBP3,RRP3,TIF32,HAL9,VTS1,SCW4,RAD54,KSS1,ERG6,SAH1,ADE5,7,MTO1,GUS1,ECM16,RPL15B,dsd1,TPI1,MDN1,GAP1,UTP10,GAR1,CIC1,HAP1,ERB1,MCM1,IMP2,RRP12,IMP4,FBA1,MAM33,YTM1,NOP19,HMG1,DPM1,RPC82,MET5,RIX1,XPT1,UTP21,FAS1,PUS1,BMS1,RPL10A,GUK1,UTP6,PRP43,GEP3,SHM1,MTR4,UTP18,ENP2,LEU1,RPA135,YJU3,RPS14,SUR4,ADE6,ERG25,RRP5,PWP1,NOP56,MSS51,LAC9,UTP4,NUG1,RPC40,VAS1,NOP7,RPL2,DIP2,ACS2,MAP1,URA2,IMP3,KLMA_60069,PDC1,EXO1,MDM20,BRX1,URB1,MDH1,DHR2,RPA190,FUN12,REV1,PYK1,LEU4,YAF9,NOP15,KRE33,EPL1,FCF2,KLMA_60313,RGT1,UGP1,NOP4,RAG5,cyp524A1,JHD2,LEU3,ERG13,RPB1,CDC60,PET309,IKI3,URA7,ROX3,RNR1,PCL6,HSL7,FAS2,SPT21,NOP14,TIF3,NOC4,RRP9,RPL19B,DYS1,PUS4,KLMA_70408,NOP9,GAP3,MET3,MNN1,RRP42,PUB1,UTP25,HDA1,NOP2,BGL2,NIP1,KLMA_80256,DEF1,VID24,TPS1,PTH2,MET6,ILS1

GO.ID	Term	Annotated gene	Significant	Expected	P-value	Genes
GO:0044085	cellular component biogenesis	746	109	75.06	4.00E-06	SPB1,UTP5,SDA1,EBP2,RPF2,RSE1,HAS1,SCO1,NOG1,UTP13,DBP7,UTP15,UTP11,ARX1,NOP53,RPS2,NOP58,NOP12,ENP1,CBF5,RLP7,UTP8,MPP10,IPI3,RPS3,RPL5,NAN1,MRT4,RRS1,IPI1,DCAF13,DRS1,NOB1,RPL3,RLI1,BFR2,MEX67,PWP2,YIH1,DBP3,RRP3,TIF32,KSS1,RRB1,ECM16,MDN1,UTP10,GAR1,CIC1,SFB3,ERB1,RRP12,IMP4,YTM1,NOP19,NMD3,RIX1,UTP21,MTR2,BMS1,UTP6,PRP43,NOC2,GEP3,MTR4,UTP18,ENP2,CWP1,RRP5,PWP1,NOP56,MSS51,UTP4,NUG1,NOP7,DIP2,UTH1,ALB1,IMP3,KLMA_60069,BRX1,URB1,DHR2,FUN12,NOP15,KRE33,FCF2,KLMA_60313,NOP4,PFK2,PET309,RSA4,NEW1,NOP14,TIF3,NOC4,RRP9,MAK21,NOP9,LTV1,RLP24,RRP42,PUB1,UTP25,HDA1,NOP2,NIP1,PUF6,RPL8B
GO:0043436	oxoacid metabolic process	303	55	30.49	4.20E-06	HIS4,SAM2,ACO2,GPM3,OLE1,GUA1,BIO2,ENO,MES1,FAU1,PGK,SCS7,MSW1,RAG2,GPM1,ADH4,MET13,CYS4,SHM2,ORT1,DTD1,PHS1,MEU1,PDC2,SER3,PAN5,SAH1,GUS1,TPI1,GAP1,FMS1,FBA1,ALD5,MET5,FAS1,SHM1,LEU1,SUR4,ADE6,VAS1,ACS2,URA2,PDC1,MDH1,PYK1,LEU4,RAG5,LEU3,CDC60,URA7,FAS2,GAP3,MET3,MET6,ILS1
GO:0055086	nucleobase-containing small molecule met...	180	38	18.11	4.20E-06	APA2,PRS5,GPM3,HPT1,GUA1,ENO,PGK,FUR1,AAH1,RAG2,URA1,GPM1,ADH4,ADK1,ADE2,HAM1,RNR2,MEU1,PDC2,SAH1,ADE5,7,TPI1,GAP1,FBA1,HMG1,XPT1,GUK1,ADE6,ACS2,URA2,PYK1,UGP1,RAG5,ERG13,URA7,RNR1,GAP3,MET6
GO:0010467	gene expression	1083	146	108.96	4.70E-06	SPB1,RpL37a,RPA49,NOP1,SOH1,UTP5,RPL17B,EBP2,RPF2,RSE1,GAL80,MES1,HAS1,YTA7,MSW1,NOG1,UTP13,RPC37,DBP7,UTP15,UTP11,NOP53,RPS2,NOP58,FHL1,KLMA_20355,NOP12,ENP1,CBF5,KLMA_20481,RLP7,RPL22A,UTP8,TPA1,MPP10,IPI3,RPS3,RPL5,NAN1,LIA1,MRT4,RRS1,IPI1,DCAF13,DRS1,ETT1,NOB1,RPL3,PAB1,RLI1,MRPL15,PDC2,BFR2,MEX67,PWP2,YIH1,DBP3,RRP3,TIF32,HAL9,VTS1,KSS1,MTO1,GUS1,ECM16,RPL15B,MDN1,UTP10,GAR1,CIC1,HAP1,ERB1,MCM1,IMP2,RRP12,IMP4,MAM33,YTM1,NOP19,RPC82,RIX1,UTP21,MTR2,PUS1,BMS1,RPL10A,UTP6,PRP43,GEP3,MTR4,UTP18,ENP2,RPA135,RPS14,RRP5,PWP1,NOP56,MSS51,LAC9,UTP4,NUG1,RPC40,VAS1,NOP7,RPL2,DIP2,MAP1,IMP3,KLMA_60069,MDM20,BRX1,URB1,DHR2,RPA190,FUN12,YAF9,NOP15,KRE33,EPL1,FCF2,KLMA

GO.ID	Term	Annotated gene	Significant	Expected	P-value	Genes
						_60313,NOP4,JHD2,CDC60,PET309,IKI3,ROX3,SPT21,NEW1,NOP14,TIF3,NOC4,RRP9,RPL19B,DYS1,PUS4,KLMA_70408,NOP9,RRP42,PUB1,UTP25,HDA1,NOP2,NIP1,KLMA_80256,ILS1
GO:0006139	nucleobase-containing compound metabolic...	1196	158	120.33	4.80E-06	SPB1,APA2,RPA49,PRS5,GPM3,NOP1,SOH1,DSS1,UTP5,HPT1,GUA1,EBP2,RPF2,RSE1,GAL80,ENO,MES1,HAS1,YTA7,PGK,MSW1,FUR1,AAH1,RAG2,NOG1,URA1,GPM1,UTP13,RPC37,DBP7,UTP15,ADH4,UTP11,ADK1,NOP53,NOP58,FHL1,NOP12,ENP1,CBF5,KLMA_20481,RLP7,UTP8,TPA1,ADE2,MPP10,IPI3,NAN1,HAM1,MRT4,RRS1,IPI1,RNR2,DCAF13,MEU1,DRS1,NOB1,RPL3,PAB1,MRPL15,PDC2,BFR2,PWP2,DBP3,RRP3,HAL9,VTS1,RAD54,KSS1,SAH1,ADE5,7,MTO1,GUS1,ECM16,TPI1,MDN1,GAP1,UTP10,GAR1,CIC1,HAP1,ERB1,MCM1,RRP12,IMP4,FBA1,YTM1,NOP19,HMG1,RPC82,RIX1,XPT1,UTP21,PUS1,BMS1,GUK1,UTP6,PRP43,GEP3,MTR4,UTP18,ENP2,RPA135,ADE6,RRP5,PWP1,NOP56,LAC9,UTP4,NUG1,RPC40,VAS1,NOP7,DIP2,ACS2,URA2,IMP3,EXO1,BRX1,URB1,DHR2,RPA190,FUN12,REV1,PYK1,YAF9,NOP15,KRE33,EPL1,FCF2,KLMA_60313,UGP1,NOP4,RAG5,JHD2,ERG13,RPB1,CDC60,IKI3,URA7,ROX3,RNR1,SPT21,NOP14,NOC4,RRP9,PUS4,KLMA_70408,NOP9,GAP3,RRP42,PUB1,UTP25,HDA1,NOP2,DEF1,MET6,ILS1
GO:0006082	organic acid metabolic process	305	55	30.69	5.20E-06	HIS4,SAM2,ACO2,GPM3,OLE1,GUA1,BIO2,ENO,MES1,FAU1,PGK,SCS7,MSW1,RAG2,GPM1,ADH4,MET13,CYS4,SHM2,ORT1,DTD1,PHS1,MEU1,PDC2,SER3,PAN5,SAH1,GUS1,TPI1,GAP1,FMS1,FBA1,ALD5,MET5,FAS1,SHM1,LEU1,SUR4,ADE6,VAS1,ACS2,URA2,PDC1,MDH1,PYK1,LEU4,RAG5,LEU3,CDC60,URA7,FAS2,GAP3,MET3,MET6,ILS1
GO:0016052	carbohydrate catabolic process	38	14	3.82	8.50E-06	GPM3,ENO,PGK,RAG2,GPM1,GAL7,GAL10,PDC2,TPI1,GAP1,FBA1,PYK1,RAG5,GAP3
GO:0019318	hexose metabolic process	4<br>3	15	4.33	8.80E-06	GAL80,PGK,RAG2,GPM1,GAL7,GAL10,GAL1,PDC2,TPI1,GAP1,FBA1,PYK1,RGT1,GAP3,VID24

GO.ID	Term	Annotated gene	Significant	Expected	P-value	Genes
GO:0019359	nicotinamide nucleotide biosynthetic pro...	25	11	2.52	1.10E-05	GPM3,ENO,PGK,RAG2,GPM1,TPI1,GAP1,FBA1,PYK1,RAG5,GAP3
GO:0019363	pyridine nucleotide biosynthetic process	25	11	2.52	1.10E-05	GPM3,ENO,PGK,RAG2,GPM1,TPI1,GAP1,FBA1,PYK1,RAG5,GAP3
GO:0009161	ribonucleoside monophosphate metabolic p...	84	22	8.45	1.60E-05	PRS5,GPM3,HPT1,GUA1,ENO,PGK,AAH1,RAG2,URA1,GPM1,ADK1,ADE2,PDC2,ADE5,7,TPI1,GAP1,FBA1,XPT1,ADE6,PYK1,RAG5,GAP3
GO:0071428	rRNA-containing ribonucleoprotein comple...	45	15	4.53	1.70E-05	SDA1,NOG1,ARX1,NOP53,RPS2,RPS3,RRS1,RLI1,MEX67,NMD3,RIX1,MTR2,NUG1,NOP9,LTV1
GO:0072521	purine-containing compound metabolic pro...	116	27	11.67	1.80E-05	GPM3,HPT1,GUA1,ENO,PGK,AAH1,RAG2,GPM1,ADK1,ADE2,MEU1,PDC2,SAH1,ADE5,7,TPI1,GAP1,FBA1,HMG1,XPT1,GUK1,ADE6,ACS2,PYK1,RAG5,ERG13,GAP3,MET6
GO:0009123	nucleoside monophosphate metabolic proce...	86	22	8.65	2.30E-05	PRS5,GPM3,HPT1,GUA1,ENO,PGK,AAH1,RAG2,URA1,GPM1,ADK1,ADE2,PDC2,ADE5,7,TPI1,GAP1,FBA1,XPT1,ADE6,PYK1,RAG5,GAP3
GO:0000054	ribosomal subunit export from nucleus	41	14	4.13	2.30E-05	SDA1,NOG1,ARX1,NOP53,RPS3,RRS1,RLI1,MEX67,NMD3,RIX1,MTR2,NUG1,NOP9,LTV1
GO:0033750	ribosome localization	41	14	4.13	2.30E-05	SDA1,NOG1,ARX1,NOP53,RPS3,RRS1,RLI1,MEX67,NMD3,RIX1,MTR2,NUG1,NOP9,LTV1
GO:0009117	nucleotide metabolic process	152	32	15.29	2.70E-05	APA2,PRS5,GPM3,HPT1,GUA1,ENO,PGK,AAH1,RAG2,URA1,GPM1,ADH4,ADK1,ADE2,HAM1,RNR2,PDC2,ADE5,7,TPI1,GAP1,FBA1,HMG1,XPT1,GUK1,ADE6,ACS2,PYK1,RAG5,ERG13,URA7,RNR1,GAP3

GO.ID	Term	Annotated gene	Significant	Expected	P-value	Genes
GO:0009126	purine nucleoside monophosphate metaboli...	75	20	7.55	2.90E-05	GPM3,HPT1,GUA1,ENO,PGK,AAH1,RAG2,GPM1,ADK1,ADE2,PDC2,ADE5,7,TPI1,GAP1,FBA1,XPT1,ADE6,PYK1,RAG5,GAP3
GO:0009167	purine ribonucleoside monophosphate meta...	75	20	7.55	2.90E-05	GPM3,HPT1,GUA1,ENO,PGK,AAH1,RAG2,GPM1,ADK1,ADE2,PDC2,ADE5,7,TPI1,GAP1,FBA1,XPT1,ADE6,PYK1,RAG5,GAP3
GO:0005996	monosaccharide metabolic process	47	15	4.73	3.00E-05	GAL80,PGK,RAG2,GPM1,GAL7,GAL10,GAL1,PDC2,TPI1,GAP1,FBA1,PYK1,RGT1,GAP3,VID24
GO:0072525	pyridine-containing compound biosyntheti...	32	12	3.22	3.10E-05	GPM3,ENO,PGK,RAG2,GPM1,TPI1,GAP1,FBA1,PDX3,PYK1,RAG5,GAP3
GO:0090305	nucleic acid phosphodiester bond hydroly...	100	24	10.06	3.10E-05	UTP13,UTP11,NOP58,ENP1,RLP7,MPP10,RRS1,NOB1,PWP2,DBP3,UTP10,NOP19,BMS1,UTP6,MTR4,UTP18,RRP5,DIP2,BRX1,FCF2,NOP14,NOC4,NOP9,RRP42
GO:0006753	nucleoside phosphate metabolic process	153	32	15.39	3.10E-05	APA2,PRS5,GPM3,HPT1,GUA1,ENO,PGK,AAH1,RAG2,URA1,GPM1,ADH4,ADK1,ADE2,HAM1,RNR2,PDC2,ADE5,7,TPI1,GAP1,FBA1,HMG1,XPT1,GUK1,ADE6,ACS2,PYK1,RAG5,ERG13,URA7,RNR1,GAP3

GO.ID	Term	Annotated gene	Significant	Expected	P-value	Genes
GO:0016070	RNA metabolic process	843	116	84.82	3.60E-05	SPB1,RPA49,NOP1,SOH1,DSS1,UTP5,EBP2,RPF2,RSE1,GAL80,MES1,HAS1,YTA7,MSW1,NOG1,UTP13,RPC37,DBP7,UTP15,UTP11,NOP53,NOP58,FHL1,NOP12,ENP1,CBF5,KLMA_20481,RLP7,UTP8,TPA1,MPP10,IPI3,NAN1,MRT4,RRS1,IPI1,DCAF13,DRS1,NOB1,RPL3,PAB1,MRPL15,PDC2,BFR2,PWP2,DBP3,RRP3,HAL9,VTS1,KSS1,MTO1,GUS1,ECM16,MDN1,UTP10,GAR1,CIC1,HAP1,ERB1,MCM1,RRP12,IMP4,YTM1,NOP19,RPC82,RIX1,UTP21,PUS1,BMS1,UTP6,PRP43,GEP3,MTR4,UTP18,ENP2,RPA135,RRP5,PWP1,NOP56,LAC9,UTP4,NUG1,RPC40,VAS1,NOP7,DIP2,IMP3,BRX1,URB1,DHR2,RPA190,FUN12,YAF9,NOP15,KRE33,EPL1,FCF2,KLMA_60313,NOP4,JHD2,CDC60,IKI3,ROX3,SPT21,NOP14,NOC4,RRP9,PUS4,KLMA_70408,NOP9,RRP42,PUB1,UTP25,HDA1,NOP2,ILS1
GO:0009058	biosynthetic process	1301	165	130.9	3.80E-05	HIS4,SAM2,RpL37a,RPA49,PRS5,GPM3,NOP1,OLE1,SOH1,UTP5,HPT1,GUA1,BIO2,RPL17B,GAL80,ENO,MES1,YTA7,LIP1,FAU1,PGK,CWH43,SCS7,MSW1,FUR1,AAH1,RAG2,URA1,TPS2,KLMA_20052,TSL1,GPM1,RPC37,UTP15,ADH4,UTP11,ADK1,RPS2,MET13,CYS4,SHM2,ERG3,FHL1,KLMA_20355,KLMA_20481,RPL22A,UTP8,TPA1,ERG28,CYP707A7,ORT1,ADE2,ERG9,IPI3,RPS3,RPL5,NAN1,LIA1,HAM1,GSH1,PHS1,IPI1,RNR2,ERG1,MEU1,ETT1,RPL3,PAB1,RLI1,KLMA_30320,GSY2,PDC2,SUR2,SER3,YIH1,PAN5,TIF32,HAL9,KSS1,ERG6,SAH1,ADE5,7,GUS1,RPL15B,dsd1,HEM13,TPI1,GAP1,RIB7,UTP10,HAP1,MCM1,FMS1,FBA1,ALD5,MAM33,HMG1,DPM1,RPC82,MET5,RIX1,XPT1,FAS1,RPL10A,GUK1,SHM1,LEU1,RPA135,PDX3,RPS14,SUR4,ADE6,ERG25,MSS51,LAC9,UTP4,RPC40,VAS1,NOP7,RPL2,ACS2,SHB17,URA2,KLMA_60069,PDC1,RPA190,FUN12,REV1,PYK1,LEU4,YAF9,EPL1,KLMA_60313,UGP1,RAG5,cyp524A1,JHD2,LEU3,ERG13,RPB1,CDC60,PET309,IKI3,URA7,ROX3,RNR1,FAS2,SPT21,TIF3,MET16,HEM14,RPL19B,DYS1,KLMA_70408,GAP3,MET3,MNN1,HDA1,NIP1,KLMA_80256,VID24,TPS1,MET6,ILS1
GO:0071826	ribonucleoprotein complex subunit organi...	115	26	11.57	4.50E-05	RPF2,RSE1,NOG1,NOP53,IPI3,RPL5,MRT4,IPI1,DRS1,RPL3,RLI1,TIF32,MDN1,RIX1,PRP43,RRP5,KLMA_60069,BRX1,FUN12,RSA4,TIF3,MAK21,RLP24,PUB1,NOP2,NIP1

GO.ID	Term	Annotated gene	Significant	Expected	P-value	Genes
GO:1901576	organic substance biosynthetic process	1289	163	129.69	5.50E-05	HIS4,SAM2,RpL37a,RPA49,PRS5,GPM3,NOP1,OLE1,SOH1,UTP5,HPT1,GUA1,BIO2,RPL17B,GAL80,ENO,MES1,YTA7,LIP1,FAU1,PGK,CWH43,SCS7,MSW1,FUR1,AAH1,RAG2,URA1,TPS2,KLMA_20052,TSL1,GPM1,RPC37,UTP15,ADH4,UTP11,ADK1,RPS2,MET13,CYS4,SHM2,ERG3,FHL1,KLMA_20355,KLMA_20481,RPL22A,UTP8,TPA1,ERG28,CYP707A7,ORT1,ADE2,ERG9,IPI3,RPS3,RPL5,NAN1,HAM1,GSH1,PHS1,IPI1,RNR2,ERG1,MEU1,ET T1,RPL3,PAB1,RLI1,KLMA_30320,GSY2,PDC2,SUR2,SER3,YIH1,PAN5,TIF32,HAL9,KSS1,ERG6,SAH1,ADE5,7,GUS1,RPL15B,dsd1,HEM13,TPI1,GAP1,RIB7,UTP10,HAP1,MCM1,FMS1,FBA1,ALD5,MAM33,HMG1,DPM1,RPC82,MET5,RIX1,XPT1,FAS1,RPL10A,GUK1,SHM1,LEU1,RPA135,PDX3,RPS14,SUR4,ADE6,ERG25,MSS51,LAC9,UTP4,RPC40,VAS1,NOP7,RPL2,ACS2,SHB17,URA2,KLMA_60069,PDC1,RPA190,FUN12,REV1,PYK1,LEU4,YAF9,EPL1,KLMA_60313,UGP1,RAG5,cyp524A1,JHD2,LEU3,ERG13,RPB1,CDC60,PET309,IKI3,URA7,ROX3,RNR1,FAS2,SPT21,TIF3,MET16,HEM14,RPL19B,KLMA_70408,GAP3,MET3,MNN1,HDA1,NIP1,KLMA_80256,VID24,TPS1,MET6,ILS1
GO:0009150	purine ribonucleotide metabolic process	97	23	9.76	5.70E-05	GPM3,HPT1,GUA1,ENO,PGK,RAG2,GPM1,ADK1,ADE2,PDC2,ADE5,7,TPI1,GAP1,FBA1,HMG1,XPT1,GUK1,ADE6,ACS2,PYK1,RAG5,ERG13,GAP3
GO:0009116	nucleoside metabolic process	34	12	3.42	6.30E-05	PRS5,HPT1,GUA1,FUR1,URA1,RNR2,MEU1,SAH1,XPT1,GUK1,URA7,RNR1
GO:0006163	purine nucleotide metabolic process	98	23	9.86	6.70E-05	GPM3,HPT1,GUA1,ENO,PGK,RAG2,GPM1,ADK1,ADE2,PDC2,ADE5,7,TPI1,GAP1,FBA1,HMG1,XPT1,GUK1,ADE6,ACS2,PYK1,RAG5,ERG13,GAP3
GO:0009142	nucleoside triphosphate biosynthetic pro...	40	13	4.02	8.30E-05	GPM3,ENO,PGK,RAG2,GPM1,TPI1,GAP1,FBA1,PYK1,RAG5,URA7,RNR1,GAP3

GO.ID	Term	Annotated gene	Significant	Expected	P-value	Genes
GO:1901657	glycosyl compound metabolic process	35	12	3.52	8.70E-05	PRS5,HPT1,GUA1,FUR1,URA1,RNR2,MEU1,SAH1,XPT1,GUK1,URA7,RNR1
GO:0005992	trehalose biosynthetic process	4	4	0.4	0.0001	TPS2,TSL1,UGP1,TPS1
GO:0009312	oligosaccharide biosynthetic process	4	4	0.4	0.0001	TPS2,TSL1,UGP1,TPS1
GO:0046351	disaccharide biosynthetic process	4	4	0.4	0.0001	TPS2,TSL1,UGP1,TPS1
GO:0044249	cellular biosynthetic process	1278	160	128.58	0.00013	HIS4,SAM2,RpL37a,RPA49,PRS5,GPM3,NOP1,OLE1,SOH1,UTP5,HPT1,GUA1,BIO2,RPL17B,GAL80,ENO,MES1,YTA7,LIP1,FAU1,PGK,CWH43,SCS7,MSW1,FUR1,AAH1,RAG2,URA1,TPS2,KLMA_20052,TSL1,GPM1,RPC37,UTP15,UTP11,ADK1,RPS2,MET13,CYS4,SHM2,ERG3,FHL1,KLMA_20355,KLMA_20481,RPL22A,UTP8,TPA1,ERG28,CYP707A7,ORT1,ADE2,ERG9,IPI3,RPS3,RPL5,NAN1,HAM1,GSH1,PHS1,IPI1,RNR2,ERG1,MEU1,ETT1,RPL3,PAB1,RLI1,KLMA_30320,GSY2,PDC2,SUR2,SER3,YIH1,PAN5,TIF32,HAL9,KSS1,ERG6,SAH1,ADE5,7,GUS1,RPL15B,dsd1,HEM13,TPI1,GAP1,RIB7,UTP10,HAP1,MCM1,FMS1,FBA1,ALD5,MAM33,HMG1,DPM1,RPC82,MET5,RIX1,XPT1,FAS1,RPL10A,GUK1,SHM1,LEU1,RPA135,PDX3,RPS14,SUR4,ADE6,ERG25,MSS51,LAC9,UTP4,RPC40,VAS1,NOP7,RPL2,ACS2,URA2,KLMA_60069,RPA190,FUN12,REV1,PYK1,LEU4,YAF9,EPL1,KLMA_60313,UGP1,RAG5,cyp524A1,JHD2,LEU3,ERG13,RPB1,CDC60,PET309,IKI3,URA7,ROX3,RNR1,FAS2,SPT21,TIF3,MET16,HEM14,RPL19B,KLMA_70408,GAP3,MET3,MNN1,HDA1,NIP1,KLMA_80256,VID24,TPS1,MET6,ILS1

GO.ID	Term	Annotated gene	Significant	Expected	P-value	Genes
GO:0006807	nitrogen compound metabolic process	1991	231	200.32	0.00013	SPB1,APA2,HIS4,SAM2,RpL37a,RPA49,PRS5,GPM3,NOP1,SOH1,DSS1,UTP5,HPT1,GUA1,BIO2,RPL17B,EBP2,RPF2,RSE1,GAL80,ENO,MES1,HAS1,TDA1,YTA7,LIP1,FAU1,PGK,CWH43,SCS7,MSW1,FUR1,AAH1,RAG2,NOG1,URA1,KLMA_20052,GPM1,UTP13,RPC37,DBP7,UTP15,HSL1,ADH4,UTP11,ADK1,NOP53,RPS2,MET13,CYS4,SHM2,NOP58,FHL1,KLMA_20355,NOP12,ENP1,CLN2,UBP3,CBF5,KLMA_20481,RLP7,RPL22A,UTP8,TPA1,ORT1,ADE2,MPP10,DTD1,IPI3,RPS3,RPL5,NAN1,LIA1,HAM1,MRT4,RRS1,GSH1,PHS1,IPI1,RNR2,DCAF13,MEU1,DRS1,ETT1,NOB1,RPL3,PAB1,RLI1,MRPL15,KLMA_30320,PDC2,SUR2,BFR2,SER3,PWP2,YIH1,DBP3,PAN5,RRP3,TIF32,HAL9,VTS1,RAD54,KSS1,SAH1,DUG1,ADE5,7,MTO1,GUS1,ECM16,RPL15B,dsd1,HEM13,TPI1,MDN1,GAP1,RIB7,UTP10,GAR1,CIC1,HAP1,ERB1,MCM1,IMP2,RRP12,IMP4,FMS1,FBA1,MAM33,YTM1,NOP19,HMG1,DPM1,RPC82,MET5,RIX1,XPT1,UTP21,PUS1,BMS1,RPL10A,GUK1,UTP6,PRP43,GEP3,SHM1,MTR4,UTP18,ENP2,LEU1,RPA135,PDX3,RPS14,ADE6,RRP5,PWP1,NOP56,MSS51,LAC9,UTP4,NUG1,RPC40,VAS1,NOP7,RPL2,DIP2,ACS2,MAP1,URA2,IMP3,KLMA_60069,PDC1,EXO1,MDM20,BRX1,URB1,DHR2,RPA190,FUN12,REV1,PYK1,LEU4,YAF9,NOP15,KRE33,EPL1,FCF2,KLMA_60313,UGP1,NOP4,RAG5,JHD2,LEU3,ERG13,RPB1,CDC60,PET309,IKI3,URA7,ROX3,RNR1,PCL6,HSL7,SPT21,NOP14,TIF3,MET16,NOC4,RRP9,HEM14,RPL19B,DYS1,PUS4,KLMA_70408,NOP9,GAP3,MET3,MNN1,RRP42,PUB1,UTP25,HDA1,NOP2,NIP1,KLMA_80256,DEF1,VID24,PTH2,MET6,ILS1
GO:0022618	ribonucleoprotein complex assembly	109	24	10.97	0.00014	RPF2,RSE1,NOG1,NOP53,IPI3,RPL5,MRT4,IPI1,DRS1,RPL3,TIF32,MDN1,RIX1,RRP5,KLMA_60069,BRX1,FUN12,RSA4,TIF3,MAK21,RLP24,PUB1,NOP2,NIP1
GO:0005975	carbohydrate metabolic process	137	28	13.78	0.00015	GPD1,GPM3,GAL80,ENO,PGK,RAG2,TPS2,TSL1,GPM1,GAL7,GAL10,GAL1,PGM2,GSY2,PDC2,SCW4,TPI1,GAP1,FBA1,MDH1,PYK1,RGT1,UGP1,RAG5,GAP3,BGL2,VID24,TPS1
GO:0009201	ribonucleoside triphosphate biosynthetic...	37	12	3.72	0.00016	GPM3,ENO,PGK,RAG2,GPM1,TPI1,GAP1,FBA1,PYK1,RAG5,URA7,GAP3

GO.ID	Term	Annotated gene	Significant	Expected	P-value	Genes
GO:0071166	ribonucleoprotein complex localization	84	20	8.45	0.00016	SDA1,NOG1,ARX1,NOP53,RPS2,ENP1,UTP8,RPS3,RRS1,RLI1,MEX67,NMD3,RIX1,MTR2,MTR4,NUG1,CEX1,NEW1,NOP9,LTV1
GO:0071426	ribonucleoprotein complex export from nu...	84	20	8.45	0.00016	SDA1,NOG1,ARX1,NOP53,RPS2,ENP1,UTP8,RPS3,RRS1,RLI1,MEX67,NMD3,RIX1,MTR2,MTR4,NUG1,CEX1,NEW1,NOP9,LTV1
GO:0006006	glucose metabolic process	32	11	3.22	0.00017	PGK,RAG2,GPM1,PDC2,TPI1,GAP1,FBA1,PYK1,RGT1,GAP3,VID24
GO:0019362	pyridine nucleotide metabolic process	43	13	4.33	0.00019	GPM3,ENO,PGK,RAG2,GPM1,ADH4,PDC2,TPI1,GAP1,FBA1,PYK1,RAG5,GAP3
GO:0046496	nicotinamide nucleotide metabolic proces...	43	13	4.33	0.00019	GPM3,ENO,PGK,RAG2,GPM1,ADH4,PDC2,TPI1,GAP1,FBA1,PYK1,RAG5,GAP3
GO:0006405	RNA export from nucleus	85	20	8.55	0.00019	SDA1,NOG1,ARX1,NOP53,RPS2,ENP1,UTP8,RPS3,RRS1,RLI1,MEX67,NMD3,RIX1,MTR2,MTR4,NUG1,CEX1,NEW1,NOP9,LTV1
GO:0032787	monocarboxylic acid metabolic process	105	23	10.56	0.00021	GPM3,OLE1,BIO2,ENO,PGK,SCS7,RAG2,GPM1,PHS1,PDC2,PAN5,TPI1,GAP1,FMS1,FBA1,ALD5,FAS1,SUR4,ACS2,PYK1,RAG5,FAS2,GAP3
GO:0072524	pyridine-containing compound metabolic p...	49	14	4.93	0.00021	GPM3,ENO,PGK,RAG2,GPM1,ADH4,PDC2,TPI1,GAP1,FBA1,PDX3,PYK1,RAG5,GAP3
GO:0009119	ribonucleoside metabolic process	23	9	2.31	0.00022	HPT1,GUA1,URA1,RNR2,MEU1,SAH1,GUK1,URA7,RNR1
GO:0046165	alcohol biosynthetic process	39	12	3.92	0.00028	ADH4,ERG3,ERG28,CYP707A7,ERG9,ERG1,ERG6,dsd1,ERG25,PDC1,cyp524A1,ERG13

GO.ID	Term	Annotated gene	Significant	Expected	P-value	Genes
GO:0090407	organophosphate biosynthetic process	178	33	17.91	0.00028	PRS5,GPM3,HPT1,GUA1,ENO,PGK,CWH43,AAH1,RAG2,URA1,KLMA_20052,GPM1,ADK1,ADE2,RNR2,SAH1,ADE5,7,TPI1,GAP1,FBA1,HMG1,DPM1,XPT1,GUK1,ADE6,ACS2,SHB17,PYK1,RAG5,ERG13,URA7,RNR1,GAP3
GO:0006732	coenzyme metabolic process	114	24	11.47	0.00029	SAM2,GPM3,BIO2,ENO,FAU1,PGK,RAG2,GPM1,ADH4,MET13,SHM2,PDC2,PAN5,TPI1,GAP1,FMS1,FBA1,HMG1,SHM1,ACS2,PYK1,RAG5,ERG13,GAP3
GO:0006754	ATP biosynthetic process	34	11	3.42	0.00031	GPM3,ENO,PGK,RAG2,GPM1,TPI1,GAP1,FBA1,PYK1,RAG5,GAP3
GO:0051188	cofactor biosynthetic process	101	22	10.16	0.00031	SAM2,GPM3,BIO2,ENO,FAU1,PGK,RAG2,GPM1,GSH1,PAN5,HEM13,TPI1,GAP1,FMS1,FBA1,PDX3,ACS2,PYK1,RAG5,MET16,HEM14,GAP3
GO:0006696	ergosterol biosynthetic process	24	9	2.41	0.00031	ERG3,ERG28,CYP707A7,ERG9,ERG1,ERG6,ERG25,cyp524A1,ERG13
GO:0008204	ergosterol metabolic process	24	9	2.41	0.00031	ERG3,ERG28,CYP707A7,ERG9,ERG1,ERG6,ERG25,cyp524A1,ERG13
GO:0016128	phytosteroid metabolic process	24	9	2.41	0.00031	ERG3,ERG28,CYP707A7,ERG9,ERG1,ERG6,ERG25,cyp524A1,ERG13
GO:0016129	phytosteroid biosynthetic process	24	9	2.41	0.00031	ERG3,ERG28,CYP707A7,ERG9,ERG1,ERG6,ERG25,cyp524A1,ERG13
GO:0044108	cellular alcohol biosynthetic process	24	9	2.41	0.00031	ERG3,ERG28,CYP707A7,ERG9,ERG1,ERG6,ERG25,cyp524A1,ERG13
GO:0097384	cellular lipid biosynthetic process	24	9	2.41	0.00031	ERG3,ERG28,CYP707A7,ERG9,ERG1,ERG6,ERG25,cyp524A1,ERG13
GO:0006611	protein export from nucleus	89	20	8.95	0.00038	SDA1,NOG1,ARX1,NOP53,RPS2,ENP1,UTP8,RPS3,RRS1,RLI1,MEX67,NMD3,RIX1,MTR2,MTR4,NUG1,CEX1,NEW1,NOP9,LTV1

GO.ID	Term	Annotated gene	Significant	Expected	P-value	Genes
GO:0009145	purine nucleoside triphosphate biosynthe...	35	11	3.52	0.00041	GPM3,ENO,PGK,RAG2,GPM1,TPI1,GAP1,FBA1,PYK1,RAG5,GAP3
GO:0009206	purine ribonucleoside triphosphate biosy...	35	11	3.52	0.00041	GPM3,ENO,PGK,RAG2,GPM1,TPI1,GAP1,FBA1,PYK1,RAG5,GAP3
GO:0051168	nuclear export	90	20	9.06	0.00044	SDA1,NOG1,ARX1,NOP53,RPS2,ENP1,UTP8,RPS3,RRS1,RLI1,MEX67,NMD3,RIX1,MTR2,MTR4,NUG1,CEX1,NEW1,NOP9,LTV1
GO:1902652	secondary alcohol metabolic process	25	9	2.52	0.00045	ERG3,ERG28,CYP707A7,ERG9,ERG1,ERG6,ERG25,cyp524A1,ERG13
GO:1902653	secondary alcohol biosynthetic process	25	9	2.52	0.00045	ERG3,ERG28,CYP707A7,ERG9,ERG1,ERG6,ERG25,cyp524A1,ERG13
GO:0006012	galactose metabolic process	5	4	0.5	0.00046	GAL80,GAL7,GAL10,GAL1
GO:0051186	cofactor metabolic process	169	31	17	0.00053	SAM2,GPM3,BIO2,ENO,FAU1,PGK,RAG2,GPM1,ADH4,MET13,SHM2,GSH1,PDC2,PAN5,SAH1,DUG1,HEM13,TPI1,GAP1,FMS1,FBA1,HMG1,SHM1,PDX3,ACS2,PYK1,RAG5,ERG13,MET16,HEM14,GAP3
GO:1901137	carbohydrate derivative biosynthetic pro...	177	32	17.81	0.00056	PRS5,GPM3,HPT1,GUA1,ENO,PGK,CWH43,RAG2,URA1,GPM1,ADK1,ADE2,RNR2,MEU1,KLMA_30320,ADE5,7,TPI1,GAP1,FBA1,DPM1,XPT1,GUK1,ADE6,ACS2,SHB17,PYK1,RAG5,URA7,RNR1,GAP3,MNN1
GO:0044107	cellular alcohol metabolic process	26	9	2.62	0.00063	ERG3,ERG28,CYP707A7,ERG9,ERG1,ERG6,ERG25,cyp524A1,ERG13

GO.ID	Term	Annotated gene	Significant	Expected	P-value	Genes
GO:0050657	nucleic acid transport	94	20	9.46	0.0008	SDA1,NOG1,ARX1,NOP53,RPS2,ENP1,UTP8,RPS3,RRS1,RLI1,MEX67,NMD3,RIX1,MTR2,MTR4,NUG1,CEX1,NEW1,NOP9,LTV1
GO:0050658	RNA transport	94	20	9.46	0.0008	SDA1,NOG1,ARX1,NOP53,RPS2,ENP1,UTP8,RPS3,RRS1,RLI1,MEX67,NMD3,RIX1,MTR2,MTR4,NUG1,CEX1,NEW1,NOP9,LTV1
GO:0051236	establishment of RNA localization	94	20	9.46	0.0008	SDA1,NOG1,ARX1,NOP53,RPS2,ENP1,UTP8,RPS3,RRS1,RLI1,MEX67,NMD3,RIX1,MTR2,MTR4,NUG1,CEX1,NEW1,NOP9,LTV1
GO:1901135	carbohydrate derivative metabolic proces...	260	42	26.16	0.00094	PRS5,GPD1,GPM3,HPT1,GUA1,PFK1,ENO,PGK,CWH43,FUR1,RAG2,URA1,GPM1,ADK1,ADE2,RNR2,MEU1,KLMA_30320,PDC2,SUR2,SAH1,ADE5,7,TPI1,GAP1,FBA1,HMG1,DPM1,XPT1,GUK1,ADE6,ACS2,SHB17,PYK1,UGP1,RAG5,ERG13,PFK2,URA7,RNR1,GAP3,MNN1
GO:1901617	organic hydroxy compound biosynthetic pr...	50	13	5.03	0.00096	ADH4,ERG3,ERG28,CYP707A7,ERG9,ERG1,ERG6,dsd1,PDX3,ERG25,PDC1,cyp524A1,ERG13
GO:0009141	nucleoside triphosphate metabolic proces...	69	16	6.94	0.00101	GPM3,ENO,PGK,RAG2,GPM1,ADK1,HAM1,PDC2,TPI1,GAP1,FBA1,PYK1,RAG5,URA7,RNR1,GAP3
GO:0000464	endonucleolytic cleavage in ITS1 upstrea...	3	3	0.3	0.00101	DBP3,RRP5,BRX1
GO:0019637	organophosphate metabolic process	270	43	27.17	0.0011	APA2,LPP1,PRS5,GPD1,GPM3,HPT1,GUA1,PFK1,ENO,PGK,CWH43,SCS7,AAH1,RAG2,URA1,KLMA_20052,GPM1,ADH4,ADK1,ADE2,HAM1,RNR2,PDC2,SUR2,SAH1,ADE5,7,TPI1,GAP1,FBA1,HMG1,DPM1,XPT1,GUK1,ADE6,ACS2,SHB17,PYK1,RAG5,ERG13,PFK2,URA7,RNR1,GAP3
GO:0009133	nucleoside diphosphate biosynthetic proc...	6	4	0.6	0.00128	ADK1,RNR2,GUK1,RNR1

GO.ID	Term	Annotated gene	Significant	Expected	P-value	Genes
GO:0006066	alcohol metabolic process	52	13	5.23	0.00143	ADH4,ERG3,ERG28,CYP707A7,ERG9,ERG1,PDC2,ERG6,dsd1,ERG25,PDC1,cyp524A1,ERG13
GO:0009163	nucleoside biosynthetic process	14	6	1.41	0.00148	HPT1,GUA1,URA1,MEU1,GUK1,URA7
GO:0042455	ribonucleoside biosynthetic process	14	6	1.41	0.00148	HPT1,GUA1,URA1,MEU1,GUK1,URA7
GO:1901659	glycosyl compound biosynthetic process	14	6	1.41	0.00148	HPT1,GUA1,URA1,MEU1,GUK1,URA7
GO:0006694	steroid biosynthetic process	29	9	2.92	0.00153	ERG3,ERG28,CYP707A7,ERG9,ERG1,ERG6,ERG25,cyp524A1,ERG13
GO:0016126	sterol biosynthetic process	29	9	2.92	0.00153	ERG3,ERG28,CYP707A7,ERG9,ERG1,ERG6,ERG25,cyp524A1,ERG13
GO:0000055	ribosomal large subunit export from nucl...	24	8	2.41	0.00167	SDA1,ARX1,NOP53,RRS1,MEX67,NMD3,MTR2,NUG1
GO:0044272	sulfur compound biosynthetic process	47	12	4.73	0.00178	SAM2,BIO2,MET13,CYS4,GSH1,MEU1,PDC2,MET5,ACS2,MET16,MET3,MET6
GO:0019439	aromatic compound catabolic process	136	25	13.68	0.00178	GPM3,DSS1,ENO,PGK,AAH1,RAG2,GPM1,TPA1,DTD1,HAM1, MRT4,PAB1,VTS1,SAH1,TPI1,GAP1,FBA1,MTR4,PDC1,PYK1,RAG5,JHD2,GAP3,RRP42,PUB1
GO:0009108	coenzyme biosynthetic process	81	17	8.15	0.00229	SAM2,GPM3,BIO2,ENO,FAU1,PGK,RAG2,GPM1,PAN5,TPI1,GAP1,FMS1,FBA1,ACS2,PYK1,RAG5,GAP3
GO:0043603	cellular amide metabolic process	347	51	34.91	0.00243	RpL37a,BIO2,RPL17B,MES1,LIP1,FAU1,MSW1,UTP11,RPS2,MET13,SHM2,KLMA_20355,RPL22A,TPA1,RPS3,RPL5,GSH1,ETT1, RPL3,PAB1,RLI1,YIH1,PAN5,TIF32,DUG1,GUS1,RPL15B,IMP2,

GO.ID	Term	Annotated gene	Significant	Expected	P-value	Genes
						FMS1,MAM33,HMG1,RPL10A,SHM1,RPS14,MSS51,VAS1,RPL2,ACS2,KLMA_60069,FUN12,KLMA_60313,ERG13,CDC60,PET309,IKI3,TIF3,MET16,RPL19B,NIP1,KLMA_80256,ILS1
GO:0016125	sterol metabolic process	31	9	3.12	0.00257	ERG3,ERG28,CYP707A7,ERG9,ERG1,ERG6,ERG25,cyp524A1,ERG13
GO:1902626	assembly of large subunit precursor of p...	7	4	0.7	0.00275	RPF2,NOG1,RLP24,NOP2
GO:0031503	protein-containing complex localization	104	20	10.46	0.00294	SDA1,NOG1,ARX1,NOP53,RPS2,ENP1,UTP8,RPS3,RRS1,RLI1,MEX67,NMD3,RIX1,MTR2,MTR4,NUG1,CEX1,NEW1,NOP9,LTV1
GO:0009112	nucleobase metabolic process	26	8	2.62	0.00297	AAH1,URA1,ADE2,ADE5,7,XPT1,URA2,URA7,MET6
GO:0017144	drug metabolic process	180	30	18.11	0.00318	SAM2,ACO2,GPM3,BIO2,ENO,PGK,RAG2,GPM1,ADK1,CYS4,SHM2,MEU1,PDC2,TPI1,GAP1,RIB7,FMS1,FBA1,ALD5,MET5,SHM1,PDX3,ACS2,PDC1,MDH1,PYK1,RAG5,GAP3,MET3,MET6
GO:0008202	steroid metabolic process	32	9	3.22	0.00326	ERG3,ERG28,CYP707A7,ERG9,ERG1,ERG6,ERG25,cyp524A1,ERG13
GO:0006520	cellular amino acid metabolic process	173	29	17.41	0.00341	HIS4,SAM2,GUA1,MES1,MSW1,ADH4,MET13,CYS4,SHM2,ORT1,DTD1,MEU1,SER3,SAH1,GUS1,MET5,SHM1,LEU1,ADE6,VAS1,URA2,PDC1,LEU4,LEU3,CDC60,URA7,MET3,MET6,ILS1
GO:0006733	oxidoreduction coenzyme metabolic proces...	57	13	5.73	0.00346	GPM3,ENO,PGK,RAG2,GPM1,ADH4,PDC2,TPI1,GAP1,FBA1,PYK1,RAG5,GAP3
GO:1901605	alpha-amino acid metabolic process	113	21	11.37	0.00358	HIS4,SAM2,GUA1,MET13,CYS4,SHM2,ORT1,DTD1,MEU1,SER3,SAH1,SHM1,LEU1,ADE6,URA2,PDC1,LEU4,LEU3,URA7,MET3,MET6
GO:1901361	organic cyclic compound catabolic proces...	143	25	14.39	0.00364	GPM3,DSS1,ENO,PGK,AAH1,RAG2,GPM1,TPA1,DTD1,HAM1,MRT4,PAB1,VTS1,SAH1,TPI1,GAP1,FBA1,MTR4,PDC1,PYK1,RAG5,JHD2,GAP3,RRP42,PUB1

GO.ID	Term	Annotated gene	Significant	Expected	P-value	Genes
GO:0009199	ribonucleoside triphosphate metabolic pr...	64	14	6.44	0.00368	GPM3,ENO,PGK,RAG2,GPM1,ADK1,PDC2,TPI1,GAP1,FBA1,PYK1,RAG5,URA7,GAP3
GO:0009403	toxin biosynthetic process	4	3	0.4	0.00374	CYS4,MET5,MET3
GO:0009404	toxin metabolic process	4	3	0.4	0.00374	CYS4,MET5,MET3
GO:0019320	hexose catabolic process	4	3	0.4	0.00374	GAL7,GAL10,PYK1
GO:0035999	tetrahydrofolate interconversion	4	3	0.4	0.00374	MET13,SHM2,SHM1
GO:0070813	hydrogen sulfide metabolic process	4	3	0.4	0.00374	CYS4,MET5,MET3
GO:0070814	hydrogen sulfide biosynthetic process	4	3	0.4	0.00374	CYS4,MET5,MET3
GO:1901070	guanosine-containing compound biosynthet...	4	3	0.4	0.00374	HPT1,GUA1,GUK1
GO:0000056	ribosomal small subunit export from nucl...	12	5	1.21	0.00436	RPS3,MEX67,MTR2,NOP9,LTV1
GO:0006730	one-carbon metabolic process	12	5	1.21	0.00436	SAM2,MET13,SHM2,SAH1,SHM1
GO:0008610	lipid biosynthetic process	138	24	13.88	0.00467	OLE1,LIP1,CWH43,SCS7,KLMA_20052,ERG3,ERG28,CYP707A7,ERG9,PHS1,ERG1,SUR2,ERG6,SAH1,dsd1,HMG1,DPM1,FAS1,SUR4,ERG25,cyp524A1,ERG13,URA7,FAS2
GO:0000103	sulfate assimilation	8	4	0.8	0.00507	MET5,MET10,MET16,MET3
GO:0005991	trehalose	8	4	0.8	0.00507	TPS2,TSL1,UGP1,TPS1

GO.ID	Term	Annotated gene	Significant	Expected	P-value	Genes
	metabolic process					
GO:0006551	leucine metabolic process	8	4	0.8	0.00507	DTD1,LEU1,LEU4,LEU3
GO:0009218	pyrimidine ribonucleotide metabolic proc...	8	4	0.8	0.00507	URA1,RNR2,URA7,RNR1
GO:0019748	secondary metabolic process	8	4	0.8	0.00507	CYS4,GSH1,MET5,MET3
GO:0044550	secondary metabolite biosynthetic proces...	8	4	0.8	0.00507	CYS4,GSH1,MET5,MET3
GO:0044270	cellular nitrogen compound catabolic pro...	139	24	13.99	0.00513	GPM3,DSS1,ENO,PGK,AAH1,RAG2,GPM1,TPA1,HAM1,MRT4,PAB1,VTS1,SAH1,TPI1,GAP1,FBA1,MTR4,PDC1,PYK1,RAG5,JHD2,GAP3,RRP42,PUB1
GO:0046700	heterocycle catabolic process	139	24	13.99	0.00513	GPM3,DSS1,ENO,PGK,AAH1,RAG2,GPM1,TPA1,HAM1,MRT4,PAB1,VTS1,SAH1,TPI1,GAP1,FBA1,MTR4,PDC1,PYK1,RAG5,JHD2,GAP3,RRP42,PUB1
GO:0006403	RNA localization	109	20	10.97	0.00515	SDA1,NOG1,ARX1,NOP53,RPS2,ENP1,UTP8,RPS3,RRS1,RLI1,MEX67,NMD3,RIX1,MTR2,MTR4,NUG1,CEX1,NEW1,NOP9,LTV1
GO:0044271	cellular nitrogen compound biosynthetic ...	910	112	91.56	0.00541	RpL37a,RPA49,PRS5,GPM3,NOP1,SOH1,UTP5,HPT1,GUA1,BIO2,RPL17B,GAL80,ENO,MES1,YTA7,LIP1,FAU1,PGK,MSW1,FUR1,AAH1,RAG2,URA1,GPM1,RPC37,UTP15,UTP11,ADK1,RPS2,FHL1,KLMA_20355,KLMA_20481,RPL22A,UTP8,TPA1,ADE2,RPS3,RPL5,NAN1,HAM1,GSH1,RNR2,MEU1,ETT1,RPL3,PAB1,RLI1,PDC2,YIH1,PAN5,TIF32,HAL9,KSS1,ADE5,7,GUS1,RPL15B,dsd1,HEM13,TPI1,GAP1,RIB7,UTP10,HAP1,MCM1,FMS1,FBA1,MAM33,RPC82,XPT1,RPL10A,GUK1,RPA135,PDX3,RPS14,ADE6,MSS51,LAC9,UTP4,RPC40,VAS1,RPL2,ACS2,URA2,KLMA_60069,RPA190,FUN12,REV1,PYK1,YAF9,EPL1,KLMA_60313,RAG

GO.ID	Term	Annotated gene	Significant	Expected	P-value	Genes
						5,JHD2,RPB1,CDC60,PET309,IKI3,URA7,ROX3,RNR1,SPT21,TIF3,MET16,HEM14,RPL19B,KLMA_70408,GAP3,HDA1,NIP1,KLMA_80256,MET6,ILS1
GO:0046034	ATP metabolic process	60	13	6.04	0.00553	GPM3,ENO,PGK,RAG2,GPM1,ADK1,PDC2,TPI1,GAP1,FBA1,PYK1,RAG5,GAP3
GO:0043604	amide biosynthetic process	302	44	30.38	0.00577	RpL37a,BIO2,RPL17B,MES1,LIP1,FAU1,MSW1,UTP11,RPS2,KLMA_20355,RPL22A,TPA1,RPS3,RPL5,GSH1,ETT1,RPL3,PAB1,RLI1,YIH1,PAN5,TIF32,GUS1,RPL15B,FMS1,MAM33,RPL10A,RPS14,MSS51,VAS1,RPL2,ACS2,KLMA_60069,FUN12,KLMA_60313,CDC60,PET309,IKI3,TIF3,MET16,RPL19B,NIP1,KLMA_80256,ILS1
GO:0000096	sulfur amino acid metabolic process	23	7	2.31	0.00578	SAM2,MET13,CYS4,MEU1,MET5,MET3,MET6
GO:0006091	generation of precursor metabolites and ...	103	19	10.36	0.00597	ACO2,GPM3,ENO,PGK,RAG2,GPM1,GSY2,PDC2,TPI1,GAP1,HAP1,FBA1,MAM33,MDH1,PYK1,UGP1,RAG5,GAP3,GDS1
GO:0006575	cellular modified amino acid metabolic p...	41	10	4.13	0.00604	FAU1,MET13,SHM2,GSH1,PAN5,SAH1,DUG1,FMS1,SHM1,MET16
GO:0070925	organelle assembly	89	17	8.95	0.00637	RPF2,NOP53,IPI3,RPL5,MRT4,IPI1,DRS1,RPL3,MDN1,RIX1,RRP5,KLMA_60069,BRX1,FUN12,RSA4,MAK21,PUB1
GO:0006913	nucleocytoplasmic transport	127	22	12.78	0.00698	GSP1,SDA1,NOG1,ARX1,NOP53,RPS2,ENP1,UTP8,RPS3,RRS1,RLI1,MEX67,NMD3,RIX1,MTR2,MTR4,NUG1,CEX1,NEW1,NOP9,LTV1,KAP123
GO:0034655	nucleobase-containing compound catabolic...	127	22	12.78	0.00698	GPM3,DSS1,ENO,PGK,RAG2,GPM1,TPA1,HAM1,MRT4,PAB1,VTS1,SAH1,TPI1,GAP1,FBA1,MTR4,PYK1,RAG5,JHD2,GAP3,RRP42,PUB1
GO:0051169	nuclear transport	127	22	12.78	0.00698	GSP1,SDA1,NOG1,ARX1,NOP53,RPS2,ENP1,UTP8,RPS3,RRS1,RLI1,MEX67,NMD3,RIX1,MTR2,MTR4,NUG1,CEX1,NEW1,NOP9,LTV1,KAP123

GO.ID	Term	Annotated gene	Significant	Expected	P-value	Genes
GO:0009205	purine ribonucleoside triphosphate metab...	62	13	6.24	0.00739	GPM3,ENO,PGK,RAG2,GPM1,ADK1,PDC2,TPI1,GAP1,FBA1,PYK1,RAG5,GAP3
GO:0090304	nucleic acid metabolic process	1024	123	103.03	0.00789	SPB1,RPA49,NOP1,SOH1,DSS1,UTP5,EBP2,RPF2,RSE1,GAL80,MES1,HAS1,YTA7,MSW1,NOG1,UTP13,RPC37,DBP7,UTP15,UTP11,ADK1,NOP53,NOP58,FHL1,NOP12,ENP1,CBF5,KLMA_20481,RLP7,UTP8,TPA1,MPP10,IPI3,NAN1,MRT4,RRS1,IPI1,DCAF13,DRS1,NOB1,RPL3,PAB1,MRPL15,PDC2,BFR2,PWP2,DBP3,RRP3,HAL9,VTS1,RAD54,KSS1,MTO1,GUS1,ECM16,MDN1,UTP10,GAR1,CIC1,HAP1,ERB1,MCM1,RRP12,IMP4,YTM1,NOP19,RPC82,RIX1,UTP21,PUS1,BMS1,UTP6,PRP43,GEP3,MTR4,UTP18,ENP2,RPA135,RRP5,PWP1,NOP56,LAC9,UTP4,NUG1,RPC40,VAS1,NOP7,DIP2,IMP3,EXO1,BRX1,URB1,DHR2,RPA190,FUN12,REV1,YAF9,NOP15,KRE33,EPL1,FCF2,KLMA_60313,NOP4,JHD2,RPB1,CDC60,IKI3,ROX3,RNR1,SPT21,NOP14,NOC4,RRP9,PUS4,KLMA_70408,NOP9,RRP42,PUB1,UTP25,HDA1,NOP2,DEF1,ILS1
GO:0006760	folic acid-containing compound metabolic...	9	4	0.91	0.00841	FAU1,MET13,SHM2,SHM1
GO:0042451	purine nucleoside biosynthetic process	9	4	0.91	0.00841	HPT1,GUA1,MEU1,GUK1
GO:0043101	purine-containing compound salvage	9	4	0.91	0.00841	HPT1,AAH1,MEU1,XPT1
GO:0046129	purine ribonucleoside biosynthetic proce...	9	4	0.91	0.00841	HPT1,GUA1,MEU1,GUK1

GO.ID	Term	Annotated gene	Significant	Expected	P-value	Genes
GO:0009144	purine nucleoside triphosphate metabolic...	63	13	6.34	0.00848	GPM3,ENO,PGK,RAG2,GPM1,ADK1,PDC2,TPI1,GAP1,FBA1,PYK1,RAG5,GAP3
GO:1901615	organic hydroxy compound metabolic proce...	70	14	7.04	0.00852	ADH4,ERG3,ERG28,CYP707A7,ERG9,ERG1,PDC2,ERG6,dsd1,PDX3,ERG25,PDC1,cyp524A1,ERG13
GO:0000097	sulfur amino acid biosynthetic process	19	6	1.91	0.00864	MET13,CYS4,MEU1,MET5,MET3,MET6
GO:0006633	fatty acid biosynthetic process	19	6	1.91	0.00864	OLE1,SCS7,PHS1,FAS1,SUR4,FAS2
GO:0046112	nucleobase biosynthetic process	19	6	1.91	0.00864	AAH1,URA1,ADE5,7,URA2,URA7,MET6
GO:0009098	leucine biosynthetic process	5	3	0.5	0.00865	LEU1,LEU4,LEU3
GO:0031120	snRNA pseudouridine synthesis	5	3	0.5	0.00865	CBF5,GAR1,PUS1
GO:0040031	snRNA modification	5	3	0.5	0.00865	CBF5,GAR1,PUS1
GO:0016051	carbohydrate biosynthetic process	50	11	5.03	0.00924	PGK,RAG2,TPS2,TSL1,GPM1,GSY2,TPI1,FBA1,UGP1,VID24,TPS1
GO:0042278	purine nucleoside metabolic process	14	5	1.41	0.00931	HPT1,GUA1,MEU1,SAH1,GUK1
GO:0046128	purine ribonucleoside	14	5	1.41	0.00931	HPT1,GUA1,MEU1,SAH1,GUK1

GO.ID	Term	Annotated gene	Significant	Expected	P-value	Genes
	metabolic process					
GO:0006790	sulfur compound metabolic process	93	17	9.36	0.00997	SAM2,BIO2,MET13,CYS4,GSH1,MEU1,PDC2,SAH1,DUG1,HMG1,MET5,MET10,ACS2,ERG13,MET16,MET3,MET6

GO terms enriched in significantly up-regulated genes (biological process)

GO.ID	Term	Annotated gene	Significant	Expected	P-value	Genes
GO:0016042	lipid catabolic process	31	19	3.08	2.50E-12	PLB,PXA1,POT1,POX1,SPO1,MDH3,SPO14,PXA2,GDE1,KLMA_50156,SPS19,TES1,ANT1,KLMA_70317,LPX1,FOX2,ICL2,PDH1,CIT3
GO:0044242	cellular lipid catabolic process	26	17	2.58	8.30E-12	PLB,PXA1,POT1,POX1,SPO1,MDH3,PXA2,GDE1,KLMA_50156,SPS19,TES1,ANT1,LPX1,FOX2,ICL2,PDH1,CIT3
GO:0009062	fatty acid catabolic process	13	12	1.29	9.10E-12	PXA1,POT1,POX1,MDH3,PXA2,SPS19,TES1,ANT1,FOX2,ICL2,PDH1,CIT3
GO:0016054	organic acid catabolic process	47	21	4.67	5.30E-10	CHA1,PXA1,POT1,POX1,ARO10,GDH2,GAD1,MDH3,UGA1,PXA2,CAR1,CAR2,PUT1,SPS19,TES1,gabD,ANT1,FOX2,ICL2,PDH1,CIT3
GO:0046395	carboxylic acid catabolic process	47	21	4.67	5.30E-10	CHA1,PXA1,POT1,POX1,ARO10,GDH2,GAD1,MDH3,UGA1,PXA2,CAR1,CAR2,PUT1,SPS19,TES1,gabD,ANT1,FOX2,ICL2,PDH1,CIT3
GO:0044282	small molecule catabolic process	68	25	6.76	1.70E-09	CHA1,URH1,MIOX5,KLMA_20057,PXA1,POT1,POX1,ARO10,GDH2,GAD1,MDH3,UGA1,PXA2,CAR1,CAR2,PUT1,SPS19,TES1,gabD,ANT1,FOX2,ICL2,PDH1,CIT3,KLMA_80176
GO:0072329	monocarboxylic acid catabolic process	24	14	2.39	5.50E-09	PXA1,POT1,POX1,MDH3,UGA1,PXA2,SPS19,TES1,gabD,ANT1,FOX2,ICL2,PDH1,CIT3
GO:0019395	fatty acid oxidation	10	9	0.99	7.80E-09	PXA1,POT1,POX1,PEX11,MDH3,PXA2,TES1,ANT1,FOX2
GO:0034440	lipid oxidation	10	9	0.99	7.80E-09	PXA1,POT1,POX1,PEX11,MDH3,PXA2,TES1,ANT1,FOX2

GO.ID	Term	Annotated gene	Significant	Expected	P-value	Genes
GO:0006635	fatty acid beta-oxidation	9	8	0.89	7.20E-08	PXA1,POT1,POX1,MDH3,PXA2,TES1,ANT1,FOX2
GO:0030258	lipid modification	20	11	1.99	5.90E-07	ATG26,PXA1,POT1,POX1,PEX11,MDH3,PXA2,TES1,YMR1,ANT1,FOX2
GO:0006631	fatty acid metabolic process	38	15	3.78	1.20E-06	PXA1,POT1,POX1,PEX11,MDH3,PXA2,SPS19,TES1,ETR1,ANT1,FOX2,ICL2,PDH1,CIT3,CRC1
GO:0016999	antibiotic metabolic process	40	15	3.98	2.60E-06	CIT1,IAH1,KLMA_30124,ACO2,ACS1,MDH3,MDH2,IDP1,ALD4,SFA1,CTT1,KLMA_60405,ICL2,PDH1,CIT3
GO:0006536	glutamate metabolic process	9	6	0.89	6.00E-05	GDH2,GAD1,IDP1,GLT1,PUT1,gabD
GO:0009065	glutamine family amino acid catabolic pr...	9	6	0.89	6.00E-05	GDH2,GAD1,CAR1,CAR2,PUT1,gabD
GO:0055114	oxidation-reduction process	106	24	10.54	7.10E-05	MIC17,CIT1,PXA1,POT1,POX1,PEX11,KLMA_30124,ACO2,ACS1,KLMA_40010,MDH3,MDH2,IDP1,PXA2,ALD4,SFA1,TES1,ETR1,PHO85,KLMA_60405,ANT1,FOX2,CIT3,SGA1
GO:0022414	reproductive process	232	41	23.06	0.00012	MATALPHA1,KAR4,PRM1,ATG26,CDC7,DIT2,STE3,FUS3,DMC1,CSM1,IME4,RIM15,SPS4,KLMA_20368,UBI4,MUS81,MSC1,MLH1,STE4,MND1,MLH3,PRR1,KLMA_40061,KLMA_40062,CHS1,SPO14,ELM1,GAS4,HOP1,ECO1,QDR3,FIG1,AXL1,SPS19,GPA1,MF(ALPHA)1,RAD1,SST2,CRR1,KLMA_70118,SNF8
GO:0005975	carbohydrate metabolic process	137	28	13.62	0.00012	ATG26,PGU1,INU1,BIG1,MIOX5,ERT1,GPD2,CTS2,RDS2,KLMA_30011,ARA1,MDH3,GID8,KLMA_40105,KLMA_40133,CHS1,MDH2,DAK1,INO1,PHO85,FBP1,CRR1,ATH1,KLMA_70303,KLMA_70317,SGA1,KLMA_80176,GUT1
GO:0032787	monocarboxylic acid metabolic process	105	23	10.44	0.00017	ECM31,PXA1,POT1,POX1,GPD2,IAH1,PEX11,ACS1,MDH3,ISA1,UGA1,PXA2,ALD4,SPS19,TES1,gabD,ETR1,ANT1,FOX2,ICL2,PDH1,CIT3,CRC1
GO:0043648	dicarboxylic acid metabolic process	23	9	2.29	0.0002	GDH2,GAD1,MDH3,IDP1,GLT1,PUT1,gabD,KLMA_60405,ARO7

GO.ID	Term	Annotated gene	Significant	Expected	P-value	Genes
GO:0019953	sexual reproduction	113	24	11.23	0.00021	KAR4,PRM1,ATG26,DIT2,STE3,FUS3,SPS4,KLMA_20368,STE4,PRR1,KLMA_40061,CHS1,SPO14,GAS4,QDR3,FIG1,AXL1,SPS19,GPA1,MF(ALPHA)1,SST2,CRR1,KLMA_70118,SNF8
GO:0044703	multi-organism reproductive process	113	24	11.23	0.00021	KAR4,PRM1,ATG26,DIT2,STE3,FUS3,SPS4,KLMA_20368,STE4,PRR1,KLMA_40061,CHS1,SPO14,GAS4,QDR3,FIG1,AXL1,SPS19,GPA1,MF(ALPHA)1,SST2,CRR1,KLMA_70118,SNF8
GO:0000003	reproduction	240	41	23.85	0.00025	MATALPHA1,KAR4,PRM1,ATG26,CDC7,DIT2,STE3,FUS3,DMC1,CSM1,IME4,RIM15,SPS4,KLMA_20368,UBI4,MUS81,MSC1,MLH1,STE4,MND1,MLH3,PRR1,KLMA_40061,KLMA_40062,CHS1,SPO14,ELM1,GAS4,HOP1,ECO1,QDR3,FIG1,AXL1,SPS19,GPA1,MF(ALPHA)1,RAD1,SST2,CRR1,KLMA_70118,SNF8
GO:0008643	carbohydrate transport	11	6	1.09	0.00028	HGT1,STL1,RAG1,LAC12
GO:0006082	organic acid metabolic process	305	49	30.31	0.00029	CHA1,GLY1,ECM31,KLMA_20057,CIT1,PXA1,POT1,DSD1,POX1,GPD2,AGX1,ARO10,IAH1,PEX11,GDH2,KLMA_30124,ACO2,DPL1,GAD1,ACS1,LYS9,MDH3,ISA1,UGA1,LYS1,MDH2,VTC1,IDP1,PXA2,ALD4,CAR1,CAR2,GLT1,PUT1,SPS19,TES1,gabD,ETR1,KLMA_60405,ARO9,ICL1,PHO84,ANT1,FOX2,ICL2,PDH1,CIT3,CRC1,ARO7
GO:0045116	protein neddylation	5	4	0.5	0.00044	UBC12,DCN1,ULA1,UBA3
GO:0043436	oxoacid metabolic process	303	48	30.11	0.00047	CHA1,GLY1,ECM31,CIT1,PXA1,POT1,DSD1,POX1,GPD2,AGX1,ARO10,IAH1,PEX11,GDH2,KLMA_30124,ACO2,DPL1,GAD1,ACS1,LYS9,MDH3,ISA1,UGA1,LYS1,MDH2,VTC1,IDP1,PXA2,ALD4,CAR1,CAR2,GLT1,PUT1,SPS19,TES1,gabD,ETR1,KLMA_60405,ARO9,ICL1,PHO84,ANT1,FOX2,ICL2,PDH1,CIT3,CRC1,ARO7
GO:0051704	multi-organism process	127	25	12.62	0.00053	KAR4,PRM1,ATG26,DIT2,STE3,MAK32,FUS3,SPS4,KLMA_20368,STE4,PRR1,KLMA_40061,CHS1,SPO14,GAS4,QDR3,FIG1,AXL1,SPS19,GPA1,MF(ALPHA)1,SST2,CRR1,KLMA_70118,SNF8
GO:1901606	alpha-amino acid catabolic process	21	8	2.09	0.00056	CHA1,ARO10,GDH2,GAD1,CAR1,CAR2,PUT1,gabD

GO.ID	Term	Annotated gene	Significant	Expected	P-value	Genes
GO:0006099	tricarboxylic acid cycle	22	8	2.19	0.0008	CIT1,KLMA_30124,ACO2,MDH3,MDH2,IDP1,KLMA_60405,CIT3
GO:0006101	citrate metabolic process	22	8	2.19	0.0008	CIT1,KLMA_30124,ACO2,MDH3,MDH2,IDP1,KLMA_60405,CIT3
GO:0072350	tricarboxylic acid metabolic process	22	8	2.19	0.0008	CIT1,KLMA_30124,ACO2,MDH3,MDH2,IDP1,KLMA_60405,CIT3
GO:0019752	carboxylic acid metabolic process	294	46	29.22	0.00083	CHA1,GLY1,ECM31,CIT1,PXA1,POT1,DSD1,POX1,GPD2,AGX1,ARO10,IAH1,PEX11,GDH2,KLMA_30124,ACO2,DPL1,GAD1,ACS1,LYS9,MDH3,ISA1,UGA1,LYS1,MDH2,IDP1,PXA2,ALD4,CAR1,CAR2,GLT1,PUT1,SPS19,TES1,gabD,ETR1,KLMA_60405,ARO9,ICL1,ANT1,FOX2,ICL2,PDH1,CIT3,CRC1,ARO7
GO:0006538	glutamate catabolic process	3	3	0.3	0.00097	GDH2,GAD1,gabD
GO:0019541	propionate metabolic process	3	3	0.3	0.00097	ICL2,PDH1,CIT3
GO:0019543	propionate catabolic process	3	3	0.3	0.00097	ICL2,PDH1,CIT3
GO:0019626	short-chain fatty acid catabolic process	3	3	0.3	0.00097	ICL2,PDH1,CIT3
GO:0019629	propionate catabolic process, 2-methylci...	3	3	0.3	0.00097	ICL2,PDH1,CIT3
GO:0043649	dicarboxylic acid catabolic process	3	3	0.3	0.00097	GDH2,GAD1,gabD
GO:0017144	drug metabolic process	180	31	17.89	0.0013	PGU1,DAL3,CIT1,GSM1,DSD1,GPD2,AGX1,ARO10,IAH1,KLMA_30124,ACO2,ACS1,THI13,MDH3,ISA1,CHS1,MDH2,IDP1,THI4,ALD4,CAR2,SFA1,CTT1,URC1,gabD,KLMA_60405,ARO9,ICL2,PDH1,CIT3,ARO7
GO:0051321	meiotic cell cycle	150	27	14.91	0.00136	KAR4,ATG26,CDC7,DIT2,FUS3,DMC1,CSM1,IME4,RIM15,SPS4,KLMA_20368,UBI4,MUS81,MSC1,MLH1,MND1,MLH3,KLMA_40062,CHS1,SPO14,GAS4,HOP1,ECO1,QDR3,SPS19,RAD1,CRR1
GO:0009063	cellular amino acid catabolic process	29	9	2.88	0.0014	CHA1,ARO10,GDH2,GAD1,UGA1,CAR1,CAR2,PUT1,gabD
GO:0015718	monocarboxylic acid transport	10	5	0.99	0.00156	FMP43,PXA1,PXA2,PUT4,FMP37

GO.ID	Term	Annotated gene	Significant	Expected	P-value	Genes
GO:0042737	drug catabolic process	19	7	1.89	0.00157	PGU1,DAL3,ARO10,CTT1,ICL2,PDH1,CIT3
GO:0000746	conjugation	67	15	6.66	0.00186	KAR4,PRM1,STE3,FUS3,STE4,PRR1,KLMA_40061,SPO14,FIG1,AXL1,GPA1,MF(ALPHA)1,SST2,KLMA_70118,SNF8
GO:0000747	conjugation with cellular fusion	67	15	6.66	0.00186	KAR4,PRM1,STE3,FUS3,STE4,PRR1,KLMA_40061,SPO14,FIG1,AXL1,GPA1,MF(ALPHA)1,SST2,KLMA_70118,SNF8
GO:0140253	cell-cell fusion	67	15	6.66	0.00186	KAR4,PRM1,STE3,FUS3,STE4,PRR1,KLMA_40061,SPO14,FIG1,AXL1,GPA1,MF(ALPHA)1,SST2,KLMA_70118,SNF8
GO:0030435	sporulation resulting in formation of a ...	56	13	5.57	0.00262	ATG26,DIT2,FUS3,SPS4,KLMA_20368,OSW5,CTS2,CHS1,SPO14,GAS4,QDR3,SPS19,CRR1
GO:0017001	antibiotic catabolic process	7	4	0.7	0.00263	CTT1,ICL2,PDH1,CIT3
GO:0009056	catabolic process	484	66	48.1	0.00282	CHA1,ECM38,HRD1,PLB,URH1,ATG27,PGU1,KLMA_10605,PEP12,UBP16,PNG1,DAL3,YUH1,AIM25,MIOX5,KLMA_20057,PXA1,POT1,POX1,GPD2,RIM15,ARO10,GDH2,KLMA_30011,RNH1,SPO1,CUE5,VTI1,SNX4,GAD1,MDH3,GID8,UGA1,SPO14,VTC1,PXA2,GDE1,CAR1,POP4,KLMA_50156,CAR2,CTT1,PUT1,SPS19,GUD1,TES1,gabD,APC11,JLP1,PHO85,PDE1,OTU1,ATH1,KLMA_70233,ANT1,KLMA_70317,LPX1,FOX2,ICL2,PDH1,CIT3,SNF8,ATG20,SGA1,KLMA_80176,VAM3
GO:0044764	multi-organism cellular process	70	15	6.96	0.00294	KAR4,PRM1,STE3,FUS3,STE4,PRR1,KLMA_40061,SPO14,FIG1,AXL1,GPA1,MF(ALPHA)1,SST2,KLMA_70118,SNF8
GO:0044281	small molecule metabolic process	540	72	53.67	0.00323	CHA1,URH1,GLY1,ECM31,MIOX5,KLMA_20057,CIT1,GSM1,PXA1,POT1,DSD1,ERT1,POX1,GPD2,AGX1,ARO10,IAH1,PEX11,GDH2,RDS2,KLMA_30124,KLMA_30282,ACO2,DPL1,GAD1,ACS1,THI13,KLMA_40010,LYS9,MDH3,GID8,ISA1,KLMA_40133,UGA1,LYS1,MDH2,VTC1,IDP1,PXA2,THI4,ALD4,CAR1,DAK1,CAR2,GLT1,SFA1,INO1,PUT1,URC1,SPS19,GUD1,TES1,gabD,KLMA_60206,ETR1,PHO85,KLMA_60405,PDE1,ARO9,FBP1,ICL1,PHO84,ANT1,KLMA_70303,FAD1,FOX2,ICL2,PDH1,CIT3,KLMA_80176,CRC1,ARO7
GO:0022413	reproductive process in single-celled or...	71	15	7.06	0.0034	PRM1,ATG26,DIT2,STE3,FUS3,SPS4,KLMA_20368,CHS1,SPO14,GAS4,QDR3,FIG1,AXL1,SPS19,CRR1
GO:0046459	short-chain fatty acid	4	3	0.4	0.00361	ICL2,PDH1,CIT3

GO.ID	Term	Annotated gene	Significant	Expected	P-value	Genes
	metabolic process					
GO:0043934	sporulation	58	13	5.76	0.00365	ATG26,DIT2,FUS3,SPS4,KLMA_20368,OSW5,CTS2,CHS1,SPO14,GAS4,QDR3,SPS19,CRR1
GO:0019236	response to pheromone	39	10	3.88	0.00376	KAR4,STE3,FUS3,STE4,PRR1,KLMA_40061,GPA1,MF(ALPHA)1,SST2,KLMA_70118
GO:0071444	cellular response to pheromone	39	10	3.88	0.00376	KAR4,STE3,FUS3,STE4,PRR1,KLMA_40061,GPA1,MF(ALPHA)1,SST2,KLMA_70118
GO:0044248	cellular catabolic process	464	63	46.12	0.00397	CHA1,ECM38,HRD1,PLB,URH1,ATG27,PGU1,KLMA_10605,PEP12,UBP16,PNG1,DAL3,YUH1,AIM25,MIOX5,KLMA_20057,PXA1,POT1,POX1,GPD2,RIM15,ARO10,GDH2,KLMA_30011,RNH1,SPO1,CUE5,VTI1,SNX4,GAD1,MDH3,GID8,UGA1,VTC1,PXA2,GDE1,CAR1,POP4,KLMA_50156,CAR2,CTT1,PUT1,SPS19,GUD1,TES1,gabD,APC11,JLP1,PHO85,PDE1,OTU1,ATH1,KLMA_70233,ANT1,LPX1,FOX2,ICL2,PDH1,CIT3,SNF8,ATG20,SGA1,VAM3
GO:0048646	anatomical structure formation involved ...	59	13	5.86	0.00427	ATG26,DIT2,FUS3,SPS4,KLMA_20368,OSW5,CTS2,CHS1,SPO14,GAS4,QDR3,SPS19,CRR1
GO:1903046	meiotic cell cycle process	133	23	13.22	0.00515	ATG26,CDC7,DIT2,FUS3,DMC1,CSM1,SPS4,KLMA_20368,MUS81,MSC1,MLH1,MND1,MLH3,KLMA_40062,CHS1,SPO14,GAS4,HOP1,ECO1,QDR3,SPS19,RAD1,CRR1
GO:0000750	pheromone-dependent signal transduction ...	18	6	1.79	0.00607	STE3,FUS3,STE4,GPA1,MF(ALPHA)1,KLMA_70118
GO:0032005	signal transduction involved in positive...	18	6	1.79	0.00607	STE3,FUS3,STE4,GPA1,MF(ALPHA)1,KLMA_70118
GO:0009653	anatomical structure morphogenesis	83	16	8.25	0.00652	ATG26,DIT2,FUS3,SPS4,KLMA_20368,OSW5,CTS2,CHS1,SPO14,ELM1,GAS4,QDR3,FIG1,KLMA_50343,SPS19,CRR1
GO:0048856	anatomical structure development	83	16	8.25	0.00652	ATG26,DIT2,FUS3,SPS4,KLMA_20368,OSW5,CTS2,CHS1,SPO14,ELM1,GAS4,QDR3,FIG1,KLMA_50343,SPS19,CRR1
GO:0030154	cell differentiation	69	14	6.86	0.00671	MATALPHA1,ATG26,DIT2,FUS3,SPS4,KLMA_20368,OSW5,CTS2,CHS1,SPO14,GAS4,QDR3,SPS19,CRR1
GO:0031137	regulation of conjugation with cellular ...	43	10	4.27	0.00791	STE3,FUS3,STE4,PRR1,KLMA_40061,AXL1,GPA1,MF(ALPHA)1,SST2,KLMA_70118

GO.ID	Term	Annotated gene	Significant	Expected	P-value	Genes
GO:0046999	regulation of conjugation	43	10	4.27	0.00791	STE3,FUS3,STE4,PRR1,KLMA_40061,AXL1,GPA1,MF(ALPHA)1,SST2,KLMA_70118
GO:0048869	cellular developmental process	130	22	12.92	0.008	MATALPHA1,ATG26,DIT2,AIM25,FUS3,HST2,RIM15,SPS4,KLMA_20368,OSW5,CTS2,EST2,CHS1,SPO14,ELM1,GAS4,QDR3,FIG1,KLMA_50343,SPS19,CRR1,PHO84
GO:0000755	cytogamy	9	4	0.89	0.00805	PRM1,STE3,FIG1,AXL1
GO:0006874	cellular calcium ion homeostasis	9	4	0.89	0.00805	PMC1,GDT1,FRQ1,VCX1
GO:0042149	cellular response to glucose starvation	9	4	0.89	0.00805	RIM15,ENA5,ELM1,FBP1
GO:0030437	ascospore formation	50	11	4.97	0.00844	ATG26,DIT2,FUS3,SPS4,KLMA_20368,CHS1,SPO14,GAS4,QDR3,SPS19,CRR1
GO:0048468	cell development	50	11	4.97	0.00844	ATG26,DIT2,FUS3,SPS4,KLMA_20368,CHS1,SPO14,GAS4,QDR3,SPS19,CRR1
GO:0034293	sexual sporulation	51	11	5.07	0.00984	ATG26,DIT2,FUS3,SPS4,KLMA_20368,CHS1,SPO14,GAS4,QDR3,SPS19,CRR1
GO:0043935	sexual sporulation resulting in formatio...	51	11	5.07	0.00984	ATG26,DIT2,FUS3,SPS4,KLMA_20368,CHS1,SPO14,GAS4,QDR3,SPS19,CRR1
GO:0000437	carbon catabolite repression of transcri...	2	2	0.2	0.00985	ERT1,RDS2
GO:0006527	arginine catabolic process	2	2	0.2	0.00985	CAR1,CAR2
GO:0006850	mitochondrial pyruvate transmembrane tra...	2	2	0.2	0.00985	FMP43,FMP37
GO:0032220	plasma membrane fusion involved in cytog...	2	2	0.2	0.00985	PRM1,FIG1
GO:0042758	long-chain fatty acid catabolic process	2	2	0.2	0.00985	PXA1,PXA2
GO:0044375	regulation of peroxisome size	2	2	0.2	0.00985	PEX11,PEX25
GO:0045026	plasma membrane fusion	2	2	0.2	0.00985	PRM1,FIG1

GO.ID	Term	Annotated gene	Significant	Expected	P-value	Genes
GO:0061415	negative regulation of transcription fro...	2	2	0.2	0.00985	ERT1,RDS2
GO:0071466	cellular response to xenobiotic stimulus	2	2	0.2	0.00985	ECM38,RDS2
GO:0009123	nucleoside monophosphate metabolic proce...	86	22	8.65	2.30E-05	PRS5,GPM3,HPT1,GUA1,ENO,PGK,AAH1,RAG2,URA1,GPM1,ADK1,ADE2,PDC2,ADE5,7,TPI1,GAP1,FBA1,XPT1,ADE6,PYK1,RAG5,GAP3
GO:0000054	ribosomal subunit export from nucleus	41	14	4.13	2.30E-05	SDA1,NOG1,ARX1,NOP53,RPS3,RRS1,RLI1,MEX67,NMD3,RIX1,MTR2,NUG1,NOP9,LTV1
GO:0033750	ribosome localization	41	14	4.13	2.30E-05	SDA1,NOG1,ARX1,NOP53,RPS3,RRS1,RLI1,MEX67,NMD3,RIX1,MTR2,NUG1,NOP9,LTV1
GO:0009117	nucleotide metabolic process	152	32	15.29	2.70E-05	APA2,PRS5,GPM3,HPT1,GUA1,ENO,PGK,AAH1,RAG2,URA1,GPM1,ADH4,ADK1,ADE2,HAM1,RNR2,PDC2,ADE5,7,TPI1,GAP1,FBA1,HMG1,XPT1,GUK1,ADE6,ACS2,PYK1,RAG5,ERG13,URA7,RNR1,GAP3
GO:0009126	purine nucleoside monophosphate metaboli...	75	20	7.55	2.90E-05	GPM3,HPT1,GUA1,ENO,PGK,AAH1,RAG2,GPM1,ADK1,ADE2,PDC2,ADE5,7,TPI1,GAP1,FBA1,XPT1,ADE6,PYK1,RAG5,GAP3
GO:0009167	purine ribonucleoside monophosphate meta...	75	20	7.55	2.90E-05	GPM3,HPT1,GUA1,ENO,PGK,AAH1,RAG2,GPM1,ADK1,ADE2,PDC2,ADE5,7,TPI1,GAP1,FBA1,XPT1,ADE6,PYK1,RAG5,GAP3
GO:0005996	monosaccharide metabolic process	47	15	4.73	3.00E-05	GAL80,PGK,RAG2,GPM1,GAL7,GAL10,GAL1,PDC2,TPI1,GAP1,FBA1,PYK1,RGT1,GAP3,VID24
GO:0072525	pyridine-containing compound biosyntheti...	32	12	3.22	3.10E-05	GPM3,ENO,PGK,RAG2,GPM1,TPI1,GAP1,FBA1,PDX3,PYK1,RAG5,GAP3
GO:0090305	nucleic acid phosphodiester bond hydroly...	100	24	10.06	3.10E-05	UTP13,UTP11,NOP58,ENP1,RLP7,MPP10,RRS1,NOB1,PWP2,DBP3,UTP10,NOP19,BMS1,UTP6,MTR4,UTP18,RRP5,DIP2,BRX1,FCF2,NOP14,NOC4,NOP9,RRP42
GO:0006753	nucleoside phosphate metabolic process	153	32	15.39	3.10E-05	APA2,PRS5,GPM3,HPT1,GUA1,ENO,PGK,AAH1,RAG2,URA1,GPM1,ADH4,ADK1,ADE2,HAM1,RNR2,PDC2,ADE5,7,TPI1,GAP1,FBA1,HMG1,XPT1,GUK1,ADE6,ACS2,PYK1,RAG5,ERG13,URA7,RNR1,GAP3
GO:0016070	RNA metabolic process	843	116	84.82	3.60E-05	SPB1,RPA49,NOP1,SOH1,DSS1,UTP5,EBP2,RPF2,RSE1,GAL80,MES1,HAS1,YTA7,MSW1,NOG1,UTP13,RPC37,DBP7,UTP15,UTP11,NOP53,NOP58,FHL1,NOP12,ENP1,CBF5,KLMA_20481,

GO.ID	Term	Annotated gene	Significant	Expected	P-value	Genes
						RLP7,UTP8,TPA1,MPP10,IPI3,NAN1,MRT4,RRS1,IPI1,DCAF13,DRS1,NOB1,RPL3,PAB1,MRPL15,PDC2,BFR2,PWP2,DBP3,RRP3,HAL9,VTS1,KSS1,MTO1,GUS1,ECM16,MDN1,UTP10,GAR1,CIC1,HAP1,ERB1,MCM1,RRP12,IMP4,YTM1,NOP19,RPC82,RIX1,UTP21,PUS1,BMS1,UTP6,PRP43,GEP3,MTR4,UTP18,ENP2,RPA135,RRP5,PWP1,NOP56,LAC9,UTP4,NUG1,RPC40,VAS1,NOP7,DIP2,IMP3,BRX1,URB1,DHR2,RPA190,FUN12,YAF9,NOP15,KRE33,EPL1,FCF2,KLMA_60313,NOP4,JHD2,CDC60,IKI3,ROX3,SPT21,NOP14,NOC4,RRP9,PUS4,KLMA_70408,NOP9,RRP42,PUB1,UTP25,HDA1,NOP2,ILS1
GO:0009058	biosynthetic process	1301	165	130.9	3.80E-05	HIS4,SAM2,RpL37a,RPA49,PRS5,GPM3,NOP1,OLE1,SOH1,UTP5,HPT1,GUA1,BIO2,RPL17B,GAL80,ENO,MES1,YTA7,LIP1,FAU1,PGK,CWH43,SCS7,MSW1,FUR1,AAH1,RAG2,URA1,TPS2,KLMA_20052,TSL1,GPM1,RPC37,UTP15,ADH4,UTP11,ADK1,RPS2,MET13,CYS4,SHM2,ERG3,FHL1,KLMA_20355,KLMA_20481,RPL22A,UTP8,TPA1,ERG28,CYP707A7,ORT1,ADE2,ERG9,IPI3,RPS3,RPL5,NAN1,LIA1,HAM1,GSH1,PHS1,IPI1,RNR2,ERG1,MEU1,ETT1,RPL3,PAB1,RLI1,KLMA_30320,GSY2,PDC2,SUR2,SER3,YIH1,PAN5,TIF32,HAL9,KSS1,ERG6,SAH1,ADE5,7,GUS1,RPL15B,dsd1,HEM13,TPI1,GAP1,RIB7,UTP10,HAP1,MCM1,FMS1,FBA1,ALD5,MAM33,HMG1,DPM1,RPC82,MET5,RIX1,XPT1,FAS1,RPL10A,GUK1,SHM1,LEU1,RPA135,PDX3,RPS14,SUR4,ADE6,ERG25,MSS51,LAC9,UTP4,RPC40,VAS1,NOP7,RPL2,ACS2,SHB17,URA2,KLMA_60069,PDC1,RPA190,FUN12,REV1,PYK1,LEU4,YAF9,EPL1,KLMA_60313,UGP1,RAG5,cyp524A1,JHD2,LEU3,ERG13,RPB1,CDC60,PET309,IKI3,URA7,ROX3,RNR1,FAS2,SPT21,TIF3,MET16,HEM14,RPL19B,DYS1,KLMA_70408,GAP3,MET3,MNN1,HDA1,NIP1,KLMA_80256,VID24,TPS1,MET6,ILS1
GO:0071826	ribonucleoprotein complex subunit organi...	115	26	11.57	4.50E-05	RPF2,RSE1,NOG1,NOP53,IPI3,RPL5,MRT4,IPI1,DRS1,RPL3,RLI1,TIF32,MDN1,RIX1,PRP43,RRP5,KLMA_60069,BRX1,FUN12,RSA4,TIF3,MAK21,RLP24,PUB1,NOP2,NIP1

GO.ID	Term	Annotated gene	Significant	Expected	P-value	Genes
GO:1901576	organic substance biosynthetic process	1289	163	129.69	5.50E-05	HIS4,SAM2,RpL37a,RPA49,PRS5,GPM3,NOP1,OLE1,SOH1,UTP5,HPT1,GUA1,BIO2,RPL17B,GAL80,ENO,MES1,YTA7,LIP1,FAU1,PGK,CWH43,SCS7,MSW1,FUR1,AAH1,RAG2,URA1,TPS2,KLMA_20052,TSL1,GPM1,RPC37,UTP15,ADH4,UTP11,ADK1,RPS2,MET13,CYS4,SHM2,ERG3,FHL1,KLMA_20355,KLMA_20481,RPL22A,UTP8,TPA1,ERG28,CYP707A7,ORT1,ADE2,ERG9,IPI3,RPS3,RPL5,NAN1,HAM1,GSH1,PHS1,IPI1,RNR2,ERG1,MEU1,ETT1,RPL3,PAB1,RLI1,KLMA_30320,GSY2,PDC2,SUR2,SER3,YIH1,PAN5,TIF32,HAL9,KSS1,ERG6,SAH1,ADE5,7,GUS1,RPL15B,dsd1,HEM13,TPI1,GAP1,RIB7,UTP10,HAP1,MCM1,FMS1,FBA1,ALD5,MAM33,HMG1,DPM1,RPC82,MET5,RIX1,XPT1,FAS1,RPL10A,GUK1,SHM1,LEU1,RPA135,PDX3,RPS14,SUR4,ADE6,ERG25,MSS51,LAC9,UTP4,RPC40,VAS1,NOP7,RPL2,ACS2,SHB17,URA2,KLMA_60069,PDC1,RPA190,FUN12,REV1,PYK1,LEU4,YAF9,EPL1,KLMA_60313,UGP1,RAG5,cyp524A1,JHD2,LEU3,ERG13,RPB1,CDC60,PET309,IKI3,URA7,ROX3,RNR1,FAS2,SPT21,TIF3,MET16,HEM14,RPL19B,KLMA_70408,GAP3,MET3,MNN1,HDA1,NIP1,KLMA_80256,VID24,TPS1,MET6,ILS1
GO:0009150	purine ribonucleotide metabolic process	97	23	9.76	5.70E-05	GPM3,HPT1,GUA1,ENO,PGK,RAG2,GPM1,ADK1,ADE2,PDC2,ADE5,7,TPI1,GAP1,FBA1,HMG1,XPT1,GUK1,ADE6,ACS2,PYK1,RAG5,ERG13,GAP3
GO:0009116	nucleoside metabolic process	34	12	3.42	6.30E-05	PRS5,HPT1,GUA1,FUR1,URA1,RNR2,MEU1,SAH1,XPT1,GUK1,URA7,RNR1
GO:0006163	purine nucleotide metabolic process	98	23	9.86	6.70E-05	GPM3,HPT1,GUA1,ENO,PGK,RAG2,GPM1,ADK1,ADE2,PDC2,ADE5,7,TPI1,GAP1,FBA1,HMG1,XPT1,GUK1,ADE6,ACS2,PYK1,RAG5,ERG13,GAP3
GO:0009142	nucleoside triphosphate biosynthetic pro...	40	13	4.02	8.30E-05	GPM3,ENO,PGK,RAG2,GPM1,TPI1,GAP1,FBA1,PYK1,RAG5,URA7,RNR1,GAP3
GO:1901657	glycosyl compound metabolic process	35	12	3.52	8.70E-05	PRS5,HPT1,GUA1,FUR1,URA1,RNR2,MEU1,SAH1,XPT1,GUK1,URA7,RNR1
GO:0005992	trehalose biosynthetic process	4	4	0.4	0.0001	TPS2,TSL1,UGP1,TPS1
GO:0009312	oligosaccharide biosynthetic process	4	4	0.4	0.0001	TPS2,TSL1,UGP1,TPS1

GO.ID	Term	Annotated gene	Significant	Expected	P-value	Genes
GO:0046351	disaccharide biosynthetic process	4	4	0.4	0.0001	TPS2,TSL1,UGP1,TPS1
GO:0044249	cellular biosynthetic process	1278	160	128.58	0.00013	HIS4,SAM2,RpL37a,RPA49,PRS5,GPM3,NOP1,OLE1,SOH1,UTP5,HPT1,GUA1,BIO2,RPL17B,GAL80,ENO,MES1,YTA7,LIP1,FAU1,PGK,CWH43,SCS7,MSW1,FUR1,AAH1,RAG2,URA1,TPS2,KLMA_20052,TSL1,GPM1,RPC37,UTP15,UTP11,ADK1,RPS2,MET13,CYS4,SHM2,ERG3,FHL1,KLMA_20355,KLMA_20481,RPL22A,UTP8,TPA1,ERG28,CYP707A7,ORT1,ADE2,ERG9,IPI3,RPS3,RPL5,NAN1,HAM1,GSH1,PHS1,IPI1,RNR2,ERG1,MEU1,ETT1,RPL3,PAB1,RLI1,KLMA_30320,GSY2,PDC2,SUR2,SER3,YIH1,PAN5,TIF32,HAL9,KSS1,ERG6,SAH1,ADE5,7,GUS1,RPL15B,dsd1,HEM13,TPI1,GAP1,RIB7,UTP10,HAP1,MCM1,FMS1,FBA1,ALD5,MAM33,HMG1,DPM1,RPC82,MET5,RIX1,XPT1,FAS1,RPL10A,GUK1,SHM1,LEU1,RPA135,PDX3,RPS14,SUR4,ADE6,ERG25,MSS51,LAC9,UTP4,RPC40,VAS1,NOP7,RPL2,ACS2,URA2,KLMA_60069,RPA190,FUN12,REV1,PYK1,LEU4,YAF9,EPL1,KLMA_60313,UGP1,RAG5,cyp524A1,JHD2,LEU3,ERG13,RPB1,CDC60,PET309,IKI3,URA7,ROX3,RNR1,FAS2,SPT21,TIF3,MET16,HEM14,RPL19B,KLMA_70408,GAP3,MET3,MNN1,HDA1,NIP1,KLMA_80256,VID24,TPS1,MET6,ILS1
GO:0006807	nitrogen compound metabolic process	1991	231	200.32	0.00013	SPB1,APA2,HIS4,SAM2,RpL37a,RPA49,PRS5,GPM3,NOP1,SOH1,DSS1,UTP5,HPT1,GUA1,BIO2,RPL17B,EBP2,RPF2,RSE1,GAL80,ENO,MES1,HAS1,TDA1,YTA7,LIP1,FAU1,PGK,CWH43,SCS7,MSW1,FUR1,AAH1,RAG2,NOG1,URA1,KLMA_20052,GPM1,UTP13,RPC37,DBP7,UTP15,HSL1,ADH4,UTP11,ADK1,NOP53,RPS2,MET13,CYS4,SHM2,NOP58,FHL1,KLMA_20355,NOP12,ENP1,CLN2,UBP3,CBF5,KLMA_20481,RLP7,RPL22A,UTP8,TPA1,ORT1,ADE2,MPP10,DTD1,IPI3,RPS3,RPL5,NAN1,LIA1,HAM1,MRT4,RRS1,GSH1,PHS1,IPI1,RNR2,DCAF13,MEU1,DRS1,ETT1,NOB1,RPL3,PAB1,RLI1,MRPL15,KLMA_30320,PDC2,SUR2,BFR2,SER3,PWP2,YIH1,DBP3,PAN5,RRP3,TIF32,HAL9,VTS1,RAD54,KSS1,SAH1,DUG1,ADE5,7,MTO1,GUS1,ECM16,RPL15B,dsd1,HEM13,TPI1,MDN1,GAP1,RIB7,UTP10,GAR1,CIC1,HAP1,ERB1,MCM1,IMP2,RRP12,IMP4,FMS1,FBA1,MAM33,YTM1,NOP19,HMG1,DPM1,RPC82,MET5,RIX1,XPT1,UTP21,PUS1,BMS1,RPL10A,GUK1,UTP6,PRP43,GEP3,SHM1,MTR4,UTP18,ENP2,LEU1,RPA135,PDX3,RPS14,ADE6,RRP5,PWP1,N

GO.ID	Term	Annotated gene	Significant	Expected	P-value	Genes
						OP56,MSS51,LAC9,UTP4,NUG1,RPC40,VAS1,NOP7,RPL2,DIP2,ACS2,MAP1,URA2,IMP3,KLMA_60069,PDC1,EXO1,MDM20,BRX1,URB1,DHR2,RPA190,FUN12,REV1,PYK1,LEU4,YAF9,NOP15,KRE33,EPL1,FCF2,KLMA_60313,UGP1,NOP4,RAG5,JHD2,LEU3,ERG13,RPB1,CDC60,PET309,IKI3,URA7,ROX3,RNR1,PCL6,HSL7,SPT21,NOP14,TIF3,MET16,NOC4,RRP9,HEM14,RPL19B,DYS1,PUS4,KLMA_70408,NOP9,GAP3,MET3,MNN1,RRP42,PUB1,UTP25,HDA1,NOP2,NIP1,KLMA_80256,DEF1,VID24,PTH2,MET6,ILS1
GO:0022618	ribonucleoprotein complex assembly	109	24	10.97	0.00014	RPF2,RSE1,NOG1,NOP53,IPI3,RPL5,MRT4,IPI1,DRS1,RPL3,TIF32,MDN1,RIX1,RRP5,KLMA_60069,BRX1,FUN12,RSA4,TIF3,MAK21,RLP24,PUB1,NOP2,NIP1
GO:0005975	carbohydrate metabolic process	137	28	13.78	0.00015	GPD1,GPM3,GAL80,ENO,PGK,RAG2,TPS2,TSL1,GPM1,GAL7,GAL10,GAL1,PGM2,GSY2,PDC2,SCW4,TPI1,GAP1,FBA1,MDH1,PYK1,RGT1,UGP1,RAG5,GAP3,BGL2,VID24,TPS1
GO:0009201	ribonucleoside triphosphate biosynthetic...	37	12	3.72	0.00016	GPM3,ENO,PGK,RAG2,GPM1,TPI1,GAP1,FBA1,PYK1,RAG5,URA7,GAP3
GO:0071166	ribonucleoprotein complex localization	84	20	8.45	0.00016	SDA1,NOG1,ARX1,NOP53,RPS2,ENP1,UTP8,RPS3,RRS1,RLI1,MEX67,NMD3,RIX1,MTR2,MTR4,NUG1,CEX1,NEW1,NOP9,LTV1
GO:0071426	ribonucleoprotein complex export from nu...	84	20	8.45	0.00016	SDA1,NOG1,ARX1,NOP53,RPS2,ENP1,UTP8,RPS3,RRS1,RLI1,MEX67,NMD3,RIX1,MTR2,MTR4,NUG1,CEX1,NEW1,NOP9,LTV1
GO:0006006	glucose metabolic process	32	11	3.22	0.00017	PGK,RAG2,GPM1,PDC2,TPI1,GAP1,FBA1,PYK1,RGT1,GAP3,VID24
GO:0019362	pyridine nucleotide metabolic process	43	13	4.33	0.00019	GPM3,ENO,PGK,RAG2,GPM1,ADH4,PDC2,TPI1,GAP1,FBA1,PYK1,RAG5,GAP3
GO:0046496	nicotinamide nucleotide metabolic proces...	43	13	4.33	0.00019	GPM3,ENO,PGK,RAG2,GPM1,ADH4,PDC2,TPI1,GAP1,FBA1,PYK1,RAG5,GAP3
GO:0006405	RNA export from nucleus	85	20	8.55	0.00019	SDA1,NOG1,ARX1,NOP53,RPS2,ENP1,UTP8,RPS3,RRS1,RLI1,MEX67,NMD3,RIX1,MTR2,MTR4,NUG1,CEX1,NEW1,NOP9,LTV1
GO:0032787	monocarboxylic acid metabolic process	105	23	10.56	0.00021	GPM3,OLE1,BIO2,ENO,PGK,SCS7,RAG2,GPM1,PHS1,PDC2,PAN5,TPI1,GAP1,FMS1,FBA1,ALD5,FAS1,SUR4,ACS2,PYK1,RAG5,FAS2,GAP3

GO.ID	Term	Annotated gene	Significant	Expected	P-value	Genes
GO:0072524	pyridine-containing compound metabolic p...	49	14	4.93	0.00021	GPM3,ENO,PGK,RAG2,GPM1,ADH4,PDC2,TPI1,GAP1,FBA1,PDX3,PYK1,RAG5,GAP3
GO:0009119	ribonucleoside metabolic process	23	9	2.31	0.00022	HPT1,GUA1,URA1,RNR2,MEU1,SAH1,GUK1,URA7,RNR1
GO:0046165	alcohol biosynthetic process	39	12	3.92	0.00028	ADH4,ERG3,ERG28,CYP707A7,ERG9,ERG1,ERG6,dsd1,ERG25,PDC1,cyp524A1,ERG13
GO:0090407	organophosphate biosynthetic process	178	33	17.91	0.00028	PRS5,GPM3,HPT1,GUA1,ENO,PGK,CWH43,AAH1,RAG2,URA1,KLMA_20052,GPM1,ADK1,ADE2,RNR2,SAH1,ADE5,7,TPI1,GAP1,FBA1,HMG1,DPM1,XPT1,GUK1,ADE6,ACS2,SHB17,PYK1,RAG5,ERG13,URA7,RNR1,GAP3
GO:0006732	coenzyme metabolic process	114	24	11.47	0.00029	SAM2,GPM3,BIO2,ENO,FAU1,PGK,RAG2,GPM1,ADH4,MET13,SHM2,PDC2,PAN5,TPI1,GAP1,FMS1,FBA1,HMG1,SHM1,ACS2,PYK1,RAG5,ERG13,GAP3
GO:0006754	ATP biosynthetic process	34	11	3.42	0.00031	GPM3,ENO,PGK,RAG2,GPM1,TPI1,GAP1,FBA1,PYK1,RAG5,GAP3
GO:0051188	cofactor biosynthetic process	101	22	10.16	0.00031	SAM2,GPM3,BIO2,ENO,FAU1,PGK,RAG2,GPM1,GSH1,PAN5,HEM13,TPI1,GAP1,FMS1,FBA1,PDX3,ACS2,PYK1,RAG5,MET16,HEM14,GAP3
GO:0006696	ergosterol biosynthetic process	24	9	2.41	0.00031	ERG3,ERG28,CYP707A7,ERG9,ERG1,ERG6,ERG25,cyp524A1,ERG13
GO:0008204	ergosterol metabolic process	24	9	2.41	0.00031	ERG3,ERG28,CYP707A7,ERG9,ERG1,ERG6,ERG25,cyp524A1,ERG13
GO:0016128	phytosteroid metabolic process	24	9	2.41	0.00031	ERG3,ERG28,CYP707A7,ERG9,ERG1,ERG6,ERG25,cyp524A1,ERG13
GO:0016129	phytosteroid biosynthetic process	24	9	2.41	0.00031	ERG3,ERG28,CYP707A7,ERG9,ERG1,ERG6,ERG25,cyp524A1,ERG13
GO:0044108	cellular alcohol biosynthetic process	24	9	2.41	0.00031	ERG3,ERG28,CYP707A7,ERG9,ERG1,ERG6,ERG25,cyp524A1,ERG13
GO:0097384	cellular lipid biosynthetic process	24	9	2.41	0.00031	ERG3,ERG28,CYP707A7,ERG9,ERG1,ERG6,ERG25,cyp524A1,ERG13
GO:0006611	protein export from nucleus	89	20	8.95	0.00038	SDA1,NOG1,ARX1,NOP53,RPS2,ENP1,UTP8,RPS3,RRS1,RLI1,MEX67,NMD3,RIX1,MTR2,MTR4,NUG1,CEX1,NEW1,NOP9,LTV1

GO.ID	Term	Annotated gene	Significant	Expected	P-value	Genes
GO:0009145	purine nucleoside triphosphate biosynthe...	35	11	3.52	0.00041	GPM3,ENO,PGK,RAG2,GPM1,TPI1,GAP1,FBA1,PYK1,RAG5,GAP3
GO:0009206	purine ribonucleoside triphosphate biosy...	35	11	3.52	0.00041	GPM3,ENO,PGK,RAG2,GPM1,TPI1,GAP1,FBA1,PYK1,RAG5,GAP3
GO:0051168	nuclear export	90	20	9.06	0.00044	SDA1,NOG1,ARX1,NOP53,RPS2,ENP1,UTP8,RPS3,RRS1,RLI1,MEX67,NMD3,RIX1,MTR2,MTR4,NUG1,CEX1,NEW1,NOP9,LTV1
GO:1902652	secondary alcohol metabolic process	25	9	2.52	0.00045	ERG3,ERG28,CYP707A7,ERG9,ERG1,ERG6,ERG25,cyp524A1,ERG13
GO:1902653	secondary alcohol biosynthetic process	25	9	2.52	0.00045	ERG3,ERG28,CYP707A7,ERG9,ERG1,ERG6,ERG25,cyp524A1,ERG13
GO:0006012	galactose metabolic process	5	4	0.5	0.00046	GAL80,GAL7,GAL10,GAL1
GO:0051186	cofactor metabolic process	169	31	17	0.00053	SAM2,GPM3,BIO2,ENO,FAU1,PGK,RAG2,GPM1,ADH4,MET13,SHM2,GSH1,PDC2,PAN5,SAH1,DUG1,HEM13,TPI1,GAP1,FMS1,FBA1,HMG1,SHM1,PDX3,ACS2,PYK1,RAG5,ERG13,MET16,HEM14,GAP3
GO:1901137	carbohydrate derivative biosynthetic pro...	177	32	17.81	0.00056	PRS5,GPM3,HPT1,GUA1,ENO,PGK,CWH43,RAG2,URA1,GPM1,ADK1,ADE2,RNR2,MEU1,KLMA_30320,ADE5,7,TPI1,GAP1,FBA1,DPM1,XPT1,GUK1,ADE6,ACS2,SHB17,PYK1,RAG5,URA7,RNR1,GAP3,MNN1
GO:0044107	cellular alcohol metabolic process	26	9	2.62	0.00063	ERG3,ERG28,CYP707A7,ERG9,ERG1,ERG6,ERG25,cyp524A1,ERG13
GO:0050657	nucleic acid transport	94	20	9.46	0.0008	SDA1,NOG1,ARX1,NOP53,RPS2,ENP1,UTP8,RPS3,RRS1,RLI1,MEX67,NMD3,RIX1,MTR2,MTR4,NUG1,CEX1,NEW1,NOP9,LTV1
GO:0050658	RNA transport	94	20	9.46	0.0008	SDA1,NOG1,ARX1,NOP53,RPS2,ENP1,UTP8,RPS3,RRS1,RLI1,MEX67,NMD3,RIX1,MTR2,MTR4,NUG1,CEX1,NEW1,NOP9,LTV1
GO:0051236	establishment of RNA localization	94	20	9.46	0.0008	SDA1,NOG1,ARX1,NOP53,RPS2,ENP1,UTP8,RPS3,RRS1,RLI1,MEX67,NMD3,RIX1,MTR2,MTR4,NUG1,CEX1,NEW1,NOP9,LTV1

GO.ID	Term	Annotated gene	Significant	Expected	P-value	Genes
GO:1901135	carbohydrate derivative metabolic proces...	260	42	26.16	0.00094	PRS5,GPD1,GPM3,HPT1,GUA1,PFK1,ENO,PGK,CWH43,FUR1,RAG2,URA1,GPM1,ADK1,ADE2,RNR2,MEU1,KLMA_30320,PDC2,SUR2,SAH1,ADE5,7,TPI1,GAP1,FBA1,HMG1,DPM1,XPT1,GUK1,ADE6,ACS2,SHB17,PYK1,UGP1,RAG5,ERG13,PFK2,URA7,RNR1,GAP3,MNN1
GO:1901617	organic hydroxy compound biosynthetic pr...	50	13	5.03	0.00096	ADH4,ERG3,ERG28,CYP707A7,ERG9,ERG1,ERG6,dsd1,PDX3,ERG25,PDC1,cyp524A1,ERG13
GO:0009141	nucleoside triphosphate metabolic proces...	69	16	6.94	0.00101	GPM3,ENO,PGK,RAG2,GPM1,ADK1,HAM1,PDC2,TPI1,GAP1,FBA1,PYK1,RAG5,URA7,RNR1,GAP3
GO:0000464	endonucleolytic cleavage in ITS1 upstrea...	3	3	0.3	0.00101	DBP3,RRP5,BRX1
GO:0019637	organophosphate metabolic process	270	43	27.17	0.0011	APA2,LPP1,PRS5,GPD1,GPM3,HPT1,GUA1,PFK1,ENO,PGK,CWH43,SCS7,AAH1,RAG2,URA1,KLMA_20052,GPM1,ADH4,ADK1,ADE2,HAM1,RNR2,PDC2,SUR2,SAH1,ADE5,7,TPI1,GAP1,FBA1,HMG1,DPM1,XPT1,GUK1,ADE6,ACS2,SHB17,PYK1,RAG5,ERG13,PFK2,URA7,RNR1,GAP3
GO:0009133	nucleoside diphosphate biosynthetic proc...	6	4	0.6	0.00128	ADK1,RNR2,GUK1,RNR1
GO:0006066	alcohol metabolic process	52	13	5.23	0.00143	ADH4,ERG3,ERG28,CYP707A7,ERG9,ERG1,PDC2,ERG6,dsd1,ERG25,PDC1,cyp524A1,ERG13
GO:0009163	nucleoside biosynthetic process	14	6	1.41	0.00148	HPT1,GUA1,URA1,MEU1,GUK1,URA7
GO:0042455	ribonucleoside biosynthetic process	14	6	1.41	0.00148	HPT1,GUA1,URA1,MEU1,GUK1,URA7
GO:1901659	glycosyl compound biosynthetic process	14	6	1.41	0.00148	HPT1,GUA1,URA1,MEU1,GUK1,URA7
GO:0006694	steroid biosynthetic process	29	9	2.92	0.00153	ERG3,ERG28,CYP707A7,ERG9,ERG1,ERG6,ERG25,cyp524A1,ERG13
GO:0016126	sterol biosynthetic process	29	9	2.92	0.00153	ERG3,ERG28,CYP707A7,ERG9,ERG1,ERG6,ERG25,cyp524A1,ERG13
GO:0000055	ribosomal large subunit export from	24	8	2.41	0.00167	SDA1,ARX1,NOP53,RRS1,MEX67,NMD3,MTR2,NUG1

GO.ID	Term	Annotated gene	Significant	Expected	P-value	Genes
	nucl...					
GO:0044272	sulfur compound biosynthetic process	47	12	4.73	0.00178	SAM2,BIO2,MET13,CYS4,GSH1,MEU1,PDC2,MET5,ACS2,MET16,MET3,MET6
GO:0019439	aromatic compound catabolic process	136	25	13.68	0.00178	GPM3,DSS1,ENO,PGK,AAH1,RAG2,GPM1,TPA1,DTD1,HAM1,MRT4,PAB1,VTS1,SAH1,TPI1,GAP1,FBA1,MTR4,PDC1,PYK1,RAG5,JHD2,GAP3,RRP42,PUB1
GO:0009108	coenzyme biosynthetic process	81	17	8.15	0.00229	SAM2,GPM3,BIO2,ENO,FAU1,PGK,RAG2,GPM1,PAN5,TPI1,GAP1,FMS1,FBA1,ACS2,PYK1,RAG5,GAP3
GO:0043603	cellular amide metabolic process	347	51	34.91	0.00243	RpL37a,BIO2,RPL17B,MES1,LIP1,FAU1,MSW1,UTP11,RPS2,MET13,SHM2,KLMA_20355,RPL22A,TPA1,RPS3,RPL5,GSH1,ETT1,RPL3,PAB1,RLI1,YIH1,PAN5,TIF32,DUG1,GUS1,RPL15B,IMP2,FMS1,MAM33,HMG1,RPL10A,SHM1,RPS14,MSS51,VAS1,RPL2,ACS2,KLMA_60069,FUN12,KLMA_60313,ERG13,CDC60,PET309,IKI3,TIF3,MET16,RPL19B,NIP1,KLMA_80256,ILS1
GO:0016125	sterol metabolic process	31	9	3.12	0.00257	ERG3,ERG28,CYP707A7,ERG9,ERG1,ERG6,ERG25,cyp524A1,ERG13
GO:1902626	assembly of large subunit precursor of p...	7	4	0.7	0.00275	RPF2,NOG1,RLP24,NOP2
GO:0031503	protein-containing complex localization	104	20	10.46	0.00294	SDA1,NOG1,ARX1,NOP53,RPS2,ENP1,UTP8,RPS3,RRS1,RLI1,MEX67,NMD3,RIX1,MTR2,MTR4,NUG1,CEX1,NEW1,NOP9,LTV1
GO:0009112	nucleobase metabolic process	26	8	2.62	0.00297	AAH1,URA1,ADE2,ADE5,7,XPT1,URA2,URA7,MET6
GO:0017144	drug metabolic process	180	30	18.11	0.00318	SAM2,ACO2,GPM3,BIO2,ENO,PGK,RAG2,GPM1,ADK1,CYS4,SHM2,MEU1,PDC2,TPI1,GAP1,RIB7,FMS1,FBA1,ALD5,MET5,SHM1,PDX3,ACS2,PDC1,MDH1,PYK1,RAG5,GAP3,MET3,MET6
GO:0008202	steroid metabolic process	32	9	3.22	0.00326	ERG3,ERG28,CYP707A7,ERG9,ERG1,ERG6,ERG25,cyp524A1,ERG13
GO:0006520	cellular amino acid metabolic process	173	29	17.41	0.00341	HIS4,SAM2,GUA1,MES1,MSW1,ADH4,MET13,CYS4,SHM2,ORT1,DTD1,MEU1,SER3,SAH1,GUS1,MET5,SHM1,LEU1,ADE6,VAS1,URA2,PDC1,LEU4,LEU3,CDC60,URA7,MET3,MET6,ILS1

GO.ID	Term	Annotated gene	Significant	Expected	P-value	Genes
GO:0006733	oxidoreduction coenzyme metabolic proces...	57	13	5.73	0.00346	GPM3,ENO,PGK,RAG2,GPM1,ADH4,PDC2,TPI1,GAP1,FBA1,PYK1,RAG5,GAP3
GO:1901605	alpha-amino acid metabolic process	113	21	11.37	0.00358	HIS4,SAM2,GUA1,MET13,CYS4,SHM2,ORT1,DTD1,MEU1,SER3,SAH1,SHM1,LEU1,ADE6,URA2,PDC1,LEU4,LEU3,URA7,MET3,MET6
GO:1901361	organic cyclic compound catabolic proces...	143	25	14.39	0.00364	GPM3,DSS1,ENO,PGK,AAH1,RAG2,GPM1,TPA1,DTD1,HAM1,MRT4,PAB1,VTS1,SAH1,TPI1,GAP1,FBA1,MTR4,PDC1,PYK1,RAG5,JHD2,GAP3,RRP42,PUB1
GO:0009199	ribonucleoside triphosphate metabolic pr...	64	14	6.44	0.00368	GPM3,ENO,PGK,RAG2,GPM1,ADK1,PDC2,TPI1,GAP1,FBA1,PYK1,RAG5,URA7,GAP3
GO:0009403	toxin biosynthetic process	4	3	0.4	0.00374	CYS4,MET5,MET3
GO:0009404	toxin metabolic process	4	3	0.4	0.00374	CYS4,MET5,MET3
GO:0019320	hexose catabolic process	4	3	0.4	0.00374	GAL7,GAL10,PYK1
GO:0035999	tetrahydrofolate interconversion	4	3	0.4	0.00374	MET13,SHM2,SHM1
GO:0070813	hydrogen sulfide metabolic process	4	3	0.4	0.00374	CYS4,MET5,MET3
GO:0070814	hydrogen sulfide biosynthetic process	4	3	0.4	0.00374	CYS4,MET5,MET3
GO:1901070	guanosine-containing compound biosynthet...	4	3	0.4	0.00374	HPT1,GUA1,GUK1
GO:0000056	ribosomal small subunit export from nucl...	12	5	1.21	0.00436	RPS3,MEX67,MTR2,NOP9,LTV1
GO:0006730	one-carbon metabolic process	12	5	1.21	0.00436	SAM2,MET13,SHM2,SAH1,SHM1
GO:0008610	lipid biosynthetic process	138	24	13.88	0.00467	OLE1,LIP1,CWH43,SCS7,KLMA_20052,ERG3,ERG28,CYP707A7,ERG9,PHS1,ERG1,SUR2,ERG6,SAH1,dsd1,HMG1,DPM1,FAS1,SUR4,ERG25,cyp524A1,ERG13,URA7,FAS2
GO:0000103	sulfate assimilation	8	4	0.8	0.00507	MET5,MET10,MET16,MET3

GO.ID	Term	Annotated gene	Significant	Expected	P-value	Genes
GO:0005991	trehalose metabolic process	8	4	0.8	0.00507	TPS2,TSL1,UGP1,TPS1
GO:0006551	leucine metabolic process	8	4	0.8	0.00507	DTD1,LEU1,LEU4,LEU3
GO:0009218	pyrimidine ribonucleotide metabolic proc...	8	4	0.8	0.00507	URA1,RNR2,URA7,RNR1
GO:0019748	secondary metabolic process	8	4	0.8	0.00507	CYS4,GSH1,MET5,MET3
GO:0044550	secondary metabolite biosynthetic proces...	8	4	0.8	0.00507	CYS4,GSH1,MET5,MET3
GO:0044270	cellular nitrogen compound catabolic pro...	139	24	13.99	0.00513	GPM3,DSS1,ENO,PGK,AAH1,RAG2,GPM1,TPA1,HAM1,MRT4,PAB1,VTS1,SAH1,TPI1,GAP1,FBA1,MTR4,PDC1,PYK1,RAG5,JHD2,GAP3,RRP42,PUB1
GO:0046700	heterocycle catabolic process	139	24	13.99	0.00513	GPM3,DSS1,ENO,PGK,AAH1,RAG2,GPM1,TPA1,HAM1,MRT4,PAB1,VTS1,SAH1,TPI1,GAP1,FBA1,MTR4,PDC1,PYK1,RAG5,JHD2,GAP3,RRP42,PUB1
GO:0006403	RNA localization	109	20	10.97	0.00515	SDA1,NOG1,ARX1,NOP53,RPS2,ENP1,UTP8,RPS3,RRS1,RLI1,MEX67,NMD3,RIX1,MTR2,MTR4,NUG1,CEX1,NEW1,NOP9,LTV1
GO:0044271	cellular nitrogen compound biosynthetic ...	910	112	91.56	0.00541	RpL37a,RPA49,PRS5,GPM3,NOP1,SOH1,UTP5,HPT1,GUA1,BIO2,RPL17B,GAL80,ENO,MES1,YTA7,LIP1,FAU1,PGK,MSW1,FUR1,AAH1,RAG2,URA1,GPM1,RPC37,UTP15,UTP11,ADK1,RPS2,FHL1,KLMA_20355,KLMA_20481,RPL22A,UTP8,TPA1,ADE2,RPS3,RPL5,NAN1,HAM1,GSH1,RNR2,MEU1,ETT1,RPL3,PAB1,RLI1,PDC2,YIH1,PAN5,TIF32,HAL9,KSS1,ADE5,7,GUS1,RPL15B,dsd1,HEM13,TPI1,GAP1,RIB7,UTP10,HAP1,MCM1,FMS1,FBA1,MAM33,RPC82,XPT1,RPL10A,GUK1,RPA135,PDX3,RPS14,ADE6,MSS51,LAC9,UTP4,RPC40,VAS1,RPL2,ACS2,URA2,KLMA_60069,RPA190,FUN12,REV1,PYK1,YAF9,EPL1,KLMA_60313,RAG5,JHD2,RPB1,CDC60,PET309,IKI3,URA7,ROX3,RNR1,SPT21,TIF3,MET16,HEM14,RPL19B,KLMA_70408,GAP3,HDA1,NIP1,KLMA_80256,MET6,ILS1
GO:0046034	ATP metabolic process	60	13	6.04	0.00553	GPM3,ENO,PGK,RAG2,GPM1,ADK1,PDC2,TPI1,GAP1,FBA1,PYK1,RAG5,GAP3

GO.ID	Term	Annotated gene	Significant	Expected	P-value	Genes
GO:0043604	amide biosynthetic process	302	44	30.38	0.00577	RpL37a,BIO2,RPL17B,MES1,LIP1,FAU1,MSW1,UTP11,RPS2,KLMA_20355,RPL22A,TPA1,RPS3,RPL5,GSH1,ETT1,RPL3,PAB1,RLI1,YIH1,PAN5,TIF32,GUS1,RPL15B,FMS1,MAM33,RPL10A,RPS14,MSS51,VAS1,RPL2,ACS2,KLMA_60069,FUN12,KLMA_60313,CDC60,PET309,IKI3,TIF3,MET16,RPL19B,NIP1,KLMA_80256,ILS1
GO:0000096	sulfur amino acid metabolic process	23	7	2.31	0.00578	SAM2,MET13,CYS4,MEU1,MET5,MET3,MET6
GO:0006091	generation of precursor metabolites and ...	103	19	10.36	0.00597	ACO2,GPM3,ENO,PGK,RAG2,GPM1,GSY2,PDC2,TPI1,GAP1,HAP1,FBA1,MAM33,MDH1,PYK1,UGP1,RAG5,GAP3,GDS1
GO:0006575	cellular modified amino acid metabolic p...	41	10	4.13	0.00604	FAU1,MET13,SHM2,GSH1,PAN5,SAH1,DUG1,FMS1,SHM1,MET16
GO:0070925	organelle assembly	89	17	8.95	0.00637	RPF2,NOP53,IPI3,RPL5,MRT4,IPI1,DRS1,RPL3,MDN1,RIX1,RRP5,KLMA_60069,BRX1,FUN12,RSA4,MAK21,PUB1
GO:0006913	nucleocytoplasmic transport	127	22	12.78	0.00698	GSP1,SDA1,NOG1,ARX1,NOP53,RPS2,ENP1,UTP8,RPS3,RRS1,RLI1,MEX67,NMD3,RIX1,MTR2,MTR4,NUG1,CEX1,NEW1,NOP9,LTV1,KAP123
GO:0034655	nucleobase-containing compound catabolic...	127	22	12.78	0.00698	GPM3,DSS1,ENO,PGK,RAG2,GPM1,TPA1,HAM1,MRT4,PAB1,VTS1,SAH1,TPI1,GAP1,FBA1,MTR4,PYK1,RAG5,JHD2,GAP3,RRP42,PUB1
GO:0051169	nuclear transport	127	22	12.78	0.00698	GSP1,SDA1,NOG1,ARX1,NOP53,RPS2,ENP1,UTP8,RPS3,RRS1,RLI1,MEX67,NMD3,RIX1,MTR2,MTR4,NUG1,CEX1,NEW1,NOP9,LTV1,KAP123
GO:0009205	purine ribonucleoside triphosphate metab...	62	13	6.24	0.00739	GPM3,ENO,PGK,RAG2,GPM1,ADK1,PDC2,TPI1,GAP1,FBA1,PYK1,RAG5,GAP3
GO:0090304	nucleic acid metabolic process	1024	123	103.03	0.00789	SPB1,RPA49,NOP1,SOH1,DSS1,UTP5,EBP2,RPF2,RSE1,GAL80,MES1,HAS1,YTA7,MSW1,NOG1,UTP13,RPC37,DBP7,UTP15,UTP11,ADK1,NOP53,NOP58,FHL1,NOP12,ENP1,CBF5,KLMA_20481,RLP7,UTP8,TPA1,MPP10,IPI3,NAN1,MRT4,RRS1,IPI1,DCAF13,DRS1,NOB1,RPL3,PAB1,MRPL15,PDC2,BFR2,PWP2,DBP3,RRP3,HAL9,VTS1,RAD54,KSS1,MTO1,GUS1,ECM16,MDN1,UTP10,GAR1,CIC1,HAP1,ERB1,MCM1,RRP12,IMP4,YTM1,NOP19,RPC82,RIX1,UTP21,PUS1,BMS1,UTP6,PRP43,GEP3,MTR4,UTP18,ENP2,RPA135,RRP5,PWP1,NOP56,LAC9,UTP4,NUG

GO.ID	Term	Annotated gene	Significant	Expected	P-value	Genes
						1,RPC40,VAS1,NOP7,DIP2,IMP3,EXO1,BRX1,URB1,DHR2,RPA190,FUN12,REV1,YAF9,NOP15,KRE33,EPL1,FCF2,KLMA_60313,NOP4,JHD2,RPB1,CDC60,IKI3,ROX3,RNR1,SPT21,NOP14,NOC4,RRP9,PUS4,KLMA_70408,NOP9,RRP42,PUB1,UTP25,HDA1,NOP2,DEF1,ILS1
GO:0006760	folic acid-containing compound metabolic...	9	4	0.91	0.00841	FAU1,MET13,SHM2,SHM1
GO:0042451	purine nucleoside biosynthetic process	9	4	0.91	0.00841	HPT1,GUA1,MEU1,GUK1
GO:0043101	purine-containing compound salvage	9	4	0.91	0.00841	HPT1,AAH1,MEU1,XPT1
GO:0046129	purine ribonucleoside biosynthetic proce...	9	4	0.91	0.00841	HPT1,GUA1,MEU1,GUK1
GO:0009144	purine nucleoside triphosphate metabolic...	63	13	6.34	0.00848	GPM3,ENO,PGK,RAG2,GPM1,ADK1,PDC2,TPI1,GAP1,FBA1,PYK1,RAG5,GAP3
GO:1901615	organic hydroxy compound metabolic proce...	70	14	7.04	0.00852	ADH4,ERG3,ERG28,CYP707A7,ERG9,ERG1,PDC2,ERG6,dsd1,PDX3,ERG25,PDC1,cyp524A1,ERG13
GO:0000097	sulfur amino acid biosynthetic process	19	6	1.91	0.00864	MET13,CYS4,MEU1,MET5,MET3,MET6
GO:0006633	fatty acid biosynthetic process	19	6	1.91	0.00864	OLE1,SCS7,PHS1,FAS1,SUR4,FAS2
GO:0046112	nucleobase biosynthetic process	19	6	1.91	0.00864	AAH1,URA1,ADE5,7,URA2,URA7,MET6
GO:0009098	leucine biosynthetic process	5	3	0.5	0.00865	LEU1,LEU4,LEU3
GO:0031120	snRNA pseudouridine synthesis	5	3	0.5	0.00865	CBF5,GAR1,PUS1
GO:0040031	snRNA modification	5	3	0.5	0.00865	CBF5,GAR1,PUS1
GO:0016051	carbohydrate biosynthetic process	50	11	5.03	0.00924	PGK,RAG2,TPS2,TSL1,GPM1,GSY2,TPI1,FBA1,UGP1,VID24,TPS1
GO:0042278	purine nucleoside metabolic process	14	5	1.41	0.00931	HPT1,GUA1,MEU1,SAH1,GUK1

GO.ID	Term	Annotated gene	Significant	Expected	P-value	Genes
GO:0046128	purine ribonucleoside metabolic process	14	5	1.41	0.00931	HPT1,GUA1,MEU1,SAH1,GUK1
GO:0006790	sulfur compound metabolic process	93	17	9.36	0.00997	SAM2,BIO2,MET13,CYS4,GSH1,MEU1,PDC2,SAH1,DUG1,HMG1,MET5,MET10,ACS2,ERG13,MET16,MET3,MET6

GO terms enriched in significantly down-regulated genes (cellular components)

GO.ID	Term	Annotated gene	Significant	Expected	P-value	Genes
GO:0030684	preribosome	116	61	11.18	< 1e-30	SPB1,NOP1,UTP5,EBP2,HAS1,NOG1,UTP13,UTP15,UTP11,ARX1,RPS2,NOP58,NOP12,ENP1,RLP7,UTP8,MPP10,RPS3,NAN1,MRT4,RRS1,DCAF13,DRS1,NOB1,BFR2,PWP2,DBP3,ECM16,UTP10,CIC1,ERB1,RRP12,IMP4,YTM1,NOP19,UTP21,BMS1,UTP6,NOC2,UTP18,ENP2,RRP5,NOP56,UTP4,NUG1,NOP7,DIP2,IMP3,BRX1,NOP15,KRE33,NOP14,NOC4,RRP9,MAK21,NOP9,LTV1,RLP24,UTP25,NOP2,PUF6
GO:0005730	nucleolus	197	72	18.99	1.20E-26	SPB1,RPA49,NOP1,UTP5,EBP2,RPF2,HAS1,NOG1,UTP13,DBP7,UTP15,UTP11,ARX1,NOP53,NOP58,FHL1,NOP12,ENP1,CBF5,RLP7,UTP8,MPP10,NAN1,MRT4,RRS1,DCAF13,NOB1,BFR2,PWP2,DBP3,RRB1,ECM16,UTP10,GAR1,CIC1,ERB1,IMP4,YTM1,NOP19,UTP21,BMS1,UTP6,MTR4,UTP18,ENP2,RPA135,RRP5,PWP1,NOP56,UTP4,NUG1,RPC40,NOP7,DIP2,ACS2,IMP3,BRX1,URB1,DHR2,RPA190,NOP15,KRE33,RSA4,NOP14,NOC4,RRP9,SRP40,NOP9,RLP24,UTP25,HDA1,NOP2
GO:0032040	small-subunit processome	41	29	3.95	2.90E-21	NOP1,UTP5,UTP13,UTP15,UTP11,RPS2,NOP58,UTP8,MPP10,NAN1,DCAF13,BFR2,PWP2,ECM16,UTP10,IMP4,UTP21,UTP6,UTP18,ENP2,RRP5,NOP56,UTP4,DIP2,IMP3,NOP14,NOC4,RRP9,UTP25
GO:1990904	ribonucleoprotein complex	470	102	45.3	7.50E-18	SPB1,RpL37a,NOP1,UTP5,RPL17B,EBP2,RSE1,HAS1,NOG1,RPL24,UTP13,UTP15,UTP11,ARX1,RPS2,CYS4,NOP58,KLMA_20355,NOP12,ENP1,CBF5,RLP7,RPL22A,UTP8,MPP10,RPS3,RPL5,NAN1,MRT4,RRS1,DCAF13,DRS1,NOB1,RPL3,PAB1,RLI1,MRPL15,BFR2,PWP2,YIH1,DBP3,TIF32,VTS1,GUS1,ECM16,RPL15B,UTP10,GAR1,CIC1,ERB1,RRP12,IMP4,YTM1,NOP19,NMD3,MET5,UTP21,BMS1,RPL10A,UTP6,PRP43,NOC2,UTP18,ENP2,LEU1,R

GO.ID	Term	Annotated gene	Significant	Expected	P-value	Genes
						PS14,RRP5,NOP56,UTP4,NUG1,NOP7,RPL2,DIP2,MAP1,IMP3,KLMA_60069,BRX1,TEF4,FUN12,NOP15,KRE33,KLMA_60313,UGP1,RPB1,NEW1,NOP14,TIF3,NOC4,RRP9,RPL19B,MAK21,NOP9,LTV1,RLP24,PUB1,UTP25,NOP2,NIP1,KLMA_80256,PUF6,KAP123,RPL8B
GO:0044452	nucleolar part	67	30	6.46	3.40E-14	RPA49,NOP1,UTP5,HAS1,UTP13,UTP15,NOP58,CBF5,UTP8,MPP10,NAN1,PWP2,UTP10,GAR1,ERB1,IMP4,YTM1,UTP21,UTP6,UTP18,RPA135,NOP56,UTP4,RPC40,NOP7,DIP2,IMP3,RPA190,RRP9,HDA1
GO:0030686	90S preribosome	40	22	3.86	5.10E-13	UTP13,ENP1,MPP10,PWP2,UTP10,ERB1,IMP4,YTM1,NOP19,UTP21,BMS1,UTP6,NOC2,UTP18,UTP4,NOP7,DIP2,IMP3,NOP14,NOC4,MAK21,NOP9
GO:0030687	preribosome, large subunit precursor	46	22	4.43	2.10E-11	SPB1,EBP2,HAS1,NOG1,ARX1,RLP7,MRT4,RRS1,DRS1,DBP3,CIC1,ERB1,YTM1,NOC2,NUG1,NOP7,BRX1,NOP15,MAK21,RLP24,NOP2,PUF6
GO:0031981	nuclear lumen	560	87	53.97	7.00E-07	SPB1,RPA49,NOP1,SOH1,UTP5,EBP2,RPF2,HAS1,NOG1,UTP13,DBP7,UTP15,UTP11,ARX1,NOP53,NOP58,FHL1,NOP12,ENP1,CBF5,RLP7,UTP8,MPP10,IPI3,NAN1,MRT4,RSC2,RRS1,IPI1,DCAF13,NOB1,BFR2,MEX67,PWP2,DBP3,RRB1,ECM16,MDN1,UTP10,GAR1,CIC1,ERB1,IMP4,YTM1,NOP19,DUS3,RIX1,UTP21,MTR2,BMS1,UTP6,MTR4,UTP18,ENP2,RPA135,RRP5,PWP1,NOP56,UTP4,NUG1,RPC40,NOP7,DIP2,ACS2,IMP3,BRX1,URB1,DHR2,RPA190,REV1,YAF9,NOP15,KRE33,EPL1,RPB1,ROX3,RSA4,NOP14,NOC4,RRP9,SRP40,NOP9,RLP24,RRP42,UTP25,HDA1,NOP2
GO:0034388	Pwp2p-containing subcomplex of 90S preri...	6	6	0.58	7.70E-07	UTP13,PWP2,UTP21,UTP6,UTP18,DIP2
GO:0043228	non-membrane-bounded organelle	883	121	85.11	2.60E-06	SPB1,RpL37a,RPA49,NOP1,UTP5,RPL17B,EBP2,RPF2,HAS1,NOG1,RPL24,UTP13,DBP7,UTP15,HSL1,UTP11,ARX1,NOP53,RPS2,CYS4,NOP58,FHL1,KLMA_20355,NOP12,ENP1,CBF5,RLP7,RPL22A,UTP8,MPP10,RPS3,RPL5,NAN1,MRT4,RSC2,RRS1,ERG1,DCAF13,NOB1,RPL3,PAB1,RLI1,MRPL15,BFR2,PWP2,YIH1,DBP3,TIF32,VTS1,ERG6,GUS1,RRB1,ECM16,RPL15B,UTP10,GAR1,CIC1,ERB1,IMP4,YTM1,NOP19,NMD3,MET5,UTP21,FAS1,BMS1,RPL10A,UTP6,MTR4,UTP18,ENP2,LEU1,RPA135,YJU3,RPS14,

GO.ID	Term	Annotated gene	Significant	Expected	P-value	Genes
						RRP5,PWP1,NOP56,UTP4,NUG1,RPC40,NOP7,RPL2,DIP2,ACS2,MAP1,IMP3,KLMA_60069,BRX1,URB1,TEF4,DHR2,RPA190,FUN12,REV1,YAF9,NOP15,KRE33,KLMA_60313,UGP1,RPB1,RSA4,HSL7,SPT21,NOP14,TIF3,NOC4,RRP9,RPL19B,SRP40,NOP9,RLP24,PUB1,UTP25,HDA1,NOP2,NIP1,KLMA_80256,PUF6,KAP123,RPL8B
GO:0043232	intracellular non-membrane-bounded organ...	883	121	85.11	2.60E-06	SPB1,RpL37a,RPA49,NOP1,UTP5,RPL17B,EBP2,RPF2,HAS1,NOG1,RPL24,UTP13,DBP7,UTP15,HSL1,UTP11,ARX1,NOP53,RPS2,CYS4,NOP58,FHL1,KLMA_20355,NOP12,ENP1,CBF5,RLP7,RPL22A,UTP8,MPP10,RPS3,RPL5,NAN1,MRT4,RSC2,RRS1,ERG1,DCAF13,NOB1,RPL3,PAB1,RLI1,MRPL15,BFR2,PWP2,YIH1,DBP3,TIF32,VTS1,ERG6,GUS1,RRB1,ECM16,RPL15B,UTP10,GAR1,CIC1,ERB1,IMP4,YTM1,NOP19,NMD3,MET5,UTP21,FAS1,BMS1,RPL10A,UTP6,MTR4,UTP18,ENP2,LEU1,RPA135,YJU3,RPS14,RRP5,PWP1,NOP56,UTP4,NUG1,RPC40,NOP7,RPL2,DIP2,ACS2,MAP1,IMP3,KLMA_60069,BRX1,URB1,TEF4,DHR2,RPA190,FUN12,REV1,YAF9,NOP15,KRE33,KLMA_60313,UGP1,RPB1,RSA4,HSL7,SPT21,NOP14,TIF3,NOC4,RRP9,RPL19B,SRP40,NOP9,RLP24,PUB1,UTP25,HDA1,NOP2,NIP1,KLMA_80256,PUF6,KAP123,RPL8B
GO:0034455	t-UTP complex	7	6	0.67	4.90E-06	UTP5,UTP15,UTP8,NAN1,UTP10,UTP4
GO:0030688	preribosome, small subunit precursor	21	10	2.02	8.10E-06	ENP1,RPS3,NOB1,UTP10,RRP12,KRE33,NOP14,NOC4,NOP9,LTV1
GO:0031428	box C/D snoRNP complex	6	5	0.58	4.50E-05	NOP1,HAS1,NOP58,NOP56,RRP9
GO:0044445	cytosolic part	97	22	9.35	8.80E-05	PFK1,ENO,ARX1,RPS2,KLMA_20355,RPL22A,RPS3,RPL5,RNR2,RPL3,RLI1,NMD3,FAS1,LEU1,MAP1,KLMA_60069,FUN12,KLMA_60313,PFK2,RNR1,FAS2,RPL19B
GO:0022626	cytosolic ribosome	48	14	4.63	0.0001	ARX1,RPS2,KLMA_20355,RPL22A,RPS3,RPL5,RPL3,RLI1,NMD3,MAP1,KLMA_60069,FUN12,KLMA_60313,RPL19B

GO.ID	Term	Annotated gene	Significant	Expected	P-value	Genes
GO:0010494	cytoplasmic stress granule	60	16	5.78	0.00011	CYS4,PAB1,TIF32,GUS1,MET5,LEU1,MAP1,TEF4,FUN12,UGP1,RPB1,TIF3,PUB1,NIP1,KLMA_80256,KAP123
GO:0044428	nuclear part	739	98	71.23	0.00016	SPB1,RPA49,NOP1,SOH1,UTP5,EBP2,RPF2,RSE1,HAS1,NOG1,UTP13,RPC37,DBP7,UTP15,UTP11,ARX1,NOP53,NOP58,FHL1,NOP12,ENP1,CBF5,RLP7,UTP8,MPP10,IPI3,NAN1,MRT4,RSC2,RRS1,IPI1,DCAF13,NOB1,NSR1,BFR2,MEX67,PWP2,DBP3,RRB1,ECM16,MDN1,UTP10,GAR1,CIC1,ERB1,NUP116,IMP4,YTM1,NOP19,HMG1,DUS3,RPC82,RIX1,UTP21,MTR2,BMS1,UTP6,PRP43,NOC2,MTR4,UTP18,ENP2,RPA135,RRP5,PWP1,NOP56,UTP4,NUG1,RPC40,NOP7,DIP2,ACS2,IMP3,BRX1,URB1,DHR2,RPA190,REV1,YAF9,NOP15,CEX1,KRE33,EPL1,RPB1,ROX3,RSA4,NOP14,NOC4,RRP9,MAK21,SRP40,NOP9,RLP24,RRP42,UTP25,HDA1,NOP2,KAP123
GO:0005634	nucleus	1128	138	108.72	0.00023	SPB1,CCN1,RPA49,KLMA_10146,NOP1,GSP1,SOH1,UTP5,SDA1,EBP2,RPF2,RSE1,GAL80,HAS1,TDA1,YTA7,AAH1,NOG1,UTP13,RPC37,DBP7,UTP15,UTP11,ARX1,NOP53,NOP58,FHL1,NOP12,ENP1,CBF5,RLP7,UTP8,TPA1,MPP10,IPI3,NAN1,LIA1,HAM1,MRT4,RSC2,RRS1,IPI1,RNR2,DCAF13,MEU1,ETT1,NOB1,PAB1,RLI1,NSR1,BFR2,MEX67,PWP2,DBP3,HAL9,VTS1,KLMA_30614,RAD54,KSS1,RRB1,ECM16,MDN1,UTP10,GAR1,CIC1,HAP1,ERB1,NUP116,MCM1,RRP12,IMP4,YTM1,NOP19,NMD3,HMG1,DUS3,RPC82,RIX1,UTP21,MTR2,PUS1,BMS1,UTP6,PRP43,NOC2,MTR4,UTP18,UPC2,ENP2,RPA135,RRP5,PWP1,NOP56,LAC9,UTP4,NUG1,RPC40,NOP7,DIP2,ACS2,ALB1,IMP3,EXO1,BRX1,URB1,DHR2,RPA190,REV1,YAF9,NOP15,CEX1,KRE33,EPL1,RGT1,JHD2,LEU3,RPB1,IKI3,ROX3,RSA4,NOP14,NOC4,RRP9,MAK21,SRP40,PCL1,KLMA_70408,NOP9,LTV1,RLP24,RRP42,PUB1,UTP25,HDA1,NOP2,TRM6,DEF1,KAP123
GO:0033553	rDNA heterochromatin	11	6	1.06	0.00023	UTP5,UTP15,UTP8,NAN1,UTP10,HDA1
GO:0030689	Noc complex	5	4	0.48	0.00039	NOC2,NOP14,NOC4,MAK21
GO:0035770	ribonucleoprotein granule	76	17	7.33	0.00067	CYS4,PAB1,TIF32,VTS1,GUS1,MET5,LEU1,MAP1,TEF4,FUN12,UGP1,RPB1,TIF3,PUB1,NIP1,KLMA_80256,KAP123

GO.ID	Term	Annotated gene	Significant	Expected	P-value	Genes
GO:0036464	cytoplasmic ribonucleoprotein granule	76	17	7.33	0.00067	CYS4,PAB1,TIF32,VTS1,GUS1,MET5,LEU1,MAP1,TEF4,FUN12,UGP1,RPB1,TIF3,PUB1,NIP1,KLMA_80256,KAP123
GO:0034457	Mpp10 complex	3	3	0.29	0.00089	MPP10,IMP4,IMP3
GO:0070545	PeBoW complex	3	3	0.29	0.00089	ERB1,YTM1,NOP7
GO:0097344	Rix1 complex	3	3	0.29	0.00089	IPI3,IPI1,RIX1
GO:0031974	membrane-enclosed lumen	790	97	76.14	0.00302	SPB1,RPA49,NOP1,SOH1,UTP5,EBP2,RPF2,HAS1,NOG1,GPM1,UTP13,DBP7,UTP15,ADH4,UTP11,ADK1,ARX1,NOP53,ERG3,NOP58,FHL1,NOP12,ENP1,CBF5,RLP7,UTP8,MPP10,IPI3,NAN1,MRT4,RSC2,RRS1,IPI1,DCAF13,KLMA_30226,NOB1,MRPL15,BFR2,MEX67,PWP2,DBP3,RRB1,ECM16,MDN1,UTP10,GAR1,CIC1,ERB1,IMP4,MAM33,YTM1,NOP19,DUS3,RIX1,UTP21,MTR2,BMS1,UTP6,MTR4,UTP18,ENP2,ERV1,RPA135,PDX3,CCP1,RRP5,PWP1,NOP56,UTP4,NUG1,RPC40,NOP7,DIP2,ACS2,IMP3,BRX1,URB1,DHR2,RPA190,REV1,YAF9,NOP15,KRE33,EPL1,RPB1,ROX3,RSA4,NOP14,NOC4,RRP9,SRP40,NOP9,RLP24,RRP42,UTP25,HDA1,NOP2
GO:0043233	organelle lumen	790	97	76.14	0.00302	SPB1,RPA49,NOP1,SOH1,UTP5,EBP2,RPF2,HAS1,NOG1,GPM1,UTP13,DBP7,UTP15,ADH4,UTP11,ADK1,ARX1,NOP53,ERG3,NOP58,FHL1,NOP12,ENP1,CBF5,RLP7,UTP8,MPP10,IPI3,NAN1,MRT4,RSC2,RRS1,IPI1,DCAF13,KLMA_30226,NOB1,MRPL15,BFR2,MEX67,PWP2,DBP3,RRB1,ECM16,MDN1,UTP10,GAR1,CIC1,ERB1,IMP4,MAM33,YTM1,NOP19,DUS3,RIX1,UTP21,MTR2,BMS1,UTP6,MTR4,UTP18,ENP2,ERV1,RPA135,PDX3,CCP1,RRP5,PWP1,NOP56,UTP4,NUG1,RPC40,NOP7,DIP2,ACS2,IMP3,BRX1,URB1,DHR2,RPA190,REV1,YAF9,NOP15,KRE33,EPL1,RPB1,ROX3,RSA4,NOP14,NOC4,RRP9,SRP40,NOP9,RLP24,RRP42,UTP25,HDA1,NOP2

GO.ID	Term	Annotated gene	Significant	Expected	P-value	Genes
GO:0070013	intracellular organelle lumen	790	97	76.14	0.00302	SPB1,RPA49,NOP1,SOH1,UTP5,EBP2,RPF2,HAS1,NOG1,GPM1,UTP13,DBP7,UTP15,ADH4,UTP11,ADK1,ARX1,NOP53,ERG3,NOP58,FHL1,NOP12,ENP1,CBF5,RLP7,UTP8,MPP10,IPI3,NAN1,MRT4,RSC2,RRS1,IPI1,DCAF13,KLMA_30226,NOB1,MRPL15,BFR2,MEX67,PWP2,DBP3,RRB1,ECM16,MDN1,UTP10,GAR1,CIC1,ERB1,IMP4,MAM33,YTM1,NOP19,DUS3,RIX1,UTP21,MTR2,BMS1,UTP6,MTR4,UTP18,ENP2,ERV1,RPA135,PDX3,CCP1,RRP5,PWP1,NOP56,UTP4,NUG1,RPC40,NOP7,DIP2,ACS2,IMP3,BRX1,URB1,DHR2,RPA190,REV1,YAF9,NOP15,KRE33,EPL1,RPB1,ROX3,RSA4,NOP14,NOC4,RRP9,SRP40,NOP9,RLP24,RRP42,UTP25,HDA1,NOP2
GO:0005732	small nucleolar ribonucleoprotein comple...	28	8	2.7	0.00378	NOP1,HAS1,NOP58,CBF5,MPP10,GAR1,NOP56,RRP9
GO:0022625	cytosolic large ribosomal subunit	24	7	2.31	0.00591	ARX1,KLMA_20355,RPL22A,RPL5,RPL3,NMD3,RPL19B
GO:0000792	heterochromatin	19	6	1.83	0.00701	UTP5,UTP15,UTP8,NAN1,UTP10,HDA1
GO:0031429	box H/ACA snoRNP complex	5	3	0.48	0.00765	HAS1,CBF5,GAR1
GO:0072588	box H/ACA RNP complex	5	3	0.48	0.00765	HAS1,CBF5,GAR1
GO:0005835	fatty acid synthase complex	2	2	0.19	0.00926	FAS1,FAS2
GO:0005945	6-phosphofructokinase complex	2	2	0.19	0.00926	PFK1,PFK2
GO:0005946	alpha,alpha-trehalose-phosphate synthase...	2	2	0.19	0.00926	TPS2,TPS1

GO.ID	Term	Annotated gene	Significant	Expected	P-value	Genes
GO:0005971	ribonucleoside-diphosphate reductase com...	2	2	0.19	0.00926	RNR2,RNR1
GO:0009337	sulfite reductase complex (NADPH)	2	2	0.19	0.00926	MET5,MET10
GO:0030690	Noc1p-Noc2p complex	2	2	0.19	0.00926	NOC2,MAK21
GO:0030692	Noc4p-Nop14p complex	2	2	0.19	0.00926	NOP14,NOC4
GO:0042272	nuclear RNA export factor complex	2	2	0.19	0.00926	MEX67,MTR2

GO terms enriched in significantly up-regulated genes (cellular components)

GO.ID	Term	Annotated gene	Significant	Expected	P-value	Genes
GO:0016021	integral component of membrane	973	176	99.84	6.10E-20	ARN2,KLMA_10026,KLMA_10039,PRM1,KLMA_10174,KLMA_10246,HRD1,MCH2,DIT2,ATG27,STE3,KLMA_10393,PMC1,PMA1,HGT1,KLMA_10560,RRT8,YKT6,DAL5,RAN1,BIG1,PEP12,UBP16,GDT1,YDC1,FCY2,KLMA_20003,FRE4,ADY2,KLMA_20010,RFT1,KLMA_20072,NIPA2,KLMA_20101,KLMA_20131,PXA1,THI72,JEN1,KLMA_20269,YHM2,KLMA_20345,KLMA_20368,OSW5,POM33,uapC,ATO2,CTS2,SNG1,PEX11,KLMA_20658,KLMA_20730,MEP2,ODC2,KLMA_20823,KHA1,FEN2,KLMA_30014,PUT4,mug157,KLMA_30124,TOK1,MMM1,KLMA_30260,KLMA_30272,VTI1,PDR12,PHM7,KLMA_30399,DPP1,KLMA_30444,KLMA_30524,KLMA_30555,KLMA_30573,KLMA_30601,STL1,mug70,KLMA_30624,KLMA_30642,PNS1,KLMA_30672,ATG32,LAC12,KLMA_40006,KLMA_40042,MSB2,NYV1,ENA5,KLMA_40213,CHS1,PDR5,KLMA_40260,KLMA_40272,KLMA_40358,GAS4,VTC1,KLMA_40446,YET3,KLMA_40484,PUN1,PXA2,GEX1,PEX25,BTN1,KLMA_50156,QDR3,FIG1,KLMA_50249,KLMA_50252,SOP4,KLMA_50327,HUT1,RAG1,HAK1,OPT2,MTC4,NCE102,VPS70,dpp1,KLMA_50601,KLMA_60005,DIP5,RMD1,KLMA_60123,KLMA_60124,VCX1,KLMA_60241,KLMA_60293,KLMA_60323,KLMA_60367,FMP37,KLMA_60426,KLMA_60475,SRF1,KLMA_60496,ERC1,KLMA_60

GO.ID	Term	Annotated gene	Significant	Expected	P-value	Genes
						558,KLMA_70001,HXT15,KLMA_70004,FMP32,KLMA_70053,PER33,KLMA_70089,ERV2,ywtG,ALG12,KLMA_70209,AGP2,PHO84,FMP27,ANT1,PTR2,mcfL,KLMA_80110,KTR5,AVT7,lag1,SGA1,SFC1,CRC1,KLMA_80231,VAM3,SFK1,HXT14
GO:0031224	intrinsic component of membrane	978	176	100.35	1.10E-19	ARN2,KLMA_10026,KLMA_10039,PRM1,KLMA_10174,KLMA_10246,HRD1,MCH2,DIT2,ATG27,STE3,KLMA_10393,PMC1,PMA1,HGT1,KLMA_10560,RRT8,YKT6,DAL5,RAN1,BIG1,PEP12,UBP16,GDT1,YDC1,FCY2,KLMA_20003,FRE4,ADY2,KLMA_20010,RFT1,KLMA_20072,NIPA2,KLMA_20101,KLMA_20131,PXA1,THI72,JEN1,KLMA_20269,YHM2,KLMA_20345,KLMA_20368,OSW5,POM33,uapC,ATO2,CTS2,SNG1,PEX11,KLMA_20658,KLMA_20730,MEP2,ODC2,KLMA_20823,KHA1,FEN2,KLMA_30014,PUT4,mug157,KLMA_30124,TOK1,MMM1,KLMA_30260,KLMA_30272,VTI1,PDR12,PHM7,KLMA_30399,DPP1,KLMA_30444,KLMA_30524,KLMA_30555,KLMA_30573,KLMA_30601,STL1,mug70,KLMA_30624,KLMA_30642,PNS1,KLMA_30672,ATG32,LAC12,KLMA_40006,KLMA_40042,MSB2,NYV1,ENA5,KLMA_40213,CHS1,PDR5,KLMA_40260,KLMA_40272,KLMA_40358,GAS4,VTC1,KLMA_40446,YET3,KLMA_40484,PUN1,PXA2,GEX1,PEX25,BTN1,KLMA_50156,QDR3,FIG1,KLMA_50249,KLMA_50252,SOP4,KLMA_50327,HUT1,RAG1,HAK1,OPT2,MTC4,NCE102,VPS70,dpp1,KLMA_50601,KLMA_60005,DIP5,RMD1,KLMA_60123,KLMA_60124,VCX1,KLMA_60241,KLMA_60293,KLMA_60323,KLMA_60367,FMP37,KLMA_60426,KLMA_60475,SRF1,KLMA_60496,ERC1,KLMA_60558,KLMA_70001,HXT15,KLMA_70004,FMP32,KLMA_70053,PER33,KLMA_70089,ERV2,ywtG,ALG12,KLMA_70209,AGP2,PHO84,FMP27,ANT1,PTR2,mcfL,KLMA_80110,KTR5,AVT7,lag1,SGA1,SFC1,CRC1,KLMA_80231,VAM3,SFK1,HXT14

GO.ID	Term	Annotated gene	Significant	Expected	P-value	Genes
GO:0044425	membrane part	1193	189	122.41	8.70E-15	ARN2,KLMA_10026,KLMA_10039,PRM1,KLMA_10174,KLMA_10246,HRD1,MCH2,DIT2,ATG27,STE3,KLMA_10393,PMC1,PMA1,HGT1,KLMA_10560,RRT8,YKT6,DAL5,RAN1,BIG1,PEP12,UBP16,GDT1,YDC1,FCY2,KLMA_20003,FRE4,ADY2,KLMA_20010,RFT1,KLMA_20072,NIPA2,KLMA_20101,KLMA_20131,PXA1,THI72,JEN1,KLMA_20269,YHM2,KLMA_20345,KLMA_20368,OSW5,IMP1,POM33,uapC,FAB1,ATO2,CTS2,SNG1,PEX11,KLMA_20658,KLMA_20730,MEP2,ODC2,STE4,KLMA_20823,KHA1,FEN2,KLMA_30014,PUT4,mug157,KLMA_30124,TOK1,MMM1,KLMA_30260,KLMA_30272,VTI1,PDR12,PHM7,KLMA_30399,SNX4,DPP1,KLMA_30428,KLMA_30444,KLMA_30524,KLMA_30555,KLMA_30573,KLMA_30601,STL1,mug70,KLMA_30624,KLMA_30642,PNS1,KLMA_30672,ATG32,LAC12,KLMA_40006,KLMA_40042,MSB2,NYV1,ENA5,KLMA_40213,CHS1,PDR5,KLMA_40260,KLMA_40272,KLMA_40333,KLMA_40358,GAS4,VTC1,KLMA_40446,YET3,KLMA_40484,PUN1,PXA2,GEX1,PEX25,BTN1,KLMA_50156,APS2,QDR3,FIG1,KLMA_50249,KLMA_50252,SOP4,KLMA_50327,HUT1,RAG1,HAK1,OPT2,MTC4,NCE102,VPS70,dpp1,KLMA_50601,KLMA_60005,DIP5,RMD1,KLMA_60123,KLMA_60124,GPA1,VCX1,KLMA_60241,KLMA_60293,KLMA_60323,KLMA_60367,FMP37,KLMA_60426,INP1,KLMA_60475,SRF1,KLMA_60496,ERC1,KLMA_60558,KLMA_70001,HXT15,KLMA_70004,FMP32,KLMA_70053,PER33,KLMA_70089,ERV2,KLMA_70118,ywtG,ALG12,KLMA_70209,AGP2,KLMA_70233,PHO84,FMP27,ANT1,PTR2,SNF8,mcfL,ATG20,KLMA_80110,KTR5,AVT7,lag1,SGA1,SFC1,CRC1,KLMA_80231,VAM3,SFK1,HXT14
GO:0016020	membrane	1335	201	136.98	1.70E-13	ARN2,KLMA_10026,KLMA_10039,PRM1,ATG26,VPS60,KLMA_10174,KLMA_10246,HRD1,PLB,MCH2,DIT2,FMP43,ATG27,STE3,KLMA_10393,PMC1,PMA1,KLMA_10486,HGT1,KLMA_10560,RRT8,YKT6,DAL5,RAN1,BIG1,PEP12,UBP16,GDT1,YDC1,FCY2,KLMA_20003,FRE4,ADY2,KLMA_20010,RFT1,KLMA_20072,NIPA2,KLMA_20101,KLMA_20131,PXA1,THI72,JEN1,KLMA_20269,YHM2,KLMA_20345,KLMA_20368,OSW5,IMP1,POM33,uapC,FAB1,ATO2,FRQ1,CTS2,SNG1,PEX11,KLMA_20658,KLMA_20730,MEP2,HSP12,ODC2,STE4,KLMA_20823,KHA1,FEN2,KLMA_30014,PUT4,mug157,KLMA_30124,TOK1,MMM1,KLMA_30260,KLMA_30272,VTI1,PDR12,PHM7,KLMA_30399,SNX4,DPP1,KLMA_30428,K

GO.ID	Term	Annotated gene	Significant	Expected	P-value	Genes
						LMA_30444,KLMA_30524,PIB1,KLMA_30555,KLMA_30573,KLMA_30601,STL1,mug70,KLMA_30624,KLMA_30642,PNS1,KLMA_30672,ATG32,LAC12,KLMA_40006,KLMA_40042,MSB2,NYV1,ENA5,KLMA_40213,CHS1,SPO14,PDR5,KLMA_40260,KLMA_40272,KLMA_40333,KLMA_40358,GAS4,VTC1,KLMA_40446,YET3,KLMA_40484,PUN1,PXA2,GEX1,PEX25,BTN1,KLMA_50156,APS2,QDR3,FIG1,KLMA_50249,KLMA_50252,SOP4,KLMA_50327,HUT1,RAG1,HAK1,OPT2,MTC4,NCE102,VPS70,dpp1,KLMA_50601,KLMA_60005,MRF1,DIP5,RMD1,KLMA_60123,KLMA_60124,GPA1,VCX1,KLMA_60241,KLMA_60293,KLMA_60323,KLMA_60367,FMP37,KLMA_60426,SST2,INP1,KLMA_60475,SRF1,KLMA_60496,ERC1,KLMA_60558,KLMA_70001,HXT15,KLMA_70004,FMP32,KLMA_70053,PER33,KLMA_70089,ERV2,KLMA_70118,ywtG,KLMA_70146,ALG12,KLMA_70209,AGP2,KLMA_70233,PHO84,FMP27,ANT1,PTR2,SNF8,mcfL,ATG20,KLMA_80110,KTR5,AVT7,lag1,SGA1,SFC1,CRC1,KLMA_80231,VAM3,SFK1,HXT14
GO:0044438	microbody part	30	12	3.08	1.80E-05	KLMA_10026,PXA1,POT1,POX1,PEX11,MDH3,PXA2,PEX25,SPS19,INP1,ANT1,LPX1
GO:0044439	peroxisomal part	30	12	3.08	1.80E-05	KLMA_10026,PXA1,POT1,POX1,PEX11,MDH3,PXA2,PEX25,SPS19,INP1,ANT1,LPX1
GO:0005777	peroxisome	52	16	5.34	3.50E-05	KLMA_10026,KLMA_20057,PXA1,POT1,POX1,PEX11,CAT2,MDH3,PXA2,PEX25,PCD1,SPS19,TES1,INP1,ANT1,LPX1
GO:0042579	microbody	52	16	5.34	3.50E-05	KLMA_10026,KLMA_20057,PXA1,POT1,POX1,PEX11,CAT2,MDH3,PXA2,PEX25,PCD1,SPS19,TES1,INP1,ANT1,LPX1
GO:0005782	peroxisomal matrix	9	5	0.92	0.00098	POT1,POX1,MDH3,SPS19,LPX1
GO:0031907	microbody lumen	9	5	0.92	0.00098	POT1,POX1,MDH3,SPS19,LPX1
GO:0005779	integral component of peroxisomal membra...	13	6	1.33	0.00103	KLMA_10026,PXA1,PEX11,PXA2,PEX25,ANT1
GO:0031231	intrinsic component of peroxisomal membr...	13	6	1.33	0.00103	KLMA_10026,PXA1,PEX11,PXA2,PEX25,ANT1

GO.ID	Term	Annotated gene	Significant	Expected	P-value	Genes
GO:1990429	peroxisomal importomer complex	13	6	1.33	0.00103	PXA1,UBI4,PEX11,CAT2,MDH3,PEX25
GO:0005834	heterotrimeric G-protein complex	3	3	0.31	0.00107	STE4,GPA1,KLMA_70118
GO:0042597	periplasmic space	3	3	0.31	0.00107	PLB,FBP1,ATH1
GO:1905360	GTPase complex	3	3	0.31	0.00107	STE4,GPA1,KLMA_70118
GO:1990415	Pex17p-Pex14p docking complex	14	6	1.44	0.00164	PXA1,UBI4,PEX11,CAT2,MDH3,PEX25
GO:0071944	cell periphery	258	41	26.47	0.00225	LSB5,PLB,STE3,PMA1,KLMA_10486,YTA6,KLMA_20368,PIN3,FRQ1,MEP2,HSP12,STE4,TOK1,DPL1,MSB2,KLMA_40061,ENA5,KLMA_40213,PDR5,ELM1,GAS4,KLMA_40476,PUN1,APS2,QDR3,FIG1,PIR1,GPA1,STL1,SST2,CRR1,KLMA_70011,KIP2,KLMA_70118,ATH1,AGP2,PHO84,KLMA_80053,AVT7,SFK1,HOF1
GO:0005778	peroxisomal membrane	21	7	2.15	0.00367	KLMA_10026,PXA1,PEX11,PXA2,PEX25,INP1,ANT1
GO:0031903	microbody membrane	21	7	2.15	0.00367	KLMA_10026,PXA1,PEX11,PXA2,PEX25,INP1,ANT1
GO:0005886	plasma membrane	145	25	14.88	0.00573	PLB,STE3,PMA1,KLMA_10486,FRQ1,MEP2,HSP12,STE4,TOK1,MSB2,ENA5,KLMA_40213,PDR5,GAS4,PUN1,APS2,QDR3,GPA1,STL1,SST2,KLMA_70118,AGP2,PHO84,AVT7,SFK1
GO:0005618	cell wall	34	9	3.49	0.00581	KLMA_20368,KLMA_40061,GAS4,FIG1,PIR1,CRR1,KLMA_70011,ATH1,KLMA_80053
GO:0030312	external encapsulating structure	34	9	3.49	0.00581	KLMA_20368,KLMA_40061,GAS4,FIG1,PIR1,CRR1,KLMA_70011,ATH1,KLMA_80053
GO:0031234	extrinsic component of cytoplasmic side ...	5	3	0.51	0.00915	STE4,GPA1,KLMA_70118

GO terms enriched in significantly down-regulated genes (molecular functions)

GO.ID	Term	Annotated gene	Significant	Expected	P-value	Genes
GO:0030515	snoRNA binding	24	17	2.53	3.10E-12	UTP13,UTP15,ENP1,UTP8,NAN1,PWP2,UTP10,GAR1,IMP4,BMS1,UTP6,RRP5,DIP2,IMP3,NOP14,RRP9,UTP25
GO:0003723	RNA binding	365	71	38.55	5.10E-08	SRO9,NOP1,PNO1,RPF2,RSE1,HAS1,UTP13,DBP7,UTP15,NOP53,RPS2,NOP58,NOP12,ENP1,UBP3,CBF5,UTP8,TPA1,DTD1,RPS3,RPL5,NAN1,TEF3,MEU1,NOB1,PAB1,RLI1,NSR1,MEX67,PWP2,TIF32,VTS1,BRE5,UTP10,GAR1,CIC1,ERB1,IMP4,PUS1,BMS1,RPL10A,UTP6,MTR4,TIF4632,RRP5,NUG1,NOP7,DIP2,MAP1,IMP3,BRX1,TEF4,FUN12,NOP15,CEX1,NOP4,PFK2,PET309,IKI3,NOP14,TIF3,RRP9,PUS4,NOP9,PUB1,UTP25,NOP2,NIP1,PUF6,PIN4,ILS1
GO:0019843	rRNA binding	36	16	3.8	1.70E-07	RPF2,NOP53,RPS2,NOP12,RPL5,NOB1,CIC1,ERB1,IMP4,RRP5,NOP7,IMP3,BRX1,NOP15,NOP4,UTP25
GO:0016705	oxidoreductase activity, acting on paire...	27	13	2.85	8.30E-07	OLE1,ERG3,TPA1,CYP707A7,LIA1,ERG1,SUR2,dsd1,fmo1,ERG25,PDAT9,cyp524A1,JHD2
GO:0016491	oxidoreductase activity	293	56	30.95	3.10E-06	HIS4,GPD1,OLE1,GAL80,KYE1,SCS7,URA1,ADH4,MET13,ERG3,TPA1,CYP707A7,TDA3,YPR1,LIA1,RNR2,ERG1,KLMA_30274,SUR2,SER3,PAN5,ADH1,dsd1,HEM13,fmo1,GAP1,ADH2,RIB7,FMS1,ALD5,HMG1,DUS3,MET5,FAS1,DLD1,MTR4,IMD4,ERV1,PDX3,CCP1,ERG25,MET10,ADE3,MDH1,FET3,PDAT9,cyp524A1,JHD2,SMM1,CFL1,RNR1,FAS2,MET16,HEM14,MIS1,GAP3
GO:0097159	organic cyclic compound binding	1315	174	138.89	4.00E-05	APA2,SRO9,HIS4,SAM2,RPA49,KLMA_10146,GPD1,NOP1,GSP1,OLE1,PNO1,GUA1,PFK1,TIF1,RPF2,RSE1,KYE1,MES1,HAS1,TDA1,YTA7,FAU1,PGK,SCS7,PRY2,MET17,MSW1,bioA,NOG1,KLMA_20090,UTP13,DBP7,UTP15,HSL1,ADK1,KLMA_20186,NOP53,RPS2,SHM2,NOP58,GAL1,FHL1,CYS3,NOP12,CCC2,ENP1,UBP3,CBF5,KLMA_20481,UTP8,TPA1,CYP707A7,ADE2,DTD1,RPS3,RPL5,NAN1,HAM1,TEF3,ERG1,MEU1,DRS1,NOB1,PAB1,RLI1,PDC2,ALK2,NSR1,MEX67,SER3,PWP2,DBP3,RRP3,TIF32,HAL9,VTS1,RAD54,KSS1,SAH1,ADE5,7,MTO1,GUS1,BRE5,ECM16,fmo1,MDN1,GAP1,RIB7,UTP10,GAR1,CIC1,HAP1,ERB1,MCM1,IMP4,MAM33,HRK1,HMG1,SFP1,DUS3,RPC82,MET5,PUS1,BMS1,RPL10A,UTP6,DLD1,PRP43,GEP3,SHM1,MTR4,KLMA_50101,TIF4632,RPA135,PTK2,PDX3,CCP1,RRP5,

GO.ID	Term	Annotated gene	Significant	Expected	P-value	Genes
						LAC9,NUG1,RPC40,VAS1,NOP7,DIP2,ACS2,MAP1,URA2,IMP3,PDC1,ADE3,EXO1,BRX1,TEF4,DHR2,RPA190,FUN12,REV1,NOP15,CYB5,CEX1,AZF1,KRE33,RGT1,SKS1,NOP4,PDAT9,RAG5,cyp524A1,PFK2,SMM1,RPB1,CDC60,PET309,IKI3,URA7,RNR1,NEW1,NOP14,TIF3,RRP9,MIS1,PUS4,KLMA_70408,NOP9,GAP3,MET3,PUB1,UTP25,NOP2,NIP1,PUF6,PIN4,DOT6,ILS1
GO:1901363	heterocyclic compound binding	1314	173	138.79	6.10E-05	APA2,SRO9,HIS4,SAM2,RPA49,KLMA_10146,GPD1,NOP1,GSP1,OLE1,PNO1,GUA1,PFK1,TIF1,RPF2,RSE1,KYE1,MES1,HAS1,TDA1,YTA7,FAU1,PGK,SCS7,MET17,MSW1,bioA,NOG1,KLMA_20090,UTP13,DBP7,UTP15,HSL1,ADK1,KLMA_20186,NOP53,RPS2,SHM2,NOP58,GAL1,FHL1,CYS3,NOP12,CCC2,ENP1,UBP3,CBF5,KLMA_20481,UTP8,TPA1,CYP707A7,ADE2,DTD1,RPS3,RPL5,NAN1,HAM1,TEF3,ERG1,MEU1,DRS1,NOB1,PAB1,RLI1,PDC2,ALK2,NSR1,MEX67,SER3,PWP2,DBP3,RRP3,TIF32,HAL9,VTS1,RAD54,KSS1,SAH1,ADE5,7,MTO1,GUS1,BRE5,ECM16,fmo1,MDN1,GAP1,RIB7,UTP10,GAR1,CIC1,HAP1,ERB1,MCM1,IMP4,MAM33,HRK1,HMG1,SFP1,DUS3,RPC82,MET5,PUS1,BMS1,RPL10A,UTP6,DLD1,PRP43,GEP3,SHM1,MTR4,KLMA_50101,TIF4632,RPA135,PTK2,PDX3,CCP1,RRP5,LAC9,NUG1,RPC40,VAS1,NOP7,DIP2,ACS2,MAP1,URA2,IMP3,PDC1,ADE3,EXO1,BRX1,TEF4,DHR2,RPA190,FUN12,REV1,NOP15,CYB5,CEX1,AZF1,KRE33,RGT1,SKS1,NOP4,PDAT9,RAG5,cyp524A1,PFK2,SMM1,RPB1,CDC60,PET309,IKI3,URA7,RNR1,NEW1,NOP14,TIF3,RRP9,MIS1,PUS4,KLMA_70408,NOP9,GAP3,MET3,PUB1,UTP25,NOP2,NIP1,PUF6,PIN4,DOT6,ILS1
GO:0005506	iron ion binding	25	10	2.64	0.00012	SCS7,ERG3,TPA1,CYP707A7,RNR2,RLI1,SUR2,ERG25,PDAT9,cyp524A1
GO:0048037	cofactor binding	181	35	19.12	0.00021	HIS4,ACO2,GPD1,OLE1,BIO2,KYE1,SCS7,MET17,bioA,SHM2,GAL10,CYS3,TPA1,CYP707A7,ERG1,SER3,SAH1,MTO1,fmo1,GAP1,RIB7,HMG1,DUS3,MET5,DLD1,SHM1,LEU1,PDX3,CCP1,PDC1,CYB5,PDAT9,cyp524A1,SMM1,GAP3
GO:0034511	U3 snoRNA binding	7	5	0.74	0.00022	NAN1,BMS1,RRP5,RRP9,UTP25

GO.ID	Term	Annotated gene	Significant	Expected	P-value	Genes
GO:0050662	coenzyme binding	120	25	12.67	0.00055	HIS4,GPD1,KYE1,MET17,bioA,SHM2,GAL10,CYS3,TPA1,ERG1,SER3,SAH1,MTO1,fmo1,GAP1,RIB7,HMG1,DUS3,MET5,DLD1,SHM1,PDX3,PDC1,SMM1,GAP3
GO:0070180	large ribosomal subunit rRNA binding	5	4	0.53	0.00056	CIC1,ERB1,NOP7,NOP4
GO:0004497	monooxygenase activity	17	7	1.8	0.00105	CYP707A7,LIA1,ERG1,fmo1,ERG25,PDAT9,cyp524A1
GO:0020037	heme binding	24	8	2.53	0.0023	OLE1,SCS7,CYP707A7,MET5,CCP1,CYB5,PDAT9,cyp524A1
GO:0046906	tetrapyrrole binding	24	8	2.53	0.0023	OLE1,SCS7,CYP707A7,MET5,CCP1,CYB5,PDAT9,cyp524A1
GO:0140098	catalytic activity, acting on RNA	194	33	20.49	0.00319	SPB1,RPA49,NOP1,DSS1,MES1,HAS1,MSW1,RPC37,DTD1,NOB1,MRPL15,DBP3,RRP3,GUS1,ECM16,TRM13,DUS3,RPC82,PRP43,MTR4,RPA135,RPC40,VAS1,KLMA_50502,DHR2,RPA190,SMM1,RPB1,CDC60,NOP2,TRM6,PTH2,ILS1
GO:0016879	ligase activity, forming carbon-nitrogen...	37	10	3.91	0.00388	GUA1,FAU1,GSH1,ADE5,7,ADE6,ACS2,URA2,ADE3,URA7,MIS1
GO:0008097	5S rRNA binding	4	3	0.42	0.00431	RPF2,RPL5,BRX1
GO:0016717	oxidoreductase activity, acting on paire...	4	3	0.42	0.00431	OLE1,ERG3,cyp524A1
GO:0032451	demethylase activity	4	3	0.42	0.00431	TPA1,CYP707A7,JHD2
GO:0003724	RNA helicase activity	21	7	2.22	0.00432	HAS1,DBP3,RRP3,ECM16,PRP43,MTR4,DHR2
GO:0016866	intramolecular transferase activity	17	6	1.8	0.00598	GPM3,GPM1,CBF5,PGM2,PUS1,PUS4
GO:0002161	aminoacyl-tRNA editing activity	8	4	0.84	0.00606	DTD1,VAS1,CDC60,ILS1
GO:0046914	transition metal ion binding	210	34	22.18	0.00625	HIS4,KLMA_10146,SCS7,SCO1,AAH1,ADH4,ERG3,GAL7,CCC2,TPA1,CYP707A7,RNR2,KLMA_30226,RLI1,SUR2,HAL9,KLMA_30614,ADH1,ADH2,SFB3,HAP1,FBA1,UPC2,ERG25,LAC9,APE2,FET3,RPA190,RGT1,PDAT9,cyp524A1,LEU3,KLMA_70408,MET6

GO.ID	Term	Annotated gene	Significant	Expected	P-value	Genes
GO:0003676	nucleic acid binding	786	102	83.02	0.008	SRO9,RPA49,KLMA_10146,NOP1,PNO1,TIF1,RPF2,RSE1,HAS1,UTP13,DBP7,UTP15,KLMA_20186,NOP53,RPS2,NOP58,FHL1,NOP12,ENP1,UBP3,CBF5,KLMA_20481,UTP8,TPA1,DTD1,RPS3,RPL5,NAN1,TEF3,MEU1,DRS1,NOB1,PAB1,RLI1,PDC2,NSR1,MEX67,PWP2,DBP3,RRP3,TIF32,HAL9,VTS1,BRE5,ECM16,UTP10,GAR1,CIC1,HAP1,ERB1,MCM1,IMP4,MAM33,SFP1,RPC82,PUS1,BMS1,RPL10A,UTP6,PRP43,MTR4,KLMA_50101,TIF4632,RPA135,RRP5,LAC9,NUG1,RPC40,NOP7,DIP2,MAP1,IMP3,EXO1,BRX1,TEF4,DHR2,RPA190,FUN12,REV1,NOP15,CEX1,AZF1,RGT1,NOP4,PFK2,RPB1,PET309,IKI3,NOP14,TIF3,RRP9,PUS4,KLMA_70408,NOP9,PUB1,UTP25,NOP2,NIP1,PUF6,PIN4,DOT6,ILS1
GO:0048029	monosaccharide binding	5	3	0.53	0.00993	GAL1,TPA1,RAG5

GO terms enriched in significantly up-regulated genes (molecular functions)

GO.ID	Term	Annotated gene	Significant	Expected	P-value	Genes
GO:0016491	oxidoreductase activity	293	58	34.09	1.50E-05	MXR2,DIT2,ZTA1,KYE1,SOU2,SOU1,yxeK,FRE4,MIOX5,KLMA_20057,POX1,GPD2,ALD2,GDH2,YUC8,CYB2,YPR1,fmo1,KLMA_30124,YIM1,KLMA_30533,ARA1,KLMA_30604,KLMA_40010,LYS9,MDH3,AHP1,LYS1,MDH2,IDP1,adh,ALD4,BDH2,KLMA_50021,sdh,GLT1,SFA1,AIM17,CTT1,PUT1,SPS19,FMP46,ACAD11,gabD,JLP1,KLMA_60268,ETR1,AO-I,KLMA_60405,KLMA_70012,SOR1,ERV2,FOX2,KLMA_70434,KLMA_80176,ADH3,ADH6

GO.ID	Term	Annotated gene	Significant	Expected	P-value	Genes
GO:0003824	catalytic activity	1905	254	221.62	0.00024	KLMA_10012,CHA1,MXR2,ATG26,YAT1,KIN3,CDC7,ECI1,ECM38,HRD1,PLB,URH1,DIT2,HEM4,PMC1,PMA1,PGU1,INU1,ZTA1,KLMA_10551,KLMA_10560,CTK3,KYE1,KLMA_10605,YKT6,RAN1,SOU2,HUL4,RAD26,UBP16,YTA6,GLY1,YDC1,SOU1,PNG1,ECM31,KLMA_10805,DAL3,yxeK,FRE4,YUH1,MAG1,FUS3,MIOX5,KLMA_20057,SAP30,KLMA_20070,CIT1,RTT109,ECM4,DMC1,PXA1,ppr1,HST2,POT1,DSD1,POX1,SLX1,GPD2,IME4,RIM15,AGX1,IMP1,RAM1,RAD55,KLMA_20431,FAB1,AOS1,MUS81,ARO10,IAH1,CTS2,ALD2,MLH1,GDH2,YUC8,PTP2,RNY1,amdS,KLMA_30011,CYB2,bioA,YPR1,ARI1,fmo1,mug157,RNH1,YKU80,KLMA_30124,SPO1,SRS2,GLO4,KLMA_30245,KLMA_30260,KLMA_30282,ACO2,UBC12,PDR12,EXO5,CMK2,TAD2,YIM1,DPP1,DPL1,MLH3,REV3,KLMA_30533,PIB1,GAD1,PRR1,APN1,IME2,ARA1,ACS1,KLMA_30604,FMP48,CAT2,SDT1,KLMA_40003,OXP1,KLMA_40010,LYS9,EST2,KLMA_40050,MDH3,KLMA_40068,KLMA_40105,KLMA_40133,AHP1,UGA1,ENA5,CHS1,SPO14,PDR5,YPS7,ELM1,KLMA_40335,KLMA_40359,LYS1,MDH2,GAS4,IDP1,PXA2,NPY1,adh,KLMA_40628,ALD4,BDH2,KLMA_50021,GDE1,CAR1,VPS21,KLMA_50093,POP4,KLMA_50123,ARO8,DAK1,ITT1,CAR2,ECO1,sdh,GLT1,SFA1,IST3,AIM17,CTT1,KLMA_50451,AXL1,BAR1,INO1,RKM5,PUT1,PCD1,URC1,SPS19,GUD1,KIN28,FMP46,ACAD11,NIT2,TDA10,TES1,YCH1,HSP78,gabD,APC11,GPA1,JLP1,KLMA_60206,KLMA_60268,ETR1,KLMA_60293,AO-I,SCY1,KLMA_60366,RAD1,PHO85,KLMA_60405,PDE1,KLMA_60413,KLMA_60415,ARO9,GRE2,KLMA_60426,FBP1,CRR1,KLMA_70002,KLMA_70012,KIP2,OTU1,SOR1,YMR1,ERV2,ATH1,TEL1,KLMA_70156,SEE1,ICL1,ALG12,KLMA_70270,KLMA_70303,KLMA_70317,LPX1,FAD1,FOX2,ICL2,KLMA_70434,SAD1,PDH1,CIT3,ULA1,KLMA_80004,PHR1,hyuA,KLMA_80053,LYS5,KLMA_80108,KTR5,SIW14,lag1,KLMA_80136,SGA1,NCE103,MSG5,KLMA_80176,UBA3,ADH3,ARO7,ADH6,SAS3,GUT1

GO.ID	Term	Annotated gene	Significant	Expected	P-value	Genes
GO:0022857	transmembrane transporter activity	267	48	31.06	0.00096	PMC1,PMA1,HGT1,RAN1,GDT1,FCY2,NIPA2,PXA1,THI72,YHM2,uapC,MEP2,ODC2,KHA1,PUT4,TOK1,PDR12,KLMA_30601,STL1,KLMA_30672,LAC12,ENA5,PDR5,PXA2,QDR3,RAG1,HAK1,DIP5,KLMA_60323,VCX1,ERC1,HXT15,ywtG,AGP2,PHO84,ANT1,PTR2,SFC1,CRC1,HXT14
GO:0016810	hydrolase activity, acting on carbon-nit...	54	14	6.28	0.00266	YDC1,PNG1,DAL3,SAP30,HST2,amdS,TAD2,OXP1,KLMA_40359,CAR1,URC1,GUD1,KLMA_60206
GO:0015079	potassium ion transmembrane transporter ...	10	5	1.16	0.00318	KHA1,TOK1,ENA5,HAK1,VCX1
GO:0016798	hydrolase activity, acting on glycosyl b...	39	11	4.54	0.00372	URH1,PGU1,INU1,MAG1,CTS2,KLMA_30011,KLMA_40105,CRR1,ATH1,KLMA_70317,SGA1
GO:0005215	transporter activity	314	52	36.53	0.00391	PMC1,PMA1,HGT1,RAN1,GDT1,FCY2,RFT1,NIPA2,PXA1,THI72,YHM2,uapC,MEP2,ODC2,KHA1,PUT4,TOK1,MMM1,PDR12,KLMA_30601,STL1,KLMA_30672,LAC12,ENA5,PDR5,PXA2,KAP122,APS2,QDR3,RAG1,HAK1,DIP5,KLMA_60323,VCX1,ERC1,HXT15,ywtG,AGP2,PHO84,ANT1,PTR2,SFC1,CRC1,HXT14
GO:0050662	coenzyme binding	120	24	13.96	0.00478	CHA1,KYE1,HST2,POX1,GPD2,KLMA_20431,ARO10,YUC8,bioA,ARI1,fmo1,DPL1,GAD1,UGA1,IDP1,KLMA_40628,ARO8,CAR2,GLT1,KLMA_50437,ACAD11,ARO9,GRE2,KLMA_80004
GO:0048037	cofactor binding	181	33	21.06	0.00484	CHA1,DIT2,KLMA_10520,KYE1,HST2,POX1,GPD2,KLMA_20431,ARO10,YUC8,ISU1,bioA,ARI1,fmo1,ACO2,DPL1,REV3,GAD1,ISA1,UGA1,IDP1,KLMA_40628,ARO8,CAR2,GLT1,CTT1,KLMA_50437,ACAD11,AO-I,ARO9,GRE2,PDH1,KLMA_80004
GO:0046914	transition metal ion binding	210	37	24.43	0.00527	YHC1,DIT2,ZTA1,MIOX5,GSM1,HST2,ERT1,KLMA_20292,ISU1,RDS2,mlo2,SIP4,KLMA_30395,TAD2,KLMA_30428,KLMA_30533,APN1,OAF1,GAT2,ISA1,adh,THI4,BDH2,CAR1,GLT1,SFA1,KLMA_50329,GUD1,ETP1,AO-I,SOR1,KLMA_70242,SAD1,KLMA_80008,NCE103,ADH3,ADH6
GO:0004622	lysophospholipase activity	4	3	0.47	0.00571	PLB,SPO1,KLMA_30260
GO:0016701	oxidoreductase activity, acting on	4	3	0.47	0.00571	MIOX5,KLMA_30533,KLMA_30604

GO.ID	Term	Annotated gene	Significant	Expected	P-value	Genes
	singl...					
GO:0016829	lyase activity	73	16	8.49	0.00821	CHA1,HEM4,GLY1,DAL3,DSD1,ARO10,ACO2,DPL1,GAD1,APN1,ICL1,FOX2,ICL2,PDH1,PHR1,NCE103
GO:0004553	hydrolase activity, hydrolyzing O-glycos...	32	9	3.72	0.00863	PGU1,INU1,CTS2,KLMA_30011,KLMA_40105,CRR1,ATH1,KLMA_70317,SGA1
GO:0008270	zinc ion binding	168	30	19.54	0.00958	YHC1,ZTA1,GSM1,HST2,ERT1,KLMA_20292,RDS2,mlo2,SIP4,KLMA_30395,TAD2,KLMA_30428,KLMA_30533,APN1,OAF1,GAT2,adh,BDH2,CAR1,SFA1,KLMA_50329,GUD1,ETP1,SOR1,KLMA_70242,SAD1,KLMA_80008,NCE103,ADH3,ADH6
GO:0004497	monooxygenase activity	17	6	1.98	0.00962	DIT2,yxeK,YUC8,fmo1,KLMA_30604

Supplementary File S3. KEGG mapper analysis of differentially expressed genes for *Kmmig1* and the parental strain of *K. marxianus*

KEGG Mapper analysis of down-regulated DEGs

No	Code	Pathway	Genes number	Gene name
1	kmx01100	Metabolic pathways	100	APA2, HIS4, SAM2, ACO2A, PRS5, GPM3, HPT1, GUA1, PFK1, BIO2, ENO, FAU1, PGK, MET17, FUR1, AAH1, RAG2, URA1, TPS2, TSL1, GPM1, ADH4b, ADK1, MET13, CYS4, SHM2, ERG3, GAL7, GAL10, GAL1, CYS3, CYP707A7, ADE2, YPR1, ERG9, PHO3, HAM1, PGM2, GSH1, PHS1, RNR2, ERG1, MEU1, KLMA_30226, GSY2, SUR2, SER3, PAN5, ERG6, SAH1, DUG1, ADE5, GUS1, ADH1, dsd1, HEM13, TPI1, LCB5, GAP1, ADH2, RIB7, FBA1, ALD5, HMG1, DPM1, MET5, FAS1, GUK1, SHM1, LEU1, IMD4, YJU3, PDX3, SUR4, ADE6, ERG25, MET10, ACS2, URA2, PDC1, ADE3, MDH1, PYK1, LEU4, UGP1, RAG5, cyp524A1, ERG13, PFK2, URA7, RNR1, RHR2, FAS2, MET16, HEM14, MIS1, GAP3, MET3, TPS1, MET6
2	kmx01110	Biosynthesis of secondary metabolites	52	HIS4, LPP1, SAM2, PRS5, GPD1, GPM3, HPT1, PFK1, ENO, PGK, MET17, RAG2, GPM1, ADH4b, ADK1, SHM2, ERG3, GAL10, CYP707A7, ADE2, ERG9, PGM2, PHS1, ERG1, PAN5, ERG6, ADE5, GUS1, ADH1, HEM13, TPI1, GAP1, ADH2, FBA1, ALD5, HMG1, SHM1, LEU1, IMD4, SUR4, ADE6, ACS2, PDC1, MDH1, PYK1, LEU4, RAG5, ERG13, PFK2, HEM14, GAP3, MET6
3	kmx01130	Biosynthesis of antibiotics	43	ACO2a, PRS5, GPM3, PFK1, ENO, PGK, MET17, RAG2, GPM1, ADH4b, ADK1, CYS4, SHM2, ERG3, GAL10, CYS3, CYP707A7, ERG9, PGM2, ERG1, SER3, ERG6, ADE5, ADH1, TPI1, GAP1, ADH2, FBA1, ALD5, HMG1, SHM1, ADE6, ERG25, ACS2, MDH1, PYK1, UGP1, RAG5, cyp524A1, ERG13, PFK2, GAP3, MET3
4	kmx03008	Ribosome biogenesis in eukaryotes	31	NOP1, GSP1, UTP5, NOG1, UTP13, UTP15, NOP58, CBF5, UTP8, MPP10, NAN1, NOB1, MEX67, PWP2, MDN1, UTP10, GAR1, IMP4, NMD3, UTP21, MTR2, BMS1, UTP6, UTP18, NOP56, UTP4, NUG1, DIP2, IMP3, KRE33, NOP4
5	kmx01230	Biosynthesis of amino acids	24	HIS4, SAM2, ACO2a, PRS5, GPM3, PFK1, ENO, PGK, MET17, GPM1, CYS4, SHM2, CYS3, SER3, TPI1, GAP1, FBA1, SHM1, LEU1, PYK1, LEU4, PFK2, GAP3, MET6
6	kmx01200	Carbon metabolism	21	PRS5, GPM3, PFK1, ENO, PGK, MET17, RAG2, GPM1, MET13, SHM2, SER3, TPI1, GAP1, FBA1, SHM1, ACS2, MDH1, PYK1, RAG5,PFK2, GAP3
7	kmx00010	Glycolysis / Gluconeogenesis	21	GPM3, PFK1, ENO, PGK, RAG2, GPM1, ADH4, GAL10, PGM2, ADH1, TPI1, GAP1, ADH2, FBA1, ALD5, ACS2, PDC1, PYK1, RAG5, PFK2, GAP3
8	kmx03010	Ribosome	19	RPL37a, RPL17B, RPL24, RPS2, KLMA_20355, RLP7, RPL22A, RPS3, RPL5, RPL3, RPL15B, RPL10A, RPS14, RPL2, KLMA_60069, KLMA_60313, RPL19B, RLP24, RPL8B
9	kmx00230	Purine metabolism	17	APA2, PRS5, HPT1, GUA1, AAH1, ADK1, ADE2, HAM1, PGM2, RNR2, ADE5, GUK1, IMD4, ADE6, PYK1, RNR1, MET3

No	Code	Pathway	Genes number	Gene name
10	kmx03013	RNA transport	13	GSP1, TIF1, PAB1, MEX67, TIF32, NUP116, NMD3, MTR2, TIF4632, FUN12, HSL7, TIF3, NIP1
11	kmx00680	Methane metabolism	10	GPM3, PFK1, ENO, GPM1, SHM2, SER3, FBA1, SHM1, ACS2, PFK2
12	kmx00500	Starch and sucrose metabolism	9	RAG2, TPS2, TSL1, PGM2, GSY2, UGP1, RAG5, BGL2, TPS1
13	kmx00270	Cysteine and methionine metabolism	9	SAM2, MET17, CYS4, CYS3, GSH1, MEU1, SAH1, MDH1, MET6
14	kmx04011	MAPK signaling pathway	8	SH3, GPD1, HSL1, CLN2, KSS1, MCM1, HSL7, KLMA_80303
15	kmx00052	Galactose metabolism	8	PFK1, GAL7, GAL10, GAL1, PGM2, UGP1, RAG5, PFK2
16	kmx03020	RNA polymerase	8	RPA49, RPC37, RPC82, RPA135, RPC40, KLMA_50426, KLMA_50502, RPA190, RPB1
17	kmx04111	Cell cycle	8	TAH11, HSL1, CLN2, KLMA_20481, MCM1, LTE1, HSL7, PCL1
18	kmx00260	Glycine, serine and threonine metabolism	7	GPM3, GPM1, CYS4, SHM2, CYS3, SER3, SHM1
19	kmx00100	Steroid biosynthesis	7	ERG3, CYP707A7, ERG9, ERG1, ERG6, ERG25, CYP524A1
20	kmx00520	Amino sugar and nucleotide sugar metabolism	7	RAG2, GAL7, GAL10, GAL1, PGM2, UGP1, RAG5
21	kmx00030	Pentose phosphate pathway	6	PRS5, PFK1, RAG2, PGM2, FBA1, PFK2
22	kmx00670	One carbon pool by folate	6	FAU1, MET13, SHM2, SHM1 ADE3, MIS1
23	kmx00920	Sulfur metabolism	6	APA2, MET17, MET5, MET10, MET16, MET3
24	kmx00970	Aminoacyl-tRNA biosynthesis	6	MES1, MSW1, GUS1, VAS1, CDC60, ILS1
25	kmx00620	Pyruvate metabolism	6	ALD5, DLD1, ACS2, MDH1, PYK1, LEU4
26	kmx01212	Fatty acid metabolism	6	OLE1, KLMA_20392, PHS1, FAS1, SUR4, FAS2
27	kmx00240	Pyrimidine metabolism	6	FUR1, URA1, RNR2, URA2, URA7, RNR1
28	kmx03018	RNA degradation	6	PFK1, ENO, PAB1, MTR4, PFK2, RRP42
29	kmx00561	Glycerolipid metabolism	5	LPP1, YPR1, ALD5, YJU3, RHR2
30	kmx00051	Fructose and mannose metabolism	5	PFK1, PFK2, RAG5, TPI1, FBA1
31	kmx00450	Selenocompound metabolism	4	MES1, CYS3, MET3, MET6
32	kmx00480	Glutathione metabolism	4	GSH1, RNR2, DUG1, RNR1
33	kmx00071	Fatty acid degradation	4	ADH4b, ADH1, ADH2, ALD5
34	kmx04113	Meiosis	4	TAH11, CLN2, KHT2, RAG1
35	kmx00630	Glyoxylate and dicarboxylate	4	SHM1, SHM2, ACS2, MDH1

No	Code	Pathway	Genes number	Gene name
		metabolism		
36	kmx00600	Sphingolipid metabolism	4	SUR2, dsd1, LCB5, KLMA_80180
37	kmx01040	Biosynthesis of unsaturated fatty acids	4	OLE1, KLMA_20392, PHS1, SUR4
38	kmx00860	Porphyrin and chlorophyll metabolism	3	GUS1, HEM13, HEM14
39	kmx01210	2-Oxocarboxylic acid metabolism	3	ACO2a, LEU1, LEU4
40	kmx00350	Tyrosine metabolism	3	ADH1, ADH2, ADH4b
41	kmx03015	mRNA surveillance pathway	3	PAB1, MEX67, MTR2
42	kmx00190	Oxidative phosphorylation	2	KLMA_30226, IPP1
43	kmx00740	Riboflavin metabolism	2	PHO3, RIB7
44	kmx00340	Histidine metabolism	2	HIS4, ALD5
45	kmx00564	Glycerophospholipid metabolism	2	LPP1, GPD1
46	kmx00290	Valine, leucine and isoleucine biosynthesis	2	LEU1, LEU4
47	kmx00280	Valine, leucine and isoleucine degradation	2	ALD5, ERG13
48	kmx03040	Spliceosome	2	RSE1, PRP43
49	kmx00730	Thiamine metabolism	2	ADK1, PHO3
50	kmx00062	Fatty acid elongation	2	PHS1, SUR4
51	kmx00061	Fatty acid biosynthesis	2	FAS1, FAS2
52	kmx00900	Terpenoid backbone biosynthesis	2	HMG1, ERG13
53	kmx00410	beta-Alanine metabolism	2	FMS1, ALD5
54	kmx00909	Sesquiterpenoid and triterpenoid biosynthesis	2	ERG9, ERG1
55	kmx00330	Arginine and proline metabolism	2	FMS1, ALD5
56	kmx00460	Cyanoamino acid metabolism	2	SHM1, SHM2
57	kmx04139	Mitophagy	2	UBP3, BRE5
58	kmx00310	Lysine degradation	1	ALD5
59	kmx00300	Lysine biosynthesis	1	ACO2a
60	kmx00380	Tryptophan metabolism	1	ALD5

No	Code	Pathway	Genes number	Gene name
61	kmx00780	Biotin metabolism	1	BIO2
62	kmx00640	Propanoate metabolism	1	ACS2
63	kmx03440	Homologous recombination	1	RAD54
64	kmx00770	Pantothenate and CoA biosynthesis	1	PAN5
65	kmx00650	Butanoate metabolism	1	ERG13
66	kmx03430	Mismatch repair	1	EXO1
67	kmx00250	Alanine, aspartate and glutamate metabolism	1	URA2
68	kmx03060	Protein export	1	IMP2
69	kmx04933	AGE-RAGE signaling pathway in diabetic complications	1	KSS1
70	kmx04141	Protein processing in endoplasmic reticulum	1	SFB3
71	kmx00072	Synthesis and degradation of ketone bodies	1	ERG13
72	kmx00750	Vitamin B6 metabolism	1	PDX3
73	kmx00261	Monobactam biosynthesis	1	MET3
74	kmx00562	Inositol phosphate metabolism	1	TPI1
75	kmx00020	Citrate cycle (TCA cycle)	1	MDH1
76	kmx00510	N-Glycan biosynthesis	1	DPM1
77	kmx00040	Pentose and glucuronate interconversions	1	UGP1
78	kmx00053	Ascorbate and aldarate metabolism	1	ALD5

KEGG Mapper analysis of up-regulated DEGs

No	Code	Pathway	Genes number	Gene name
1	kmx01100	Metabolic pathways	80	KLMA_10012, CHA1, ECM38, HEM4, PGU1, INU1, GLY1, ECM31, DAL3, KLMA_20057, CIT1, ppr1, HST2, POT1, GPD2, AGX1, FAB1, ARO10, CTS2, ALD2, GDH2, KLMA_30011, CYB2, YPR1, KLMA_30124, KLMA_30282, ACO2b, KLMA_30365, DPL1, GAD1, ACS1, TH113, OXP1, LYS9, MDH3, UGA1, SPO14, KLMA_40359, LYS1, MDH2, IDP1, NPY1, THI4, ALD4, CAR1, ARO8, DAK1, CAR2, sdh, GLT1, SFA1, KLMA_50451, INO1, PUT1, GUD1, ACAD11, KLMA_60039, TDA10, TES1, gabD, KLMA_60206, ETR1, KLMA_60327, ARO9, FBP1, YMR1, ATH1, ICL1, ALG12, FAD1, ICL2, CIT3, lag1, KLMA_80136, SGA1, ADH3, ARO7, ADH6, GUT1
2	kmx01110	Biosynthesis of secondary metabolites	45	CHA1, HEM4, GLY1, ECM31, CIT1, ppr1, POT1, POX1, GPD2, AGX1, ALD2, KLMA_30011, KLMA_30124, ACO2b, DPP1, GAD1, ACS1, LYS9, MDH3, SPO14, LYS1, MDH2, IDP1, ALD4, CAR1, ARO8, CAR2, GLT1, SFA1, CIT1, PUT1, dpp1, ACAD11, TDA10, TES1, KLMA_60327, ARO9, FBP1, ICL1, FAD1, ICL2, CIT3, ADH3, ARO7, ADH6
3	kmx01130	Biosynthesis of antibiotics	33	CHA1, GLY1, CIT1, POT1, GPD2, AGX1, RAM1, KLMA_30124, ACO2b, ACS1, LYS9, MDH3, LYS1, MDH2, IDP1, ALD4, CAR1, ARO8, CAR2, GLT1, SFA1, CTT1, KLMA_50451, INO1, PUT1, ACAD11, TDA10, ARO9, FBP1, CIT3, ADH3, ARO7, ADH6
4	kmx01200	Carbon metabolism	19	CHA1, CIT1, GPD2, AGX1, KLMA_30124, ACO2b, ACS1, MDH3, MDH2, IDP1, DAK1, SFA1, CTT1, ACAD11, TDA10, FBP1, ICL1, ICL2, CIT3
5	kmx04138	Autophagy	18	ATG12, ATG27, YKT6, ATG17, RIM15, ATG14, ATG1, VTI1, KLMA_30673, ELM1, ATG4, PHO85, ATG3, YMR1, KLMA_70233, ATG9, VAM3, ATG8
6	kmx04011	MAPK signaling pathway	15	STE3, FUS3, STE4, PTP2, MSB2, CTT1, BAR1, GPA1, KLMA_60356, KLMA_60404, SST2, KLMA_70118, TEC1, SSK1, MSG5
7	kmx01230	Biosynthesis of amino acids	13	CHA1, GLY1, CIT1, GPD2, ACO2b, LYS9, LYS1, IDP1, CAR1, ARO8, GLT1, CIT3, ARO7
8	kmx04113	Meiosis	13	CDC7, SSP1, KLMA_10523, DMC1, RIM15, SPS4, ZIP1, IME2, REC8, RME1, HOP1, APC2, APC11
9	kmx04146	Peroxisome	12	YAT1, ECI1, PXA1, POT1, POX1, AGX1, CAT2, IDP1, PXA2, NPY1, CTT1, SPS19
10	kmx00630	Glyoxylate and dicarboxylate metabolism	11	CIT1, AGX1, ACO2b, ACS1, MDH3, MDH2, CTT1, TDA10, ICL1, ICL2, CIT3
11	kmx03040	Spliceosome	9	KLMA_10570, SMD1, CUS1, SYF2, SNU23, KLMA_40041, LEA1, PRP45, SAD1
12	kmx00071	Fatty acid degradation	8	ECI1, DIT2, POT1, POX1, ALD4, SFA1, ACAD11, ADH3
13	kmx00010	Glycolysis / Gluconeogenesis	8	GPD2, ALD2, ACS1, ALD4, SFA1, FBP1, ADH3, ADH6
14	kmx00561	Glycerolipid metabolism	8	YPR1, DPP1, ALD4, DAK1, dpp1, TDA10, ADH6, GUT1

No	Code	Pathway	Genes number	Gene name
15	kmx04144	Endocytosis	8	VTA1, IST1, VPS60, SNX4, SPO14, KLMA_50160, APS2, SNF8
16	kmx00330	Arginine and proline metabolism	8	ppr1, KLMA_20834, KLMA_40359, ALD4, CAR1, CAR2, PUT1, KLMA_80318
17	kmx00020	Citrate cycle (TCA cycle)	7	CIT1, KLMA_30124, ACO2b, MDH3, MDH2, IDP1, CIT3
18	kmx00260	Glycine, serine and threonine metabolism	7	CHA1, GLY1, DSD1, AGX1, sdh, TDA10, KLMA_60327
19	kmx00350	Tyrosine metabolism	7	ALD2, ARO8, SFA1, gabD, KLMA_60327, ARO9, ADH3
20	kmx00360	Phenylalanine metabolism	7	ARO10, ALD2, KLMA_20834, ARO8, KLMA_60327, ARO9, KLMA_80318
21	kmx04136	Autophagy	6	ATG12, ATG1, ATG4, ATG3, ATG9, ATG8
22	kmx00620	Pyruvate metabolism	6	CYB2, GLO4, ACS1, MDH3, MDH2, ALD4
23	kmx04120	Ubiquitin mediated proteolysis	6	HRD1, AOS1, UBC12, APC2, APC11, UBA3
24	kmx00760	Nicotinate and nicotinamide metabolism	6	URH1, HST2, KLMA_30282, SDT1, NPY1, gabD
25	kmx00380	Tryptophan metabolism	6	DIT2, KLMA_20834, ALD4, ARO8, CTT1, KLMA_80318
26	kmx00410	beta-Alanine metabolism	6	ALD2, GAD1, UGA1, ALD4, ACAD11, KLMA_60327
27	kmx00250	Alanine, aspartate and glutamate metabolism	6	AGX1, GDH2, GAD1, UGA1, GLT1, gabD
28	kmx01210	2-Oxocarboxylic acid metabolism	5	CIT1, ACO2b, IDP1, ARO8, CIT3
29	kmx03420	Nucleotide excision repair	5	RAD26, SRS2, DPB3, KIN28, RAD1
30	kmx00564	Glycerophospholipid metabolism	5	PLB, DPP1, SPO14, GDE1, dpp1
31	kmx04139	Mitophagy	5	MMM1, ATG1, ATG32, SSK1, ATG8
32	kmx00230	Purine metabolism	5	DAL3, KLMA_20057, GUD1, KLMA_60206, PDE1
33	kmx00680	Methane metabolism	5	AGX1, ACS1, DAK1, SFA1, FBP1
34	kmx04141	Protein processing in endoplasmic reticulum	4	HRD1, PNG1, HSP26, OTU1
35	kmx00480	Glutathione metabolism	4	KLMA_10012, ECM38, OXP1, IDP1
36	kmx00051	Fructose and mannose metabolism	4	SOU2, SOU1, DAK1, FBP1
37	kmx04130	SNARE interactions in vesicular transport	4	YKT6, VTI1, KLMA_30673, VAM3
38	kmx00640	Propanoate metabolism	4	ACS1, UGA1, ACAD11, PDH1
39	kmx04111	Cell cycle	4	CDC7, APC2, APC11, PHO85

No	Code	Pathway	Genes number	Gene name
40	kmx01212	Fatty acid metabolism	4	POT1, POX1, ACAD11, ETR1
41	kmx00500	Starch and sucrose metabolism	4	INU1, KLMA_30011, ATH1, SGA1
42	kmx04213	Longevity regulating pathway	4	HST2, RIM15, CTT1, HSP78
43	kmx00280	Valine, leucine and isoleucine degradation	4	POT1, UGA1, ALD4, ACAD11
44	kmx00562	Inositol phosphate metabolism	4	MIOX5, FAB1, INO1, YMR1
45	kmx00270	Cysteine and methionine metabolism	4	CHA1, MDH3, MDH2, ARO8
46	kmx00650	Butanoate metabolism	4	GAD1, UGA1, BDH2, gabD
47	kmx00310	Lysine degradation	4	LYS9, LYS1, ALD4, gabD
48	kmx00910	Nitrogen metabolism	3	GDH2, GLT1, NCE103
49	kmx00400	Phenylalanine, tyrosine and tryptophan biosynthesis	3	ARO8, ARO9, ARO7
50	kmx03410	Base excision repair	3	MAG1, APN1, DPB3
51	kmx00430	Taurine and hypotaurine metabolism	3	ECM38, GDH2, GAD1
52	kmx03430	Mismatch repair	3	MLH1, SRS2, MLH3
53	kmx03440	Homologous recombination	3	RAD55, MUS81, TEL1
54	kmx01040	Biosynthesis of unsaturated fatty acids	3	POT1, POX1, TES1
55	kmx02010	ABC transporters	3	PXA1, PDR5, PXA2
56	kmx00600	Sphingolipid metabolism	3	YDC1, DPL1, lag1
57	kmx00300	Lysine biosynthesis	3	LYS9, LYS1, ARO8
58	kmx04070	Phosphatidylinositol signaling system	3	FAB1, KLMA_40476, YMR1
59	kmx03030	DNA replication	2	RNH1, DPB3
60	kmx00062	Fatty acid elongation	2	TES1, ETR1
61	kmx00592	alpha-Linolenic acid metabolism	2	POT1, POX1
62	kmx00240	Pyrimidine metabolism	2	URH1, sdh
63	kmx00460	Cyanoamino acid metabolism	2	ECM38, KLMA_30011
64	kmx00340	Histidine metabolism	2	ALD2, ALD4
65	kmx00190	Oxidative phosphorylation	2	PMA1, KLMA_30124
66	kmx00040	Pentose and glucuronate	2	PGU1, ADH6

No	Code	Pathway	Genes number	Gene name
		interconversions		
67	kmx00770	Pantothenate and CoA biosynthesis	2	ECM31, LYS5
68	kmx00053	Ascorbate and aldarate metabolism	2	MIOX5, ALD4
69	kmx00563	Glycosylphosphatidylinositol (GPI)-anchor biosynthesis	2	KLMA_60039, KLMA_80136
70	kmx00520	Amino sugar and nucleotide sugar metabolism	2	CTS2, CHS1
71	kmx00730	Thiamine metabolism	2	THI13, THI4
72	kmx03022	Basal transcription factors	2	TFG2, KIN28
73	kmx00220	Arginine biosynthesis	2	GDH2, CAR1
74	kmx00030	Pentose phosphate pathway	1	FBP1
75	kmx00290	Valine, leucine and isoleucine biosynthesis	1	CHA1
76	kmx00860	Porphyrin and chlorophyll metabolism	1	HEM4
77	kmx00510	N-Glycan biosynthesis	1	ALG12
78	kmx00052	Galactose metabolism	1	INU1
79	kmx00130	Ubiquinone and other terpenoid-quinone biosynthesis	1	ARO8
80	kmx00740	Riboflavin metabolism	1	FAD1
81	kmx04122	Sulfur relay system	1	AHP1
82	kmx03450	Non-homologous end-joining	1	YKU80
83	kmx04392	Hippo signaling pathway	1	TEC1
84	kmx04145	Phagosome	1	FAB1
85	kmx03060	Protein export	1	IMP1
86	kmx03008	Ribosome biogenesis in eukaryotes	1	POP4
87	kmx00565	Ether lipid metabolism	1	SPO14
88	kmx00750	Vitamin B6 metabolism	1	KLMA_30365
89	kmx03013	RNA transport	1	POP4
90	kmx00311	Penicillin and cephalosporin biosynthesis	1	KLMA_50451

No	Code	Pathway	Genes number	Gene name
91	kmx04933	AGE-RAGE signaling pathway in diabetic complications	1	FUS3
92	kmx00900	Terpenoid backbone biosynthesis	1	RAM1
93	kmx00513	Various types of N-glycan biosynthesis	1	ALG12
94	kmx00052	Galactose metabolism	1	INU1
95	kmx03013	RNA transport	1	POP4
96	kmx00740	Riboflavin metabolism	1	FAD1

Supplementary File S4. Unique exon reads and fold changes of genes for *Kmmig1* and the parental strain of *K. marxianus*

Locus_tag	UniProt_gene	Product	Unique exon reads						log_2 Fold Change
			KmWT.1	KmWT.2	KmWT.3	Kmmig1.1	Kmmig1.2	Kmmig1.3	
KLMA_10001	TY2B-GR2	RVT_2 super family	81.13	61.53	105.98	169.89	174.39	217.82	1.18
KLMA_10002		hypothetical protein	0.00	2.24	0.00	0.00	4.21	5.19	2.04
KLMA_10004	Nqo2	ribosyldihydronicotinamide dehydrogenase [quinone]	111.69	130.88	84.05	69.81	80.04	74.33	-0.54
KLMA_10005	BIO5	7-keto 8-aminopelargonic acid transporter	103.46	100.68	102.32	109.34	51.39	51.00	-0.53
KLMA_10006	BIO4	dethiobiotin synthetase	32.92	17.90	32.89	16.82	20.22	14.69	-0.69
KLMA_10007	BIO3	adenosylmethionine-8-amino-7-oxononanoate aminotransferase	179.89	76.07	183.94	248.11	130.58	150.40	0.27
KLMA_10008	ARN2	siderophore iron transporter ARN2	190.47	69.36	124.25	773.77	248.53	203.12	1.67
KLMA_10009		drug resistance protein YOR378W	324.50	314.34	263.12	695.56	320.14	290.42	0.53
KLMA_10010	ARN2	siderophore iron transporter ARN2	427.97	303.15	384.93	1301.96	566.99	542.81	1.11
KLMA_10011	FEN2	probable transporter SEO1	184.59	136.48	276.51	312.87	133.11	171.14	0.05
KLMA_10012		uncharacterized protein C11D3.14c	54.08	35.80	73.09	364.18	155.02	191.89	2.13
KLMA_10013		hypothetical protein	0.00	0.00	0.00	18.50	1.68	9.51	5.52
KLMA_10014	MATALPHA1	mating-type protein ALPHA1	9.41	0.00	0.00	336.42	158.39	191.89	6.19
KLMA_10015	HMLALPHA2	mating-type protein ALPHA2	16.46	0.00	2.44	296.89	275.49	217.82	5.39
KLMA_10016	HMLALPHA3	mating-type protein ALPHA3	29.39	2.24	2.44	1198.51	609.95	649.13	6.17
KLMA_10018	CHA1	catabolic L-serine/threonine dehydratase	49.38	34.68	51.16	196.81	69.93	100.27	1.44
KLMA_10020	VAC17	hypothetical protein	626.66	413.90	554.25	281.75	423.77	430.45	-0.49
KLMA_10021	MRC1	mediator of replication checkpoint protein 1	343.31	271.83	305.75	231.29	289.81	266.22	-0.23
KLMA_10022	KRR1	ribosomal RNA	804.20	627.56	735.75	407.07	378.27	423.53	-0.84

Locus_tag	UniProt_gene	Product	Unique exon reads						log$_2$ Fold Change
			KmWT.1	KmWT.2	KmWT.3	Kmmig1.1	Kmmig1.2	Kmmig1.3	
		assembly protein KRR1							
KLMA_10023		hypothetical protein	96.41	83.90	113.29	157.28	133.95	124.47	0.50
KLMA_10024	MOS1	UPF0327 protein AFR743W	166.95	144.31	162.01	238.86	306.66	248.93	0.75
KLMA_10025	PRD1	saccharolysin	572.58	750.61	517.70	758.63	593.10	618.88	0.10
KLMA_10026		hypothetical protein	82.30	105.15	75.52	354.93	243.48	248.93	1.69
KLMA_10027		hypothetical protein	3946.92	4149.07	3802.99	3484.51	2722.04	2578.37	-0.44
KLMA_10028		hypothetical protein	4.70	2.24	2.44	10.09	1.68	6.91	1.00
KLMA_10029	KAR4	karyogamy protein KAR4	44.68	48.10	41.42	227.09	171.87	188.43	2.13
KLMA_10030		CMAS	1093.43	896.04	1104.84	693.03	770.87	962.03	-0.35
KLMA_10031	SPB1	adoMet-dependent rRNA methyltransferase SPB1	1212.18	1096.28	1302.18	552.58	559.40	642.21	-1.04
KLMA_10032	PBN1	protein PBN1	939.41	719.29	847.82	1097.58	904.82	922.27	0.22
KLMA_10033	LRE1	hypothetical protein	435.02	272.95	428.78	550.89	417.87	421.80	0.29
KLMA_10034	QCR7	cytochrome b-c1 complex subunit 7	1219.23	1193.60	1216.91	843.58	1224.12	991.41	-0.25
KLMA_10035	APA2	5',5'''-P-1,P-4-tetraphosphate phosphorylase 2	4434.85	3051.68	4108.74	916.75	1216.54	1240.35	-1.78
KLMA_10036	CAB1	pantothenate kinase	812.43	624.21	761.33	329.69	395.12	397.60	-0.97
KLMA_10037	KRE28	spindle pole body component YDR532C	152.84	134.24	170.54	194.28	221.57	206.58	0.45
KLMA_10038	RBA50	RNA polymerase II-associated protein RBA50	502.04	530.24	557.90	861.24	449.88	527.26	0.21
KLMA_10039		protein SNA2	110.52	180.10	92.58	2891.56	1354.70	1354.44	3.87
KLMA_10040		uncharacterized protein YDR524C-B	9100.14	7717.57	8761.98	9381.17	8773.56	6142.96	-0.07
KLMA_10041		hypothetical protein	79.95	61.53	86.49	112.70	62.34	51.86	0.00
KLMA_10043	SPS1	sporulation-specific protein 1	65.84	81.66	62.12	229.61	65.71	97.67	0.91
KLMA_10044	SPS22	cell wall mannoprotein PST1	44.68	39.15	64.56	249.79	78.35	76.06	1.45

Locus_tag	UniProt_gene	Product	Unique exon reads						$\log_2$ Fold Change
			KmWT.1	KmWT.2	KmWT.3	Kmmig1.1	Kmmig1.2	Kmmig1.3	
KLMA_10045	URC2	uracil catabolism protein 2	701.91	574.99	726.00	629.95	526.55	552.32	-0.23
KLMA_10046	FPR2	FK506-binding protein 2	330.38	401.60	337.42	491.18	625.12	618.01	0.70
KLMA_10047	EMC1	DUF1620 super family	994.67	1126.48	1039.06	896.57	1262.87	1093.41	0.04
KLMA_10048	MGR1	mitochondrial inner membrane i-AAA protease complex subunit MGR1	633.72	639.87	582.26	723.31	599.00	552.32	0.01
KLMA_10049	PDI1	protein disulfide-isomerase	4197.35	4532.77	4306.08	3339.00	3328.63	3533.48	-0.35
KLMA_10050	GRH1	GRASP65 homolog protein 1	658.41	708.11	733.31	257.36	340.36	322.40	-1.19
KLMA_10051	GLK1	glucokinase-1	1113.41	1010.14	940.39	887.32	428.82	571.34	-0.70
KLMA_10052	GID7	glucose-induced degradation protein 7	639.60	788.65	669.97	624.91	659.66	722.60	-0.06
KLMA_10053	ATG22	autophagy-related protein 22	1105.18	929.60	918.47	1240.56	1215.69	1129.71	0.28
KLMA_10054	SRO9	RNA-binding protein SRO9	807.73	934.07	768.64	284.28	425.45	450.33	-1.11
KLMA_10055		ribonuclease H-like domain	409.15	467.60	473.85	267.46	262.01	329.32	-0.65
KLMA_10056		dnaQ_like_exo super family	320.97	369.15	292.35	301.94	185.34	154.72	-0.61
KLMA_10057		hypothetical protein	2951.08	2798.86	2897.92	1444.10	2322.71	2026.04	-0.58
KLMA_10058	GRX2	glutaredoxin-1	1307.41	845.70	1207.16	2329.73	1926.75	1539.41	0.79
KLMA_10059	LSB5	LAS seventeen-binding protein 5	559.65	595.12	477.51	1252.34	1087.64	1198.86	1.12
KLMA_10060	MXR2	selR super family	72.90	98.44	62.12	244.75	213.15	205.72	1.50
KLMA_10061		DUF3128 super family	2.35	0.00	0.00	1.68	0.00	1.73	0.54
KLMA_10062	STE50	protein STE50	411.50	439.63	326.46	444.08	379.96	401.92	0.06
KLMA_10063	RRP7	ribosomal RNA-processing protein 7	820.66	712.58	761.33	493.70	693.36	621.47	-0.34
KLMA_10064	ACN9	acetate non-utilizing protein 9	320.97	328.88	299.66	229.61	192.93	135.70	-0.77
KLMA_10065	HIS4	histidine biosynthesis trifunctional protein	7253.07	4350.43	6954.28	2286.84	2690.03	2795.32	-1.26

Locus_tag	UniProt_gene	Product	Unique exon reads						log2 Fold Change
			KmWT.1	KmWT.2	KmWT.3	Kmmig1.1	Kmmig1.2	Kmmig1.3	
KLMA_10066	BIK1	CAP_GLY super family	343.31	236.04	327.68	407.91	373.22	411.43	0.40
KLMA_10067	PRY2	SCP super family	2686.54	2355.88	2569.03	1486.99	2094.40	1656.97	-0.54
KLMA_10068		SH3 super family	311.57	363.56	393.45	196.81	160.91	149.53	-1.07
KLMA_10069	HBN1	putative nitroreductase HBN1	197.52	237.15	194.90	263.25	284.76	282.64	0.40
KLMA_10070	HBN1	nitroreductase-like family 4	91.71	77.19	84.05	195.97	92.67	111.50	0.66
KLMA_10071	GNP1	high-affinity glutamine permease	3992.77	3094.19	4430.33	3586.27	2069.97	1404.57	-0.71
KLMA_10072	SMT3	ubiquitin-like protein SMT3	427.97	384.82	403.20	259.89	320.14	250.66	-0.55
KLMA_10073	GIN4	serine/threonine-protein kinase GIN4	1243.92	1383.77	1108.49	772.93	871.96	987.09	-0.51
KLMA_10074		putative NADPH dehydrogenase C23G7.10c	459.71	264.00	437.31	449.13	290.65	307.71	-0.15
KLMA_10075	GMC1	putative multicopper oxidase YDR506C	212.81	134.24	191.25	317.08	224.10	308.57	0.66
KLMA_10076		protein PSP1	1908.21	1577.30	1749.23	1185.89	1030.35	1108.10	-0.65
KLMA_10077	TFS1	carboxypeptidase Y inhibitor	496.16	741.67	468.98	956.28	824.79	847.93	0.62
KLMA_10078	LPP1	lipid phosphate phosphatase 1	186.94	148.78	207.08	68.97	65.71	101.99	-1.20
KLMA_10079	SAM2	S-adenosylmethionine synthetase 2	22156.60	12975.23	19526.56	3407.13	6371.66	6352.13	-1.76
KLMA_10080	VTA1	vacuolar protein sorting-associated protein VTA1	117.57	154.37	110.85	337.26	275.49	340.56	1.31
KLMA_10081	SWI6	regulatory protein SWI6	668.99	604.07	674.84	535.75	716.11	718.28	0.02
KLMA_10082	PLM2	protein PLM2	644.30	821.09	752.80	464.26	485.27	536.76	-0.58
KLMA_10083	RpL37a	ribosomal_L37e super family	3269.70	2610.93	3393.70	1047.96	1835.76	1472.86	-1.09
KLMA_10084	LCD1	DNA damage checkpoint protein LCD1	235.15	262.88	193.68	295.21	262.85	308.57	0.32
KLMA_10085	EMG1	hypothetical protein	821.83	719.29	835.63	493.70	550.98	481.44	-0.64
KLMA_10086	PRM1	plasma membrane fusion protein PRM1	7.05	22.37	9.75	93.36	37.07	48.40	2.18

Locus_tag	UniProt_gene	Product	Unique exon reads						log$_2$ Fold Change
			KmWT.1	KmWT.2	KmWT.3	Kmmig1.1	Kmmig1.2	Kmmig1.3	
KLMA_10088	SKG3	PH-like super family protein	998.19	981.06	1015.92	823.40	625.96	772.73	-0.43
KLMA_10089	MDL1	ATP-dependent permease MDL1	706.61	780.82	727.22	368.38	550.14	477.99	-0.67
KLMA_10090	MET2	homoserine O-acetyltransferase	2015.20	1299.87	1978.24	848.63	911.56	1064.02	-0.91
KLMA_10091	ATG26	sterol 3-beta-glucosyltransferase	406.80	745.02	461.67	2203.57	1609.97	1850.58	1.81
KLMA_10092	ISC1	inositol phosphosphingolipids phospholipase C	833.59	915.06	877.05	1079.92	895.55	1106.37	0.23
KLMA_10093	SPC25	probable kinetochore protein SPC25	114.05	119.70	104.76	132.89	107.84	104.59	0.03
KLMA_10094	PER1	protein PER1	1877.64	1396.08	1663.96	1127.02	1320.16	1163.42	-0.45
KLMA_10095	BOR1	boron transporter 1	1380.31	1041.46	1260.76	1641.75	1483.60	1469.40	0.32
KLMA_10096		uncharacterized protein YCR043C	143.44	155.49	159.57	153.07	167.65	235.10	0.28
KLMA_10097	GOR1	putative 2-hydroxyacid dehydrogenase YNL274C	793.62	948.62	679.71	952.08	988.23	877.32	0.22
KLMA_10098	TAF2	transcription initiation factor TFIID subunit 2	634.89	757.33	646.83	470.15	531.60	525.53	-0.42
KLMA_10099	SLP1	sad1_UNC super family	511.44	351.26	437.31	324.65	411.13	351.79	-0.26
KLMA_10100	CCN1	G1/S-specific cyclin CLN1	2069.28	1513.53	1968.49	695.56	663.87	703.58	-1.43
KLMA_10101	TOF1	topoisomerase 1-associated factor 1	805.37	698.04	723.57	623.22	518.97	614.56	-0.34
KLMA_10102	AVT2	vacuolar amino acid transporter 2	244.55	172.27	220.48	179.99	246.00	300.80	0.19
KLMA_10103	SEC2	rab guanine nucleotide exchange factor SEC2	537.31	531.36	482.38	408.75	401.02	404.52	-0.35
KLMA_10104	BNI1	FH2 super family	978.21	996.72	975.72	820.87	700.94	712.23	-0.40
KLMA_10106	CAN1	arginine permease	10360.52	5809.15	10468.58	2946.23	2642.01	2021.72	-1.81
KLMA_10107	LYP1	lysine-specific permease	9650.38	7117.97	9824.19	3037.90	3362.33	2910.28	-1.51
KLMA_10108	PIK1	phosphatidylinositol 4-kinase PIK1	1239.22	1548.21	1356.99	751.91	791.93	802.12	-0.82
KLMA_10109	TAM41	mitochondrial import protein MMP37	717.19	790.89	738.18	1190.10	1048.04	1004.38	0.53

Locus_tag	UniProt_gene	Product	Unique exon reads						log2 Fold Change
			KmWT.1	KmWT.2	KmWT.3	Kmmig1.1	Kmmig1.2	Kmmig1.3	
KLMA_10110	IST1	vacuolar protein sorting-associated protein IST1	324.50	315.46	343.51	1104.31	815.52	864.35	1.50
KLMA_10111	TFC4	transcription factor tau 131 kDa subunit	550.24	718.17	680.93	889.84	784.35	689.75	0.28
KLMA_10112	UFD1	ubiquitin fusion degradation protein 1	370.35	415.02	349.60	513.05	374.06	377.72	0.16
KLMA_10113		uncharacterized membrane protein YGL140C	2722.99	2258.56	2724.95	1182.53	924.20	1040.68	-1.29
KLMA_10114	PDR17	phosphatidylinositol transfer protein PDR17	384.46	342.31	388.58	671.16	572.88	512.56	0.66
KLMA_10115	CWC23	pre-mRNA-splicing factor CWC23	103.46	119.70	115.72	257.36	246.00	188.43	1.03
KLMA_10116	YIF1	protein transport protein YIF1	924.12	671.19	948.92	583.69	761.60	777.92	-0.26
KLMA_10117	POL2	DNA polymerase epsilon catalytic subunit A	1069.91	1105.23	1020.79	836.85	617.54	703.58	-0.57
KLMA_10118	ORC5	origin recognition complex subunit 5	631.37	534.71	684.59	697.24	556.88	635.30	0.03
KLMA_10119		GYF super family	217.51	176.75	210.74	378.48	280.54	267.09	0.62
KLMA_10120		uncharacterized ORAOV1 family protein YNL260C	69.37	61.53	77.96	264.09	138.17	131.38	1.35
KLMA_10121	ATX1	metal homeostasis factor ATX1	74.07	57.05	64.56	100.09	86.78	60.50	0.34
KLMA_10122	DSL1	protein transport protein DSL1	557.30	559.33	602.97	470.99	536.66	592.08	-0.10
KLMA_10123	SIP3	protein SIP3	805.37	944.14	935.52	1160.66	983.17	977.58	0.22
KLMA_10124	TGL2	lipase 2	343.31	373.63	342.29	602.20	438.09	463.29	0.51
KLMA_10125	COQ6	ubiquinone biosynthesis monooxygenase COQ6	1066.39	948.62	1045.15	852.83	848.37	1027.72	-0.17
KLMA_10126	FOL1	folic acid synthesis protein FOL1	3296.74	1726.08	2992.94	1072.35	1629.35	1673.39	-0.87
KLMA_10127	GIS2	zinc finger protein GIS2	6204.32	4989.18	5674.03	2576.16	3988.29	3246.51	-0.78
KLMA_10128	RTC4	hypothetical protein	158.72	212.54	187.59	243.07	240.95	178.92	0.25

Locus_tag	UniProt_gene	Product	Unique exon reads						log2 Fold Change
			KmWT.1	KmWT.2	KmWT.3	Kmmig1.1	Kmmig1.2	Kmmig1.3	
KLMA_10129	TEX1	protein TEX1	337.43	342.31	369.09	490.34	417.03	391.55	0.31
KLMA_10130	MRPL17	54S ribosomal protein L17	645.47	608.55	711.39	344.83	613.32	384.64	-0.55
KLMA_10131	NRD1	protein NRD1	523.20	506.75	527.45	704.81	672.30	776.19	0.47
KLMA_10132	RAD50	DNA repair protein RAD50	312.74	409.43	386.15	732.56	480.21	437.36	0.57
KLMA_10133	MPA43	protein MPA43	509.09	476.55	561.56	315.40	365.64	349.20	-0.59
KLMA_10134	RPA49	DNA-directed RNA polymerase I subunit RPA49	2068.11	1843.54	2079.34	803.21	991.60	947.33	-1.13
KLMA_10135		diphthamide biosynthesis protein 4	111.69	90.61	121.81	173.26	149.12	89.03	0.35
KLMA_10136		uncharacterized protein YJR098C	322.15	391.53	337.42	432.30	247.69	284.37	-0.12
KLMA_10137		cysteinyl-tRNA synthetase	1576.65	1557.16	1627.42	831.81	1089.32	953.38	-0.73
KLMA_10138	VPS75	vacuolar protein sorting-associated protein 75	730.13	601.83	679.71	804.89	622.59	580.85	0.00
KLMA_10139	CWC25	pre-mRNA-splicing factor CWC25	135.21	174.51	168.10	292.69	148.28	198.80	0.42
KLMA_10140	SUI1	eukaryotic translation initiation factor eIF-1	746.59	672.31	699.20	1424.75	1121.34	1038.95	0.76
KLMA_10141	SLA2	ANTH_AP180_CALM	1092.25	1228.28	922.12	1607.26	1434.74	1453.84	0.47
KLMA_10144	LAA1	hypothetical protein	644.30	535.83	566.43	589.58	734.64	783.97	0.27
KLMA_10145		putative transcriptional regulatory protein YJL206C	1151.04	915.06	1015.92	1058.89	834.89	803.85	-0.19
KLMA_10146		Zn2/Cys6 DNA-binding domain	1176.90	1873.74	1230.31	645.93	588.89	624.93	-1.20
KLMA_10147		RNase_H2_suC super family	48.20	25.73	19.49	43.74	83.41	59.64	1.00
KLMA_10148		F-box domain	209.28	164.44	237.53	353.24	258.64	304.25	0.58
KLMA_10149	RCY1	recyclin-1	345.66	434.04	426.34	570.24	498.75	446.01	0.33
KLMA_10150		pre-mRNA-splicing factor sap114	197.52	175.63	188.81	335.58	276.33	251.53	0.62
KLMA_10151	ECM25	protein ECM25	273.94	293.09	210.74	595.47	502.12	526.39	1.06

Locus_tag	UniProt_gene	Product	Unique exon reads						log2 Fold Change
			KmWT.1	KmWT.2	KmWT.3	Kmmig1.1	Kmmig1.2	Kmmig1.3	
KLMA_10152	BUD5	rasGEF super family	431.49	492.21	423.91	635.84	531.60	636.16	0.42
KLMA_10153	ACO2	probable aconitate hydratase 2	2369.09	1449.77	2023.31	663.60	1044.67	1148.73	-1.03
KLMA_10155	PHO87	inorganic phosphate transporter PHO87	5119.12	4031.62	4860.32	3455.91	2519.01	2408.09	-0.74
KLMA_10156	UBP12	ubiquitin carboxyl-terminal hydrolase 12	775.98	1011.26	853.91	709.85	662.19	787.43	-0.29
KLMA_10157	RBK1	probable ribokinase	356.25	378.10	386.15	462.58	529.92	516.02	0.43
KLMA_10158	RRP43	exosome complex component RRP43	659.58	674.55	660.22	463.42	531.60	491.82	-0.42
KLMA_10159	FEN1	elongation of fatty acids protein 2	3040.43	2553.88	3074.55	1795.66	2058.17	1845.39	-0.60
KLMA_10160	SNT1	probable DNA-binding protein SNT1	1380.31	1512.42	1421.55	955.44	908.19	910.16	-0.64
KLMA_10161	CDC6	cell division control protein 6	202.23	205.83	204.65	110.18	183.66	196.21	-0.32
KLMA_10162	VPS60	vacuolar protein-sorting-associated protein 60	251.61	177.87	158.36	470.99	401.02	363.89	1.07
KLMA_10163	COQ3	hexaprenyldihydroxybenzoate methyltransferase	376.23	381.46	336.20	470.15	465.05	421.80	0.31
KLMA_10164	VPS72	vacuolar protein sorting-associated protein 72	371.53	365.80	341.08	693.87	587.21	562.69	0.77
KLMA_10165	HMI1	ATP-dependent DNA helicase HMI1	190.47	241.63	215.61	354.93	323.51	330.18	0.64
KLMA_10166	VPS52	vacuolar protein sorting-associated protein 52	297.46	335.60	274.08	561.83	550.98	507.38	0.84
KLMA_10167	RFC4	replication factor C subunit 4	261.01	295.32	308.19	275.03	323.51	280.05	0.02
KLMA_10168	TRM10	tRNA (guanine-N(1)-)-methyltransferase	285.70	223.73	238.75	249.79	258.64	272.27	0.06
KLMA_10169		uncharacterized membrane protein YOL092W	886.50	568.27	878.27	630.79	886.29	732.97	-0.05
KLMA_10170		SMC_prok_B	122.28	62.64	115.72	430.62	203.04	242.02	1.54
KLMA_10171		CRT10 super family[	305.69	349.02	294.79	572.76	412.81	482.31	0.63

Locus_tag	UniProt_gene	Product	Unique exon reads						log2 Fold Change
			KmWT.1	KmWT.2	KmWT.3	Kmmig1.1	Kmmig1.2	Kmmig1.3	
KLMA_10172	MPS1	serine/threonine-protein kinase MPS1	606.68	566.04	649.26	474.36	478.53	456.38	-0.37
KLMA_10173	ARP2	actin-related protein 2	2299.73	2317.84	2268.15	2719.14	2874.53	2982.88	0.32
KLMA_10174		hypothetical protein	183.41	308.75	197.34	1090.85	664.71	726.92	1.85
KLMA_10175	APM4	AP-2 complex subunit mu	618.43	581.70	549.37	657.71	616.69	618.88	0.11
KLMA_10176	PRS5	ribose-phosphate pyrophosphokinase 5	1915.26	1591.84	1802.83	715.74	759.07	741.62	-1.26
KLMA_10177	RTK1	probable serine/threonine-protein kinase YDL025C	775.98	1013.50	726.00	719.95	603.21	705.31	-0.31
KLMA_10178	MAM3	protein MAM3	1648.37	1333.43	1369.17	1835.19	1476.02	1774.52	0.23
KLMA_10179	GPD1	glycerol-3-phosphate dehydrogenase [NAD+] 1	23113.65	16530.30	19894.44	6058.15	5027.90	4333.00	-1.95
KLMA_10180	ARG1	argininosuccinate synthase	8762.70	6319.26	8923.99	3388.63	4585.60	4868.90	-0.90
KLMA_10181		probable dipeptidyl-peptidase 3	1918.79	2028.11	1806.48	922.64	961.27	1035.50	-0.98
KLMA_10182	GPM3	phosphoglycerate mutase 3	2580.72	1920.72	2408.24	386.89	378.27	343.15	-2.64
KLMA_10183	RPN4	zinc finger	1429.69	1922.96	1480.02	1408.77	1164.30	1235.16	-0.34
KLMA_10184	och1	initiation-specific alpha-1,6-mannosyltransferase	420.91	533.60	438.53	489.50	351.31	452.06	-0.11
KLMA_10185	OSH2	oxysterol-binding protein homolog 1	2028.13	2714.96	2180.45	2784.75	2613.36	2612.08	0.21
KLMA_10186	YAT1	putative mitochondrial carnitine O-acetyltransferase	134.03	135.36	112.07	874.70	780.98	917.08	2.75
KLMA_10187	CDC15	cell division control protein 15	229.27	304.27	238.75	427.26	315.09	360.44	0.51
KLMA_10188	KIN3	serine/threonine-protein kinase KIN3	121.10	156.61	124.25	597.99	534.97	455.51	1.98
KLMA_10189	ADE1	phosphoribosylaminoimidazole-succinocarboxamide synthase	2606.59	2311.13	2621.41	1037.03	1669.79	1353.58	-0.89
KLMA_10190	BUD14	bud site selection protein 14	159.90	186.81	168.10	191.76	195.45	223.87	0.25

Locus_tag	UniProt_gene	Product	Unique exon reads						log$_2$ Fold Change
			KmWT.1	KmWT.2	KmWT.3	Kmmig1.1	Kmmig1.2	Kmmig1.3	
KLMA_10191	KAP104	importin subunit beta-2	907.66	951.97	1051.24	572.76	709.37	702.72	-0.55
KLMA_10192	DAL1	allantoinase	130.51	128.64	131.56	289.32	210.62	161.63	0.76
KLMA_10194	ERP3	protein ERP3	68.19	123.05	60.91	134.57	130.58	114.09	0.59
KLMA_10195	CDC7	cell division control protein 7	122.28	158.85	132.78	322.97	235.05	301.66	1.05
KLMA_10196	TSC13	enoyl reductase TSC13	1642.49	1645.53	1544.58	2047.98	2063.23	2037.28	0.35
KLMA_10197	FLO5	flocculation protein FLO5	621.96	511.22	553.03	1116.93	732.96	1079.58	0.80
KLMA_10199	NOP1	rRNA 2'-O-methyltransferase fibrillarin	6478.26	4397.42	5309.81	1192.62	2572.93	2495.39	-1.37
KLMA_10201	SLX5	e3 ubiquitin-protein ligase complex SLX5-SLX8 subunit SLX5	465.59	606.31	460.45	332.22	325.20	338.83	-0.62
KLMA_10202		uncharacterized protein C12G12.12	753.64	917.29	719.91	1080.76	743.91	901.52	0.19
KLMA_10203		hypothetical protein	151.67	135.36	142.52	174.10	197.14	143.48	0.26
KLMA_10204		uncharacterized ABC transporter ATP-binding protein/permease YOL075C	570.23	535.83	659.01	646.77	581.31	669.87	0.11
KLMA_10206	PAC10	prefoldin subunit 3	232.79	164.44	226.57	216.99	300.76	285.24	0.37
KLMA_10207		uncharacterized membrane protein YOL073C	174.01	203.59	176.63	434.83	283.91	352.66	0.95
KLMA_10208	PEX8	peroxisomal biogenesis factor 8	170.48	177.87	177.85	328.01	296.55	369.94	0.92
KLMA_10209	DBP9	ATP-dependent RNA helicase DBP9	793.62	708.11	919.69	423.89	497.90	533.31	-0.73
KLMA_10210	YSH1	endoribonuclease YSH1	931.18	827.80	965.97	1018.52	974.75	990.55	0.13
KLMA_10211		GAL4	1148.69	1203.67	1164.53	830.12	577.10	624.93	-0.79
KLMA_10212		uncharacterized peptide chain release factor-like protein YLR281C	63.49	78.31	58.47	105.13	51.39	88.16	0.29
KLMA_10213		DUF1640	92.88	112.98	103.54	200.17	198.82	197.94	0.95

Locus_tag	UniProt_gene	Product	Unique exon reads						log2 Fold Change
			KmWT.1	KmWT.2	KmWT.3	Kmmig1.1	Kmmig1.2	Kmmig1.3	
KLMA_10214	ECI1	3,2-trans-enoyl-CoA isomerase	38.80	68.24	48.73	173.26	175.24	151.26	1.68
KLMA_10215	NNT1	putative nicotinamide N-methyltransferase	545.54	429.56	477.51	518.93	592.26	576.52	0.22
KLMA_10216	CTS1	endochitinase	2222.13	2275.34	2067.16	2013.49	1900.63	1874.78	-0.18
KLMA_10217	LAS17	proline-rich protein LAS17	277.47	357.97	327.68	267.46	302.45	375.13	-0.03
KLMA_10218		uncharacterized protein YLR287C	306.87	362.44	297.22	277.55	295.71	283.51	-0.17
KLMA_10220	SER1	phosphoserine aminotransferase	2108.08	1637.70	2187.75	1359.99	1742.24	1706.23	-0.30
KLMA_10221	MEC3	DNA damage checkpoint control protein MEC3	122.28	195.76	138.87	246.43	207.25	267.09	0.66
KLMA_10222	GUF1	GTP-binding protein GUF1	211.63	325.53	244.84	179.99	200.51	159.04	-0.54
KLMA_10223	PET18	protein PET18	29.39	43.63	41.42	71.49	74.14	47.54	0.76
KLMA_10224	GCD7	translation initiation factor eIF-2B subunit beta	326.85	315.46	350.82	488.66	413.66	335.37	0.32
KLMA_10225	GSP1	GTP-binding nuclear protein GSP1/Ran	3918.70	3150.12	3538.66	1249.81	1834.07	1647.46	-1.16
KLMA_10226		hypothetical protein	128.15	144.31	86.49	168.21	132.27	168.55	0.38
KLMA_10227		ATP synthase subunit H	850.05	955.33	867.31	730.04	862.70	729.51	-0.20
KLMA_10228	YHC1	U1 small nuclear ribonucleoprotein C homolog	105.82	111.87	108.41	320.44	224.10	244.61	1.27
KLMA_10229	TUF1	elongation factor Tu	2995.76	2741.81	2600.70	1880.61	2834.09	2434.02	-0.22
KLMA_10231	ECM38	gamma-glutamyltransferase	135.21	203.59	116.94	474.36	431.35	461.56	1.58
KLMA_10232	MSB1	rhoGAP_fMSB1	653.71	831.16	643.17	761.16	556.88	693.21	-0.08
KLMA_10233		hypothetical protein	221.04	209.19	172.97	257.36	213.15	254.12	0.26
KLMA_10234		glucan 1,3-beta-glucosidase	1621.33	1732.79	1197.42	2021.06	1029.51	1379.51	-0.04
KLMA_10235		WW domain-containing protein YFL010C	398.57	355.73	350.82	1093.38	791.09	893.74	1.33
KLMA_10236	ULS1	SNF2_N	509.09	601.83	553.03	636.68	320.98	375.99	-0.32

Locus_tag	UniProt_gene	Product	Unique exon reads						log2 Fold Change
			KmWT.1	KmWT.2	KmWT.3	Kmmig1.1	Kmmig1.2	Kmmig1.3	
KLMA_10237	HRI1	uncharacterized protein YLR301W	3704.72	3426.43	3717.72	2790.63	2487.84	2179.90	-0.54
KLMA_10238		hypothetical protein	3107.45	2086.28	2983.19	527.34	770.87	656.04	-2.06
KLMA_10239		hypothetical protein	0.00	2.24	2.44	188.40	53.08	51.86	5.97
KLMA_10240	ERV14	ER-derived vesicles protein ERV14	613.73	515.70	563.99	595.47	742.22	618.01	0.21
KLMA_10241		hypothetical protein	62.31	112.98	97.45	127.84	75.82	95.08	0.13
KLMA_10242	NGR1	RRM	143.44	157.73	146.18	359.97	181.13	260.17	0.84
KLMA_10243	MET8	siroheme biosynthesis protein MET8	623.14	324.41	565.21	412.96	654.60	661.23	0.19
KLMA_10244	OLE1	acyl-CoA desaturase 1	46438.93	51536.23	52301.47	33168.83	16294.35	15170.27	-1.22
KLMA_10245	SDS23	protein SDS23	1421.46	1373.70	1203.51	576.97	567.83	658.64	-1.15
KLMA_10246		hypothetical protein	79.95	45.86	47.51	199.33	106.99	125.33	1.32
KLMA_10247	UBC2	ubiquitin-conjugating enzyme E2 2	302.16	285.26	253.37	494.54	454.94	387.23	0.67
KLMA_10248	PKP2	probable protein kinase YGL059W	517.32	503.39	468.98	690.51	599.84	659.50	0.39
KLMA_10249	HPC2	cylicin-2	311.57	289.73	244.84	418.85	373.22	448.60	0.55
KLMA_10250	YBP1	YAP1-binding protein 1	763.05	865.84	728.44	529.87	606.58	511.70	-0.52
KLMA_10251	ATG12	autophagy-related protein 12	19.99	32.44	9.75	50.46	64.03	56.18	1.45
KLMA_10252	DUO1	DASH complex subunit DUO1	136.38	149.90	126.69	97.56	169.34	146.94	0.00
KLMA_10253	PYC2	pyruvate carboxylase 2	6716.94	3699.38	5945.67	5773.03	6690.11	6843.09	0.24
KLMA_10255	SOH1	mediator of RNA polymerase II transcription subunit 31	77.60	72.71	46.29	36.17	28.64	25.93	-1.12
KLMA_10256	RRN11	RNA polymerase I-specific transcription initiation factor RRN11	319.80	298.68	348.38	200.17	232.52	220.41	-0.57
KLMA_10257	HRD1	ERAD-associated E3 ubiquitin-protein ligase HRD1	474.99	550.38	493.34	1571.10	1043.83	1101.19	1.29

Locus_tag	UniProt_gene	Product	Unique exon reads						log2 Fold Change
			KmWT.1	KmWT.2	KmWT.3	Kmmig1.1	Kmmig1.2	Kmmig1.3	
KLMA_10258		OPA3-like protein	54.08	67.12	59.69	227.09	182.82	178.92	1.70
KLMA_10259	HTZ1	histone H2A.Z	707.79	627.56	744.28	682.10	669.77	618.88	-0.08
KLMA_10260	PLB	lysophospholipase	3830.52	3155.71	3002.68	10396.33	7514.05	8092.08	1.38
KLMA_10261	TAF4	transcription initiation factor TFIID subunit 4	629.01	623.09	610.28	372.59	456.62	417.48	-0.58
KLMA_10262	RCL1	RNA 3'-terminal phosphate cyclase-like protein	720.72	695.80	920.90	338.95	434.72	420.94	-0.97
KLMA_10263	MVP1	sorting nexin MVP1	257.48	344.54	291.13	433.15	422.92	508.24	0.61
KLMA_10265	MDM12	mitochondrial distribution and morphology protein 12	181.06	163.32	177.85	212.79	225.78	235.97	0.37
KLMA_10266	COQ10	coenzyme Q-binding protein COQ10	75.25	71.59	102.32	95.04	128.90	100.27	0.38
KLMA_10267	AIM34	SAP super family	1107.54	1094.04	1073.17	721.63	767.50	811.63	-0.51
KLMA_10268	MIC17	mitochondrial intermembrane space cysteine motif-containing protein MIC17	246.90	231.56	243.63	705.65	551.82	582.57	1.35
KLMA_10269	TOP1	DNA topoisomerase 1	427.97	462.00	518.92	746.02	522.34	501.33	0.33
KLMA_10270	CDC5	cell cycle serine/threonine-protein kinase CDC5/MSD2	564.35	493.32	565.21	717.42	903.14	886.83	0.63
KLMA_10271	RPB11	DNA-directed RNA polymerase II subunit RPB11	226.92	143.19	191.25	406.23	330.25	235.97	0.79
KLMA_10272	SIN3	transcriptional regulatory protein SIN3	2303.25	2624.35	2381.44	2254.04	1815.54	1893.80	-0.29
KLMA_10273	PFA4	palmitoyltransferase PFA4	222.21	215.90	283.82	181.67	213.99	190.16	-0.30
KLMA_10274	KU70	ATP-dependent DNA helicase II subunit 1	532.61	463.12	533.54	344.83	399.33	336.23	-0.50
KLMA_10275	NGL2	RNA exonuclease NGL2	233.97	217.02	265.55	296.89	227.47	238.56	0.09

Locus_tag	UniProt_gene	Product	Unique exon reads						log2 Fold Change
			KmWT.1	KmWT.2	KmWT.3	Kmmig1.1	Kmmig1.2	Kmmig1.3	
KLMA_10276	MRPL33	54S ribosomal protein L33	92.88	66.00	97.45	55.51	106.99	70.88	-0.13
KLMA_10277		hypothetical protein	192.82	151.02	151.05	422.21	425.45	344.88	1.27
KLMA_10278	DSS1	exoribonuclease II	824.19	846.82	956.23	428.10	372.37	365.62	-1.17
KLMA_10279	NDI1	rotenone-insensitive NADH-ubiquinone oxidoreductase	3904.59	3415.24	3767.66	3741.87	5328.67	4543.91	0.30
KLMA_10280	GTR1	GTP-binding protein GTR1	243.38	293.09	247.28	245.59	275.49	248.07	-0.03
KLMA_10281	PTH1	peptidyl-tRNA hydrolase	130.51	107.39	143.74	132.89	133.95	129.65	0.06
KLMA_10282	PNO1	pre-rRNA-processing protein PNO1	968.80	776.34	853.91	430.62	433.03	426.99	-1.01
KLMA_10283	MDM32	mitochondrial distribution and morphology protein 32	500.86	627.56	605.41	520.62	404.39	400.20	-0.39
KLMA_10284	SPP2	hypothetical protein	47.03	62.64	82.83	144.66	108.68	114.09	0.93
KLMA_10285	SHE9	she9_MDM33 super family	122.28	143.19	113.29	206.06	203.04	187.56	0.65
KLMA_10286	RPT3	26S protease regulatory subunit 6B homolog	1576.65	1686.92	1595.75	1387.75	1629.35	1547.19	-0.09
KLMA_10289	SMP3	GPI mannosyltransferase 4	203.40	186.81	196.12	252.32	326.04	318.95	0.61
KLMA_10290	SXM1	IBN_N super family	1690.70	1458.72	1617.67	952.08	1202.21	1243.80	-0.49
KLMA_10292	NCB2	negative cofactor 2 complex subunit beta	331.56	342.31	405.64	552.58	366.48	423.53	0.32
KLMA_10293	UTP5	U3 small nucleolar RNA-associated protein 5	1046.40	1078.38	1191.33	405.39	554.35	554.05	-1.13
KLMA_10294	HPT1	hypoxanthine-guanine phosphoribosyltransferase	2259.75	2056.08	2415.54	968.06	939.36	741.62	-1.35
KLMA_10295	MCH2	probable transporter MCH2	182.24	227.09	121.81	1857.06	1248.55	1084.76	2.98
KLMA_10296		UPF0676 protein C1494.01	1554.31	1242.82	1202.29	1489.51	1537.52	1250.72	0.10

Locus_tag	UniProt_gene	Product	Unique exon reads						log2 Fold Change
			KmWT.1	KmWT.2	KmWT.3	Kmmig1.1	Kmmig1.2	Kmmig1.3	
KLMA_10297	URH1	uridine nucleosidase	390.34	338.95	310.62	1333.08	710.21	858.30	1.48
KLMA_10298	DIT2	cytochrome P450-DIT2	262.19	191.29	174.19	653.50	421.24	459.84	1.29
KLMA_10299	DIT1	spore wall maturation protein DIT1	222.21	185.70	181.50	308.67	233.37	272.27	0.47
KLMA_10300	RPB7	DNA-directed RNA polymerase II subunit RPB7	507.91	513.46	554.25	382.68	563.62	422.67	-0.20
KLMA_10301	MRPL44	hypothetical protein	84.65	76.07	112.07	75.70	128.06	88.16	0.10
KLMA_10302	RRP36	UPF0399 protein YOR287C	282.17	192.41	216.83	180.83	200.51	199.67	-0.25
KLMA_10303	RDL2	putative thiosulfate sulfurtransferase FMP31	263.36	337.83	321.59	550.05	481.90	523.80	0.75
KLMA_10304	RDL1	putative thiosulfate sulfurtransferase YOR285W	163.43	285.26	192.46	487.81	381.64	383.77	0.97
KLMA_10305		probable phosphoglycerate mutase YOR283W	864.16	757.33	975.72	316.24	387.54	372.54	-1.27
KLMA_10306	MRE11	double-strand break repair protein MRE11	435.02	410.54	479.94	702.28	481.05	515.15	0.36
KLMA_10307	PLP2	phosducin-like protein 2	171.66	137.59	151.05	236.34	267.91	280.91	0.77
KLMA_10308	UBP8	ubiquitin carboxyl-terminal hydrolase 8	174.01	185.70	200.99	248.11	264.54	274.00	0.49
KLMA_10309	FSH2	family of serine hydrolases 2	138.74	158.85	144.96	217.83	165.13	189.29	0.37
KLMA_10310	FSH3	family of serine hydrolases 3	189.29	161.09	209.52	204.38	169.34	228.19	0.11
KLMA_10311	FMP42	protein FMP42	959.39	869.19	962.32	1455.87	1181.15	1199.72	0.46
KLMA_10312		transcriptional repressor Rfm1	177.54	117.46	186.37	255.68	191.24	230.78	0.50
KLMA_10313	ERG8	phosphomevalonate kinase	480.87	417.26	458.02	506.32	554.35	629.25	0.32
KLMA_10314		silent chromatin protein ESC1	591.39	666.72	612.72	713.22	599.84	559.24	0.00
KLMA_10315	TRS130	transport protein particle 130 kDa subunit	387.99	295.32	381.27	333.06	326.04	362.16	-0.06
KLMA_10316	HEM4	uroporphyrinogen-	39.97	51.46	42.63	111.02	140.69	165.96	1.64

Locus_tag	UniProt_gene	Product	Unique exon reads						log2 Fold Change
			KmWT.1	KmWT.2	KmWT.3	Kmmig1.1	Kmmig1.2	Kmmig1.3	
		III synthase							
KLMA_10317	GUA1	GMP synthase	12336.92	8757.91	11549.06	2758.67	4921.75	4442.78	-1.43
KLMA_10318		RRM	266.89	256.17	266.77	200.17	222.41	312.90	-0.10
KLMA_10319	SKY1	serine/threonine-protein kinase SKY1	1000.54	1080.62	900.20	1217.85	1057.31	1119.34	0.19
KLMA_10320	GAS3	probable 1,3-beta-glucanosyltransferas e GAS3	6365.39	4684.91	5989.53	2344.03	2934.35	2766.80	-1.08
KLMA_10321	DNAJA1	dnaJ-related protein SCJ1	116.40	107.39	148.61	175.78	212.30	196.21	0.65
KLMA_10322	CAF20	hypothetical protein	355.07	276.31	366.66	301.10	429.66	420.94	0.21
KLMA_10323	MTG2	GTPase MTG2	99.94	142.07	141.30	326.33	192.08	238.56	0.98
KLMA_10324		THO complex subunit THP2	92.88	121.93	138.87	139.62	143.22	172.01	0.36
KLMA_10325	CDC23	anaphase-promoting complex subunit CDC23	125.80	133.12	131.56	152.23	176.92	191.02	0.41
KLMA_10326	PRP8	pre-mRNA-splicing factor 8	887.68	892.68	868.52	693.03	513.07	530.71	-0.61
KLMA_10327		hypothetical protein	59.96	52.58	65.78	116.07	107.84	103.72	0.88
KLMA_10328	DNA2	DNA replication ATP-dependent helicase DNA2	235.15	307.63	305.75	428.94	354.68	388.09	0.47
KLMA_10329		RRM	1088.72	1307.70	1025.66	2444.12	1635.25	1701.05	0.76
KLMA_10330	MGA1	HSF_DNA-bind super family	1397.94	1231.63	1247.36	647.62	679.04	665.55	-0.96
KLMA_10331	SOL3	6-phosphogluconolact onase 3	362.12	330.00	401.98	426.42	485.27	659.50	0.52
KLMA_10332	CPD1	2',3'-cyclic-nucleotide 3'-phosphodiesterase	199.87	291.97	333.77	449.13	440.62	458.11	0.71
KLMA_10333	TDS4	transcription factor IIIB 70 kDa subunit	457.36	562.68	570.08	606.40	467.57	525.53	0.01
KLMA_10334	SDA1	protein SDA1	1375.60	1264.08	1361.87	539.96	513.91	629.25	-1.25
KLMA_10336	LSC2	succinyl-CoA ligase [ADP-forming] subunit beta	5716.39	6657.09	5325.65	2838.57	5216.62	4815.31	-0.46
KLMA_10338	FMP43	UPF0041 protein FMP43	87.00	139.83	118.16	222.04	255.27	219.55	1.01

Locus_tag	UniProt_gene	Product	Unique exon reads						log$_2$ Fold Change
			KmWT.1	KmWT.2	KmWT.3	Kmmig1.1	Kmmig1.2	Kmmig1.3	
KLMA_10339	YAP1801	clathrin coat assembly protein AP180A	440.90	628.68	376.40	479.40	444.83	493.55	-0.03
KLMA_10341	PFK1	6-phosphofructokinase subunit alpha	27940.01	24876.55	26111.76	3655.24	4612.56	4387.46	-2.64
KLMA_10342	LCB3	dihydrosphingosine 1-phosphate phosphatase LCB3	462.06	351.26	510.39	245.59	399.33	395.87	-0.35
KLMA_10343	DYN1	dynein heavy chain	446.78	533.60	437.31	625.75	479.37	538.49	0.21
KLMA_10344	RHO4	GTP-binding protein RHO4	163.43	121.93	155.92	161.48	236.74	195.34	0.43
KLMA_10345	TRM2	tRNA (uracil-5-)-methyltransferase	684.27	614.14	665.10	418.85	576.25	570.47	-0.33
KLMA_10346		hypothetical protein	2.35	0.00	2.44	9.25	9.27	8.64	2.53
KLMA_10347	GLG1	GT8_Glycogenin	1375.60	993.36	1307.05	704.81	769.18	893.74	-0.63
KLMA_10348	TIF1	ATP-dependent RNA helicase eIF4A	19243.15	17092.98	18215.86	6461.85	10349.83	9296.99	-1.06
KLMA_10349	UTP30	ribosome biogenesis protein UTP30	198.70	232.68	193.68	123.64	177.76	165.96	-0.42
KLMA_10350	YUR1	probable mannosyltransferase YUR1	417.38	453.05	372.75	536.60	495.38	554.91	0.35
KLMA_10351	RPB4	DNA-directed RNA polymerase II subunit RPB4	554.94	441.87	499.43	607.24	684.93	615.42	0.35
KLMA_10352	YAK1	dual specificity protein kinase YAK1	704.26	656.65	659.01	782.18	698.41	738.16	0.14
KLMA_10353	TIM17	mitochondrial import inner membrane translocase subunit TIM17	1214.53	997.84	1068.30	895.73	1020.24	852.25	-0.24
KLMA_10355		hypothetical protein	32.92	69.36	36.54	100.09	73.30	57.05	0.73
KLMA_10356	BIO2	biotin synthase	2012.85	1210.38	1804.05	455.85	353.00	475.39	-1.97
KLMA_10357	SFH5	phosphatidylinositol transfer protein SFH5	149.32	194.65	137.65	236.34	260.33	285.24	0.70
KLMA_10358		hypothetical protein	332.73	378.10	303.31	300.26	363.95	414.03	0.09
KLMA_10359		uncharacterized protein YJL147C	644.30	747.26	640.73	825.92	602.37	675.06	0.05

Locus_tag	UniProt_gene	Product	Unique exon reads						log2 Fold Change
			KmWT.1	KmWT.2	KmWT.3	Kmmig1.1	Kmmig1.2	Kmmig1.3	
KLMA_10360	TFA2	transcription initiation factor IIE subunit beta	564.35	479.90	546.94	591.26	557.72	587.76	0.13
KLMA_10361	RPA34	DNA-directed RNA polymerase I subunit RPA34	511.44	384.82	445.83	391.93	362.27	354.38	-0.28
KLMA_10362	LAS1	protein LAS1	109.34	91.73	109.63	192.60	178.61	206.58	0.90
KLMA_10363	OAF3	uncharacterized transcriptional regulatory protein YKR064W	961.75	775.22	1006.17	894.05	642.81	751.12	-0.26
KLMA_10364	DAS1	F-box protein YJL149W	614.91	709.22	585.92	762.84	652.08	714.82	0.16
KLMA_10365	PAM17	presequence translocated-associated motor subunit PAM17	915.89	995.60	861.22	525.66	732.11	657.77	-0.53
KLMA_10366		hypothetical protein	168.13	147.66	196.12	188.40	127.21	143.48	-0.16
KLMA_10367		hypothetical protein	196.35	145.42	181.50	325.49	195.45	299.07	0.65
KLMA_10368	RIM1	single-stranded DNA-binding protein RIM1	1797.69	1299.87	1603.05	1350.74	1613.34	1486.69	-0.08
KLMA_10369	GON7	protein GON7	54.08	66.00	46.29	65.60	79.19	65.69	0.34
KLMA_10370	FEN2	pantothenate transporter FEN2	1028.76	600.72	900.20	576.97	980.64	1011.29	0.02
KLMA_10371	MNN11	probable alpha-1,6-mannosyltransferase MNN11	1025.24	896.04	1096.31	878.91	960.42	1043.27	-0.07
KLMA_10372		UPF0508 protein KLLA0A06237g	1179.26	1193.60	1132.86	603.88	578.78	669.01	-0.92
KLMA_10373	ATP12	protein ATP12	235.15	309.87	248.50	211.11	259.48	230.78	-0.18
KLMA_10374	PFD1	prefoldin subunit 1	54.08	55.93	59.69	48.78	66.56	76.06	0.17
KLMA_10375	ATG27	autophagy-related protein 27	545.54	711.46	533.54	1597.17	1073.32	1112.42	1.08
KLMA_10376	RPL17B	ribosomal_L22	12572.07	11452.74	12747.69	4045.49	7085.23	6127.40	-1.09
KLMA_10378	COY1	protein CASP	500.86	684.61	572.52	397.82	531.60	539.36	-0.26
KLMA_10379	SWI3	RSC chromatin remodeling complex subunit RSC8	1007.60	994.48	884.36	687.99	867.75	823.73	-0.28

Locus_tag	UniProt_gene	Product	Unique exon reads						log2 Fold Change
			KmWT.1	KmWT.2	KmWT.3	Kmmig1.1	Kmmig1.2	Kmmig1.3	
KLMA_10380	KRE9	cell wall synthesis protein KRE9	222.21	378.10	225.35	394.46	256.96	397.60	0.34
KLMA_10381	RFA3	replication factor A protein 3	89.36	126.41	75.52	89.99	103.62	90.76	-0.04
KLMA_10382	STE3	pheromone a factor receptor	146.97	82.78	120.59	624.07	374.90	433.91	2.03
KLMA_10383	SET2	histone-lysine N-methyltransferase	480.87	474.31	516.49	574.44	530.76	545.41	0.17
KLMA_10384	LST4	protein LST4	934.70	1076.14	969.63	1131.22	759.07	834.97	-0.13
KLMA_10385	ZRT3	zinc-regulated transporter 3	701.91	682.38	611.50	1353.26	914.09	938.69	0.68
KLMA_10386	TPO5	polyamine transporter TPO5	480.87	466.48	572.52	658.55	629.33	661.23	0.36
KLMA_10387	SNU114	U5 small nuclear ribonucleoprotein component	238.67	327.76	242.41	333.90	395.96	424.40	0.51
KLMA_10388	EBP2	rRNA-processing protein EBP2	1011.13	909.46	911.16	425.58	470.94	517.75	-1.00
KLMA_10389	FPS1	farnesyl pyrophosphate synthetase	2949.90	2181.37	2659.17	1622.40	2824.83	2554.16	-0.15
KLMA_10390	QCR8	cytochrome b-c1 complex subunit 8	1365.02	1434.11	1393.54	1155.61	1740.56	1280.11	-0.01
KLMA_10391	NNK1	protein Kinases	770.10	858.00	730.88	587.90	482.74	506.51	-0.58
KLMA_10392	rplN	54S ribosomal protein L38	235.15	251.70	253.37	201.01	333.62	240.29	0.07
KLMA_10393		lrgB super family	340.96	350.14	366.66	1952.10	853.43	1140.95	1.90
KLMA_10394	GAP1	general amino-acid permease GAP1	699.56	1011.26	755.24	911.71	1222.43	1118.47	0.40
KLMA_10395		matE super family	351.54	481.02	332.55	858.72	545.08	661.23	0.83
KLMA_10396	YNG2	chromatin modification-related protein YNG2	192.82	138.71	241.19	455.01	278.86	319.81	0.88
KLMA_10397		uncharacterized protein YNL295W	182.24	213.66	192.46	337.26	250.22	269.68	0.54
KLMA_10398	RIM21	pH-response regulator protein palH/RIM21	257.48	268.48	267.99	269.14	275.49	299.07	0.09
KLMA_10399	MSB3	GTPase-activating protein GYP3	536.13	566.04	487.25	280.07	311.72	365.62	-0.73
KLMA_10400	MDY2	ubiquitin-like protein MDY2	523.20	475.43	539.63	754.43	534.13	546.27	0.25

Locus_tag	UniProt_gene	Product	Unique exon reads						log2 Fold Change
			KmWT.1	KmWT.2	KmWT.3	Kmmig1.1	Kmmig1.2	Kmmig1.3	
KLMA_10401	ERF4	ras modification protein ERF4	24.69	57.05	41.42	69.81	51.39	65.69	0.60
KLMA_10402	TGL4	lipase 5	727.78	912.82	736.97	772.93	600.69	642.21	-0.24
KLMA_10403	DIA2	protein DIA2	320.97	277.43	297.22	186.72	167.65	185.84	-0.73
KLMA_10404	ATX2	zinc/iron permease	723.07	533.60	710.17	210.26	267.07	261.90	-1.41
KLMA_10405		bud site selection protein 21	391.52	325.53	354.47	163.17	201.35	218.68	-0.88
KLMA_10406	TVP38	Golgi apparatus membrane protein TVP38	391.52	415.02	399.55	640.05	577.94	514.29	0.52
KLMA_10407	RTS2	zinc finger protein RTS2	47.03	60.41	71.87	121.11	100.25	130.52	0.97
KLMA_10408	OMA1	mitochondrial metalloendopeptidase OMA1	231.62	290.85	271.64	448.28	366.48	363.03	0.57
KLMA_10409	PRP16	pre-mRNA-splicing factor ATP-dependent RNA helicase PRP16	252.78	338.95	266.77	407.07	304.98	308.57	0.25
KLMA_10410	MRPL20	54S ribosomal protein L20	433.84	457.53	348.38	313.71	404.39	395.01	-0.16
KLMA_10411		hypothetical protein	14.11	23.49	12.18	62.24	17.69	39.76	1.26
KLMA_10412	HBS1	eukaryotic peptide chain release factor GTP-binding subunit	927.65	1058.24	946.48	667.80	727.90	743.34	-0.46
KLMA_10413	UFE1	syntaxin UFE1	163.43	159.97	87.71	125.32	124.69	131.38	-0.11
KLMA_10414	CDC21	thymidylate synthase	390.34	393.76	342.29	248.95	308.35	281.78	-0.43
KLMA_10415	SGO1	shugoshin	54.08	73.83	59.69	63.08	65.71	77.79	0.14
KLMA_10416	DAD2	DASH complex subunit DAD2	16.46	35.80	17.05	43.74	37.91	36.30	0.76
KLMA_10417	NUP133	nucleoporin NUP133	1405.00	1512.42	1426.43	854.52	1094.38	1063.15	-0.53
KLMA_10418	RPF2	ribosome biogenesis protein RPF2	1035.82	1077.26	1184.02	414.64	552.67	472.80	-1.19
KLMA_10419	folD	methylenetetrahydrofolate dehydrogenase [NAD+]	1840.02	1592.96	1890.53	756.95	1073.32	974.13	-0.92
KLMA_10420	RAD7	DNA repair protein RAD7	376.23	375.87	400.76	592.11	350.47	434.77	0.26
KLMA_10421	PRP39	pre-mRNA-processing factor 39	253.96	272.95	248.50	467.63	393.44	433.91	0.74

Locus_tag	UniProt_gene	Product	Unique exon reads						log2 Fold Change
			KmWT.1	KmWT.2	KmWT.3	Kmmig1.1	Kmmig1.2	Kmmig1.3	
KLMA_10422	BFA1	mitotic check point protein BFA1	351.54	426.21	315.49	771.25	561.09	649.13	0.86
KLMA_10423		hypothetical protein	3.53	1.12	0.00	0.00	0.00	0.00	-3.31
KLMA_10838		transposon Ty2-LR1 Gag-Pol polyprotein	323.33	337.83	345.95	323.81	172.71	171.14	-0.59
KLMA_10424	GSF2	glucose-signaling factor 2	10920.17	11129.45	10865.69	4883.19	6123.12	5537.05	-0.99
KLMA_10425	RSE1	pre-mRNA-splicing factor RSE1	1169.85	1054.89	1164.53	620.70	509.70	517.75	-1.04
KLMA_10426	AIM32	hypothetical protein	28.22	42.51	41.42	115.23	106.99	121.87	1.62
KLMA_10427	GAL80	galactose/lactose metabolism regulatory protein GAL80	12011.24	12103.80	13170.38	2693.91	2745.63	2656.16	-2.20
KLMA_10428	SUR7	protein SUR7	1295.65	1269.67	1282.69	1939.48	1540.89	1593.87	0.40
KLMA_10429		NADH dehydrogenase	1272.14	1475.50	1230.31	721.63	713.58	684.57	-0.91
KLMA_10430	PES4	RRM	64.67	66.00	56.03	740.97	330.25	457.24	3.03
KLMA_10431	LSB3	DUF500 super family	1065.21	1175.70	1081.70	968.90	820.57	919.67	-0.29
KLMA_10432	YSD83	NADB_Rossmann super family	484.40	473.19	438.53	1291.87	829.00	806.44	1.07
KLMA_10433	ARG4	argininosuccinate lyase	2846.44	1750.69	2581.21	1529.89	2282.27	2135.82	-0.27
KLMA_10434	DED81	asparaginyl-tRNA synthetase	2987.53	2449.84	2718.86	1732.58	2526.59	2406.36	-0.29
KLMA_10435		putative prolyl-tRNA synthetase YHR020W	3399.03	2892.83	3437.55	1571.10	2584.72	2643.19	-0.52
KLMA_10436	HIS2	histidinol-phosphatase	146.97	119.70	142.52	132.89	143.22	138.30	0.02
KLMA_10437	DAO1	D-amino-acid oxidase	720.72	500.04	739.40	558.46	597.32	604.18	-0.15
KLMA_10438		hypothetical protein	594.92	590.65	579.83	397.82	393.44	360.44	-0.62
KLMA_10439	PGS1	CDP-diacylglycerol--glycerol-3-phosphate 3-phosphatidyltransferase	352.72	355.73	310.62	429.78	351.31	362.16	0.17
KLMA_10440		hypothetical protein	203.40	220.37	214.39	471.83	262.85	248.93	0.62
KLMA_10441	RER1	protein RER1	730.13	517.94	666.32	891.52	884.60	867.81	0.47

Locus_tag	UniProt_gene	Product	Unique exon reads						log$_2$ Fold Change
			KmWT.1	KmWT.2	KmWT.3	Kmmig1.1	Kmmig1.2	Kmmig1.3	
KLMA_10442	DOM34	protein DOM34	550.24	476.55	546.94	779.66	828.16	762.36	0.59
KLMA_10443		hypothetical protein	51.73	35.80	47.51	433.15	147.43	229.05	2.59
KLMA_10444	AML1	N(6)-adenine-specific DNA methyltransferase-like 1	673.69	655.53	649.26	746.02	727.90	755.45	0.17
KLMA_10445	TFC3	transcription factor tau 138 kDa subunit	554.94	713.70	645.61	613.97	616.69	622.33	-0.05
KLMA_10446	NUP60	uncharacterized protein DDB_G0271670	563.17	563.80	622.46	475.20	566.99	566.15	-0.12
KLMA_10448	ERP1	protein ERP1	1506.11	1318.89	1393.54	900.77	1465.91	1274.06	-0.21
KLMA_10449	SWD1	COMPASS component SWD1	243.38	243.87	263.12	291.85	302.45	316.35	0.28
KLMA_10450	CDH1	APC/C activator protein CDH1	364.48	388.17	410.51	284.28	324.35	375.99	-0.24
KLMA_10451	RFA1	replication factor A protein 1	1285.07	1474.38	1231.53	1672.87	1337.01	1533.36	0.19
KLMA_10452	RPN14	26S proteasome regulatory subunit RPN14	174.01	209.19	144.96	225.40	190.40	203.12	0.23
KLMA_10453	COG7	conserved oligomeric Golgi complex subunit 7	123.45	159.97	126.69	65.60	145.75	136.57	-0.24
KLMA_10454	PMC1	calcium-transporting ATPase 2	1951.71	1712.65	1645.69	9593.96	5193.03	6337.44	1.99
KLMA_10456	SEN34	tRNA-splicing endonuclease subunit SEN34	81.13	38.03	62.12	117.75	90.15	96.81	0.75
KLMA_10457	PMA1	plasma membrane ATPase	34696.92	28017.72	29872.11	104007.00	122537.47	105578.19	1.84
KLMA_10458	KYE1	enoate reductase 1	20949.13	20334.83	18913.85	2724.19	2612.52	1737.35	-3.09
KLMA_10459	PUF4	pumilio homology domain family member 4	1570.77	1408.38	1633.51	881.43	1048.04	1003.51	-0.65
KLMA_10460	PDR1	GAL4-like Zn2Cys6 binuclear cluster DNA-binding domain	385.64	364.68	334.98	401.18	445.67	452.06	0.26
KLMA_10461	SPC97	spindle pole body component alp4	176.36	173.39	172.97	141.30	166.81	156.45	-0.17
KLMA_10462	ENO	enolase	411273.37	230220.51	373849.05	41283.36	66550.64	63455.65	-2.57

Locus_tag	UniProt_gene	Product	Unique exon reads						log$_2$ Fold Change
			KmWT.1	KmWT.2	KmWT.3	Kmmig1.1	Kmmig1.2	Kmmig1.3	
KLMA_10463		ctr super family	166.95	167.80	163.23	84.11	120.47	135.70	-0.55
KLMA_10464	FMO1	thiol-specific monooxygenase	115.22	202.48	115.72	157.28	96.88	219.55	0.13
KLMA_10465	STB5	protein STB5	497.33	494.44	505.52	570.24	460.84	523.80	0.05
KLMA_10466	SVP26	erv26 super family	676.04	522.41	565.21	483.61	411.13	420.08	-0.42
KLMA_10467		hypothetical protein	25.87	20.14	18.27	31.96	48.86	47.54	1.00
KLMA_10468	GND1	6-phosphogluconate dehydrogenase	8145.45	7858.52	7967.76	5336.52	5372.48	6444.62	-0.48
KLMA_10469	SSP1	hypothetical protein	37.62	44.75	48.73	159.80	88.46	123.60	1.50
KLMA_10471	MTM1	mitochondrial carrier protein MTM1	245.73	225.97	247.28	390.25	368.16	356.11	0.63
KLMA_10472		hypothetical protein	0.00	5.59	3.65	21.87	5.90	12.10	2.10
KLMA_10473	RAD2	DNA repair protein RAD2	108.17	121.93	109.63	243.07	144.91	197.94	0.79
KLMA_10475	TNA1	high-affinity nicotinic acid transporter	17717.05	4852.71	14559.05	2761.20	2827.35	2420.19	-2.21
KLMA_10476	KOG1	target of rapamycin complex 1 subunit KOG1	980.56	1127.60	1013.48	619.02	729.59	669.87	-0.63
KLMA_10477	IKI1	elongator complex protein 5	270.42	289.73	276.51	396.98	305.82	345.74	0.33
KLMA_10478	GPI16	GPI transamidase component GPI16	747.76	793.12	705.30	633.32	679.88	713.96	-0.15
KLMA_10479	HSH155	U2 snRNP component HSH155	210.46	276.31	237.53	484.45	402.70	462.43	0.90
KLMA_10480	APL6	AP-3 complex subunit beta	530.25	630.92	561.56	296.05	385.01	491.82	-0.56
KLMA_10481	ABZ2	aminodeoxychorismate lyase	256.31	365.80	255.81	448.28	285.60	327.59	0.27
KLMA_10482	BUD32	serine/threonine-protein kinase BUD32	201.05	265.12	215.61	138.77	208.09	190.16	-0.35
KLMA_10483	MES1	methionyl-tRNA synthetase	4805.20	3330.22	4426.67	1381.02	2091.87	2042.47	-1.19
KLMA_10484	HAS1	ATP-dependent RNA helicase HAS1	2306.78	1988.96	2190.19	664.44	818.05	716.55	-1.56
KLMA_10486		uncharacterized protein YGR266W	110.52	67.12	73.09	305.30	289.81	414.89	2.01
KLMA_10487	TDA1	probable serine/threonine-	2416.12	1786.48	2320.53	525.66	320.14	285.24	-2.53

Locus_tag	UniProt_gene	Product	Unique exon reads						log $_2$ Fold Change
			KmWT.1	KmWT.2	KmWT.3	Kmmig1.1	Kmmig1.2	Kmmig1.3	
		protein kinase YMR291W							
KLMA_10488	FOL2	GTP cyclohydrolase 1	854.75	486.61	734.53	560.99	627.65	732.97	-0.11
KLMA_10489		hypothetical protein	97.59	105.15	97.45	200.17	160.07	180.65	0.85
KLMA_10491	YTA7	TAT-binding homolog 7	2641.86	3317.92	2660.39	1333.92	1172.73	1243.80	-1.20
KLMA_10493	HER2	glutamyl-tRNA(Gln) amidotransferase subunit A	413.86	453.05	369.09	317.08	331.09	277.46	-0.42
KLMA_10494		dynamitin	68.19	83.90	75.52	129.52	153.33	129.65	0.86
KLMA_10495	SLH1	antiviral helicase SLH1	727.78	845.70	851.47	936.10	823.94	864.35	0.11
KLMA_10496	EFG1	rRNA-processing protein EFG1	325.68	312.10	328.89	193.44	213.15	227.32	-0.61
KLMA_10497		uncharacterized protein YMR295C	2512.53	2245.13	2236.48	4095.96	1984.03	1914.54	0.19
KLMA_10498	LCB1	serine palmitoyltransferase 1	2276.21	2534.86	2158.52	1545.02	1662.21	1685.49	-0.51
KLMA_10499	PRC1	carboxypeptidase Y	2467.85	5695.05	2837.02	7831.94	5225.89	6100.61	0.80
KLMA_10500	LIP1	ceramide synthase subunit LIP1	427.97	369.15	498.21	172.42	221.57	225.60	-1.06
KLMA_10501	TAF1	transcription initiation factor TFIID subunit 1	353.89	429.56	376.40	459.22	409.44	441.68	0.18
KLMA_10502		rtt102p super family	166.95	133.12	153.48	203.54	234.21	194.48	0.48
KLMA_10503	RNH70	RNA exonuclease 1	1172.20	1192.48	1207.16	686.30	744.75	772.73	-0.70
KLMA_10504	DYN3	cytoplasmic dynein intermediate light chain DYN3	42.33	76.07	75.52	97.56	70.77	83.84	0.38
KLMA_10505	ADE4	amidophosphoribosyltransferase	4882.80	3764.26	4877.38	2261.61	3360.64	2905.09	-0.67
KLMA_10506	ATM1	iron-sulfur clusters transporter ATM1	493.81	481.02	504.30	322.97	286.44	385.50	-0.57
KLMA_10507	PRE5	proteasome component PRE5	570.23	704.75	517.70	623.22	663.87	758.90	0.19
KLMA_10508	TGL3	lipase 3	659.58	858.00	726.00	942.83	713.58	790.88	0.13
KLMA_10509	ELP6	elongator complex protein 6	372.71	236.04	266.77	550.89	536.66	587.76	0.94
KLMA_10510	SKN7	HSF_DNA-bind	385.64	410.54	492.12	619.86	428.82	424.40	0.19

Locus_tag	UniProt_gene	Product	Unique exon reads						log2 Fold Change
			KmWT.1	KmWT.2	KmWT.3	Kmmig1.1	Kmmig1.2	Kmmig1.3	
		super family							
KLMA_10511	SET5	potential protein lysine methyltransferase SET5	340.96	328.88	343.51	491.18	406.07	447.74	0.41
KLMA_10512	FMP10	uncharacterized mitochondrial membrane protein FMP10	331.56	449.70	400.76	532.39	386.70	421.80	0.18
KLMA_10513	FAU1	5-formyltetrahydrofolate cyclo-ligase	1019.36	363.56	1110.93	435.67	303.29	412.30	-1.11
KLMA_10514	BAT1	branched-chain-amino-acid aminotransferase	10901.35	6083.22	10402.80	4508.07	4782.74	4912.99	-0.95
KLMA_10515		GAL4-like Zn2Cys6 binuclear cluster DNA-binding domain	242.20	251.70	248.50	240.54	185.34	239.43	-0.16
KLMA_10516	PGU1	polygalacturonase	116.40	110.75	110.85	534.91	182.82	174.60	1.40
KLMA_10517		uncharacterized protein conserved in bacteria	500.86	439.63	437.31	174.10	265.38	230.78	-1.04
KLMA_10518	INU1	inulinase	403.27	252.82	267.99	1036.18	494.53	533.31	1.16
KLMA_10519	ARN1	siderophore iron transporter ARN1	1206.30	703.63	1277.81	3386.94	1128.92	639.62	0.69
KLMA_10520		uncharacterized protein AN0679	58.79	25.73	34.11	189.24	252.74	306.85	2.66
KLMA_10521	FMP23	protein FMP23	144.61	117.46	182.72	151.39	48.86	37.17	-0.90
KLMA_10522	ZTA1	probable quinone oxidoreductase	392.69	606.31	497.00	1256.54	911.56	998.33	1.08
KLMA_10523		hypothetical protein	48.20	58.17	91.36	198.49	151.65	150.40	1.34
KLMA_10524	SES1	seryl-tRNA synthetase	2310.31	2306.66	2325.40	1068.99	1764.15	1618.93	-0.64
KLMA_10525		atg31 super family	43.50	44.75	56.03	107.66	74.98	120.15	1.07
KLMA_10526	FAL1	ATP-dependent RNA helicase FAL1	111.69	162.20	144.96	149.71	148.28	127.92	0.02
KLMA_10527	TLG2	t-SNARE affecting a late Golgi compartment protein 2	205.75	242.75	226.57	266.62	265.38	274.00	0.26

Locus_tag	UniProt_gene	Product	Unique exon reads						log2 Fold Change
			KmWT.1	KmWT.2	KmWT.3	Kmmig1.1	Kmmig1.2	Kmmig1.3	
KLMA_10528		pH-response regulator protein palI/RIM9	913.54	1083.97	786.91	1444.10	1037.09	1331.97	0.45
KLMA_10529		hypothetical protein	4.70	2.24	0.00	2.52	5.05	1.73	0.42
KLMA_10530		UPF0726 protein YFR011C	269.24	236.04	231.44	250.64	425.45	345.74	0.47
KLMA_10531	UBP6	ubiquitin carboxyl-terminal hydrolase 6	681.92	937.43	615.15	913.39	1080.90	1019.94	0.43
KLMA_10532	TAT2	tryptophan permease	4705.27	2516.96	4284.15	1358.31	1288.99	1040.68	-1.64
KLMA_10533	DIS3	exosome complex exonuclease DIS3	1656.60	1556.04	1589.65	1121.97	1088.48	1176.39	-0.50
KLMA_10534	GCN20	protein GCN20	1073.44	1161.16	1093.88	578.65	767.50	759.77	-0.66
KLMA_10535	TSR4	uncharacterized protein YOL022C	385.64	390.41	455.58	410.44	347.94	373.40	-0.12
KLMA_10536	IFM1	translation initiation factor IF-2	392.69	491.09	420.25	407.07	328.57	358.71	-0.25
KLMA_10537		trimethyllysine dioxygenase	562.00	879.26	566.43	1316.26	1119.65	1170.33	0.84
KLMA_10539		inorganic pyrophosphatase	90.53	55.93	91.36	101.77	70.77	101.13	0.20
KLMA_10540	PGK	phosphoglycerate kinase	133709.73	106244.95	135392.30	12779.05	17356.72	18829.08	-2.94
KLMA_10541	POL4	DNA polymerase IV	199.87	171.15	163.23	351.56	244.32	282.64	0.72
KLMA_10542		HAD_like super family	30.57	26.85	30.45	40.37	24.43	49.27	0.38
KLMA_10543		uncharacterized protein YCR016W	183.41	166.68	129.12	137.09	121.32	160.77	-0.19
KLMA_10544		glycoside hydrolase	166.95	140.95	142.52	161.48	152.49	210.90	0.22
KLMA_10545	HGT1	high-affinity glucose transporter	139.91	156.61	172.97	604.72	1686.64	1899.85	3.16
KLMA_10546	HGT1	high-affinity glucose transporter	1042.87	1145.50	1046.37	2363.37	1108.70	1413.22	0.59
KLMA_10547	HGT1	high-affinity glucose transporter	140235.02	127692.84	121734.67	113584.97	122776.73	110556.87	-0.17
KLMA_10548	CWH43	protein CWH43	3732.94	2652.32	3388.83	909.18	1259.50	1203.18	-1.54
KLMA_10549	GLC3	1,4-alpha-glucan-branching enzyme	739.53	984.41	847.82	1971.44	1402.72	1470.27	0.91
KLMA_10550	UBC8	ubiquitin-conjugating enzyme E2-24 kDa	242.20	303.15	282.61	592.95	418.71	466.75	0.84
KLMA_10551		N-terminal	21.16	7.83	17.05	37.85	53.92	57.05	1.70

Locus_tag	UniProt_gene	Product	Unique exon reads						log2 Fold Change
			KmWT.1	KmWT.2	KmWT.3	Kmmig1.1	Kmmig1.2	Kmmig1.3	
		acetyltransferase C complex subunit MAK31							
KLMA_10552	MAK32	protein MAK32	223.39	203.59	208.30	507.16	386.70	452.92	1.08
KLMA_10553	VAC8	vacuolar protein 8	800.67	1000.07	805.18	698.92	757.39	809.90	-0.20
KLMA_10556	EDC3	enhancer of mRNA-decapping protein 3	226.92	200.24	264.33	121.95	164.28	197.07	-0.52
KLMA_10557	rhb1	GTP-binding protein rhb1	230.44	212.54	272.86	211.11	182.82	185.84	-0.30
KLMA_10558	ARDH	D-arabinitol 2-dehydrogenase [ribulose-forming]	1011.13	1160.04	986.68	2381.04	1480.23	1560.16	0.78
KLMA_10559	NPP1	sulfatase super family	258.66	193.53	258.24	259.89	238.42	248.07	0.07
KLMA_10560		glutathione transferase 3	517.32	372.51	404.42	1656.89	1634.41	1463.35	1.88
KLMA_10561		plasma membrane ATPase proteolipid 2	70.54	102.92	91.36	111.86	283.07	51.00	0.75
KLMA_10562	EAF5	chromatin modification-related protein EAF5	71.72	58.17	74.31	157.28	91.83	103.72	0.79
KLMA_10563	MMS21	e3 SUMO-protein ligase MMS21	110.52	96.20	91.36	163.17	110.36	141.75	0.48
KLMA_10564	SLM5	asparaginyl-tRNA synthetase	265.71	337.83	260.68	299.42	301.61	287.83	0.04
KLMA_10565		PGA2 super family	356.25	325.53	375.18	299.42	415.34	360.44	0.03
KLMA_10566	RPC31	DNA-directed RNA polymerase III subunit RPC7	228.09	210.31	248.50	201.85	203.04	204.85	-0.17
KLMA_10567		hypothetical protein	109.34	146.54	90.14	504.64	322.67	369.08	1.79
KLMA_10568	INN1	uncharacterized protein YNL152W	182.24	208.07	181.50	202.70	201.35	191.89	0.06
KLMA_10569	THO2	THO complex subunit 2	930.00	1162.28	1084.13	1154.77	1010.97	911.89	-0.05
KLMA_10570		SF3b10 super family	17.64	29.08	14.62	66.44	53.92	28.52	1.28
KLMA_10571		nucleotide-binding	208.10	238.27	199.77	319.60	326.88	345.74	0.62
KLMA_10572	SRV2	adenylyl cyclase-associated protein	1368.55	1740.62	1453.22	1668.66	1840.81	2065.81	0.29
KLMA_10573		37S ribosomal protein NAM9	1596.64	1727.20	1510.48	773.77	1168.52	986.23	-0.72

Locus_tag	UniProt_gene	Product	Unique exon reads						log2 Fold Change
			KmWT.1	KmWT.2	KmWT.3	Kmmig1.1	Kmmig1.2	Kmmig1.3	
KLMA_10574	EAF7	chromatin modification-related protein EAF7	199.87	159.97	185.16	260.73	265.38	241.15	0.49
KLMA_10575	FCP1	RNA polymerase II subunit A C-terminal domain phosphatase	311.57	302.04	309.40	382.68	322.67	373.40	0.22
KLMA_10576	TAF8	transcription initiation factor TFIID subunit 8	284.53	332.24	291.13	257.36	249.37	233.38	-0.30
KLMA_10577	SCS7	inositolphosphorylceramide-B C-26 hydroxylase	5394.24	2840.25	5291.54	2252.36	1753.20	1694.13	-1.25
KLMA_10578		uncharacterized protein YML108W	104.64	77.19	101.10	26.07	50.55	25.07	-1.47
KLMA_10579	ZDS2	protein ZDS2	304.51	364.68	278.95	308.67	248.53	329.32	-0.10
KLMA_10580	RCE1	CAAX prenyl protease 2	291.58	221.49	300.88	200.17	298.24	278.32	-0.07
KLMA_10581	COQ5	ubiquinone biosynthesis methyltransferase COQ5	823.01	766.28	903.85	489.50	671.45	595.54	-0.51
KLMA_10582	BUL1	ubiquitin ligase-binding protein BUL1	724.25	916.17	768.64	577.81	437.25	449.46	-0.72
KLMA_10583	CTK3	CTD kinase subunit gamma	191.64	233.80	230.23	869.65	395.12	503.92	1.43
KLMA_10584		hypothetical protein	691.33	633.16	617.59	524.82	518.12	540.22	-0.29
KLMA_10585	DSK2	ubiquitin domain-containing protein DSK2	1027.59	978.82	883.14	1545.87	1123.02	1281.84	0.45
KLMA_10586		pre-mRNA leakage protein 39	208.10	215.90	229.01	201.01	204.72	201.39	-0.10
KLMA_10587	URA5	orotate phosphoribosyltransferase	3142.72	2304.42	2855.29	1222.90	1870.30	1688.08	-0.80
KLMA_10588	SEC65	signal recognition particle SEC65 subunit	455.01	347.90	421.47	368.38	412.81	405.38	-0.04
KLMA_10589	RRN9	RNA polymerase I-specific transcription initiation factor RRN9	157.55	145.42	129.12	203.54	197.98	213.50	0.51
KLMA_10590	MDM1	structural protein MDM1	371.53	363.56	358.13	698.08	536.66	487.50	0.66

Locus_tag	UniProt_gene	Product	Unique exon reads						log2 Fold Change
			KmWT.1	KmWT.2	KmWT.3	Kmmig1.1	Kmmig1.2	Kmmig1.3	
KLMA_10591	TMA23	protein TMA23	174.01	167.80	209.52	185.87	139.01	125.33	-0.29
KLMA_10592	KYE1	enoate reductase 1	425.61	493.32	367.87	1688.00	887.13	921.40	1.44
KLMA_10593		hypothetical protein	624.31	833.39	559.12	1428.12	1041.30	1089.95	0.82
KLMA_10594	SEC9	protein transport protein SEC9	732.48	795.36	665.10	864.61	763.28	906.71	0.21
KLMA_10595	TIF6	eukaryotic translation initiation factor 6	3751.75	3027.07	3625.14	2373.47	2416.22	2201.51	-0.57
KLMA_10596		hemocyanin	7.05	6.71	0.00	93.36	16.01	33.71	3.37
KLMA_10597	RLF2	chromatin assembly factor 1 subunit p90	365.65	349.02	417.82	436.51	354.68	383.77	0.05
KLMA_10598	RRT8	DUF540	10.58	19.02	14.62	94.20	63.19	45.81	2.20
KLMA_10599	LCB2	serine palmitoyltransferase 2	2230.36	1872.62	2052.54	1206.08	1472.65	1479.77	-0.57
KLMA_10600		uncharacterized ABC transporter ATP-binding protein YDR061W	438.55	344.54	364.22	765.36	604.90	662.96	0.83
KLMA_10601		PQ-loop super family	112.87	60.41	132.78	203.54	123.84	205.72	0.80
KLMA_10602		vacuolar protein sorting-associated protein 51	69.37	91.73	68.22	137.09	165.13	146.94	0.97
KLMA_10603	IRS4	increased rDNA silencing protein 4	92.88	143.19	80.40	268.30	161.76	208.31	1.01
KLMA_10604	IML2	mitochondrial outer membrane protein IML2	1894.10	1747.33	1730.96	1173.28	1058.99	1051.92	-0.71
KLMA_10605		RING finger protein YKR017C	261.01	318.82	299.66	789.75	608.27	619.74	1.20
KLMA_10606	ARP4	actin-related protein 4	892.38	816.61	823.45	985.72	1077.53	1038.09	0.29
KLMA_10607	FCJ1	uncharacterized protein YKR016W	1188.66	1315.53	1156.00	742.65	891.34	885.96	-0.54
KLMA_10608		hypothetical protein	172.83	174.51	144.96	296.89	155.02	173.74	0.35
KLMA_10609	YPT52	GTP-binding protein YPT52	670.17	690.21	592.01	694.71	736.33	671.60	0.11
KLMA_10610	SCP160	protein SCP160	1785.93	1722.72	1805.26	997.50	1545.10	1417.54	-0.42
KLMA_10611	PRY3	SCP_PRY1_like	47.03	49.22	65.78	74.01	55.60	48.40	0.14

Locus_tag	UniProt_gene	Product	Unique exon reads						log$_2$ Fold Change
			KmWT.1	KmWT.2	KmWT.3	Kmmig1.1	Kmmig1.2	Kmmig1.3	
KLMA_10612	PRY2	protein PRY1	786.56	795.36	710.17	226.24	264.54	401.06	-1.36
KLMA_10613		uncharacterized protein KLLA0D02464g	24.69	60.41	37.76	216.15	175.24	203.99	2.27
KLMA_10614	MPD1	PDI_a_MPD1_like	139.91	164.44	141.30	268.30	185.34	225.60	0.61
KLMA_10615	rplM	54S ribosomal protein L23	272.77	361.32	359.35	176.62	323.51	280.91	-0.35
KLMA_10616	CRN1	DUF1900 super family	1080.49	1118.65	1137.73	1031.98	1034.56	1063.15	-0.09
KLMA_10617	MAG2	RING-finger protein MAG2	504.39	528.00	437.31	720.79	534.13	528.98	0.28
KLMA_10618	SPC2	signal peptidase complex subunit SPC2	312.74	314.34	315.49	374.27	417.87	352.66	0.28
KLMA_10619	TDA5	uncharacterized oxidoreductase YLR426W	204.58	151.02	250.93	100.93	99.41	127.06	-0.89
KLMA_10620	TUS1	rho1 guanine nucleotide exchange factor TUS1	547.89	635.39	515.27	536.60	506.33	490.09	-0.15
KLMA_10621	CYB2	cytochrome b2	917.07	1061.60	1280.25	720.79	511.38	596.40	-0.83
KLMA_10622	YKT6	synaptobrevin homolog YKT6	348.02	332.24	370.31	885.63	821.42	779.65	1.24
KLMA_10623	MIA40	mitochondrial intermembrane space import and assembly protein 40	777.16	1075.02	818.58	1466.81	1076.69	1140.95	0.46
KLMA_10624	SPP382	pre-mRNA-splicing factor SPP382	106.99	136.48	84.05	477.72	248.53	329.32	1.69
KLMA_10625	ATG17	autophagy-related protein 17	72.90	91.73	70.65	485.29	326.88	276.59	2.21
KLMA_10626	MST1	threonyl-tRNA synthetase	268.07	262.88	283.82	424.73	404.39	388.09	0.58
KLMA_10627		uncharacterized protein YNL040W	491.45	501.16	468.98	514.73	544.24	513.43	0.11
KLMA_10628	SDS22	protein phosphatase 1 regulatory subunit SDS22	338.61	390.41	353.26	497.91	454.10	481.44	0.40
KLMA_10629	ACP1	acyl carrier protein	460.89	455.29	504.30	278.39	486.11	420.08	-0.26
KLMA_10630		DOCK-like protein YLR422W	1957.59	1708.18	1717.56	970.58	1312.58	1157.37	-0.65
KLMA_10631	RPN13	26S proteasome regulatory subunit	442.07	534.71	430.00	456.69	440.62	417.48	-0.10

Locus_tag	UniProt_gene	Product	Unique exon reads						log$_2$ Fold Change
			KmWT.1	KmWT.2	KmWT.3	Kmmig1.1	Kmmig1.2	Kmmig1.3	
		RPN13							
KLMA_10633	URA4	dihydroorotase	1014.65	850.17	860.00	557.62	769.18	817.68	-0.35
KLMA_10634	DPH2	diphthamide biosynthesis protein 2	378.58	322.17	320.37	398.66	369.85	401.92	0.20
KLMA_10635	DAL5	allantoate permease	276.30	244.98	197.34	693.03	413.66	520.34	1.18
KLMA_10636	CNB1	calcineurin subunit B	250.43	244.98	231.44	502.11	375.74	382.04	0.79
KLMA_10637		hypothetical protein	9.41	11.19	10.96	190.92	73.30	63.96	3.38
KLMA_10638	CDC73	cell division control protein 73	733.65	715.94	739.40	698.92	931.78	828.92	0.17
KLMA_10639		putative ATP-dependent RNA helicase YLR419W	431.49	423.97	448.27	391.93	338.68	385.50	-0.22
KLMA_10640	NPY1	NADH pyrophosphatase	507.91	460.88	414.16	698.92	530.76	647.40	0.44
KLMA_10641	HRR25	casein kinase I homolog HRR25	1824.73	1695.87	1735.83	1152.25	1093.54	985.36	-0.70
KLMA_10642	TPK2	cAMP-dependent protein kinase type 2	732.48	965.40	757.67	1182.53	1177.78	1117.61	0.50
KLMA_10643	MNP1	39S ribosomal protein L12	775.98	664.48	775.95	412.12	689.15	544.54	-0.43
KLMA_10645	RPB9	DNA-directed RNA polymerase II subunit RPB9	142.26	144.31	187.59	90.83	189.56	136.57	-0.18
KLMA_10646	AFT1	AFT super family	1240.39	1240.58	1229.09	1703.14	1267.93	1474.59	0.26
KLMA_10647		hypothetical protein	174.01	198.00	160.79	780.50	717.79	739.02	2.07
KLMA_10648	HSF	heat shock factor protein	1792.99	2408.45	1796.74	1505.49	1104.49	1217.01	-0.65
KLMA_10649	DLD1	D-lactate dehydrogenase [cytochrome]	487.93	597.36	490.90	1144.68	968.01	1013.02	0.99
KLMA_10650	MPS2	hypothetical protein	37.62	67.12	59.69	72.33	67.40	70.01	0.35
KLMA_10651		smr domain-containing protein YPL199C	279.82	284.14	325.24	370.07	293.18	332.78	0.16
KLMA_10652		hypothetical protein	11637.36	8791.47	11847.50	3714.12	7500.57	5920.82	-0.91
KLMA_10653	SNQ2	protein SNQ2	2002.27	1625.40	1907.59	1991.63	1585.54	1790.94	-0.04
KLMA_10654	FUR4	uracil permease	1362.67	943.02	1075.61	422.21	356.37	480.58	-1.43

Locus_tag	UniProt_gene	Product	Unique exon reads						log$_2$ Fold Change
			KmWT.1	KmWT.2	KmWT.3	Kmmig1.1	Kmmig1.2	Kmmig1.3	
KLMA_10655	CDR4	ABC_PDR_domain2	3731.76	3030.42	3731.12	7509.81	6251.18	6523.28	0.95
KLMA_10656	CHS3	chitin synthase 3	1235.69	1107.46	1191.33	667.80	680.72	693.21	-0.79
KLMA_10657		uncharacterized hydrolase HI0588	74.07	108.51	87.71	180.83	112.89	118.42	0.61
KLMA_10658	CHS2	chitin synthase 2	691.33	474.31	606.63	901.61	1321.85	1244.67	0.97
KLMA_10659	engD	uncharacterized GTP-binding protein OLA1	4711.14	4894.10	4711.71	2119.47	3584.74	3098.71	-0.70
KLMA_10660	SCO1	SCO	1782.40	1457.60	1671.27	457.54	650.39	630.98	-1.50
KLMA_10661	SIT4	serine/threonine-protein phosphatase PP1-1	961.75	811.02	961.10	793.12	846.69	681.97	-0.24
KLMA_10662	THP1	nuclear mRNA export protein THP1	372.71	369.15	406.85	306.15	286.44	305.12	-0.36
KLMA_10663	NDK1	nucleoside diphosphate kinase	1681.29	1944.21	1624.98	1613.99	2757.43	2285.35	0.34
KLMA_10664	UPF3	smg4_UPF3 super family	159.90	214.78	144.96	177.46	252.74	233.38	0.35
KLMA_10666		peroxisomal membrane protein 2	111.69	119.70	105.98	116.07	155.86	172.01	0.40
KLMA_10667	SMD1	small nuclear ribonucleoprotein Sm D1	101.11	114.10	105.98	349.04	303.29	276.59	1.53
KLMA_10668	PRP38	pre-mRNA-splicing factor 38	141.09	186.81	149.83	282.60	261.17	245.48	0.72
KLMA_10669	MRPL25	54S ribosomal protein L25	253.96	225.97	263.12	154.75	253.59	229.05	-0.22
KLMA_10670	DPS1	aspartyl-tRNA synthetase	3990.42	3873.89	3962.56	1645.11	2265.42	2076.18	-0.98
KLMA_10671	OCA5	uncharacterized protein YHL029C	3355.53	2575.13	3273.11	486.13	660.50	677.65	-2.33
KLMA_10672	PRP19	pre-mRNA-splicing factor 19	437.37	399.36	417.82	330.54	454.10	379.45	-0.11
KLMA_10673	GPX2	glutathione peroxidase 2	98.76	134.24	93.80	273.34	111.21	119.28	0.62
KLMA_10674	ENT4	epsin-4	79.95	93.97	90.14	360.81	305.82	280.91	1.84
KLMA_10675	COX19	cytochrome c oxidase assembly protein COX19	47.03	53.70	56.03	47.10	80.04	51.86	0.19
KLMA_10676		zinc finger	638.42	715.94	740.62	973.95	799.51	666.42	0.22
KLMA_10677	MET17	protein MET17	27407.41	14478.69	23053.04	4879.82	8280.71	8911.49	-1.56

Locus_tag	UniProt_gene	Product	Unique exon reads						log2 Fold Change
			KmWT.1	KmWT.2	KmWT.3	Kmmig1.1	Kmmig1.2	Kmmig1.3	
KLMA_10678	BPT1	bile pigment transporter 1	805.37	522.41	717.48	1121.97	904.82	1025.99	0.58
KLMA_10679	RAN1	putative copper-transporting ATPase 3	1069.91	1883.81	1215.69	9019.51	6105.43	5529.27	2.31
KLMA_10680	CDC3	cell division control protein 3	1386.18	1412.86	1320.45	1321.30	1679.06	1514.35	0.13
KLMA_10681	CDC12	cell division control protein 12	1247.45	1142.14	1175.49	868.81	1243.50	1185.03	-0.11
KLMA_10682	TRR1	thioredoxin reductase	935.88	708.11	1042.72	483.61	645.34	643.08	-0.60
KLMA_10683	XYL1	NAD(P)H-dependent D-xylose reductase	9325.88	9902.29	9033.62	7893.34	10318.66	11255.61	0.06
KLMA_10684		vacuolar integral membrane protein YDR352W	144.61	162.20	158.36	82.42	101.94	99.40	-0.71
KLMA_10685	SBE2	protein SBE2	1065.21	1372.58	1037.84	808.26	684.09	786.56	-0.61
KLMA_10686	KIC1	serine/threonine-protein kinase KIC1	696.03	736.07	637.08	545.01	438.09	560.10	-0.42
KLMA_10687	ATP22	mitochondrial translation factor ATP22	166.95	191.29	230.23	329.69	214.83	211.77	0.36
KLMA_10688	BIG1	protein BIG1	94.06	97.32	93.80	238.86	161.76	211.77	1.10
KLMA_10689	PEP12	syntaxin PEP12	185.77	213.66	188.81	741.81	432.19	480.58	1.49
KLMA_10690	CIA1	probable cytosolic iron-sulfur protein assembly protein 1	218.69	198.00	249.72	281.75	342.05	275.73	0.43
KLMA_10691	CYC2	cytochrome c mitochondrial import factor CYC2	111.69	100.68	144.96	162.32	173.55	169.41	0.50
KLMA_10692	MSW1	tryptophanyl-tRNA synthetase	625.49	407.19	447.05	156.44	228.31	185.84	-1.37
KLMA_10693	HIR2	protein HIR2	937.06	916.17	890.45	825.92	891.34	883.37	-0.08
KLMA_10694	CKB2	casein kinase II subunit beta'	640.77	562.68	592.01	471.83	608.27	541.95	-0.15
KLMA_10695	NDE1	external NADH-ubiquinone oxidoreductase 1	18621.19	13668.79	17578.78	7564.48	9206.59	8480.17	-0.98
KLMA_10696		GAL4	433.84	962.04	406.85	1136.27	834.05	843.61	0.64
KLMA_10697		hypothetical protein	223.39	337.83	291.13	332.22	168.50	188.43	-0.31

Locus_tag	UniProt_gene	Product	Unique exon reads						$\log_2$ Fold Change
			KmWT.1	KmWT.2	KmWT.3	Kmmig1.1	Kmmig1.2	Kmmig1.3	
KLMA_10698	SUB2	ATP-dependent RNA helicase SUB2	5381.31	5089.86	5198.96	2405.43	4198.06	3760.80	-0.60
KLMA_10699		hypothetical protein	285.70	248.34	281.39	390.25	308.35	333.64	0.34
KLMA_10700	RPS16	40S ribosomal protein S16	16608.34	12946.14	16290.00	5623.32	10857.85	8745.53	-0.86
KLMA_10701	RPL13A	ribosomal_L13e super family	12461.55	11468.40	12287.24	4404.62	7802.18	6644.29	-0.94
KLMA_10702	RPP1A	60S acidic ribosomal protein P1-alpha	5873.94	4720.71	5993.18	2444.96	4269.67	3156.62	-0.75
KLMA_10703	SIP5	protein SIP5	308.04	415.02	280.17	800.69	501.27	542.81	0.88
KLMA_10704	bioA	uncharacterized aminotransferase C1771.03c	590.22	432.92	566.43	289.32	137.32	203.99	-1.33
KLMA_10705	UTR4	enolase-phosphatase E1	268.07	380.34	336.20	272.50	320.98	371.67	-0.03
KLMA_10706	RAD23	UV excision repair protein RAD23	759.52	793.12	744.28	363.34	561.93	532.44	-0.66
KLMA_10707	ANP1	mannan polymerase II complex ANP1 subunit	806.55	844.58	1137.73	857.04	1000.86	1023.39	0.05
KLMA_10708	HYP2	eukaryotic translation initiation factor 5A-2	10849.62	7736.59	9853.42	5259.98	8616.86	6984.84	-0.45
KLMA_10709	MCM3	DNA replication licensing factor MCM3	805.37	986.65	722.35	539.12	616.69	643.94	-0.48
KLMA_10710	SPF1	probable cation-transporting ATPase 1	9506.94	6991.57	9045.81	4362.57	5487.90	5144.63	-0.77
KLMA_10711	TAH11	hypothetical protein	333.91	376.99	483.60	157.28	139.01	175.46	-1.34
KLMA_10712		heat shock protein SSC1	5671.71	6145.87	5301.29	2843.62	4144.15	3666.59	-0.68
KLMA_10713	VPS55	vacuolar protein sorting-associated protein 55	335.08	302.04	319.15	481.93	492.85	455.51	0.58
KLMA_10714	BUD16	putative pyridoxal kinase BUD16	373.88	232.68	369.09	238.86	362.27	369.08	-0.01
KLMA_10715	SOU2	sorbose reductase homolog SOU2	116.40	121.93	69.43	5511.46	2366.52	3358.88	5.19
KLMA_10716		DNA polymerase delta subunit 3	122.28	86.14	86.49	121.95	96.04	122.74	0.21
KLMA_10717	VMA3	v-type proton ATPase subunit c	3209.74	3200.46	3090.39	6604.83	4743.99	4035.67	0.70

Locus_tag	UniProt_gene	Product	Unique exon reads						log2 Fold Change
			KmWT.1	KmWT.2	KmWT.3	Kmmig1.1	Kmmig1.2	Kmmig1.3	
KLMA_10718	NUP85	nucleoporin NUP85	971.15	1053.77	1059.77	704.81	871.96	828.05	-0.36
KLMA_10719	URB2	nucleolar pre-ribosomal-associated protein 2	1312.11	1211.50	1374.05	682.94	695.89	590.35	-0.98
KLMA_10720	GEF1	protein GEF1	721.90	529.12	733.31	528.18	655.45	703.58	-0.07
KLMA_10721	SNU13	13 kDa ribonucleoprotein-associated protein	2165.69	1437.47	2039.14	1200.19	1713.60	1461.62	-0.37
KLMA_10722	HUL4	probable E3 ubiquitin-protein ligase HUL4	76.42	232.68	75.52	513.05	261.17	317.22	1.50
KLMA_10723	RIP1	cytochrome b-c1 complex subunit Rieske	5676.42	3943.24	5453.55	2898.29	4035.47	3241.33	-0.57
KLMA_10724		uncharacterized protein YEL023C	115.22	134.24	136.43	281.75	141.54	172.01	0.63
KLMA_10725	RAD26	DNA repair and recombination protein RAD26	228.09	210.31	183.94	1952.94	632.70	850.52	2.46
KLMA_10726	PET191	mitochondrial protein PET191	219.86	314.34	239.97	248.95	238.42	280.05	-0.01
KLMA_10727	RAV1	regulator of V-ATPase in vacuolar membrane protein 1	563.17	672.31	609.06	650.98	540.87	509.97	-0.12
KLMA_10728	CPR7	peptidyl-prolyl cis-trans isomerase CYP7	138.74	158.85	118.16	184.19	177.76	190.16	0.41
KLMA_10729	GEA2	ARF guanine-nucleotide exchange factor 2	1965.82	2026.99	1901.50	1165.71	1431.37	1407.17	-0.56
KLMA_10730		mito_carr super family	191.64	318.82	242.41	528.18	331.94	325.00	0.65
KLMA_10731	URA3	orotidine 5'-phosphate decarboxylase	466.76	437.39	490.90	477.72	529.92	513.43	0.13
KLMA_10732	TIM9	mitochondrial import inner membrane translocase subunit TIM9	157.55	152.14	125.47	163.17	240.95	180.65	0.43
KLMA_10733	ALF1	cell polarity protein alp11	232.79	213.66	183.94	284.28	315.93	228.19	0.39
KLMA_10734	LSM7	sm_like super family	212.81	210.31	205.86	207.74	324.35	266.22	0.34
KLMA_10735	FUR1	uracil	1976.40	1028.04	1637.16	372.59	577.94	493.55	-1.68

Locus_tag	UniProt_gene	Product	Unique exon reads						log2 Fold Change
			KmWT.1	KmWT.2	KmWT.3	Kmmig1.1	Kmmig1.2	Kmmig1.3	
		phosphoribosyltransferase							
KLMA_10736	ro-4	actin-like protein	225.74	161.09	140.08	100.09	164.28	153.85	-0.33
KLMA_10737		UPF0652 protein	399.75	483.26	398.33	334.74	419.55	371.67	-0.19
KLMA_10738		PH domain-containing protein YHR131C	1653.07	2019.16	1769.94	1508.86	1064.89	1136.62	-0.55
KLMA_10739	KTR3	probable mannosyltransferase KTR3	1827.08	1236.11	1583.56	696.40	1187.89	1192.81	-0.59
KLMA_10740		esterase_lipase super family	139.91	123.05	133.99	181.67	197.98	143.48	0.40
KLMA_10741	LDH1	putative peroxisomal lipase YBR204C	71.72	57.05	77.96	142.14	134.80	133.11	0.99
KLMA_10742	ALD6	magnesium-activated aldehyde dehydrogenase	1109.89	807.67	1071.95	4560.22	844.16	1141.81	1.13
KLMA_10743	COS111	F-box protein COS111	677.22	743.90	762.55	2395.34	1116.28	1084.76	1.07
KLMA_10744	TIM50	mitochondrial import inner membrane translocase subunit TIM50	1134.58	1191.36	1053.68	809.10	858.48	897.20	-0.40
KLMA_10745	VPS28	vacuolar protein sorting-associated protein 28	124.63	128.64	138.87	211.95	237.58	194.48	0.72
KLMA_10746		uncharacterized protein YPL066W	182.24	247.22	209.52	306.15	283.91	304.25	0.48
KLMA_10747	AAH1	adenosine deaminase	1115.77	1007.90	1230.31	444.92	351.31	365.62	-1.53
KLMA_10748	MCM7	DNA replication licensing factor CDC47	629.01	596.24	638.30	457.54	599.84	615.42	-0.16
KLMA_10749		uncharacterized protein YPL067C	217.51	238.27	213.17	516.41	438.09	527.26	1.15
KLMA_10750		hypothetical protein	91.71	92.85	90.14	103.45	85.09	101.99	0.08
KLMA_10751		degradation in the endoplasmic reticulum protein 1	148.14	153.26	177.85	319.60	272.12	269.68	0.85
KLMA_10752	BTS1	geranylgeranyl pyrophosphate synthetase	87.00	93.97	107.20	202.70	191.24	155.58	0.93
KLMA_10753	MUK1	protein MUK1	326.85	366.92	339.86	595.47	518.97	564.42	0.70

Locus_tag	UniProt_gene	Product	Unique exon reads						$\log_2$ Fold Change
			KmWT.1	KmWT.2	KmWT.3	Kmmig1.1	Kmmig1.2	Kmmig1.3	
KLMA_10754		uncharacterized protein YPL071C	32.92	24.61	32.89	154.75	94.36	112.37	2.00
KLMA_10755	BEM1	bud emergence protein 1	787.74	712.58	744.28	541.64	667.24	637.03	-0.28
KLMA_10756	UBP16	hypothetical protein	292.76	437.39	252.15	893.20	821.42	885.10	1.40
KLMA_10757	YTA6	probable 26S protease subunit YTA6	78.77	110.75	75.52	500.43	326.04	316.35	2.11
KLMA_10758	GCR1	glycolytic genes transcriptional activator GCR1	5105.01	4549.55	4682.48	524.82	726.22	675.92	-2.90
KLMA_10761	TAF5	transcription initiation factor TFIID subunit 5	1330.92	1200.31	1192.55	773.77	903.98	917.08	-0.52
KLMA_10762	GPI2	phosphatidylinositol N-acetylglucosaminyltransferase GPI2 subunit	380.94	256.17	333.77	210.26	221.57	218.68	-0.58
KLMA_10763	RAG2	glucose-6-phosphate isomerase	54067.05	35154.71	47114.69	10058.22	16410.61	16380.36	-1.67
KLMA_10764	MSI1	chromatin assembly factor 1 subunit p50	190.47	165.56	137.65	143.82	182.82	148.67	-0.06
KLMA_10765	ATP4	ATP synthase subunit 4	6140.83	5891.93	6027.29	4122.87	5129.00	4762.59	-0.37
KLMA_10766	AIM4	hypothetical protein	118.75	116.34	108.41	103.45	130.58	111.50	0.01
KLMA_10767	MED8	mediator of RNA polymerase II transcription subunit 8	181.06	128.64	164.45	132.89	172.71	173.74	0.02
KLMA_10768	RIM2	mitochondrial carrier protein RIM2	791.27	666.72	858.78	372.59	358.90	356.11	-1.09
KLMA_10769	RPL21A	60S ribosomal protein L21-A	13708.99	11527.69	13266.61	4221.27	9295.89	7286.50	-0.89
KLMA_10770		hypothetical protein	15222.16	12443.87	14880.63	4306.22	9042.31	7398.87	-1.04
KLMA_10771	LYS21	homocitrate synthase	2349.11	2016.93	2248.66	1925.18	2721.20	2387.34	0.09
KLMA_10772	MOT1	DUF3535	1923.49	1882.69	2168.26	917.59	1073.32	1089.95	-0.96
KLMA_10773		hypothetical protein	16.46	23.49	25.58	26.07	25.27	19.88	0.12
KLMA_10774	GDT1	UPF0016 membrane protein YBR187W	526.73	528.00	585.92	2264.97	1758.25	1503.11	1.75
KLMA_10775	PCH2	pachytene	72.90	68.24	79.18	322.13	237.58	230.78	1.84

Locus_tag	UniProt_gene	Product	Unique exon reads						log$_2$ Fold Change
			KmWT.1	KmWT.2	KmWT.3	Kmmig1.1	Kmmig1.2	Kmmig1.3	
		checkpoint protein 2							
KLMA_10776	SEN54	tRNA-splicing endonuclease subunit SEN54	225.74	225.97	239.97	317.08	288.13	317.22	0.42
KLMA_10777	BRO1	vacuolar protein-sorting protein BRO1	513.79	629.80	582.26	767.05	666.40	572.20	0.22
KLMA_10778	MBA1	protein MBA1	591.39	535.83	599.32	256.52	432.19	386.37	-0.68
KLMA_10779	SEC16	hypothetical protein	1185.13	1531.43	1321.67	915.91	856.80	946.47	-0.57
KLMA_10780	ELP3	elongator complex protein 3	1183.96	1107.46	1197.42	693.87	756.54	707.04	-0.69
KLMA_10781	GLY1	low specificity L-threonine aldolase	90.53	146.54	121.81	383.52	366.48	342.28	1.60
KLMA_10782	YDC1	alkaline ceramidase YDC1	839.47	553.73	705.30	5433.24	2112.09	1959.49	2.18
KLMA_10783	SOU1	sorbose reductase SOU1	192.82	345.66	177.85	2973.98	3926.79	4215.45	3.95
KLMA_10784	SMP1	MADS_MEF2_like	726.60	1288.69	767.42	905.82	544.24	685.43	-0.38
KLMA_10786	RPS6	40S ribosomal protein S5	29506.08	24586.82	27381.04	10711.72	21354.27	16962.94	-0.73
KLMA_10787	GLR1	glutathione reductase	951.16	1045.94	917.25	806.58	1237.60	1319.87	0.21
KLMA_10788	SSU1	sulfite efflux pump SSU1	1240.39	960.92	1045.15	1805.75	1706.86	1728.71	0.69
KLMA_10789	DTR1	uncharacterized transporter YBR180W	114.05	291.97	177.85	487.81	310.03	350.06	0.97
KLMA_10791	NOG1	nucleolar GTP-binding protein 1	3450.76	3156.83	3550.84	1396.16	1406.94	1490.15	-1.24
KLMA_10793	SEC62	translocation protein SEC62	546.71	511.22	551.81	516.41	572.88	594.68	0.07
KLMA_10794	FZO1	transmembrane GTPase FZO1	972.33	978.82	952.57	496.22	500.43	566.15	-0.89
KLMA_10795	EHT1	medium-chain fatty acid ethyl ester synthase/esterase 2	1028.76	1156.68	1001.30	946.19	1070.79	1083.90	-0.04
KLMA_10796	PNG1	peptide-N(4)-(N-acetyl-beta-glucosaminyl)asparagine amidase	121.10	198.00	144.96	361.66	324.35	347.47	1.15
KLMA_10797	MSY1	tyrosyl-tRNA synthetase	485.58	547.02	440.96	423.89	577.94	545.41	0.07

Locus_tag	UniProt_gene	Product	Unique exon reads						log$_2$ Fold Change
			KmWT.1	KmWT.2	KmWT.3	Kmmig1.1	Kmmig1.2	Kmmig1.3	
KLMA_10798	MGR2	protein MGR2	195.17	229.32	267.99	188.40	239.26	235.97	-0.06
KLMA_10799	AIM43	mitochondrial protein FMP14	190.47	210.31	169.32	254.84	288.97	241.15	0.46
KLMA_10800	ATG21	autophagy-related protein 21	607.85	550.38	640.73	462.58	380.80	392.42	-0.54
KLMA_10802	ELP4	elongator complex protein 4	236.32	268.48	264.33	346.52	249.37	294.74	0.21
KLMA_10803	FMP30	N-acyl-phosphatidylethanolamine-hydrolyzing phospholipase D	162.25	192.41	116.94	325.49	192.08	270.54	0.74
KLMA_10804	ECM31	3-methyl-2-oxobutanoate hydroxymethyltransferase	117.57	120.81	143.74	312.03	216.52	244.61	1.02
KLMA_10805		uncharacterized abhydrolase domain-containing protein YGR015C	27.04	70.47	30.45	1099.26	646.18	739.02	4.27
KLMA_10806		hypothetical protein	12.93	13.42	10.96	63.08	32.86	38.90	1.85
KLMA_10807	MSD1	aspS_bact	616.08	629.80	610.28	497.07	502.96	499.60	-0.31
KLMA_10808	SWD3	COMPASS component SWD3	191.64	210.31	191.25	330.54	308.35	292.15	0.65
KLMA_10809	UMP1	proteasome maturation factor UMP1	312.74	347.90	272.86	617.34	539.19	590.35	0.90
KLMA_10810	SYH1	uncharacterized protein YPL105C	1166.32	1033.63	1205.95	779.66	885.44	917.08	-0.40
KLMA_10811	SEC66	translocation protein SEC66	684.27	512.34	551.81	462.58	549.30	525.53	-0.19
KLMA_10812	NPL4	nuclear protein localization protein 4	719.55	776.34	655.35	919.28	879.55	903.25	0.33
KLMA_10813	SSE1	heat shock protein homolog SSE1	3702.37	3823.55	3656.82	2328.05	3116.32	2868.79	-0.43
KLMA_10814		oxidored-like super family	151.67	154.37	133.99	419.69	295.71	274.00	1.17
KLMA_10815	PEX32	peroxisomal membrane protein PEX32	306.87	230.44	267.99	338.11	311.72	281.78	0.21
KLMA_10816	POP7	ribonucleases P/MRP protein subunit POP7	95.23	80.54	92.58	89.15	106.99	77.79	0.03
KLMA_10817		hypothetical protein	148.14	128.64	119.38	209.42	143.22	152.99	0.35

Locus_tag	UniProt_gene	Product	Unique exon reads						log2 Fold Change
			KmWT.1	KmWT.2	KmWT.3	Kmmig1.1	Kmmig1.2	Kmmig1.3	
KLMA_10818	DAL3	ureidoglycolate hydrolase	55.26	55.93	46.29	230.45	167.65	139.16	1.77
KLMA_10819	TYR1	prephenate dehydrogenase [NADP+]	375.06	365.80	341.08	442.40	433.03	488.36	0.33
KLMA_10820		hypothetical protein	345.66	318.82	321.59	353.24	334.46	338.83	0.06
KLMA_10821	ARL1	ADP-ribosylation factor-like protein 1	560.82	497.80	499.43	629.11	786.03	628.39	0.39
KLMA_10822		ABC1 family protein YPL109C	353.89	356.85	345.95	485.29	453.25	399.33	0.34
KLMA_10824	str3	Sit1p	542.01	549.26	533.54	588.74	341.20	274.86	-0.43
KLMA_10826		hypothetical protein	195.17	146.54	115.72	362.50	281.39	243.75	0.96
KLMA_10827		MFS_1	1302.71	626.44	1099.97	1072.35	663.03	647.40	-0.35
KLMA_10828		hypothetical protein	47.03	31.32	26.80	42.89	61.50	34.57	0.40
KLMA_10829		hypothetical protein	152.84	155.49	146.18	270.82	323.51	240.29	0.88
KLMA_10830	Nqo2	ribosyldihydronicotinamide dehydrogenase [quinone]	951.16	727.12	707.73	2278.43	2169.38	1761.55	1.38
KLMA_10831	YCT1	uncharacterized transporter YLL055W	453.83	399.36	458.02	427.26	427.14	396.74	-0.07
KLMA_10832	URA1	dihydroorotate dehydrogenase	5823.38	4224.02	5610.69	677.89	691.67	670.74	-2.94
KLMA_10833		uncharacterized protein C800.11	3236.78	2050.49	3001.46	3010.99	4906.59	4498.96	0.58
KLMA_10834	PHO12	repressible acid phosphatase	279.82	219.26	289.91	601.36	331.94	403.65	0.76
KLMA_10835		flocculation protein FLO5	438.55	398.24	417.82	508.00	358.05	472.80	0.09
KLMA_10836	FCY2	purine-cytosine permease FCY2	8.23	4.47	9.75	272.50	73.30	116.69	4.37
KLMA_20002		flocculation protein FLO5	1058.16	523.53	1159.66	1238.04	952.84	970.67	0.21
KLMA_20003		conserved hypothetical membrane protein	4.70	10.07	6.09	41.21	27.80	25.93	2.18
KLMA_20004	OPT1	oligopeptide transporter 1	335.08	186.81	310.62	635.00	406.92	508.24	0.90
KLMA_20005	ADH4	alcohol dehydrogenase 4	451.48	82.78	315.49	434.83	187.03	160.77	-0.12

Locus_tag	UniProt_gene	Product	Unique exon reads						log_2 Fold Change
			KmWT.1	KmWT.2	KmWT.3	Kmmig1.1	Kmmig1.2	Kmmig1.3	
KLMA_20006	DUR3	probable urea active transporter 1	610.20	206.95	518.92	721.63	387.54	418.35	0.19
KLMA_20007	yxeK	putative monooxygenase yxeK	83.48	83.90	108.41	258.21	152.49	158.18	1.05
KLMA_20008	FRE4	ferric reductase transmembrane component 4	375.06	157.73	317.93	1180.85	626.80	401.92	1.38
KLMA_20009	ADY2	hypothetical protein	37.62	67.12	65.78	848.63	2427.18	2395.12	5.06
KLMA_20010		protein crtK	29.39	31.32	26.80	121.95	94.36	70.88	1.71
KLMA_20011	MNT4	probable alpha-1	272.77	241.63	221.70	402.87	419.55	489.22	0.83
KLMA_20012	yciC	ras_like_GTPase super family	152.84	88.37	198.55	1685.48	674.82	331.05	2.61
KLMA_20013		PX_Bem1p	527.90	401.60	548.16	574.44	563.62	592.08	0.23
KLMA_20014		hypothetical protein	136.38	175.63	136.43	368.38	423.77	375.13	1.38
KLMA_20015	YUH1	ubiquitin carboxyl-terminal hydrolase YUH1	115.22	138.71	91.36	286.80	353.00	372.54	1.55
KLMA_20016	THP3	PCI super family	643.12	504.51	655.35	510.52	435.56	478.85	-0.34
KLMA_20017	uch2	ubiquitin carboxyl-terminal hydrolase isozyme L5	292.76	342.31	269.21	660.23	537.50	509.10	0.92
KLMA_20018	AIM25	altered inheritance rate of mitochondria protein 25	261.01	274.07	235.10	650.98	527.39	459.84	1.09
KLMA_20019	PPH3	serine/threonine-protein phosphatase 4 catalytic subunit	892.38	783.06	845.38	563.51	570.36	518.61	-0.61
KLMA_20020		protein PEA2	357.42	281.90	339.86	952.08	547.61	556.64	1.07
KLMA_20021	SPT15	TATA-box-binding protein	566.70	577.22	498.21	964.69	753.17	737.29	0.58
KLMA_20022	SCC4	sister chromatid cohesion protein 4	677.22	582.82	651.70	416.32	313.40	349.20	-0.83
KLMA_20023	TPS2	trehalose-phosphatase	10559.22	11720.10	10629.37	6119.54	4506.41	5033.13	-1.07
KLMA_20024	IPT1	inositolphosphotransferase 1	1602.52	2030.35	1532.40	1383.54	1100.28	1433.10	-0.40
KLMA_20025	LSM5	U6 snRNA-associated Sm-like protein LSm5	130.51	151.02	118.16	112.70	130.58	104.59	-0.20

Locus_tag	UniProt_gene	Product	Unique exon reads						log2 Fold Change
			KmWT.1	KmWT.2	KmWT.3	Kmmig1.1	Kmmig1.2	Kmmig1.3	
KLMA_20026	PAA1	polyamine N-acetyltransferase 1	584.34	670.07	577.39	874.70	882.07	823.73	0.49
KLMA_20027		hypothetical protein	4.70	13.42	0.00	258.21	124.69	159.04	4.89
KLMA_20028	DOA4	ubiquitin carboxyl-terminal hydrolase 4	476.17	514.58	484.81	746.86	577.94	635.30	0.41
KLMA_20029	DOS2	protein DOS2	391.52	352.37	347.17	460.06	550.98	413.16	0.38
KLMA_20030	OCA6	putative tyrosine-protein phosphatase OCA6	178.71	171.15	158.36	154.75	193.77	150.40	-0.03
KLMA_20031	DDI1	DNA damage-inducible protein 1	217.51	316.58	244.84	390.25	404.39	405.38	0.62
KLMA_20032	MAG1	DNA-3-methyladenine glycosylase	75.25	102.92	69.43	259.89	241.79	144.35	1.38
KLMA_20033	COX15	cytochrome c oxidase assembly protein COX15	3909.30	2330.15	3766.45	1662.77	2206.45	1969.86	-0.78
KLMA_20035	PIM1	lon protease homolog	1105.18	1403.91	1081.70	683.78	639.44	669.87	-0.85
KLMA_20036	HAP3	transcriptional activator HAP3	61.14	85.02	65.78	88.31	44.65	71.74	-0.05
KLMA_20037	RFT1	oligosaccharide translocation protein RFT1	513.79	437.39	482.38	1012.63	1189.58	949.92	1.14
KLMA_20038	APN2	DNA-(apurinic or apyrimidinic site) lyase 2	232.79	276.31	236.32	244.75	356.37	317.22	0.30
KLMA_20039	POP8	ribonucleases P/MRP protein subunit POP8	32.92	42.51	46.29	43.74	53.92	40.62	0.18
KLMA_20040	VPS10	vacuolar protein sorting/targeting protein PEP1	1047.57	1402.79	1203.51	2867.17	1918.32	1913.68	0.87
KLMA_20041	FUS3	mitogen-activated protein kinase FUS3	8.23	8.95	19.49	365.86	170.18	187.56	4.31
KLMA_20042	ACH1	acetyl-CoA hydrolase	3917.53	3577.44	3470.44	4981.59	5792.03	5433.33	0.56
KLMA_20043		methionyl-tRNA formyltransferase	67.02	67.12	71.87	42.05	58.13	61.37	-0.35
KLMA_20044		hypothetical protein	34.10	49.22	48.73	89.15	58.13	81.25	0.79
KLMA_20045	PGD1	mediator of RNA polymerase II transcription subunit	270.42	256.17	252.15	151.39	197.98	244.61	-0.39

Locus_tag	UniProt_gene	Product	Unique exon reads						log$_2$ Fold Change
			KmWT.1	KmWT.2	KmWT.3	Kmmig1.1	Kmmig1.2	Kmmig1.3	
		3							
KLMA_20046	TRP5	tryptophan synthase	5157.92	2732.86	4782.36	2987.44	4195.54	4791.98	-0.08
KLMA_20047	RRN6	RNA polymerase I-specific transcription initiation factor RRN6	181.06	175.63	203.43	226.24	133.95	163.36	-0.10
KLMA_20048	CWH41	mannosyl-oligosaccharide glucosidase	319.80	382.58	378.84	517.25	507.17	565.29	0.56
KLMA_20049	SCW11	probable family 17 glucosidase SCW11	2936.97	2521.44	2671.35	1508.02	2003.41	2045.06	-0.55
KLMA_20050	MIOX5	inositol oxygenase 1	59.96	91.73	75.52	158.12	152.49	171.14	1.08
KLMA_20051	CGR1	rRNA-processing protein CGR1	142.26	127.53	174.19	102.61	115.42	133.11	-0.34
KLMA_20052		uncharacterized membrane protein YGR149W	1104.01	426.21	784.47	269.14	304.13	343.15	-1.34
KLMA_20053	RPL30	60S ribosomal protein L30	11185.88	8967.10	10725.60	4200.25	6960.55	5368.50	-0.90
KLMA_20054	RPL24	60S ribosomal protein L24	10861.38	10538.81	11162.91	3577.02	6321.95	5269.96	-1.10
KLMA_20055		hypothetical protein	0.00	0.00	8.53	9.25	1.68	4.32	0.86
KLMA_20056	NAT2	DUF1279 super family	186.94	237.15	186.37	154.75	208.09	217.82	-0.07
KLMA_20057		uricase	23.51	24.61	48.73	66.44	80.04	73.47	1.19
KLMA_20058	HOP2	hypothetical protein	9.41	3.36	8.53	11.77	8.42	6.91	0.36
KLMA_20059	MIG1	regulatory protein MIG1	678.40	768.51	734.53	926.01	482.74	574.80	-0.14
KLMA_20060	PRP24	U4/U6 snRNA-associated-splicing factor PRP24	179.89	189.05	191.25	158.96	152.49	183.24	-0.18
KLMA_20061	NUP188	nucleoporin NUP188	1486.12	1418.45	1495.86	841.90	1315.95	1166.88	-0.40
KLMA_20062	PPA2	inorganic pyrophosphatase	126.98	228.20	169.32	131.21	138.17	133.97	-0.38
KLMA_20063	RSN1	uncharacterized protein RSN1	12256.97	9042.05	12312.82	7745.31	5971.48	5731.53	-0.79
KLMA_20064		hypothetical conserved protein	28.22	36.92	25.58	137.09	90.99	92.49	1.82
KLMA_20065		DUF2410 super family	37.62	38.03	46.29	85.79	68.24	74.33	0.91

Locus_tag	UniProt_gene	Product	Unique exon reads						log$_2$ Fold Change
			KmWT.1	KmWT.2	KmWT.3	Kmmig1.1	Kmmig1.2	Kmmig1.3	
KLMA_20067	CAC2	chromatin assembly factor 1 subunit p60	272.77	112.98	191.25	319.60	336.15	481.44	0.98
KLMA_20068	CUE1	coupling of ubiquitin conjugation to ER degradation protein 1	451.48	350.14	386.15	487.81	403.55	487.50	0.22
KLMA_20069	SAP30	transcriptional regulatory protein SAP30	146.97	190.17	162.01	868.81	374.06	467.62	1.78
KLMA_20070		metallo-dependent_hydrolases	30.57	35.80	36.54	201.01	128.06	221.27	2.42
KLMA_20071		DUP super family	886.50	705.87	760.11	1788.93	770.87	849.66	0.54
KLMA_20072		hypothetical conserved protein	185.77	218.14	208.30	762.00	384.17	440.82	1.37
KLMA_20073		hypothetical conserved protein	293.93	344.54	271.64	489.50	335.31	393.28	0.42
KLMA_20074	TSL1	trehalose synthase complex regulatory subunit TPS3	5322.52	4988.06	5246.47	2021.91	1541.73	1920.59	-1.50
KLMA_20075	TIF11	eukaryotic translation initiation factor 1A	1071.09	1118.65	1069.51	707.33	1146.61	971.53	-0.21
KLMA_20076	NIPA2	magnesium transporter NIPA2	97.59	89.49	77.96	343.15	266.22	295.61	1.77
KLMA_20077	SIF2	SIR4-interacting protein SIF2	253.96	334.48	196.12	184.19	204.72	298.20	-0.19
KLMA_20078	EXO84	exocyst complex component EXO84	318.62	462.00	347.17	313.71	331.94	366.49	-0.16
KLMA_20079	FES1	hsp70 nucleotide exchange factor FES1	315.10	221.49	236.32	231.29	357.21	277.46	0.16
KLMA_20080	EME1	crossover junction endonuclease EME1	129.33	147.66	138.87	347.36	208.93	234.24	0.93
KLMA_20081	SEC8	exocyst complex component SEC8	676.04	698.04	722.35	460.06	615.01	552.32	-0.37
KLMA_20082		hypothetical protein	249.25	287.49	247.28	348.20	361.42	356.98	0.44
KLMA_20083	DAP2	dipeptidyl aminopeptidase B	601.97	1016.85	553.03	1047.96	1002.55	1072.66	0.52
KLMA_20084	RPN1	26S proteasome regulatory subunit RPN1	2354.98	2962.19	2343.67	1534.93	1727.92	1829.84	-0.59
KLMA_20085	VMA16	v-type proton ATPase subunit c"	1122.82	948.62	955.01	1540.82	1424.63	1392.47	0.53

Locus_tag	UniProt_gene	Product	Unique exon reads						$\log_2$ Fold Change
			KmWT.1	KmWT.2	KmWT.3	Kmmig1.1	Kmmig1.2	Kmmig1.3	
KLMA_20086	THR1	homoserine kinase	1126.35	765.16	951.36	732.56	888.81	1004.38	-0.11
KLMA_20087	MAS2	mitochondrial-processing peptidase subunit alpha	413.86	503.39	465.32	301.10	385.01	380.32	-0.37
KLMA_20088	MYO1	myosin-1	755.99	854.65	763.77	624.91	770.02	707.04	-0.18
KLMA_20089		conserved hypothetical protein	198.70	200.24	183.94	230.45	206.41	169.41	0.06
KLMA_20090		ras-related protein rapC	332.73	296.44	356.91	169.05	133.95	166.82	-1.07
KLMA_20091		ribosomal protein S27	8856.76	6549.70	9092.09	4325.56	6626.93	5135.12	-0.61
KLMA_20092	RSM22	37S ribosomal protein S22	329.20	441.87	364.22	220.36	235.05	245.48	-0.70
KLMA_20093	SRP102	signal recognition particle receptor subunit beta	810.08	800.95	812.49	329.69	515.60	497.00	-0.85
KLMA_20094	NUP192	nucleoporin NUP192	2009.32	2095.23	2013.56	1155.61	1582.17	1576.58	-0.50
KLMA_20095	NSP1	nucleoporin NSP1	1675.41	1905.06	1772.37	811.62	1137.34	1021.67	-0.85
KLMA_20096	MHP1	MAP-homologous protein 1	726.60	1010.14	830.76	1464.28	1293.20	1486.69	0.72
KLMA_20097		hypothetical protein	23.51	60.41	36.54	45.42	37.07	32.85	-0.07
KLMA_20098	GPM1	phosphoglycerate mutase 1	67502.10	51898.67	67722.95	4346.59	7060.80	6282.99	-3.40
KLMA_20099	ADY2	hypothetical protein	27.04	25.73	21.93	32.80	18.53	32.85	0.17
KLMA_20100	RVS161	reduced viability upon starvation protein 161	1245.10	1315.53	1158.44	2005.92	1578.80	1592.14	0.48
KLMA_20101		sterol-sensing super family	926.47	936.31	959.88	3794.86	1684.95	1626.71	1.33
KLMA_20102	SAT4	serine/threonine-protein kinase HAL4/SAT4	1661.30	1174.58	1320.45	1126.18	938.52	923.13	-0.48
KLMA_20103		hypothetical protein	19.99	13.42	24.36	26.07	18.53	17.29	0.10
KLMA_20104		hypothetical protein	1.18	1.12	4.87	5.05	4.21	4.32	0.94
KLMA_20105	CIT1	citrate synthase	7468.23	8963.75	7055.39	16453.63	18391.28	15134.83	1.09
KLMA_20106	YCP4	flavoprotein-like protein YCP4	3217.97	3126.63	3217.07	4402.10	4368.24	4310.53	0.45
KLMA_20107		jmjC super family	931.18	918.41	989.12	445.76	343.73	387.23	-1.27

Locus_tag	UniProt_gene	Product	Unique exon reads						log2 Fold Change
			KmWT.1	KmWT.2	KmWT.3	Kmmig1.1	Kmmig1.2	Kmmig1.3	
KLMA_20108	MRPL32	ribosomal_L32p super family	649.00	580.58	615.15	306.99	524.02	439.96	-0.54
KLMA_20109	CDC10	cell division control protein 10	1093.43	1134.31	1040.28	806.58	981.49	968.08	-0.25
KLMA_20110	DNM1	dynamin-related protein DNM1	1179.26	1333.43	1159.66	935.26	1107.02	897.20	-0.32
KLMA_20111	RTT109	histone acetyltransferase RTT109	137.56	121.93	126.69	351.56	276.33	263.63	1.21
KLMA_20112	PRE3	proteasome component PRE3	674.87	708.11	528.67	541.64	741.38	644.81	0.01
KLMA_20113	SFI1	protein SFI1	324.50	370.27	306.97	426.42	394.28	388.09	0.27
KLMA_20114	OST1	dolichyl-diphosphooligosaccharide--protein glycosyltransferase subunit 1	727.78	811.02	802.75	592.11	862.70	913.62	0.02
KLMA_20115	ORC3	origin recognition complex subunit 3	293.93	307.63	339.86	343.15	238.42	316.35	-0.07
KLMA_20116	COX16	cytochrome c oxidase assembly protein COX16	765.40	687.97	743.06	728.36	480.21	541.09	-0.33
KLMA_20117	ADR1	regulatory protein ADR1	139.91	134.24	129.12	2354.12	1534.99	1538.55	3.75
KLMA_20118	RAD9	rad9_Rad53_bind super family	312.74	267.36	378.84	285.96	288.97	309.44	-0.12
KLMA_20119		hypothetical protein	126.98	130.88	147.39	200.17	197.98	149.53	0.44
KLMA_20120	SPR28	CDC_Septin	81.13	58.17	68.22	97.56	83.41	96.81	0.42
KLMA_20121	BUR2	protein BUR2	486.75	475.43	448.27	330.54	236.74	248.93	-0.79
KLMA_20122		hypothetical protein	295.11	346.78	332.55	555.10	379.11	413.16	0.47
KLMA_20123	GTB1	glucosidase 2 subunit beta	239.85	300.92	229.01	373.43	353.00	404.52	0.55
KLMA_20124		SVF1-like protein YDR222W	55.26	32.44	37.76	104.29	38.75	60.50	0.70
KLMA_20125	IFH1	protein IFH1	954.69	1102.99	1121.89	553.42	497.90	597.27	-0.95
KLMA_20126	UTP13	U3 small nucleolar RNA-associated protein 13	1278.02	950.85	1191.33	510.52	438.93	566.15	-1.17
KLMA_20128	RSA3	ribosome assembly protein 3	155.20	155.49	136.43	113.54	89.30	99.40	-0.57
KLMA_20129	CCC1	protein CCC1	921.77	644.34	706.51	764.52	690.83	759.77	-0.04

Locus_tag	UniProt_gene	Product	Unique exon reads						log$_2$ Fold Change
			KmWT.1	KmWT.2	KmWT.3	Kmmig1.1	Kmmig1.2	Kmmig1.3	
KLMA_20130	ECM4	glutathione S-transferase omega-like 2	337.43	277.43	275.30	6563.62	3117.17	3947.50	3.94
KLMA_20131		Golgi apparatus membrane protein TVP15	841.82	806.55	794.22	4293.60	2160.95	2221.39	1.83
KLMA_20132	BMH2	protein BMH2	12202.89	11478.47	11482.06	17328.33	11559.63	11211.53	0.19
KLMA_20133	IRC20	hypothetical protein	276.30	259.53	233.88	412.96	347.94	334.50	0.51
KLMA_20135	RCK2	serine/threonine-protein kinase srk1	1134.58	1221.57	1125.55	1160.66	1202.21	1275.79	0.06
KLMA_20136	MEF2	ribosome-releasing factor 2	181.06	251.70	202.21	138.77	137.32	153.85	-0.56
KLMA_20137	GCN3	translation initiation factor eIF-2B subunit alpha	498.51	485.49	553.03	258.21	347.10	328.45	-0.72
KLMA_20138	RPC37	DNA-directed RNA polymerase III subunit rpc5	504.39	451.93	492.12	232.13	191.24	256.71	-1.09
KLMA_20139	DBP7	ATP-dependent RNA helicase DBP7	716.02	723.77	853.91	301.94	293.18	325.00	-1.32
KLMA_20140	GSM1	glucose starvation modulator protein 1	64.67	151.02	80.40	319.60	310.87	302.52	1.65
KLMA_20141		uncharacterized protein YKR023W	174.01	154.37	172.97	412.12	214.83	277.46	0.85
KLMA_20143	PAM16	mitochondrial import inner membrane translocase subunit TIM16	332.73	308.75	304.53	358.29	233.37	304.25	-0.08
KLMA_20144		hap4 transcription factor	479.70	436.27	444.62	677.89	536.66	495.27	0.33
KLMA_20145	CTF13	centromere DNA-binding protein complex CBF3 subunit C	48.20	98.44	57.25	76.54	77.51	98.54	0.31
KLMA_20146	SLD2	DNA replication regulator SLD2	45.85	35.80	77.96	85.79	63.19	70.88	0.47
KLMA_20147	UTP15	U3 small nucleolar RNA-associated protein 15	1154.57	1023.57	1409.37	438.19	518.12	520.34	-1.28
KLMA_20148	AIP1	actin-interacting protein 1	1315.64	1234.99	1232.74	733.40	1150.82	1244.67	-0.27
KLMA_20149	NPL6	chromatin structure-remodeling complex subunit RSC7	211.63	237.15	199.77	339.79	249.37	318.08	0.48

Locus_tag	UniProt_gene	Product	Unique exon reads						$\log_2$ Fold Change
			KmWT.1	KmWT.2	KmWT.3	Kmmig1.1	Kmmig1.2	Kmmig1.3	
KLMA_20150	YTA12	mitochondrial respiratory chain complexes assembly protein RCA1	812.43	1135.43	825.89	638.36	562.77	649.99	-0.58
KLMA_20151	VBA1	vacuolar basic amino acid transporter 1	946.46	558.21	640.73	689.67	600.69	769.27	-0.06
KLMA_20152		probable ADP-ribose 1"-phosphate phosphatase YML087W	342.14	192.41	272.86	164.85	215.67	158.18	-0.58
KLMA_20153	AAT1	aspartate aminotransferase	281.00	261.76	310.62	233.81	205.56	228.19	-0.35
KLMA_20154		uncharacterized protein YMR086W	518.50	760.68	546.94	527.34	663.03	679.38	0.03
KLMA_20155	GFA1	glucosamine--fructose-6-phosphate aminotransferase [isomerizing]	1896.45	2046.01	1741.92	1425.59	1856.82	2237.81	-0.04
KLMA_20156	LAP4	vacuolar aminopeptidase 1	763.05	893.80	713.82	958.81	1123.86	1350.12	0.53
KLMA_20157	HSL1	probable serine/threonine-protein kinase HSL1	1489.65	1614.21	1592.09	643.41	831.53	871.27	-1.00
KLMA_20158	ADH4	alcohol dehydrogenase 4	5548.26	5312.47	5046.70	1347.38	369.01	362.16	-2.94
KLMA_20159	UTP11	U3 small nucleolar RNA-associated protein 11	309.22	252.82	244.84	129.52	141.54	111.50	-1.08
KLMA_20160	MTC2	hypothetical protein	514.97	467.60	567.65	427.26	310.87	325.86	-0.54
KLMA_20161	GGC1	putative mitochondrial carrier protein YHM1/SHM1	3851.69	2646.73	3633.67	2033.68	2919.18	2351.04	-0.47
KLMA_20162	PCF11	protein PCF11	213.98	260.65	242.41	275.03	315.09	354.38	0.40
KLMA_20163		hypothetical protein	417.38	381.46	328.89	336.42	276.33	274.00	-0.35
KLMA_20164		hypothetical protein	54.08	86.14	51.16	210.26	140.69	182.38	1.48
KLMA_20165	ADK1	adenylate kinase 1	3854.04	3483.48	3656.82	1141.32	1814.70	1484.10	-1.31
KLMA_20166	MSC3	hypothetical protein	94.06	127.53	69.43	136.25	130.58	197.94	0.67
KLMA_20168	HTA1	histone H2A	4176.19	4954.50	4465.65	3919.33	4289.05	3823.04	-0.18
KLMA_20169	HTB1	histone H2B.1	1894.10	1694.76	2128.07	1047.12	1372.40	1418.40	-0.57

Locus_tag	UniProt_gene	Product	Unique exon reads						log2 Fold Change
			KmWT.1	KmWT.2	KmWT.3	Kmmig1.1	Kmmig1.2	Kmmig1.3	
KLMA_20170	SPO71	sporulation-specific protein 71	352.72	450.82	372.75	303.62	208.09	218.68	-0.69
KLMA_20171	STE5	protein STE5	271.59	250.58	226.57	349.04	249.37	375.13	0.38
KLMA_20172	PDA1	pyruvate dehydrogenase E1 component subunit alpha	3843.46	3815.72	3368.12	3490.39	3855.18	3396.91	-0.04
KLMA_20173	ARX1	probable metalloprotease ARX1	1332.10	1204.79	1309.49	310.35	350.47	426.13	-1.82
KLMA_20174	DMC1	meiotic recombination protein DMC1	57.61	31.32	56.03	102.61	83.41	121.01	1.09
KLMA_20175	TMS1	membrane protein TMS1	632.54	582.82	595.66	965.54	834.89	782.24	0.51
KLMA_20176		actin superfamily	55.26	55.93	70.65	121.95	141.54	119.28	1.08
KLMA_20177		hypothetical protein	279.82	299.80	296.00	246.43	283.91	295.61	-0.08
KLMA_20178		hypothetical protein	32.92	13.42	7.31	2.52	5.05	12.97	-1.39
KLMA_20179	RDH54	DNA repair and recombination protein RDH54	117.57	222.61	193.68	292.69	283.07	284.37	0.69
KLMA_20180	VMS1	zinc finger protein YDR049W	345.66	331.12	265.55	275.03	325.20	311.17	-0.05
KLMA_20181	HEM12	uroporphyrinogen decarboxylase	1580.18	1418.45	1338.72	1226.26	1598.18	1427.05	-0.03
KLMA_20182		uncharacterized zinc metalloprotease YBR074W	1329.75	1349.09	1231.53	1226.26	1520.67	1433.96	0.10
KLMA_20183		conserved hypothetical protein	1427.33	1210.38	1274.16	1628.29	1419.57	1502.25	0.22
KLMA_20184	DSE4	endo-1,3-beta-glucanase	1280.37	777.46	1020.79	608.09	1281.41	979.31	-0.10
KLMA_20186		conserved hypothetical protein	1369.72	1061.60	1332.63	734.24	319.30	262.76	-1.52
KLMA_20187	DBF4	protein DBF4	551.42	550.38	503.09	244.75	313.40	297.34	-0.91
KLMA_20188	SLM4	protein SLM4	219.86	180.10	181.50	132.05	150.80	110.64	-0.56
KLMA_20189	RGP1	reduced growth phenotype protein 1	202.23	323.29	261.90	354.93	386.70	401.06	0.54
KLMA_20190	AVL9	late secretory pathway protein avl9	683.10	695.80	715.04	1116.08	720.32	725.19	0.29
KLMA_20191	HPR1	THO complex	551.42	635.39	562.77	557.62	603.21	552.32	-0.03

Locus_tag	UniProt_gene	Product	Unique exon reads						log$_2$ Fold Change
			KmWT.1	KmWT.2	KmWT.3	Kmmig1.1	Kmmig1.2	Kmmig1.3	
		subunit HPR1							
KLMA_20192	NOP53	ribosome biogenesis protein NOP53	1071.09	730.48	1064.64	402.87	530.76	497.87	-1.00
KLMA_20193	PXA1	peroxisomal long-chain fatty acid import protein 2	35.27	26.85	36.54	458.38	235.05	298.20	3.33
KLMA_20194		UPF0613 protein PB24D3.06c	205.75	334.48	191.25	1058.05	615.85	738.16	1.72
KLMA_20195	ppr1	acetyltransferases	118.75	123.05	121.81	393.62	338.68	354.38	1.58
KLMA_20196	ABP140	hypothetical protein	425.61	383.70	433.65	446.60	621.75	560.10	0.39
KLMA_20199	ATG5	autophagy protein 5	99.94	85.02	116.94	137.93	115.42	112.37	0.28
KLMA_20200		SGT1 super family	42.33	99.56	74.31	191.76	131.43	138.30	1.09
KLMA_20201		putative serine/threonine-protein kinase YPL150W	938.23	1009.02	925.78	948.71	732.11	808.17	-0.21
KLMA_20202	MET7	folylpolyglutamate synthase	591.39	454.17	719.91	386.89	422.92	456.38	-0.48
KLMA_20203	SSP2	sporulation-specific protein 2	2.35	0.00	2.44	236.34	72.45	71.74	6.34
KLMA_20204	PUS7	multisubstrate pseudouridine synthase 7	1258.03	1127.60	1208.38	698.08	662.19	653.45	-0.84
KLMA_20205	PRP46	pre-mRNA-splicing factor PRP46	242.20	365.80	328.89	296.89	283.07	303.39	-0.09
KLMA_20206	ESA1	histone acetyltransferase ESA1	310.39	270.71	297.22	337.26	306.66	322.40	0.14
KLMA_20207	RRD2	serine/threonine-protein phosphatase 2A activator 2	391.52	363.56	395.89	354.09	357.21	311.17	-0.17
KLMA_20208	DGA1	diacylglycerol O-acyltransferase 1	1061.68	906.11	1073.17	825.92	762.44	834.97	-0.33
KLMA_20209	RAD53	serine/threonine-protein kinase RAD53	513.79	442.99	487.25	301.94	278.86	299.07	-0.71
KLMA_20210	SLD5	DNA replication complex GINS protein SLD5	76.42	68.24	70.65	91.68	109.52	129.65	0.62
KLMA_20211	PAC11	WD repeat-containing protein PAC11	62.31	82.78	87.71	112.70	139.85	102.86	0.61

Locus_tag	UniProt_gene	Product	Unique exon reads						log2 Fold Change
			KmWT.1	KmWT.2	KmWT.3	Kmmig1.1	Kmmig1.2	Kmmig1.3	
KLMA_20212	HST2	NAD-dependent deacetylase HST2	49.38	87.25	43.85	127.00	112.05	124.47	1.01
KLMA_20213	MSH4	mutS protein homolog 4	256.31	354.61	258.24	511.36	404.39	414.89	0.61
KLMA_20214	ULP1	ULP1	272.77	325.53	333.77	354.09	372.37	334.50	0.19
KLMA_20215	BAS1	myb-like DNA-binding protein BAS1	496.16	476.55	570.08	398.66	222.41	274.00	-0.79
KLMA_20216		DUF1748 super family	48.20	44.75	58.47	90.83	97.73	97.67	0.92
KLMA_20217	COA1	cytochrome oxidase assembly protein 1	395.04	425.09	400.76	232.13	374.06	376.86	-0.31
KLMA_20218	SKG1	suppressor of lethality of KEX2 GAS1 double null mutant protein 1	667.81	627.56	584.70	487.81	470.10	592.95	-0.28
KLMA_20219	BNR1	BNI1-related protein 1	126.98	154.37	154.70	174.94	165.13	199.67	0.31
KLMA_20220	POT1	3-ketoacyl-CoA thiolase	32.92	44.75	36.54	172.42	263.70	368.21	2.81
KLMA_20221	THI72	thiamine transporter	121.10	93.97	103.54	259.05	365.64	369.08	1.64
KLMA_20222		GAL4	312.74	284.14	276.51	397.82	261.17	327.59	0.18
KLMA_20223	DSD1	D-serine dehydratase	279.82	325.53	320.37	757.79	632.70	634.44	1.13
KLMA_20224	CDC20	APC/C activator protein CDC20	78.77	106.27	112.07	160.64	187.03	180.65	0.83
KLMA_20225	SNF4	nuclear protein SNF4	922.95	965.40	877.05	1023.57	1053.10	1103.78	0.20
KLMA_20226		putative oligopeptide transporter YGL114W	1008.78	690.21	778.38	996.65	1179.47	1217.87	0.45
KLMA_20227	SWC5	SWR1-complex protein 5	149.32	130.88	123.03	171.58	164.28	140.89	0.24
KLMA_20228	PBP2	PAB1-binding protein 2	235.15	262.88	237.53	303.62	353.00	334.50	0.43
KLMA_20229	SLD3	DNA replication regulator SLD3	230.44	213.66	207.08	381.00	238.42	302.52	0.50
KLMA_20230	TAF6	transcription initiation factor TFIID subunit 6	482.05	590.65	534.76	479.40	582.99	539.36	-0.01
KLMA_20231	DAD3	DASH complex subunit DAD3	11.76	25.73	9.75	23.55	14.32	26.79	0.45
KLMA_20232	ARC40	actin-related protein 2/3 complex subunit	1317.99	1463.19	1257.11	1620.72	2204.76	2338.08	0.61

Locus_tag	UniProt_gene	Product	Unique exon reads						$\log_2$ Fold Change
			KmWT.1	KmWT.2	KmWT.3	Kmmig1.1	Kmmig1.2	Kmmig1.3	
		1							
KLMA_20233	NSA1	hypothetical protein	510.27	448.58	493.34	306.15	315.09	363.89	-0.56
KLMA_20234	CUE3	hypothetical protein	322.15	321.05	392.24	301.94	262.85	291.29	-0.27
KLMA_20235		transmembrane amino acid transporter protein	851.23	838.99	934.30	533.23	443.14	439.09	-0.89
KLMA_20236	ABD1	mRNA cap guanine-N7 methyltransferase	325.68	381.46	366.66	343.15	246.85	320.68	-0.24
KLMA_20237	PRP5	pre-mRNA-processing ATP-dependent RNA helicase PRP5	255.13	231.56	225.35	313.71	271.28	268.81	0.26
KLMA_20238		uncharacterized protein YGL108C	83.48	115.22	87.71	53.83	123.84	81.25	-0.15
KLMA_20239	RMD9	protein RMD9	11872.51	12149.66	12063.10	3584.59	3187.93	3427.16	-1.82
KLMA_20240	MLC1	myosin light chain 1	404.45	488.85	400.76	337.26	516.44	525.53	0.09
KLMA_20241	ERT1	uncharacterized transcriptional regulatory protein YBR239C	194.00	317.70	303.31	1024.41	452.41	569.61	1.33
KLMA_20242	ARC1	GU4 nucleic-binding protein 1	1939.95	2005.74	1974.58	926.85	1386.72	1223.92	-0.74
KLMA_20243	TRX3	thioredoxin-3	197.52	209.19	238.75	522.30	273.81	257.58	0.71
KLMA_20244	COQ2	para-hydroxybenzoate--polyprenyltransferase	340.96	256.17	287.48	449.97	405.23	486.63	0.60
KLMA_20246	MVD1	diphosphomevalonate decarboxylase	1416.75	1195.84	1503.17	597.15	842.48	984.50	-0.76
KLMA_20247	TUP1	general transcriptional corepressor TUP1	2459.62	2425.23	2252.32	1594.65	1934.33	1937.88	-0.38
KLMA_20248	CSM1	monopolin complex subunit CSM1	63.49	57.05	63.34	134.57	139.85	145.21	1.19
KLMA_20249		UPF0743 protein YCR087C-A	253.96	225.97	236.32	124.48	188.71	126.20	-0.70
KLMA_20250	ABP1	actin-binding protein	1322.69	1780.89	1422.77	621.54	973.90	1016.48	-0.79
KLMA_20251		UPF0587 protein YCR090C	151.67	228.20	209.52	243.07	256.11	291.29	0.42

Locus_tag	UniProt_gene	Product	Unique exon reads						log2 Fold Change
			KmWT.1	KmWT.2	KmWT.3	Kmmig1.1	Kmmig1.2	Kmmig1.3	
KLMA_20252	YMD8	uncharacterized transporter C22E12.01	186.94	275.19	222.92	278.39	223.26	316.35	0.26
KLMA_20253	STP2	transcription factor STP1	3527.19	3677.00	3353.50	2127.04	1909.90	1849.72	-0.84
KLMA_20254	MCM6	DNA replication licensing factor MCM6	500.86	536.95	449.49	360.81	416.18	444.28	-0.28
KLMA_20256	GPH1	glycogen phosphorylase	4353.72	5734.20	4734.86	3121.17	1975.61	2638.87	-0.94
KLMA_20258	JEN1	putative sialic acid transporter	64.67	43.63	58.47	510.52	365.64	363.03	2.90
KLMA_20259	ARO8	aromatic amino acid aminotransferase 1	2083.39	1976.66	1850.33	1746.88	2034.58	1984.56	-0.04
KLMA_20260	KEX1	carboxypeptidase KEX1	913.54	1038.11	785.69	1228.79	865.22	987.96	0.17
KLMA_20261		hypothetical protein	21.16	22.37	34.11	74.01	53.92	98.54	1.55
KLMA_20262	CHO1	CDP-diacylglycerol--serine O-phosphatidyltransferase	1013.48	727.12	732.09	1227.10	1341.22	1460.76	0.70
KLMA_20263	POX1	acyl-coenzyme A oxidase	148.14	133.12	119.38	607.24	470.94	694.94	2.15
KLMA_20264	CHC1	clathrin heavy chain	7066.13	8171.74	7115.08	5164.94	5663.97	5769.56	-0.43
KLMA_20265	SPT16	FACT complex subunit SPT16	1922.32	2089.64	1875.91	946.19	1270.46	1231.70	-0.77
KLMA_20266		hypothetical protein	63.49	74.95	73.09	80.74	59.82	67.42	-0.02
KLMA_20267	NAB2	nuclear polyadenylated RNA-binding protein NAB2	1831.79	2181.37	1776.03	2725.87	2075.86	2053.70	0.24
KLMA_20268	RPS2	40S ribosomal protein S2	27914.15	18349.22	24833.94	6843.69	13895.82	12287.65	-1.11
KLMA_20269		mitochondrial outer membrane protein OM14	123.45	436.27	165.67	3478.62	2151.69	2421.05	3.47
KLMA_20270	MON1	vacuolar fusion protein MON1	518.50	454.17	464.11	530.71	558.56	534.17	0.18
KLMA_20271		zinc finger DNA binding domain	206.93	157.73	202.21	286.80	269.59	220.41	0.46
KLMA_20272	ROT2	glucosidase 2 subunit alpha	685.45	766.28	674.84	1153.09	1022.77	1133.17	0.64
KLMA_20273	SLX1	structure-specific	63.49	66.00	56.03	722.47	491.16	439.96	3.15

Locus_tag	UniProt_gene	Product	Unique exon reads						$\log_2$ Fold Change
			KmWT.1	KmWT.2	KmWT.3	Kmmig1.1	Kmmig1.2	Kmmig1.3	
		endonuclease subunit SLX1							
KLMA_20274	MCX1	mitochondrial clpX-like chaperone MCX1	235.15	290.85	272.86	273.34	261.17	323.27	0.10
KLMA_20275		uncharacterized protein YBR225W	1114.59	1106.35	1043.93	1385.22	1233.39	1381.24	0.29
KLMA_20276	MET13	methylenetetrahydrofolate reductase 2	2170.39	1448.65	1836.93	356.61	544.24	613.69	-1.85
KLMA_20277	TDP1	TYROSYL-DNA PHOSPHODIESTERASE 1	38.80	107.39	51.16	56.35	50.55	87.30	-0.03
KLMA_20278	PDB1	pyruvate dehydrogenase E1 component subunit beta	4410.16	4831.45	3928.46	1514.75	2511.42	2750.37	-0.96
KLMA_20279		uncharacterized membrane protein YBR220C	277.47	260.65	285.04	258.21	283.07	271.41	-0.02
KLMA_20280	YRM1	zinc finger transcription factor YRR1	3981.02	3281.00	3310.87	2507.20	2441.50	2588.74	-0.49
KLMA_20281	SCS3	FIT family protein SCS3	546.71	833.39	696.77	730.04	648.71	589.49	-0.08
KLMA_20282	HSP60	heat shock protein 60	3440.18	3575.21	3081.86	2017.70	3014.38	2487.61	-0.43
KLMA_20283	ATG22	autophagy-related protein 22	368.00	224.85	249.72	338.11	177.76	184.11	-0.27
KLMA_20284		uncharacterized transporter YIL166C	138.74	139.83	166.88	176.62	80.04	127.92	-0.21
KLMA_20285	RNA15	mRNA 3'-end-processing protein RNA15	144.61	146.54	192.46	188.40	215.67	223.00	0.38
KLMA_20286	DST1	transcription elongation factor S-II	343.31	267.36	289.91	368.38	428.82	378.59	0.39
KLMA_20287		hypothetical protein	108.17	86.14	107.20	115.23	174.39	146.94	0.54
KLMA_20288	CYS4	cystathionine beta-synthase	6013.85	4007.01	5308.59	1890.70	2709.41	2757.29	-1.06
KLMA_20289	VPS20	vacuolar protein sorting-associated protein 20	170.48	194.65	193.68	211.11	221.57	211.77	0.21
KLMA_20290	HEM2	delta-aminolevulinic acid dehydratase	2267.98	1567.23	2135.38	1872.20	2178.65	1893.80	-0.01

Locus_tag	UniProt_gene	Product	Unique exon reads						log2 Fold Change
			KmWT.1	KmWT.2	KmWT.3	Kmmig1.1	Kmmig1.2	Kmmig1.3	
KLMA_20291		UPF0424 protein P35G2.02	184.59	167.80	147.39	370.07	251.06	323.27	0.92
KLMA_20292		uncharacterized protein YEL137C	82.30	61.53	90.14	290.17	240.11	197.07	1.64
KLMA_20293	LSM2	U6 snRNA-associated Sm-like protein LSm2	123.45	121.93	192.46	141.30	211.46	184.11	0.30
KLMA_20294	GDI1	rab GDP-dissociation inhibitor	1145.16	1425.16	1098.75	1891.54	1860.19	1923.19	0.63
KLMA_20295		putative 2-hydroxyacid dehydrogenase YGL185C	251.61	223.73	203.43	534.91	401.02	344.88	0.92
KLMA_20296	GPD2	glyceraldehyde-3-phosphate dehydrogenase 2	4.70	8.95	7.31	178.30	94.36	100.27	4.15
KLMA_20297	HOS2	probable histone deacetylase HOS2	492.63	453.05	458.02	465.95	464.20	438.23	-0.04
KLMA_20298	IME4	N6-adenosine-methyltransferase IME4	37.62	69.36	19.49	449.13	195.45	230.78	2.79
KLMA_20299	COX13	cytochrome c oxidase subunit 6A	1728.32	1375.94	1505.60	621.54	1144.93	852.25	-0.82
KLMA_20300	CDC55	protein phosphatase PP2A regulatory subunit B	1059.33	1162.28	1006.17	597.99	762.44	724.33	-0.63
KLMA_20301	RIM15	serine/threonine-protein kinase RIM15	843.00	988.89	963.54	3032.02	1713.60	2268.06	1.33
KLMA_20302	REX2	oligoribonuclease	335.08	315.46	299.66	328.01	282.23	339.69	0.00
KLMA_20303		basic-leucine zipper (bZIP) transcription factor	14212.21	15799.82	13596.73	40856.95	23053.55	28429.45	1.08
KLMA_20304	AGX1	alanine--glyoxylate aminotransferase 1	191.64	178.98	163.23	1249.81	1727.08	1964.68	3.21
KLMA_20305	SHM2	serine hydroxymethyltransferase	22315.33	13080.38	20296.42	3752.80	9086.12	8147.40	-1.41
KLMA_20306	MNL2	uncharacterized glycosyl hydrolase YLR057W	574.93	478.78	624.90	552.58	562.77	547.14	-0.01
KLMA_20307	CAK1	serine/threonine-protein kinase CAK1	179.89	206.95	213.17	236.34	276.33	300.80	0.44
KLMA_20308	ERG3	c-5 sterol desaturase	7142.55	5221.86	7674.20	1197.67	1347.96	1384.69	-2.35

Locus_tag	UniProt_gene	Product	Unique exon reads						log$_2$ Fold Change
			KmWT.1	KmWT.2	KmWT.3	Kmmig1.1	Kmmig1.2	Kmmig1.3	
KLMA_20309	CAF16	CCR4-associated factor 16	418.56	331.12	317.93	232.13	322.67	264.49	-0.38
KLMA_20310	SPT8	transcription factor SPT8	278.65	249.46	241.19	200.17	298.24	351.79	0.14
KLMA_20311	GYP8	GTPase-activating protein GYP8	288.05	274.07	411.73	291.85	318.46	297.34	-0.10
KLMA_20312	coa6	uncharacterized protein YMR244C-A	225.74	308.75	225.35	225.40	296.55	229.92	-0.02
KLMA_20313		uncharacterized protein YMR244W	76.42	295.32	54.82	104.29	41.28	57.05	-1.07
KLMA_20314	ZRC1	zinc/cadmium resistance protein	2141.00	1817.81	2091.52	1931.91	1889.68	1730.44	-0.12
KLMA_20315		hypothetical protein	191.64	154.37	147.39	158.96	229.15	269.68	0.41
KLMA_20316	isp4	sexual differentiation process protein isp4	305.69	321.05	341.08	261.57	194.61	204.85	-0.55
KLMA_20317	SPS4	sporulation-specific protein 4	42.33	49.22	59.69	221.20	101.10	101.99	1.49
KLMA_20318	RPL20B	60S ribosomal protein L20	13926.50	11921.46	14164.37	4416.40	9111.39	7144.75	-0.95
KLMA_20319	DGK1	CTP-dependent diacylglycerol kinase 1	1784.76	1023.57	1464.19	567.71	845.85	850.52	-0.92
KLMA_20320	NOP58	nucleolar protein 58	6534.70	6096.65	6374.45	2228.81	3495.44	3067.59	-1.11
KLMA_20322	SNU66	U4/U6.U5 small nuclear ribonucleoprotein	396.22	345.66	354.47	440.71	315.93	311.17	-0.04
KLMA_20323	SLY41	uncharacterized transporter SLY41	664.29	652.17	722.35	342.31	320.98	324.13	-1.05
KLMA_20324	YHM2	mitochondrial DNA replication protein YHM2	1279.19	784.17	1153.57	1934.43	2337.03	2483.29	1.07
KLMA_20326	MCH5	riboflavin transporter MCH5	1191.01	887.09	1075.61	660.23	376.59	407.11	-1.13
KLMA_20327	RRG7	uncharacterized protein YOR305W	41.15	38.03	60.91	85.79	87.62	111.50	1.03
KLMA_20328		hypothetical protein	0.00	1.12	2.44	3.36	0.84	6.91	1.65
KLMA_20329	CUS1	protein CUS1	138.74	149.90	141.30	375.11	294.87	331.05	1.22
KLMA_20330	NAT1	N-terminal acetyltransferase A complex subunit NAT1	1452.02	1475.50	1471.50	958.81	1359.76	1183.30	-0.33

Locus_tag	UniProt_gene	Product	Unique exon reads						log2 Fold Change
			KmWT.1	KmWT.2	KmWT.3	Kmmig1.1	Kmmig1.2	Kmmig1.3	
KLMA_20331	GAL7	galactose-1-phosphate uridylyltransferase	25152.36	32194.76	28999.93	7550.18	9137.51	7952.92	-1.81
KLMA_20332	GAL10	bifunctional protein GAL10	74652.88	69465.96	71518.63	9622.55	12489.72	12316.17	-2.65
KLMA_20333	GAL1	galactokinase	93495.11	84709.81	93697.06	21646.33	30248.30	27985.18	-1.77
KLMA_20334		hypothetical protein	27.04	11.19	17.05	77.38	40.44	53.59	1.64
KLMA_20335	MRPL51	54S ribosomal protein L51	196.35	171.15	181.50	144.66	230.84	187.56	0.04
KLMA_20336	GCD2	translation initiation factor eIF-2B subunit delta	911.19	889.33	840.51	468.47	589.73	557.51	-0.71
KLMA_20337	MRP13	37S ribosomal protein MRP13	553.77	559.33	566.43	295.21	575.41	463.29	-0.33
KLMA_20338		hypothetical protein	75.25	88.37	104.76	95.04	99.41	118.42	0.22
KLMA_20339	RPL11	60S ribosomal protein L11	15834.71	12821.97	15057.26	5208.68	10297.60	7778.32	-0.91
KLMA_20340	PRE2	proteasome component PRE2	870.04	1030.28	931.87	966.38	1056.47	1069.21	0.13
KLMA_20341	FHL1	pre-rRNA-processing protein FHL1	886.50	836.75	897.76	380.16	432.19	422.67	-1.09
KLMA_20342	COG4	conserved oligomeric Golgi complex subunit 4	322.15	416.14	467.76	264.93	340.36	299.93	-0.41
KLMA_20343	PIL1	sphingolipid long chain base-responsive protein PIL1	1432.04	1943.10	1540.93	2685.50	2431.39	2827.30	0.69
KLMA_20344	YTH1	mRNA 3'-end-processing protein YTH1	30.57	40.27	28.02	37.01	45.49	48.40	0.40
KLMA_20345		protein FUN14	132.86	307.63	216.83	1595.49	1272.98	1580.04	2.76
KLMA_20346	RTS1	serine/threonine-protein phosphatase 2A 56 kDa regulatory subunit delta isoform	1855.30	2420.76	1963.62	1354.10	1222.43	1278.38	-0.69
KLMA_20347	SPO7	sporulation-specific protein SPO7	192.82	193.53	197.34	264.93	213.15	254.12	0.33
KLMA_20348	MDM10	protein of unknown function DUF3722	309.22	323.29	235.10	262.41	208.09	235.97	-0.30

Locus_tag	UniProt_gene	Product	Unique exon reads						log2 Fold Change
			KmWT.1	KmWT.2	KmWT.3	Kmmig1.1	Kmmig1.2	Kmmig1.3	
KLMA_20349	SWC3	SWR1-complex protein 3	471.47	524.65	506.74	998.34	606.58	643.94	0.58
KLMA_20350	SMC5	structural maintenance of chromosomes protein 5	459.71	558.21	498.21	885.63	628.49	716.55	0.56
KLMA_20351	MSE1	glutamyl-tRNA synthetase	202.23	228.20	259.46	178.30	213.99	159.91	-0.32
KLMA_20352	OPI10	protein OPI10	55.26	134.24	95.01	136.25	196.30	152.99	0.77
KLMA_20353	SIL1	nucleotide exchange factor SIL1	191.64	284.14	231.44	317.92	333.62	324.13	0.46
KLMA_20354	CYS3	cystathionine gamma-lyase	6475.91	4146.84	5579.02	1277.57	1746.46	1954.30	-1.70
KLMA_20355		60S acidic ribosomal protein P2-alpha	4612.38	3482.36	4351.15	1242.24	2633.58	2266.34	-1.02
KLMA_20356	RPS15	40S ribosomal protein S15	11736.12	9149.44	11052.06	3385.26	7760.90	6208.65	-0.88
KLMA_20357	DEP1	sds3 super family	1106.36	1048.18	991.55	758.63	826.47	787.43	-0.41
KLMA_20359	NOP12	nucleolar protein 12	1033.47	907.23	1065.86	406.23	476.84	469.34	-1.15
KLMA_20360	SYN8	syntaxin-8	246.90	185.70	154.70	512.20	277.18	345.74	0.95
KLMA_20361		hypothetical protein	529.08	494.44	566.43	315.40	279.70	251.53	-0.91
KLMA_20362	NGL1	RNA exonuclease NGL1	172.83	198.00	190.03	174.94	160.91	163.36	-0.17
KLMA_20363	NTG2	DNA base excision repair N-glycosylase 2	380.94	314.34	331.33	504.64	390.07	497.87	0.44
KLMA_20364	TPD3	protein phosphatase PP2A regulatory subunit A	3754.10	3857.11	3735.99	2376.83	3240.17	3239.60	-0.36
KLMA_20365		hypothetical protein	237.50	171.15	168.10	162.32	219.04	165.09	-0.08
KLMA_20366	MUP1	low-affinity methionine permease	686.63	413.90	726.00	292.69	390.91	422.67	-0.72
KLMA_20367	PSK1	probable serine/threonine-protein kinase YAL017W	1105.18	1567.23	1314.36	1623.24	1209.80	1247.26	0.03
KLMA_20368		uncharacterized membrane protein YAL018C	2.35	0.00	0.00	18.50	11.79	5.19	3.92
KLMA_20369	GAL11	mediator of RNA polymerase II	1084.02	1079.50	976.94	957.12	829.84	837.56	-0.26

Locus_tag	UniProt_gene	Product	Unique exon reads						log2 Fold Change
			KmWT.1	KmWT.2	KmWT.3	Kmmig1.1	Kmmig1.2	Kmmig1.3	
		transcription subunit 15							
KLMA_20370	GSH2	glutathione synthetase	436.20	435.16	443.40	243.07	421.24	434.77	-0.26
KLMA_20371		hypothetical protein	4.70	4.47	2.44	10.09	10.11	17.29	1.68
KLMA_20372	FUN30	uncharacterized ATP-dependent helicase FUN30	420.91	450.82	482.38	421.37	417.87	407.97	-0.12
KLMA_20373	RAT1	5'-3' exoribonuclease 2	1036.99	1082.85	1118.24	737.61	684.09	775.33	-0.56
KLMA_20374	CCC2	copper-transporting ATPase	2196.26	1980.01	2290.08	907.50	1091.01	960.30	-1.13
KLMA_20375		SERF-like protein YDL085C-A	62.31	60.41	53.60	31.12	24.43	22.47	-1.18
KLMA_20376	TIF34	eukaryotic translation initiation factor 3 subunit I	2998.11	2609.81	2946.65	1364.20	2326.08	2044.20	-0.58
KLMA_20377		putative carboxymethylenebutenolidase	644.30	683.50	735.75	523.98	643.65	569.61	-0.25
KLMA_20379		hypothetical protein	110.52	92.85	97.45	74.01	50.55	47.54	-0.80
KLMA_20380		hypothetical protein	4.70	31.32	4.87	314.56	125.53	141.75	3.83
KLMA_20381	OSW5	hypothetical protein	0.00	2.24	0.00	52.99	22.75	22.47	5.40
KLMA_20382		dolichyl-diphosphooligosaccharide--protein glycosyltransferase subunit SWP1	1021.71	1181.29	984.25	806.58	1291.52	1299.99	0.09
KLMA_20383	IMP1	mitochondrial inner membrane protease subunit 1	39.97	33.56	31.67	76.54	83.41	63.96	1.09
KLMA_20384		hypothetical protein	0.00	2.24	2.44	50.46	13.48	13.83	4.06
KLMA_20385	LUC7	protein LUC7	155.20	193.53	148.61	341.47	246.85	258.44	0.77
KLMA_20386	ASM4	nucleoporin ASM4	693.68	673.43	644.39	539.12	608.27	619.74	-0.19
KLMA_20387	NUR1	uncharacterized membrane protein YDL089W	317.45	392.65	377.62	353.24	361.42	366.49	-0.01
KLMA_20388	RIM13	calpain-like protease palB/RIM13	337.43	363.56	264.33	423.89	346.26	363.89	0.23
KLMA_20389	RAM1	protein farnesyltransferase	108.17	134.24	92.58	263.25	262.01	245.48	1.20

Locus_tag	UniProt_gene	Product	Unique exon reads						log$_2$ Fold Change
			KmWT.1	KmWT.2	KmWT.3	Kmmig1.1	Kmmig1.2	Kmmig1.3	
		subunit beta							
KLMA_20390	UBX3	UBX domain-containing protein 3	520.85	635.39	478.72	817.51	725.37	704.45	0.46
KLMA_20391		signal recognition particle subunit SRP14	154.02	190.17	147.39	154.75	161.76	183.24	0.02
KLMA_20392		delta(12) fatty acid desaturase	2079.86	3023.71	2487.41	1225.42	948.63	911.03	-1.30
KLMA_20393	STP3	zinc finger protein STP4	5025.06	4764.33	4490.01	3261.63	2403.59	2136.68	-0.87
KLMA_20394	ARC35	actin-related protein 2/3 complex subunit 2	1608.40	1644.42	1549.46	2044.61	1984.03	1889.48	0.30
KLMA_20395	MRPS12	37S ribosomal protein S12	413.86	469.83	472.63	285.96	346.26	279.19	-0.57
KLMA_20396	PTC6	protein phosphatase 2C homolog 6	138.74	260.65	200.99	283.44	221.57	292.15	0.41
KLMA_20397	RSM19	37S ribosomal protein S19	64.67	54.81	59.69	30.28	62.34	55.32	-0.28
KLMA_20398	DBP6	ATP-dependent RNA helicase DBP6	378.58	335.60	366.66	254.84	320.14	304.25	-0.30
KLMA_20399	SRB8	mediator of RNA polymerase II transcription subunit 12	668.99	771.87	646.83	673.69	598.16	546.27	-0.20
KLMA_20400	ZRG17	protein ZRG17	1004.07	930.72	967.19	1004.22	1050.57	1031.17	0.09
KLMA_20401		hypothetical protein	623.14	531.36	601.75	523.98	516.44	446.87	-0.24
KLMA_20402		probable metabolite transport protein YBR241C	390.34	408.31	377.62	711.54	698.41	688.89	0.84
KLMA_20403	RPL28	ribosomal_L18e super family	17690.01	12629.56	16646.91	6592.22	10040.64	8470.66	-0.90
KLMA_20404		HD domain-containing protein YBR242W	412.68	346.78	460.45	141.30	283.91	261.90	-0.83
KLMA_20405	ALG7	UDP-N-acetylglucosamine--dolichyl-phosphate N-acetylglucosaminephosphotransferase	527.90	476.55	464.11	402.03	573.73	580.85	0.08
KLMA_20406	SEH1	nucleoporin SEH1	778.33	628.68	655.35	398.66	417.87	446.87	-0.71

Locus_tag	UniProt_gene	Product	Unique exon reads						log2 Fold Change
			KmWT.1	KmWT.2	KmWT.3	Kmmig1.1	Kmmig1.2	Kmmig1.3	
KLMA_20407	HYR1	peroxiredoxin HYR1	678.40	808.78	743.06	727.52	712.74	558.37	-0.16
KLMA_20408	ISW1	ISWI chromatin-remodeling complex ATPase ISW1	1232.16	1158.92	1159.66	685.46	786.03	772.73	-0.66
KLMA_20409	LSG1	large subunit GTPase 1	1321.52	990.01	1225.44	571.08	592.26	716.55	-0.91
KLMA_20410	USE1	protein transport protein USE1	221.04	264.00	218.04	251.48	242.63	266.22	0.11
KLMA_20411	ENP1	essential nuclear protein 1	1234.51	1013.50	1279.03	372.59	418.71	412.30	-1.55
KLMA_20412	SRM1	regulator of chromosome condensation	1250.98	1047.06	1232.74	545.85	763.28	715.68	-0.80
KLMA_20413	HIS7	imidazole glycerol phosphate synthase hisHF	1377.95	902.75	1274.16	952.92	1176.10	1304.31	-0.05
KLMA_20414	ARO4	phospho-2-dehydro-3-deoxyheptonate aldolase	4045.68	2599.74	3798.12	1346.53	2160.11	2080.50	-0.90
KLMA_20415	BBP1	protein BBP1	233.97	272.95	241.19	251.48	272.96	258.44	0.06
KLMA_20416		hypothetical protein	51.73	24.61	25.58	30.28	35.38	26.79	-0.14
KLMA_20417	CLN2	G1/S-specific cyclin CLN2	3462.52	3358.19	3567.89	973.10	1204.74	1342.34	-1.56
KLMA_20418	ROT1	protein ROT1	840.65	984.41	694.33	1132.06	1091.01	1267.14	0.47
KLMA_20419	VPS27	vacuolar protein sorting-associated protein 27	460.89	539.19	412.94	654.34	582.99	627.52	0.40
KLMA_20422	RAD14	DNA excision repair protein	146.97	100.68	180.28	242.22	197.98	244.61	0.68
KLMA_20423	MCH1	probable transporter MCH1	911.19	579.46	905.07	1359.99	1037.09	984.50	0.50
KLMA_20424	SEC61	protein transport protein SEC61 subunit alpha	2921.69	2599.74	3005.12	1922.66	2331.98	2189.41	-0.40
KLMA_20425	RSM26	37S ribosomal protein S26	566.70	572.75	576.17	364.18	464.20	417.48	-0.46
KLMA_20426	RAD55	p-loop NTPase super family	226.92	286.37	266.77	851.99	472.63	618.01	1.32
KLMA_20427		hypothetical protein	4533.61	11903.56	2845.54	42887.26	18219.41	27190.83	2.20
KLMA_20428	MUC1	flocculation protein FLO11	302.16	244.98	282.61	746.86	511.38	603.32	1.17

Locus_tag	UniProt_gene	Product	Unique exon reads						log$_2$ Fold Change
			KmWT.1	KmWT.2	KmWT.3	Kmmig1.1	Kmmig1.2	Kmmig1.3	
KLMA_20429	UBP3	ubiquitin carboxyl-terminal hydrolase 3	1246.27	1355.80	1377.70	682.10	633.54	604.18	-1.05
KLMA_20430	SHU2	uncharacterized protein YDR078C	199.87	157.73	196.12	117.75	114.58	114.09	-0.68
KLMA_20431		uncharacterized protein YER152C	129.33	240.51	196.12	497.07	461.68	443.41	1.31
KLMA_20432	PET122	PET122 super family	223.39	258.41	324.02	254.84	284.76	268.81	0.01
KLMA_20433	OXA1	mitochondrial inner membrane protein OXA1	688.98	748.38	593.23	372.59	585.52	570.47	-0.41
KLMA_20434	BEM2	GTPase-activating protein BEM2/IPL2	1305.06	1137.67	1311.92	1377.65	1390.09	1589.55	0.21
KLMA_20435		KH domain-containing protein YLL032C	369.18	330.00	344.73	835.17	776.76	861.76	1.24
KLMA_20436	IRC19	sporulation protein YLL033W	37.62	42.51	36.54	68.13	58.97	45.81	0.57
KLMA_20437	RIX7	ribosome biogenesis ATPase RIX7	688.98	637.63	666.32	413.80	430.51	365.62	-0.72
KLMA_20439	GRC3	protein GRC3	188.12	205.83	205.86	218.68	212.30	197.07	0.07
KLMA_20440	RIM101	pH-response transcription factor pacC/RIM101	3259.12	2115.37	2597.05	3961.39	3689.21	3612.13	0.50
KLMA_20441		cell wall integrity and stress response component 4	1136.93	821.09	885.58	1180.85	868.59	944.74	0.07
KLMA_20442	UBI4	ubiquitin	4370.18	4739.72	3936.98	15385.49	9174.58	8821.59	1.36
KLMA_20443	ECM29	proteasome component ECM29	808.90	1135.43	775.95	1406.25	1049.73	1129.71	0.40
KLMA_20444	TFC6	transcription factor tau 91 kDa subunit	576.11	628.68	525.01	641.73	524.02	517.75	-0.04
KLMA_20445	UBA4	adenylyltransferase and sulfurtransferase UBA4	620.78	552.61	583.48	602.20	483.58	536.76	-0.11
KLMA_20446	ERP5	protein ERP5	137.56	165.56	113.29	241.38	248.53	246.34	0.82
KLMA_20447	CTM1	cytochrome c lysine N-methyltransferase 1	229.27	266.24	286.26	279.23	262.01	314.62	0.13
KLMA_20448	BCP1	protein BCP1	397.40	350.14	355.69	238.86	281.39	293.88	-0.44
KLMA_20449	EAF1	chromatin modification-related protein VID21	691.33	1025.80	810.05	603.04	481.05	523.80	-0.65

Locus_tag	UniProt_gene	Product	Unique exon reads						log$_2$ Fold Change
			KmWT.1	KmWT.2	KmWT.3	Kmmig1.1	Kmmig1.2	Kmmig1.3	
KLMA_20450	GGA2	ADP-ribosylation factor-binding protein GGA2	1093.43	1227.16	1140.17	1134.59	1463.38	1408.90	0.21
KLMA_20451	CLN1	uncharacterized protein YDR357C	48.20	33.56	36.54	63.92	69.08	55.32	0.67
KLMA_20452	SPC110	protein NUF1	337.43	347.90	352.04	317.08	356.37	388.96	0.03
KLMA_20453		uncharacterized protein YBR271W	243.38	206.95	242.41	213.63	198.82	187.56	-0.21
KLMA_20454	TRP4	anthranilate phosphoribosyltransferase	719.55	505.63	751.58	475.20	599.84	637.03	-0.21
KLMA_20455		NASP-related protein sim3	204.58	193.53	168.10	369.22	336.99	336.23	0.88
KLMA_20456		conserved hypothetical protein	636.07	686.85	571.30	480.24	406.92	445.14	-0.51
KLMA_20457	POM33	UPF0121 membrane protein YLL023C	965.27	760.68	867.31	2352.44	1807.96	1647.46	1.16
KLMA_20458		putative uncharacterized protein YAL004W	51.73	31.32	63.34	25.23	62.34	70.88	0.12
KLMA_20459	SSA2	heat shock protein SSA2	4152.67	2822.36	3633.67	2323.00	4941.97	4350.29	0.13
KLMA_20460		uncharacterized mitochondrial carrier YPR011C	172.83	195.76	179.06	240.54	280.54	293.02	0.57
KLMA_20461		UPF0495 protein KLLA0D04334g	537.31	416.14	630.99	302.78	543.40	312.03	-0.45
KLMA_20462	uapC	purine permease	296.28	219.26	213.17	1174.12	1106.17	1080.44	2.21
KLMA_20463	HAH1	UPF0082 protein YGR021W	122.28	127.53	131.56	128.68	117.10	140.03	0.02
KLMA_20464	VMA7	v-type proton ATPase subunit F	597.27	571.63	609.06	510.52	746.43	616.28	0.08
KLMA_20465	CHS5	chitin biosynthesis protein CHS5	725.42	812.14	634.64	663.60	764.97	840.15	0.06
KLMA_20466	SDH1	succinate dehydrogenase [ubiquinone] flavoprotein subunit	7498.80	6263.32	6878.76	5257.46	9356.55	8625.38	0.17
KLMA_20467	DBR1	lariat debranching enzyme	166.95	164.44	170.54	343.15	257.80	286.10	0.82
KLMA_20468	MCR1	NADH-cytochrome b5 reductase 2	2188.03	2480.05	2271.81	3793.17	4172.79	3891.32	0.77
KLMA_20469		uncharacterized protein YKL151C	417.38	513.46	512.83	1040.39	877.02	783.10	0.90

Locus_tag	UniProt_gene	Product	Unique exon reads						log2 Fold Change
			KmWT.1	KmWT.2	KmWT.3	Kmmig1.1	Kmmig1.2	Kmmig1.3	
KLMA_20470	RPC34	DNA-directed RNA polymerase III subunit RPC6	304.51	253.93	261.90	207.74	224.10	236.83	-0.29
KLMA_20471	ADP1	probable ATP-dependent permease	1864.71	1907.30	1856.42	1463.44	1588.07	1663.88	-0.26
KLMA_20472		uncharacterized protein YFR018C	638.42	570.51	543.28	637.52	602.37	570.47	0.05
KLMA_20473	FAB1	1-phosphatidylinositol-3-phosphate 5-kinase FAB1	977.03	904.99	1065.86	2963.05	2464.25	3066.73	1.53
KLMA_20474	ATG18	autophagy-related protein 18	590.22	606.31	538.41	637.52	550.14	528.12	-0.02
KLMA_20475	ROD1	protein ROD1	1142.81	1540.38	1355.77	1872.20	1382.51	1459.89	0.22
KLMA_20476	STP22	suppressor protein STP22 of temperature-sensitive alpha-factor receptor and arginine permease	47.03	111.87	96.23	119.43	106.99	77.79	0.25
KLMA_20477		chromosome segregation ATPases	177.54	297.56	224.14	299.42	238.42	250.66	0.17
KLMA_20478	CBF5	centromere/microtubule-binding protein CBF5	3829.35	3427.55	3638.54	1084.97	1527.41	1396.80	-1.44
KLMA_20479	DNAJB13	protein SIS1	596.09	563.80	644.39	735.09	889.66	809.90	0.43
KLMA_20480	LST8	target of rapamycin complex subunit LST8	1158.09	865.84	986.68	689.67	659.66	624.93	-0.61
KLMA_20481		RFX-like DNA-binding protein RFX1	562.00	494.44	655.35	201.85	198.82	183.24	-1.55
KLMA_20482	RLP7	ribosome biogenesis protein RLP7	1335.63	1290.92	1361.87	494.54	654.60	624.06	-1.17
KLMA_20483	LEU2	3-isopropylmalate dehydrogenase	2284.44	1460.96	2130.50	981.52	1101.12	1272.33	-0.81
KLMA_20484	NFS1	cysteine desulfurase	949.99	936.31	974.50	982.36	1061.52	1065.75	0.12
KLMA_20485	PET8	putative mitochondrial carrier protein PET8	245.73	171.15	219.26	150.55	203.88	228.19	-0.13
KLMA_20486	DCC1	sister chromatid cohesion protein DCC1	125.80	130.88	104.76	86.63	101.94	108.91	-0.28

Locus_tag	UniProt_gene	Product	Unique exon reads						log_2 Fold Change
			KmWT.1	KmWT.2	KmWT.3	Kmmig1.1	Kmmig1.2	Kmmig1.3	
KLMA_20487	BUD3	bud site selection protein 3 homolog	873.57	896.04	845.38	622.38	756.54	726.92	-0.31
KLMA_20488	HRB1	RRM (RNA recognition motif)	1098.13	856.89	1089.00	479.40	553.51	513.43	-0.98
KLMA_20489		GAL4-like Zn2Cys6 binuclear cluster DNA-binding domain	405.63	333.36	475.07	315.40	313.40	307.71	-0.37
KLMA_20490	TPN1	vitamin B6 transporter TPN1	2010.50	1007.90	1784.56	1328.03	1838.29	1775.38	0.04
KLMA_20491	COX4	cytochrome c oxidase subunit 4	4193.82	4211.72	4508.29	2019.38	2837.46	2261.15	-0.86
KLMA_20492	RPS26A	40S ribosomal protein S26-A	13868.89	12121.70	13621.09	4618.25	8740.70	6829.26	-0.97
KLMA_20493	FRS1	phenylalanyl-tRNA synthetase beta chain	2988.70	3028.19	3196.36	1258.22	2010.99	1901.58	-0.83
KLMA_20494		uncharacterized membrane protein YFL034W	460.89	535.83	506.74	737.61	551.82	611.96	0.34
KLMA_20495	RPL22A	60S ribosomal protein L22-A	8596.93	6196.21	8775.38	2444.96	5230.94	4039.12	-1.01
KLMA_20496		hypothetical protein	119.92	98.44	99.89	63.92	133.11	128.79	0.03
KLMA_20497	GAB1	GPI transamidase component GAB1	1239.22	1186.89	1193.76	1041.23	1011.82	1003.51	-0.24
KLMA_20498	SEC23	protein transport protein SEC23	4862.81	3968.97	4454.69	1817.53	3595.69	3405.55	-0.59
KLMA_20499	AOS1	DNA damage tolerance protein RHC31	154.02	178.98	163.23	381.00	369.01	326.73	1.12
KLMA_20500	HDA3	HDA1 complex subunit 3	537.31	585.05	556.68	460.06	479.37	436.50	-0.29
KLMA_20501	PRP4	U4/U6 small nuclear ribonucleoprotein PRP4	155.20	159.97	174.19	272.50	275.49	271.41	0.74
KLMA_20502	BET2	geranylgeranyl transferase type-2 subunit beta	191.64	214.78	191.25	223.72	316.77	318.08	0.52
KLMA_20503	DPB2	DNA polymerase epsilon subunit B	385.64	293.09	283.82	248.11	223.26	279.19	-0.36
KLMA_20504		NAP1-binding protein	108.17	93.97	131.56	272.50	212.30	219.55	1.08
KLMA_20505		uncharacterized vacuolar membrane protein YGR125W	1377.95	925.12	1533.62	603.88	633.54	711.36	-0.98

Locus_tag	UniProt_gene	Product	Unique exon reads						log$_2$ Fold Change
			KmWT.1	KmWT.2	KmWT.3	Kmmig1.1	Kmmig1.2	Kmmig1.3	
KLMA_20506		hypothetical protein	130.51	149.90	133.99	183.35	126.37	133.97	0.10
KLMA_20507		uncharacterized protein YGR127W	306.87	364.68	304.53	725.83	491.16	449.46	0.77
KLMA_20508	UTP8	U3 small nucleolar RNA-associated protein 8	1317.99	1068.31	1259.54	468.47	580.47	592.08	-1.15
KLMA_20509	SYF2	pre-mRNA-splicing factor SYF2	71.72	72.71	75.52	172.42	123.84	159.04	1.05
KLMA_20510		uncharacterized protein YPR148C	620.78	713.70	529.88	587.06	611.64	618.01	-0.04
KLMA_20511	RSM18	ribosomal_S18 super family	380.94	350.14	398.33	224.56	421.24	298.20	-0.26
KLMA_20512	PKP1	[Pyruvate dehydrogenase [lipoamide]] kinase	399.75	384.82	438.53	608.09	579.62	602.45	0.55
KLMA_20513	GVP36	protein GVP36	1345.03	1306.58	1119.46	861.24	1126.39	974.13	-0.35
KLMA_20514	TPA1	PKHD-type hydroxylase TPA1	2592.48	2450.96	2762.71	795.64	676.51	707.04	-1.84
KLMA_20515	ISD11	protein ISD11	65.84	82.78	34.11	77.38	103.62	63.96	0.42
KLMA_20516		hypothetical protein	194.00	156.61	143.74	376.79	207.25	196.21	0.66
KLMA_20517	CAJ1	protein CAJ1	674.87	651.05	593.23	1116.93	1083.43	959.43	0.72
KLMA_20518	TED1	protein TED1	513.79	645.46	715.04	587.90	780.98	853.98	0.25
KLMA_20519		protein SAP1	465.59	574.99	511.61	264.93	333.62	398.47	-0.64
KLMA_20520		putative dioxygenase C576.01c	42.33	26.85	23.14	99.24	30.33	52.73	0.98
KLMA_20521	NOT3	general negative regulator of transcription subunit 3	1198.07	1042.58	1165.75	767.05	859.33	854.85	-0.46
KLMA_20522		pheromone-regulated membrane protein 3	81.13	119.70	182.72	269.98	183.66	203.12	0.78
KLMA_20523		hypothetical protein	38.80	63.76	46.29	169.05	115.42	142.62	1.52
KLMA_20524	CST6	bZIP_1 super family	1212.18	1459.84	1104.84	629.95	732.11	797.80	-0.81
KLMA_20525	CKA1	casein kinase II subunit alpha	958.22	884.85	989.12	784.71	946.10	859.17	-0.13
KLMA_20526	CAP2	F-actin-capping protein subunit beta	1108.71	1086.21	1048.81	788.07	1321.85	1116.74	-0.01
KLMA_20527	ERG28	ergosterol	1319.17	823.33	1182.80	422.21	608.27	546.27	-1.08

Locus_tag	UniProt_gene	Product	Unique exon reads						log2 Fold Change
			KmWT.1	KmWT.2	KmWT.3	Kmmig1.1	Kmmig1.2	Kmmig1.3	
		biosynthetic protein 28							
KLMA_20528	PKAR	cAMP-dependent protein kinase regulatory subunit	1959.94	2412.93	1942.91	2872.22	2356.41	2392.53	0.27
KLMA_20529	IRC5	uncharacterized ATP-dependent helicase YFR038W	331.56	354.61	287.48	369.22	239.26	227.32	-0.22
KLMA_20530	RSC8	chromatin structure-remodeling complex protein RSC8	992.31	883.73	974.50	686.30	851.74	844.47	-0.26
KLMA_20531		protein SRN2	96.41	82.78	107.20	168.21	183.66	112.37	0.70
KLMA_20532	PEX7	peroxisomal targeting signal 2 receptor	154.02	225.97	147.39	206.06	176.92	224.73	0.20
KLMA_20533	SAN1	protein SAN1	605.50	607.43	562.77	470.99	456.62	522.93	-0.29
KLMA_20534	YPS1	aspartic proteinase 3	1481.42	2076.22	1302.18	1808.28	1237.60	2083.96	0.08
KLMA_20535		ubiquitin-conjugating enzyme E2-21 kDa	106.99	108.51	84.05	161.48	88.46	152.13	0.42
KLMA_20536		yjgF_YER057c_UK114_family	241.02	194.65	202.21	694.71	441.46	548.86	1.40
KLMA_20537	SUE1	PET20 super family	530.25	338.95	499.43	760.32	473.47	436.50	0.29
KLMA_20538	CAF130	protein CAF130	438.55	387.05	417.82	412.12	328.57	392.42	-0.13
KLMA_20539	URN1	pre-mRNA-splicing factor URN1	208.10	204.71	185.16	328.85	289.81	272.27	0.57
KLMA_20540	PRE9	proteasome component Y13	696.03	822.21	691.90	952.08	1061.52	1084.76	0.49
KLMA_20541		hypothetical protein	72.90	46.98	45.07	79.90	66.56	89.03	0.51
KLMA_20542	PIN3	[PSI+] inducibility protein 3	386.81	288.61	372.75	1989.10	1219.91	1489.28	2.16
KLMA_20543	NCA2	nuclear control of ATPase protein 2	312.74	465.36	376.40	744.34	492.01	532.44	0.62
KLMA_20544	TPO3	polyamine transporter 3	2466.68	1609.74	2419.20	3947.93	3633.60	3428.89	0.76
KLMA_20545	CBF2	centromere DNA-binding protein complex CBF3 subunit A	498.51	462.00	511.61	455.01	410.29	406.25	-0.21
KLMA_20546	VPS62	vacuolar protein sorting-associated	315.10	346.78	359.35	1581.19	1181.15	1201.45	1.96

Locus_tag	UniProt_gene	Product	Unique exon reads						log2 Fold Change
			KmWT.1	KmWT.2	KmWT.3	Kmmig1.1	Kmmig1.2	Kmmig1.3	
		protein 62							
KLMA_20547		protein BTN2	119.92	105.15	81.61	330.54	321.83	223.87	1.51
KLMA_20548		kpsF-like protein	681.92	646.58	704.08	1461.76	1073.32	1036.36	0.81
KLMA_20549	KRE6	beta-glucan synthesis-associated protein KRE6	3031.03	2995.75	2609.23	2344.87	1948.65	2280.16	-0.39
KLMA_20550	SPP41	DUF3020 super family	519.67	554.85	568.86	563.51	454.94	554.91	-0.06
KLMA_20551	CYP707A7	lanosterol 14-alpha demethylase	8286.53	5427.69	8045.72	977.31	1707.70	1895.53	-2.25
KLMA_20552	RMT2	arginine N-methyltransferase 2	543.19	435.16	462.89	277.55	248.53	257.58	-0.88
KLMA_20553	SOD2	superoxide dismutase [Mn]	1270.96	1106.35	1235.18	1756.97	1802.06	1861.82	0.59
KLMA_20554	PKH3	serine/threonine-protein kinase PKH3	422.09	434.04	409.29	391.93	324.35	454.65	-0.11
KLMA_20555	TDA3	UPF0673 membrane protein YHR009C	2143.35	2619.88	2358.29	894.05	1032.88	1099.46	-1.23
KLMA_20556	UGO1	mitochondrial fusion and transport protein UGO1	484.40	583.94	479.94	421.37	488.64	486.63	-0.15
KLMA_20557		SMC_prok_A	176.36	156.61	176.63	148.03	137.32	159.91	-0.19
KLMA_20558	RPL27A	60S ribosomal protein L27-A	15700.68	13305.23	15431.22	5869.75	10727.26	8600.32	-0.82
KLMA_20559	DIA4	seryl-tRNA synthetase	192.82	334.48	196.12	207.74	208.09	242.88	-0.14
KLMA_20560	VPS29	vacuolar protein sorting-associated protein 29	129.33	152.14	107.20	172.42	187.87	215.22	0.56
KLMA_20561	ARD1	N-terminal acetyltransferase A complex catalytic subunit ARD1	291.58	291.97	353.26	179.99	217.36	224.73	-0.59
KLMA_20562	TRS31	transport protein particle 31 kDa subunit	72.90	79.42	103.54	69.81	79.19	90.76	-0.09
KLMA_20564	ELG1	telomere length regulation protein ELG1	238.67	229.32	179.06	256.52	210.62	235.97	0.12
KLMA_20565	SPT3	protein SPT3	538.48	525.77	573.74	300.26	330.25	331.91	-0.77
KLMA_20566	THI80	thiamine pyrophosphokinase	483.22	393.76	459.23	188.40	277.18	285.24	-0.83

Locus_tag	UniProt_gene	Product	Unique exon reads						log$_2$ Fold Change
			KmWT.1	KmWT.2	KmWT.3	Kmmig1.1	Kmmig1.2	Kmmig1.3	
KLMA_20567		hypothetical protein	5.88	2.24	2.44	1.68	1.68	0.86	-1.31
KLMA_20568	LSC1	succinyl-CoA ligase [ADP-forming] subunit alpha	1466.13	1639.94	1330.19	531.55	1024.45	1014.75	-0.79
KLMA_20571	ARP8	actin-like protein ARP8	698.38	727.12	678.50	505.48	512.23	534.17	-0.44
KLMA_20572	SFL1	flocculation suppression protein	767.75	859.12	732.09	509.68	470.10	491.82	-0.68
KLMA_20573	RUP1	hypothetical protein	466.76	542.55	370.31	814.14	760.76	875.59	0.83
KLMA_20574	SIA1	protein SIA1	757.17	1025.80	644.39	732.56	481.90	658.64	-0.37
KLMA_20575	IDH2	isocitrate dehydrogenase [NAD] subunit 2	1239.22	1274.14	1196.20	1568.57	2421.28	1978.51	0.69
KLMA_20577	UBA2	ubiquitin-activating enzyme E1-like	973.50	963.16	962.32	375.11	530.76	549.73	-0.99
KLMA_20578	SAC7	GTPase-activating protein SAC7	516.14	549.26	437.31	472.67	389.22	362.16	-0.30
KLMA_20579	RVS167	reduced viability upon starvation protein 167	1201.59	1145.50	1199.85	1534.09	1237.60	1574.85	0.29
KLMA_20580		probable metabolite transport protein YDR387C	1303.88	1529.20	1388.66	1502.13	1016.03	956.84	-0.28
KLMA_20581	MUS81	crossover junction endonuclease MUS81	129.33	127.53	119.38	363.34	204.72	261.90	1.14
KLMA_20582	EFT1	elongation factor 2	93816.08	65512.65	83837.54	33114.16	52739.07	49628.59	-0.84
KLMA_20584	VPS17	vacuolar protein sorting-associated protein 17	516.14	467.60	505.52	650.98	578.78	513.43	0.23
KLMA_20585		putative uncharacterized hydrolase YOR131C	185.77	192.41	249.72	484.45	299.92	319.81	0.82
KLMA_20586	ATO2	hypothetical protein	3577.74	1614.21	2154.87	7876.51	4210.70	3436.67	1.08
KLMA_20587	ATO3	ammonia transport outward protein 3	2548.98	1444.18	2076.91	3160.70	1959.60	1797.86	0.19
KLMA_20588		hypothetical protein	250.43	212.54	224.14	132.89	191.24	176.33	-0.46
KLMA_20590		60S acidic ribosomal protein P2-B	6011.50	4246.40	5508.37	2165.73	4314.33	3372.71	-0.68
KLMA_20591	ORT1	mitochondrial ornithine carrier	552.59	409.43	595.66	259.05	250.22	235.10	-1.06

Locus_tag	UniProt_gene	Product	Unique exon reads						log2 Fold Change
			KmWT.1	KmWT.2	KmWT.3	Kmmig1.1	Kmmig1.2	Kmmig1.3	
		protein							
KLMA_20592	PMP47A	peroxisomal membrane protein PMP47B	533.78	428.44	587.14	436.51	515.60	539.36	-0.05
KLMA_20593		uncharacterized mitochondrial outer membrane protein YDR381C-A	70.54	80.54	51.16	49.62	64.87	58.78	-0.23
KLMA_20594	YRA1	hypothetical protein	2093.97	1977.77	2036.71	1799.87	2087.66	1943.93	-0.07
KLMA_20596	AFI1	ARF3-interacting protein 1	489.10	580.58	506.74	320.44	373.22	337.96	-0.61
KLMA_20597	ARO10	transaminated amino acid decarboxylase	325.68	241.63	265.55	849.47	544.24	656.04	1.30
KLMA_20598	ADE2	phosphoribosylaminoimidazole carboxylase	3489.56	2855.91	3431.46	1268.32	1912.42	1669.07	-1.01
KLMA_20599		GAL4-like Zn2Cys6 binuclear cluster DNA-binding domain	1748.31	2406.22	1822.32	1354.95	1103.65	1089.09	-0.75
KLMA_20600	ugcg-b	glyco_tranf_GTA_type super family	320.97	374.75	308.19	751.07	344.57	325.00	0.50
KLMA_20601	RGA1	rho-type GTPase-activating protein 1	1377.95	1307.70	1163.31	1162.34	986.54	982.77	-0.30
KLMA_20602	LSM6	U6 snRNA-associated Sm-like protein LSm6	139.91	119.70	118.16	192.60	201.35	160.77	0.55
KLMA_20603	ARH1	probable NADPH:adrenodoxin oxidoreductase	155.20	200.24	158.36	234.66	197.98	218.68	0.34
KLMA_20604	IAH1	isoamyl acetate-hydrolyzing esterase	21.16	39.15	9.75	627.43	427.14	521.21	4.48
KLMA_20605		mitochondrial chaperone BCS1	238.67	295.32	282.61	319.60	394.28	345.74	0.38
KLMA_20606		hypothetical protein	25.87	26.85	32.89	38.69	32.86	25.07	0.18
KLMA_20607		YTH super family	7.05	11.19	9.75	518.09	271.28	314.62	5.30
KLMA_20608	CAT5	ubiquinone biosynthesis monooxygenase Coq7	765.40	770.75	857.56	513.05	598.16	599.00	-0.48
KLMA_20609	FRQ1	calcium-binding protein NCS-1	349.19	350.14	344.73	895.73	663.03	601.59	1.05

Locus_tag	UniProt_gene	Product	Unique exon reads						log2 Fold Change
			KmWT.1	KmWT.2	KmWT.3	Kmmig1.1	Kmmig1.2	Kmmig1.3	
KLMA_20610	UBP2	ubiquitin carboxyl-terminal hydrolase 2	567.88	864.72	645.61	917.59	804.57	841.88	0.30
KLMA_20611	VPS74	vacuolar protein sorting-associated protein 74	676.04	748.38	727.22	563.51	764.13	700.99	-0.09
KLMA_20612	CTS2	sporulation-specific chitinase 2	35.27	174.51	38.98	582.85	245.16	401.06	2.30
KLMA_20613	LEO1	RNA polymerase-associated protein LEO1	604.32	680.14	510.39	456.69	578.78	656.04	-0.09
KLMA_20614	PFY1	profilin binds actin monomers	1502.58	1650.01	1478.81	1468.49	1646.20	1428.78	-0.03
KLMA_20615		YCII super family	174.01	181.22	174.19	370.91	329.41	298.20	0.92
KLMA_20616		uncharacterized protein YDR370C	532.61	364.68	553.03	770.41	647.02	612.83	0.49
KLMA_20617		SAM50-like protein SpAC17C9.06	584.34	712.58	650.48	476.88	513.07	487.50	-0.40
KLMA_20618		DNA repair protein XRS2	399.75	441.87	381.27	508.00	443.14	469.34	0.22
KLMA_20619	YPR1	putative reductase 1	9452.86	8112.45	9189.54	2960.53	3838.33	3166.13	-1.42
KLMA_20620	LEM3	alkylphosphocholine resistance protein LEM3	530.25	570.51	532.32	517.25	693.36	661.23	0.20
KLMA_20621	SNG1	nitrosoguanidine resistance protein SNG1	277.47	303.15	276.51	829.28	614.17	635.30	1.28
KLMA_20622	FIG4	polyphosphoinositid e phosphatase	464.41	410.54	461.67	500.43	550.14	436.50	0.15
KLMA_20623	RRP40	exosome complex component RRP40	239.85	248.34	304.53	275.87	231.68	261.03	-0.04
KLMA_20624	PFA3	palmitoyltransferase PFA3	351.54	227.09	361.78	267.46	267.07	229.05	-0.30
KLMA_20625	EGT2	hypothetical protein	4943.94	4594.30	4159.90	5252.41	8179.61	7111.90	0.58
KLMA_20626	MDJ2	mitochondrial DnaJ homolog 2	104.64	109.63	104.76	76.54	95.20	75.20	-0.37
KLMA_20627	PEX6	peroxisomal biogenesis factor 6	273.94	326.65	306.97	374.27	517.28	509.97	0.63
KLMA_20628	RPD3	histone deacetylase RPD3	1114.59	1083.97	1012.26	870.50	1153.35	1121.07	-0.03
KLMA_20629	RIB4	6,7-dimethyl-8-ribityllumazine synthase	352.72	356.85	381.27	232.97	415.34	305.12	-0.19
KLMA_20630	NOP8	60S ribosome	326.85	372.51	352.04	275.87	256.11	296.47	-0.34

Locus_tag	UniProt_gene	Product	Unique exon reads						log2 Fold Change
			KmWT.1	KmWT.2	KmWT.3	Kmmig1.1	Kmmig1.2	Kmmig1.3	
		subunit biogenesis protein NOP8							
KLMA_20631	CTR9	RNA polymerase-associated protein CTR9	1563.72	1695.87	1615.24	687.14	961.27	822.86	-0.98
KLMA_20632	PSF3	DNA replication complex GINS protein PSF3	110.52	93.97	112.07	52.15	111.21	98.54	-0.27
KLMA_20633		hypothetical protein	0.00	0.00	2.44	0.00	0.00	0.00	-2.37
KLMA_20634	PEX11	peroxisomal membrane protein PMP27	104.64	139.83	71.87	354.09	209.78	248.07	1.36
KLMA_20635	SPT20	transcription factor SPT20	253.96	338.95	247.28	276.71	230.00	279.19	-0.10
KLMA_20636	DCP1	mRNA-decapping enzyme subunit 1	293.93	285.26	298.44	347.36	412.81	360.44	0.35
KLMA_20637	SDL1	L-serine dehydratase	183.41	222.61	227.79	376.79	302.45	344.88	0.69
KLMA_20638	PLT5	putative polyol transporter 2	574.93	421.73	556.68	515.57	401.86	418.35	-0.22
KLMA_20639	MGT1	methylated-DNA--protein-cysteine methyltransferase	47.03	19.02	41.42	37.85	32.01	25.93	-0.16
KLMA_20640	PET494	COX3 mRNA-specific translational activator PET494	469.12	389.29	425.13	137.93	183.66	183.24	-1.35
KLMA_20641	MSS11	transcription activator MSS11	238.67	279.66	274.08	105.97	183.66	152.99	-0.84
KLMA_20642	INP2	inheritance of peroxisomes protein 2	340.96	317.70	313.06	221.20	314.24	262.76	-0.28
KLMA_20643	QRI1	UDP-N-acetylglucosamine pyrophosphorylase	165.78	135.36	116.94	132.05	137.32	182.38	0.11
KLMA_20644	DNF3	hypothetical protein	656.06	674.55	687.02	984.88	673.14	672.47	0.21
KLMA_20645		protein HLJ1	445.60	504.51	428.78	368.38	367.32	337.96	-0.36
KLMA_20646	POL3	DNA polymerase delta catalytic subunit	696.03	774.11	620.03	569.40	534.97	581.71	-0.31
KLMA_20647	DUN1	DNA damage response protein kinase DUN1	238.67	224.85	192.46	282.60	242.63	277.46	0.29
KLMA_20648		uncharacterized protein YMR160W	358.60	378.10	292.35	461.74	310.87	357.84	0.14

Locus_tag	UniProt_gene	Product	Unique exon reads						log$_2$ Fold Change
			KmWT.1	KmWT.2	KmWT.3	Kmmig1.1	Kmmig1.2	Kmmig1.3	
KLMA_20649	ATG16	autophagy protein 16	23.51	31.32	34.11	65.60	42.12	41.49	0.75
KLMA_20650	GET3	ATPase GET3	1120.47	1064.96	980.59	791.44	1128.08	987.09	-0.12
KLMA_20651		binder of USO1 and GRH1 protein 1	402.10	447.46	366.66	589.58	630.17	683.70	0.65
KLMA_20652	MRPS8	37S ribosomal protein S8	445.60	379.22	431.22	279.23	378.27	389.82	-0.26
KLMA_20653	AIM36	uncharacterized protein YMR157C	224.56	262.88	193.68	232.13	241.79	216.09	0.02
KLMA_20654	SNU23	U4/U6.U5 small nuclear ribonucleoprotein component	52.91	50.34	51.16	176.62	135.64	107.18	1.44
KLMA_20655	TPP1	polynucleotide 3'-phosphatase	137.56	132.00	120.59	111.86	137.32	168.55	0.10
KLMA_20656	RPN6	26S proteasome regulatory subunit RPN6	1353.26	1732.79	1480.02	1291.87	1296.57	1282.70	-0.24
KLMA_20657	PMT1	dolichyl-phosphate-mannose--protein mannosyltransferase 1	5832.79	5319.18	5557.09	3514.78	4007.66	3845.51	-0.56
KLMA_20658		uncharacterized membrane protein YMR155W	279.82	277.43	208.30	1119.45	855.96	1019.94	1.97
KLMA_20659	MSC1	meiotic sister chromatid recombination protein 1	152.84	535.83	137.65	5102.70	3004.27	4132.47	3.89
KLMA_20660		UPF0549 protein C1D4.09c	122.28	118.58	87.71	507.16	241.79	267.09	1.63
KLMA_20661	PRE8	proteasome component Y7	1007.60	1194.72	905.07	1076.56	1180.31	1083.04	0.10
KLMA_20662	UTP14	U3 small nucleolar RNA-associated protein 14	1056.98	979.94	997.65	568.56	577.94	576.52	-0.82
KLMA_20663	GIM5	prefoldin alpha subunit	217.51	269.59	293.57	158.96	274.65	240.29	-0.21
KLMA_20664	RAD10	DNA repair protein RAD10	115.22	125.29	123.03	177.46	123.00	127.92	0.24
KLMA_20665		asparagine synthetase domain-containing protein YML096W	309.22	305.39	345.95	527.34	424.61	407.11	0.50

Locus_tag	UniProt_gene	Product	Unique exon reads						log$_2$ Fold Change
			KmWT.1	KmWT.2	KmWT.3	Kmmig1.1	Kmmig1.2	Kmmig1.3	
KLMA_20666	VPS9	vacuolar protein sorting-associated protein 9	433.84	493.32	394.67	427.26	391.75	463.29	-0.04
KLMA_20667	ERO1	endoplasmic oxidoreductin-1	1901.15	1708.18	1895.40	1228.79	1074.16	1013.89	-0.73
KLMA_20668	TAF13	transcription initiation factor TFIID subunit 13	199.87	204.71	143.74	178.30	189.56	193.62	0.03
KLMA_20669	ARG81	arginine metabolism regulation protein II	155.20	249.46	179.06	177.46	149.96	148.67	-0.30
KLMA_20670	EAR1	protein EAR1	803.02	921.77	823.45	1844.44	1178.63	1208.37	0.73
KLMA_20671	PHO2	regulatory protein PHO2	1300.36	1152.21	1127.98	825.92	815.52	753.72	-0.58
KLMA_20672	aldA	putative aldehyde dehydrogenase-like protein C922.07c	194.00	241.63	144.96	306.99	188.71	312.90	0.48
KLMA_20673	ALD2	aldehyde dehydrogenase [NAD(P)+] 1	216.33	411.66	247.28	816.67	647.02	699.26	1.30
KLMA_20674	CEP3	centromere DNA-binding protein complex CBF3 subunit B	296.28	247.22	250.93	189.24	246.00	238.56	-0.24
KLMA_20675	MLH1	DNA mismatch repair protein MLH1	101.11	121.93	107.20	248.95	218.20	240.29	1.10
KLMA_20676	NSE4	non-structural maintenance of chromosome element 4	130.51	168.92	143.74	202.70	190.40	195.34	0.41
KLMA_20677	gcp	putative protease QRI7	192.82	177.87	198.55	138.77	142.38	161.63	-0.36
KLMA_20678		uncharacterized mitochondrial carrier YMR166C	757.17	674.55	773.51	517.25	705.15	673.33	-0.22
KLMA_20679	PAH1	protein SMP2	823.01	841.23	780.82	489.50	514.75	539.36	-0.66
KLMA_20680		FAS1 domain-containing protein YLR001C	670.17	745.02	760.11	502.11	516.44	556.64	-0.47
KLMA_20681	AVT1	vacuolar amino acid transporter 1	1361.49	1163.40	1305.83	1415.50	1336.17	1456.44	0.14
KLMA_20682	MPP10	U3 small nucleolar RNA-associated protein MPP10	998.19	976.58	1073.17	336.42	532.45	480.58	-1.18

Locus_tag	UniProt_gene	Product	Unique exon reads						log$_2$ Fold Change
			KmWT.1	KmWT.2	KmWT.3	Kmmig1.1	Kmmig1.2	Kmmig1.3	
KLMA_20683	NOC3	nucleolar complex-associated protein 3	750.11	722.65	912.38	439.87	447.36	452.92	-0.83
KLMA_20684		hypothetical protein	181.06	143.19	142.52	251.48	200.51	169.41	0.41
KLMA_20685		uncharacterized protein YJR003C	176.36	209.19	191.25	519.77	356.37	392.42	1.14
KLMA_20686		hypothetical protein	64.67	86.14	38.98	107.66	129.74	116.69	0.90
KLMA_20687	GPI14	GPI mannosyltransferase 1	325.68	325.53	384.93	697.24	459.15	524.66	0.70
KLMA_20688	SKI6	exosome complex component SKI6	298.64	351.26	316.71	254.84	254.43	306.85	-0.24
KLMA_20689	FYV8	protein FYV8	156.37	210.31	175.41	508.84	455.78	465.02	1.40
KLMA_20690	SMF2	manganese transporter SMF2	1266.26	1236.11	1333.85	878.07	794.46	882.50	-0.59
KLMA_20691	GRP78	78 kDa glucose-regulated protein homolog	2191.56	2591.91	2067.16	2187.59	1998.36	1944.80	-0.16
KLMA_20692	PML1	pre-mRNA leakage protein 1	146.97	105.15	158.36	105.97	91.83	120.15	-0.37
KLMA_20693	DBP4	ATP-dependent RNA helicase DBP4	819.48	682.38	909.94	426.42	493.69	554.91	-0.71
KLMA_20694	CTK2	CTD kinase subunit beta	253.96	224.85	292.35	210.26	233.37	224.73	-0.21
KLMA_20695	BET4	protein prenyltransferase	131.68	147.66	148.61	265.77	251.06	229.92	0.80
KLMA_20696	MAD2	mitotic spindle checkpoint component MAD2	30.57	35.80	40.20	53.83	66.56	57.91	0.74
KLMA_20697		hypothetical protein	32.92	43.63	35.33	74.01	70.77	53.59	0.82
KLMA_20698	VPS53	hypothetical protein	524.37	756.21	592.01	523.98	597.32	602.45	-0.12
KLMA_20699	CCT8	T-complex protein 1 subunit theta	1325.05	1642.18	1342.38	1184.21	1329.43	1286.16	-0.18
KLMA_20700	SAK1	serine/threonine-protein kinase PAK1	644.30	652.17	752.80	533.23	546.77	506.51	-0.37
KLMA_20701	CDC25	cell division control protein 25	2153.93	2144.45	2243.79	1519.79	1419.57	1337.15	-0.61
KLMA_20702	SMD2	small nuclear ribonucleoprotein Sm D2	63.49	62.64	46.29	91.68	91.83	92.49	0.68
KLMA_20703	MCM5	minichromosome maintenance protein 5	503.21	563.80	465.32	262.41	299.92	337.10	-0.77

Locus_tag	UniProt_gene	Product	Unique exon reads						log2 Fold Change
			KmWT.1	KmWT.2	KmWT.3	Kmmig1.1	Kmmig1.2	Kmmig1.3	
KLMA_20704	GAC1	CBM_21 super family	3531.89	2646.73	2922.28	1435.69	1498.77	1341.48	-1.09
KLMA_20705		hypothetical protein	8.23	20.14	7.31	10.09	1.68	1.73	-1.40
KLMA_20706	CDC34	ubiquitin-conjugating enzyme E2-34 kDa	529.08	548.14	573.74	384.36	342.05	281.78	-0.71
KLMA_20707	MDM36	mitochondrial distribution and morphology protein 36	1783.58	1871.50	1734.61	956.28	949.47	1037.22	-0.87
KLMA_20708	VMA2	v-type proton ATPase subunit B	3375.52	3506.97	3193.93	2475.24	3410.35	3263.80	-0.14
KLMA_20709	ATG14	autophagy-related protein 14	34.10	83.90	34.11	630.79	235.05	248.93	2.87
KLMA_20710		uncharacterized protein YPR084W	543.19	589.53	574.96	539.96	481.90	422.67	-0.24
KLMA_20711	ASA1	uncharacterized protein YPR085C	145.79	128.64	131.56	248.95	202.19	199.67	0.68
KLMA_20712	OPY1	protein OPY1	264.54	230.44	311.84	699.76	457.47	397.60	0.95
KLMA_20713	SHE3	uncharacterized protein YBR130C	224.56	236.04	260.68	329.69	363.11	301.66	0.46
KLMA_20714	SRP54	signal recognition particle subunit SRP54	1569.60	1359.16	1416.68	762.84	903.98	870.40	-0.78
KLMA_20715	CCZ1	vacuolar fusion protein CCZ1	202.23	206.95	187.59	283.44	250.22	318.08	0.51
KLMA_20716		ATPase	74.07	93.97	82.83	116.91	52.23	70.88	-0.06
KLMA_20717	SUI3	eukaryotic translation initiation factor 2 subunit beta	3360.23	2949.88	3158.60	2235.53	3143.28	2736.54	-0.22
KLMA_20720	YAR1	ankyrin repeat-containing protein YAR1	232.79	222.61	237.53	139.62	123.84	145.21	-0.76
KLMA_20721		uncharacterized protein YMR185W	800.67	705.87	884.36	629.95	480.21	546.27	-0.53
KLMA_20722	ERG9	squalene synthetase	2123.37	1837.94	2240.13	846.11	1014.34	1086.49	-1.07
KLMA_20723	maa	maltose O-acetyltransferase	171.66	202.48	174.19	271.66	320.98	324.13	0.74
KLMA_20724		non-structural maintenance of chromosome element 3	455.01	332.24	380.06	171.58	187.03	211.77	-1.03

Locus_tag	UniProt_gene	Product	Unique exon reads						log$_2$ Fold Change
			KmWT.1	KmWT.2	KmWT.3	Kmmig1.1	Kmmig1.2	Kmmig1.3	
KLMA_20725	NCP1	NADPH-cytochrome P450 reductase	1550.79	1342.38	1688.32	708.17	984.01	963.75	-0.79
KLMA_20726	DTD1	D-tyrosyl-tRNA(Tyr) deacylase	456.18	283.02	387.36	104.29	133.11	128.79	-1.62
KLMA_20727	DUG3	probable glutamine amidotransferase DUG3	1230.99	704.75	1270.51	836.01	428.82	482.31	-0.88
KLMA_20728		hypothetical protein	1437.92	1193.60	1283.91	635.00	1041.30	1428.78	-0.33
KLMA_20729	SRP1	importin subunit alpha	1527.27	1651.13	1607.93	864.61	907.35	1012.16	-0.78
KLMA_20730		hypothetical protein	59.96	49.22	76.74	346.52	171.02	182.38	1.91
KLMA_20731	TIM22	mitochondrial import inner membrane translocase subunit TIM22	156.37	149.90	144.96	241.38	240.95	252.39	0.70
KLMA_20732	SWT21	uncharacterized protein YNL187W	118.75	125.29	113.29	172.42	155.02	137.43	0.38
KLMA_20733	RRI1	COP9 signalosome complex subunit 5	124.63	152.14	102.32	100.09	90.99	101.99	-0.37
KLMA_20734	GDH2	NAD-specific glutamate dehydrogenase	826.54	910.58	736.97	5071.58	4134.04	3879.22	2.40
KLMA_20735	UBP10	ubiquitin carboxyl-terminal hydrolase 10	819.48	1010.14	827.11	649.30	417.87	495.27	-0.77
KLMA_20736	rplK	54S ribosomal protein L19	352.72	341.19	308.19	208.58	367.32	324.13	-0.16
KLMA_20737	NPR1	nitrogen permease reactivator protein	1414.40	1295.40	1363.08	1190.10	1246.87	1379.51	-0.09
KLMA_20738	NOP6	nucleolar protein 6	217.51	168.92	192.46	106.81	111.21	136.57	-0.71
KLMA_20739	IPI3	pre-rRNA-processing protein IPI3	748.94	663.36	874.61	277.55	327.72	369.08	-1.23
KLMA_20740		uncharacterized protein YNL181W	679.57	787.53	771.07	389.41	545.93	482.31	-0.66
KLMA_20741	ced-10	rho (Ras homology) family.	750.11	819.97	745.49	780.50	925.04	769.27	0.10
KLMA_20742	RPS3	40S ribosomal protein S3	27804.80	23430.13	27124.02	8165.84	14044.10	11677.42	-1.21
KLMA_20744	SHR3	secretory component protein SHR3	900.61	865.84	892.89	625.75	717.79	792.61	-0.32
KLMA_20745	MRPL22	54S ribosomal	1029.94	916.17	973.28	407.91	667.24	510.83	-0.88

Locus_tag	UniProt_gene	Product	Unique exon reads						log2 Fold Change
			KmWT.1	KmWT.2	KmWT.3	Kmmig1.1	Kmmig1.2	Kmmig1.3	
		protein L22							
KLMA_20746		hypothetical protein	1494.35	1105.23	1314.36	854.52	877.86	1164.28	-0.43
KLMA_20747	NOP13	nucleolar protein 13	851.23	635.39	789.35	343.15	486.11	454.65	-0.83
KLMA_20749	CRP1	uncharacterized protein YHR146W	1789.46	2289.88	1684.67	1116.08	1489.50	1432.23	-0.51
KLMA_20750	APC1	anaphase-promoting complex subunit 1	302.16	417.26	420.25	382.68	383.33	424.40	0.06
KLMA_20751	PSD1	phosphatidylserine decarboxylase proenzyme 1	1098.13	677.90	822.24	323.81	547.61	649.99	-0.77
KLMA_20752	DCD1	deoxycytidylate deaminase	767.75	420.61	585.92	392.77	404.39	497.87	-0.45
KLMA_20753	FMP41	uncharacterized mitochondrial hydrolase FMP41	457.36	440.75	444.62	497.07	668.08	703.58	0.48
KLMA_20754		CRE-binding bZIP protein SKO1	1522.57	1276.38	1599.40	1037.87	935.15	913.62	-0.61
KLMA_20755	RPC10	DNA-directed RNA polymerases I	103.46	89.49	84.05	80.74	85.93	60.50	-0.29
KLMA_20756	BNI5	bud neck protein 5	317.45	313.22	275.30	450.81	390.07	451.19	0.51
KLMA_20757		uncharacterized protein YNL165W	424.44	378.10	420.25	1328.87	824.79	883.37	1.31
KLMA_20758		protein DSE2	1504.93	1445.30	1380.14	1316.26	1293.20	1236.89	-0.17
KLMA_20759	IBD2	protein IBD2	92.88	139.83	170.54	301.10	195.45	223.87	0.84
KLMA_20760	RIA1	ribosome assembly protein 1	1163.97	1271.91	1112.15	832.65	831.53	818.54	-0.52
KLMA_20761	CHS7	chitin synthase export chaperone	726.60	675.66	741.84	959.65	860.17	927.45	0.36
KLMA_20762	RPL44	conserved hypothetical protein	9663.31	8166.15	10032.49	3074.91	6045.62	4861.12	-0.99
KLMA_20764	CBK1	serine/threonine-protein kinase CBK1	838.29	855.77	741.84	529.03	553.51	662.09	-0.48
KLMA_20765		UPF0641 membrane protein YHR140W	325.68	346.78	364.22	570.24	421.24	360.44	0.38
KLMA_20766	MEP2	ammonium transporter MEP2	85.83	130.88	87.71	640.05	673.14	718.28	2.74
KLMA_20767		uncharacterized membrane protein YLR050C	109.34	99.56	102.32	65.60	73.30	86.44	-0.47
KLMA_20768	GNA1	glucosamine 6-phosphate N-	383.29	297.56	381.27	215.31	240.95	206.58	-0.68

Locus_tag	UniProt_gene	Product	Unique exon reads						log$_2$ Fold Change
			KmWT.1	KmWT.2	KmWT.3	Kmmig1.1	Kmmig1.2	Kmmig1.3	
		acetyltransferase							
KLMA_20769		small nuclear ribonucleoprotein G	55.26	41.39	62.12	51.30	60.66	40.62	-0.05
KLMA_20770	dnaJ	dnaJ homolog 1	376.23	384.82	369.09	317.08	338.68	382.04	-0.12
KLMA_20771	HSP12	12 kDa heat shock protein	4.70	101.80	7.31	4011.01	2219.08	2556.76	6.27
KLMA_20772		hypothetical protein	135.21	124.17	132.78	232.97	167.65	152.13	0.50
KLMA_20773	IES1	ino eighty subunit 1	1287.42	1262.96	1342.38	2025.27	1693.38	1853.17	0.52
KLMA_20774	dao1	D-amino-acid oxidase	1560.19	1113.06	1466.62	768.73	792.77	772.73	-0.83
KLMA_20775	CDC4	WD40 domain	688.98	780.82	940.39	741.81	629.33	815.09	-0.14
KLMA_20776		hypothetical protein	534.96	421.73	471.41	668.64	443.99	447.74	0.13
KLMA_20777	SMC1	structural maintenance of chromosomes protein 1	734.83	804.31	640.73	465.11	449.88	509.97	-0.61
KLMA_20778	KES1	protein KES1	1383.83	1537.03	1432.52	920.12	1024.45	1110.69	-0.51
KLMA_20779	DFR1	dihydrofolate reductase	192.82	139.83	175.41	116.91	110.36	187.56	-0.29
KLMA_20780		chaperone_DMP super family	29.39	26.85	38.98	77.38	59.82	73.47	1.15
KLMA_20781	YUC8	trkA	44.68	57.05	60.91	569.40	962.11	1242.08	4.09
KLMA_20782		hypothetical protein	3.53	3.36	9.75	7.57	5.05	0.86	-0.29
KLMA_20783	RPL33B	60S ribosomal protein L33-B	7857.39	6305.83	8383.15	2650.18	5054.02	4134.20	-0.93
KLMA_20784	FRK1	putative serine/threonine-protein kinase YPL141C	736.01	900.51	831.98	492.86	619.22	724.33	-0.43
KLMA_20785	mge1	grpE protein homolog	773.63	908.34	788.13	433.99	736.33	624.93	-0.46
KLMA_20786	MKK1	MAP kinase kinase MKK2/SSP33	567.88	470.95	447.05	745.18	687.46	702.72	0.52
KLMA_20787	WTM2	transcriptional modulator WTM2	574.93	684.61	475.07	719.95	695.89	732.11	0.31
KLMA_20788	SPP1	COMPASS component SPP1	233.97	223.73	221.70	271.66	230.84	232.51	0.11
KLMA_20789		DUF1691 super family	300.99	303.15	365.44	228.77	274.65	229.92	-0.40

Locus_tag	UniProt_gene	Product	Unique exon reads						log2 Fold Change
			KmWT.1	KmWT.2	KmWT.3	Kmmig1.1	Kmmig1.2	Kmmig1.3	
KLMA_20790	GIP3	GLC7-interacting protein 3	1595.46	1334.55	1514.13	1084.97	1134.82	1046.73	-0.44
KLMA_20791	ISU1	iron sulfur cluster assembly protein 1	567.88	604.07	453.14	1259.06	1145.77	1074.39	1.10
KLMA_20792	RPB8	DNA-directed RNA polymerases I	360.95	349.02	376.40	218.68	235.05	242.88	-0.64
KLMA_20793		MTP18 super family	377.41	522.41	473.85	1365.88	1246.02	1233.43	1.48
KLMA_20794		uncharacterized membrane protein YOR223W	312.74	360.21	306.97	590.42	518.12	447.74	0.67
KLMA_20795	ODC2	mitochondrial 2-oxodicarboxylate carrier 2	2817.05	1346.86	2603.14	2733.44	5973.16	5022.76	1.02
KLMA_20796	MCT1	malonyl CoA-acyl carrier protein transacylase	222.21	215.90	287.48	365.02	429.66	437.36	0.76
KLMA_20797	RDS2	regulator of drug sensitivity 2	216.33	275.19	199.77	443.24	432.19	520.34	1.01
KLMA_20798	COX11	ctaG_Cox11 super family	447.95	399.36	393.45	218.68	230.84	198.80	-0.94
KLMA_20799		regulator of calcineurin 2	429.14	489.97	328.89	952.92	603.21	751.99	0.89
KLMA_20800	RPL5	60S ribosomal protein L5	24033.07	20570.86	23987.34	7622.51	12437.49	10993.71	-1.14
KLMA_20801	STE13	dipeptidyl aminopeptidase A	340.96	417.26	348.38	572.76	439.77	547.14	0.49
KLMA_20802		replication factor C subunit 1	737.18	802.07	663.88	538.28	669.77	586.90	-0.30
KLMA_20803	RUD3	GRIP domain-containing protein RUD3	359.77	318.82	343.51	378.48	372.37	375.99	0.14
KLMA_20804	AIM41	gatB_Yqey super family	144.61	161.09	120.59	200.17	274.65	200.53	0.66
KLMA_20805	TAF14	transcription initiation factor TFIID subunit 14	828.89	912.82	835.63	965.54	907.35	913.62	0.11
KLMA_20806	STE4	guanine nucleotide-binding protein subunit beta	368.00	316.58	314.28	1129.54	771.71	821.14	1.45
KLMA_20807	TBF1	protein TBF1	627.84	616.38	667.53	562.67	295.71	360.44	-0.65
KLMA_20808	MGM1	dynamin-like GTPase MGM1	881.80	995.60	973.28	682.10	571.20	585.17	-0.63
KLMA_20809	RPB10	DNA-directed RNA polymerases I	96.41	77.19	76.74	79.06	91.83	57.91	-0.13

Locus_tag	UniProt_gene	Product	Unique exon reads						log2 Fold Change
			KmWT.1	KmWT.2	KmWT.3	Kmmig1.1	Kmmig1.2	Kmmig1.3	
KLMA_20810	NAN1	NET1-associated nuclear protein 1	1504.93	1427.40	1651.78	672.85	750.65	745.94	-1.08
KLMA_20811	NPT1	nicotinate phosphoribosyltransferase	1617.80	1835.71	1793.08	2835.21	2454.98	2218.80	0.52
KLMA_20812	KAP120	importin beta-like protein KAP120	1014.65	1042.58	997.65	934.42	839.11	869.54	-0.21
KLMA_20813	PTP2	tyrosine-protein phosphatase 2	298.64	583.94	297.22	1672.02	890.50	1122.80	1.64
KLMA_20814	SPC29	hypothetical protein	206.93	206.95	183.94	378.48	284.76	241.15	0.60
KLMA_20815	RNY1	ribonuclease T2-like	89.36	135.36	57.25	645.93	492.85	518.61	2.55
KLMA_20816	RNY1	ribonuclease T2-like	88.18	99.56	107.20	932.73	454.94	457.24	2.65
KLMA_20817	ARG2	amino-acid acetyltransferase	573.76	374.75	594.45	604.72	418.71	463.29	-0.05
KLMA_20818	PSF2	DNA replication complex GINS protein PSF2	78.77	88.37	87.71	89.15	60.66	84.71	-0.12
KLMA_20819		hypothetical protein	12.93	16.78	7.31	636.68	308.35	395.87	5.17
KLMA_20820		hypothetical protein	323.33	328.88	344.73	451.65	417.87	434.77	0.39
KLMA_20821	APE3	aminopeptidase Y	1407.35	1664.55	1432.52	1731.74	2267.11	2224.85	0.47
KLMA_20822		dnaJ-like chaperone JEM1	262.19	262.88	257.02	543.32	373.22	394.15	0.74
KLMA_20823		uncharacterized transporter YBR287W	1254.50	1165.63	1299.74	5496.32	2239.30	2300.04	1.43
KLMA_20824	SCP1	transgelin	182.24	189.05	202.21	336.42	332.78	318.08	0.78
KLMA_20825	KHA1	K(+)/H(+) antiporter 1	1209.82	1105.23	1170.62	2950.43	2054.80	2262.88	1.06
KLMA_20826	FEN2	pantothenate transporter FEN2	87.00	104.03	129.12	465.95	353.84	382.04	1.91
KLMA_20827	amdS	acetamidase	271.59	194.65	277.73	435.67	336.99	439.96	0.71
KLMA_20828	MRS6	rab proteins geranylgeranyltransferase component A	1461.43	1398.31	1497.08	1123.65	1404.41	1451.25	-0.13
KLMA_20829	PHO3	repressible acid phosphatase	404.45	283.02	342.29	58.87	98.57	87.30	-2.07
KLMA_20830	LAC12	lactose permease	5255.51	2089.64	4645.93	1926.87	1963.81	1826.38	-1.07
KLMA_20831	BIO3	adenosylmethionine-8-amino-7-	68.19	62.64	53.60	52.15	38.75	31.12	-0.60

Locus_tag	UniProt_gene	Product	Unique exon reads						log2 Fold Change
			KmWT.1	KmWT.2	KmWT.3	Kmmig1.1	Kmmig1.2	Kmmig1.3	
KLMA_20833	BIO5	oxononanoate aminotransferase 7-keto 8-aminopelargonic acid transporter	78.77	104.03	69.43	138.77	99.41	95.94	0.40
KLMA_20834	amdS	acetamidase	7.05	32.44	14.62	140.46	71.61	60.50	2.33
KLMA_20835		uncharacterized MFS-type transporter C1271.10c	116.40	90.61	123.03	205.22	112.05	139.16	0.47
KLMA_20836		uncharacterized permease C1683.05	536.13	217.02	316.71	378.48	305.82	273.14	-0.16
KLMA_20837		FMN_red super family	122.28	57.05	176.63	474.36	251.90	255.85	1.47
KLMA_20838		hypothetical protein	0.00	0.00	0.00	1.68	0.00	3.46	2.99
KLMA_30009	LAC4	beta-galactosidase	82512.63	149686.62	101656.29	64966.73	52176.30	63630.25	-0.89
KLMA_30010	LAC12	lactose permease	116405.35	146854.20	128908.21	57212.17	33457.30	45005.16	-1.53
KLMA_30011		beta-glucosidase	1937.60	3119.92	2036.71	6856.31	6052.36	6196.55	1.43
KLMA_30012	mirA	siderophore iron transporter mirA	538.48	653.29	539.63	698.92	435.56	497.87	-0.09
KLMA_30013	CYB2	cytochrome b2	37.62	50.34	38.98	185.87	85.93	104.59	1.57
KLMA_30014		conserved hypothetical transmembrane protein	90.53	89.49	101.10	368.38	181.98	188.43	1.39
KLMA_30015	bioA	uncharacterized aminotransferase C1771.03c	89.36	72.71	91.36	617.34	363.95	441.68	2.49
KLMA_30016	YPR1	putative reductase 1	202.23	274.07	191.25	1737.63	1497.08	1687.22	2.88
KLMA_30017	PUT4	probable proline-specific permease put4	90.53	87.25	115.72	1639.22	598.16	686.30	3.32
KLMA_30018	ARI1	putative uncharacterized oxidoreductase YGL157W	111.69	78.31	90.14	396.98	152.49	174.60	1.37
KLMA_30019	fmo1	thiol-specific monooxygenase	117.57	90.61	125.47	740.97	308.35	374.27	2.09
KLMA_30024	YHB1	flavohemoprotein	1607.22	1258.48	1492.20	801.53	1126.39	822.00	-0.66

Locus_tag	UniProt_gene	Product	Unique exon reads						log2 Fold Change
			KmWT.1	KmWT.2	KmWT.3	Kmmig1.1	Kmmig1.2	Kmmig1.3	
KLMA_30025	MIR1	mitochondrial phosphate carrier protein	8064.32	7990.52	7640.09	14504.06	14387.83	12305.80	0.80
KLMA_30026		uncharacterized RNA-binding protein YKL214C	201.05	153.26	183.94	178.30	240.95	191.89	0.18
KLMA_30027	DOA1	protein DOA1	604.32	698.04	623.68	696.40	829.84	847.07	0.30
KLMA_30028	SAC1	phosphoinositide phosphatase SAC1	1979.93	2066.15	1836.93	1359.15	1941.07	1940.47	-0.17
KLMA_30029	TRP3	anthranilate synthase component 2	1182.78	799.84	1248.58	1007.59	1230.02	1287.02	0.13
KLMA_30030	CDC11	cell division control protein 11	720.72	853.53	669.97	793.96	913.25	1031.17	0.29
KLMA_30031	HOC1	putative glycosyltransferase HOC1	630.19	778.58	612.72	533.23	623.43	639.62	-0.17
KLMA_30032	MOG1	nuclear import protein MOG1	409.15	381.46	297.22	345.68	322.67	352.66	-0.09
KLMA_30033	UBA1	ubiquitin-activating enzyme E1 1	2926.39	3970.09	2845.54	3366.76	2997.53	3275.90	-0.02
KLMA_30034	STE6	alpha-factor-transporting ATPase	636.07	397.12	500.65	442.40	638.60	632.71	0.16
KLMA_30035	OPI3	methylene-fatty-acyl-phospholipid synthase	6813.35	1926.32	4553.36	1322.14	3112.95	2750.37	-0.89
KLMA_30036		UDP-N-acetylenolpyruvoylglucosamine reductase	158.72	166.68	163.23	389.41	292.34	294.74	1.00
KLMA_30037	mug157	meiotically up-regulated gene 157 protein	191.64	308.75	200.99	914.23	672.30	874.73	1.81
KLMA_30038	AIM27	ER membrane protein complex subunit 3	305.69	357.97	339.86	617.34	660.50	627.52	0.92
KLMA_30039	NPA3	GTPase NPA3	316.27	391.53	348.38	555.10	476.00	495.27	0.53
KLMA_30040	ADD66	proteasome chaperone 2	579.63	613.02	598.10	1154.77	894.71	873.00	0.71
KLMA_30041	LIA1	deoxyhypusine hydroxylase	2774.72	2380.49	2694.50	792.28	752.33	722.60	-1.79
KLMA_30042	HAM1	protein HAM1	155.20	177.87	159.57	69.81	57.29	84.71	-1.22
KLMA_30043	LOS1	exportin-T	726.60	649.94	729.66	497.07	622.59	637.89	-0.26
KLMA_30044	RFC2	replication factor C subunit 2	268.07	305.39	216.83	206.06	203.88	213.50	-0.34

Locus_tag	UniProt_gene	Product	Unique exon reads						log$_2$ Fold Change
			KmWT.1	KmWT.2	KmWT.3	Kmmig1.1	Kmmig1.2	Kmmig1.3	
KLMA_30045	YAE1	uncharacterized protein YAE1	31.74	48.10	31.67	47.10	67.40	54.45	0.60
KLMA_30046	TOR2	serine/threonine-protein kinase TOR2	1439.09	1426.28	1433.73	1048.80	1001.71	1166.01	-0.42
KLMA_30047	THI20	phosphomethylpyrimidine kinase THI20	50.56	24.61	80.40	103.45	37.91	71.74	0.46
KLMA_30048	ARP3	actin-related protein 3	2363.21	2637.78	2169.48	2185.91	2371.57	2543.79	-0.01
KLMA_30049	CCT5	T-complex protein 1 subunit epsilon	1390.89	1331.19	1196.20	704.81	665.56	886.83	-0.80
KLMA_30050		uncharacterized protein YJR061W	857.11	1320.01	956.23	629.11	478.53	567.88	-0.90
KLMA_30051	NTA1	protein N-terminal amidase	177.54	243.87	224.14	124.48	181.98	193.62	-0.37
KLMA_30052	MNN4	uncharacterized protein YJR061W	249.25	337.83	241.19	318.76	306.66	369.08	0.26
KLMA_30053	CTF18	chromosome transmission fidelity protein 18	122.28	99.56	104.76	178.30	136.48	175.46	0.59
KLMA_30055	YAP3	bZIP_1 super family	300.99	278.54	316.71	275.03	177.76	232.51	-0.39
KLMA_30056		ubiquitin-binding protein CUE2	21.16	16.78	19.49	42.89	36.23	46.68	1.13
KLMA_30057	MIF2	protein MIF2	70.54	83.90	74.31	77.38	50.55	96.81	-0.03
KLMA_30058	STE20	serine/threonine-protein kinase STE20	1236.87	1248.41	1123.11	583.69	738.85	799.53	-0.77
KLMA_30059	UFD4	ubiquitin fusion degradation protein 4	808.90	1062.72	818.58	1370.08	1221.59	1475.45	0.60
KLMA_30060	MRT4	mRNA turnover protein 4	1586.06	1484.45	1500.73	463.42	734.64	688.89	-1.28
KLMA_30061		suppressor of HU sensitivity involved in recombination	38.80	30.20	17.05	30.28	28.64	31.98	0.08
KLMA_30062	MRP4	37S ribosomal protein MRP4	736.01	844.58	836.85	349.88	588.05	545.41	-0.70
KLMA_30063	LAG1	sphingosine N-acyltransferase LAG1	3121.56	2068.38	2927.16	1792.30	1511.40	1531.63	-0.75
KLMA_30064	HSE1	class E vacuolar protein-sorting machinery protein HSE1	679.57	719.29	645.61	741.81	657.97	786.56	0.10

Locus_tag	UniProt_gene	Product	Unique exon reads						log_2 Fold Change
			KmWT.1	KmWT.2	KmWT.3	Kmmig1.1	Kmmig1.2	Kmmig1.3	
KLMA_30065	CAP1	F-actin-capping protein subunit alpha	352.72	431.80	444.62	343.15	499.59	552.32	0.18
KLMA_30066	RPL14B	60S ribosomal protein L14-B	12338.10	8802.66	11528.35	5691.44	8978.28	7915.75	-0.53
KLMA_30067	AUR1	inositol phosphorylceramide synthase	1340.33	1217.09	1469.06	2503.83	1501.29	1762.42	0.52
KLMA_30068	MRP17	37S ribosomal protein MRP17	179.89	129.76	165.67	158.96	260.33	203.12	0.39
KLMA_30069	DID4	DOA4-independent degradation protein 4	810.08	727.12	719.91	1129.54	835.74	943.87	0.37
KLMA_30070	MET14	adenylyl-sulfate kinase	2887.59	1856.96	2599.48	1034.50	1624.30	1586.95	-0.79
KLMA_30071	VPS1	vacuolar protein sorting-associated protein 1	1711.86	1830.11	1757.76	2159.84	2438.97	2497.98	0.42
KLMA_30072	PAP1	poly(A) polymerase	651.35	775.22	690.68	653.50	627.65	686.30	-0.11
KLMA_30073	MUP1	high-affinity methionine permease	7838.58	4391.82	9229.74	3293.59	2070.81	2279.30	-1.49
KLMA_30074	RSC2	chromatin structure-remodeling complex subunit RSC2	1180.43	1353.57	1321.67	443.24	684.09	697.53	-1.08
KLMA_30075	ADE13	adenylosuccinate lyase	8846.18	6532.92	8735.18	3148.92	5487.05	4979.54	-0.82
KLMA_30076	LST7	protein LST7	261.01	221.49	200.99	299.42	303.29	344.88	0.47
KLMA_30077		vacuolar protein sorting-associated protein 38	302.16	260.65	320.37	400.34	326.04	349.20	0.29
KLMA_30078	DCR2	phosphatase DCR2	731.30	640.99	601.75	661.91	544.24	644.81	-0.09
KLMA_30079		centromere DNA-binding protein complex CBF3 subunit	69.37	124.17	52.38	243.91	186.19	197.94	1.35
KLMA_30080	STE11	ste11p: Ser/Thr protein kinase	522.02	644.34	510.39	637.52	518.97	643.94	0.10
KLMA_30081	PEP5	vacuolar membrane protein PEP5	380.94	442.99	365.44	354.93	476.84	382.91	0.03
KLMA_30082	FUS2	nuclear fusion protein FUS2	7.05	2.24	9.75	23.55	23.59	25.07	1.94
KLMA_30083	RRS1	regulator of ribosome biosynthesis	748.94	705.87	767.42	264.93	366.48	418.35	-1.08

Locus_tag	UniProt_gene	Product	Unique exon reads						log2 Fold Change
			KmWT.1	KmWT.2	KmWT.3	Kmmig1.1	Kmmig1.2	Kmmig1.3	
KLMA_30085		uncharacterized protein YOR296W	400.92	520.17	453.14	810.78	636.07	656.04	0.61
KLMA_30086	mug14	meiotically up-regulated gene 14 protein	99.94	120.81	92.58	627.43	376.59	396.74	2.16
KLMA_30087	RNH1	ribonuclease H	17.64	7.83	21.93	77.38	42.12	39.76	1.76
KLMA_30088	FAA1	long-chain-fatty-acid--CoA ligase 1	4102.12	4857.18	4234.21	2479.44	2622.63	2669.99	-0.76
KLMA_30089	CLD1	uncharacterized protein YGR110W	1247.45	1004.55	1281.47	616.50	807.09	825.46	-0.65
KLMA_30090	MUB1	MYND-type zinc finger protein MUB1	343.31	357.97	330.11	334.74	268.75	307.71	-0.18
KLMA_30091	VPH2	vacuolar ATPase assembly integral membrane protein VPH2	112.87	124.17	99.89	215.31	141.54	196.21	0.71
KLMA_30092	SRT1	putative dehydrodolichyl diphosphate synthetase	31.74	14.54	45.07	58.03	39.60	56.18	0.76
KLMA_30093	OAC1	mitochondrial oxaloacetate transport protein	945.29	602.95	990.34	965.54	686.62	611.10	-0.17
KLMA_30094		WD repeat-containing protein YMR102C	647.83	713.70	565.21	745.18	614.17	588.62	0.02
KLMA_30095		signal recognition particle subunit SRP21	176.36	171.15	144.96	550.05	421.24	424.40	1.50
KLMA_30096	SSH4	protein SSH4	799.50	777.46	726.00	1259.06	1032.88	1209.23	0.60
KLMA_30097	RRN3	RNA polymerase I-specific transcription initiation factor RRN3	389.17	362.44	405.64	685.46	347.94	409.70	0.32
KLMA_30098	YPK1	serine/threonine-protein kinase YPK1	1787.11	1751.81	1618.89	1932.75	1870.30	2311.28	0.25
KLMA_30099	PGM2	phosphoglucomutase -2	10345.24	9703.17	10172.57	1570.26	1525.73	1963.81	-2.58
KLMA_30100	YKU80	ATP-dependent DNA helicase II subunit 2	92.88	108.51	105.98	378.48	211.46	267.09	1.48
KLMA_30101	SPG4	uncharacterized protein YMR107W	36.45	21.25	19.49	1505.49	1117.12	1112.42	5.60

Locus_tag	UniProt_gene	Product	Unique exon reads						log2 Fold Change
			KmWT.1	KmWT.2	KmWT.3	Kmmig1.1	Kmmig1.2	Kmmig1.3	
KLMA_30102	PMU1	uncharacterized protein YKL128C	342.14	242.75	263.12	145.50	86.78	120.15	-1.27
KLMA_30103	dur3-1	hypothetical protein	69.37	50.34	64.56	75.70	31.17	63.96	-0.11
KLMA_30105	ILV2	acetolactate synthase	1963.47	1283.09	1942.91	1252.34	1103.65	1321.60	-0.50
KLMA_30106	MYO1	myosin-1	1060.51	1503.47	1162.09	981.52	1016.03	1127.12	-0.25
KLMA_30107	HFD1	putative fatty aldehyde dehydrogenase HFD1	610.20	827.80	559.12	769.57	729.59	779.65	0.19
KLMA_30108	SHE2	uncharacterized protein YKL130C	148.14	177.87	160.79	161.48	209.78	155.58	0.11
KLMA_30109		uncharacterized protein YMR111C	231.62	257.29	229.01	243.91	225.78	242.88	-0.01
KLMA_30110	MED11	mediator of RNA polymerase II transcription subunit 11	141.09	157.73	153.48	108.50	107.84	101.99	-0.51
KLMA_30111	FOL3	folylpolyglutamate synthase	199.87	290.85	285.04	285.96	220.73	261.03	-0.02
KLMA_30112		uncharacterized protein YMR114C	55.26	135.36	85.27	468.47	358.05	377.72	2.12
KLMA_30113	MGR3	mitochondrial inner membrane i-AAA protease supercomplex subunit MGR3	442.07	633.16	525.01	593.79	600.69	616.28	0.18
KLMA_30114	Oct-01	mitochondrial intermediate peptidase	340.96	397.12	345.95	289.32	316.77	261.03	-0.32
KLMA_30115	ASC1	guanine nucleotide-binding protein subunit beta-like	41598.45	32390.53	38318.60	16984.34	28573.46	23226.91	-0.71
KLMA_30117	APL2	AP-1 complex subunit beta-1	1461.43	1794.32	1400.85	1203.55	1331.11	1430.51	-0.23
KLMA_30119	CMC1	COX assembly mitochondrial protein	139.91	105.15	96.23	31.96	58.97	74.33	-1.05
KLMA_30120	MRPL31	54S ribosomal protein L31	348.02	344.54	325.24	201.01	308.35	244.61	-0.43
KLMA_30121	SPC24	probable kinetochore protein SPC24	88.18	121.93	76.74	183.35	176.92	204.85	0.98
KLMA_30122		DASH complex subunit HSK3	27.04	20.14	36.54	41.21	48.02	37.17	0.60

Locus_tag	UniProt_gene	Product	Unique exon reads						log$_2$ Fold Change
			KmWT.1	KmWT.2	KmWT.3	Kmmig1.1	Kmmig1.2	Kmmig1.3	
KLMA_30123	CTK1	CTD kinase subunit alpha	198.70	276.31	298.44	333.90	262.01	309.44	0.23
KLMA_30124		succinate dehydrogenase [ubiquinone] cytochrome b subunit	2504.30	2942.05	2570.25	6471.95	5236.00	4536.13	1.02
KLMA_30125	VTC2	vacuolar transporter chaperone 2	1067.56	1226.04	1305.83	1494.56	1116.28	1252.45	0.10
KLMA_30127	SEC4	ras-related protein SEC4	797.14	686.85	736.97	596.31	799.51	923.13	0.06
KLMA_30128	BLM10	proteasome activator BLM10	1174.55	1447.53	1236.40	1148.89	1171.89	1210.96	-0.13
KLMA_30129		hypothetical protein	135.21	316.58	159.57	1981.53	1096.91	1299.99	2.84
KLMA_30130	CFT2	cleavage factor two protein 2	476.17	559.33	518.92	867.97	679.04	671.60	0.51
KLMA_30133	RRG1	uncharacterized protein YDR065W	148.14	172.27	143.74	529.03	282.23	337.96	1.31
KLMA_30134	RTR1	RNA polymerase II-associated protein 2 homolog	77.60	92.85	73.09	359.13	255.27	235.97	1.80
KLMA_30135		protein TAPT1 homolog	369.18	457.53	427.56	784.71	534.13	650.86	0.65
KLMA_30136	gabD	putative succinate-semialdehyde dehydrogenase C1002.12c [NADP+]	335.08	449.70	314.28	349.88	367.32	375.99	-0.01
KLMA_30137	mlo2	protein mlo2	225.74	289.73	199.77	1317.10	873.65	930.04	2.12
KLMA_30138	MCM2	DNA replication licensing factor MCM2	386.81	416.14	403.20	571.92	456.62	552.32	0.39
KLMA_30139	NCL1	tRNA (cytosine-5-)-methyltransferase NCL1	2255.05	1868.15	2139.03	991.61	1166.83	1164.28	-0.91
KLMA_30140	RRN10	factor RRN10	37.62	14.54	32.89	15.14	30.33	19.02	-0.40
KLMA_30141	EMP24	endosomal protein P24B	1256.85	1029.16	1071.95	932.73	1122.18	1214.42	-0.04
KLMA_30142	GLC7	serine/threonine-protein phosphatase PP1-2	1753.01	1650.01	1769.94	1090.01	1312.58	1384.69	-0.45
KLMA_30143	YIP4	protein YIP4	435.02	479.90	470.20	523.98	393.44	471.07	0.00

Locus_tag	UniProt_gene	Product	Unique exon reads						log2 Fold Change
			KmWT.1	KmWT.2	KmWT.3	Kmmig1.1	Kmmig1.2	Kmmig1.3	
KLMA_30144	MDS3	negative regulator of sporulation MDS3	663.11	819.97	749.15	560.15	495.38	531.58	-0.49
KLMA_30145	GCN1	translational activator GCN1	3388.45	2722.80	3162.26	1312.05	2085.97	2207.56	-0.73
KLMA_30146	SGF29	SAGA-associated factor 29	103.46	74.95	110.85	142.98	166.81	119.28	0.57
KLMA_30147	MRPL2	60S ribosomal protein L2	780.68	940.79	829.54	398.66	555.19	608.50	-0.71
KLMA_30148	ILV6	acetolactate synthase small subunit	5309.59	3143.41	4940.72	2762.04	3620.97	3936.27	-0.38
KLMA_30149	IDP2	isocitrate dehydrogenase [NADP] cytoplasmic	3323.78	3445.44	3315.74	2443.28	4065.80	3987.26	0.06
KLMA_30150		uncharacterized phosphatase YNL010W	478.52	361.32	427.56	507.16	381.64	473.67	0.10
KLMA_30151	SSP120	protein SSP120	176.36	155.49	147.39	328.85	402.70	478.85	1.34
KLMA_30152	SPO1	putative meiotic phospholipase SPO1	12.93	23.49	9.75	148.87	80.88	95.08	2.81
KLMA_30153	TEF3	elongation factor 3	83773.01	51613.42	70685.43	18369.56	29947.54	28273.01	-1.43
KLMA_30154	GSH1	glutamate--cysteine ligase	2869.95	1930.79	2978.32	1061.42	1138.19	1266.28	-1.17
KLMA_30155	LSB6	phosphatidylinositol 4-kinase LSB6	875.92	730.48	920.90	774.62	532.45	652.59	-0.37
KLMA_30156	CHS6	chitin biosynthesis protein CHS6	649.00	792.00	743.06	561.83	695.89	719.14	-0.14
KLMA_30157	SAP190	SIT4-associating protein SAP190	1555.49	1539.26	1520.22	899.93	1089.32	1223.06	-0.52
KLMA_30158	PHS1	protein PHS1	491.45	574.99	540.85	215.31	306.66	281.78	-1.00
KLMA_30159	MRPL49	54S ribosomal protein L49	444.43	383.70	466.54	326.33	453.25	411.43	-0.12
KLMA_30160	BCK1	serine/threonine-protein kinase BCK1/SLK1/SSP31	476.17	645.46	535.98	866.29	652.92	776.19	0.47
KLMA_30161	TOK1	outward-rectifier potassium channel TOK1	191.64	152.14	203.43	753.59	439.77	521.21	1.65
KLMA_30162	SRS2	ATP-dependent DNA helicase SRS2	203.40	220.37	204.65	379.32	256.96	310.30	0.59
KLMA_30163	SRS2	ATP-dependent DNA helicase SRS2	157.55	157.73	199.77	411.28	300.76	319.81	1.00
KLMA_30164	GWT1	GPI-anchored wall transfer protein 1	1079.32	663.36	1138.95	407.91	556.88	593.81	-0.89

Locus_tag	UniProt_gene	Product	Unique exon reads						log$_2$ Fold Change
			KmWT.1	KmWT.2	KmWT.3	Kmmig1.1	Kmmig1.2	Kmmig1.3	
KLMA_30165	DPB11	DNA replication regulator DPB11	197.52	199.12	266.77	181.67	141.54	155.58	-0.47
KLMA_30166	SIP4	protein SIP4	221.04	212.54	222.92	1970.60	738.85	857.44	2.44
KLMA_30167		hypothetical protein	62.31	38.03	79.18	58.03	69.08	72.61	0.16
KLMA_30168	ARG3	ornithine carbamoyltransferas e	2163.34	1534.79	2187.75	1234.67	1534.15	1818.60	-0.36
KLMA_30170	TRL1	tRNA ligase	259.84	255.05	333.77	327.17	310.87	297.34	0.14
KLMA_30171	EXO70	exocyst complex protein EXO70	437.37	419.49	410.51	659.39	672.30	607.64	0.61
KLMA_30172	ALY2	UPF0675 protein YJL084C	898.26	962.04	1017.14	828.44	804.57	882.50	-0.19
KLMA_30173	RAD4	DNA repair protein RAD4	233.97	222.61	261.90	418.85	315.93	276.59	0.49
KLMA_30174		protein SPT2	155.20	158.85	152.27	120.27	143.22	133.11	-0.23
KLMA_30175		uncharacterized protein YDR089W	464.41	400.48	479.94	626.59	402.70	495.27	0.18
KLMA_30176	SLU7	pre-mRNA-splicing factor SLU7	136.38	119.70	136.43	105.97	58.97	108.91	-0.52
KLMA_30177	PUS6	pseudouridylate synthase 6	273.94	265.12	310.62	238.86	228.31	233.38	-0.28
KLMA_30178	BBC1	myosin tail region-interacting protein MTI1	760.70	996.72	783.26	708.17	763.28	804.71	-0.16
KLMA_30179		uncharacterized protein YGR168C	163.43	151.02	147.39	288.48	320.14	307.71	0.99
KLMA_30180	CLC1	clathrin light chain	777.16	964.28	734.53	759.48	1030.35	1025.99	0.19
KLMA_30181		beta-glucan synthesis-associated protein KRE11	168.13	162.20	163.23	238.86	250.22	280.91	0.64
KLMA_30182	SIR2	NAD-dependent histone deacetylase SIR2	1904.68	1813.33	2007.47	1561.00	1401.88	1544.60	-0.34
KLMA_30183	RTG1	retrograde regulation protein 1	30.57	39.15	24.36	95.04	48.02	66.56	1.15
KLMA_30184	RPT2	26S protease regulatory subunit 4 homolog	1073.44	1332.31	973.28	2194.32	1997.51	2119.39	0.90
KLMA_30185	PTC1	protein phosphatase 2C homolog 1	290.40	385.93	306.97	622.38	531.60	629.25	0.86
KLMA_30186	MED2	mediator of RNA polymerase II	835.94	613.02	678.50	932.73	778.45	665.55	0.16

Locus_tag	UniProt_gene	Product	Unique exon reads						log2 Fold Change
			KmWT.1	KmWT.2	KmWT.3	Kmmig1.1	Kmmig1.2	Kmmig1.3	
		transcription subunit 2							
KLMA_30187	ATP16	ATP synthase subunit delta	2503.13	2556.12	2543.45	2249.83	2924.24	2224.85	-0.04
KLMA_30188	MCD1	sister chromatid cohesion protein 1	911.19	1013.50	1039.06	518.09	612.48	637.03	-0.75
KLMA_30189		uncharacterized protein YDR333C	299.81	266.24	269.21	193.44	197.98	214.36	-0.46
KLMA_30190	IPI1	pre-rRNA-processing protein IPI1	225.74	185.70	257.02	78.22	47.18	73.47	-1.75
KLMA_30191	NAM8	protein NAM8	806.55	553.73	762.55	2295.25	1034.56	926.59	1.00
KLMA_30192		protein PET130	108.17	86.14	93.80	322.97	286.44	286.97	1.64
KLMA_30193		uncharacterized protein YGR169C-A	88.18	79.42	96.23	139.62	110.36	89.03	0.36
KLMA_30194	PSD2	phosphatidylserine decarboxylase proenzyme 2	728.95	696.92	623.68	961.33	671.45	898.93	0.30
KLMA_30195	POL31	DNA polymerase delta small subunit	202.23	190.17	174.19	223.72	227.47	234.24	0.27
KLMA_30196	MSM1	methionyl-tRNA synthetase	339.79	375.87	336.20	269.98	288.13	223.87	-0.43
KLMA_30197	RBG2	uncharacterized GTP-binding protein YGR173W	519.67	491.09	517.70	308.67	413.66	407.11	-0.44
KLMA_30198	YIP1	protein transport protein YIP1	1046.40	767.39	953.79	544.16	756.54	758.90	-0.43
KLMA_30199	RNR2	ribonucleoside-diphosphate reductase small chain 1	7116.68	5834.88	6256.30	2101.81	1968.03	2085.69	-1.64
KLMA_30200	OKP1	central kinetochore subunit OKP1	117.57	138.71	155.92	310.35	264.54	260.17	1.02
KLMA_30201	PBP1	PAB1-binding protein 1	1278.02	1518.01	1386.23	1291.87	1285.62	1413.22	-0.07
KLMA_30202	RRN7	RNA polymerase I-specific transcription initiation factor RRN7	176.36	178.98	205.86	191.76	140.69	159.91	-0.19
KLMA_30203	ATF1	alcohol O-acetyltransferase 1	1018.18	821.09	1309.49	438.19	443.99	508.24	-1.18
KLMA_30204	ERG1	squalene monooxygenase	5518.87	3585.27	5700.83	535.75	989.07	1034.63	-2.53

Locus_tag	UniProt_gene	Product	Unique exon reads						log$_2$ Fold Change
			KmWT.1	KmWT.2	KmWT.3	Kmmig1.1	Kmmig1.2	Kmmig1.3	
KLMA_30205	CBP4	assembly factor CBP4	196.35	199.12	215.61	153.07	189.56	187.56	-0.20
KLMA_30206	APS3	AP-3 complex subunit sigma	285.70	297.56	285.04	293.53	375.74	354.38	0.24
KLMA_30207	TIM13	mitochondrial import inner membrane translocase subunit TIM13	144.61	138.71	140.08	117.75	208.09	164.23	0.21
KLMA_30208	QCR9	c reductase complex	951.16	734.95	945.27	559.30	861.85	690.62	-0.32
KLMA_30209	UBR1	E3 ubiquitin-protein ligase UBR1	1408.52	1850.25	1450.79	1555.12	1182.00	1330.24	-0.21
KLMA_30210	UBR2	E3 ubiquitin-protein ligase UBR2	469.12	653.29	515.27	961.33	725.37	809.03	0.61
KLMA_30211	TYS1	tyrosyl-tRNA synthetase	2078.69	1929.67	1931.95	952.08	1288.99	1357.04	-0.72
KLMA_30212	TFG1	transcription initiation factor IIF subunit alpha	832.42	911.70	754.02	672.85	642.81	598.13	-0.38
KLMA_30214	HGH1	protein HGH1	768.93	660.00	797.87	400.34	477.68	433.04	-0.76
KLMA_30216		uncharacterized protein YLL014W	216.33	171.15	209.52	114.38	203.04	140.89	-0.38
KLMA_30217	PUF3	mRNA-binding protein PUF3	1013.48	1023.57	992.77	779.66	755.70	736.43	-0.42
KLMA_30218	IZH3	ADIPOR-like receptor IZH3	2309.13	2298.83	2684.75	4365.94	2759.96	2490.20	0.40
KLMA_30219	SDO1	ribosome maturation protein SDO1	232.79	233.80	288.70	223.72	264.54	299.07	0.06
KLMA_30220	IRC25	proteasome chaperone 3	122.28	132.00	143.74	196.81	180.29	204.85	0.55
KLMA_30221	YEH2	sterol esterase 2	634.89	616.38	689.46	471.83	489.48	583.44	-0.33
KLMA_30222	DCAF13	protein SOF1	681.92	623.09	688.24	282.60	326.04	298.20	-1.14
KLMA_30223	PSR1	phosphatase PSR1	1611.92	1532.55	1727.30	1444.10	1263.72	1339.75	-0.27
KLMA_30224	POM34	nucleoporin POM34	691.33	638.75	624.90	778.82	707.68	641.35	0.12
KLMA_30225	MEU1	multicopy enhancer of UAS2	2389.08	1637.70	2040.36	826.76	1117.12	1078.71	-1.01
KLMA_30226		cytochrome c oxidase copper chaperone	156.37	147.66	109.63	51.30	59.82	70.88	-1.19
KLMA_30227	CYR1	adenylate cyclase	1357.97	1495.64	1588.44	1344.85	1116.28	1153.05	-0.30
KLMA_30228	SYS1	protein SYS1	181.06	212.54	160.79	209.42	317.61	254.12	0.49

Locus_tag	UniProt_gene	Product	Unique exon reads						log_2 Fold Change
			KmWT.1	KmWT.2	KmWT.3	Kmmig1.1	Kmmig1.2	Kmmig1.3	
KLMA_30229	DRS1	ATP-dependent RNA helicase DRS1	1098.13	870.31	1048.81	407.07	517.28	508.24	-1.07
KLMA_30230		uncharacterized protein YLL007C	881.80	815.50	839.29	661.07	649.55	694.08	-0.34
KLMA_30231	MMM1	maintenance of mitochondrial morphology protein 1	101.11	101.80	108.41	259.89	190.40	186.70	1.03
KLMA_30232	SPO75	sporulation-specific protein 75	159.90	185.70	193.68	341.47	302.45	267.95	0.76
KLMA_30233	PMT2	dolichyl-phosphate-mannose--protein mannosyltransferase 2	7719.83	5536.20	6579.10	3774.67	5301.71	4988.18	-0.50
KLMA_30234	FUN26	nucleoside transporter FUN26	624.31	539.19	577.39	562.67	500.43	519.48	-0.14
KLMA_30235	CCR4	glucose-repressible alcohol dehydrogenase transcriptional effector	2498.42	2410.69	2336.37	1067.30	1359.76	1408.90	-0.92
KLMA_30236	ATS1	protein ATS1	407.98	465.36	370.31	288.48	262.01	335.37	-0.49
KLMA_30237	MTH1	protein MTH1	5028.59	6190.61	4866.41	2271.70	2053.12	1712.28	-1.41
KLMA_30238	DBP5	ATP-dependent RNA helicase DBP5	938.23	866.95	1056.12	661.07	767.50	751.12	-0.39
KLMA_30239		plasma membrane proteolipid 3	122.28	155.49	116.94	95.88	188.71	77.79	-0.12
KLMA_30240		mitochondrial import receptor subunit TOM6	47.03	58.17	47.51	71.49	79.19	74.33	0.56
KLMA_30241	WHI2	growth regulation protein	1219.23	1139.90	1388.66	1353.26	1054.78	1145.27	-0.08
KLMA_30242	CUE5	ubiquitin-binding protein CUE5	319.80	569.39	382.49	972.26	810.46	866.08	1.06
KLMA_30243	GLO4	hydroxyacylglutathi one hydrolase	276.30	336.71	275.30	2135.45	1319.32	1471.99	2.47
KLMA_30244		WD40 super family protein	146.97	149.90	114.50	837.69	438.09	475.39	2.09
KLMA_30245		protein midA homolog	58.79	93.97	73.09	337.26	285.60	260.17	1.97
KLMA_30246	RSB1	sphingoid long-chain base transporter RSB1	723.07	512.34	752.80	1266.63	974.75	1064.88	0.73

Locus_tag	UniProt_gene	Product	Unique exon reads						log$_2$ Fold Change
			KmWT.1	KmWT.2	KmWT.3	Kmmig1.1	Kmmig1.2	Kmmig1.3	
KLMA_30247	GPT2	glycerol-3-phosphate O-acyltransferase 2	1303.88	1249.53	1450.79	1084.97	900.61	968.08	-0.44
KLMA_30248	BET3	transport protein particle 22 kDa subunit	87.00	125.29	80.40	89.99	139.85	130.52	0.30
KLMA_30249	ETT1	uncharacterized protein YOR051C	2144.53	1787.60	2035.49	317.08	459.15	522.93	-2.20
KLMA_30250	MET1	uroporphyrinogen-III C-methyltransferase	1330.92	821.09	1617.67	397.82	712.74	898.06	-0.91
KLMA_30251		uncharacterized protein YKR070W	139.91	120.81	142.52	206.90	202.19	224.73	0.65
KLMA_30252		AN1-type zinc finger protein YOR052C	697.21	684.61	641.95	1460.92	1068.26	1102.92	0.84
KLMA_30253	DRE2	protein DRE2	1179.26	991.12	1201.07	829.28	669.77	616.28	-0.67
KLMA_30254	VHS3	protein VHS3	979.38	967.63	935.52	444.92	441.46	530.71	-1.02
KLMA_30255	NOB1	20S-pre-rRNA D-site endonuclease NOB1	455.01	508.99	415.38	181.67	155.86	196.21	-1.37
KLMA_30257	SGT1	protein SGT1	118.75	90.61	109.63	179.15	95.20	150.40	0.41
KLMA_30258	ASE1	anaphase spindle elongation protein	239.85	220.37	269.21	490.34	625.96	512.56	1.16
KLMA_30259	AIM29	altered inheritance rate of mitochondria protein 29	192.82	178.98	218.04	209.42	223.26	226.46	0.16
KLMA_30260		putative lipase YOR059C	363.30	374.75	333.77	691.35	735.48	821.14	1.07
KLMA_30261	SLD7	mitochondrial morphogenesis protein YOR060C	185.77	178.98	191.25	139.62	181.13	151.26	-0.24
KLMA_30262	CKA2	casein kinase II subunit alpha'	633.72	503.39	582.26	582.01	780.98	867.81	0.38
KLMA_30263		uncharacterized protein YKR075C	6458.28	6092.17	6259.95	1788.93	1528.25	1551.51	-1.95
KLMA_30264	RPL3	60S ribosomal protein L3	47105.56	35540.65	42264.11	13239.10	20994.53	17931.01	-1.26
KLMA_30266	YNG1	protein YNG1	71.72	74.95	98.67	84.11	74.14	80.38	-0.04
KLMA_30267	CYT1	cytochrome c1	7184.88	5512.71	6800.80	6213.74	6584.80	5346.03	-0.10
KLMA_30268		g1-specific transcription factors activator MSA1	498.51	493.32	528.67	458.38	529.08	560.97	0.03

Locus_tag	UniProt_gene	Product	Unique exon reads						log$_2$ Fold Change
			KmWT.1	KmWT.2	KmWT.3	Kmmig1.1	Kmmig1.2	Kmmig1.3	
KLMA_30269	ALG8	dolichyl pyrophosphate Glc1Man9GlcNAc2 alpha-1	1100.48	965.40	1029.32	1534.09	1634.41	1448.66	0.58
KLMA_30270	VPS5	vacuolar protein sorting-associated protein 5	760.70	710.34	751.58	843.58	1053.94	1018.21	0.39
KLMA_30271	TRZ1	ribonuclease Z	440.90	444.10	510.39	306.99	329.41	337.96	-0.52
KLMA_30272		DUF676 super family[cl10636]	188.12	124.17	174.19	402.87	363.11	426.99	1.30
KLMA_30273		TRAPP-associated protein TCA17	84.65	52.58	58.47	115.23	135.64	121.87	0.93
KLMA_30274		fumarate reductase	8050.21	6667.16	7927.57	1521.48	1919.16	2150.51	-2.02
KLMA_30275	GLY1	low specificity L-threonine aldolase	4575.94	3195.98	4554.57	2081.62	2194.65	1918.86	-0.99
KLMA_30276	SIT1	siderophore iron transporter 1	657.23	434.04	600.54	608.09	449.04	510.83	-0.11
KLMA_30278	IES6	chromatin-remodeling complex subunit IES6	172.83	191.29	185.16	273.34	270.44	208.31	0.45
KLMA_30279		uncharacterized protein YEL043W	464.41	477.66	412.94	592.95	475.16	497.00	0.21
KLMA_30280	ISY1	pre-mRNA-splicing factor ISY1	215.16	190.17	219.26	459.22	272.12	343.15	0.78
KLMA_30281	GDA1	guanosine-diphosphatase	560.82	552.61	512.83	449.13	591.42	583.44	0.00
KLMA_30282		NAD(+) kinase	276.30	294.21	266.77	676.21	538.34	643.94	1.15
KLMA_30283	UTR2	probable glycosidase CRH2	663.11	544.78	678.50	592.95	669.77	701.86	0.06
KLMA_30284	CYCK	cytochrome c	2471.38	2097.47	2884.52	1439.05	2256.15	1613.75	-0.49
KLMA_30285	TMT1	trans-aconitate 3-methyltransferase	70.54	69.36	75.52	71.49	44.65	48.40	-0.39
KLMA_30286	GRX3	monothiol glutaredoxin-3	1132.23	1007.90	980.59	1517.27	1221.59	1130.57	0.31
KLMA_30288	RAD24	checkpoint protein RAD24	246.90	269.59	272.86	291.01	233.37	280.05	0.03
KLMA_30289	BRR2	pre-mRNA-splicing helicase BRR2	1116.94	1039.23	1194.98	848.63	753.17	728.65	-0.52
KLMA_30290	RAD3	DNA repair helicase RAD3	659.58	655.53	644.39	373.43	476.84	459.84	-0.58
KLMA_30292	ADK2	adenylate kinase 2	435.02	449.70	476.29	208.58	327.72	344.01	-0.63

Locus_tag	UniProt_gene	Product	Unique exon reads						log$_2$ Fold Change
			KmWT.1	KmWT.2	KmWT.3	Kmmig1.1	Kmmig1.2	Kmmig1.3	
KLMA_30293	MSH6	DNA mismatch repair protein MSH6	853.58	858.00	861.22	508.00	507.17	471.07	-0.79
KLMA_30294	RPH1	DNA damage-responsive transcriptional repressor RPH1	932.35	1258.48	1160.87	1110.20	835.74	859.17	-0.26
KLMA_30295	CCA1	tRNA nucleotidyltransferase	542.01	598.48	598.10	415.48	389.22	466.75	-0.45
KLMA_30296		protein BCK2	1981.10	1796.55	1873.48	962.17	1056.47	990.55	-0.91
KLMA_30297	DNF1	probable phospholipid-transporting ATPase DNF1	1622.51	1646.65	1728.52	1558.48	1453.27	1580.90	-0.12
KLMA_30298	UBC13	ubiquitin-conjugating enzyme E2 13	144.61	259.53	169.32	293.53	233.37	273.14	0.48
KLMA_30299	PAB1	polyadenylate-binding protein	5863.36	5073.08	5257.43	2012.65	2925.92	2952.63	-1.04
KLMA_30300	CHD1	chromo domain-containing protein 1	1075.79	1233.87	1203.51	560.99	605.74	653.45	-0.95
KLMA_30301	RLI1	translation initiation factor RLI1	6087.92	3779.92	5703.27	1384.38	3069.99	2870.52	-1.09
KLMA_30302	GPI13	GPI ethanolamine phosphate transferase 3	522.02	529.12	578.61	475.20	638.60	635.30	0.10
KLMA_30303	MCM4	cell division control protein 54	460.89	413.90	414.16	359.13	412.81	433.04	-0.10
KLMA_30305	DUR3	urea active transporter	285.70	324.41	339.86	417.17	297.39	374.27	0.20
KLMA_30306	ATP20	ATP synthase subunit g	677.22	635.39	657.79	360.81	708.52	582.57	-0.25
KLMA_30307	ROK1	ATP-dependent RNA helicase ROK1	605.50	640.99	667.53	298.58	331.94	340.56	-0.98
KLMA_30308	MRPL15	54S ribosomal protein L15	1002.90	1017.97	1013.48	329.69	613.32	545.41	-1.03
KLMA_30309	SPA2	protein SPA2	1042.87	1003.43	1009.83	597.99	806.25	808.17	-0.47
KLMA_30310	MCH2	probable transporter MCH2	1122.82	745.02	1054.90	881.43	652.08	665.55	-0.41
KLMA_30312	ACO2	aconitate hydratase	15237.44	10347.52	11655.03	17568.03	35698.29	29327.52	1.15
KLMA_30314	STT4	phosphatidylinositol 4-kinase STT4	472.64	445.22	537.19	359.13	389.22	350.06	-0.41
KLMA_30315		hypothetical protein	172.83	149.90	199.77	232.97	185.34	216.09	0.28

Locus_tag	UniProt_gene	Product	Unique exon reads						log2 Fold Change
			KmWT.1	KmWT.2	KmWT.3	Kmmig1.1	Kmmig1.2	Kmmig1.3	
KLMA_30316	UBC12	NEDD8-conjugating enzyme UBC12	29.39	30.20	29.24	81.58	50.55	78.66	1.25
KLMA_30317		hypothetical protein	328.03	243.87	315.49	111.02	81.72	88.16	-1.66
KLMA_30318	CDA2	chitin deacetylase 2	56.43	63.76	36.54	40.37	32.01	24.20	-0.70
KLMA_30319	IMH1	Golgin IMH1	1049.93	987.77	996.43	809.94	844.16	863.49	-0.27
KLMA_30320		glyco_transf_15 super family protein	1949.36	2057.20	2030.62	952.92	884.60	973.26	-1.10
KLMA_30321	ATG1	serine/threonine-protein kinase ATG1	753.64	922.89	844.16	2053.87	1519.83	1725.25	1.07
KLMA_30322	MND1	meiotic nuclear division protein 1	3.53	0.00	0.00	31.96	20.22	23.34	4.43
KLMA_30323		zinc finger protein YER130C	775.98	843.46	763.77	5523.23	2317.65	2485.88	2.12
KLMA_30324		GAL4-like Zn2Cys6 binuclear cluster DNA-binding domain; found in transcription regulators like GAL4	997.02	931.84	1012.26	2023.59	990.75	1113.29	0.49
KLMA_30325	STR3	cystathionine beta-lyase	692.50	899.40	817.36	1532.41	753.17	1082.17	0.48
KLMA_30326	PIB2	phosphatidylinositol-3-phosphate-binding protein 2	2008.14	1940.86	1852.77	1106.83	926.72	866.95	-1.00
KLMA_30327	SCT1	glycerol-3-phosphate O-acyltransferase 1	2122.19	2175.78	2202.37	1492.88	1310.05	1316.41	-0.66
KLMA_30328	PPQ1	serine/threonine-protein phosphatase PPQ	1775.35	1905.06	1784.56	1640.91	1409.46	1369.14	-0.31
KLMA_30329	CBC2	nuclear cap-binding protein subunit 2	406.80	416.14	436.09	285.96	471.79	400.20	-0.12
KLMA_30330	CUP9	homeodomain super family	1582.53	1378.18	1358.21	880.59	889.66	880.78	-0.70
KLMA_30331		hypothetical protein	31.74	26.85	23.14	194.28	90.15	111.50	2.28
KLMA_30332		sporulation protein 23	162.25	139.83	155.92	1845.28	1230.02	1503.98	3.32
KLMA_30333	HFI1	transcriptional coactivator HFI1/ADA1	445.60	478.78	489.69	235.50	296.55	282.64	-0.80
KLMA_30334	CIK1	spindle pole body-associated protein CIK1	309.22	324.41	334.98	229.61	297.39	309.44	-0.21

Locus_tag	UniProt_gene	Product	Unique exon reads						log2 Fold Change
			KmWT.1	KmWT.2	KmWT.3	Kmmig1.1	Kmmig1.2	Kmmig1.3	
KLMA_30335	YAH1	adrenodoxin homolog	210.46	234.92	224.14	166.53	256.11	219.55	-0.06
KLMA_30336	VTI1	t-SNARE VTI1	519.67	516.82	518.92	1185.89	946.94	1031.17	1.02
KLMA_30337		uncharacterized protein YMR196W	1079.32	1096.28	1009.83	1799.87	1422.94	1670.80	0.62
KLMA_30338		protein ICY2	393.87	283.02	406.85	372.59	347.94	308.57	-0.07
KLMA_30339		ATP synthase subunit b	7.05	7.83	10.96	25.23	5.90	19.02	0.96
KLMA_30340	SWA2	auxilin-like clathrin uncoating factor SWA2	550.24	553.73	587.14	378.48	369.01	377.72	-0.59
KLMA_30341	ERG7	lanosterol synthase	942.93	781.94	964.76	406.23	512.23	577.39	-0.85
KLMA_30342	YFT2	FIT family protein YFT2	291.58	168.92	299.66	190.92	179.45	210.90	-0.39
KLMA_30343		central kinetochore subunit MCM21	129.33	172.27	159.57	661.91	528.23	491.82	1.87
KLMA_30344		protein HIM1	195.17	267.36	227.79	3227.98	1917.48	2340.67	3.44
KLMA_30345		hypothetical protein	2420.83	1657.84	2026.96	1698.94	1536.68	1536.82	-0.36
KLMA_30346	CWC21	pre-mRNA-splicing factor CWC21	43.50	66.00	48.73	84.11	37.91	31.12	-0.05
KLMA_30347		ornithine decarboxylase antizyme	156.37	124.17	164.45	201.85	203.88	175.46	0.39
KLMA_30348	AQY1	aquaporin-2	7865.62	6870.75	6882.41	9916.92	7689.29	7917.48	0.24
KLMA_30349	MNT2	probable mannosyltransferase MNT2	880.62	740.55	803.96	685.46	867.75	881.64	0.01
KLMA_30350	CNS1	hsp70/Hsp90 co-chaperone CNS1	410.33	344.54	375.18	263.25	321.83	318.95	-0.32
KLMA_30351		INCENP_ARK-bind super family protein	399.75	442.99	422.69	625.75	411.97	481.44	0.26
KLMA_30352	SSY1	SPS-sensor component SSY1	258.66	267.36	272.86	397.82	342.89	391.55	0.50
KLMA_30353		hypothetical protein	206.93	134.24	170.54	243.91	145.75	197.07	0.20
KLMA_30354	LEE1	YTH1[COG5084]	362.12	340.07	328.89	1725.01	1139.87	1509.16	2.08
KLMA_30355	AMN1	antagonist of mitotic exit network protein 1	931.18	827.80	877.05	433.99	475.16	519.48	-0.88
KLMA_30356	LGE1	transcriptional regulatory protein	176.36	202.48	210.74	214.47	195.45	216.95	0.09

Locus_tag	UniProt_gene	Product	Unique exon reads						log2 Fold Change
			KmWT.1	KmWT.2	KmWT.3	Kmmig1.1	Kmmig1.2	Kmmig1.3	
		LGE1							
KLMA_30357		3-ketoacyl-CoA reductase	1102.83	1017.97	1114.59	1127.02	1269.61	1321.60	0.20
KLMA_30358	CDC28	cell division control protein 28	433.84	484.38	549.37	312.03	374.90	400.20	-0.43
KLMA_30359	SUR1	mannosyl phosphorylinositol ceramide synthase SUR1	4802.85	4067.41	4435.20	10299.60	5347.20	6154.20	0.71
KLMA_30360	TOS1	protein TOS1	10754.39	8039.74	9713.34	10806.76	13851.17	12239.25	0.37
KLMA_30361		protein YSY6	51.73	61.53	45.07	47.94	61.50	47.54	-0.01
KLMA_30362	PDR12	ATP-dependent permease PDR12	969.98	550.38	914.81	554.26	573.73	570.47	-0.52
KLMA_30363	PDR12	ATP-dependent permease PDR12	763.05	813.26	667.53	3924.38	2629.37	2854.10	2.07
KLMA_30364	EXO5	defects in morphology protein 1	82.30	102.92	109.63	322.97	197.14	246.34	1.38
KLMA_30365		putative pyridoxal reductase	302.16	232.68	232.66	1685.48	1477.71	1827.24	2.70
KLMA_30366	GRX5	monothiol glutaredoxin-5	385.64	398.24	365.44	440.71	469.26	410.57	0.20
KLMA_30367		uncharacterized protein YLR257W	265.71	306.51	221.70	430.62	337.83	383.77	0.54
KLMA_30368		dentin matrix acidic phosphoprotein 1	1047.57	925.12	863.65	1798.18	1391.77	1477.18	0.72
KLMA_30369		hypothetical protein	1328.57	1826.76	1152.35	3772.15	2377.47	3002.76	1.09
KLMA_30370	GSY2	glycogen [starch] synthase isoform 2	7570.52	9695.34	8346.60	2715.78	2292.38	2344.99	-1.80
KLMA_30371	CMK2	calcium/calmodulin-dependent protein kinase II	447.95	653.29	430.00	1664.46	1077.53	1249.86	1.38
KLMA_30372	PDC2	protein PDC2	1152.21	1128.72	1170.62	438.19	399.33	423.53	-1.45
KLMA_30373	VPS41	vacuolar protein sorting-associated protein 41	811.25	778.58	756.46	582.85	581.31	567.88	-0.44
KLMA_30374	COG3	conserved oligomeric Golgi complex subunit 3	631.37	526.88	585.92	460.06	534.97	554.05	-0.17
KLMA_30375	PET100	protein PET100	554.94	542.55	532.32	445.76	424.61	385.50	-0.38
KLMA_30376		UPF0160 protein	1651.90	1387.13	1586.00	1766.22	1629.35	1706.23	0.14

Locus_tag	UniProt_gene	Product	Unique exon reads						log2 Fold Change
			KmWT.1	KmWT.2	KmWT.3	Kmmig1.1	Kmmig1.2	Kmmig1.3	
KLMA_30377		YER156C cation transport regulator-like protein	335.08	376.99	330.11	386.05	273.81	305.98	-0.11
KLMA_30378	STT3	dolichyl-diphosphooligosaccharide--protein glycosyltransferase subunit STT3	2128.07	2449.84	1950.22	2058.07	2635.27	2678.63	0.18
KLMA_30379		uncharacterized protein YBL010C	215.16	241.63	215.61	142.14	197.14	222.14	-0.26
KLMA_30380	ALK2	serine/threonine-protein kinase Haspin homolog	891.20	822.21	898.98	254.00	477.68	528.12	-1.05
KLMA_30381		hypothetical protein	76.42	55.93	99.89	105.97	97.73	106.32	0.42
KLMA_30382	NSR1	nuclear localization sequence-binding protein	3066.30	2825.71	3357.16	783.03	812.15	819.41	-1.94
KLMA_30383	RIM8	pH-response regulator protein palF/RIM8	519.67	601.83	492.12	903.30	521.49	694.08	0.39
KLMA_30384	CCT2	T-complex protein 1 subunit beta	1441.44	1523.60	1404.50	966.38	1341.22	1336.29	-0.26
KLMA_30385	END3	actin cytoskeleton-regulatory complex protein END3	832.42	892.68	828.33	1622.40	1347.96	1408.90	0.78
KLMA_30386	AXL2	protein AXL2	1907.03	2105.30	1861.30	1211.12	1491.18	1637.95	-0.44
KLMA_30387	Acot10	acyl-coenzyme A thioesterase 10	995.84	951.97	1196.20	689.67	1111.23	1478.04	0.06
KLMA_30388	SAL1	calcium-binding mitochondrial carrier SAL1	483.22	498.92	639.52	1246.45	582.15	676.79	0.63
KLMA_30389	REV7	DNA polymerase zeta processivity subunit	124.63	100.68	114.50	115.23	124.69	118.42	0.08
KLMA_30390	PMS1	DNA mismatch repair protein PMS1	457.36	428.44	473.85	941.99	612.48	629.25	0.68
KLMA_30391		37S ribosomal protein SWS2	219.86	198.00	158.36	132.89	246.85	184.11	-0.03
KLMA_30392		UPF0657 nucleolar protein YBR141C	213.98	208.07	194.90	328.01	279.70	260.17	0.49
KLMA_30393	MAK5	ATP-dependent RNA helicase MAK5	679.57	636.51	750.37	461.74	446.51	463.29	-0.59

Locus_tag	UniProt_gene	Product	Unique exon reads						log2 Fold Change
			KmWT.1	KmWT.2	KmWT.3	Kmmig1.1	Kmmig1.2	Kmmig1.3	
KLMA_30394	SUP45	eukaryotic peptide chain release factor subunit 1	2794.71	2814.52	2867.47	1472.69	1892.20	1758.96	-0.73
KLMA_30395		ZZ_NBR1_like protein	328.03	539.19	303.31	857.04	770.87	839.29	1.07
KLMA_30396	PHM7	phosphate metabolism protein 7	253.96	485.49	194.90	4467.70	2595.67	3249.97	3.46
KLMA_30397		uncharacterized protein YDR282C	382.11	567.16	410.51	465.11	437.25	426.13	-0.03
KLMA_30398	DOG2	2-deoxyglucose-6-phosphate phosphatase 2	9655.08	5736.44	8439.18	5018.60	4499.67	3930.22	-0.83
KLMA_30399		conserved hypothetical membrane protein	14.11	2.24	12.18	70.65	32.86	24.20	2.17
KLMA_30400	RRP45	exosome complex component RRP45	322.15	280.78	282.61	285.96	308.35	252.39	-0.06
KLMA_30401	RNH202	ribonuclease H2 subunit B	206.93	173.39	165.67	124.48	152.49	175.46	-0.27
KLMA_30402		putative uncharacterized protein YHR045W	285.70	255.05	325.24	210.26	233.37	226.46	-0.37
KLMA_30403	APE2	aminopeptidase 2	2809.99	3498.02	2994.15	3566.93	3683.31	3829.95	0.25
KLMA_30404	FSH1	family of serine hydrolases 1	184.59	128.64	185.16	349.04	135.64	188.43	0.43
KLMA_30405	TAD2	tRNA-specific adenosine deaminase subunit TAD2	57.61	35.80	43.85	105.13	104.47	132.25	1.32
KLMA_30406	SNX4	sorting nexin-4	181.06	278.54	168.10	618.18	472.63	479.72	1.32
KLMA_30407	BRE2	COMPASS component BRE2	146.97	140.95	157.14	301.94	243.48	270.54	0.88
KLMA_30408	PPR1	pyrimidine pathway regulatory protein 1	344.49	314.34	394.67	395.30	328.57	403.65	0.10
KLMA_30409		glutaredoxin-like protein YDR286C	98.76	99.56	103.54	64.76	96.88	87.30	-0.28
KLMA_30410	ZIP1	synaptonemal complex protein ZIP1	117.57	83.90	124.25	378.48	350.47	280.91	1.63
KLMA_30411	YIM1	uncharacterized protein YMR152W	1697.75	1823.40	1659.09	4206.98	3840.01	3836.00	1.20
KLMA_30412	DPP1	diacylglycerol pyrophosphate phosphatase 1	1198.07	974.34	997.65	3671.22	2229.19	2080.50	1.33

Locus_tag	UniProt_gene	Product	Unique exon reads						log2 Fold Change
			KmWT.1	KmWT.2	KmWT.3	Kmmig1.1	Kmmig1.2	Kmmig1.3	
KLMA_30413	GCN2	serine/threonine-protein kinase GCN2	232.79	379.22	280.17	471.83	409.44	435.63	0.56
KLMA_30414	SRB2	mediator of RNA polymerase II transcription subunit	81.13	119.70	82.83	142.14	152.49	123.60	0.56
KLMA_30415	RTT103	regulator of Ty1 transposition	310.39	300.92	282.61	330.54	369.85	327.59	0.20
KLMA_30416	HRQ1	putative ATP-dependent helicase HRQ1	350.37	542.55	388.58	912.55	580.47	587.76	0.70
KLMA_30417	SRP101	signal recognition particle receptor subunit alpha homolog	1492.00	1463.19	1616.45	1037.87	989.07	977.58	-0.61
KLMA_30418	OLE1	probable acyl-CoA desaturase	1613.10	1370.35	1504.39	1206.08	930.09	985.36	-0.52
KLMA_30419	BCD1	box C/D snoRNA protein 1	92.88	110.75	96.23	206.90	100.25	120.15	0.51
KLMA_30420	VMA10	v-type proton ATPase subunit G	288.05	247.22	313.06	220.36	411.13	303.39	0.14
KLMA_30421	ALDH22A1	putative aldehyde dehydrogenase-like protein YHR039C	1979.93	1162.28	1671.27	844.42	1169.36	1418.40	-0.49
KLMA_30422	RRF1	ribosome-recycling factor	252.78	255.05	224.14	235.50	323.51	295.61	0.22
KLMA_30423	SSD1	protein SSD1	3034.56	3454.39	2850.42	2719.14	2543.44	2638.01	-0.24
KLMA_30424		delta-1-pyrroline-5-carboxylate dehydrogenase	674.87	837.87	677.28	1205.24	873.65	1006.11	0.49
KLMA_30425	BRL1	nucleus export protein BRL1	350.37	341.19	431.22	222.04	246.85	255.85	-0.63
KLMA_30426	DPL1	sphingosine-1-phosphate lyase	511.44	866.95	576.17	4165.76	2535.01	3091.79	2.32
KLMA_30427	HDA2	HDA1 complex subunit 2	912.37	808.78	835.63	828.44	840.79	767.55	-0.07
KLMA_30428		uncharacterized protein YHR035W	210.46	264.00	221.70	687.99	564.46	645.67	1.45
KLMA_30429	MHR1	mitochondrial homologous recombination protein 1	792.44	740.55	706.51	336.42	552.67	455.51	-0.74
KLMA_30430	SUR2	sphingolipid C4-hydroxylase SUR2	7027.33	3678.12	6251.42	1977.33	1693.38	1646.59	-1.67
KLMA_30431	atpH	ATP synthase	5136.76	4908.64	5092.99	4476.11	5206.51	4141.12	-0.13

Locus_tag	UniProt_gene	Product	Unique exon reads						log2 Fold Change
			KmWT.1	KmWT.2	KmWT.3	Kmmig1.1	Kmmig1.2	Kmmig1.3	
		subunit 5							
KLMA_30432	BFR2	protein BFR2	815.96	759.56	800.31	309.51	289.81	341.42	-1.34
KLMA_30433		uncharacterized transporter C4B3.13	611.38	496.68	515.27	760.32	675.67	591.22	0.32
KLMA_30434	PIH1	protein interacting with Hsp90 1	121.10	115.22	115.72	270.82	226.63	267.09	1.12
KLMA_30435	PRO1	glutamate 5-kinase	653.71	505.63	657.79	335.58	351.31	417.48	-0.72
KLMA_30436	CFT1	protein CFT1	460.89	542.55	618.81	406.23	503.80	498.73	-0.20
KLMA_30437	GPI11	glycosylphosphatidylinositol anchor biosynthesis protein 11	281.00	293.09	292.35	298.58	443.14	358.71	0.34
KLMA_30438		conserved hypothetical protein	204.58	198.00	147.39	195.97	277.18	223.00	0.34
KLMA_30439	CPR5	peptidyl-prolyl cis-trans isomerase D	2026.96	1750.69	1911.24	1524.00	1866.93	1804.77	-0.13
KLMA_30440	MED6	mediator of RNA polymerase II transcription subunit 6	637.24	536.95	568.86	746.02	569.51	663.82	0.18
KLMA_30441	FYV4	protein FYV4	135.21	149.90	97.45	179.15	225.78	168.55	0.58
KLMA_30442		F-box protein YDR306C	322.15	277.43	324.02	692.19	459.99	494.41	0.83
KLMA_30443	BEM4	bud emergence protein 4	316.27	322.17	319.15	581.17	598.16	564.42	0.86
KLMA_30444		vacuolar membrane protein YPL162C	121.10	166.68	110.85	317.08	330.25	397.60	1.39
KLMA_30445		conserved hypothetical protein	5779.88	4570.81	5279.36	4096.80	5182.08	5503.34	-0.08
KLMA_30446	MLH3	DNA mismatch repair protein MLH3	158.72	177.87	236.32	588.74	332.78	407.97	1.22
KLMA_30447		anaphase-promoting complex subunit 5	209.28	293.09	261.90	391.09	325.20	388.09	0.53
KLMA_30448	CLP1	protein CLP1	228.09	193.53	188.81	301.10	309.19	293.88	0.57
KLMA_30449	TUM1	putative thiosulfate sulfurtransferase	400.92	606.31	434.87	682.10	631.86	602.45	0.41
KLMA_30450	SET6	potential protein lysine methyltransferase SET6	284.53	421.73	315.49	490.34	374.90	335.37	0.23
KLMA_30451	ATG29	autophagy-related	106.99	154.37	158.36	238.86	179.45	197.07	0.55

Locus_tag	UniProt_gene	Product	Unique exon reads						log$_2$ Fold Change
			KmWT.1	KmWT.2	KmWT.3	Kmmig1.1	Kmmig1.2	Kmmig1.3	
		protein 29							
KLMA_30452	REV3	DNA polymerase zeta catalytic subunit	124.63	167.80	146.18	470.99	281.39	286.97	1.24
KLMA_30453	TMA16	translation machinery-associated protein 16	404.45	436.27	456.80	251.48	294.02	274.86	-0.66
KLMA_30454		uncharacterized protein YPL168W	151.67	143.19	162.01	182.51	278.02	237.70	0.61
KLMA_30455	NAT5	N-terminal acetyltransferase A complex subunit NAT5	239.85	189.05	227.79	215.31	298.24	273.14	0.26
KLMA_30456		protein translocation protein SEC63	759.52	834.51	740.62	592.11	755.70	737.29	-0.16
KLMA_30457	MEX67	mRNA export factor MEX67	1172.20	1114.18	1162.09	470.15	536.66	591.22	-1.11
KLMA_30458		outer spore wall protein 1	32.92	71.59	51.16	141.30	80.04	67.42	0.89
KLMA_30459	DAP1	damage response protein 1	734.83	737.19	795.44	472.67	490.32	458.97	-0.67
KLMA_30460	TRE2	putative zinc metalloprotease TRE2	1053.45	1064.96	958.67	1052.16	838.26	925.72	-0.13
KLMA_30461	SPT14	phosphatidylinositol N-acetylglucosaminyltransferase GPI3 subunit	258.66	274.07	261.90	325.49	257.80	290.42	0.14
KLMA_30462	NIP100	protein NIP100	145.79	165.56	181.50	393.62	309.19	323.27	1.06
KLMA_30463	CDC31	cell division control protein 31	104.64	104.03	108.41	113.54	171.02	133.97	0.40
KLMA_30464	MRPL40	54S ribosomal protein L40	466.76	426.21	459.23	157.28	235.89	284.37	-1.00
KLMA_30465	HNT3	aprataxin-like protein	109.34	89.49	107.20	157.28	157.54	165.96	0.65
KLMA_30466	RPT4	26S protease subunit RPT4	1102.83	1361.40	1074.39	1139.63	1185.37	1185.89	-0.01
KLMA_30467	GCD1	translation initiation factor eIF-2B subunit gamma	1325.05	1083.97	1247.36	730.04	868.59	837.56	-0.59
KLMA_30468	COX10	protoheme IX farnesyltransferase	713.67	767.39	689.46	624.07	731.27	761.50	-0.04
KLMA_30469	RPN8	26S proteasome regulatory subunit	788.91	870.31	762.55	948.71	1125.55	1211.82	0.44

Locus_tag	UniProt_gene	Product	Unique exon reads						log$_2$ Fold Change
			KmWT.1	KmWT.2	KmWT.3	Kmmig1.1	Kmmig1.2	Kmmig1.3	
		RPN8							
KLMA_30470		GPN-loop GTPase 2 homolog	99.94	110.75	90.14	122.79	106.99	115.82	0.20
KLMA_30471		protein DSE3	705.44	767.39	738.18	731.72	689.15	795.21	0.00
KLMA_30472	RBL2	tubulin-specific chaperone A	90.53	88.37	86.49	79.06	120.47	134.84	0.33
KLMA_30473		uncharacterized transporter YBR287W	1029.94	748.38	873.40	1128.70	1099.43	1119.34	0.34
KLMA_30474	NET1	nucleolar protein NET1	1132.23	1186.89	1074.39	625.75	733.80	703.58	-0.72
KLMA_30475		uncharacterized protein YKR011C	296.28	325.53	343.51	330.54	320.14	357.84	0.06
KLMA_30476	SPO22	sporulation-specific protein 22	686.63	344.54	687.02	264.09	303.29	312.03	-0.97
KLMA_30477	SER3	D-3-phosphoglycerate dehydrogenase 1	9362.33	5643.59	8738.84	2291.88	3294.93	3565.46	-1.38
KLMA_30478	RPN2	26S proteasome regulatory subunit RPN2	3718.83	4438.81	3694.58	2767.08	3214.05	3154.03	-0.38
KLMA_30479	UTP7	U3 small nucleolar RNA-associated protein 7	606.68	528.00	602.97	238.86	317.61	325.86	-0.98
KLMA_30480	URA9	dihydroorotate dehydrogenase	1245.10	1056.01	1233.96	603.04	780.98	774.46	-0.71
KLMA_30482		glyoxalase super family protein	532.61	591.77	643.17	2439.91	2787.76	2694.19	2.16
KLMA_30483	GET2	Golgi to ER traffic protein 2	634.89	625.33	662.66	371.75	420.40	525.53	-0.55
KLMA_30484	SEC28	coatomer subunit epsilon	625.49	663.36	615.15	333.06	400.18	509.97	-0.62
KLMA_30485	ARE2	sterol O-acyltransferase 2	1589.58	1336.79	1649.34	1120.29	792.77	879.05	-0.71
KLMA_30486	ATP23	mitochondrial inner membrane protease ATP23	290.40	307.63	285.04	174.10	301.61	248.07	-0.29
KLMA_30487		ankyrin repeat-containing protein YCR051W	366.83	293.09	337.42	229.61	282.23	269.68	-0.35
KLMA_30488		UPF0674 endoplasmic reticulum membrane protein YNR021W	1370.90	1519.13	1461.75	809.10	1240.97	1200.59	-0.42

Locus_tag	UniProt_gene	Product	Unique exon reads						log$_2$ Fold Change
			KmWT.1	KmWT.2	KmWT.3	Kmmig1.1	Kmmig1.2	Kmmig1.3	
KLMA_30489	MRPL50	54S ribosomal protein L50	216.33	168.92	208.30	148.03	222.41	165.09	-0.15
KLMA_30490	RSC6	transcription regulatory protein SNF12	759.52	785.29	806.40	456.69	519.81	601.59	-0.58
KLMA_30491	THR4	threonine synthase	5132.06	3699.38	4826.22	2384.40	3069.99	3031.29	-0.69
KLMA_30492	CTR86	copper transport protein 86	191.64	165.56	179.06	232.13	163.44	216.09	0.19
KLMA_30493	PWP2	periodic tryptophan protein 2	1506.11	1175.70	1517.79	549.21	717.79	661.23	-1.12
KLMA_30494		m-phase phosphoprotein 6 homolog	117.57	119.70	107.20	61.40	71.61	68.28	-0.78
KLMA_30495	YIH1	protein IMPACT homolog	409.15	338.95	427.56	157.28	185.34	182.38	-1.16
KLMA_30496	TAH1	TPR repeat-containing protein associated with Hsp90	63.49	38.03	56.03	42.89	32.86	27.66	-0.60
KLMA_30497	FKS3	1,3-beta-glucan synthase component GSC2	978.21	793.12	1154.78	630.79	489.48	502.19	-0.85
KLMA_30498		uncharacterized membrane protein YCR061W	4698.21	3352.60	3935.77	3952.13	3476.90	2656.16	-0.25
KLMA_30499	BUD31	bud site selection protein 31	11.76	4.47	10.96	29.44	6.74	15.56	0.93
KLMA_30500	HCM1	forkhead transcription factor HCM1	759.52	784.17	713.82	426.42	393.44	520.34	-0.75
KLMA_30501	RAD18	postreplication repair E3 ubiquitin-protein ligase RAD18	170.48	181.22	202.21	137.09	178.61	173.74	-0.18
KLMA_30502	SEC12	putative guanine nucleotide-exchange factor SED4	835.94	976.58	934.30	511.36	794.46	681.97	-0.47
KLMA_30504	BUD17	putative pyridoxal kinase BUD17	527.90	340.07	486.03	401.18	446.51	433.04	-0.08
KLMA_30505	ATG15	putative lipase ATG15	566.70	682.38	605.41	870.50	595.63	675.92	0.21
KLMA_30506		GAL4 super family protein	613.73	616.38	646.83	669.48	514.75	624.06	-0.05
KLMA_30507	SLX9	ribosome biogenesis protein SLX9	293.93	250.58	294.79	184.19	181.98	221.27	-0.51

Locus_tag	UniProt_gene	Product	Unique exon reads						log$_2$ Fold Change
			KmWT.1	KmWT.2	KmWT.3	Kmmig1.1	Kmmig1.2	Kmmig1.3	
KLMA_30508		PX domain-containing protein YPR097W	364.48	280.78	399.55	622.38	546.77	608.50	0.77
KLMA_30509	HNM1	choline transport protein	5467.14	2933.10	4682.48	1017.68	1955.39	2015.67	-1.39
KLMA_30511	DBP3	ATP-dependent RNA helicase DBP3	1602.52	1157.80	1449.57	519.77	577.94	612.83	-1.30
KLMA_30512	MNN10	uncharacterized alpha-1	423.26	399.36	422.69	407.91	529.08	528.12	0.23
KLMA_30513	PEX5	peroxisomal targeting signal receptor	823.01	825.56	845.38	1105.15	874.49	870.40	0.19
KLMA_30514	PRP28	pre-mRNA-splicing ATP-dependent RNA helicase PRP28	212.81	230.44	276.51	466.79	272.12	286.97	0.51
KLMA_30515	CWC2	pre-mRNA-splicing factor CWC2	156.37	219.26	193.68	222.88	256.96	196.21	0.25
KLMA_30516	NHP2	H/ACA ribonucleoprotein complex subunit 2	1152.21	954.21	1071.95	452.49	686.62	669.01	-0.81
KLMA_30517	SNU56	U1 small nuclear ribonucleoprotein component	250.43	285.26	235.10	485.29	289.81	388.09	0.59
KLMA_30518		uncharacterized protein YDR239C	125.80	209.19	116.94	481.93	344.57	369.94	1.40
KLMA_30519	SEC26	coatomer subunit beta	3289.69	3419.71	3405.88	2167.41	2903.18	2822.98	-0.36
KLMA_30520	rplE	54S ribosomal protein L7	861.81	756.21	821.02	416.32	658.82	582.57	-0.56
KLMA_30521	GLE1	nucleoporin GLE1	371.53	357.97	322.80	310.35	299.92	261.90	-0.27
KLMA_30523	FMN1	riboflavin kinase	192.82	221.49	171.76	290.17	308.35	243.75	0.52
KLMA_30524		putative cation exchanger YDL206W	239.85	362.44	219.26	846.11	639.44	769.27	1.46
KLMA_30525	PRP42	U1 small nuclear ribonucleoprotein component PRP42	204.58	217.02	207.08	229.61	216.52	248.07	0.14
KLMA_30526	HEM3	porphobilinogen deaminase	785.39	720.41	828.33	582.85	918.30	781.38	-0.03
KLMA_30527	LYS4	homoaconitase	2343.23	1776.42	2265.71	1190.94	1764.99	1801.31	-0.42
KLMA_30529	RTN1	reticulon-like protein 1	5112.07	3976.80	4870.07	5023.64	5893.13	5913.04	0.27

Locus_tag	UniProt_gene	Product	Unique exon reads						log$_2$ Fold Change
			KmWT.1	KmWT.2	KmWT.3	Kmmig1.1	Kmmig1.2	Kmmig1.3	
KLMA_30530	HEM1	5-aminolevulinate synthase	2691.24	1828.99	2259.62	2450.00	1841.66	1413.22	-0.25
KLMA_30531	ACK1	activator of C kinase protein 1	196.35	212.54	232.66	197.65	154.17	201.39	-0.21
KLMA_30532	COX20	cytochrome c oxidase protein 20	74.07	93.97	92.58	137.09	168.50	153.85	0.82
KLMA_30533		4,5-DOPA dioxygenase extradiol-like protein	163.43	204.71	135.21	681.26	533.29	545.41	1.80
KLMA_30534	IVY1	protein IVY1	509.09	552.61	504.30	644.25	519.81	445.14	0.04
KLMA_30535	MRPL28	54S ribosomal protein L28	211.63	262.88	254.59	181.67	263.70	257.58	-0.05
KLMA_30536	CGI121	protein CGI121	68.19	55.93	85.27	132.05	138.17	134.84	0.95
KLMA_30537	TFB3	RNA polymerase II transcription factor B subunit 3	279.82	244.98	288.70	294.37	287.28	280.05	0.08
KLMA_30538		hypothetical protein	91.71	76.07	71.87	546.69	230.84	215.22	2.05
KLMA_30539	PMT7	probable dolichyl-phosphate-mannose--protein mannosyltransferase 7	587.86	684.61	668.75	1083.28	817.20	828.92	0.49
KLMA_30540	SRB7	mediator of RNA polymerase II transcription subunit 21	125.80	104.03	104.76	126.16	169.34	124.47	0.33
KLMA_30541	VMA22	vacuolar ATPase assembly protein VMA22	81.13	87.25	120.59	174.10	166.81	120.15	0.68
KLMA_30542	GIC2	GTPase-interacting component 2	257.48	274.07	250.93	185.87	258.64	385.50	0.08
KLMA_30543	RPP1	ribonuclease P/MRP protein subunit RPP1	163.43	137.59	164.45	129.52	163.44	195.34	0.07
KLMA_30544	SUM1	suppressor of mar1-1 protein	1810.62	2135.50	1966.06	988.24	1050.57	1025.99	-0.95
KLMA_30545	PAN5	2-dehydropantoate 2-reductase	768.93	625.33	730.88	268.30	309.19	336.23	-1.22
KLMA_30546	SSZ1	ribosome-associated complex subunit SSZ1	5543.56	5349.39	5262.31	2409.63	3034.60	2938.80	-0.95
KLMA_30547	TFB1	RNA polymerase II transcription factor	402.10	419.49	448.27	226.24	252.74	266.22	-0.77

Locus_tag	UniProt_gene	Product	Unique exon reads						log2 Fold Change
			KmWT.1	KmWT.2	KmWT.3	Kmmig1.1	Kmmig1.2	Kmmig1.3	
		B subunit 1							
KLMA_30548	RRP3	ATP-dependent rRNA helicase RRP3	696.03	695.80	881.92	288.48	367.32	376.86	-1.14
KLMA_30549	SSF1	ribosome biogenesis protein SSF2	942.93	882.62	1008.61	363.34	486.95	535.90	-1.03
KLMA_30550	PIB1	E3 ubiquitin-protein ligase PIB1	126.98	163.32	148.61	438.19	326.88	326.73	1.31
KLMA_30551	SEC18	vesicular-fusion protein SEC18	1254.50	1511.30	1231.53	1458.40	1602.39	1614.61	0.23
KLMA_30552	TIF32	eukaryotic translation initiation factor 3 subunit A	5721.10	5452.30	5748.34	1679.59	2490.36	2447.85	-1.35
KLMA_30553	Plekha8	glycolipid transfer protein	197.52	201.36	191.25	220.36	295.71	252.39	0.38
KLMA_30554	ECM33	cell wall protein ECM33	18404.85	15003.34	17402.15	10812.65	9413.84	8570.93	-0.82
KLMA_30555		conserved hypothetical transmembrane protein	18.81	60.41	4.87	1423.91	748.96	651.72	5.07
KLMA_30556	GAD1	glutamate decarboxylase	2307.96	2134.39	1940.48	5413.89	4027.04	4981.27	1.18
KLMA_30557	SPE2	S-adenosylmethionine decarboxylase proenzyme	1126.35	924.01	1050.02	832.65	977.27	952.52	-0.17
KLMA_30558	HSH49	protein HSH49	142.26	137.59	154.70	232.13	195.45	152.13	0.42
KLMA_30559	GNT1-B	glucose N-acetyltransferase 1-B	447.95	535.83	492.12	782.18	738.85	658.64	0.56
KLMA_30560	RKR1	RING finger protein YMR247C	1060.51	1267.43	1171.84	1104.31	968.01	944.74	-0.21
KLMA_30561	SBA1	co-chaperone protein SBA1	435.02	436.27	360.57	606.40	732.11	637.03	0.68
KLMA_30562		UPF0010 protein YMR099C	609.03	619.73	601.75	623.22	978.96	988.82	0.50
KLMA_30563	PRR1	probable serine/threonine-protein kinase YKL116C	201.05	289.73	282.61	1403.73	852.59	884.23	2.02
KLMA_30564	APN1	DNA-(apurinic or apyrimidinic site) lyase 1	131.68	112.98	114.50	289.32	204.72	277.46	1.10

Locus_tag	UniProt_gene	Product	Unique exon reads						log2 Fold Change
			KmWT.1	KmWT.2	KmWT.3	Kmmig1.1	Kmmig1.2	Kmmig1.3	
KLMA_30565	FEN1	structure-specific endonuclease RAD27	118.75	234.92	163.23	263.25	178.61	233.38	0.38
KLMA_30566	ABF1	transcription factor BAF1	405.63	456.41	479.94	813.30	651.23	593.81	0.62
KLMA_30567	ATP25	uncharacterized protein YMR098C	517.32	539.19	526.23	629.11	540.03	540.22	0.11
KLMA_30568	KTI12	protein KTI12	211.63	225.97	238.75	164.85	219.89	190.16	-0.23
KLMA_30569	MTG1	mitochondrial GTPase 1	159.90	173.39	208.30	265.77	154.17	164.23	0.11
KLMA_30570	SET3	SET domain-containing protein 3	819.48	617.50	937.96	677.89	447.36	538.49	-0.51
KLMA_30571	fap2	L-saccharopine oxidase	141.09	173.39	174.19	379.32	281.39	306.85	0.99
KLMA_30572	IME2	meiosis induction protein kinase IME2/SME1	79.95	66.00	109.63	253.16	160.91	174.60	1.21
KLMA_30573		conserved hypothetical transmembrane protein	743.06	779.70	683.37	7323.10	3592.32	4021.84	2.76
KLMA_30574	MPD2	protein disulfide isomerase MPD2	315.10	548.14	321.59	272.50	321.83	274.86	-0.45
KLMA_30575	APD1	actin patches distal protein 1	337.43	303.15	348.38	259.89	232.52	263.63	-0.39
KLMA_30576	HAL9	halotolerance protein 9	2605.41	3295.54	2894.27	1395.32	1267.09	1391.61	-1.12
KLMA_30577	ARA1	D-arabinose dehydrogenase [NAD(P)+] heavy chain	101.11	136.48	120.59	549.21	445.67	586.03	2.14
KLMA_30578	MSH2	DNA mismatch repair protein MSH2	728.95	737.19	756.46	550.89	498.75	517.75	-0.50
KLMA_30579		uncharacterized protein JIP4	415.03	355.73	389.80	549.21	488.64	484.90	0.39
KLMA_30580	ARL8A	arl10_like protein	1047.57	834.51	975.72	1679.59	1058.99	1102.05	0.43
KLMA_30582	SNF1	carbon catabolite-derepressing protein kinase	825.36	836.75	864.87	795.64	693.36	873.00	-0.10
KLMA_30583	SMF1	manganese transporter SMF1	2052.82	2129.91	2063.51	2116.10	1683.27	1628.44	-0.20
KLMA_30584	SNX3	sorting nexin-3	192.82	204.71	193.68	252.32	262.85	232.51	0.34
KLMA_30585	HAP5	transcriptional	369.18	383.70	370.31	203.54	261.17	248.07	-0.66

Locus_tag	UniProt_gene	Product	Unique exon reads						log2 Fold Change
			KmWT.1	KmWT.2	KmWT.3	Kmmig1.1	Kmmig1.2	Kmmig1.3	
		activator HAP5							
KLMA_30586	PTA1	pre-tRNA-processing protein PTA1	624.31	662.24	693.11	629.11	609.11	618.01	-0.09
KLMA_30587	VTS1	protein VTS1	1775.35	1609.74	1813.79	612.29	935.15	852.25	-1.12
KLMA_30588	PDE2	cAMP phosphodiesterase	1879.99	1836.82	1879.57	923.48	986.54	961.16	-0.96
KLMA_30589	GCV3	glycine cleavage system H protein	2451.39	1889.40	2343.67	1400.36	1904.84	1708.83	-0.41
KLMA_30590		uncharacterized bolA-like protein YAL044W-A	35.27	38.03	60.91	50.46	77.51	60.50	0.49
KLMA_30591	AIM1	bolA-like protein 3	35.27	35.80	54.82	41.21	102.78	78.66	0.82
KLMA_30592	PRT1	eukaryotic translation initiation factor 3 subunit B	3814.06	3452.16	3778.63	1599.69	2403.59	2211.02	-0.83
KLMA_30593		spindle pole component SPC72	119.92	161.09	109.63	202.70	204.72	218.68	0.68
KLMA_30594	PRE10	proteasome component C1	718.37	832.28	744.28	725.83	915.77	919.67	0.16
KLMA_30595	GEM1	mitochondrial Rho GTPase 1	519.67	605.19	598.10	632.48	592.26	516.88	0.02
KLMA_30596	OAF1	oleate-activated transcription factor 1	932.35	1036.99	888.01	1038.71	923.36	804.71	-0.05
KLMA_30597	OAF1	peroxisome proliferation transcriptional regulator	376.23	334.48	300.88	783.03	669.77	704.45	1.09
KLMA_30598	OAF1	oleate-activated transcription factor 1	122.28	162.20	142.52	438.19	340.36	337.96	1.39
KLMA_30599	AIM2	protein AIM2	605.50	728.24	595.66	1206.08	1316.79	1216.15	0.95
KLMA_30600	FLC2	flavin carrier protein 2	1008.78	966.51	931.87	1749.40	1497.92	1686.35	0.76
KLMA_30601		probable metabolite transport protein C1271.09	109.34	93.97	108.41	314.56	156.70	216.95	1.14
KLMA_30602	ACS1	acetyl-coenzyme A synthetase 1	201.05	199.12	235.10	1841.92	6104.59	7296.01	4.58
KLMA_30603		pol_alpha_B_N super family[cl07152]	244.55	271.83	286.26	357.45	306.66	253.26	0.19
KLMA_30604		probable 2-nitropropane	130.51	156.61	159.57	623.22	422.92	502.19	1.79

Locus_tag	UniProt_gene	Product	Unique exon reads						log2 Fold Change
			KmWT.1	KmWT.2	KmWT.3	Kmmig1.1	Kmmig1.2	Kmmig1.3	
		dioxygenase							
KLMA_30605	MRPL11	54S ribosomal protein L11	496.16	479.90	432.43	347.36	410.29	367.35	-0.32
KLMA_30606	TRM8	tRNA (guanine-N(7)-)-methyltransferase	270.42	253.93	327.68	179.15	260.33	250.66	-0.30
KLMA_30607	STL1	sugar transporter STL1	324.50	579.46	285.04	2717.46	2043.85	2616.40	2.63
KLMA_30608	SCW4	probable family 17 glucosidase SCW10	1133.40	1323.36	1042.72	460.90	445.67	640.49	-1.18
KLMA_30609		cell wall integrity sensor MID2	503.21	621.97	382.49	332.22	390.91	579.12	-0.21
KLMA_30610	PMR1	calcium-transporting ATPase 1	1222.76	1318.89	1318.01	940.30	1311.74	1426.18	-0.07
KLMA_30612	SUA5	protein SUA5	558.47	517.94	584.70	328.85	320.14	326.73	-0.77
KLMA_30613		YPT-interacting protein	411.50	436.27	356.91	596.31	715.26	718.28	0.75
KLMA_30614		hypothetical protein	5250.80	4022.67	4234.21	910.87	882.92	930.91	-2.31
KLMA_30615	RAD54	DNA repair and recombination protein RAD54	741.88	660.00	745.49	365.02	299.92	327.59	-1.11
KLMA_30616	HAA1	transcriptional activator HAA1	2048.12	2297.71	1944.13	1380.18	1019.40	1020.80	-0.88
KLMA_30617	FTR1	plasma membrane iron permease	17058.65	13260.48	15372.75	15298.02	9280.73	5750.54	-0.59
KLMA_30618		factor arrest protein 7	23.51	46.98	26.80	27.75	21.90	10.37	-0.70
KLMA_30619	REC8	meiotic recombination protein REC8	89.36	91.73	80.40	227.93	166.81	159.04	1.08
KLMA_30620	RSC4	chromatin structure-remodeling complex subunit RSC4	471.47	535.83	518.92	303.62	491.16	449.46	-0.29
KLMA_30621		hypothetical protein	237.50	267.36	231.44	274.19	295.71	288.69	0.22
KLMA_30622	MRPL13	54S ribosomal protein L13	589.04	481.02	533.54	334.74	500.43	449.46	-0.32
KLMA_30623	mug70	meiotically up-regulated gene 70 protein	174.01	214.78	225.35	285.96	502.12	451.19	1.01
KLMA_30624		conserved hypothetical transmembrane protein	39.97	60.41	20.71	174.10	107.84	123.60	1.74

Locus_tag	UniProt_gene	Product	Unique exon reads						log$_2$ Fold Change
			KmWT.1	KmWT.2	KmWT.3	Kmmig1.1	Kmmig1.2	Kmmig1.3	
KLMA_30625		protein ECM9	119.92	86.14	131.56	188.40	197.98	203.12	0.81
KLMA_30626	OSH7	oxysterol-binding protein homolog 7	935.88	856.89	808.84	748.54	767.50	849.66	-0.14
KLMA_30627		eukaryotic translation initiation factor 2A	1340.33	1418.45	1305.83	749.38	1271.30	1146.13	-0.36
KLMA_30628		hypothetical protein	118.75	106.27	113.29	87.47	85.93	93.35	-0.34
KLMA_30629	FMP48	calcium/calmodulin-dependent protein kinase type 1D	1872.94	2489.00	1860.08	9217.16	5334.57	5374.55	1.68
KLMA_30630		conserved hypothetical membrane protein	2073.99	2233.95	1911.24	4123.71	1968.03	2269.79	0.43
KLMA_30631	ILV5	ketol-acid reductoisomerase	13104.67	7465.87	13245.91	7773.06	11114.80	13113.11	-0.08
KLMA_30632	PRX1	peroxiredoxin-like protein DDB_G0282517	50.56	31.32	64.56	41.21	53.08	70.88	0.18
KLMA_30633	RME1	conserved hypothetical membrane protein	317.45	277.43	291.13	1367.56	848.37	933.50	1.83
KLMA_30634	TAL1	transaldolase	1773.00	1748.45	1871.04	1838.55	3130.65	3485.07	0.65
KLMA_30635		GAL4	843.00	879.26	821.02	722.47	909.88	779.65	-0.08
KLMA_30636		uncharacterized protein YGR042W	146.97	125.29	116.94	193.44	147.43	123.60	0.26
KLMA_30637		bud site selection protein 8	339.79	302.04	331.33	402.03	440.62	452.92	0.41
KLMA_30638		F-box protein YLR352W	206.93	212.54	181.50	263.25	251.06	298.20	0.43
KLMA_30639	KSS1	mitogen-activated protein kinase KSS1	591.39	539.19	553.03	232.13	208.09	260.17	-1.27
KLMA_30640	dao1	D-amino-acid oxidase	464.41	376.99	453.14	826.76	560.25	622.33	0.63
KLMA_30641	NIT3	probable hydrolase NIT3	499.68	522.41	462.89	658.55	618.38	611.96	0.35
KLMA_30642		conserved hypothetical transmembrane protein	82.30	155.49	110.85	381.84	188.71	220.41	1.18
KLMA_30643	ISN1	IMP-specific 5'-nucleotidase 1	523.20	550.38	515.27	819.19	607.43	628.39	0.37
KLMA_30644	PNS1	protein PNS1	409.15	415.02	420.25	2579.53	1411.15	1743.40	2.20

Locus_tag	UniProt_gene	Product	Unique exon reads						log$_2$ Fold Change
			KmWT.1	KmWT.2	KmWT.3	Kmmig1.1	Kmmig1.2	Kmmig1.3	
KLMA_30645	MTR10	mRNA transport regulator MTR10	1725.97	1365.87	1696.85	966.38	1071.63	1044.14	-0.64
KLMA_30646	SME1	small nuclear ribonucleoprotein E	64.67	39.15	67.00	37.01	62.34	68.28	-0.03
KLMA_30647	PET123	37S ribosomal protein PET123	703.09	714.82	721.13	512.20	730.43	652.59	-0.17
KLMA_30648	TRS120	transport protein particle 120 kDa subunit	701.91	797.60	824.67	722.47	755.70	720.87	-0.08
KLMA_30649	PUP1	proteasome component PUP1	1379.13	1388.25	1259.54	1121.13	1476.86	1561.89	0.05
KLMA_30650	ADE8	phosphoribosylglycinamide formyltransferase	982.91	840.11	924.56	539.96	938.52	754.58	-0.30
KLMA_30651	SIZ1	e3 SUMO-protein ligase SIZ1	469.12	417.26	434.87	229.61	275.49	336.23	-0.65
KLMA_30652	STE14	protein-S-isoprenylcysteine O-methyltransferase	424.44	374.75	403.20	690.51	569.51	584.30	0.62
KLMA_30653	VPS71	vacuolar protein sorting-associated protein 71	170.48	149.90	183.94	300.26	249.37	240.29	0.65
KLMA_30654	CAT2	carnitine O-acetyltransferase	78.77	129.76	144.96	385.20	807.94	1027.72	2.65
KLMA_30655	DFM1	DER1-like family member protein 1	118.75	85.02	109.63	111.86	92.67	121.87	0.06
KLMA_30656	RRP17	ribosomal RNA-processing protein 17	420.91	290.85	411.73	288.48	282.23	256.71	-0.44
KLMA_30657	ERD1	protein ERD1	336.26	221.49	321.59	494.54	521.49	606.78	0.88
KLMA_30658	RML2	54S ribosomal protein RML2	979.38	956.45	962.32	576.13	683.25	630.11	-0.62
KLMA_30659		probable aminopeptidase YDR415C	857.11	1009.02	864.87	1419.71	1616.71	1554.11	0.75
KLMA_30660	VMA8	v-type proton ATPase subunit D	707.79	683.50	606.63	521.46	666.40	726.06	-0.06
KLMA_30661	SYF1	pre-mRNA-splicing factor SYF1	569.05	664.48	665.10	950.40	665.56	757.17	0.32
KLMA_30662	AFG1	protein AFG1	482.05	636.51	562.77	619.02	534.13	567.02	0.03
KLMA_30663	MAK10	N-terminal acetyltransferase C complex subunit MAK10	828.89	970.99	836.85	639.20	743.91	865.22	-0.23

Locus_tag	UniProt_gene	Product	Unique exon reads						log$_2$ Fold Change
			KmWT.1	KmWT.2	KmWT.3	Kmmig1.1	Kmmig1.2	Kmmig1.3	
KLMA_30664	RPL12B	60S ribosomal protein L12	14236.90	12560.21	14537.12	4664.51	8826.63	7548.40	-0.97
KLMA_30665		ACBP super family protein	201.05	200.24	210.74	254.84	308.35	284.37	0.47
KLMA_30666	RAD30	DNA polymerase eta	209.28	199.12	232.66	384.36	274.65	282.64	0.56
KLMA_30667	HKR1	herpes_gp2	1528.45	1717.13	1523.88	594.63	688.30	721.74	-1.25
KLMA_30668	ARO80	transcriptional activator ARO80	451.48	519.05	472.63	343.15	305.82	303.39	-0.60
KLMA_30669	GIS4	protein GIS4	707.79	525.77	696.77	629.11	505.49	459.84	-0.28
KLMA_30670	YAP1	AP-1-like transcription factor	2076.34	2021.40	1853.99	1270.00	979.80	1079.58	-0.84
KLMA_30671	ERG6	sterol 24-C-methyltransferase	1816.50	1471.03	2008.69	494.54	576.25	715.68	-1.57
KLMA_30672		probable metabolite transport protein C1271.09	196.35	171.15	152.27	3567.77	1577.96	2200.64	3.82
KLMA_30673		vacuolar morphogenesis protein 7	161.07	209.19	168.10	476.88	457.47	433.91	1.34
KLMA_30674	SKI8	antiviral protein SKI8	348.02	454.17	349.60	371.75	463.36	444.28	0.15
KLMA_30675	CLG1	PHO85 cyclin CLG1	13036.48	11326.34	12154.46	6668.75	5714.52	5895.76	-1.00
KLMA_30676	FRA2	uncharacterized bolA-like protein YGL220W	47.03	53.70	40.20	40.37	54.76	29.39	-0.18
KLMA_30677	ARB1	ABC transporter ATP-binding protein ARB1	4011.59	4087.55	3908.97	1768.75	2213.19	2191.14	-0.96
KLMA_30678		hypothetical protein	339.79	360.21	326.46	470.15	471.79	446.01	0.43
KLMA_30679	SDT1	protein SSM1	112.87	183.46	87.71	926.85	706.00	725.19	2.62
KLMA_30680		DNA repair protein KRE29	118.75	145.42	118.16	131.21	147.43	142.62	0.14
KLMA_30681	VRG4	GDP-mannose transporter	2066.93	1964.35	2026.96	2296.09	2168.54	2232.63	0.14
KLMA_30682		nitrogen regulatory protein GLN3	1689.52	1437.47	1743.14	1016.84	1109.54	1056.24	-0.61
KLMA_30683	YEN1	uncharacterized protein YER041W	417.38	283.02	382.49	370.07	314.24	369.94	-0.04
KLMA_30684	MXR1	peptide methionine sulfoxide reductase	333.91	290.85	313.06	498.75	395.12	514.29	0.59
KLMA_30685		uncharacterized	131.68	112.98	152.27	152.23	203.04	172.01	0.41

Locus_tag	UniProt_gene	Product	Unique exon reads						log2 Fold Change
			KmWT.1	KmWT.2	KmWT.3	Kmmig1.1	Kmmig1.2	Kmmig1.3	
		protein YGL226W							
KLMA_30686	VID30	vacuolar import and degradation protein 30	478.52	734.95	533.54	947.03	781.82	808.17	0.54
KLMA_30687	SAH1	adenosylhomocysteinase	38028.94	19122.21	29974.43	8506.47	18147.80	14394.94	-1.09
KLMA_30688	SHE10	conserved hypothetical protein	1046.40	1191.36	1068.30	688.83	685.78	698.40	-0.67
KLMA_30689	SAP155	SIT4-associating protein SAP155	1452.02	1581.77	1458.10	1283.46	1165.15	1209.23	-0.30
KLMA_30690	ERJ5	ER-localized J domain-containing protein 5	315.10	293.09	264.33	285.96	311.72	346.61	0.11
KLMA_30691	KEG1	conserved hypothetical protein	168.13	212.54	177.85	243.07	209.78	213.50	0.25
KLMA_30692	EMC4	ER membrane protein complex subunit 4	235.15	287.49	285.04	419.69	388.38	389.82	0.57
KLMA_30693	TAN1	tRNA acetyltransferase TAN1	362.12	321.05	311.84	259.89	336.99	298.20	-0.15
KLMA_30694	SEC15	exocyst complex component SEC15	827.71	1013.50	801.53	703.13	847.53	761.50	-0.19
KLMA_30695	DUG1	cys-Gly metallodipeptidase DUG1	3183.87	3612.12	2913.76	1065.62	1224.12	1319.00	-1.43
KLMA_30696		uncharacterized mitochondrial carrier YFR045W	570.23	606.31	592.01	284.28	388.38	370.81	-0.76
KLMA_30697	ADE5,7	bifunctional purine biosynthetic protein ADE5	13050.59	9709.89	13247.12	4185.95	6465.17	6331.39	-1.08
KLMA_30698	MTO1	mitochondrial translation optimization protein 1	672.52	627.56	747.93	350.72	336.99	328.45	-1.01
KLMA_30699	HAP2	transcriptional activator HAP2	158.72	165.56	152.27	332.22	355.53	239.43	0.96
KLMA_30700	CSE1	importin alpha re-exporter	1589.58	1491.16	1550.67	823.40	1212.32	1089.09	-0.57
KLMA_30701	DOC1	anaphase-promoting complex subunit DOC1	215.16	186.81	222.92	347.36	307.50	247.21	0.53
KLMA_30702		hypothetical protein	134.03	133.12	130.34	195.13	155.86	188.43	0.44

Locus_tag	UniProt_gene	Product	Unique exon reads						log2 Fold Change
			KmWT.1	KmWT.2	KmWT.3	Kmmig1.1	Kmmig1.2	Kmmig1.3	
KLMA_30703	KAP114	importin subunit beta-5	652.53	580.58	645.61	496.22	519.81	494.41	-0.31
KLMA_30704		ankyrin repeat-containing protein YGL242C	225.74	215.90	192.46	307.83	304.98	283.51	0.50
KLMA_30705	TAD1	tRNA-specific adenosine deaminase 1	299.81	345.66	300.88	235.50	298.24	262.76	-0.25
KLMA_30706	RTF1	RNA polymerase-associated protein RTF1	848.88	922.89	918.47	777.14	893.87	902.39	-0.06
KLMA_30707	RMD8	sporulation protein RMD8	610.20	617.50	587.14	557.62	511.38	617.15	-0.11
KLMA_30709	GUS1	glutamyl-tRNA synthetase	8672.17	7499.43	8377.05	2889.04	3936.90	3694.25	-1.22
KLMA_30710	ATG32	protein ECM37	380.94	457.53	342.29	1072.35	759.07	911.89	1.22
KLMA_30711	SLN1	osmosensing histidine protein kinase SLN1	1375.60	1290.92	1452.01	1147.20	962.95	1026.85	-0.39
KLMA_30712	RPL40B	ubiquitin-60S ribosomal protein L40	7903.25	6245.43	7612.07	2630.83	5277.28	3964.79	-0.87
KLMA_30713	MLP1	protein MLP1	2123.37	2289.88	2173.14	1283.46	1537.52	1379.51	-0.65
KLMA_30714	MCM10	minichromosome maintenance protein 10	115.22	109.63	105.98	274.19	162.60	172.87	0.88
KLMA_30715		LCB5	3306.15	1324.48	2620.19	830.12	934.31	1010.43	-1.39
KLMA_30716	PCC1	polarized growth chromatin-associated controller 1	101.11	86.14	124.25	74.01	71.61	61.37	-0.59
KLMA_30717		uncharacterized protein YKR096W	5766.95	5032.81	5473.04	1815.00	1830.70	1885.16	-1.56
KLMA_30718		hypothetical protein	54.08	90.61	103.54	120.27	123.00	85.57	0.41
KLMA_30719	PCK1	phosphoenolpyruvate carboxykinase [ATP]	293.93	255.05	249.72	693.03	292.34	389.82	0.78
KLMA_30720	RRD1	serine/threonine-protein phosphatase 2A activator 1	245.73	260.65	285.04	428.10	385.01	439.96	0.66
KLMA_30721	IMP2'	sugar utilization regulatory protein IMP2	419.74	453.05	395.89	682.10	603.21	535.03	0.52

Locus_tag	UniProt_gene	Product	Unique exon reads						log2 Fold Change
			KmWT.1	KmWT.2	KmWT.3	Kmmig1.1	Kmmig1.2	Kmmig1.3	
KLMA_30722	GUT2	glycerol-3-phosphate dehydrogenase	229.27	275.19	237.53	405.39	424.61	447.74	0.78
KLMA_30723	UBP11	ubiquitin carboxyl-terminal hydrolase 7	552.59	696.92	672.41	470.15	590.58	588.62	-0.22
KLMA_30724	SNZ3	probable pyridoxine biosynthesis protein SNZ3	6949.73	5578.71	6644.88	9900.10	11594.17	9813.87	0.71
KLMA_30726	SNO3	probable glutamine amidotransferase SNO3	2819.40	1973.30	2678.66	2460.10	3463.42	2957.82	0.25
KLMA_30727	SMC3	structural maintenance of chromosomes protein 3	771.28	731.60	756.46	750.22	549.30	662.96	-0.20
KLMA_30728	LAC12	lactose permease	119.92	130.88	129.12	1194.30	406.92	457.24	2.44
KLMA_30729		putative uncharacterized oxidoreductase YGL039W	101.11	119.70	110.85	236.34	144.06	139.16	0.65
KLMA_30730		hypothetical protein	112.87	130.88	124.25	107.66	70.77	111.50	-0.34
KLMA_40002	FET3	iron transport multicopper oxidase FET5	89.36	64.88	242.41	393.62	221.57	287.83	1.19
KLMA_40003		probable acid phosphatase	4.70	4.47	2.44	1627.45	772.55	1186.76	8.26
KLMA_40004	THI13	pyrimidine precursor biosynthesis enzyme THI13	4.70	2.24	3.65	48.78	28.64	37.17	3.44
KLMA_40005		UPF0317 protein C5H10.01	27.04	19.02	46.29	124.48	66.56	76.06	1.54
KLMA_40006		uncharacterized transporter C1683.12	109.34	100.68	121.81	291.01	317.61	302.52	1.46
KLMA_40007	OXP1	uncharacterized protein YKL215C	97.59	112.98	92.58	294.37	311.72	385.50	1.71
KLMA_40008		thiamine repressible genes regulatory protein thi1	806.55	503.39	747.93	601.36	492.85	524.66	-0.35
KLMA_40009	DUR3	urea active transporter	83.48	109.63	125.47	169.05	92.67	126.20	0.28
KLMA_40010		uncharacterized protein YMR315W	1025.24	889.33	755.24	1846.12	2700.14	1994.06	1.29
KLMA_40011	ESF2	pre-rRNA-processing protein ESF2	237.50	173.39	190.03	616.50	494.53	446.87	1.37

Locus_tag	UniProt_gene	Product	Unique exon reads						log2 Fold Change
			KmWT.1	KmWT.2	KmWT.3	Kmmig1.1	Kmmig1.2	Kmmig1.3	
KLMA_40012	NOG2	nucleolar GTP-binding protein 2	2158.64	1934.15	2360.73	1223.74	1220.75	1116.74	-0.86
KLMA_40013	POP2	nucleolar GTP-binding protein 2	678.40	658.89	637.08	797.32	612.48	662.09	0.07
KLMA_40014	BRE5	UBP3-associated protein BRE5	2373.80	2132.15	2475.23	878.91	1133.13	968.08	-1.23
KLMA_40015	OCA4	protein OCA4	235.15	239.39	230.23	235.50	213.15	188.43	-0.15
KLMA_40016	LYS9	saccharopine dehydrogenase [NADP+	4226.74	3164.66	3852.93	4680.49	10108.88	9016.07	1.08
KLMA_40017		protein MSO1	156.37	151.02	127.90	270.82	214.83	230.78	0.72
KLMA_40018	OXP1	uncharacterized protein YKL215C	409.15	517.94	440.96	857.88	852.59	860.90	0.91
KLMA_40019	YCT1	uncharacterized transporter YLL055W	1252.15	747.26	1176.71	839.38	283.91	358.71	-1.10
KLMA_40020	UBC1	ubiquitin-conjugating enzyme E2-24 kDa	318.62	436.27	376.40	464.26	417.87	390.69	0.17
KLMA_40021	CDC50	cell division control protein 50	471.47	548.14	443.40	418.01	470.94	397.60	-0.19
KLMA_40022	CDC39	general negative regulator of transcription subunit 1	3916.35	4140.13	4201.32	2243.94	2380.00	2205.83	-0.84
KLMA_40023	MSH3	DNA mismatch repair protein MSH3	467.94	449.70	442.18	638.36	497.06	443.41	0.22
KLMA_40024	FPK1	probable serine/threonine-protein kinase YNR047W	1735.38	2037.06	1582.35	2275.90	1936.01	1876.51	0.19
KLMA_40025	TRM112	multifunctional methyltransferase subunit TRM112	319.80	278.54	333.77	201.01	185.34	171.14	-0.74
KLMA_40026	ERG26	sterol-4-alpha-carboxylate 3-dehydrogenase	2089.27	1816.69	1869.82	888.16	1148.30	1040.68	-0.91
KLMA_40027	VPS8	vacuolar protein sorting-associated protein 8	664.29	703.63	648.04	761.16	638.60	702.72	0.06
KLMA_40028	EFB1	elongation factor 1-beta	15443.19	10273.69	13956.07	9544.33	11892.41	9965.13	-0.34
KLMA_40029	SFH1	chromatin structure-remodeling complex	427.97	470.95	493.34	591.26	613.32	611.10	0.38

Locus_tag	UniProt_gene	Product	Unique exon reads						log$_2$ Fold Change
			KmWT.1	KmWT.2	KmWT.3	Kmmig1.1	Kmmig1.2	Kmmig1.3	
		subunit SFH1							
KLMA_40030	TAD3	tRNA-specific adenosine deaminase subunit TAD3	122.28	184.58	190.03	214.47	187.03	160.77	0.18
KLMA_40031	EST2	telomerase reverse transcriptase	288.05	264.00	264.33	710.69	476.84	528.98	1.07
KLMA_40032	BUD6	bud site selection protein 6	654.88	600.72	579.83	492.02	478.53	546.27	-0.28
KLMA_40034	MMS22	methyl methanesulfonate-sensitivity protein 22	333.91	317.70	360.57	546.69	374.06	369.08	0.35
KLMA_40035	SWC4	SWR1-complex protein 4	337.43	403.83	381.27	401.18	351.31	330.18	-0.05
KLMA_40036	CUL3	cullin-3	393.87	407.19	392.24	535.75	353.00	416.62	0.13
KLMA_40037	CWC24	pre-mRNA-splicing factor CWC24	156.37	127.53	141.30	264.93	165.97	210.04	0.59
KLMA_40038	PEX30	peroxisomal membrane protein PEX30	875.92	1284.21	875.83	989.93	898.08	958.57	-0.09
KLMA_40039	RPL38	60S ribosomal protein L38	2374.97	1955.40	2349.77	842.74	1918.32	1305.17	-0.72
KLMA_40040	TFG2	transcription initiation factor IIF subunit beta	558.47	586.17	470.20	1349.06	897.24	1025.12	1.02
KLMA_40041		pre-mRNA-splicing factor 18	44.68	36.92	34.11	112.70	82.56	74.33	1.22
KLMA_40042		uncharacterized membrane protein YLR326W	94.06	73.83	54.82	218.68	148.28	216.09	1.39
KLMA_40044	ECT1	ethanolamine-phosphate cytidylyltransferase	141.09	145.42	154.70	181.67	127.21	173.74	0.13
KLMA_40045	STF2	ATPase-stabilizing factor 15 kDa protein	810.08	1238.35	747.93	2354.12	1561.95	1338.88	0.91
KLMA_40046	NMA1	nicotinamide-nucleotide adenylyltransferase 1	646.65	460.88	632.21	1373.45	767.50	974.13	0.84
KLMA_40047		putative cysteine synthase	420.91	505.63	380.06	305.30	231.68	282.64	-0.67
KLMA_40048	SNU71	U1 small nuclear ribonucleoprotein component SNU71	194.00	212.54	263.12	339.79	286.44	250.66	0.39

Locus_tag	UniProt_gene	Product	Unique exon reads						log$_2$ Fold Change
			KmWT.1	KmWT.2	KmWT.3	Kmmig1.1	Kmmig1.2	Kmmig1.3	
KLMA_40049	MSB2	hansenula MRAKII killer toxin-resistant protein	1480.24	1398.31	1516.57	4377.71	3790.30	4258.67	1.50
KLMA_40050		uncharacterized abhydrolase domain-containing protein YGR015C	217.51	236.04	215.61	1097.58	816.36	818.54	2.03
KLMA_40051		uncharacterized membrane protein YGR016W	90.53	96.20	120.59	190.92	202.19	153.85	0.83
KLMA_40052		meiotic recombination protein REC102	102.29	120.81	127.90	315.40	235.89	276.59	1.24
KLMA_40053	THI3	thiamine metabolism regulatory protein THI3	1153.39	851.29	1107.28	593.79	658.82	857.44	-0.56
KLMA_40054	RIM11	serine/threonine-protein kinase MDS1/RIM11	1728.32	2335.74	1606.71	2194.32	1794.48	1795.26	0.03
KLMA_40055	MDH3	malate dehydrogenase	467.94	369.15	437.31	1729.22	1125.55	1575.72	1.80
KLMA_40056	CIN4	GTP-binding protein CIN4	79.95	61.53	65.78	100.09	70.77	91.62	0.34
KLMA_40057	PSO2	interstrand crosslink repair protein	286.88	284.14	233.88	893.20	550.98	739.02	1.44
KLMA_40058	GAT2	hypothetical protein	249.25	250.58	254.59	630.79	425.45	551.46	1.09
KLMA_40059	GID8	glucose-induced degradation protein 8	106.99	148.78	163.23	361.66	347.10	333.64	1.32
KLMA_40060		uncharacterized protein YMR134W	239.85	214.78	296.00	321.28	327.72	316.35	0.36
KLMA_40061		cell wall protein YLR040C	11.76	11.19	14.62	449.13	247.69	297.34	4.73
KLMA_40062		meiotic recombination protein	2.35	4.47	2.44	29.44	9.27	13.83	2.50
KLMA_40063	JLP2	protein JLP2	49.38	34.68	35.33	25.23	27.80	27.66	-0.56
KLMA_40064	RRB1	ribosome assembly protein RRB1	1851.77	1339.02	1699.29	572.76	708.52	585.17	-1.39
KLMA_40065	RIC1	protein RIC1	322.15	380.34	363.00	388.57	461.68	409.70	0.24
KLMA_40066	COX12	cytochrome c oxidase subunit 6B	1105.18	1009.02	1132.86	423.89	803.72	500.46	-0.91
KLMA_40067		uncharacterized	163.43	140.95	115.72	148.87	140.69	141.75	0.04

Locus_tag	UniProt_gene	Product	Unique exon reads						log2 Fold Change
			KmWT.1	KmWT.2	KmWT.3	Kmmig1.1	Kmmig1.2	Kmmig1.3	
		protein YMR130W							
KLMA_40068		DNA mismatch repair protein MLH2	18.81	11.19	20.71	386.89	179.45	176.33	3.88
KLMA_40069	POM152	nucleoporin POM152	1657.78	1639.94	1734.61	871.34	1200.53	1131.44	-0.65
KLMA_40070	SMF3	iron transporter SMF3	1874.11	1619.81	1874.70	1803.23	1426.31	1471.99	-0.19
KLMA_40071	RSC58	chromatin structure-remodeling complex protein RSC58	620.78	519.05	579.83	492.86	642.81	573.93	-0.01
KLMA_40072	ECM16	probable ATP-dependent RNA helicase DHR1	600.80	636.51	638.30	268.30	290.65	356.11	-1.04
KLMA_40073	SAS2	histone acetyltransferase SAS2	217.51	239.39	219.26	157.28	163.44	148.67	-0.53
KLMA_40074	RAD5	DNA repair protein RAD5	164.60	166.68	200.99	378.48	223.26	254.98	0.69
KLMA_40075	DLT1	uncharacterized membrane protein YMR126C	382.11	428.44	437.31	529.03	417.87	442.55	0.16
KLMA_40076	CBC1	nuclear cap-binding protein complex subunit 1	2139.83	1712.65	2258.41	1075.71	1502.14	1333.70	-0.64
KLMA_40077		uncharacterized protein YMR124W	293.93	300.92	311.84	154.75	214.83	265.36	-0.51
KLMA_40078		v-type ATPase assembly factor PKR1	4203.23	5460.13	3924.80	4083.34	2749.85	3788.46	-0.36
KLMA_40080	RPL15B	60S ribosomal protein L15-B	11877.21	9449.24	11234.78	3143.88	5236.00	4319.17	-1.36
KLMA_40081	ADE17	bifunctional purine biosynthesis protein ADE17	13137.59	9439.17	12060.67	6328.13	9037.25	9367.86	-0.49
KLMA_40082	ASI1	protein ASI1	998.19	1016.85	892.89	626.59	570.36	669.01	-0.64
KLMA_40083	AAT2	aspartate aminotransferase	2386.73	1863.67	2318.09	1540.82	1438.11	1606.83	-0.52
KLMA_40084	fnx1	multidrug resistance protein fnx1	1195.72	807.67	958.67	2409.63	1441.48	1776.25	0.93
KLMA_40085	SED5	integral membrane protein SED5	223.39	233.80	186.37	411.28	347.94	400.20	0.85
KLMA_40086	SNF7	vacuolar-sorting protein SNF7	422.09	648.82	439.74	952.08	615.01	726.06	0.60

Locus_tag	UniProt_gene	Product	Unique exon reads						log2 Fold Change
			KmWT.1	KmWT.2	KmWT.3	Kmmig1.1	Kmmig1.2	Kmmig1.3	
KLMA_40087	ARN2	siderophore iron transporter ARN2	2868.78	3504.73	3573.98	2812.50	726.22	431.31	-1.33
KLMA_40088	YMC1	carrier protein YMC1	397.40	283.02	372.75	800.69	411.97	455.51	0.66
KLMA_40089	YKE4	zinc transporter YKE4	148.14	176.75	142.52	202.70	166.81	240.29	0.38
KLMA_40090		acyl-protein thioesterase 1	184.59	217.02	181.50	209.42	236.74	298.20	0.35
KLMA_40091		NEDD8-like protein RUB1	366.83	483.26	386.15	298.58	286.44	335.37	-0.43
KLMA_40093	BBP	branchpoint-bridging protein	376.23	398.24	397.11	200.17	215.67	235.10	-0.85
KLMA_40094	RPS13	40S ribosomal protein S13	18136.79	13348.86	17617.76	6464.38	10750.01	8374.72	-0.94
KLMA_40095	AIM7	uncharacterized protein YDR063W	126.98	149.90	146.18	158.96	235.89	250.66	0.61
KLMA_40096	TPO1	polyamine transporter 1	1620.15	1828.99	1441.04	1719.96	985.70	1158.23	-0.34
KLMA_40097	FRA1	putative Xaa-Pro aminopeptidase	2739.45	2572.90	2407.02	1958.83	2228.35	2258.56	-0.26
KLMA_40098	ISA1	iron sulfur assembly-related protein	298.64	447.46	304.53	1180.85	812.99	834.97	1.43
KLMA_40099	HSP104	heat shock protein 104	708.96	809.90	722.35	1169.07	1032.88	1121.93	0.57
KLMA_40100	RIM4	meiotic activator RIM4	219.86	164.44	275.30	215.31	299.92	312.90	0.33
KLMA_40101	NPR3	protein RMD11	590.22	586.17	663.88	365.02	344.57	355.25	-0.79
KLMA_40102	ADH1	alcohol dehydrogenase 1	92020.74	137401.60	103300.76	43612.26	54665.82	60675.03	-1.07
KLMA_40103	CHO2	phosphatidylethanolamine N-methyltransferase	3172.12	990.01	2252.32	748.54	1966.34	1811.69	-0.50
KLMA_40104	MTR3	exosome complex component MTR3	340.96	310.98	345.95	197.65	223.26	228.19	-0.62
KLMA_40105		uncharacterized glycosyl hydrolase YBR056W	164.60	192.41	130.34	917.59	557.72	718.28	2.17
KLMA_40106		uncharacterized protein YDR210W	34.10	53.70	29.24	126.16	104.47	107.18	1.52
KLMA_40107	ARO3	phospho-2-dehydro-3-deoxyheptonate aldolase	2861.72	1797.67	2862.60	1238.88	1728.76	1682.03	-0.69
KLMA_40108	MUM2	hypothetical protein	148.14	128.64	168.10	294.37	227.47	257.58	0.81

Locus_tag	UniProt_gene	Product	Unique exon reads						log2 Fold Change
			KmWT.1	KmWT.2	KmWT.3	Kmmig1.1	Kmmig1.2	Kmmig1.3	
KLMA_40109	UBP14	ubiquitin carboxyl-terminal hydrolase 14	999.37	1128.72	1040.28	535.75	621.75	635.30	-0.82
KLMA_40110	EHD3	3-hydroxyisobutyryl-CoA hydrolase	1908.21	2028.11	1817.44	1006.75	1526.57	1636.22	-0.46
KLMA_40111	dsd1	dihydroceramide delta(4)-desaturase	1455.55	755.09	1321.67	407.91	372.37	431.31	-1.54
KLMA_40112	AKL1	serine/threonine-protein kinase ppk29	1100.48	1087.33	1071.95	985.72	968.01	961.16	-0.16
KLMA_40113	ORC2	origin recognition complex subunit 2	317.45	289.73	373.96	206.90	197.98	225.60	-0.64
KLMA_40114	RPC11	DNA-directed RNA polymerase III subunit RPC10	116.40	80.54	93.80	58.87	70.77	55.32	-0.65
KLMA_40115	HEM13	coproporphyrinogen-III oxidase	2767.66	1846.89	2597.05	418.85	704.31	708.77	-1.98
KLMA_40117	NRG1	transcriptional regulator NRG1	126.98	168.92	120.59	296.05	299.92	379.45	1.23
KLMA_40118	SLT11	pre-mRNA-splicing factor SLT11	118.75	130.88	138.87	181.67	110.36	132.25	0.13
KLMA_40119		hypothetical protein	81.13	93.97	97.45	160.64	128.06	114.09	0.56
KLMA_40120		30S ribosomal protein S10	420.91	454.17	433.65	247.27	402.70	352.66	-0.38
KLMA_40121	TRM7	tRNA (uridine-2'-O-)-methyltransferase TRM7	217.51	187.93	242.41	181.67	264.54	272.27	0.15
KLMA_40122	KRS1	lysyl-tRNA synthetase	5019.19	4210.60	4739.73	1805.75	3283.98	2852.37	-0.81
KLMA_40123	BAP3	valine amino-acid permease	7211.92	3813.48	7020.06	1591.28	934.31	713.09	-2.48
KLMA_40124	DET1	putative phosphoglycerate mutase DET1	102.29	119.70	164.45	190.08	122.16	122.74	0.17
KLMA_40125	TPI1	triosephosphate isomerase	37357.60	32259.64	38535.43	4064.84	6026.24	5441.97	-2.80
KLMA_40126	ALG14	UDP-N-acetylglucosamine transferase subunit ALG14	213.98	210.31	170.54	190.08	265.38	293.02	0.33
KLMA_40127		chaperone protein htpG	104.64	137.59	137.65	107.66	159.23	150.40	0.14
KLMA_40128	HSP26	heat shock protein 26	201.05	295.32	199.77	1609.79	1202.21	1028.58	2.46

Locus_tag	UniProt_gene	Product	Unique exon reads						log$_2$ Fold Change
			KmWT.1	KmWT.2	KmWT.3	Kmmig1.1	Kmmig1.2	Kmmig1.3	
KLMA_40129	EMP70	transmembrane 9 superfamily member 1	1200.42	1289.80	1212.04	1048.80	1286.46	1144.40	-0.09
KLMA_40130	TRS85	transport protein particle 85 kDa subunit	317.45	392.65	383.71	346.52	304.13	323.27	-0.17
KLMA_40131	RAX2	bud site selection protein RAX2	2691.24	2094.11	2392.40	1326.35	1982.35	1956.90	-0.45
KLMA_40132		transcription activator	1379.13	1419.57	1336.28	538.28	454.10	461.56	-1.51
KLMA_40133		uncharacterized sugar kinase YDR109C	604.32	1469.91	727.22	2656.90	2817.24	3620.78	1.70
KLMA_40134	ARP6	actin-like protein ARP6	159.90	195.76	160.79	124.48	149.96	150.40	-0.28
KLMA_40135	SMC4	structural maintenance of chromosomes protein 4	835.94	929.60	902.63	618.18	531.60	558.37	-0.64
KLMA_40136	CSF1	protein CSF1	800.67	812.14	744.28	873.86	625.96	640.49	-0.14
KLMA_40137	GAA1	GPI transamidase component GAA1	815.96	705.87	816.14	736.77	1016.03	818.54	0.14
KLMA_40138	FOB1	DNA replication fork-blocking protein FOB1	352.72	368.04	367.87	232.97	303.29	278.32	-0.42
KLMA_40139	ALT1	probable alanine aminotransferase	2887.59	2138.86	2556.85	2456.73	2444.03	2153.97	-0.10
KLMA_40140	DNAJA2	dnaJ protein homolog XDJ1	362.12	334.48	324.02	399.50	333.62	370.81	0.11
KLMA_40141		hypothetical protein	264.54	247.22	282.61	387.73	409.44	378.59	0.57
KLMA_40142	GEP5	uncharacterized protein YLR091W	162.25	204.71	137.65	158.96	149.96	152.99	-0.13
KLMA_40143		54S ribosomal protein L34	175.18	135.36	164.45	124.48	214.83	152.13	0.05
KLMA_40144		54S ribosomal protein L1	402.10	375.87	397.11	228.77	321.83	299.07	-0.47
KLMA_40145	TMA64	translation machinery-associated protein 64	402.10	399.36	333.77	1184.21	743.06	851.39	1.29
KLMA_40146		hypothetical protein	74.07	63.76	101.10	110.18	98.57	99.40	0.37
KLMA_40147	SUL2	sulfate permease 2	14938.81	8595.71	13935.36	5537.53	5546.87	6441.16	-1.10
KLMA_40148		putative aryl-alcohol dehydrogenase	258.66	204.71	259.46	571.92	687.46	980.18	1.63

Locus_tag	UniProt_gene	Product	Unique exon reads						log2 Fold Change
			KmWT.1	KmWT.2	KmWT.3	Kmmig1.1	Kmmig1.2	Kmmig1.3	
		YPL088W							
KLMA_40149		hypothetical protein	6102.03	5900.88	6453.63	2424.77	2106.19	1779.70	-1.55
KLMA_40150	TRM1	N2,N2-dimethylguanosine-specific tRNA methyltransferase	459.71	462.00	447.05	203.54	243.48	303.39	-0.87
KLMA_40151	DPB4	DNA polymerase epsilon subunit D	82.30	73.83	77.96	62.24	60.66	89.89	-0.14
KLMA_40152	NYV1	vacuolar v-SNARE NYV1	216.33	255.05	203.43	570.24	465.89	474.53	1.16
KLMA_40153	fmo1	thiol-specific monooxygenase	1292.13	945.26	1087.79	356.61	375.74	395.01	-1.56
KLMA_40154	GIS3	hypothetical protein	313.92	507.87	393.45	284.28	256.96	286.10	-0.56
KLMA_40155	IOC2	ISWI one complex protein 2	450.30	500.04	426.34	340.63	223.26	269.68	-0.72
KLMA_40156	KIN1	serine/threonine-protein kinase KIN2	1347.38	1742.86	1375.26	1278.41	1187.05	1293.07	-0.25
KLMA_40157	HRT3	F-box protein HRT3	83.48	39.15	51.16	42.05	64.03	60.50	-0.06
KLMA_40158	CHA4	activatory protein CHA4	92.88	136.48	121.81	301.10	173.55	187.56	0.91
KLMA_40159	ECM18	protein ECM18	262.19	281.90	238.75	338.11	386.70	293.88	0.38
KLMA_40160	SWF1	palmitoyltransferase SWF1	190.47	166.68	194.90	206.06	210.62	245.48	0.26
KLMA_40161	ARO1	pentafunctional AROM polypeptide	6505.31	4523.82	6195.39	2432.34	3551.04	3459.14	-0.87
KLMA_40162	MTC5	WD repeat-containing protein YDR128W	467.94	514.58	468.98	603.88	446.51	462.43	0.06
KLMA_40163	ERG27	3-keto-steroid reductase	392.69	382.58	456.80	207.74	267.91	267.09	-0.73
KLMA_40164	SAC6	fimbrin	3021.62	3491.31	2981.97	4304.54	3979.02	4133.34	0.39
KLMA_40166	CDC45	cell division control protein 45	152.84	195.76	188.81	219.52	173.55	190.16	0.12
KLMA_40167	SEN2	tRNA-splicing endonuclease subunit SEN2	208.10	182.34	204.65	322.97	259.48	294.74	0.56
KLMA_40168	MDN1	midasin	3809.36	2726.15	3069.68	1296.91	1152.51	950.79	-1.50
KLMA_40169	REX3	RNA exonuclease 3	224.56	279.66	257.02	304.46	232.52	280.05	0.10
KLMA_40170		filament protein FIN1	83.48	81.66	75.52	159.80	127.21	87.30	0.64

Locus_tag	UniProt_gene	Product	Unique exon reads						log2 Fold Change
			KmWT.1	KmWT.2	KmWT.3	Kmmig1.1	Kmmig1.2	Kmmig1.3	
KLMA_40171		F-box protein YDR131C	355.07	265.12	299.66	380.16	306.66	345.74	0.17
KLMA_40172		uncharacterized protein YDR132C	363.30	328.88	349.60	1069.83	983.17	1008.70	1.56
KLMA_40173	AHP1	peroxiredoxin type-2	1745.96	2815.64	1813.79	11726.04	6545.21	6773.07	1.97
KLMA_40174		uncharacterized cell wall protein YDR134C	60303.12	47554.95	51548.67	54246.60	56607.73	57026.59	0.07
KLMA_40175	HOG1	mitogen-activated protein kinase HOG1	1380.31	1544.86	1336.28	1198.51	1075.84	1160.83	-0.31
KLMA_40176	YCF1	metal resistance protein YCF1	1700.10	1893.88	1514.13	1950.42	2044.69	2214.47	0.28
KLMA_40177	YCS4	condensin complex subunit 1	741.88	691.33	683.37	608.93	589.73	499.60	-0.32
KLMA_40178	HEM15	ferrochelatase	542.01	559.33	499.43	626.59	738.01	856.57	0.47
KLMA_40179	ALE1	acyltransferase for lyso-phosphatidylethanol amine	2014.02	2371.54	2199.94	2447.48	1649.57	1685.49	-0.19
KLMA_40180	MED4	hypothetical protein	168.13	196.88	205.86	163.17	227.47	221.27	0.10
KLMA_40181		hypothetical protein	98.76	114.10	110.85	278.39	173.55	215.22	1.04
KLMA_40182	DCS1	hypothetical protein	1354.44	1312.18	1231.53	1571.10	1376.61	1504.84	0.19
KLMA_40183	SEC22	protein transport protein SEC22	798.32	927.36	884.36	565.19	711.89	769.27	-0.35
KLMA_40184	DDP1	diphosphoinositol polyphosphate phosphohydrolase DDP1	264.54	297.56	300.88	206.06	156.70	146.08	-0.76
KLMA_40185	GET4	UPF0363 protein YOR164C	1058.16	1006.79	931.87	741.81	1121.34	1044.14	-0.04
KLMA_40186	SEY1	protein SEY1	1024.06	1238.35	979.37	625.75	853.43	759.77	-0.53
KLMA_40187		non-homologous end-joining protein 1	129.33	137.59	126.69	133.73	143.22	132.25	0.06
KLMA_40188	SWT1	transcriptional protein SWT1	282.17	304.27	286.26	257.36	289.81	294.74	-0.05
KLMA_40189	RPS28	40S ribosomal protein S28	3403.73	2492.35	3413.19	1140.48	3219.95	1941.34	-0.56
KLMA_40190	RED1	protein RED1	332.73	234.92	292.35	423.05	253.59	219.55	0.06
KLMA_40191	GLN4	glutaminyl-tRNA synthetase	1745.96	2069.50	1991.64	1096.74	1527.41	1449.52	-0.51

Locus_tag	UniProt_gene	Product	Unique exon reads						log$_2$ Fold Change
			KmWT.1	KmWT.2	KmWT.3	Kmmig1.1	Kmmig1.2	Kmmig1.3	
KLMA_40192		translation machinery-associated protein 7	59.96	76.07	79.18	52.15	106.15	70.88	0.09
KLMA_40193	YPT6	GTP-binding protein YPT6	411.50	416.14	442.18	407.07	545.93	424.40	0.12
KLMA_40194	LCB5	sphingoid long chain base kinase 5	2403.19	2078.45	2253.53	1127.02	895.55	1112.42	-1.10
KLMA_40196	PTI1	protein PTI1	222.21	287.49	207.08	181.67	140.69	196.21	-0.47
KLMA_40198	UGA1	4-aminobutyrate aminotransferase	271.59	343.43	263.12	4649.37	2351.35	2021.72	3.36
KLMA_40199		putative magnesium-dependent phosphatase YER134C	324.50	290.85	356.91	624.91	440.62	496.14	0.68
KLMA_40200		probable inorganic polyphosphate/ATP-NAD kinase	338.61	403.83	314.28	841.06	592.26	563.56	0.92
KLMA_40201		zinc finger protein YPR013C	1698.93	1630.99	1504.39	1177.48	1029.51	926.59	-0.63
KLMA_40202	ENA5	sodium transport ATPase 5	144.61	132.00	137.65	1180.85	594.79	783.97	2.63
KLMA_40204		transcriptional regulatory protein UME6	290.40	431.80	381.27	221.20	199.67	173.74	-0.89
KLMA_40205	EBS1	protein EBS1	1206.30	1547.09	1394.75	878.07	785.19	898.06	-0.70
KLMA_40206	TOP3	DNA topoisomerase 3	103.46	114.10	110.85	97.56	78.35	117.55	-0.16
KLMA_40207	MSC2	probable zinc transporter MSC2	597.27	695.80	599.32	575.28	618.38	603.32	-0.08
KLMA_40208		uncharacterized protein YNL200C	122.28	192.41	160.79	223.72	200.51	213.50	0.42
KLMA_40209	GCR2	hypothetical protein	514.97	642.11	532.32	172.42	194.61	198.80	-1.58
KLMA_40210	SHS1	seventh homolog of septin 1	659.58	711.46	638.30	485.29	564.46	559.24	-0.32
KLMA_40211	WHI3	protein WHI3	2390.26	2170.18	2257.19	1639.22	1596.49	1472.86	-0.53
KLMA_40212		solute carrier family 2	16.46	27.97	20.71	586.22	281.39	306.85	4.17
KLMA_40213		MOG interacting and ectopic P-granules protein	105.82	148.78	110.85	1363.36	480.21	645.67	2.77
KLMA_40215	CDC13	cell division control protein 13	278.65	237.15	333.77	768.73	480.21	561.83	1.09

Locus_tag	UniProt_gene	Product	Unique exon reads						log_2 Fold Change
			KmWT.1	KmWT.2	KmWT.3	Kmmig1.1	Kmmig1.2	Kmmig1.3	
KLMA_40216		uncharacterized protein YNL193W	74.07	134.24	93.80	1173.28	531.60	724.33	3.01
KLMA_40217	CHS1	chitin synthase 1	1002.90	1305.47	918.47	3222.94	1846.71	2652.70	1.26
KLMA_40218	GAP1	glyceraldehyde-3-phosphate dehydrogenase 1	157427.70	116128.23	151667.69	19211.46	17515.94	18014.86	-2.96
KLMA_40220	ADH2	alcohol dehydrogenase 2	120173.56	70459.32	110542.52	906.66	487.79	413.16	-7.38
KLMA_40221	MRPS9	40S ribosomal protein S9	603.15	675.66	672.41	379.32	608.27	520.34	-0.37
KLMA_40222		uncharacterized protein YOL086W-A	79.95	61.53	57.25	98.40	90.99	86.44	0.47
KLMA_40223		uncharacterized WD repeat-containing protein YOL087C	801.85	860.24	721.13	735.93	782.66	811.63	-0.03
KLMA_40224	RPB5	DNA-directed RNA polymerases I	773.63	634.27	775.95	411.28	637.76	547.14	-0.45
KLMA_40225	RIB7	5-amino-6-(5-phosphoribosylamino)uracil reductase	11034.21	8264.59	11311.52	3882.33	2803.76	2761.61	-1.70
KLMA_40226	SPP381	pre-mRNA-splicing factor SPP381	835.94	897.16	940.39	253.16	200.51	226.46	-1.97
KLMA_40227		POU domain	1701.28	2211.57	1740.70	2394.49	1266.24	1552.38	-0.12
KLMA_40228	UTP10	U3 small nucleolar RNA-associated protein 10	3391.98	2395.03	3224.38	1074.03	1101.96	1098.59	-1.46
KLMA_40229	GMH1	protein GMH1	510.27	391.53	448.27	429.78	447.36	371.67	-0.11
KLMA_40230		hypothetical protein	31.74	42.51	28.02	151.39	76.67	123.60	1.78
KLMA_40231	SPO14	phospholipase D1	88.18	177.87	87.71	259.89	227.47	253.26	1.06
KLMA_40232	GZF3	protein GZF3	957.04	798.72	886.80	481.09	443.14	572.20	-0.82
KLMA_40233	DID2	vacuolar protein-sorting-associated protein 46	413.86	403.83	378.84	602.20	498.75	549.73	0.46
KLMA_40234	CCT7	T-complex protein 1 subunit eta	1812.97	1840.18	1783.34	1450.83	1690.01	1589.55	-0.20
KLMA_40235	MDV1	mitochondrial division protein 1	243.38	288.61	270.42	560.99	421.24	492.68	0.88
KLMA_40236	SPC34	DASH complex subunit SPC34	188.12	159.97	147.39	257.36	279.70	251.53	0.67
KLMA_40237	gcp	putative glycoprotein	496.16	506.75	498.21	1036.18	967.16	943.87	0.97

Locus_tag	UniProt_gene	Product	Unique exon reads						$\log_2$ Fold Change
			KmWT.1	KmWT.2	KmWT.3	Kmmig1.1	Kmmig1.2	Kmmig1.3	
		endopeptidase KAE1							
KLMA_40238	cys2	uncharacterized protein C106.17c	641.95	594.00	538.41	446.60	411.97	490.09	-0.40
KLMA_40239		37S ribosomal protein S28	790.09	823.33	808.84	597.99	888.81	675.06	-0.16
KLMA_40240	GAR1	H/ACA ribonucleoprotein complex subunit 1	2104.55	1405.03	1875.91	530.71	1011.82	852.25	-1.17
KLMA_40241		uncharacterized GTP-binding protein YDR336W	150.49	111.87	105.98	131.21	101.10	114.09	-0.09
KLMA_40242	MSN5	protein MSN5	1069.91	1184.65	1169.40	968.06	1069.95	1064.88	-0.14
KLMA_40243	RPF1	ribosome production factor 1	654.88	496.68	707.73	330.54	371.53	401.06	-0.75
KLMA_40244	PDR5	ATP-dependent permease PDR15	150.49	129.76	149.83	1427.28	636.07	769.27	2.72
KLMA_40245	PDR5	pleiotropic ABC efflux transporter of multiple drugs	781.86	611.90	669.97	715.74	774.24	860.03	0.19
KLMA_40246		hypothetical protein	137.56	98.44	148.61	369.22	409.44	426.13	1.65
KLMA_40247	RTC3	SDO1-like protein YHR087W	17.64	30.20	14.62	619.02	436.40	340.56	4.47
KLMA_40248	SWR1	helicase SWR1	616.08	591.77	661.44	536.60	503.80	524.66	-0.26
KLMA_40249	ERC1	uncharacterized transporter YHR032W	1883.52	1316.65	2006.25	1135.43	1232.54	1200.59	-0.54
KLMA_40250		hypothetical protein	16.46	14.54	17.05	10.93	6.74	4.32	-1.12
KLMA_40251	CIC1	proteasome-interacting protein CIC1	1432.04	1086.21	1291.21	499.59	724.53	603.32	-1.06
KLMA_40252	ELF1	transcription elongation factor 1	500.86	545.90	523.79	523.14	738.85	702.72	0.32
KLMA_40253	COX6	cytochrome c oxidase subunit 6	4494.81	4132.29	4647.15	1758.65	3102.84	2210.15	-0.91
KLMA_40254		calcipressin-like protein	109.34	126.41	125.47	52.99	51.39	61.37	-1.12
KLMA_40255	TMA22	translation machinery-associated protein 22	654.88	552.61	562.77	609.77	791.93	669.87	0.23
KLMA_40256	YPP1	cargo-transport protein YPP1	391.52	362.44	361.78	465.95	485.27	577.39	0.45

Locus_tag	UniProt_gene	Product	Unique exon reads						log$_2$ Fold Change
			KmWT.1	KmWT.2	KmWT.3	Kmmig1.1	Kmmig1.2	Kmmig1.3	
KLMA_40257	PMT6	dolichyl-phosphate-mannose--protein mannosyltransferase 6	1036.99	814.38	939.18	612.29	727.90	709.63	-0.44
KLMA_40258	ELP2	elongator complex protein 2	853.58	826.68	830.76	548.37	732.11	697.53	-0.34
KLMA_40259	ILV3	dihydroxy-acid dehydratase	7698.67	4076.36	6672.90	4004.28	8568.84	8555.37	0.20
KLMA_40260		hypothetical protein	0.00	0.00	0.00	18.50	3.37	8.64	5.56
KLMA_40261	TRA1	transcription-associated protein 1	2787.65	2182.49	2512.99	1726.69	1739.72	1644.86	-0.55
KLMA_40262	YPS7	aspartic proteinase yapsin-7	382.11	297.56	372.75	1317.94	979.80	984.50	1.64
KLMA_40263	SFB3	SED5-binding protein 3	1240.39	893.80	1164.53	349.88	503.80	477.12	-1.31
KLMA_40264		uncharacterized protein YDR348C	1131.05	1436.35	1225.44	1730.06	1049.73	1310.36	0.11
KLMA_40265	MRP1	37S ribosomal protein MRP1	744.24	646.58	718.69	380.16	661.34	543.68	-0.41
KLMA_40266		conserved hypothetical protein	88.18	96.20	54.82	117.75	100.25	106.32	0.44
KLMA_40267	OCH1	initiation-specific alpha-1	411.50	797.60	443.40	479.40	532.45	528.12	-0.10
KLMA_40268	PNC1	nicotinamidase	190.47	316.58	208.30	436.51	396.81	426.13	0.81
KLMA_40269		protein MTC2	230.44	204.71	185.16	350.72	311.72	333.64	0.68
KLMA_40270	KNS1	dual specificity protein kinase KNS1	1368.55	1685.81	1369.17	2114.42	1138.19	1205.77	0.01
KLMA_40271		zinc finger protein	0.00	0.00	0.00	55.51	21.90	34.57	7.44
KLMA_40272		v-type proton ATPase catalytic subunit A	83.48	137.59	109.63	580.33	256.11	382.91	1.88
KLMA_40273	NUP49	nuclear pore complex subunit	1074.62	837.87	993.99	597.99	799.51	667.28	-0.49
KLMA_40274	XRN1	5'-3' exoribonuclease 1	4332.56	4420.91	4317.04	2007.61	2512.27	2325.11	-0.93
KLMA_40275	CWC26	pre-mRNA-splicing factor CWC26	119.92	98.44	95.01	216.99	170.18	161.63	0.81
KLMA_40276	SAE2	hypothetical protein	48.20	79.42	80.40	166.53	138.17	123.60	1.04
KLMA_40277	NUF2	probable kinetochore protein NUF2	191.64	187.93	213.17	224.56	223.26	193.62	0.11
KLMA_40278	PRP11	pre-mRNA-splicing factor PRP11	253.96	217.02	214.39	227.09	214.83	227.32	-0.03

Locus_tag	UniProt_gene	Product	Unique exon reads						log2 Fold Change
			KmWT.1	KmWT.2	KmWT.3	Kmmig1.1	Kmmig1.2	Kmmig1.3	
KLMA_40279		hypothetical protein	1124.00	1153.33	1106.06	731.72	1145.77	953.38	-0.26
KLMA_40281	MPS3	spindle pole body assembly component MPS3	190.47	162.20	157.14	239.70	213.99	215.22	0.39
KLMA_40282		uncharacterized protein YJL016W/YJL017W	556.12	483.26	551.81	2481.12	2539.23	2599.11	2.26
KLMA_40283	CCT3	T-complex protein 1 subunit gamma	1309.76	1346.86	1322.89	807.42	1204.74	1089.95	-0.36
KLMA_40284	BUB1	checkpoint serine/threonine-protein kinase BUB1	208.10	239.39	221.70	301.10	294.02	354.38	0.50
KLMA_40285	VTC4	vacuolar transporter chaperone 4	922.95	1077.26	896.54	1470.17	1285.62	1465.94	0.54
KLMA_40286	RPC17	DNA-directed RNA polymerase III subunit RPC9	34.10	29.08	28.02	10.93	19.38	19.02	-0.89
KLMA_40287	CRH1	probable glycosidase CRH1	2292.67	2935.34	1843.03	1883.97	1796.16	2204.97	-0.27
KLMA_40288		hypothetical protein	22.34	36.92	17.05	108.50	108.68	106.32	2.08
KLMA_40289	APS1	AP-1 complex subunit sigma-1	253.96	210.31	227.79	148.87	276.33	215.22	-0.11
KLMA_40290	DPH5	diphthine synthase	917.07	828.92	939.18	629.95	636.07	611.10	-0.52
KLMA_40291		putative uncharacterized protein YLR173W	405.63	431.80	431.22	607.24	422.08	412.30	0.18
KLMA_40292		hypothetical protein	479.70	529.12	538.41	423.05	442.30	446.87	-0.24
KLMA_40293		leucine-rich repeat-containing protein 6	15.28	11.19	34.11	136.25	58.13	72.61	2.15
KLMA_40294	SYM1	protein SYM1	464.41	307.63	348.38	438.19	347.94	289.56	-0.06
KLMA_40295		ABC1 family protein YLR253W	498.51	614.14	511.61	412.96	534.13	482.31	-0.18
KLMA_40296		uncharacterized protein YGL159W	1201.59	814.38	1095.10	480.24	736.33	751.12	-0.66
KLMA_40297	HAP1	hap1 transcriptional regulatory prottein	4013.94	3708.33	4606.95	795.64	668.08	761.50	-2.47
KLMA_40298	AIM14	uncharacterized membrane protein YGL160W	438.55	438.51	456.80	459.22	710.21	693.21	0.48
KLMA_40301		UPF0067 GAF domain-containing	96.41	97.32	129.12	121.95	144.06	112.37	0.23

Locus_tag	UniProt_gene	Product	Unique exon reads						log$_2$ Fold Change
			KmWT.1	KmWT.2	KmWT.3	Kmmig1.1	Kmmig1.2	Kmmig1.3	
		protein YKL069W							
KLMA_40302	ERB1	ribosome biogenesis protein ERB1	1497.88	1247.30	1542.15	545.01	567.83	630.98	-1.30
KLMA_40303	CSM3	chromosome segregation in meiosis protein 3	81.13	138.71	77.96	84.95	74.14	95.94	-0.23
KLMA_40304		hypothetical protein	77.60	68.24	107.20	31.96	33.70	17.29	-1.61
KLMA_40305	NUP116	nucleoporin NUP116/NSP116	3489.56	3684.83	3513.08	1444.94	1797.85	1778.84	-1.09
KLMA_40306	IOC4	ISWI one complex protein 4	239.85	252.82	274.08	247.27	291.50	287.83	0.11
KLMA_40307	MCM1	pheromone receptor transcription factor	915.89	889.33	794.22	369.22	365.64	325.00	-1.29
KLMA_40308		hypothetical protein	10737.93	7585.57	10371.13	4768.80	3214.89	2472.92	-1.46
KLMA_40309	YET1	endoplasmic reticulum transmembrane protein 1	298.64	363.56	328.89	572.76	377.43	481.44	0.53
KLMA_40310	ARA2	D-arabinose 1-dehydrogenase	928.83	692.44	874.61	915.07	789.40	772.73	-0.01
KLMA_40311	SUB1	RNA polymerase II transcriptional coactivator SUB1	748.94	703.63	782.04	613.97	585.52	639.62	-0.28
KLMA_40312	MNR2	manganese resistance protein MNR2	1589.58	1635.47	1749.23	1591.28	1476.86	1607.70	-0.09
KLMA_40313	CCS1	superoxide dismutase 1 copper chaperone	379.76	410.54	384.93	484.45	476.00	501.33	0.31
KLMA_40314		hypothetical protein	196.35	220.37	216.83	269.98	209.78	267.09	0.24
KLMA_40315	MSN2	hypothetical protein	892.38	891.56	839.29	829.28	643.65	668.15	-0.29
KLMA_40316	ELM1	probable myosin light chain kinase DDB_G0271550	32.92	35.80	34.11	95.88	103.62	101.99	1.55
KLMA_40317	MIH1	m-phase inducer phosphatase	542.01	655.53	572.52	788.07	759.91	821.14	0.42
KLMA_40318	IMP2	mitochondrial inner membrane protease subunit 2	95.23	91.73	109.63	53.83	43.81	34.57	-1.16
KLMA_40319		uncharacterized membrane protein YMR034C	3347.30	2773.13	3325.48	2679.61	1477.71	1565.34	-0.72

Locus_tag	UniProt_gene	Product	Unique exon reads						log$_2$ Fold Change
			KmWT.1	KmWT.2	KmWT.3	Kmmig1.1	Kmmig1.2	Kmmig1.3	
KLMA_40320		uncharacterized protein YOL098C	733.65	804.31	783.26	646.77	655.45	672.47	-0.23
KLMA_40321	AEP3	ATPase expression protein 3	275.12	246.10	265.55	475.20	301.61	296.47	0.45
KLMA_40322	NCR1	niemann-Pick type C-related protein 1	723.07	967.63	808.84	1379.34	1334.48	1159.96	0.63
KLMA_40323	TFC8	transcription factor tau 60 kDa subunit	272.77	260.65	297.22	308.67	280.54	297.34	0.09
KLMA_40324	CHL1	ATP-dependent RNA helicase CHL1	264.54	300.92	267.99	387.73	321.83	269.68	0.23
KLMA_40325		hypothetical protein	650.18	672.31	651.70	461.74	473.47	417.48	-0.55
KLMA_40326		hypothetical protein	939.41	1141.02	1043.93	2359.17	1972.24	1714.88	0.95
KLMA_40327		37S ribosomal protein S16	226.92	228.20	238.75	93.36	244.32	175.46	-0.44
KLMA_40328	RRP12	ribosomal RNA-processing protein 12	2498.42	2314.49	2601.92	926.01	962.95	1010.43	-1.35
KLMA_40329	TAF3	transcription initiation factor TFIID subunit 3	252.78	240.51	208.30	309.51	273.81	287.83	0.31
KLMA_40330	RET3	coatomer subunit zeta	700.73	730.48	695.55	603.04	617.54	633.57	-0.20
KLMA_40331	TAE2	uncharacterized protein YPL009C	536.13	649.94	555.47	499.59	597.32	550.59	-0.08
KLMA_40332	PAT1	DNA topoisomerase 2-associated protein PAT1	2096.32	2059.44	2070.81	1118.61	1205.58	1324.19	-0.77
KLMA_40333		uncharacterized protein YCR075W-A	11.76	30.20	14.62	63.08	79.19	62.23	1.85
KLMA_40334		LMBR1 domain-containing protein 2 homolog	621.96	766.28	735.75	1170.75	1026.14	1108.97	0.64
KLMA_40335		pyruvate dehydrogenase kinase	105.82	132.00	102.32	432.30	278.86	373.40	1.67
KLMA_40336	ERS1	cystine transporter	166.95	138.71	158.36	270.82	246.00	279.19	0.78
KLMA_40337	SYG1	protein SYG1	1902.33	1184.65	1856.42	1446.62	1770.04	1724.39	0.00
KLMA_40338	HIS1	ATP phosphoribosyltransferase	1811.80	955.33	1587.22	1206.92	1992.46	1870.46	0.22
KLMA_40339	MET30	F-box protein MET30	412.68	418.38	501.87	247.27	264.54	312.90	-0.69

Locus_tag	UniProt_gene	Product	Unique exon reads						log2 Fold Change
			KmWT.1	KmWT.2	KmWT.3	Kmmig1.1	Kmmig1.2	Kmmig1.3	
KLMA_40340	AGE2	ADP-ribosylation factor GTPase-activating protein effector protein 2	705.44	591.77	661.44	547.53	611.64	566.15	-0.18
KLMA_40341		hypothetical protein	500.86	409.43	499.43	700.60	505.49	431.31	0.22
KLMA_40342	HOM3	aspartokinase	2219.78	1518.01	2153.65	1196.83	1541.73	1524.72	-0.47
KLMA_40343	CBR1	NADH-cytochrome b5 reductase 1	1422.63	1435.23	1458.10	926.01	1549.32	1312.09	-0.19
KLMA_40344	JHD1	jmjC domain-containing histone demethylation protein 1	276.30	283.02	305.75	219.52	196.30	204.85	-0.48
KLMA_40345	ulp2	ubiquitin-like-specific protease 2	333.91	300.92	364.22	699.76	441.46	485.77	0.70
KLMA_40346	SSM4	protein SSM4	1165.15	771.87	1101.19	1102.63	1219.91	1189.35	0.21
KLMA_40347	SSM4	protein SSM4	443.25	341.19	415.38	389.41	397.65	442.55	0.04
KLMA_40348		hypothetical protein	25.87	34.68	32.89	68.13	48.86	62.23	0.94
KLMA_40349	EMC5	ER membrane protein complex subunit 5	212.81	227.09	264.33	227.93	232.52	260.17	0.03
KLMA_40350	IRR1	cohesin subunit SCC3	612.55	594.00	616.37	502.95	395.96	483.17	-0.40
KLMA_40351		hypothetical protein	74.07	73.83	53.60	1176.64	589.73	632.71	3.57
KLMA_40352		hypothetical protein	52.91	69.36	49.94	95.88	86.78	79.52	0.60
KLMA_40353	WSS1	DNA damage response protein WSS1	71.72	118.58	113.29	212.79	120.47	153.85	0.68
KLMA_40354	NSG2	protein NSG2	1537.85	1269.67	1492.20	1668.66	1721.18	1602.51	0.22
KLMA_40355	IGO2	protein IGO2	340.96	357.97	334.98	565.19	556.88	414.03	0.57
KLMA_40356	ECM14	protein ECM14	1493.18	1428.52	1416.68	1440.73	1429.68	1566.21	0.03
KLMA_40357		protein PGA1	69.37	89.49	68.22	107.66	80.88	92.49	0.31
KLMA_40358		protein ASI2	90.53	95.09	115.72	207.74	231.68	197.07	1.08
KLMA_40359		putative agmatinase 2	146.97	175.63	192.46	1094.22	690.83	781.38	2.32
KLMA_40360	LPE10	mitochondrial inner membrane magnesium transporter LPE10	169.30	172.27	146.18	228.77	211.46	223.00	0.44

Locus_tag	UniProt_gene	Product	Unique exon reads						log $_2$ Fold Change
			KmWT.1	KmWT.2	KmWT.3	Kmmig1.1	Kmmig1.2	Kmmig1.3	
KLMA_40361	FTH1	iron transporter FTH1	961.75	976.58	989.12	1085.81	978.96	874.73	0.01
KLMA_40362	DUR1,2	urea amidolyase	510.27	511.22	460.45	709.01	715.26	744.21	0.55
KLMA_40363	PAN3	PAB-dependent poly(A)-specific ribonuclease subunit PAN3	466.76	442.99	500.65	440.71	524.02	535.90	0.09
KLMA_40364		uncharacterized oxidoreductase YIR035C	605.50	592.88	607.85	1076.56	944.42	826.32	0.66
KLMA_40365		uridylate kinase	839.47	909.46	868.52	549.21	689.15	590.35	-0.52
KLMA_40366		hypothetical protein	322.15	345.66	317.93	269.98	222.41	267.95	-0.37
KLMA_40367	CDC16	hypothetical protein	462.06	541.43	489.69	258.21	331.94	344.01	-0.68
KLMA_40368	LYS1	saccharopine dehydrogenase [NAD+	1310.94	1106.35	1179.15	1777.16	2878.74	2856.69	1.06
KLMA_40369	MAK11	protein MAK11	733.65	843.46	803.96	381.00	418.71	406.25	-0.98
KLMA_40370		hypothetical protein	496.16	573.87	477.51	919.28	659.66	763.22	0.60
KLMA_40371	TMA108	protein TMA108	590.22	583.94	622.46	443.24	525.71	543.68	-0.25
KLMA_40372	APJ1	J domain-containing protein APJ1	1634.26	1607.50	1604.27	1762.02	1200.53	1225.65	-0.21
KLMA_40373	OM45	hypothetical protein	149.32	403.83	171.76	1644.27	782.66	1137.49	2.30
KLMA_40374	MKS1	negative regulator of RAS-cAMP pathway	1200.42	1257.36	1197.42	676.21	507.17	487.50	-1.13
KLMA_40375	IMP4	U3 small nucleolar ribonucleoprotein protein IMP4	292.76	312.10	347.17	105.13	114.58	129.65	-1.45
KLMA_40376		viable in a HAL3 SIT4 background protein 2	951.16	988.89	863.65	576.13	519.81	704.45	-0.64
KLMA_40377		hypothetical protein	3.53	4.47	10.96	1.68	4.21	3.46	-1.01
KLMA_40378	FMS1	polyamine oxidase FMS1	585.51	658.89	632.21	279.23	215.67	218.68	-1.39
KLMA_40379	MAC1	metal-binding activator 1	550.24	479.90	571.30	304.46	326.04	350.93	-0.71
KLMA_40380	UBC7	ubiquitin-conjugating enzyme E2-18 kDa	95.23	64.88	79.18	54.67	78.35	92.49	-0.09
KLMA_40381	DCW1	mannan endo-1	1495.53	1547.09	1240.05	2233.01	1919.16	2201.51	0.57

Locus_tag	UniProt_gene	Product	Unique exon reads						log2 Fold Change
			KmWT.1	KmWT.2	KmWT.3	Kmmig1.1	Kmmig1.2	Kmmig1.3	
KLMA_40382		uncharacterized protein YKL047W	205.75	232.68	209.52	418.01	388.38	327.59	0.81
KLMA_40383	BLI1	hypothetical protein	29.39	25.73	36.54	62.24	70.77	54.45	1.04
KLMA_40384	MSS1	tRNA modification GTPase MSS1	259.84	278.54	324.02	428.10	465.89	401.92	0.59
KLMA_40385	MRPL3	54S ribosomal protein L3	1118.12	1069.43	1050.02	539.96	884.60	752.85	-0.57
KLMA_40386		hypothetical protein	61.14	55.93	70.65	74.01	84.25	64.83	0.25
KLMA_40387		peroxisome assembly protein 12	236.32	284.14	265.55	221.20	287.28	274.86	-0.01
KLMA_40388	FBA1	fructose-bisphosphate aldolase	188947.80	117925.90	173793.73	15620.14	27474.87	25787.99	-2.80
KLMA_40389		conserved hypothetical membrane protein	4.70	4.47	12.18	8.41	4.21	2.59	-0.48
KLMA_40390	TRM13	tRNA guanosine-2'-O-methyltransferase TRM13	480.87	428.44	411.73	241.38	213.99	191.89	-1.03
KLMA_40391	MDH2	malate dehydrogenase	4488.93	4477.96	3594.69	10792.46	8172.87	7965.02	1.10
KLMA_40392	BXI1	uncharacterized vacuolar membrane protein YNL305C	958.22	730.48	948.92	497.91	497.90	401.06	-0.92
KLMA_40393	RPL25	ribosomal protein L23	9625.69	8573.34	9853.42	3079.96	6309.31	5307.13	-0.93
KLMA_40394	MRPS18	37S ribosomal protein S18	579.63	551.49	578.61	269.98	471.79	370.81	-0.62
KLMA_40395	MCK1	protein kinase MCK1	1280.37	991.12	1288.78	1096.74	1329.43	1375.19	0.09
KLMA_40396	VPS68	vacuolar protein sorting-associated protein 68	598.45	545.90	531.10	945.35	769.18	772.73	0.57
KLMA_40397	KRI1	protein KRI1	724.25	591.77	710.17	624.07	579.62	622.33	-0.15
KLMA_40398	RFA2	replication factor A protein 2	806.55	824.45	817.36	760.32	716.95	693.21	-0.17
KLMA_40399	GAS4	glycolipid-anchored surface protein 4 precursor	104.64	71.59	113.29	216.15	192.93	197.94	1.07
KLMA_40400	SKP2	F-box protein YNL311C	704.26	563.80	674.84	718.26	755.70	748.53	0.19
KLMA_40401	ZIM17	DNL zinc finger domain-containing	138.74	172.27	172.97	142.98	124.69	132.25	-0.28

Locus_tag	UniProt_gene	Product	Unique exon reads						log2 Fold Change
			KmWT.1	KmWT.2	KmWT.3	Kmmig1.1	Kmmig1.2	Kmmig1.3	
		protein							
KLMA_40402		protein STB1	257.48	277.43	242.41	298.58	236.74	268.81	0.05
KLMA_40403	VTC1	vacuolar transporter chaperone 1	513.79	557.09	544.50	1960.51	1042.14	950.79	1.29
KLMA_40404	ALD5	aldehyde dehydrogenase 5	2271.51	2316.73	2450.87	430.62	97.73	137.43	-3.40
KLMA_40405	RPS24B	40S ribosomal protein S24	16055.75	13025.57	15153.49	4858.80	10224.30	7995.27	-0.94
KLMA_40406	PTP3	tyrosine-protein phosphatase 3	641.95	731.60	722.35	715.74	556.03	725.19	-0.07
KLMA_40407	MAM33	mitochondrial acidic protein MAM33	991.14	1233.87	1041.50	428.94	556.03	471.07	-1.17
KLMA_40408		uncharacterized protein YER077C	112.87	154.37	155.92	349.04	245.16	223.00	0.95
KLMA_40409	PCI8	COP9 signalosome complex subunit 11	171.66	189.05	194.90	415.48	275.49	299.07	0.83
KLMA_40410	ICP55	uncharacterized peptidase YER078C	241.02	252.82	231.44	306.15	368.16	309.44	0.44
KLMA_40411	HOP1	meiosis-specific protein HOP1	43.50	48.10	36.54	243.91	101.10	121.87	1.86
KLMA_40412		uncharacterized protein YER079W	439.72	404.95	373.96	481.93	451.57	459.84	0.19
KLMA_40413	AIM9	uncharacterized protein YER080W	803.02	947.50	926.99	540.80	835.74	859.17	-0.26
KLMA_40414	PNT1	pentamidine resistance factor	285.70	257.29	238.75	168.21	282.23	267.09	-0.12
KLMA_40415	HRK1	serine/threonine-protein kinase HRK1	3935.16	3043.85	3878.51	1330.55	1144.93	1108.97	-1.60
KLMA_40416	PAC1	nuclear distribution protein PAC1	162.25	162.20	188.81	85.79	92.67	115.82	-0.80
KLMA_40417	VPH1	v-type proton ATPase subunit a	5310.77	4122.23	4898.09	4385.28	4530.84	4583.67	-0.09
KLMA_40418	ERG12	mevalonate kinase	1112.24	885.97	1106.06	535.75	518.12	522.07	-0.98
KLMA_40419	FSF1	probable mitochondrial transport protein FSF1	2302.08	927.36	2044.02	678.73	1235.07	1293.94	-0.72
KLMA_40420	TY1B-DR6	conserved hypothetical protein	51.73	23.49	64.56	47.10	38.75	49.27	-0.05
KLMA_40421		hypothetical protein	44.68	24.61	28.02	64.76	33.70	36.30	0.47
KLMA_40422	YTM1	ribosome biogenesis protein YTM1	1442.62	1126.48	1607.93	532.39	642.81	649.99	-1.19

Locus_tag	UniProt_gene	Product	Unique exon reads						log_2 Fold Change
			KmWT.1	KmWT.2	KmWT.3	Kmmig1.1	Kmmig1.2	Kmmig1.3	
KLMA_40423		uncharacterized protein YMR209C	222.21	233.80	221.70	253.16	322.67	315.49	0.39
KLMA_40424	TPO4	uncharacterized MFS-type transporter C530.15c	721.90	789.77	712.60	1605.58	1120.49	883.37	0.70
KLMA_40425	DML1	protein DML1	186.94	164.44	171.76	281.75	242.63	258.44	0.58
KLMA_40426	miaA	tRNA isopentenyltransferas e	255.13	259.53	193.68	323.81	304.13	262.76	0.33
KLMA_40427	RIM20	pH-response regulator protein palA/RIM20	560.82	627.56	617.59	539.12	497.90	523.80	-0.21
KLMA_40428	EFR3	protein EFR3	908.84	833.39	834.42	649.30	943.57	828.05	-0.09
KLMA_40429	CEF1	pre-mRNA-splicing factor CEF1	324.50	321.05	354.47	554.26	400.18	420.08	0.46
KLMA_40430	DBP8	ATP-dependent RNA helicase DBP8	373.88	337.83	416.60	285.96	178.61	271.41	-0.62
KLMA_40431	NOP19	uncharacterized protein YGR251W	168.13	121.93	105.98	72.33	66.56	57.91	-1.01
KLMA_40432	NMD3	60S ribosomal export protein NMD3	1579.00	1642.18	1670.05	602.20	673.98	712.23	-1.30
KLMA_40433	ATG7	autophagy-related protein 7	318.62	456.41	278.95	793.12	636.91	651.72	0.98
KLMA_40434	GCN5	histone acetyltransferase GCN5	445.60	393.76	471.41	418.85	406.07	426.99	-0.07
KLMA_40435	PUP2	proteasome component PUP2	788.91	1081.74	793.00	858.72	947.79	919.67	0.03
KLMA_40436	ERG4	delta(24(24(1)))-sterol reductase	2356.16	2368.18	2170.70	1095.06	1490.34	1570.53	-0.73
KLMA_40437		hypothetical protein	131.68	116.34	179.06	205.22	187.03	141.75	0.32
KLMA_40438		membrane-associated progesterone receptor	157.55	198.00	170.54	247.27	156.70	168.55	0.12
KLMA_40439	Fbxl7	hypothetical protein	1781.23	2199.27	1777.25	2263.29	1834.07	1873.05	0.05
KLMA_40440	VMA6	v-type proton ATPase subunit d	2613.64	2125.44	2231.61	1249.81	1925.06	1707.10	-0.51
KLMA_40441	COG8	conserved oligomeric Golgi complex component	460.89	482.14	505.52	315.40	326.88	284.37	-0.64

Locus_tag	UniProt_gene	Product	Unique exon reads						log2 Fold Change
			KmWT.1	KmWT.2	KmWT.3	Kmmig1.1	Kmmig1.2	Kmmig1.3	
KLMA_40442	TCB3	tricalbin-3	3729.41	4580.87	3901.66	3714.12	3646.24	3837.73	-0.13
KLMA_40443	RPL6B	60S ribosomal protein L6-B	21435.88	17527.02	20831.18	7755.40	14259.77	11080.15	-0.85
KLMA_40444	FPR3	FK506-binding protein 3	3176.82	2699.30	3101.35	1191.78	1634.41	1465.08	-1.06
KLMA_40445	HMG1	3-hydroxy-3-methylglutaryl-coenzyme A reductase 1	7811.54	4982.47	7931.22	1536.61	2882.11	2393.40	-1.61
KLMA_40446		conserved hypothetical membrane protein	7.05	5.59	4.87	79.06	20.22	12.10	2.67
KLMA_40447	WAR1	weak acid resistance protein 1	581.99	529.12	563.99	719.95	630.17	677.65	0.28
KLMA_40448	BET5	transport protein particle 18 kDa subunit	75.25	74.95	73.09	58.03	74.98	63.10	-0.19
KLMA_40449	ENT3	epsin-3	586.69	576.11	568.86	1123.65	1002.55	1034.63	0.87
KLMA_40450		uncharacterized protein YLR407W	479.70	513.46	472.63	795.64	540.03	611.10	0.41
KLMA_40451	RXT3	transcriptional regulatory protein RXT3	28.22	63.76	48.73	100.09	87.62	82.98	0.94
KLMA_40452	RPL31	60S ribosomal protein L31-A	9417.59	7360.72	8515.92	3868.03	7821.56	5841.30	-0.53
KLMA_40453	DUS4	tRNA-dihydrouridine synthase 4	208.10	184.58	225.35	160.64	161.76	133.11	-0.44
KLMA_40454	BRE1	E3 ubiquitin-protein ligase BRE1	1018.18	1042.58	1160.87	875.54	860.17	808.17	-0.34
KLMA_40455		UPF0592 protein YDL073W	462.06	523.53	453.14	407.07	330.25	372.54	-0.37
KLMA_40456		hypothetical protein	129.33	99.56	97.45	111.02	228.31	200.53	0.73
KLMA_40457	SFP1	zinc finger protein SFP1	1890.57	2033.71	1983.11	483.61	489.48	523.80	-1.98
KLMA_40458	DUS3	tRNA-dihydrouridine synthase 3	505.56	491.09	534.76	219.52	227.47	271.41	-1.09
KLMA_40459	YET3	endoplasmic reticulum transmembrane protein 3	228.09	356.85	203.43	646.77	513.91	554.05	1.12
KLMA_40460	BDF1	bromodomain-	3006.34	2933.10	2821.18	3329.75	2085.97	2039.87	-0.23

Locus_tag	UniProt_gene	Product	Unique exon reads						log2 Fold Change
			KmWT.1	KmWT.2	KmWT.3	Kmmig1.1	Kmmig1.2	Kmmig1.3	
		containing factor 1							
KLMA_40461	GNT1-A	glucose N-acetyltransferase 1-A	504.39	502.27	417.82	393.62	454.94	453.79	-0.13
KLMA_40462	YEA4	UDP-N-acetylglucosamine transporter YEA4	483.22	353.49	430.00	325.49	443.14	427.86	-0.08
KLMA_40463	SKI2	antiviral helicase SKI2	800.67	823.33	764.98	683.78	832.37	810.76	-0.04
KLMA_40464		beta tubulin	109.34	120.81	154.70	221.20	175.24	155.58	0.52
KLMA_40465	AFG2	ATPase family gene 2 protein	551.42	604.07	562.77	774.62	530.76	682.84	0.21
KLMA_40466	VPS33	vacuolar protein sorting-associated protein 33	189.29	221.49	225.35	391.09	423.77	435.63	0.98
KLMA_40467	COX9	cytochrome c oxidase subunit 7A	1235.69	1145.50	1242.49	593.79	953.68	686.30	-0.70
KLMA_40468		cytochrome c oxidase polypeptide VIII	409.15	429.56	449.49	290.17	439.77	293.88	-0.33
KLMA_40469	CST9	hypothetical protein	42.33	60.41	47.51	322.97	177.76	191.89	2.20
KLMA_40470	IDP1	isocitrate dehydrogenase [NADP]	1101.66	1252.89	1242.49	2642.61	3911.62	4014.06	1.55
KLMA_40471	DPM1	dolichol-phosphate mannosyltransferase	2397.31	2152.28	2324.18	873.86	1220.75	1289.62	-1.02
KLMA_40472	GDB1	glycogen debranching enzyme	1840.02	2652.32	2141.47	3213.69	2838.31	3217.12	0.48
KLMA_40473	ATG13	autophagy-related protein 13	537.31	581.70	514.05	938.62	757.39	881.64	0.66
KLMA_40474	PZF1	transcription factor IIIA	186.94	193.53	226.57	137.09	107.84	111.50	-0.77
KLMA_40475	RPO26	DNA-directed RNA polymerases I	552.59	422.85	567.65	243.91	358.90	329.32	-0.73
KLMA_40476		myosin light chain 2	115.22	157.73	148.61	312.87	330.25	286.10	1.14
KLMA_40477	SKI3	superkiller protein 3	815.96	831.16	839.29	710.69	647.86	714.82	-0.26
KLMA_40478	RPC82	DNA-directed RNA polymerase III subunit RPC3	938.23	1086.21	1151.13	322.13	413.66	421.80	-1.46
KLMA_40479	QCR2	cytochrome b-c1 complex subunit 2	4841.65	4958.98	4835.96	1931.07	3681.63	2971.65	-0.77
KLMA_40480	SCH9	serine/threonine-protein kinase SCH9	2066.93	2058.32	1913.68	1312.89	1123.86	1160.83	-0.75

Locus_tag	UniProt_gene	Product	Unique exon reads						log_2 Fold Change
			KmWT.1	KmWT.2	KmWT.3	Kmmig1.1	Kmmig1.2	Kmmig1.3	
KLMA_40481	MNL1	ER degradation-enhancing alpha-mannosidase-like protein 1	402.10	472.07	430.00	502.95	468.42	492.68	0.17
KLMA_40482	RPS4B	40S ribosomal protein S4	29763.57	26671.98	28546.79	9577.98	19189.95	16140.94	-0.92
KLMA_40483		uncharacterized protein YHR202W	385.64	525.77	420.25	711.54	705.15	652.59	0.64
KLMA_40484		hypothetical protein	7.05	2.24	2.44	107.66	42.12	55.32	4.13
KLMA_40485	MGM101	mitochondrial genome maintenance protein MGM101	232.79	219.26	211.95	293.53	331.94	282.64	0.45
KLMA_40486	PMT4	dolichyl-phosphate-mannose--protein mannosyltransferase 4	2862.90	2503.54	2622.63	1435.69	2113.78	2111.62	-0.50
KLMA_40487		uncharacterized protein YJR142W	315.10	300.92	277.73	519.77	479.37	464.16	0.71
KLMA_40488	PPX1	exopolyphosphatase	235.15	276.31	270.42	323.81	320.98	297.34	0.27
KLMA_40489		uncharacterized protein YJR141W	123.45	177.87	146.18	281.75	211.46	247.21	0.73
KLMA_40490	HIR3	histone transcription regulator 3 homolog	784.21	873.67	823.45	812.46	676.51	722.60	-0.17
KLMA_40491	HOM6	homoserine dehydrogenase	3534.24	2287.64	3196.36	2074.89	3604.96	3307.02	0.00
KLMA_40492	IML1	vacuolar membrane-associated protein IML1	557.30	703.63	684.59	785.55	638.60	671.60	0.11
KLMA_40493	RPN10	26S proteasome regulatory subunit RPN10	409.15	557.09	423.91	580.33	589.73	584.30	0.34
KLMA_40494	MET5	sulfite reductase [NADPH] subunit beta	16301.48	12933.84	15084.06	4850.39	7352.30	8434.36	-1.10
KLMA_40495	AIM18	uncharacterized protein YHR199C	409.15	553.73	430.00	813.30	513.91	597.27	0.47
KLMA_40496		pseudouridine-metabolizing bifunctional protein C1861.05	319.80	496.68	328.89	793.96	629.33	628.39	0.84
KLMA_40497	TTI2	TEL2-interacting protein 2	218.69	212.54	185.16	288.48	219.89	247.21	0.29
KLMA_40498	TIM8	mitochondrial import inner membrane	116.40	102.92	103.54	95.04	153.33	132.25	0.24

Locus_tag	UniProt_gene	Product	Unique exon reads						log$_2$ Fold Change
			KmWT.1	KmWT.2	KmWT.3	Kmmig1.1	Kmmig1.2	Kmmig1.3	
		translocase subunit TIM8							
KLMA_40499		central kinetochore subunit MCM22	98.76	53.70	53.60	148.03	111.21	125.33	0.90
KLMA_40500	RIX1	pre-rRNA-processing protein RIX1	1453.20	1356.92	1533.62	698.08	714.42	725.19	-1.02
KLMA_40501	SGM1	protein SGM1	972.33	948.62	1142.60	576.97	451.57	449.46	-1.05
KLMA_40502	XPT1	xanthine phosphoribosyltransferase 1	1650.72	1267.43	1548.24	522.30	631.86	529.85	-1.41
KLMA_40503	UTP9	U3 small nucleolar RNA-associated protein 9	1441.44	1268.55	1677.36	636.68	839.95	775.33	-0.96
KLMA_40504	NMD5	nonsense-mediated mRNA decay protein 5	1289.77	1078.38	1163.31	989.93	989.91	1127.12	-0.18
KLMA_40505	MDM31	mitochondrial distribution and morphology protein 31	313.92	347.90	291.13	243.07	277.18	343.15	-0.14
KLMA_40506	EGD2	nascent polypeptide-associated complex subunit alpha	3229.73	2604.22	3285.29	1410.46	2674.87	2236.08	-0.53
KLMA_40507		uncharacterized membrane protein YHR192W	288.05	249.46	258.24	260.73	380.80	349.20	0.32
KLMA_40508	CTF8	chromosome transmission fidelity protein 8	36.45	31.32	28.02	18.50	32.01	32.85	-0.20
KLMA_40509	MNS1	endoplasmic reticulum mannosyl-oligosaccharide 1	418.56	477.66	352.04	414.64	513.07	540.22	0.23
KLMA_40510	STR2	cystathionine gamma-synthase	537.31	723.77	533.54	645.09	668.08	714.82	0.18
KLMA_40511		ATP synthase subunit J	232.79	228.20	247.28	127.84	224.10	130.52	-0.55
KLMA_40512	DAL2	allantoicase	197.52	215.90	229.01	257.36	277.18	267.09	0.32
KLMA_40513		uncharacterized protein YJR129C	65.84	111.87	93.80	79.90	116.26	114.09	0.19
KLMA_40514		hypothetical protein	185.77	136.48	118.16	173.26	199.67	171.14	0.30
KLMA_40515	TDA9	zinc finger protein YML081W	813.60	836.75	839.29	552.58	442.30	491.82	-0.74

Locus_tag	UniProt_gene	Product	Unique exon reads						log2 Fold Change
			KmWT.1	KmWT.2	KmWT.3	Kmmig1.1	Kmmig1.2	Kmmig1.3	
KLMA_40516	DUS1	tRNA-dihydrouridine synthase 1	350.37	344.54	342.29	276.71	282.23	256.71	-0.35
KLMA_40517		uncharacterized protein YML079W	182.24	193.53	177.85	282.60	195.45	212.63	0.32
KLMA_40518	VPS70	vacuolar protein sorting-associated protein 70	544.36	717.05	534.76	922.64	895.55	965.48	0.63
KLMA_40519	CPR3	peptidyl-prolyl cis-trans isomerase C	1555.49	1295.40	1352.12	1915.09	2572.93	2144.46	0.66
KLMA_40520	BLS1	hypothetical protein	11.76	15.66	14.62	38.69	30.33	27.66	1.20
KLMA_40521	VAM6	vacuolar morphogenesis protein 6	272.77	355.73	315.49	432.30	366.48	379.45	0.32
KLMA_40522	UTP21	U3 small nucleolar RNA-associated protein 21	1635.44	1457.60	1681.01	566.87	695.04	632.71	-1.33
KLMA_40523	VIP1	inositol hexakisphosphate and diphosphoinositol-pentakisphosphate kinase	1662.48	1438.58	1536.06	1309.53	1212.32	1309.50	-0.28
KLMA_40524	PRS1	ribose-phosphate pyrophosphokinase 1	3275.58	2887.24	3143.98	1460.08	1671.47	1631.90	-0.97
KLMA_40525	BER1	SRR1-like protein BER1	91.71	70.47	76.74	77.38	61.50	84.71	-0.10
KLMA_40526	FAS1	fatty acid synthase subunit beta	24057.76	29787.43	26886.48	6969.01	13573.15	13421.68	-1.25
KLMA_40527	LOT5	protein LOT5	552.59	747.26	641.95	818.35	445.67	359.57	-0.26
KLMA_40528	SPE1	ornithine decarboxylase	1459.08	976.58	1089.00	1066.46	1026.14	1064.02	-0.16
KLMA_40529		transcriptional regulatory protein ASH1	560.82	632.04	484.81	411.28	498.75	477.99	-0.27
KLMA_40530	MTR2	mRNA transport regulator MTR2	161.07	172.27	197.34	79.06	90.15	92.49	-1.02
KLMA_40531		uncharacterized protein YKL187C	21046.72	23750.07	22537.77	10879.09	10650.60	8769.73	-1.15
KLMA_40532	PUN1	cell membrane protein YLR414C	166.95	261.76	67.00	3015.20	1714.44	2572.32	3.88
KLMA_40533	PXA2	peroxisomal long-chain fatty acid import protein 1	49.38	62.64	38.98	480.24	353.00	467.62	3.10

Locus_tag	UniProt_gene	Product	Unique exon reads						log$_2$ Fold Change
			KmWT.1	KmWT.2	KmWT.3	Kmmig1.1	Kmmig1.2	Kmmig1.3	
KLMA_40534	VPS36	vacuolar protein-sorting-associated protein 36	268.07	286.37	313.06	373.43	283.91	341.42	0.20
KLMA_40535	HYM1	protein HYM1	383.29	429.56	327.68	453.33	396.81	413.16	0.15
KLMA_40536	NPY1	NADH pyrophosphatase	98.76	126.41	109.63	323.81	189.56	239.43	1.17
KLMA_40537	PGC1	probable glycerophosphodiester phosphodiesterase YPL206C	470.29	415.02	332.55	759.48	589.73	700.99	0.75
KLMA_40538	SGF73	SAGA-associated factor 73	603.15	585.05	574.96	627.43	465.89	506.51	-0.14
KLMA_40539	ALG2	alpha-1,3-mannosyltransferase ALG2	613.73	684.61	559.12	920.12	850.90	924.86	0.54
KLMA_40540		hypothetical protein	236.32	253.93	241.19	412.96	272.12	297.34	0.43
KLMA_40541		hypothetical protein	1253.33	753.97	1062.21	812.46	898.08	484.04	-0.48
KLMA_40542	RKM1	ribosomal N-lysine methyltransferase 1	281.00	321.05	259.46	337.26	276.33	260.17	0.02
KLMA_40543	IPL1	spindle assembly checkpoint kinase	148.14	162.20	148.61	328.85	246.85	288.69	0.91
KLMA_40544	MRH4	ATP-dependent RNA helicase MRH4	285.70	264.00	296.00	313.71	295.71	218.68	-0.03
KLMA_40545	SRP72	signal recognition particle subunit SRP72	1547.26	1440.82	1601.84	945.35	1477.71	1185.03	-0.35
KLMA_40546	NIP7	60S ribosome subunit biogenesis protein NIP7	733.65	618.61	661.44	376.79	448.20	398.47	-0.72
KLMA_40547	PUS1	tRNA pseudouridine synthase 1	832.42	852.41	829.54	377.64	443.99	398.47	-1.04
KLMA_40548	RSM23	37S ribosomal protein S23	810.08	832.28	891.67	464.26	705.15	588.62	-0.53
KLMA_40549	CEG1	mRNA-capping enzyme subunit alpha	514.97	553.73	500.65	541.64	516.44	561.83	0.05
KLMA_40550	SNT2	SANT domain-containing protein 2	345.66	368.04	376.40	889.00	544.24	582.57	0.89
KLMA_40551	LEA1	U2 small nuclear ribonucleoprotein A'	75.25	107.39	58.47	173.26	164.28	160.77	1.04
KLMA_40552	THI6	thiamine biosynthetic	203.40	231.56	216.83	305.30	277.18	222.14	0.30

Locus_tag	UniProt_gene	Product	Unique exon reads						log2 Fold Change
			KmWT.1	KmWT.2	KmWT.3	Kmmig1.1	Kmmig1.2	Kmmig1.3	
		bifunctional enzyme							
KLMA_40553	CBP3	protein CBP3	614.91	589.53	662.66	363.34	480.21	463.29	-0.51
KLMA_40554	ITC1	imitation switch two complex protein 1	763.05	752.85	812.49	782.18	893.87	801.26	0.09
KLMA_40555	BMS1	ribosome biogenesis protein BMS1	2186.86	2090.76	1910.02	732.56	811.31	796.93	-1.40
KLMA_40556	SAR1	small COPII coat GTPase SAR1	1343.86	1157.80	1300.96	1176.64	1779.31	1535.09	0.24
KLMA_40557		PHO85 cyclin-8	1795.34	2026.99	2001.38	1657.73	1555.21	1558.43	-0.29
KLMA_40558	RPL10A	60S ribosomal protein L10a	19943.88	15104.02	19462.00	6005.16	11314.47	9172.52	-1.04
KLMA_40559	MRM2	ribosomal RNA methyltransferase MRM2	135.21	219.26	164.45	181.67	156.70	159.04	-0.06
KLMA_40560	SEC27	coatomer subunit beta'	1330.92	1408.38	1240.05	672.01	959.58	919.67	-0.64
KLMA_40561		hypothetical protein	31.74	22.37	41.42	25.23	21.90	13.83	-0.64
KLMA_40562	FLC1	flavin carrier protein 1	3961.03	4200.53	4060.01	3534.97	2734.68	3026.97	-0.39
KLMA_40563		ATP-dependent protease La	812.43	733.83	925.78	871.34	723.69	834.97	-0.02
KLMA_40564		hypothetical protein	2.35	0.00	3.65	119.43	22.75	16.42	4.75
KLMA_40565	FMP40	UPF0061 protein FMP40	236.32	406.07	293.57	792.28	613.32	576.52	1.08
KLMA_40566	SRC1	src1p	1002.90	1162.28	1079.26	957.97	954.53	996.60	-0.16
KLMA_40567	TOM1	probable E3 ubiquitin-protein ligase TOM1	1387.36	1544.86	1403.28	1264.11	1101.96	1174.66	-0.29
KLMA_40568	RAD52	DNA repair and recombination protein RAD52	178.71	184.58	174.19	207.74	171.02	202.26	0.11
KLMA_40569	NDC1	nucleoporin NDC1	1026.41	996.72	1134.08	592.11	531.60	601.59	-0.87
KLMA_40570	NHX1	endosomal/prevacuolar sodium/hydrogen exchanger	1138.11	1281.97	1294.87	999.18	986.54	1031.17	-0.30
KLMA_40571	GUK1	guanylate kinase	1040.52	889.33	1107.28	323.81	454.94	421.80	-1.34
KLMA_40572	RCF1	HIG1 domain-containing protein YML030W	114.05	144.31	141.30	161.48	108.68	90.76	-0.15

Locus_tag	UniProt_gene	Product	Unique exon reads						log2 Fold Change
			KmWT.1	KmWT.2	KmWT.3	Kmmig1.1	Kmmig1.2	Kmmig1.3	
KLMA_40573	USA1	U1 SNP1-associating protein 1	1352.09	1259.60	1246.14	2777.18	1714.44	2247.32	0.80
KLMA_40574	TSA1	peroxiredoxin TSA1	3373.17	3427.55	2878.43	3286.86	3635.29	3028.69	0.04
KLMA_40575	PPN1	endopolyphosphatase	873.57	1007.90	1041.50	719.11	874.49	860.90	-0.25
KLMA_40576	YOX1	homeobox protein YOX1	425.61	364.68	370.31	258.21	338.68	358.71	-0.28
KLMA_40577	rps13	40S ribosomal protein S18	15244.50	12093.73	14404.34	5042.15	9895.74	7852.65	-0.87
KLMA_40578	YML6	54S ribosomal protein YmL6	637.24	613.02	628.55	269.14	412.81	400.20	-0.80
KLMA_40579	UTP6	U3 small nucleolar RNA-associated protein 6	672.52	706.99	695.55	339.79	369.85	309.44	-1.03
KLMA_40580	ADA2	transcriptional adapter 2	748.94	846.82	825.89	452.49	486.11	499.60	-0.75
KLMA_40581	RPS17A	40S ribosomal protein S17-B	7991.43	7262.28	8156.57	3230.51	6367.44	4562.92	-0.73
KLMA_40582		hypothetical protein	323.33	140.95	305.75	85.79	56.45	65.69	-1.89
KLMA_40583	DLD1	D-lactate dehydrogenase [cytochrome] 1	16457.85	8325.00	14880.63	370.91	483.58	504.78	-4.87
KLMA_40584		putative lipase YDR444W	213.98	289.73	297.22	225.40	196.30	177.19	-0.42
KLMA_40585	SSN2	mediator of RNA polymerase II transcription subunit 13	632.54	671.19	669.97	728.36	679.04	729.51	0.11
KLMA_40586	NSE5	non-structural maintenance of chromosome element 5	819.48	577.22	727.22	722.47	618.38	686.30	-0.07
KLMA_40587	APT1	adenine phosphoribosyltransferase	966.45	619.73	873.40	780.50	898.92	922.27	0.08
KLMA_40588	DOT1	histone-lysine N-methyltransferase	229.27	238.27	218.04	501.27	373.22	321.54	0.80
KLMA_40589	UNG1	uracil-DNA glycosylase	149.32	181.22	160.79	169.05	171.87	177.19	0.08
KLMA_40590		uncharacterized protein YML020W	349.19	423.97	367.87	548.37	411.97	431.31	0.29
KLMA_40591		hypothetical protein	118.75	95.09	65.78	147.19	158.39	146.08	0.69
KLMA_40592	OST6	dolichyl-	389.17	368.04	370.31	482.77	597.32	658.64	0.62

Locus_tag	UniProt_gene	Product	Unique exon reads						log2 Fold Change
			KmWT.1	KmWT.2	KmWT.3	Kmmig1.1	Kmmig1.2	Kmmig1.3	
KLMA_40593		diphosphooligosaccharide-protein glycosyltransferase subunit OST6 uncharacterized vacuolar membrane protein YML018C	2439.64	1760.76	2543.45	966.38	814.68	1019.94	-1.27
KLMA_40594	PSP2	protein PSP2	1067.56	1138.79	964.76	667.80	529.08	652.59	-0.78
KLMA_40595	GPI19	phosphatidylinositol N-acetylglucosaminyltransferase subunit GPI19	44.68	58.17	52.38	42.89	44.65	51.00	-0.17
KLMA_40596	PPZ1	serine/threonine-protein phosphatase PP-Z1	952.34	1204.79	892.89	1524.00	1470.12	1600.78	0.59
KLMA_40597	PPM1	leucine carboxyl methyltransferase 1	261.01	231.56	182.72	354.93	328.57	311.17	0.56
KLMA_40598	TAF11	transcription initiation factor TFIID subunit 11	243.38	218.14	275.30	245.59	323.51	247.21	0.15
KLMA_40599	GPI17	GPI transamidase component GPI17	1212.18	1087.33	1121.89	712.38	824.79	894.61	-0.49
KLMA_40600	TRM9	tRNA (uracil-5-)-methyltransferase TRM9	141.09	133.12	159.57	238.02	178.61	199.67	0.51
KLMA_40601	UBX2	UBX domain-containing protein 2	666.64	630.92	624.90	726.67	633.54	514.29	-0.04
KLMA_40602	NPL3	heterogeneous nuclear ribonucleoprotein A3	3959.85	3413.00	3602.00	1895.75	2133.15	2017.40	-0.86
KLMA_40603	CYM1	mitochondrial presequence protease	603.15	667.83	673.62	748.54	592.26	630.11	0.02
KLMA_40604	ERV25	endoplasmic reticulum vesicle protein 25	850.05	850.17	884.36	605.56	1077.53	814.22	-0.05
KLMA_40605	RAD33	DNA repair protein RAD33	130.51	120.81	125.47	331.38	248.53	274.86	1.18
KLMA_40606	SPT5	transcription elongation factor SPT5	1560.19	1456.48	1672.49	1225.42	1191.26	1146.13	-0.40
KLMA_40607		54S ribosomal protein L39	22.34	30.20	17.05	12.62	26.96	19.88	-0.23

Locus_tag	UniProt_gene	Product	Unique exon reads						log2 Fold Change
			KmWT.1	KmWT.2	KmWT.3	Kmmig1.1	Kmmig1.2	Kmmig1.3	
KLMA_40608	TIF35	eukaryotic translation initiation factor 3 subunit G	1700.10	1445.30	1671.27	763.68	1248.55	1199.72	-0.58
KLMA_40609	RPN9	26S proteasome regulatory subunit RPN9	1038.17	976.58	934.30	817.51	1153.35	900.66	-0.04
KLMA_40610	SNX41	sorting nexin-41	366.83	510.10	322.80	730.88	544.24	658.64	0.69
KLMA_40611	KIF1C	kinesin family protein	143.44	170.03	159.57	284.28	272.96	239.43	0.75
KLMA_40612	NCS6	cytoplasmic tRNA 2-thiolation protein 1	748.94	726.00	782.04	643.41	516.44	527.26	-0.42
KLMA_40613	ZRG8	zinc-regulated protein 8	331.56	385.93	281.39	428.94	385.85	416.62	0.30
KLMA_40614	FIR1	factor interacting with REF2	472.64	409.43	495.78	452.49	575.41	587.76	0.23
KLMA_40632		GTP-binding protein	907.66	816.61	827.11	715.74	826.47	853.12	-0.09
KLMA_40615		histone H2A.Z-specific chaperone CHZ1	322.15	239.39	305.75	341.47	323.51	294.74	0.15
KLMA_40616	SMB1	small nuclear ribonucleoprotein-associated protein B	455.01	432.92	473.85	294.37	353.00	302.52	-0.52
KLMA_40617		putaive regulatory protein	211.63	236.04	238.75	152.23	98.57	162.50	-0.73
KLMA_40618	GAL83	SNF1 protein kinase subunit beta-3	612.55	695.80	604.19	438.19	462.52	523.80	-0.43
KLMA_40619	PRP43	pre-mRNA-splicing factor ATP-dependent RNA helicase PRP43	2042.24	1814.45	2208.46	860.40	990.75	882.50	-1.15
KLMA_40620	COQ8	protein ABC1	572.58	738.31	657.79	465.95	404.39	470.21	-0.55
KLMA_40621		hypothetical protein	8.23	8.95	15.84	126.16	21.90	48.40	2.58
KLMA_40622		hypothetical protein	1608.40	956.45	1367.96	707.33	686.62	615.42	-0.97
KLMA_40623		delta(12) fatty acid desaturase	1108.71	1024.68	1311.92	1272.52	1449.06	1443.47	0.27
KLMA_40624	adh	alcohol dehydrogenase	1640.14	1792.08	1555.55	5520.71	7502.26	7425.66	2.04
KLMA_40625	THI4	thiazole biosynthetic enzyme	205.75	183.46	187.59	465.11	369.01	402.79	1.10
KLMA_40626		flocculation protein FLO9	570.23	956.45	532.32	248.11	256.11	302.52	-1.35

Locus_tag	UniProt_gene	Product	Unique exon reads						log2 Fold Change
			KmWT.1	KmWT.2	KmWT.3	Kmmig1.1	Kmmig1.2	Kmmig1.3	
KLMA_40627	GEX1	siderophore iron transporter ARN1	471.47	393.76	420.25	3233.87	1003.39	866.08	1.99
KLMA_40628		NADPH-dependent methylglyoxal reductase GRE2	41.15	36.92	76.74	137.09	92.67	152.13	1.31
KLMA_40629		transposon Ty1-H Gag-Pol polyprotein	36.45	23.49	43.85	84.95	76.67	66.56	1.14
KLMA_40630	TY2B-GR2	transposon Ty2-F/Ty2-GR2 Gag-Pol polyprotein	2.35	2.24	12.18	22.71	16.85	32.85	2.13
KLMA_50005	GTT1	glutathione S-transferase 1	831.24	2666.86	1152.35	1735.10	1569.54	1519.53	0.05
KLMA_50006	PEX22	peroxisome assembly protein 22	335.08	256.17	367.87	276.71	310.87	260.17	-0.18
KLMA_50007	RAD17	DNA damage checkpoint control protein RAD17	329.20	285.26	311.84	439.87	439.77	423.53	0.49
KLMA_50008	RPS12	40S ribosomal protein S12	12085.31	8208.66	11037.44	3984.94	8068.40	6135.18	-0.78
KLMA_50009	GPB1	guanine nucleotide-binding protein subunit beta 1	926.47	1064.96	979.37	1667.82	1068.26	1248.99	0.42
KLMA_50010	NDD1	nuclear division defective protein 1	1078.14	1148.85	1086.57	1190.10	1163.46	1160.83	0.08
KLMA_50011	NUD1	protein NUD1	383.29	477.66	420.25	896.57	517.28	572.20	0.63
KLMA_50012	ALD4	potassium-activated aldehyde dehydrogenase	81.13	118.58	110.85	18758.97	20590.14	19069.37	7.55
KLMA_50013	cal1	calnexin homolog	1300.36	1567.23	1337.50	1319.62	1781.84	1694.13	0.19
KLMA_50014	ECM1	protein ECM1	686.63	482.14	600.54	699.76	624.28	607.64	0.13
KLMA_50016	BDH1	(2R,3R)-2,3-butanediol dehydrogenase	7681.03	8269.06	8162.66	5969.83	4014.40	4412.52	-0.74
KLMA_50017	BDH2	(2R,3R)-2,3-butanediol dehydrogenase	1272.14	856.89	952.57	4977.39	3624.34	2921.52	1.90
KLMA_50018		hypothetical protein	493.81	418.38	559.12	817.51	469.26	533.31	0.31
KLMA_50019	GDH3	NADP-specific glutamate dehydrogenase 2	1748.31	1615.33	2245.01	1851.17	2277.22	2549.84	0.25
KLMA_50020	FRE3	ferric reductase transmembrane component 3	853.58	979.94	1006.17	1630.81	1068.26	1071.80	0.41

Locus_tag	UniProt_gene	Product	Unique exon reads						$\log_2$ Fold Change
			KmWT.1	KmWT.2	KmWT.3	Kmmig1.1	Kmmig1.2	Kmmig1.3	
KLMA_50021		zinc-type alcohol dehydrogenase-like protein C2E1P3.01	308.04	185.70	192.46	588.74	362.27	447.74	1.03
KLMA_50022	GDE1	glycerophosphodiester phosphodiesterase GDE1	404.45	529.12	436.09	1335.60	908.19	1066.61	1.27
KLMA_50023		hypothetical protein	48.20	51.46	38.98	1471.01	1141.56	1279.24	4.81
KLMA_50024	CAR1	arginase	77.60	109.63	85.27	452.49	324.35	476.26	2.20
KLMA_50025	PEX25	peroxisomal membrane protein PEX25	237.50	278.54	275.30	670.32	546.77	611.10	1.21
KLMA_50026	TOA1	transcription initiation factor IIA large subunit	739.53	694.68	688.24	829.28	722.00	662.96	0.06
KLMA_50027	SLK19	kinetochore protein SLK19	322.15	302.04	305.75	375.95	312.56	336.23	0.14
KLMA_50028		putative 2-hydroxyacid dehydrogenase YPL113C	368.00	345.66	359.35	496.22	700.94	661.23	0.79
KLMA_50030	BEM3	GTPase-activating protein BEM3	853.58	826.68	965.97	610.61	536.66	586.90	-0.61
KLMA_50031		lipoyl synthase	1045.22	1061.60	1127.98	1129.54	1094.38	1147.00	0.06
KLMA_50032	HGT1	high-affinity glucose transporter	148.14	171.15	133.99	10912.74	5198.08	6072.95	5.61
KLMA_50033	HOS3	histone deacetylase HOS3	748.94	746.14	621.24	629.95	579.62	573.93	-0.25
KLMA_50034	MCA1	metacaspase-1	774.81	742.78	756.46	644.25	600.69	751.12	-0.19
KLMA_50035	BFR1	nuclear segregation protein BFR1	2432.58	2188.08	2093.96	951.24	1911.58	1637.09	-0.58
KLMA_50036	IDI1	isopentenyl-diphosphate Delta-isomerase	1126.35	975.46	1025.66	576.97	710.21	670.74	-0.68
KLMA_50037	MRM1	rRNA methyltransferase	297.46	344.54	354.47	327.17	231.68	282.64	-0.24
KLMA_50038	HIS3	imidazoleglycerol-phosphate dehydratase	546.71	349.02	449.49	637.52	611.64	628.39	0.48
KLMA_50039		uncharacterized protein in HIS3 3'region	741.88	947.50	831.98	557.62	795.30	726.92	-0.28
KLMA_50040	AGP1	high-affinity glutamine permease	243.38	309.87	232.66	500.43	273.81	272.27	0.41

Locus_tag	UniProt_gene	Product	Unique exon reads						log_2 Fold Change
			KmWT.1	KmWT.2	KmWT.3	Kmmig1.1	Kmmig1.2	Kmmig1.3	
KLMA_50041	DED1	ATP-dependent RNA helicase DED1	7102.58	6246.54	6817.85	5490.43	4035.47	4180.88	-0.56
KLMA_50042	TFB2	RNA polymerase II transcription factor B subunit 2	465.59	413.90	428.78	200.17	283.91	265.36	-0.80
KLMA_50043	RET1	DNA-directed RNA polymerase III subunit RPC2	1169.85	962.04	1037.84	555.10	576.25	560.10	-0.91
KLMA_50044	MEI5	meiosis protein 5	35.27	30.20	36.54	13.46	11.79	13.83	-1.38
KLMA_50045	NOC2	nucleolar complex protein 2	1798.86	1560.52	1869.82	636.68	805.41	706.18	-1.28
KLMA_50046	GEP3	mitochondrial protein FMP38	489.10	528.00	600.54	243.07	284.76	268.81	-1.02
KLMA_50047	VPS30	vacuolar protein sorting-associated protein 30	158.72	203.59	194.90	411.28	224.10	247.21	0.66
KLMA_50048	RNR1	ribonucleoside-diphosphate reductase large chain 1	237.50	287.49	316.71	338.11	313.40	298.20	0.17
KLMA_50049	THI72	thiamine transporter THI72	126.98	145.42	135.21	354.93	199.67	261.90	1.00
KLMA_50050	SHM1	serine hydroxymethyltransferase	2939.32	3174.73	2967.36	1040.39	1277.20	1226.52	-1.36
KLMA_50051	VPS21	vacuolar protein sorting-associated protein 21	251.61	173.39	153.48	423.05	375.74	387.23	1.04
KLMA_50052	RIF1	telomere length regulator protein RIF1	685.45	588.41	677.28	723.31	621.75	572.20	-0.02
KLMA_50053	CHK1	serine/threonine-protein kinase CHK1	385.64	317.70	298.44	393.62	292.34	350.06	0.05
KLMA_50054	UBX7	UBX domain-containing protein 7	502.04	616.38	583.48	748.54	589.73	605.05	0.19
KLMA_50055		uncharacterized protein YJL049W	331.56	281.90	294.79	834.33	469.26	503.92	0.99
KLMA_50056	HSM3	DNA mismatch repair protein HSM3	356.25	352.37	291.13	665.28	544.24	535.90	0.80
KLMA_50057	MTR4	ATP-dependent RNA helicase DOB1	1782.40	1690.28	1896.62	804.05	803.72	861.76	-1.12
KLMA_50058		uncharacterized protein IRC8	190.47	178.98	253.37	264.93	383.33	373.40	0.71
KLMA_50059	RTK1	probable	236.32	234.92	283.82	417.17	380.80	407.97	0.68

Locus_tag	UniProt_gene	Product	Unique exon reads						log$_2$ Fold Change
			KmWT.1	KmWT.2	KmWT.3	Kmmig1.1	Kmmig1.2	Kmmig1.3	
KLMA_50060	PEP8	serine/threonine-protein kinase vacuolar protein sorting-associated protein 26	224.56	315.46	267.99	396.98	396.81	374.27	0.53
KLMA_50061	TIM54	mitochondrial import inner membrane translocase subunit TIM54	407.98	519.05	470.20	371.75	326.04	313.76	-0.47
KLMA_50062	ABF2	putative high mobility group protein B3-like protein	730.13	794.24	813.71	1047.12	1150.82	967.21	0.44
KLMA_50063	IKS1	probable serine/threonine-protein kinase IKS1	390.34	438.51	397.11	855.36	437.25	549.73	0.59
KLMA_50064	BIT61	target of rapamycin complex 2 subunit BIT61,HbrB super family conserved domain	197.52	200.24	204.65	329.69	342.89	315.49	0.71
KLMA_50065	BTN1	protein BTN1	197.52	196.88	170.54	412.96	467.57	442.55	1.23
KLMA_50066		hypothetical protein	105.82	100.68	81.61	90.83	82.56	85.57	-0.15
KLMA_50067	BNA3	probable kynurenine--oxoglutarate transaminase BNA3	1200.42	871.43	1226.65	1291.03	1483.60	1801.31	0.47
KLMA_50068	FMP21	protein FMP21	79.95	101.80	58.47	148.03	140.69	107.18	0.72
KLMA_50069	NUP82	nucleoporin NUP82	1008.78	979.94	976.94	684.62	747.28	726.06	-0.46
KLMA_50070	MRPL37	54S ribosomal protein L37	64.67	76.07	68.22	60.56	101.94	73.47	0.17
KLMA_50071	LAS21	GPI ethanolamine phosphate transferase 2	474.99	469.83	498.21	373.43	538.34	449.46	-0.08
KLMA_50072	REI1	pre-60S factor REI1	870.04	683.50	818.58	563.51	468.42	538.49	-0.59
KLMA_50073	TSC10	3-ketodihydrosphingosine reductase TSC10	590.22	469.83	627.34	767.05	754.86	867.81	0.50
KLMA_50074		mitochondrial membrane protein YJL062W-A	203.40	218.14	214.39	144.66	169.34	120.15	-0.55

Locus_tag	UniProt_gene	Product	Unique exon reads						log$_2$ Fold Change
			KmWT.1	KmWT.2	KmWT.3	Kmmig1.1	Kmmig1.2	Kmmig1.3	
KLMA_50075	MRPL8	54S ribosomal protein L8	357.42	290.85	324.02	243.07	449.04	343.15	0.09
KLMA_50076	cysB	cysteine synthase	844.17	797.60	830.76	608.09	657.97	625.79	-0.39
KLMA_50077	PPS1	dual specificity protein phosphatase PPS1	924.12	869.19	1020.79	1230.47	930.94	1017.34	0.18
KLMA_50078	DPB3	DNA polymerase epsilon subunit C	43.50	52.58	24.36	189.24	68.24	106.32	1.59
KLMA_50079	MPM1	mitochondrial peculiar membrane protein 1	157.55	285.26	215.61	391.09	291.50	322.40	0.61
KLMA_50080	CPS1	putative carboxypeptidase YOL153C	375.06	517.94	315.49	737.61	552.67	903.25	0.86
KLMA_50081	PAF1	RNA polymerase II-associated protein 1	683.10	638.75	572.52	504.64	598.16	488.36	-0.25
KLMA_50082		S-formylglutathione hydrolase	718.37	834.51	639.52	1326.35	1357.23	1257.63	0.85
KLMA_50083	SAF1	SCF-associated factor 1	413.86	772.99	436.09	1161.50	932.62	966.35	0.91
KLMA_50084	DUG2	probable di- and tripeptidase DUG2	798.32	617.50	885.58	623.22	585.52	608.50	-0.34
KLMA_50085	UTP18	U3 small nucleolar RNA-associated protein 18	823.01	703.63	907.50	370.91	381.64	411.43	-1.06
KLMA_50086	MRPL27	54S ribosomal protein L27	364.48	401.60	320.37	312.03	384.17	357.84	-0.04
KLMA_50087	SSH1	sec sixty-one protein homolog	4070.37	3419.71	4114.83	3347.41	4136.56	3727.09	-0.05
KLMA_50088	AIM5	uncharacterized protein YBR262C	47.03	49.22	41.42	50.46	64.03	55.32	0.30
KLMA_50089	TAE1	methyltransferase-like protein YBR261C	217.51	201.36	209.52	192.60	218.20	235.97	0.04
KLMA_50090	RGD1	RHO GTPase-activating protein RGD1	690.15	930.72	689.46	892.36	761.60	815.09	0.10
KLMA_50091		COG5647 (Cullin	176.36	173.39	163.23	787.23	445.67	493.55	1.75
KLMA_50092		hypothetical protein	219.86	203.59	181.50	635.00	392.59	386.37	1.22
KLMA_50093		COMPASS component SHG1	74.07	46.98	53.60	137.09	144.91	80.38	1.05
KLMA_50094	POP4	RNases MRP/P 32.9 kDa subunit	162.25	180.10	197.34	614.81	409.44	340.56	1.34

Locus_tag	UniProt_gene	Product	Unique exon reads						$\log_2$ Fold Change
			KmWT.1	KmWT.2	KmWT.3	Kmmig1.1	Kmmig1.2	Kmmig1.3	
KLMA_50095	AIM22	putative lipoate-protein ligase A	225.74	270.71	227.79	453.33	347.10	348.33	0.66
KLMA_50096	PTR3	SPS-sensor component PTR3	457.36	420.61	501.87	848.63	563.62	591.22	0.54
KLMA_50097		hypothetical protein	48.20	22.37	46.29	126.16	42.12	32.85	0.79
KLMA_50098		fungal_trans super family conserved domain	1065.21	1119.77	1269.29	1153.93	955.37	904.98	-0.20
KLMA_50099	RPA43	DNA-directed RNA polymerase I subunit RPA43	766.58	771.87	830.76	368.38	502.96	417.48	-0.88
KLMA_50100	MTW1	kinetochore-associated protein MTW1	99.94	97.32	105.98	223.72	207.25	167.68	0.98
KLMA_50101		SWIRM domain-containing protein YOR338W	4673.52	5412.03	4293.90	2641.77	1492.03	1718.33	-1.30
KLMA_50102	TEA1	TY1 enhancer activator	365.65	368.04	373.96	526.50	236.74	345.74	0.00
KLMA_50103	KRE5	killer toxin-resistance protein 5	478.52	799.84	514.05	709.85	641.97	645.67	0.16
KLMA_50104	ALA1	alanyl-tRNA synthetase	3807.01	3529.34	3586.16	1817.53	2281.43	2189.41	-0.80
KLMA_50105	POP5	ribonucleases P/MRP protein subunit POP5	104.64	97.32	92.58	75.70	95.20	61.37	-0.34
KLMA_50106	PRP45	pre-mRNA-processing protein 45	194.00	171.15	144.96	412.12	307.50	321.54	1.03
KLMA_50107	GIP4	GLC7-interacting protein 4	704.26	798.72	679.71	574.44	605.74	581.71	-0.31
KLMA_50108	MRS2	mitochondrial inner membrane magnesium transporter mrs2	259.84	327.76	253.37	277.55	269.59	316.35	0.04
KLMA_50109	VMA4	v-type proton ATPase subunit E	1749.48	1815.57	1626.20	1402.04	1852.61	1667.34	-0.08
KLMA_50110	MIP1	DNA polymerase gamma	1128.70	1205.91	1294.87	1359.99	1075.84	1107.24	-0.03
KLMA_50111	SCD5	protein SCD5	152.84	260.65	191.25	560.99	282.23	283.51	0.90
KLMA_50112	SNC2	synaptobrevin homolog 2	425.61	425.09	421.47	291.01	414.50	331.05	-0.30
KLMA_50113	MYO2	myosin-2	2009.32	2411.81	2098.83	1526.52	1614.19	1554.11	-0.47

Locus_tag	UniProt_gene	Product	Unique exon reads						log2 Fold Change
			KmWT.1	KmWT.2	KmWT.3	Kmmig1.1	Kmmig1.2	Kmmig1.3	
KLMA_50114		ras_like_GTPase super family	503.21	451.93	521.36	544.16	505.49	476.26	0.05
KLMA_50115	SAW1	single-strand annealing weakened protein 1	79.95	102.92	79.18	212.79	172.71	218.68	1.20
KLMA_50117	PRO2	gamma-glutamyl phosphate reductase	1096.95	813.26	975.72	933.58	1129.76	1092.54	0.13
KLMA_50118	DRS2	probable phospholipid-transporting ATPase DRS2	1410.87	1393.84	1388.66	960.49	998.34	1036.36	-0.49
KLMA_50119	MAK16	protein MAK16	632.54	605.19	641.95	258.21	356.37	374.27	-0.93
KLMA_50120	LTE1	guanine nucleotide exchange factor LTE1	1375.60	1634.35	1464.19	764.52	746.43	706.18	-1.01
KLMA_50121		uncharacterized transporter C417.10	202.23	213.66	177.85	341.47	213.99	238.56	0.42
KLMA_50122	LDB19	protein LDB19	986.44	1115.29	1115.80	646.77	711.89	851.39	-0.54
KLMA_50123		uncharacterized CDP-alcohol phosphatidyltransferase class-I family protein with HAD_like super family	156.37	253.93	182.72	5085.04	2867.79	4091.85	4.34
KLMA_50124	AHA1	hsp90 co-chaperone AHA1	543.19	661.12	467.76	439.03	592.26	415.75	-0.21
KLMA_50125	UPC2	sterol regulatory element-binding protein ECM22	2516.06	1856.96	2481.32	325.49	455.78	482.31	-2.44
KLMA_50126	TCP1	T-complex protein 1 subunit alpha	1728.32	1742.86	1721.21	957.97	1375.77	1372.59	-0.49
KLMA_50127	CDC42	cell division control protein 42	989.96	813.26	989.12	487.81	609.11	549.73	-0.76
KLMA_50128	GCD6	translation initiation factor eIF-2B subunit epsilon	1203.95	1137.67	1107.28	672.01	800.35	717.41	-0.66
KLMA_50129	MSS4	probable phosphatidylinositol-4-phosphate 5-kinase MSS4	456.18	415.02	443.40	767.89	545.93	671.60	0.59
KLMA_50130		pH-response regulator palI/RIM9 homolog 1	479.70	291.97	375.18	580.33	293.18	376.86	0.13

Locus_tag	UniProt_gene	Product	Unique exon reads						log2 Fold Change
			KmWT.1	KmWT.2	KmWT.3	Kmmig1.1	Kmmig1.2	Kmmig1.3	
KLMA_50131		uncharacterized protein YLR290C	223.39	252.82	214.39	222.04	272.12	265.36	0.14
KLMA_50132	GCD7	translation initiation factor eIF-2B subunit beta	344.49	260.65	348.38	221.20	338.68	262.76	-0.21
KLMA_50133	SEC72	translocation protein SEC72	261.01	211.42	260.68	374.27	414.50	381.18	0.68
KLMA_50134	GSP1	GTP-binding nuclear protein GSP1/Ran	4218.51	2902.90	3759.14	1965.55	2365.68	2293.13	-0.72
KLMA_50135		ATP synthase subunit H	681.92	800.95	706.51	371.75	618.38	480.58	-0.57
KLMA_50136	TUF1	elongation factor Tu	2877.01	2905.14	2558.07	1124.50	1691.69	1423.59	-0.98
KLMA_50138		zinc finger	451.48	436.27	344.73	598.83	351.31	322.40	0.05
KLMA_50139	fnx1	multidrug resistance protein fnx1	1100.48	658.89	933.08	2179.18	1240.13	1312.09	0.81
KLMA_50140	ENP2	ribosome biogenesis protein ENP2	1181.61	1118.65	1246.14	528.18	588.05	566.15	-1.08
KLMA_50141	GTR2	GTP-binding protein GTR2	597.27	609.66	568.86	592.95	657.13	656.91	0.10
KLMA_50142	TIF4632	eukaryotic initiation factor 4F subunit p150	3595.38	2928.63	3421.72	1307.85	1591.44	1452.11	-1.19
KLMA_50143	RPT6	26S protease regulatory subunit 8 homolog	962.92	1015.73	970.85	830.97	871.12	957.70	-0.15
KLMA_50144	ALG13	UDP-N-acetylglucosamine transferase subunit ALG13	77.60	109.63	109.63	124.48	103.62	153.85	0.36
KLMA_50145	GET1	Golgi to ER traffic protein 1	921.77	921.77	840.51	752.75	673.14	795.21	-0.27
KLMA_50146	CKB1	casein kinase II subunit beta	404.45	497.80	450.71	372.59	383.33	422.67	-0.20
KLMA_50147	JAC1	j-type co-chaperone JAC1	649.00	606.31	612.72	521.46	691.67	562.69	-0.07
KLMA_50148	ATE1	arginyl-tRNA--protein transferase 1	250.43	243.87	257.02	479.40	406.92	421.80	0.80
KLMA_50149	KAP122	pleiotropic drug resistance regulatory protein 6	206.93	225.97	175.41	488.66	435.56	462.43	1.19
KLMA_50151	ARO8	aromatic amino acid aminotransferase 1	150.49	192.41	182.72	790.60	434.72	484.90	1.70
KLMA_50152	HIR1	protein HIR1	385.64	327.76	378.84	238.02	278.02	305.98	-0.41

Locus_tag	UniProt_gene	Product	Unique exon reads						log$_2$ Fold Change
			KmWT.1	KmWT.2	KmWT.3	Kmmig1.1	Kmmig1.2	Kmmig1.3	
KLMA_50154	LEU1	3-isopropylmalate dehydratase	6330.12	3937.65	5764.17	1661.93	2208.13	2539.47	-1.32
KLMA_50155	SLA1	actin cytoskeleton-regulatory complex protein SLA1	2276.21	2892.83	2327.84	1828.46	2040.48	2032.10	-0.35
KLMA_50156		uncharacterized endoplasmic reticulum membrane protein YGL010W	271.59	325.53	281.39	1222.06	499.59	505.65	1.34
KLMA_50157	SCL1	proteasome component C7-alpha	698.38	832.28	679.71	982.36	946.10	1005.24	0.41
KLMA_50158		chromatin structure-remodeling complex protein RSC14	178.71	233.80	226.57	191.76	205.56	199.67	-0.10
KLMA_50159		GAL4-like Zn2Cys6 binuclear cluster DNA-binding conserved domain	427.97	380.34	325.24	354.09	228.31	274.86	-0.40
KLMA_50160		BAR super family and SH3	16.46	24.61	3.65	117.75	58.97	76.93	2.49
KLMA_50161	DAK1	dihydroxyacetone kinase 1	848.88	1226.04	788.13	5302.03	5598.26	7406.65	2.68
KLMA_50162	POB3	FACT complex subunit POB3	1024.06	1097.40	1003.74	744.34	1065.73	1036.36	-0.13
KLMA_50163	ITT1	translation termination inhibitor protein ITT1	269.24	286.37	254.59	784.71	512.23	449.46	1.11
KLMA_50164		putative transcriptional activator YLR445W	8.23	6.71	15.84	37.01	34.54	28.52	1.71
KLMA_50165	ERV41	ER-derived vesicles protein ERV41	497.33	538.07	516.49	418.85	569.51	516.02	-0.04
KLMA_50166	SMA2	spore membrane assembly protein 2	269.24	297.56	383.71	306.15	280.54	341.42	-0.03
KLMA_50167	ECM7	protein ECM7	306.87	271.83	293.57	431.46	331.09	322.40	0.32
KLMA_50168	ORC1	origin recognition complex subunit 1	559.65	647.70	604.19	636.68	668.08	644.81	0.11
KLMA_50169		protein TEM1	69.37	117.46	109.63	135.41	114.58	121.01	0.32
KLMA_50170	RPS1	40S ribosomal protein S1	27917.67	25373.23	27658.78	8667.95	18018.06	14543.61	-0.97
KLMA_50171		THO complex subunit MFT1	181.06	224.85	197.34	132.05	221.57	172.01	-0.20
KLMA_50172	SEC39	protein transport protein SEC39	463.24	544.78	510.39	342.31	451.57	436.50	-0.30

Locus_tag	UniProt_gene	Product	Unique exon reads						log$_2$ Fold Change
			KmWT.1	KmWT.2	KmWT.3	Kmmig1.1	Kmmig1.2	Kmmig1.3	
KLMA_50173	PIF1	DNA repair and recombination protein PIF1or ATP-dependent helicase PIF1	385.64	457.53	470.20	406.23	335.31	389.82	-0.22
KLMA_50174	OGG1	N-glycosylase/DNA lyase	155.20	137.59	127.90	213.63	141.54	167.68	0.31
KLMA_50175	MRPL4	54S ribosomal protein L4	739.53	663.36	762.55	566.87	705.15	709.63	-0.13
KLMA_50176	LSM3	U6 snRNA-associated Sm-like protein LSm3	92.88	85.02	125.47	110.18	171.02	142.62	0.48
KLMA_50177	NTE1	lysophospholipase NTE1	356.25	472.07	370.31	608.93	605.74	607.64	0.60
KLMA_50178	CAR2	ornithine aminotransferase	61.14	79.42	42.63	385.20	344.57	592.08	2.85
KLMA_50179	DIF1	uncharacterized protein YLR437C	1027.59	1060.48	1030.53	1180.85	1132.29	1042.41	0.11
KLMA_50180		hypothetical protein	1191.01	1086.21	1106.06	1045.44	1011.82	1035.50	-0.13
KLMA_50181	TSR2	pre-rRNA-processing protein TSR2	345.66	297.56	328.89	201.85	264.54	246.34	-0.45
KLMA_50182	CMP2	serine/threonine-protein phosphatase 2B catalytic subunit A1	784.21	948.62	794.22	855.36	770.87	808.17	-0.05
KLMA_50183	IMD4	inosine-5'-monophosphate dehydrogenase	27122.88	19103.19	25371.13	5702.38	6733.08	6585.51	-1.91
KLMA_50185	ATG23	autophagy-related protein 23	213.98	368.04	214.39	357.45	357.21	384.64	0.46
KLMA_50186	SEN1	helicase SEN1	597.27	684.61	707.73	544.16	497.06	536.76	-0.33
KLMA_50187	RPB2	DNA-directed RNA polymerase II subunit RPB2	4815.78	4831.45	4721.46	2442.43	2901.49	2749.51	-0.83
KLMA_50188	MRP20	54S ribosomal protein L41	885.32	926.24	951.36	514.73	699.26	609.37	-0.60
KLMA_50189	ORM1	protein ORM1	477.35	519.05	445.83	408.75	449.04	455.51	-0.14
KLMA_50190	ACB1	acyl-CoA-binding protein,ACBP	1001.72	1314.41	1125.55	819.19	1133.97	956.84	-0.24
KLMA_50191	KAP95	importin subunit beta-1	2766.49	2768.66	2572.68	1878.93	3021.12	2961.28	-0.04
KLMA_50192	CAX4	dolichyldiphosphatas	268.07	346.78	337.42	145.50	294.87	303.39	-0.36

Locus_tag	UniProt_gene	Product	Unique exon reads						log2 Fold Change
			KmWT.1	KmWT.2	KmWT.3	Kmmig1.1	Kmmig1.2	Kmmig1.3	
		e							
KLMA_50193		putative 6-phosphofructo-2-kinase/fructose-2	510.27	834.51	433.65	665.28	749.80	652.59	0.22
KLMA_50194	RPL26B	60S ribosomal protein L26-B	10414.60	8774.69	10909.54	3339.84	6546.89	5293.30	-0.99
KLMA_50195	TIM21	mitochondrial import inner membrane translocase subunit TIM21	336.26	421.73	297.22	408.75	347.94	365.62	0.09
KLMA_50196	GAS2	1,3-beta-glucanosyltransferase GAS2	150.49	130.88	141.30	165.69	149.12	144.35	0.12
KLMA_50197	FKS1	1,3-beta-glucan synthase component FKS1	23971.93	22620.23	20777.58	20455.39	19203.43	19419.43	-0.19
KLMA_50198		nested antisense gene NAG1	185.77	168.92	120.59	651.82	313.40	320.68	1.44
KLMA_50199		hypothetical protein	29.39	22.37	21.93	154.75	44.65	70.88	1.88
KLMA_50200	RPP0	60S acidic ribosomal protein P0	31660.02	21747.68	28286.11	8860.55	18146.96	16805.63	-0.90
KLMA_50201		ribonucleases P/MRP protein subunit POP6	39.97	36.92	29.24	27.75	46.34	35.44	0.04
KLMA_50202		DUF3445 super family conserved domain	148.14	144.31	159.57	421.37	372.37	447.74	1.46
KLMA_50203	VRP1	verprolin	158.72	187.93	144.96	113.54	126.37	181.51	-0.22
KLMA_50206	ERV1	mitochondrial FAD-linked sulfhydryl oxidase ERV1	526.73	427.32	477.51	79.90	120.47	78.66	-2.36
KLMA_50207	MSP1	protein MSP1	589.04	684.61	581.05	423.89	347.10	446.01	-0.61
KLMA_50208	SGD1	suppressor of glycerol defect protein1with MIF4G super family conserved domain	652.53	519.05	678.50	343.15	363.95	403.65	-0.74
KLMA_50209	NUP2	nucleoporin NUP2	1274.49	1370.35	1246.14	783.03	1048.88	1011.29	-0.45
KLMA_50210	RPS25B	40S ribosomal protein S25	11381.05	9749.04	11395.57	4777.21	7862.00	6221.62	-0.79
KLMA_50211		uncharacterized membrane protein	1045.22	1077.26	1020.79	952.08	1020.24	1011.29	-0.08

Locus_tag	UniProt_gene	Product	Unique exon reads						log$_2$ Fold Change
			KmWT.1	KmWT.2	KmWT.3	Kmmig1.1	Kmmig1.2	Kmmig1.3	
		YGR026W							
KLMA_50212	THG1	tRNA(His) guanylyltransferase	221.04	201.36	272.86	348.20	262.85	267.09	0.34
KLMA_50214	RPA135	DNA-directed RNA polymerase I subunit RPA2	5354.27	4423.14	5131.97	1663.61	1853.45	2054.57	-1.42
KLMA_50215	CWP1	cell wall protein CWP1	22225.97	29482.03	17421.64	9615.82	8439.94	10938.39	-1.25
KLMA_50216	YJU2	protein CWC16	81.13	158.85	91.36	300.26	121.32	165.09	0.82
KLMA_50217	YJU3	serine hydrolase YJU3	1524.92	2174.66	1811.35	1206.92	560.25	552.32	-1.25
KLMA_50218		protein MBR1	3212.09	3806.77	3187.84	1292.71	1080.90	1119.34	-1.55
KLMA_50219	NAM7	ATP-dependent helicase NAM7	1115.77	1106.35	1078.04	714.90	792.77	815.09	-0.51
KLMA_50220	BUD2	inhibitory regulator protein BUD2/CLA2	671.34	611.90	621.24	641.73	706.84	694.94	0.10
KLMA_50221	SEC14	SEC14 cytosolic factor	2613.64	1995.67	2282.77	1581.19	2630.21	2433.16	-0.05
KLMA_50222	PTK2	serine/threonine-protein kinase PTK2	2746.50	3821.31	2946.65	1482.79	1180.31	1300.85	-1.26
KLMA_50223	PEX1	peroxisomal ATPase PEX1	50.56	43.63	34.11	48.78	61.50	91.62	0.65
KLMA_50225	APS2	AP-2 complex subunit sigma	70.54	46.98	42.63	243.07	228.31	219.55	2.11
KLMA_50226	CDC8	thymidylate kinase	162.25	147.66	137.65	254.00	227.47	207.44	0.62
KLMA_50227	CDC14	tyrosine-protein phosphatase CDC14	400.92	442.99	427.56	275.87	420.40	405.38	-0.21
KLMA_50228	ECO1	N-acetyltransferase ECO1	39.97	80.54	38.98	143.82	112.89	120.15	1.24
KLMA_50229	DAS2	putative uridine kinase YDR020C	144.61	132.00	118.16	135.41	110.36	100.27	-0.19
KLMA_50230	TCM62	mitochondrial chaperone TCM62	245.73	233.80	286.26	185.87	215.67	247.21	-0.24
KLMA_50231	GCV1	aminomethyltransferase	4113.87	2916.32	4274.41	1979.01	2230.88	1920.59	-0.88
KLMA_50232	SAM3	S-adenosylmethionine permease SAM3	3843.46	2949.88	3614.18	3805.79	2340.40	2408.95	-0.28
KLMA_50233	QDR3	uncharacterized transporter YBR043C	188.12	174.51	192.46	512.20	371.53	516.02	1.33
KLMA_50234	CST26	uncharacterized acyltransferase	940.58	1077.26	1135.29	1418.02	1118.81	1130.57	0.22

Locus_tag	UniProt_gene	Product	Unique exon reads						log_2 Fold Change
			KmWT.1	KmWT.2	KmWT.3	Kmmig1.1	Kmmig1.2	Kmmig1.3	
		CST26							
KLMA_50235	FAT1	very long-chain fatty acid transport protein	1586.06	1551.57	1700.50	1725.01	1185.37	1370.00	-0.18
KLMA_50236	FIG1	factor-induced gene 1 protein	0.00	6.71	0.00	285.12	140.69	210.90	6.55
KLMA_50237	ATP3	ATP synthase subunit gamma	4799.32	4320.23	4528.99	2995.85	4471.87	3958.74	-0.26
KLMA_50238	KCS1	inositol hexakisphosphate kinase 1	658.41	738.31	816.14	443.24	468.42	526.39	-0.62
KLMA_50239	DAD1	DASH complex subunit DAD1	56.43	55.93	68.22	66.44	86.78	38.03	0.08
KLMA_50240		UPF0695 membrane protein YPL279C	337.43	248.34	350.82	142.14	148.28	201.39	-0.93
KLMA_50241	PDX3	pyridoxamine 5'-phosphate oxidase	1539.03	939.67	1411.81	387.73	631.86	507.38	-1.35
KLMA_50242		protein RAD61	136.38	149.90	142.52	169.05	160.07	161.63	0.19
KLMA_50243	PSF1	DNA replication complex GINS protein PSF1	47.03	50.34	49.94	65.60	42.12	78.66	0.34
KLMA_50244	HMT1	HNRNP arginine N-methyltransferase	1454.38	1096.28	1487.33	737.61	923.36	956.84	-0.63
KLMA_50245	RPL4B	60S ribosomal protein L4-B	64987.22	45138.67	56373.67	24802.82	38493.63	30800.37	-0.82
KLMA_50246	RKM3	ribosomal N-lysine methyltransferase 3	245.73	299.80	252.15	229.61	215.67	216.09	-0.27
KLMA_50247	MRP49	54S ribosomal protein MRP49	129.33	127.53	138.87	65.60	160.07	112.37	-0.23
KLMA_50248	TPK1	cAMP-dependent protein kinase type 3	981.73	901.63	877.05	2148.90	1444.01	1374.32	0.85
KLMA_50249		uncharacterized membrane protein YJL163C	765.40	726.00	663.88	3119.49	2509.74	2644.06	1.94
KLMA_50250	MCD4	GPI ethanolamine phosphate transferase 1	910.01	789.77	896.54	1359.15	1078.37	1028.58	0.42
KLMA_50251		J protein JJJ2	112.87	144.31	177.85	984.04	354.68	326.73	1.94
KLMA_50252		mitochondrial membrane protein FMP33	27.04	29.08	21.93	111.02	114.58	121.87	2.15
KLMA_50253	PIR1	cell wall mannoprotein	8594.58	8251.17	6767.91	19564.71	16577.42	16873.91	1.17

Locus_tag	UniProt_gene	Product	Unique exon reads						log_2 Fold Change
			KmWT.1	KmWT.2	KmWT.3	Kmmig1.1	Kmmig1.2	Kmmig1.3	
		HSP150							
KLMA_50254	PIR1	cell wall mannoprotein HSP150	28179.86	27452.80	21867.80	14849.73	14266.51	21102.33	-0.63
KLMA_50255	PIR3	cell wall mannoprotein HSP150	4947.47	5372.88	4585.03	1530.73	2571.24	3338.13	-1.00
KLMA_50256	FAR1	cyclin-dependent kinase inhibitor FAR1	106.99	117.46	119.38	262.41	82.56	140.03	0.50
KLMA_50257	SSY5	SPS-sensor serine protease component SSY5	1201.59	1028.04	1244.93	1123.65	1013.50	1151.32	-0.08
KLMA_50258	FBP26	fructose-2	676.04	816.61	606.63	730.04	693.36	701.86	0.02
KLMA_50259	VPS35	vacuolar protein sorting-associated protein 35	1127.52	1213.74	1129.20	1047.96	1079.21	1098.59	-0.11
KLMA_50260	CCP1	cytochrome c peroxidase	9663.31	8838.46	10888.83	1889.86	2160.11	1827.24	-2.32
KLMA_50261	SWE1	mitosis inhibitor protein kinase SWE1	991.14	991.12	1011.04	472.67	518.97	631.84	-0.88
KLMA_50262	RPS22	40S ribosomal protein S22	8785.04	6803.63	8958.10	2535.79	6071.73	4747.03	-0.88
KLMA_50264	RPS14	40S ribosomal protein S14	13596.12	10732.33	13716.10	3947.09	7701.93	6019.36	-1.11
KLMA_50265	SOP4	protein SOP4	205.75	247.22	211.95	439.03	501.27	484.04	1.10
KLMA_50266	BPH1	beige protein homolog 1	350.37	521.29	387.36	839.38	688.30	661.23	0.80
KLMA_50267		uncharacterized transporter YJL193W	1387.36	775.22	1251.02	1333.92	758.23	1012.16	-0.14
KLMA_50268		hypothetical protein	0.00	6.71	6.09	217.83	46.34	101.13	4.83
KLMA_50269	WRS1	tryptophanyl-tRNA synthetase	2162.16	1882.69	2100.05	1353.26	1675.69	1542.01	-0.43
KLMA_50270	RIB3	3,4-dihydroxy-2-butanone 4-phosphate synthase	825.36	657.77	803.96	385.20	624.28	500.46	-0.60
KLMA_50271	SUR4	elongation of fatty acids protein 3	3014.57	2346.93	3109.88	857.04	1709.39	1424.45	-1.09
KLMA_50272	ROM2	RHO1 GDP-GTP exchange protein 2	1235.69	1424.04	1416.68	1104.31	1091.01	1019.07	-0.34
KLMA_50273	ARC18	actin-related protein 2/3 complex subunit	533.78	455.29	489.69	603.04	705.15	708.77	0.45

Locus_tag	UniProt_gene	Product	Unique exon reads						log2 Fold Change
			KmWT.1	KmWT.2	KmWT.3	Kmmig1.1	Kmmig1.2	Kmmig1.3	
		3							
KLMA_50274	SSQ1	heat shock protein SSQ1	523.20	781.94	589.57	714.90	711.89	710.50	0.17
KLMA_50275		F-box domain	185.77	185.70	175.41	338.95	259.48	352.66	0.80
KLMA_50276	ART5	arrestin-related trafficking adapter 5	199.87	177.87	218.04	125.32	128.90	136.57	-0.61
KLMA_50277		conserved hypothetical protein	3022.80	2525.91	2308.35	1307.01	1882.09	2213.61	-0.54
KLMA_50278	VHT1	vitamin H transporter	3439.01	2320.08	3480.19	1762.86	931.78	991.41	-1.33
KLMA_50279	GRX8	glutaredoxin-like protein YLR364W	215.16	185.70	221.70	185.87	213.15	178.92	-0.11
KLMA_50280	SPT4	transcription elongation factor SPT4	205.75	195.76	213.17	107.66	128.90	103.72	-0.85
KLMA_50281	COX18	mitochondrial inner membrane protein COX18	460.89	390.41	395.89	236.34	269.59	217.82	-0.78
KLMA_50282		uncharacterized protein YLR363W-A	54.08	80.54	85.27	57.19	38.75	43.22	-0.66
KLMA_50283	ADE6	phosphoribosylformylglycinamidine synthase	9067.22	5630.17	8735.18	2668.68	4333.70	3997.64	-1.09
KLMA_50284	ERG25	c-4 methylsterol oxidase	5557.67	3149.00	5631.40	1211.97	1506.35	1565.34	-1.74
KLMA_50285	NMD4	nonsense-mediated decay protein 4	329.20	317.70	388.58	296.05	197.98	203.99	-0.57
KLMA_50286	SPR3	cell division control protein 3	21.16	8.95	9.75	53.83	19.38	24.20	1.29
KLMA_50287	PEF1	peflin or Penta-EF hand domain-containing protein 1	711.32	858.00	598.10	474.36	395.12	486.63	-0.68
KLMA_50288	RPS10A	40S ribosomal protein S10-A	7069.66	5803.56	6802.02	2761.20	5054.02	3466.06	-0.80
KLMA_50289		vacuolar membrane protein YOR292C	813.60	690.21	811.27	1675.39	984.86	1073.53	0.69
KLMA_50290	YPK9	probable cation-transporting ATPase 2	1792.99	1688.04	1858.86	1046.28	1309.21	1337.15	-0.53
KLMA_50291	RRP5	rRNA biogenesis protein RRP5	3988.07	3249.68	3634.89	1384.38	1463.38	1276.65	-1.40
KLMA_50292		conserved hypothetical	36.45	26.85	37.76	144.66	63.19	34.57	1.26

Locus_tag	UniProt_gene	Product	Unique exon reads						log2 Fold Change
			KmWT.1	KmWT.2	KmWT.3	Kmmig1.1	Kmmig1.2	Kmmig1.3	
		membrane protein							
KLMA_50293	MTF1	mitochondrial replication protein MTF1	169.30	173.39	154.70	167.37	141.54	190.16	0.00
KLMA_50294	TAF7	transcription initiation factor TFIID subunit 7	330.38	368.04	332.55	743.50	383.33	379.45	0.55
KLMA_50295	SNF2	transcription regulatory protein SNF2	1200.42	1490.04	1704.16	883.95	674.82	800.39	-0.90
KLMA_50296	sdh	uncharacterized oxidoreductase YMR226C	315.10	368.04	276.51	861.24	994.97	1096.86	1.62
KLMA_50297	ILV1	threonine dehydratase	3783.49	2231.71	3387.61	2413.84	2652.12	2922.38	-0.24
KLMA_50298		DSL1 super family conserved domain	785.39	520.17	806.40	296.05	428.82	426.13	-0.88
KLMA_50299	PEX13	peroxisomal membrane protein PAS20	350.37	307.63	297.22	439.87	380.80	470.21	0.43
KLMA_50300		hypothetical protein	67.02	202.48	87.71	2038.73	1149.14	1722.66	3.78
KLMA_50301	DLD1	D-lactate dehydrogenase [cytochrome]	904.14	957.56	1032.97	1419.71	817.20	961.16	0.14
KLMA_50302	HCR1	eukaryotic translation initiation factor 3 subunit J	357.42	317.70	341.08	203.54	323.51	364.76	-0.19
KLMA_50303		protein PAR32;Protein phosphorylated after rapamycin 32	139.91	82.78	125.47	179.99	191.24	166.82	0.63
KLMA_50304	GLT1	glutamate synthase [NADH]	3757.63	2794.39	3841.97	5164.10	9409.63	9838.94	1.23
KLMA_50305	UGA3	transcriptional activator protein UGA3	460.89	365.80	522.58	270.82	214.83	191.02	-0.99
KLMA_50306	UPS1	protein UPS1	714.84	623.09	627.34	926.85	934.31	949.06	0.52
KLMA_50307		predicted solute binding protein	297.46	337.83	289.91	304.46	312.56	307.71	0.00
KLMA_50308	NMT1	glycylpeptide N-tetradecanoyltransferase	1029.94	1104.11	995.21	656.87	843.32	769.27	-0.46
KLMA_50309	PWP1	periodic tryptophan	1298.00	993.36	1257.11	590.42	540.03	557.51	-1.07

Locus_tag	UniProt_gene	Product	Unique exon reads						log2 Fold Change
			KmWT.1	KmWT.2	KmWT.3	Kmmig1.1	Kmmig1.2	Kmmig1.3	
		protein 1							
KLMA_50310		protein UGX2	383.29	357.97	400.76	1276.73	637.76	783.97	1.24
KLMA_50311	NOP56	nucleolar protein 56	5085.03	4956.74	5028.43	1639.22	2397.69	2243.00	-1.26
KLMA_50312	PBA1	proteasome chaperone 1;Proteasome biogenesis-associated protein 1	323.33	352.37	302.10	518.93	485.27	494.41	0.62
KLMA_50313	YKE2	prefoldin subunit 6	154.02	132.00	109.63	127.84	202.19	155.58	0.30
KLMA_50314	COQ9	ubiquinone biosynthesis protein COQ9	496.16	547.02	482.38	228.77	358.05	312.90	-0.76
KLMA_50315	SFA1	S-(hydroxymethyl)glutathione dehydrogenase	1608.40	1972.18	1723.65	4343.23	4948.71	5735.85	1.50
KLMA_50316	NRP1	asparagine-rich protein; Protein ARP	275.12	357.97	313.06	359.13	215.67	267.95	-0.17
KLMA_50317	FAP7	putative adenylate kinase FAP7	146.97	162.20	116.94	234.66	217.36	215.22	0.65
KLMA_50318	CDC36	general negative regulator of transcription subunit 2	185.77	253.93	299.66	422.21	386.70	471.94	0.79
KLMA_50319	MSS51	protein MSS51	2215.07	1909.54	2120.76	625.75	629.33	656.91	-1.71
KLMA_50320		conserved hypothetical protein	559.65	594.00	622.46	379.32	311.72	234.24	-0.94
KLMA_50321	HMX1	heme-binding protein HMX1	4779.34	3357.07	5576.58	8445.07	4470.18	4188.66	0.32
KLMA_50322	CDC9	DNA ligase 1	545.54	489.97	465.32	233.81	249.37	324.13	-0.89
KLMA_50323		conserved hypothetical membrane protein	65.84	114.10	81.61	89.15	105.31	108.04	0.21
KLMA_50324		uncharacterized protein YPL250W-A	2526.64	2220.52	2566.59	963.01	1945.28	1422.73	-0.76
KLMA_50325	MRPL24	54S ribosomal protein L24	621.96	642.11	651.70	285.12	501.27	415.75	-0.67
KLMA_50326	GYP5	GTPase-activating protein GYP5	1314.46	1249.53	1099.97	748.54	641.13	572.20	-0.90
KLMA_50327		uncharacterized MFS-type	38.80	62.64	53.60	274.19	137.32	168.55	1.90

Locus_tag	UniProt_gene	Product	Unique exon reads						log2 Fold Change
			KmWT.1	KmWT.2	KmWT.3	Kmmig1.1	Kmmig1.2	Kmmig1.3	
		transporter C1271.10c							
KLMA_50328		putative amidase C550.07	59.96	96.20	65.78	164.01	114.58	105.45	0.79
KLMA_50329		GAL4-like Zn2Cys6 binuclear cluster DNA-binding conserved domain	56.43	57.05	68.22	456.69	292.34	370.81	2.62
KLMA_50330	LAC9	lactose regulatory protein LAC9	2480.79	3307.85	2567.81	1119.45	1420.42	1365.68	-1.10
KLMA_50331	SGS1	ATP-dependent helicase SGS1	457.36	600.72	543.28	536.60	456.62	455.51	-0.14
KLMA_50332	SEO1	probable transporter SEO1	4050.38	2246.25	4415.71	1445.78	1041.30	1303.45	-1.50
KLMA_50333	GCV2	glycine dehydrogenase [decarboxylating]	5528.28	3165.78	6010.24	1502.13	3320.20	3128.96	-0.89
KLMA_50334		WD repeat-containing protein YPL247C	346.84	462.00	353.26	399.50	437.25	437.36	0.13
KLMA_50335		transposon Ty1-H Gag-Pol polyprotein	7.05	13.42	10.96	37.01	22.75	20.74	1.35
KLMA_50336	MRPS17	37S ribosomal protein S17	432.67	502.27	409.29	435.67	440.62	437.36	-0.03
KLMA_50337	RBD2	rhomboid protein 2	376.23	419.49	465.32	625.75	716.95	726.06	0.71
KLMA_50338		uncharacterized protein YPL245W	292.76	242.75	305.75	65.60	63.19	66.56	-2.11
KLMA_50339	HUT1	UDP-galactose transporter homolog 1	191.64	195.76	200.99	365.86	508.01	437.36	1.16
KLMA_50340	SRP68	signal recognition particle subunit SRP68	920.60	786.41	995.21	605.56	909.03	773.60	-0.24
KLMA_50341		uncharacterized protein YMR187C	192.82	230.44	157.14	291.01	273.81	223.00	0.44
KLMA_50342	IQG1	ras GTPase-activating-like protein IQG1	429.14	393.76	375.18	574.44	774.24	728.65	0.79
KLMA_50343		tubulin-specific chaperone C	31.74	21.25	36.54	126.16	96.88	103.72	1.87
KLMA_50344	HSP82	ATP-dependent molecular chaperone HSC82	3738.82	4036.09	3459.48	2825.96	3673.20	3204.16	-0.21
KLMA_50345	ASP1	L-asparaginase 1	543.19	368.04	399.55	385.20	574.57	584.30	0.24

Locus_tag	UniProt_gene	Product	Unique exon reads						$\log_2$ Fold Change
			KmWT.1	KmWT.2	KmWT.3	Kmmig1.1	Kmmig1.2	Kmmig1.3	
KLMA_50346		UPF0676 protein C1494.01	162.25	129.76	149.83	190.08	164.28	188.43	0.30
KLMA_50347	QNS1	glutamine-dependent NAD(+) synthetase	1211.00	1226.04	1314.36	936.10	1335.33	1470.27	0.00
KLMA_50348	PPE1	protein phosphatase methylesterase 1	427.97	454.17	324.02	463.42	465.05	362.16	0.10
KLMA_50349	APE2	aminopeptidase 2	1486.12	2880.53	1241.27	962.17	713.58	879.91	-1.13
KLMA_50350	MRPL35	54S ribosomal protein L35	824.19	907.23	883.14	392.77	556.88	548.86	-0.80
KLMA_50351	TIM11	ATP synthase subunit e	311.57	412.78	450.71	339.79	477.68	388.09	0.04
KLMA_50352	PTC7	protein phosphatase 2C homolog 7	498.51	541.43	437.31	644.25	605.74	579.12	0.31
KLMA_50353	NMD2	nonsense-mediated mRNA decay protein 2	457.36	492.21	449.49	662.75	508.86	566.15	0.31
KLMA_50354	PEP7	vacuolar segregation protein PEP7	205.75	213.66	160.79	321.28	218.20	267.09	0.47
KLMA_50355		uncharacterized protein YHR078W	398.57	307.63	382.49	613.13	589.73	518.61	0.66
KLMA_50356	UTP4	U3 small nucleolar RNA-associated protein 4	1593.11	1428.52	1601.84	486.97	668.08	583.44	-1.41
KLMA_50357	YCG1	condensin complex subunit 3	453.83	536.95	459.23	557.62	484.42	490.09	0.08
KLMA_50358	IRE1	serine/threonine-protein kinase/endoribonuclease IRE1	928.83	1050.41	1034.19	761.16	710.21	725.19	-0.46
KLMA_50359		hypothetical protein	27.04	12.31	31.67	23.55	16.01	5.19	-0.66
KLMA_50360	KHT2	hexose transporter 2	24572.73	10372.13	20229.42	2731.76	1871.14	1318.14	-3.22
KLMA_50361	KHT2	hexose transporter 2	37623.31	13196.72	30289.93	7418.98	2379.16	1879.97	-2.80
KLMA_50362	KHT2	hexose transporter	42441.45	52820.44	44616.31	2624.94	754.86	922.27	-5.02
KLMA_50363	RAG1	low-affinity glucose transporter	371.53	408.31	434.87	116.91	63.19	49.27	-2.40
KLMA_50364	RAG1	low-affinity glucose transporter	75.25	144.31	114.50	910.03	272.96	369.94	2.22
KLMA_50365	SVF1	survival factor 1	2347.93	2570.66	2216.99	2164.04	2160.11	2118.53	-0.15
KLMA_50366	RRM3	uncharacterized ATP-dependent helicase YHR031C	217.51	581.70	221.70	299.42	224.10	201.39	-0.49

Locus_tag	UniProt_gene	Product	Unique exon reads						log2 Fold Change
			KmWT.1	KmWT.2	KmWT.3	Kmmig1.1	Kmmig1.2	Kmmig1.3	
KLMA_50368	PAN1	actin cytoskeleton-regulatory complex protein PAN1	1232.16	1446.42	1212.04	1047.12	1044.67	1213.55	-0.24
KLMA_50369	IST3	U2 snRNP component IST3	34.10	41.39	36.54	126.16	78.35	86.44	1.38
KLMA_50370		uncharacterized protein YER010C	70.54	195.76	90.14	244.75	177.76	172.01	0.74
KLMA_50371	DJP1	dnaJ-like protein 1	624.31	775.22	683.37	672.01	729.59	643.08	-0.03
KLMA_50372		uncharacterized protein YIR003W	259.84	400.48	246.06	318.76	199.67	313.76	-0.12
KLMA_50373	NTF2	nuclear transport factor 2	1520.22	1162.28	1363.08	1019.36	1115.44	1026.85	-0.36
KLMA_50374	SEC3	exocyst complex component SEC3	779.51	1106.35	866.09	692.19	734.64	798.66	-0.31
KLMA_50375	MPH1	ATP-dependent DNA helicase MPH1	257.48	378.10	287.48	373.43	269.59	283.51	0.00
KLMA_50376	TMA20	translation machinery-associated protein 20	661.94	548.14	616.37	425.58	548.45	481.44	-0.33
KLMA_50377	PAC2	protein PAC2	139.91	163.32	142.52	189.24	135.64	158.18	0.12
KLMA_50378	NUG1	nuclear GTP-binding protein NUG1	832.42	845.70	935.52	402.87	417.03	420.94	-1.07
KLMA_50379	HAK1	high affinity potassium transporter	423.26	410.54	389.80	78279.01	46700.20	53754.15	7.19
KLMA_50380	SUA7	transcription initiation factor IIB	1348.56	1180.18	1242.49	1388.59	1254.45	1278.38	0.06
KLMA_50381	YND1	Golgi apyrase	932.35	752.85	863.65	669.48	539.19	567.02	-0.52
KLMA_50382	FMP52	protein FMP52	485.58	445.22	503.09	938.62	908.19	815.95	0.89
KLMA_50383		dynactin subunit 5	21.16	24.61	21.93	58.87	47.18	48.40	1.19
KLMA_50384	PMI40	mannose-6-phosphate isomerase	3212.09	2273.10	2721.29	1445.78	1953.70	2152.24	-0.56
KLMA_50385	NOP16	nucleolar protein 16	519.67	464.24	543.28	309.51	338.68	362.16	-0.60
KLMA_50386	SGN1	nuclear localization sequence-binding protein	1089.90	1155.57	1177.93	639.20	817.20	792.61	-0.61
KLMA_50387	IRC22	uncharacterized protein YEL001C	352.72	393.76	370.31	339.79	482.74	436.50	0.17
KLMA_50388		ankyrin repeat-containing protein	377.41	325.53	414.16	573.60	583.84	548.00	0.61

Locus_tag	UniProt_gene	Product	Unique exon reads						log $_2$ Fold Change
			KmWT.1	KmWT.2	KmWT.3	Kmmig1.1	Kmmig1.2	Kmmig1.3	
		YIL001W							
KLMA_50389	INP51	inositol-1	567.88	545.90	540.85	1127.02	821.42	930.91	0.80
KLMA_50390	WBP1	dolichyl-diphosphooligosaccharide--protein glycosyltransferase subunit WBP1	881.80	941.90	883.14	895.73	1258.66	1289.62	0.35
KLMA_50391	GIM4	prefoldin subunit 2	121.10	101.80	141.30	109.34	160.07	124.47	0.11
KLMA_50392	VAB2	protein VAB2	281.00	219.26	287.48	656.87	437.25	508.24	1.03
KLMA_50393	CFD1	cytosolic Fe-S cluster assembly factor CFD1	364.48	289.73	348.38	518.09	472.63	507.38	0.58
KLMA_50394	BET1	protein transport protein BET1	136.38	120.81	113.29	97.56	128.90	135.70	-0.03
KLMA_50395	EPS1	putative protein disulfide-isomerase YIL005W	1196.89	1105.23	1168.18	686.30	672.30	739.02	-0.73
KLMA_50396	YIA6	uncharacterized mitochondrial carrier YIL006W	352.72	369.15	366.66	650.98	480.21	472.80	0.56
KLMA_50397		gti1_Pac2 super family conserved domain	369.18	402.71	394.67	157.28	100.25	95.94	-1.72
KLMA_50398	GCN4	general control protein GCN4	16814.09	11762.61	15484.82	6682.21	8114.74	8449.92	-0.92
KLMA_50399	NAS2	probable 26S proteasome regulatory subunit p27	141.09	95.09	146.18	60.56	75.82	60.50	-0.96
KLMA_50400	URM1	ubiquitin-related modifier 1	209.28	154.37	169.32	167.37	162.60	135.70	-0.19
KLMA_50402	LEU4	2-isopropylmalate synthase	3732.94	2745.17	3394.92	4968.97	4957.98	5424.68	0.64
KLMA_50403	EST3	telomere replication protein EST3	25.87	29.08	13.40	37.85	36.23	53.59	0.90
KLMA_50404	APM1	AP-1 complex subunit mu-1-I	693.68	855.77	730.88	569.40	656.29	624.93	-0.30
KLMA_50406	DOT5	peroxiredoxin DOT5	323.33	389.29	320.37	359.97	512.23	485.77	0.39
KLMA_50407		UPF0662 protein YPL260W	1682.47	2254.08	1911.24	1156.46	1067.42	1115.02	-0.81
KLMA_50408		protein SLG1	1034.64	1029.16	974.50	1025.25	756.54	847.07	-0.21

Locus_tag	UniProt_gene	Product	Unique exon reads						log$_2$ Fold Change
			KmWT.1	KmWT.2	KmWT.3	Kmmig1.1	Kmmig1.2	Kmmig1.3	
KLMA_50409	MUC1	flo11 super family	47201.97	26273.74	36439.03	1000.86	1802.90	2292.27	-4.43
KLMA_50410		bZIP_1 super family conserved domain	178.71	132.00	192.46	100.09	69.93	77.79	-1.02
KLMA_50411	GAS5	hypothetical protein	5872.76	3353.71	5457.21	2630.83	3947.85	3655.35	-0.52
KLMA_50412	ADI1	1,2-dihydroxy-3-keto-5-methylthiopentene dioxygenase; Acireductone dioxygenase	600.80	389.29	525.01	389.41	276.33	319.81	-0.62
KLMA_50413		uncharacterized membrane protein YMR010W	485.58	442.99	501.87	336.42	407.76	414.03	-0.30
KLMA_50414	PET127	putative mitochondrial translation system component PET127	323.33	270.71	285.04	282.60	286.44	262.76	-0.08
KLMA_50415	AIM17	uncharacterized oxidoreductase YHL021C	44.68	85.02	60.91	473.52	355.53	359.57	2.64
KLMA_50416	SPO11	meiosis-specific protein SPO11	7.05	11.19	8.53	21.87	7.58	3.46	0.30
KLMA_50417	ERP4	protein ERP4	1046.40	931.84	1012.26	714.90	1028.66	931.77	-0.16
KLMA_50418	RPN7	26S proteasome regulatory subunit RPN7	728.95	1032.51	677.28	764.52	894.71	833.24	0.03
KLMA_50419	CTT1	catalase T	162.25	253.93	177.85	2588.78	1269.61	1759.82	3.24
KLMA_50422	NNF2	protein NNF2	687.80	664.48	757.67	298.58	351.31	356.11	-1.07
KLMA_50424	UTP22	U3 small nucleolar RNA-associated protein 22	1908.21	1688.04	2001.38	826.76	969.69	1085.63	-0.96
KLMA_50425		uncharacterized membrane protein YPR109W	151.67	112.98	114.50	216.99	173.55	219.55	0.69
KLMA_50426	RPC40	DNA-directed RNA polymerases I and III subunit RPAC1	1473.19	909.46	1321.67	298.58	476.84	494.41	-1.54
KLMA_50427	PRP31	pre-mRNA-processing factor 31	118.75	215.90	118.16	115.23	57.29	96.81	-0.75
KLMA_50428	DBF2	cell cycle protein kinase DBF2	150.49	180.10	116.94	146.34	191.24	201.39	0.27

Locus_tag	UniProt_gene	Product	Unique exon reads						log$_2$ Fold Change
			KmWT.1	KmWT.2	KmWT.3	Kmmig1.1	Kmmig1.2	Kmmig1.3	
KLMA_50429	DRN1	CWF19-like protein DRN1	244.55	195.76	198.55	152.23	141.54	174.60	-0.45
KLMA_50430	MRD1	multiple RNA-binding domain-containing protein 1	1046.40	1059.36	1001.30	545.01	490.32	518.61	-1.00
KLMA_50431	VAS1	valyl-tRNA synthetase	3167.41	2449.84	3005.12	790.60	1157.56	1208.37	-1.45
KLMA_50432	RRP46	exosome complex component RRP46	634.89	528.00	629.77	313.71	403.55	333.64	-0.77
KLMA_50433	TPC1	mitochondrial thiamine pyrophosphate carrier 1	118.75	111.87	91.36	91.68	87.62	90.76	-0.25
KLMA_50434	PIS1	CDP-diacylglycerol-inositol 3-phosphatidyltransferase	932.35	921.77	878.27	1281.77	1031.19	939.55	0.25
KLMA_50435		uncharacterized TLC domain-containing protein YPR114W	771.28	572.75	657.79	903.30	980.64	922.27	0.49
KLMA_50436	RGC1	protein ASK10	1403.82	1540.38	1506.82	920.12	738.01	881.64	-0.81
KLMA_50437		NADH-dependent flavin oxidoreductase (predicted); FlaRed super family conserved domain	38.80	67.12	26.80	113.54	122.16	81.25	1.25
KLMA_50438	RRG8	uncharacterized protein YPR116W	58.79	72.71	60.91	126.16	100.25	83.84	0.69
KLMA_50439	ESP1	separin	398.57	488.85	421.47	545.01	451.57	525.53	0.22
KLMA_50440	TEL2	telomere length regulation protein TEL2	176.36	130.88	176.63	506.32	303.29	296.47	1.19
KLMA_50441		uncharacterized protein YPR117W	1199.24	1023.57	1163.31	771.25	623.43	652.59	-0.73
KLMA_50442	MDR1	GTPase-activating protein GYP2	1408.52	1315.53	1328.98	750.22	706.84	677.65	-0.92
KLMA_50443	PCP1	rhomboid protein 1	283.35	284.14	241.19	355.77	347.10	382.04	0.42
KLMA_50444	GTF1	glutamyl-tRNA(Gln) amidotransferase subunit F	204.58	180.10	163.23	254.84	240.11	246.34	0.44
KLMA_50445	NOP7	pescadillo homolog	2472.56	2363.71	2321.75	799.85	1010.97	930.91	-1.38

Locus_tag	UniProt_gene	Product	Unique exon reads						log_2 Fold Change
			KmWT.1	KmWT.2	KmWT.3	Kmmig1.1	Kmmig1.2	Kmmig1.3	
KLMA_50446	SRB5	mediator of RNA polymerase II transcription subunit 18	116.40	137.59	110.85	185.87	256.96	241.15	0.91
KLMA_50447	VMA21	vacuolar ATPase assembly integral membrane protein VMA21	97.59	86.14	101.10	87.47	117.95	90.76	0.06
KLMA_50448		v0 assembly protein 1	1343.86	1227.16	1220.56	1158.14	1327.74	1410.63	0.04
KLMA_50449		fungal_trans super family conserved domain	322.15	158.85	313.06	349.04	280.54	391.55	0.36
KLMA_50450	OPT2	oligopeptide transporter 2	299.81	249.46	333.77	788.91	640.28	786.56	1.33
KLMA_50451		acyl-coenzyme A:6-aminopenicillanic-acid-acyltransferase 40 kDa form	0.00	6.71	0.00	57.19	39.60	69.15	4.58
KLMA_50452	MRI1	methylthioribose-1-phosphate isomerase	553.77	413.90	533.54	220.36	433.88	337.10	-0.60
KLMA_50453	fabG	3-oxoacyl-(acyl-carrier-protein) reductase	116.40	52.58	133.99	152.23	162.60	140.89	0.59
KLMA_50454	CLB2	G2/mitotic-specific cyclin-2	685.45	666.72	877.05	451.65	652.92	644.81	-0.35
KLMA_50455	CLB5	S-phase entry cyclin-5	141.09	174.51	181.50	61.40	116.26	127.92	-0.70
KLMA_50456		hypothetical protein	12.93	4.47	9.75	47.94	50.55	70.01	2.64
KLMA_50457	AXL1	putative protease AXL1	51.73	50.34	69.43	211.11	125.53	141.75	1.48
KLMA_50458		uncharacterized protein YGR111W	52.91	97.32	53.60	384.36	308.35	394.15	2.41
KLMA_50459	DUT1	deoxyuridine 5'-triphosphate nucleotidohydrolase	216.33	198.00	224.14	110.18	136.48	146.08	-0.70
KLMA_50460		mediator of RNA polymerase II transcription subunit 22	2.35	14.54	6.09	6.73	6.74	12.97	0.19
KLMA_50461	DSN1	kinetochore-associated protein DSN1	158.72	128.64	174.19	82.42	106.15	129.65	-0.54
KLMA_50462	TRS20	transport protein	29.39	33.56	40.20	60.56	82.56	84.71	1.14

Locus_tag	UniProt_gene	Product	Unique exon reads						log2 Fold Change
			KmWT.1	KmWT.2	KmWT.3	Kmmig1.1	Kmmig1.2	Kmmig1.3	
		particle 20 kDa subunit							
KLMA_50463	MTC4	conserved hypothetical membrane protein	181.06	193.53	133.99	429.78	351.31	365.62	1.17
KLMA_50464		uncharacterized protein YBR255C-A	165.78	134.24	194.90	127.00	187.87	126.20	-0.16
KLMA_50465	STS1	protein DBF8	812.43	664.48	869.74	540.80	481.05	542.81	-0.58
KLMA_50466	SQT1	ribosome assembly protein SQT1	1120.47	904.99	1174.27	764.52	766.65	909.30	-0.39
KLMA_50467	RIB5	riboflavin synthase alpha chain	685.45	542.55	697.99	416.32	567.83	551.46	-0.33
KLMA_50468	BAR1	aspartic proteinase yapsin-3	114.05	99.56	118.16	319.60	176.08	202.26	1.07
KLMA_50469		HSP70 co-chaperone SNL1	197.52	214.78	222.92	154.75	171.02	163.36	-0.38
KLMA_50470	VID28	vacuolar import and degradation protein 28	435.02	663.36	416.60	1183.37	852.59	828.92	0.92
KLMA_50471	MET10	sulfite reductase [NADPH] flavoprotein component	13156.40	8197.47	12020.47	3100.98	5016.11	5264.78	-1.32
KLMA_50472	SMC2	structural maintenance of chromosomes protein 2	543.19	547.02	544.50	405.39	452.41	410.57	-0.37
KLMA_50473	RPL2	60S ribosomal protein L2	42509.64	33699.35	41119.07	12222.26	24332.43	20295.89	-1.05
KLMA_50474		60S ribosomal protein L29	2367.92	2340.22	2442.34	1262.43	1898.10	1386.42	-0.65
KLMA_50475	QCR6	cytochrome b-c1 complex subunit 6	997.02	1138.79	1233.96	513.05	1119.65	814.22	-0.46
KLMA_50476	FAF1	protein FAF1	176.36	135.36	172.97	154.75	104.47	114.96	-0.37
KLMA_50477	HIS6	1-(5-phosphoribosyl)-5-[(5-phosphoribosylamino)methylideneamino] imidazole-4-carboxamide isomerase	850.05	621.97	649.26	463.42	452.41	528.98	-0.55
KLMA_50478	RPB3	DNA-directed RNA polymerase II	882.97	821.09	823.45	546.69	695.04	693.21	-0.39

Locus_tag	UniProt_gene	Product	Unique exon reads						log2 Fold Change
			KmWT.1	KmWT.2	KmWT.3	Kmmig1.1	Kmmig1.2	Kmmig1.3	
		subunit RPB3							
KLMA_50479		hypothetical protein	151.67	130.88	163.23	117.75	162.60	165.96	0.00
KLMA_50480	TIM44	mitochondrial import inner membrane translocase subunit TIM44	901.78	982.18	961.10	459.22	740.54	694.08	-0.59
KLMA_50481		uncharacterized protein YGR130C	1326.22	2194.79	1586.00	2086.67	1940.23	2045.06	0.25
KLMA_50482	NCE102	non-classical export protein 2	545.54	573.87	518.92	2565.23	1583.86	1450.39	1.77
KLMA_50483	PHB1	prohibitin-1	824.19	1024.68	797.87	870.50	832.37	806.44	-0.08
KLMA_50484	APC2	anaphase-promoting complex subunit 2	110.52	127.53	114.50	325.49	271.28	225.60	1.22
KLMA_50485	DCN1	defective in cullin neddylation protein 1	49.38	104.03	68.22	206.90	193.77	152.99	1.32
KLMA_50486	TAF12	transcription initiation factor TFIID subunit 12	317.45	398.24	342.29	171.58	231.68	259.31	-0.68
KLMA_50487	DIP2	U3 small nucleolar RNA-associated protein 12	1911.73	1421.80	1786.99	648.46	816.36	788.29	-1.18
KLMA_50488	INO1	inositol-3-phosphate synthase	2411.42	739.43	1874.70	12071.71	9880.57	11813.98	2.75
KLMA_50489	ZRT2	zinc-regulated transporter 2	22271.83	12705.63	16579.92	14701.71	6265.50	4092.71	-1.04
KLMA_50490	SWI5	metallothionein expression activator	463.24	351.26	331.33	381.84	675.67	564.42	0.50
KLMA_50491		uncharacterized protein YLR132C	157.55	101.80	121.81	196.81	211.46	229.92	0.74
KLMA_50492	CKI1	choline kinase	2262.10	1096.28	1581.13	696.40	1112.07	1025.12	-0.80
KLMA_50493	KGD2	dihydrolipoyllysine-residue succinyltransferase component of 2-oxoglutarate dehydrogenase complex	906.49	1396.08	935.52	810.78	1155.88	1291.34	0.01
KLMA_50494	SLX4	structure-specific endonuclease subunit SLX4	197.52	259.53	203.43	132.89	120.47	147.80	-0.72
KLMA_50495	NUM1	nuclear migration protein NUM1	1736.55	1983.37	1788.21	915.07	1016.03	1012.16	-0.90

Locus_tag	UniProt_gene	Product	Unique exon reads						log2 Fold Change
			KmWT.1	KmWT.2	KmWT.3	Kmmig1.1	Kmmig1.2	Kmmig1.3	
KLMA_50496	VPS70	vacuolar protein sorting-associated protein 70	694.86	965.40	688.24	1813.32	1703.49	2044.20	1.24
KLMA_50497	CTH1	mRNA decay factor CTH1	1049.93	951.97	1004.95	853.67	422.08	362.16	-0.88
KLMA_50498	GIR2	protein GIR2	250.43	314.34	243.63	321.28	331.09	350.06	0.31
KLMA_50500	ENT5	epsin-like protein	1620.15	1685.81	1531.18	1045.44	945.26	1011.29	-0.69
KLMA_50501	CPR1	peptidyl-prolyl cis-trans isomerase	4932.18	4467.89	4413.27	2214.51	2973.10	2981.16	-0.76
KLMA_50502		DNA-directed RNA polymerase I subunit RPA14	812.43	589.53	760.11	291.01	231.68	224.73	-1.53
KLMA_50503	HOM2	aspartate-semialdehyde dehydrogenase	10531.00	5122.30	8735.18	4356.68	5503.06	5665.84	-0.65
KLMA_50504	RKM5	uncharacterized protein YLR137W	31.74	42.51	47.51	100.09	66.56	115.82	1.21
KLMA_50505	SAC3	nuclear mRNA export protein SAC3	431.49	601.83	566.43	390.25	442.30	470.21	-0.30
KLMA_50506	NHA1	Na(+)/H(+) antiporter	3040.43	2746.29	2972.23	1427.28	1322.69	1310.36	-1.11
KLMA_50507	SSY1	SPS-sensor component SSY1	552.59	494.44	568.86	809.94	550.14	659.50	0.32
KLMA_50508		UPF0661 TPR repeat-containing protein YDR161W	890.03	757.33	884.36	550.05	631.02	547.14	-0.55
KLMA_50509	NBP2	SH3 super family	238.67	228.20	192.46	433.99	354.68	360.44	0.80
KLMA_50510	CWC15	pre-mRNA-splicing factor CWC15	126.98	123.05	155.92	206.06	177.76	187.56	0.49
KLMA_50511	SLS1	sigma-like sequence protein 1	631.37	647.70	617.59	582.85	413.66	447.74	-0.39
KLMA_50512		RNA polymerase I-specific transcription initiation factor RRN5	102.29	93.97	97.45	191.76	89.30	116.69	0.44
KLMA_50513	PUT1	proline dehydrogenase	605.50	560.44	589.57	1786.41	1253.61	1299.99	1.31
KLMA_50514		putative ribonuclease YLR143W	422.09	397.12	454.36	374.27	416.18	398.47	-0.10
KLMA_50515		hypothetical protein	176.36	177.87	185.16	163.17	138.17	178.06	-0.17
KLMA_50516	SEC1	protein transport protein SEC1	444.43	547.02	587.14	567.71	532.45	550.59	0.06

Locus_tag	UniProt_gene	Product	Unique exon reads						log$_2$ Fold Change
			KmWT.1	KmWT.2	KmWT.3	Kmmig1.1	Kmmig1.2	Kmmig1.3	
KLMA_50517	ACF2	endo-1,3-beta-glucanase	306.87	434.04	331.33	663.60	524.02	626.66	0.76
KLMA_50518		ribonuclease MRP protein subunit RMP1	44.68	55.93	74.31	161.48	88.46	96.81	0.99
KLMA_50519	SPE4	spermine synthase	426.79	371.39	401.98	264.93	278.86	292.15	-0.52
KLMA_50520	TRM82	tRNA (guanine-N(7)-)-methyltransferase subunit TRM82	660.76	644.34	695.55	386.05	464.20	424.40	-0.65
KLMA_50521		hypothetical protein	7.05	10.07	4.87	8.41	10.95	11.24	0.47
KLMA_50522	SEC5	exocyst complex component SEC5	794.79	864.72	803.96	633.32	807.94	739.89	-0.18
KLMA_50523	SMD3	small nuclear ribonucleoprotein Sm D3	69.37	42.51	64.56	55.51	88.46	55.32	0.18
KLMA_50524	PEP3	vacuolar membrane protein PEP3	346.84	449.70	365.44	603.04	563.62	432.18	0.46
KLMA_50525	TAF10	transcription initiation factor TFIID subunit 10	370.35	382.58	325.24	619.86	521.49	488.36	0.60
KLMA_50526	CDC37	hsp90 co-chaperone Cdc37	611.38	691.33	626.12	694.71	705.15	624.93	0.07
KLMA_50527		uncharacterized protein YLR149C	1036.99	1190.24	883.14	1513.91	984.01	919.67	0.14
KLMA_50528	STB3	protein STB3	295.11	269.59	209.52	619.86	439.77	519.48	1.03
KLMA_50529		HYALURONIC ACID-BINDING PROTEIN 4	6136.13	5274.44	6094.29	1938.64	3933.53	3364.06	-0.92
KLMA_50530	PCD1	peroxisomal coenzyme A diphosphatase 1	111.69	105.15	92.58	222.88	197.98	220.41	1.05
KLMA_50531		uncharacterized transporter YLR152C	4445.43	3486.83	4045.40	1307.01	1369.87	1412.35	-1.55
KLMA_50533	ACS2	acetyl-coenzyme A synthetase 2	11135.32	12327.53	12689.22	2937.82	4101.18	4071.97	-1.70
KLMA_50534	SEC7	protein transport protein SEC7	2025.78	2533.74	2062.29	1883.97	2002.57	2100.38	-0.15
KLMA_50535	URC1	putative GTP cyclohydrolase URC1	56.43	58.17	54.82	320.44	182.82	261.90	2.17
KLMA_50536	ARG82	inositol	132.86	108.51	103.54	184.19	167.65	170.28	0.60

Locus_tag	UniProt_gene	Product	Unique exon reads						$\log_2$ Fold Change
			KmWT.1	KmWT.2	KmWT.3	Kmmig1.1	Kmmig1.2	Kmmig1.3	
		polyphosphate multikinase							
KLMA_50537	SUP35	eukaryotic peptide chain release factor GTP-binding subunit	3183.87	2773.13	2933.25	1321.30	2065.75	1836.75	-0.77
KLMA_50546	HMO1	high mobility group protein 1	1432.04	1311.06	1310.70	795.64	1078.37	952.52	-0.52
KLMA_50547	RSM24	37S ribosomal protein S24	1268.61	1054.89	997.65	645.09	823.94	800.39	-0.55
KLMA_50548	NGG1	chromatin-remodeling complexes subunit NGG1	581.99	588.41	565.21	477.72	528.23	553.19	-0.15
KLMA_50549	MAS1	mitochondrial-processing peptidase subunit beta	852.40	987.77	730.88	692.19	759.91	679.38	-0.27
KLMA_50550	SDH4	succinate dehydrogenase [ubiquinone] cytochrome b small subunit	787.74	630.92	800.31	436.51	925.04	726.06	-0.09
KLMA_50551	CSN9	COP9 signalosome complex subunit 9	72.90	77.19	93.80	53.83	60.66	44.95	-0.61
KLMA_50552		uncharacterized protein YDR179W-A	325.68	352.37	300.88	396.98	354.68	350.06	0.17
KLMA_50553	PUS5	pseudouridylate synthase PUS5	165.78	118.58	198.55	192.60	156.70	193.62	0.17
KLMA_50554	SCC2	sister chromatid cohesion protein 2	837.12	589.53	775.95	545.01	572.04	545.41	-0.41
KLMA_50555	SAS4	something about silencing protein 4	226.92	276.31	236.32	309.51	286.44	259.31	0.21
KLMA_50556	CDC1	cell division control protein 1	681.92	777.46	600.54	537.44	687.46	650.86	-0.14
KLMA_50557	SEC10	exocyst complex component SEC10	840.65	917.29	875.83	866.29	941.89	892.88	0.04
KLMA_50558	PLP1	phosducin-like protein 1	114.05	85.02	154.70	140.46	144.06	111.50	0.17
KLMA_50559	ubi3	ubiquitin-40S ribosomal protein S27a	16460.20	12994.24	15834.42	5628.36	10828.36	8153.45	-0.88
KLMA_50561	ATC1	protein ATC1/LIC4	376.23	344.54	409.29	158.12	204.72	254.98	-0.87
KLMA_50562	UPS2	protein MSF1	798.32	602.95	823.45	743.50	667.24	624.06	-0.13

Locus_tag	UniProt_gene	Product	Unique exon reads						log2 Fold Change
			KmWT.1	KmWT.2	KmWT.3	Kmmig1.1	Kmmig1.2	Kmmig1.3	
KLMA_50563	ERF2	palmitoyltransferase ERF2	466.76	393.76	506.74	648.46	584.68	560.97	0.39
KLMA_50564		uncharacterized protein YDR186C	513.79	431.80	392.24	955.44	632.70	765.82	0.82
KLMA_50565	CDD1	cytidine deaminase	102.29	120.81	73.09	216.15	186.19	173.74	0.96
KLMA_50566	MAP1	methionine aminopeptidase 1	1590.76	1459.84	1553.11	783.03	704.31	719.14	-1.06
KLMA_50567	CCT6	T-complex protein 1 subunit zeta	1878.81	1908.42	1716.34	1283.46	1813.85	1458.16	-0.27
KLMA_50568	SLY1	protein SLY1	993.49	1051.53	917.25	729.20	864.38	803.85	-0.31
KLMA_50569	RVB1	ruvB-like helicase 1	1437.92	1522.48	1404.50	1130.38	1473.49	1421.86	-0.12
KLMA_50570	HST4	NAD-dependent histone deacetylase HST4	245.73	251.70	215.61	389.41	345.42	364.76	0.62
KLMA_50571		GPN-loop GTPase 3 homolog KLLA0D18557g	543.19	519.05	576.17	340.63	386.70	386.37	-0.56
KLMA_50572		nucleoporin NUP42	685.45	495.56	644.39	604.72	564.46	526.39	-0.11
KLMA_50573	ARV1	protein ARV1	604.32	427.32	562.77	437.35	422.92	372.54	-0.37
KLMA_50574		uncharacterized membrane protein YLR241W	1737.73	1411.74	1594.53	1119.45	1047.20	966.35	-0.60
KLMA_50575	VPS34	phosphatidylinositol 3-kinase VPS34	417.38	468.71	494.56	592.95	609.11	647.40	0.42
KLMA_50576	MSS116	ATP-dependent RNA helicase MSS116	1600.17	1899.47	2093.96	978.15	1131.45	1101.19	-0.80
KLMA_50577	REF2	RNA end formation protein 2	580.81	629.80	617.59	590.42	654.60	611.96	0.02
KLMA_50578	CAB5	dephospho-CoA kinase CAB5	724.25	548.14	503.09	924.32	711.89	643.94	0.36
KLMA_50579	CBS2	cytochrome B translational activator protein CBS2	275.12	268.48	215.61	216.99	229.15	206.58	-0.22
KLMA_50580	RKM2	ribosomal N-lysine methyltransferase 2	470.29	469.83	515.27	714.90	624.28	599.86	0.41
KLMA_50581	LIPB	octanoyltransferase	125.80	161.09	170.54	220.36	240.11	268.81	0.67
KLMA_50582	VPS64	factor arrest protein 10	376.23	430.68	352.04	558.46	528.23	460.70	0.42
KLMA_50583	SPC19	DASH complex	186.94	143.19	152.27	264.09	276.33	210.04	0.64

Locus_tag	UniProt_gene	Product	Unique exon reads						log2 Fold Change
			KmWT.1	KmWT.2	KmWT.3	Kmmig1.1	Kmmig1.2	Kmmig1.3	
		subunit SPC19							
KLMA_50584	RAV2	regulator of V-ATPase in vacuolar membrane protein 2	373.88	448.58	347.17	470.15	452.41	440.82	0.22
KLMA_50585	COQ4	ubiquinone biosynthesis protein COQ4	681.92	726.00	702.86	719.11	686.62	700.13	0.00
KLMA_50586	PSY2	serine/threonine-protein phosphatase 4 regulatory subunit 3	886.50	947.50	1120.68	576.97	550.98	520.34	-0.84
KLMA_50587	SPS19	peroxisomal 2	31.74	43.63	26.80	337.26	479.37	633.57	3.82
KLMA_50588	GCS1	ADP-ribosylation factor GTPase-activating protein GCS1	891.20	865.84	850.25	1093.38	1067.42	1045.00	0.30
KLMA_50589	dpp1	probable diacylglycerol pyrophosphate phosphatase 1	41.15	44.75	36.54	207.74	108.68	94.21	1.74
KLMA_50590	RTT106	histone chaperone RTT106	238.67	232.68	207.08	179.99	233.37	199.67	-0.15
KLMA_50591	RIO2	serine/threonine-protein kinase RIO2	487.93	613.02	573.74	380.16	412.81	471.94	-0.41
KLMA_50592	SSB	heat shock protein SSB	65765.55	43588.22	57024.15	18093.69	33852.42	31772.77	-0.99
KLMA_50594		conserved hypothetical protein	48.20	35.80	64.56	52.15	57.29	60.50	0.20
KLMA_50595	PTP1	tyrosine-protein phosphatase 1	391.52	399.36	414.16	428.94	406.92	426.13	0.07
KLMA_50596	BRE4	protein BRE4	3029.85	2662.39	3336.45	1153.93	991.60	1051.92	-1.50
KLMA_50597		dolichyl-diphosphooligosaccharide--protein glycosyltransferase	2.35	1.12	0.00	2.52	1.68	0.00	0.28
KLMA_50598		subunit OST4	3.53	2.24	3.65	6.73	4.21	6.91	0.93
KLMA_50599	VID27	vacuolar import and degradation protein 27	232.79	255.05	292.35	343.99	240.11	284.37	0.16
KLMA_50600	RRG9	uncharacterized protein YNL213C	192.82	196.88	203.43	208.58	139.85	113.23	-0.36
KLMA_50601		peroxisomal membrane protein	170.48	135.36	185.16	635.84	432.19	518.61	1.69

Locus_tag	UniProt_gene	Product	Unique exon reads						log$_2$ Fold Change
			KmWT.1	KmWT.2	KmWT.3	Kmmig1.1	Kmmig1.2	Kmmig1.3	
		PEX17							
KLMA_50602		PAPA-1 super family conserved domain	340.96	368.04	304.53	749.38	524.86	556.64	0.85
KLMA_50603	GYP7	GTPase-activating protein GYP7	592.57	877.02	551.81	1183.37	925.88	857.44	0.55
KLMA_50604	RAP1	DNA-binding protein RAP1	1497.88	1589.60	1623.76	1106.83	1048.88	1005.24	-0.58
KLMA_50605		putative metallophosphoesterase YNL217W	473.82	502.27	447.05	698.92	558.56	560.97	0.35
KLMA_50606	YPD1	phosphorelay intermediate protein YPD1	199.87	183.46	200.99	260.73	305.82	233.38	0.45
KLMA_50607	MGS1	DNA-dependent ATPase MGS1	281.00	267.36	271.64	486.97	493.69	475.39	0.83
KLMA_50608	PHO13	4-nitrophenylphosphatase	3814.06	2747.41	3286.50	1730.06	2028.69	1830.70	-0.82
KLMA_50609	ALG9	probable mannosyltransferase ALG9	2632.46	2106.42	2385.09	1432.32	1498.77	1292.21	-0.75
KLMA_50610		adenylosuccinate synthetase	10689.72	8220.96	11199.45	4816.74	9253.77	6963.23	-0.52
KLMA_50611	POP1	ribonucleases P/MRP protein subunit POP1	1325.05	1164.52	1210.82	1686.32	1181.15	1133.17	0.11
KLMA_50612	AIM6	uncharacterized protein YDL237W	1192.19	630.92	970.85	942.83	620.06	650.86	-0.34
KLMA_50613	GUD1	probable guanine deaminase	148.14	256.17	109.63	1255.70	786.87	764.09	2.45
KLMA_50614	SSU72	RNA polymerase II subunit A C-terminal domain phosphatase SSU72	151.67	161.09	168.10	255.68	258.64	278.32	0.72
KLMA_50615	ATG4	probable cysteine protease ATG4	365.65	417.26	334.98	1183.37	901.45	919.67	1.43
KLMA_50616	SQS1	protein SQS1	576.11	532.48	495.78	677.05	640.28	622.33	0.27
KLMA_50617		Golgin IMH1	320.97	403.83	421.47	638.36	491.16	547.14	0.55
KLMA_50618	JJJ1	J protein JJJ1	450.30	488.85	430.00	624.91	478.53	535.90	0.26
KLMA_50619	URE2	protein URE2	913.54	828.92	968.41	733.40	901.45	847.07	-0.13

Locus_tag	UniProt_gene	Product	Unique exon reads						$\log_2$ Fold Change
			KmWT.1	KmWT.2	KmWT.3	Kmmig1.1	Kmmig1.2	Kmmig1.3	
KLMA_50620	ELA1	elongin-A	202.23	211.42	272.86	341.47	267.07	245.48	0.32
KLMA_50621	PDR16	phosphatidylinositol transfer protein PDR16	906.49	710.34	952.57	1660.25	1577.12	1416.68	0.86
KLMA_50622	CSL4	exosome complex component CSL4	262.19	303.15	334.98	376.79	375.74	436.50	0.40
KLMA_50623	BNI4	protein BNI4	643.12	609.66	676.06	674.53	708.52	699.26	0.11
KLMA_50624		uncharacterized globin-like protein YNL234W	1087.55	1060.48	1168.18	615.65	767.50	796.07	-0.61
KLMA_50625	FET4	low-affinity Fe(2+) transport protein	387.99	306.51	425.13	393.62	286.44	326.73	-0.15
KLMA_50626	SIN4	mediator of RNA polymerase II transcription subunit 16	719.55	765.16	667.53	707.33	591.42	567.88	-0.21
KLMA_50627	YTP1	protein YTP1	1385.01	1185.77	1386.23	944.51	946.10	867.81	-0.52
KLMA_50628	KEX1	KEX1 protease	1676.59	1635.47	1832.06	1242.24	1193.79	1198.86	-0.50
KLMA_50630	NAR1	cytosolic Fe-S cluster assembly factor NAR1	1026.41	1012.38	920.90	1827.62	1120.49	1216.15	0.49
KLMA_50631	ZWF	glucose-6-phosphate 1-dehydrogenase	2564.26	2767.54	2492.29	2357.49	2800.39	2655.29	0.00
KLMA_50632	LRG1	rho-GTPase-activating protein LRG1	674.87	840.11	775.95	941.99	1025.29	838.42	0.29
KLMA_50633	ATG2	autophagy-related protein 2	681.92	1010.14	740.62	1285.98	832.37	946.47	0.33
KLMA_50634		glutathione S-transferase 1	407.98	432.92	354.47	357.45	403.55	396.74	-0.05
KLMA_50635	BIO3	adenosylmethionine-8-amino-7-oxononanoate aminotransferase	42.33	61.53	67.00	43.74	18.53	42.35	-0.71
KLMA_50637	BIO5	7-keto 8-aminopelargonic acid transporter	92.88	111.87	120.59	121.11	61.50	72.61	-0.35
KLMA_50638	Nqo2	ribosyldihydronicoti namide dehydrogenase	49.38	29.08	43.85	48.78	42.97	57.05	0.28
KLMA_60001		DUP super family	122.28	99.56	148.61	192.60	129.74	143.48	0.33

Locus_tag	UniProt_gene	Product	Unique exon reads						log$_2$ Fold Change
			KmWT.1	KmWT.2	KmWT.3	Kmmig1.1	Kmmig1.2	Kmmig1.3	
KLMA_60002	PYD2	dihydropyrimidinase	96.41	92.85	76.74	217.83	112.89	149.53	0.85
KLMA_60003	yxeK	putative monooxygenase yxeK	205.75	202.48	194.90	863.77	517.28	522.07	1.66
KLMA_60004		arylsulfotrans	191.64	195.76	176.63	452.49	250.22	290.42	0.82
KLMA_60005		UNC93-like protein	78.77	49.22	85.27	235.50	144.91	133.97	1.27
KLMA_60006		uncharacterized sulfatase PB10D8.02c	325.68	297.56	264.33	447.44	255.27	337.96	0.23
KLMA_60007		flocculation protein FLO9	4206.76	5240.88	3363.25	473.52	686.62	725.19	-2.76
KLMA_60008	HUL5	probable E3 ubiquitin-protein ligase HUL5	340.96	370.27	263.12	716.58	473.47	481.44	0.78
KLMA_60009	GPI10	GPI mannosyltransferase 3	880.62	703.63	860.00	1060.57	886.29	909.30	0.23
KLMA_60010	MSS2	protein MSS2	182.24	259.53	221.70	322.13	320.14	336.23	0.56
KLMA_60011		L-2-hydroxyglutarate dehydrogenase	270.42	351.26	303.31	579.49	459.99	507.38	0.74
KLMA_60012	KIN28	serine/threonine-protein kinase KIN28	211.63	274.07	211.95	875.54	599.84	742.48	1.67
KLMA_60013	MRF1	peptide chain release factor 1	156.37	144.31	142.52	609.77	411.13	427.86	1.71
KLMA_60014	ROG1	putative lipase ROG1	316.27	252.82	215.61	486.13	371.53	454.65	0.74
KLMA_60015	TIP20	protein transport protein TIP20	384.46	513.46	472.63	787.23	554.35	560.97	0.47
KLMA_60016	ASF1	histone chaperone ASF1	299.81	335.60	275.30	568.56	508.86	541.09	0.83
KLMA_60017		conserved hypothetical protein	225.74	314.34	205.86	197.65	181.13	224.73	-0.31
KLMA_60018	UTH1	protein UTH1	1212.18	2135.50	1176.71	797.32	476.00	507.38	-1.35
KLMA_60019	SHB17	uncharacterized protein YKR043C	736.01	542.55	607.85	201.85	319.30	234.24	-1.32
KLMA_60020	PHO86	inorganic phosphate transporter PHO86	1419.10	1061.60	1385.01	1036.18	1092.69	1120.20	-0.25
KLMA_60021	CPS1	carboxypeptidase S	109.34	125.29	141.30	149.71	87.62	137.43	0.00
KLMA_60022	PET10	protein PET10	4276.12	3467.82	4312.17	4210.34	2554.39	3332.08	-0.26

Locus_tag	UniProt_gene	Product	Unique exon reads						log_2 Fold Change
			KmWT.1	KmWT.2	KmWT.3	Kmmig1.1	Kmmig1.2	Kmmig1.3	
KLMA_60023	NAP1	nucleosome assembly protein	1039.34	1164.52	920.90	1201.03	1106.17	1245.53	0.18
KLMA_60024	RPE1	ribulose-phosphate 3-epimerase	1353.26	1035.87	1219.34	2333.10	1798.69	1903.31	0.74
KLMA_60025	ALB1	ribosome biogenesis protein ALB1	479.70	515.70	488.47	222.88	244.32	243.75	-1.06
KLMA_60026	MTC1	UPF0615 protein YJL123C	887.68	896.04	831.98	1246.45	1084.27	1230.84	0.45
KLMA_60027	FMP46	putative redox protein FMP46	116.40	99.56	90.14	351.56	284.76	344.88	1.68
KLMA_60028	LSM1	sm-like protein LSm1	525.55	472.07	470.20	510.52	589.73	471.94	0.10
KLMA_60029	ACAD11	acyl-CoA dehydrogenase family member 11	15.28	45.86	14.62	414.64	149.96	172.87	3.28
KLMA_60030		hypothetical protein	323.33	564.92	427.56	1407.93	920.83	1011.29	1.34
KLMA_60031	TRM61	tRNA (adenine-N(1)-)-methyltransferase catalytic subunit TRM61	420.91	394.88	432.43	367.54	293.18	335.37	-0.33
KLMA_60032	NIT2	probable hydrolase NIT2	282.17	345.66	367.87	917.59	647.02	693.21	1.18
KLMA_60033	SPT10	protein SPT10	85.83	168.92	97.45	191.76	188.71	192.75	0.70
KLMA_60034		PNGaseA super family	1764.77	1741.74	1635.94	1205.24	1151.67	1057.97	-0.59
KLMA_60035	PBS2	serine/threonine-protein kinase ste20	1062.86	1141.02	1013.48	984.88	984.86	981.04	-0.13
KLMA_60036	TRK1	low-affinity potassium transport protein	591.39	620.85	708.95	472.67	492.85	462.43	-0.43
KLMA_60037	URA2	protein URA1	21507.60	11339.76	17467.93	3798.22	7050.69	6353.86	-1.55
KLMA_60038	AIM23	uncharacterized protein YJL131C	203.40	221.49	219.26	250.64	282.23	203.99	0.19
KLMA_60039		uncharacterized protein YJL132W	185.77	304.27	244.84	1076.56	979.80	1077.85	2.09
KLMA_60040		transmembrane protein 184 homolog YKR051W	246.90	267.36	232.66	340.63	310.03	320.68	0.38
KLMA_60041	MRS4	mitochondrial RNA-splicing protein MRS4	1401.47	1180.18	1219.34	773.77	704.31	716.55	-0.79
KLMA_60042	PEX21	peroxisomal	666.64	781.94	715.04	432.30	247.69	235.97	-1.24

Locus_tag	UniProt_gene	Product	Unique exon reads						log2 Fold Change
			KmWT.1	KmWT.2	KmWT.3	Kmmig1.1	Kmmig1.2	Kmmig1.3	
		membrane protein PEX21							
KLMA_60043	TDA11	hypothetical protein	67.02	83.90	62.12	79.90	85.93	93.35	0.28
KLMA_60044	KEL1	kelch repeat-containing protein 1	614.91	638.75	637.08	433.99	540.03	475.39	-0.38
KLMA_60045		conserved hypothetical membrane protein	4.70	10.07	2.44	16.82	5.90	1.73	0.50
KLMA_60046		uncharacterized protein YGR237C	1449.67	1610.86	1325.32	1336.44	821.42	804.71	-0.57
KLMA_60047	MOS2	uncharacterized protein YGR235C	596.09	497.80	565.21	491.18	607.43	545.41	-0.01
KLMA_60048	RTT107	regulator of Ty1 transposition protein 107	558.47	574.99	573.74	341.47	314.24	293.88	-0.85
KLMA_60049		hypothetical protein	0.00	0.00	0.00	10.93	10.11	5.19	5.35
KLMA_60050	PHO81	phosphate system positive regulatory protein PHO81	640.77	500.04	500.65	747.70	625.12	742.48	0.37
KLMA_60051		probable 26S proteasome regulatory subunit p28	90.53	154.37	159.57	277.55	365.64	304.25	1.23
KLMA_60052	PHB2	prohibitin-2	1091.08	1237.23	1013.48	1102.63	1299.94	1172.06	0.10
KLMA_60053		spo12 super family protein	16.46	24.61	30.45	53.83	58.13	40.62	1.10
KLMA_60054		KNR4/SMI1 homolog	1939.95	1971.06	1851.55	1270.84	1545.95	1513.48	-0.41
KLMA_60055	ALG10	alpha-1,2 glucosyltransferase	634.89	439.63	531.10	757.79	755.70	778.78	0.51
KLMA_60056	MTC6	uncharacterized protein YHR151C	141.09	174.51	171.76	254.00	198.82	223.87	0.47
KLMA_60057	PEX28	peroxisomal membrane protein PEX28	186.94	277.43	216.83	502.11	371.53	390.69	0.89
KLMA_60058	AMA1	meiosis-specific APC/C activator protein AMA1	54.08	36.92	26.80	97.56	48.86	63.10	0.83
KLMA_60059	HSV2	SVP1-like protein 2	94.06	126.41	90.14	268.30	262.85	318.08	1.45
KLMA_60060	PET54	protein PET54	386.81	344.54	383.71	318.76	262.85	261.90	-0.40
KLMA_60061	SKG6	uncharacterized protein YHR149C	774.81	736.07	862.43	656.03	638.60	586.90	-0.33

Locus_tag	UniProt_gene	Product	Unique exon reads						log$_2$ Fold Change
			KmWT.1	KmWT.2	KmWT.3	Kmmig1.1	Kmmig1.2	Kmmig1.3	
KLMA_60062	IMP3	U3 small nucleolar ribonucleoprotein protein IMP3	248.08	290.85	244.84	116.91	135.64	127.06	-1.05
KLMA_60063	rplF	54S ribosomal protein L6	409.15	454.17	472.63	259.89	386.70	331.91	-0.45
KLMA_60064	rplC	54S ribosomal protein L9	712.49	776.34	677.28	355.77	608.27	464.16	-0.60
KLMA_60065	CRM1	exportin-1	2199.79	2480.05	2348.55	1722.49	1906.53	1876.51	-0.35
KLMA_60066	CCH1	calcium-channel protein CCH1	737.18	826.68	690.68	926.85	773.39	988.82	0.25
KLMA_60067	GPI1	phosphatidylinositol N-acetylglucosaminyltransferase subunit GPI1	357.42	312.10	255.81	449.13	484.42	555.78	0.69
KLMA_60068	RSM27	mitochondrial 37S ribosomal protein S27	137.56	147.66	115.72	68.97	159.23	102.86	-0.28
KLMA_60069		40S ribosomal protein S0	21537.00	14725.92	19094.13	5141.39	11064.25	9327.24	-1.12
KLMA_60070	FRE8	probable ferric reductase transmembrane component 8	261.01	333.36	300.88	444.92	222.41	293.02	0.10
KLMA_60071	ZPR1	zinc finger protein ZPR1	980.56	956.45	967.19	536.60	730.43	710.50	-0.55
KLMA_60072		uncharacterized GTP-binding protein YGR210C	213.98	178.98	199.77	145.50	183.66	191.89	-0.19
KLMA_60073	JEN1	carboxylic acid transporter protein homolog	542.01	704.75	451.92	1624.08	891.34	733.84	0.94
KLMA_60074	DIP5	dicarboxylic amino acid permease	172.83	82.78	179.06	287.64	513.07	433.91	1.51
KLMA_60075	PDC1	pyruvate decarboxylase	138566.66	87723.45	125704.54	17855.68	25414.17	25543.38	-2.35
KLMA_60076	STU2	protein STU2	349.19	354.61	306.97	220.36	280.54	281.78	-0.37
KLMA_60077	TRX2	thioredoxin-2	861.81	1101.87	796.65	1683.80	1486.13	1225.65	0.67
KLMA_60078	SER2	phosphoserine phosphatase	175.18	166.68	165.67	201.85	254.43	232.51	0.44
KLMA_60079	CIR1	probable electron transfer flavoprotein subunit beta	379.76	354.61	382.49	331.38	342.89	317.22	-0.17

Locus_tag	UniProt_gene	Product	Unique exon reads						$\log_2$ Fold Change
			KmWT.1	KmWT.2	KmWT.3	Kmmig1.1	Kmmig1.2	Kmmig1.3	
KLMA_60081		mvb12 super family protein	39.97	50.34	41.42	42.05	37.07	35.44	-0.20
KLMA_60082	MDE1	putative aldolase class 2 protein YJR024C	357.42	269.59	341.08	304.46	205.56	274.00	-0.30
KLMA_60083		sm_like super family protein	101.11	105.15	114.50	82.42	64.03	66.56	-0.59
KLMA_60084		mer2 super family protein	0.00	2.24	2.44	1.68	1.68	0.00	-0.47
KLMA_60085	TDA10	uncharacterized kinase YGR205W	116.40	93.97	103.54	189.24	283.07	314.62	1.33
KLMA_60086	TES1	peroxisomal acyl-coenzyme A thioester hydrolase 1	199.87	206.95	175.41	412.96	600.69	617.15	1.49
KLMA_60087	ADE3	c-1-tetrahydrofolate synthase	8558.13	6584.38	8112.72	2005.08	3120.54	2759.88	-1.56
KLMA_60088	ESS1	peptidyl-prolyl cis-trans isomerase ESS1	357.42	351.26	302.10	448.28	325.20	391.55	0.20
KLMA_60089	YCH1	uncharacterized protein YGR203W	132.86	87.25	119.38	716.58	540.03	680.25	2.51
KLMA_60090	PCT1	choline-phosphate cytidylyltransferase	1955.24	1242.82	1628.63	777.14	889.66	877.32	-0.92
KLMA_60091	GEP4	UPF0660 protein YHR100C	117.57	58.17	62.12	75.70	48.86	44.08	-0.50
KLMA_60092		LIM domain and RING finger protein YDR266C	679.57	586.17	679.71	334.74	349.63	359.57	-0.90
KLMA_60093	PEX10	peroxisome biogenesis factor 10	97.59	148.78	97.45	217.83	174.39	199.67	0.78
KLMA_60094	SHE4	SWI5-dependent HO expression protein 4	198.70	237.15	207.08	370.91	351.31	397.60	0.80
KLMA_60095	AKR1	palmitoyltransferase AKR1	520.85	505.63	470.20	709.01	721.16	852.25	0.61
KLMA_60096	EXO1	exodeoxyribonuclease 1	422.09	361.32	422.69	132.89	154.17	216.09	-1.26
KLMA_60097	HMS1	probable transcription factor HMS1	5089.73	5881.86	5144.15	2247.31	1947.81	2018.27	-1.38
KLMA_60098		FAS1 domain-containing protein KLLA0E16841g	1895.27	1877.10	1835.72	2493.74	1678.21	1835.02	0.10
KLMA_60099	DFG16	protein DFG16	625.49	656.65	595.66	809.94	621.75	658.64	0.15
KLMA_60100		hypothetical protein	47.03	60.41	43.85	78.22	44.65	42.35	0.13

Locus_tag	UniProt_gene	Product	Unique exon reads						log$_2$ Fold Change
			KmWT.1	KmWT.2	KmWT.3	Kmmig1.1	Kmmig1.2	Kmmig1.3	
KLMA_60101	HSP78	heat shock protein 78	476.17	890.45	557.90	1766.22	1395.14	1236.89	1.19
KLMA_60102	YSP2	GRAM domain-containing protein YSP2	2673.61	3091.95	3254.83	1567.73	1568.69	1697.59	-0.90
KLMA_60103	SKP1	suppressor of kinetochore protein 1	563.17	550.38	495.78	549.21	673.98	567.88	0.15
KLMA_60104	PEX3	peroxisomal biogenesis factor 3	293.93	398.24	266.77	501.27	455.78	464.16	0.57
KLMA_60105		hypothetical protein	14.11	31.32	24.36	108.50	72.45	53.59	1.75
KLMA_60106	LRP1	exosome complex protein LRP1	179.89	164.44	194.90	317.92	226.63	206.58	0.48
KLMA_60107	UBX5	UBX domain-containing protein 5	400.92	493.32	443.40	788.91	681.56	681.97	0.69
KLMA_60108	GPI8	GPI-anchor transamidase	577.28	501.16	583.48	395.30	566.99	548.86	-0.14
KLMA_60109	KSP1	serine/threonine-protein kinase KSP1	1266.26	1514.65	1327.76	973.10	1018.55	1038.09	-0.44
KLMA_60110	IRC3	putative ATP-dependent helicase IRC3	201.05	220.37	203.43	271.66	191.24	232.51	0.15
KLMA_60111	SAM35	sorting assembly machinery 35 kDa subunit	198.70	229.32	172.97	152.23	139.85	148.67	-0.45
KLMA_60112	STE12	protein STE12	229.27	297.56	242.41	352.40	306.66	382.04	0.44
KLMA_60113	GPI18	GPI mannosyltransferase 2	312.74	305.39	369.09	312.87	351.31	326.73	0.01
KLMA_60114	COQ1	hexaprenyl pyrophosphate synthetase	562.00	686.85	511.61	470.15	615.85	554.05	-0.10
KLMA_60115	RER2	dehydrodolichyl diphosphate synthetase	241.02	257.29	310.62	260.73	277.18	273.14	0.00
KLMA_60116	YRB1	ran-specific GTPase-activating protein 1	1393.24	1290.92	1218.13	960.49	1318.48	1114.15	-0.20
KLMA_60117	NTH1	neutral trehalase	2547.80	2162.35	2275.46	2808.29	1735.50	2008.76	-0.09
KLMA_60118	HTB1	histone H2B.2	10534.53	7735.47	11167.78	3344.05	6993.40	5320.96	-0.91
KLMA_60119	HTA1	histone H2A	8234.80	7148.18	8589.01	3424.79	6197.26	4686.53	-0.74
KLMA_60120	RMD1	sporulation protein RMD1	171.66	223.73	182.72	448.28	405.23	437.36	1.16

Locus_tag	UniProt_gene	Product	Unique exon reads						log2 Fold Change
			KmWT.1	KmWT.2	KmWT.3	Kmmig1.1	Kmmig1.2	Kmmig1.3	
KLMA_60121	NHP10	HMG-box containing protein	248.08	259.53	275.30	363.34	371.53	341.42	0.46
KLMA_60122	UTP20	U3 small nucleolar RNA-associated protein 20	2156.29	1644.42	2045.23	1481.95	940.20	1229.98	-0.68
KLMA_60123		RCR super family protein	241.02	206.95	252.15	941.14	461.68	516.02	1.45
KLMA_60124		RCR super family protein	18.81	23.49	20.71	341.47	110.36	215.22	3.40
KLMA_60125	gabD	succinate-semialdehyde dehydrogenase [NADP+]	59.96	132.00	74.31	2021.06	892.18	876.45	3.83
KLMA_60126	RAD57	DNA repair protein RAD57	68.19	95.09	48.73	121.11	73.30	121.87	0.57
KLMA_60127	MAF1	repressor of RNA polymerase III transcription MAF1	1392.06	1204.79	1338.72	901.61	925.04	895.47	-0.53
KLMA_60128	HHF1	histone H4	5119.12	6050.78	5353.67	4507.23	4882.16	4241.38	-0.28
KLMA_60129	HHT1	histone H3	29004.05	30223.70	30818.60	18269.48	18624.64	17157.42	-0.74
KLMA_60130	TRP1	N-(5'-phosphoribosyl)anthranilate isomerase	478.52	364.68	472.63	371.75	270.44	348.33	-0.41
KLMA_60131	IPP1	inorganic pyrophosphatase	13313.95	12505.39	12951.12	3858.78	5445.77	5517.17	-1.39
KLMA_60132	APC11	anaphase-promoting complex subunit 11	32.92	30.20	29.24	101.77	79.19	77.79	1.49
KLMA_60133	GRX7	monothiol glutaredoxin-7	259.84	294.21	281.39	488.66	391.75	521.21	0.75
KLMA_60134	MNN2	protein TTP1	1544.91	1378.18	1529.97	1370.93	1267.09	1302.58	-0.18
KLMA_60135	MDM20	N-terminal acetyltransferase B complex subunit MDM20	1704.81	1402.79	1767.50	525.66	589.73	622.33	-1.49
KLMA_60136	BRX1	ribosome biogenesis protein BRX1	830.06	768.51	844.16	354.09	411.13	438.23	-1.02
KLMA_60137		ATP synthase subunit K	199.87	236.04	213.17	145.50	282.23	224.73	0.01
KLMA_60138		putative serine carboxypeptidase YBR139W	679.57	792.00	685.81	873.02	1104.49	1098.59	0.51
KLMA_60139	AVO1	target of rapamycin complex 2 subunit	736.01	870.31	741.84	632.48	763.28	654.32	-0.20

Locus_tag	UniProt_gene	Product	Unique exon reads						log2 Fold Change
			KmWT.1	KmWT.2	KmWT.3	Kmmig1.1	Kmmig1.2	Kmmig1.3	
		AVO1							
KLMA_60140	REX4	RNA exonuclease 4	225.74	140.95	211.95	121.11	108.68	90.76	-0.85
KLMA_60141	IRA2	inhibitory regulator protein IRA2	1804.74	2005.74	1958.75	2063.12	1994.99	2230.03	0.12
KLMA_60142	EOS1	N-glycosylation protein EOS1	412.68	261.76	336.20	428.10	304.98	374.27	0.13
KLMA_60143	TPM2	tropomyosin-2	1367.37	1384.89	1402.06	1191.78	1888.83	1745.13	0.22
KLMA_60144	MGA2	protein MGA2	1608.40	1743.98	1718.78	772.09	847.53	1039.82	-0.93
KLMA_60145	RAM2	protein farnesyltransferase/geranylgeranyltransferase type-1 subunit alpha	71.72	153.26	112.07	183.35	172.71	198.80	0.72
KLMA_60146	YVH1	tyrosine-protein phosphatase YVH1	328.03	308.75	324.02	215.31	146.59	158.18	-0.89
KLMA_60147		apc15p super family protein	305.69	285.26	226.57	527.34	414.50	471.94	0.79
KLMA_60148		DUF1748 super family protein	145.79	123.05	131.56	221.20	182.82	177.19	0.54
KLMA_60149	SWD2	COMPASS component SWD2	550.24	426.21	497.00	746.02	671.45	639.62	0.48
KLMA_60150	HCS1	DNA polymerase alpha-associated DNA helicase A	259.84	363.56	334.98	495.38	509.70	493.55	0.64
KLMA_60151	ATP7	ATP synthase subunit d	3308.50	3532.70	3485.06	2042.93	3032.92	2357.96	-0.47
KLMA_60152	PUT3	proline utilization trans-activator	871.21	965.40	1036.63	1059.73	1037.93	1032.90	0.12
KLMA_60153	URB1	nucleolar pre-ribosomal-associated protein 1	1588.41	1351.33	1354.56	682.10	665.56	701.86	-1.07
KLMA_60154		uncharacterized protein YIR024C	172.83	237.15	166.88	191.76	214.83	167.68	-0.01
KLMA_60155	DAL81	transcriptional activator protein DAL81	954.69	978.82	1097.53	654.34	568.67	607.64	-0.73
KLMA_60156	ARC19	actin-related protein 2/3 complex subunit 4	319.80	353.49	252.15	402.03	468.42	356.98	0.41
KLMA_60157	PRP40	pre-mRNA-processing protein PRP40	357.42	360.21	303.31	286.80	293.18	319.81	-0.18

Locus_tag	UniProt_gene	Product	Unique exon reads						log2 Fold Change
			KmWT.1	KmWT.2	KmWT.3	Kmmig1.1	Kmmig1.2	Kmmig1.3	
KLMA_60158	SEC11	signal peptidase complex catalytic subunit SEC11	450.30	359.09	386.15	285.96	579.62	450.33	0.14
KLMA_60159		UPF0659 protein YMR090W	808.90	946.38	857.56	1176.64	1256.13	1373.46	0.54
KLMA_60160	CCE1	cruciform cutting endonuclease 1	39.97	70.47	49.94	78.22	46.34	70.01	0.28
KLMA_60161		hypothetical protein	0.00	0.00	0.00	20.19	12.64	19.02	6.33
KLMA_60162	CAB3	flavoprotein super family protein	416.21	459.77	360.57	224.56	254.43	226.46	-0.81
KLMA_60163	TIM10	mitochondrial import inner membrane translocase subunit TIM10	156.37	152.14	149.83	140.46	194.61	168.55	0.14
KLMA_60164	CYT2	cytochrome c1 heme lyase	502.04	418.38	531.10	309.51	304.13	219.55	-0.80
KLMA_60165	GPA1	guanine nucleotide-binding protein alpha-1 subunit	4.70	2.24	4.87	106.81	61.50	84.71	4.43
KLMA_60166	SRX1	sulfiredoxin	21.16	13.42	20.71	37.01	20.22	26.79	0.61
KLMA_60167	MDH1	malate dehydrogenase	10668.56	9357.51	10172.57	2217.87	3252.80	3263.80	-1.79
KLMA_60168	HOT13	helper of Tim protein 13	14.11	14.54	12.18	16.82	17.69	18.15	0.37
KLMA_60169	AVO2	hypothetical protein	155.20	156.61	182.72	226.24	170.18	189.29	0.24
KLMA_60170		UBX domain-containing protein 4	72.90	106.27	71.87	122.79	132.27	103.72	0.51
KLMA_60171	SOV1	protein SOV1	236.32	237.15	208.30	301.94	300.76	299.93	0.40
KLMA_60172	RRP14	ribosomal RNA-processing protein 14	630.19	590.65	689.46	305.30	358.05	356.98	-0.90
KLMA_60173	TEF4	eukaryotic elongation factor 1Bgamma	5335.46	4612.20	5452.33	1645.11	3097.79	2646.65	-1.06
KLMA_60175	VMA5	v-type proton ATPase subunit C	969.98	827.80	1014.70	639.20	898.08	966.35	-0.17
KLMA_60176	JLP1	alpha-ketoglutarate-dependent sulfonate dioxygenase	89.36	67.12	68.22	464.26	219.89	340.56	2.19
KLMA_60177	KAR5	nuclear fusion protein KAR5	47.03	39.15	80.40	102.61	67.40	75.20	0.56
KLMA_60178	AEP1	nuclear control of ATPase messenger	225.74	304.27	239.97	148.87	175.24	189.29	-0.59

Locus_tag	UniProt_gene	Product	Unique exon reads						log2 Fold Change
			KmWT.1	KmWT.2	KmWT.3	Kmmig1.1	Kmmig1.2	Kmmig1.3	
		RNA expression protein							
KLMA_60179	DHR2	probable ATP-dependent RNA helicase DHR2	282.17	205.83	260.68	121.95	93.51	108.91	-1.21
KLMA_60180	STL1	sugar transporter STL1	85.83	183.46	52.38	767.89	250.22	382.04	2.12
KLMA_60181		arginine biosynthesis bifunctional protein ARG7	1218.05	732.72	1125.55	1322.14	1361.44	1488.42	0.44
KLMA_60182	RNA14	mRNA 3'-end-processing protein RNA14	550.24	547.02	545.72	423.89	563.62	484.90	-0.16
KLMA_60183	SAM37	sorting assembly machinery 37 kDa subunit	197.52	185.70	214.39	266.62	210.62	254.12	0.29
KLMA_60184	SEN15	tRNA-splicing endonuclease subunit SEN15	35.27	26.85	26.80	45.42	34.54	60.50	0.66
KLMA_60185		uncharacterized protein YKL077W	585.51	621.97	628.55	502.11	550.98	565.29	-0.18
KLMA_60186		uncharacterized protein YKL075C	195.17	184.58	259.46	265.77	202.19	254.12	0.18
KLMA_60187	FET3	iron transport multicopper oxidase FET3	22949.05	16695.86	20594.86	8625.06	5826.57	3139.33	-1.78
KLMA_60189	BUB2	mitotic check point protein BUB2	28.22	22.37	43.85	44.58	67.40	70.88	0.96
KLMA_60190		splicing factor MUD2	177.54	172.27	187.59	145.50	100.25	143.48	-0.47
KLMA_60191	LHS1	heat shock protein 70 homolog LHS1	404.45	515.70	389.80	508.84	477.68	471.07	0.15
KLMA_60192	STV1	v-type proton ATPase subunit a	914.72	984.41	924.56	1079.92	1136.50	1209.23	0.28
KLMA_60193	STB2	protein STB2	221.04	285.26	190.03	367.54	262.85	375.13	0.53
KLMA_60194	FAR3	factor arrest protein 3	115.22	155.49	107.20	145.50	140.69	126.20	0.12
KLMA_60195		tRNA_bindingDomain super family protein	34.10	20.14	15.84	40.37	56.45	66.56	1.22
KLMA_60196	RSR1	ras-related protein RSR1	601.97	308.75	476.29	1107.67	713.58	898.93	0.97
KLMA_60197	RRP1	ribosomal RNA-processing protein 1	297.46	266.24	333.77	203.54	161.76	167.68	-0.75

Locus_tag	UniProt_gene	Product	Unique exon reads						log$_2$ Fold Change
			KmWT.1	KmWT.2	KmWT.3	Kmmig1.1	Kmmig1.2	Kmmig1.3	
KLMA_60198		protein transport protein SSS1	145.79	112.98	144.96	161.48	162.60	145.21	0.22
KLMA_60199	BUR6	H2A super family protein	440.90	353.49	557.90	1153.09	507.17	656.91	0.78
KLMA_60200		SSF super family protein	901.78	900.51	838.07	2169.09	909.03	1070.07	0.65
KLMA_60202		hypothetical protein	639.60	738.31	599.32	403.71	332.78	406.25	-0.79
KLMA_60203	TVP23	Golgi apparatus membrane protein TVP23	281.00	308.75	292.35	226.24	283.07	304.25	-0.12
KLMA_60204	RRP8	ribosomal RNA-processing protein 8	832.42	645.46	727.22	447.44	375.74	477.12	-0.76
KLMA_60205		conserved hypothetical phospatase and actin regulator 2	61.14	88.37	80.40	119.43	78.35	92.49	0.34
KLMA_60206		probable 5-hydroxyisourate hydrolase	85.83	59.29	67.00	197.65	165.13	149.53	1.27
KLMA_60207	IOC3	ISWI one complex protein 3	293.93	308.75	336.20	227.93	239.26	301.66	-0.29
KLMA_60208	VCX1	vacuolar calcium ion transporter	82.30	31.32	41.42	463.42	203.04	281.78	2.61
KLMA_60209	RPA190	DNA-directed RNA polymerase I subunit RPA1	4587.69	3921.99	4717.80	1456.71	1605.76	1629.31	-1.50
KLMA_60210	FUN12	eukaryotic translation initiation factor 5B	3948.10	4330.30	4102.65	1620.72	2157.58	2128.04	-1.07
KLMA_60212	RBG1	GTP-binding protein RBG1	908.84	1091.80	931.87	545.85	655.45	718.28	-0.61
KLMA_60214		uncharacterized protein YOR342C	3056.89	2484.52	2963.70	288.48	308.35	251.53	-3.33
KLMA_60215		hypothetical protein	2.35	4.47	3.65	12.62	8.42	15.56	1.80
KLMA_60217	TYE7	hypothetical protein	2296.20	1934.15	1940.48	462.58	508.86	467.62	-2.10
KLMA_60218	REV1	DNA repair protein REV1	1332.10	1353.57	1411.81	483.61	353.00	346.61	-1.79
KLMA_60219	PYK1	pyruvate kinase	130695.16	85223.27	121539.77	8930.36	14463.65	14939.49	-3.14
KLMA_60221	PUT4	proline-specific permease	452.66	356.85	404.42	2408.79	1600.71	1213.55	2.11
KLMA_60222		uncharacterized aminotransferase C660.12c	897.08	545.90	970.85	925.16	981.49	1109.83	0.32

Locus_tag	UniProt_gene	Product	Unique exon reads						log$_2$ Fold Change
			KmWT.1	KmWT.2	KmWT.3	Kmmig1.1	Kmmig1.2	Kmmig1.3	
KLMA_60223	CIN1	chromosome instability protein 1	418.56	356.85	412.94	370.07	283.91	319.81	-0.29
KLMA_60225	MEK1	meiosis-specific serine/threonine-protein kinase MEK1	68.19	76.07	54.82	95.04	35.38	41.49	-0.21
KLMA_60226	LEU4	2-isopropylmalate synthase	5018.01	3109.85	4412.05	1481.95	1615.87	1905.90	-1.33
KLMA_60227	INP53	inositol polyphosphate 5-phosphatase	918.24	935.19	891.67	594.63	662.19	731.24	-0.47
KLMA_60229	YAF9	protein AF-9 homolog	912.37	753.97	891.67	342.31	336.99	362.16	-1.30
KLMA_60230	TFC7	transcription factor tau 55 kDa subunit	5095.61	3930.94	5392.65	1159.82	1026.14	1113.29	-2.13
KLMA_60231		maf-like protein YOR111W	39.97	17.90	24.36	62.24	48.02	43.22	0.90
KLMA_60232	NOP15	ribosome biogenesis protein 15	831.24	733.83	791.78	306.15	360.58	344.01	-1.22
KLMA_60233	CYB5	cytochrome b5	2576.02	1793.20	3064.81	722.47	1293.20	1102.92	-1.25
KLMA_60234	CEX1	uncharacterized protein YOR112W	839.47	657.77	926.99	278.39	432.19	352.66	-1.19
KLMA_60235	DBP2	ATP-dependent RNA helicase DBP2	6137.30	4630.09	6688.73	3717.48	2502.16	2695.05	-0.97
KLMA_60236	RPC19	DNA-directed RNA polymerases I and III subunit RPAC2	529.08	617.50	515.27	267.46	288.13	355.25	-0.87
KLMA_60237	AZF1	zf-C2H2 super family protein	2879.36	2637.78	2729.82	1365.88	1182.84	1174.66	-1.15
KLMA_60238		uncharacterized protein YOR114W	112.87	184.58	127.90	93.36	107.84	82.98	-0.58
KLMA_60239	TRS33	transport protein particle 33 kDa subunit	168.13	167.80	121.81	231.29	269.59	281.78	0.77
KLMA_60240	BZZ1	protein BZZ1	487.93	566.04	595.66	660.23	708.52	733.84	0.35
KLMA_60241		uncharacterized vacuolar membrane protein SCY_4679	477.35	722.65	527.45	1538.30	1273.83	1486.69	1.31
KLMA_60242	DMA2	E3 ubiquitin-protein ligase DMA2	1161.62	1003.43	1147.47	572.76	686.62	625.79	-0.81
KLMA_60243	MLS1	malate synthase 1	143.44	224.85	120.59	278.39	178.61	216.09	0.46
KLMA_60244	RTM1	protein RTM1	1447.32	1463.19	1321.67	4093.43	2100.30	1994.06	0.95

Locus_tag	UniProt_gene	Product	Unique exon reads						log_2 Fold Change
			KmWT.1	KmWT.2	KmWT.3	Kmmig1.1	Kmmig1.2	Kmmig1.3	
KLMA_60245	DCP2	mRNA-decapping enzyme subunit 2	1072.26	1313.30	1210.82	786.39	850.06	859.17	-0.53
KLMA_60246	NCS2	cytoplasmic tRNA 2-thiolation protein 2	242.20	229.32	254.59	269.14	196.30	235.10	-0.05
KLMA_60247	COX23	cytochrome c oxidase-assembly factor COX23	48.20	74.95	48.73	36.17	36.23	32.85	-0.71
KLMA_60248	TOM70	mitochondrial import receptor subunit TOM70	2265.63	2934.22	2477.67	1269.16	1395.98	1376.05	-0.93
KLMA_60249		putative mitochondrial ribosomal protein YNL122C	50.56	24.61	42.63	32.80	37.91	35.44	-0.15
KLMA_60250	NMA111	pro-apoptotic serine protease NMA111	1272.14	1038.11	1184.02	687.14	780.98	772.73	-0.64
KLMA_60251	ORC6	origin recognition complex subunit 6	38.80	52.58	67.00	48.78	37.91	51.86	-0.19
KLMA_60252	SET1	histone-lysine N-methyltransferase	409.15	543.66	400.76	550.89	374.90	452.92	0.03
KLMA_60253	MSH1	DNA mismatch repair protein MSH1	703.09	672.31	674.84	1473.53	756.54	873.86	0.60
KLMA_60254	NAF1	H/ACA ribonucleoprotein complex non-core subunit NAF1	311.57	354.61	319.15	290.17	182.82	203.99	-0.54
KLMA_60256	ESBP6	uncharacterized transporter ESBP6	1405.00	1378.18	1393.54	1963.03	1183.68	1224.79	0.07
KLMA_60257	SPC98	spindle pole body component SPC98	415.03	408.31	432.43	434.83	471.79	436.50	0.10
KLMA_60258	FAR11	factor arrest protein 11	502.04	532.48	534.76	398.66	425.45	367.35	-0.40
KLMA_60259	LSM12	protein LSM12	362.12	266.24	344.73	340.63	378.27	316.35	0.09
KLMA_60260	TEP1	probable phosphatidylinositol-3	69.37	61.53	108.41	169.05	105.31	129.65	0.76
KLMA_60261	NRK1	nicotinamide riboside kinase	87.00	96.20	118.16	133.73	98.57	96.81	0.13
KLMA_60262		UPF0195 protein YHR122W	462.06	425.09	484.81	375.11	401.02	363.89	-0.27
KLMA_60263	CPT1	cholinephosphotransferase 1	1617.80	1422.92	1623.76	2839.41	2101.14	1975.91	0.57
KLMA_60264		mitochondrial import receptor subunit	755.99	671.19	774.73	470.15	845.00	758.04	-0.09

Locus_tag	UniProt_gene	Product	Unique exon reads						log2 Fold Change
			KmWT.1	KmWT.2	KmWT.3	Kmmig1.1	Kmmig1.2	Kmmig1.3	
		TOM22							
KLMA_60265	KRE33	UPF0202 protein YNL132W	1791.81	1567.23	1765.06	622.38	674.82	687.16	-1.37
KLMA_60266	NDT80	NDT80_PhoG super family protein	4.70	12.31	17.05	74.85	19.38	22.47	1.78
KLMA_60267	FYV6	protein FYV6	146.97	117.46	127.90	181.67	146.59	132.25	0.23
KLMA_60268		uncharacterized protein YNL134C	746.59	977.70	649.26	6434.10	3873.71	3836.00	2.57
KLMA_60269		lysine-rich arabinogalactan protein 19	225.74	130.88	209.52	254.00	276.33	452.92	0.80
KLMA_60270		hypothetical protein	45.85	35.80	36.54	26.91	40.44	18.15	-0.47
KLMA_60271	ETR1	probable trans-2-enoyl-CoA reductase	490.28	966.51	538.41	1880.61	1785.21	1869.60	1.47
KLMA_60272	MNN2	protein TTP1	2584.25	2398.39	2391.18	1854.53	1945.28	1997.52	-0.35
KLMA_60273		hypothetical protein	18.81	19.02	28.02	66.44	46.34	38.90	1.21
KLMA_60274	YPK3	probable serine/threonine-protein kinase YBR028C	467.94	345.66	443.40	338.11	324.35	323.27	-0.35
KLMA_60275	CDS1	phosphatidate cytidylyltransferase	4861.64	2157.88	3982.05	1646.79	2813.03	2408.09	-0.68
KLMA_60276	PRP9	pre-mRNA-splicing factor PRP9	332.73	317.70	338.64	309.51	275.49	231.65	-0.28
KLMA_60277	DBP10	ATP-dependent RNA helicase DBP10	1165.15	988.89	1179.15	589.58	599.84	530.71	-0.95
KLMA_60278	SLM3	mitochondrial tRNA-specific 2-thiouridylase 1	110.52	134.24	162.01	96.72	129.74	108.91	-0.28
KLMA_60279	MET22	3'(2')5'-bisphosphate nucleotidase	1459.08	1117.53	1290.00	839.38	1202.21	1343.21	-0.19
KLMA_60280	GPR1	G protein-coupled receptor GPR1	974.68	1033.63	1008.61	709.01	716.95	890.28	-0.38
KLMA_60281	INP54	inositol-1	393.87	334.48	364.22	365.02	281.39	291.29	-0.22
KLMA_60282	RIB2	bifunctional protein RIB2	322.15	391.53	384.93	442.40	362.27	359.57	0.08
KLMA_60283	RNT1	ribonuclease 3	873.57	653.29	836.85	672.85	710.21	651.72	-0.22
KLMA_60284	ISW2	ISWI chromatin-remodeling complex ATPase ISW2	584.34	675.66	679.71	904.14	631.86	672.47	0.19

Locus_tag	UniProt_gene	Product	Unique exon reads						log2 Fold Change
			KmWT.1	KmWT.2	KmWT.3	Kmmig1.1	Kmmig1.2	Kmmig1.3	
KLMA_60285		ATS1	845.35	825.56	836.85	2025.27	1137.34	1201.45	0.80
KLMA_60286	CPA1	carbamoyl-phosphate synthase arginine-specific small chain	10609.77	5708.47	9072.60	8443.39	7912.55	7564.82	-0.09
KLMA_60287	RAX1	bud site selection protein RAX1	494.98	586.17	527.45	278.39	346.26	373.40	-0.69
KLMA_60288	DFG5	mannan endo-1	912.37	1110.82	699.20	1209.44	1095.22	1106.37	0.32
KLMA_60289	BUD7	protein BCH1	901.78	929.60	923.34	921.80	1027.82	1054.51	0.12
KLMA_60290	TAF9	transcription initiation factor TFIID subunit 9	212.81	196.88	233.88	323.81	337.83	295.61	0.57
KLMA_60291	MBF1	multiprotein-bridging factor 1	813.60	1033.63	845.38	726.67	799.51	802.12	-0.21
KLMA_60292	RNA1	ran GTPase-activating protein 1	1132.23	1113.06	1042.72	811.62	889.66	791.75	-0.40
KLMA_60293		LPLAT super family protein	27.04	26.85	24.36	137.09	60.66	74.33	1.80
KLMA_60294	TIM18	succinate dehydrogenase [ubiquinone] cytochrome b small subunit	212.81	221.49	248.50	195.97	281.39	241.15	0.07
KLMA_60295	STE2	pheromone alpha factor receptor	89.36	91.73	80.40	148.87	123.84	126.20	0.61
KLMA_60296	BST1	GPI inositol-deacylase	502.04	560.44	535.98	264.93	335.31	357.84	-0.74
KLMA_60297	EPL1	enhancer of polycomb-like protein 1	1555.49	1877.10	1767.50	830.12	743.91	671.60	-1.21
KLMA_60298	BUD27	bud site selection protein 27	398.57	435.16	367.87	485.29	287.28	352.66	-0.09
KLMA_60299		GCR1_C super family protein	1261.56	1031.40	1313.14	827.60	818.89	985.36	-0.45
KLMA_60300	OSW2	outer spore wall protein 2	159.90	166.68	205.86	218.68	106.15	108.91	-0.30
KLMA_60301	FRS2	phenylalanyl-tRNA synthetase alpha chain	2270.33	1897.23	2009.91	1179.16	1798.69	1564.48	-0.44
KLMA_60302		ino eighty subunit 3	221.04	236.04	174.19	204.38	164.28	202.26	-0.15
KLMA_60303	FCF2	rRNA-processing protein FCF2	252.78	312.10	310.62	174.94	113.73	142.62	-1.02

Locus_tag	UniProt_gene	Product	Unique exon reads						log $_2$ Fold Change
			KmWT.1	KmWT.2	KmWT.3	Kmmig1.1	Kmmig1.2	Kmmig1.3	
KLMA_60304	CLU1	protein TIF31 homolog	2852.32	3322.39	2978.32	1195.14	2315.13	2048.52	-0.72
KLMA_60305	VPS24	vacuolar protein-sorting-associated protein 24	233.97	233.80	214.39	308.67	278.86	286.10	0.36
KLMA_60306		opi1 super family protein	519.67	341.19	478.72	319.60	302.45	370.81	-0.43
KLMA_60307		hypothetical protein	758.34	337.83	600.54	433.99	449.88	498.73	-0.29
KLMA_60308	APM2	AP-1 complex subunit mu-1	122.28	158.85	157.14	235.50	307.50	304.25	0.95
KLMA_60309		putative pterin-4-alpha-carbinolamine dehydratase	37.62	42.51	56.03	50.46	85.09	89.89	0.73
KLMA_60310	NFU1	nifU-like protein	364.48	420.61	348.38	755.27	631.86	686.30	0.87
KLMA_60311	PTM1	membrane protein PTM1	1634.26	1358.04	1405.72	1706.51	1815.54	1870.46	0.29
KLMA_60312	URC4	uracil catabolism protein 4	285.70	342.31	282.61	780.50	468.42	592.08	1.02
KLMA_60313		40S ribosomal protein S20	14983.48	12437.16	14554.17	3994.19	7656.43	5932.92	-1.26
KLMA_60314	engD	putative GTP-binding protein YLF2	67.02	111.87	93.80	116.91	122.16	122.74	0.41
KLMA_60315	OTU2	OTU domain-containing protein 2	276.30	323.29	309.40	216.99	309.19	272.27	-0.19
KLMA_60316	RGT1	uncharacterized transcriptional regulatory protein YKL038W	3870.50	4667.01	3975.96	1719.96	1483.60	1589.55	-1.38
KLMA_60317	UGP1	UTP--glucose-1-phosphate uridylyltransferase	7690.44	7329.40	7082.19	1693.89	2305.02	2228.30	-1.83
KLMA_60318	TUL1	transmembrane E3 ubiquitin-protein ligase 1	580.81	607.43	479.94	698.92	592.26	624.93	0.20
KLMA_60319		putative uncharacterized hydrolase YKL033W-A	362.12	440.75	420.25	400.34	345.42	363.03	-0.14
KLMA_60320	PRS3	ribose-phosphate pyrophosphokinase 3	2501.95	1861.43	2305.91	1243.93	1540.89	1483.23	-0.64
KLMA_60321	ETP1	RING finger protein ETP1	169.30	221.49	211.95	614.81	392.59	406.25	1.23

Locus_tag	UniProt_gene	Product	Unique exon reads						log2 Fold Change
			KmWT.1	KmWT.2	KmWT.3	Kmmig1.1	Kmmig1.2	Kmmig1.3	
KLMA_60322	TTI1	TEL2-interacting protein 1	142.26	130.88	146.18	253.16	167.65	167.68	0.49
KLMA_60323		uncharacterized transporter YHL008C	131.68	193.53	168.10	516.41	332.78	420.94	1.36
KLMA_60324	PDS5	sister chromatid cohesion protein PDS5	707.79	939.67	738.18	899.93	658.82	732.11	-0.06
KLMA_60325	RCO1	transcriptional regulatory protein RCO1	350.37	350.14	288.70	495.38	345.42	374.27	0.30
KLMA_60326		uncharacterized protein YMR074C	132.86	133.12	102.32	105.97	96.04	95.08	-0.31
KLMA_60327	AO-I	copper amine oxidase 1	166.95	144.31	158.36	497.07	374.06	607.64	1.66
KLMA_60328	IRC21	uncharacterized heme-binding protein YMR073C	27.04	27.97	25.58	43.74	34.54	31.12	0.44
KLMA_60329	IXR1	HMGB-UBF_HMG-box containing protein	1365.02	1143.26	1182.80	708.17	706.00	720.01	-0.79
KLMA_60330		hypothetical protein	311.57	249.46	372.75	232.13	250.22	285.24	-0.28
KLMA_60331	TVP18	Golgi apparatus membrane protein TVP18	565.53	515.70	588.35	650.98	577.94	579.12	0.12
KLMA_60332	MOT3	transcriptional activator/repressor MOT3	439.72	496.68	489.69	676.21	441.46	487.50	0.17
KLMA_60333		NAT_SF super family protein	49.38	50.34	47.51	61.40	63.19	61.37	0.34
KLMA_60334	NEM1	nuclear envelope morphology protein 1	259.84	302.04	320.37	242.22	237.58	259.31	-0.26
KLMA_60335	MAE1	NAD-dependent malic enzyme	835.94	625.33	934.30	825.92	994.12	983.63	0.23
KLMA_60336	TFA1	transcription initiation factor IIE subunit alpha	262.19	394.88	348.38	260.73	272.96	299.93	-0.27
KLMA_60337		uncharacterized protein YKL027W	732.48	693.56	817.36	403.71	473.47	385.50	-0.83
KLMA_60338	LEU5	mitochondrial carrier protein LEU5	387.99	384.82	383.71	762.84	707.68	722.60	0.92
KLMA_60339	CTP1	tricarboxylate transport protein	636.07	420.61	652.92	351.56	420.40	414.03	-0.53

Locus_tag	UniProt_gene	Product	Unique exon reads						log2 Fold Change
			KmWT.1	KmWT.2	KmWT.3	Kmmig1.1	Kmmig1.2	Kmmig1.3	
KLMA_60340	BSD2	metal homeostatis protein BSD2	455.01	432.92	398.33	882.27	766.65	835.83	0.95
KLMA_60341	SNF5	SWI/SNF chromatin-remodeling complex subunit SNF5	433.84	422.85	445.83	749.38	418.71	441.68	0.31
KLMA_60342	APM3	AP-3 complex subunit mu	304.51	415.02	297.22	295.21	349.63	375.13	0.00
KLMA_60343	CTI6	histone deacetylase complex subunit CTI6	533.78	567.16	444.62	415.48	540.87	497.00	-0.09
KLMA_60344	VPS45	vacuolar protein sorting-associated protein 45	718.37	760.68	605.41	878.07	915.77	785.70	0.31
KLMA_60345	TCO89	target of rapamycin complex 1 subunit TCO89	362.12	458.65	370.31	415.48	416.18	375.99	0.02
KLMA_60346	PAN2	PAB-dependent poly(A)-specific ribonuclease subunit PAN2	784.21	664.48	845.38	828.44	772.55	739.02	0.03
KLMA_60347	RTT10	uncharacterized WD repeat-containing protein YPL183C	295.11	332.24	352.04	502.11	399.33	372.54	0.38
KLMA_60348		54S ribosomal protein YPL183W-A	58.79	70.47	75.52	56.35	75.82	53.59	-0.14
KLMA_60349	SPC105	spindle pole body component SPC105	357.42	383.70	376.40	876.38	518.97	539.36	0.79
KLMA_60350	NUP145	nucleoporin NUP145	1627.21	1861.43	1896.62	991.61	1061.52	1066.61	-0.79
KLMA_60351	NBP35	cytosolic Fe-S cluster assembly factor NBP35	299.81	269.59	280.17	260.73	262.01	304.25	-0.04
KLMA_60352		hypothetical protein	2.35	2.24	0.00	5.89	1.68	10.37	1.95
KLMA_60354	MRN1	uncharacterized RNA-binding protein YPL184C	758.34	695.80	706.51	359.13	385.01	388.09	-0.93
KLMA_60355		ULP1-interacting protein 4	264.54	335.60	243.63	1567.73	874.49	899.79	1.99
KLMA_60356	MF(ALPHA)1	mating factor alpha-1	5.88	4.47	15.84	270.82	129.74	183.24	4.49
KLMA_60357	POS5	NADH kinase POS5	330.38	332.24	388.58	433.15	452.41	415.75	0.31
KLMA_60358	MMS2	ubiquitin-conjugating enzyme	406.80	482.14	430.00	419.69	438.93	411.43	-0.05

Locus_tag	UniProt_gene	Product	Unique exon reads						log$_2$ Fold Change
			KmWT.1	KmWT.2	KmWT.3	Kmmig1.1	Kmmig1.2	Kmmig1.3	
		variant MMS2							
KLMA_60359	MAD1	spindle assembly checkpoint component MAD1	371.53	384.82	410.51	507.16	334.46	335.37	0.01
KLMA_60360	LCL3	probable endonuclease YGL085W	236.32	191.29	207.08	232.97	228.31	161.63	-0.03
KLMA_60361	GUP1	glycerol uptake protein 1	2082.22	1958.76	1839.37	1562.69	1743.93	1738.22	-0.22
KLMA_60362	SCY1	protein kinase-like protein SCY1	298.64	337.83	326.46	1508.02	550.98	609.37	1.47
KLMA_60363		hypothetical protein	27.04	30.20	21.93	21.87	49.71	23.34	0.26
KLMA_60364	NAB3	nuclear polyadenylated RNA-binding protein 3	491.45	721.53	610.28	1172.44	750.65	802.98	0.58
KLMA_60365		uncharacterized protein YOR389W	277.47	401.60	249.72	698.08	447.36	464.16	0.79
KLMA_60366		uncharacterized protein YGL082W	851.23	910.58	750.37	2222.08	1438.95	1413.22	1.01
KLMA_60367		hypothetical membrane protein	306.87	498.92	296.00	1867.99	1290.68	1525.58	2.09
KLMA_60368		hypothetical protein	41.15	40.27	28.02	349.04	116.26	115.82	2.41
KLMA_60369	RSA1	ribosome assembly 1 protein	78.77	82.78	107.20	316.24	144.06	160.77	1.21
KLMA_60370	FMP37	UPF0041 protein FMP37	292.76	359.09	304.53	589.58	794.46	658.64	1.09
KLMA_60371	DDC1	DNA damage checkpoint protein 1	152.84	184.58	160.79	266.62	306.66	316.35	0.84
KLMA_60372	APL5	AP-3 complex subunit delta	743.06	854.65	721.13	844.42	793.61	758.04	0.05
KLMA_60373	KXD1	uncharacterized protein YGL079W	51.73	51.46	67.00	47.10	58.13	38.90	-0.24
KLMA_60374	OXR1	oxidation resistance protein 1	298.64	290.85	291.13	266.62	262.85	242.88	-0.19
KLMA_60375		delta6-FADS-like protein	543.19	503.39	500.65	761.16	466.73	584.30	0.23
KLMA_60376	SWI1	SWI/SNF chromatin-remodeling complex subunit SWI1	979.38	1049.29	1004.95	993.29	841.63	799.53	-0.20
KLMA_60377	RAD1	DNA repair protein RAD1	128.15	208.07	175.41	483.61	346.26	331.05	1.18

Locus_tag	UniProt_gene	Product	Unique exon reads						log2 Fold Change
			KmWT.1	KmWT.2	KmWT.3	Kmmig1.1	Kmmig1.2	Kmmig1.3	
KLMA_60378	MET12	methylenetetrahydro folate reductase 1	1055.80	897.16	1032.97	926.01	986.54	1083.04	0.00
KLMA_60379	RMI1	recQ-mediated genome instability protein 1	92.88	109.63	103.54	84.11	85.09	111.50	-0.13
KLMA_60380	TRS23	transport protein particle 23 kDa subunit	413.86	342.31	343.51	91.68	38.75	44.95	-2.65
KLMA_60381	SKS1	serine/threonine-protein kinase SKS1	11466.88	8433.51	9140.82	1397.00	1209.80	1158.23	-2.95
KLMA_60382		probable gluconokinase	68.19	67.12	58.47	93.36	139.01	133.97	0.92
KLMA_60383	ERG10	acetyl-CoA acetyltransferase	2947.55	2291.00	2822.40	2274.22	3262.07	3190.33	0.11
KLMA_60385	SUV3	ATP-dependent RNA helicase SUV3	152.84	167.80	140.08	345.68	250.22	288.69	0.94
KLMA_60386	TRM44	tRNA (uracil-O(2)-)-methyltransferase	246.90	290.85	302.10	514.73	437.25	450.33	0.74
KLMA_60387	PHO85	negative regulator of the PHO system	584.34	597.36	489.69	1454.19	1369.87	1332.83	1.31
KLMA_60388		uncharacterized protein YDR249C	403.27	728.24	430.00	1047.96	754.86	746.80	0.71
KLMA_60389	SVL3	styryl dye vacuolar localization protein 3	2347.93	2833.54	2269.37	3706.55	3503.86	3577.56	0.53
KLMA_60390	EGD1	nascent polypeptide-associated complex subunit beta	2641.86	2146.69	2483.76	1084.12	2296.59	2028.64	-0.43
KLMA_60391	MET32	zinc_figer_C2H2_2 protein	540.84	468.71	533.54	383.52	320.14	383.77	-0.50
KLMA_60392		uncharacterized protein YPL039W	957.04	737.19	920.90	575.28	450.73	540.22	-0.74
KLMA_60393	ISM1	isoleucyl-tRNA synthetase	871.21	1002.31	922.12	464.26	535.82	512.56	-0.89
KLMA_60394		uncharacterized membrane protein YPL041C	106.99	73.83	98.67	104.29	110.36	78.66	0.07
KLMA_60395	SSN3	serine/threonine-protein kinase SSN3	288.05	340.07	349.60	508.00	415.34	383.77	0.42
KLMA_60396	NOP4	nucleolar protein 4	1163.97	964.28	1235.18	415.48	562.77	592.08	-1.10
KLMA_60397	VPS16	vacuolar protein sorting-associated protein 16	540.84	522.41	520.14	679.58	687.46	629.25	0.33
KLMA_60398		elongin-C	38.80	40.27	30.45	58.87	43.81	40.62	0.39

Locus_tag	UniProt_gene	Product	Unique exon reads						log$_2$ Fold Change
			KmWT.1	KmWT.2	KmWT.3	Kmmig1.1	Kmmig1.2	Kmmig1.3	
KLMA_60399	RMD5	sporulation protein RMD5	566.70	573.87	497.00	646.77	535.82	483.17	0.02
KLMA_60400	SGF11	SAGA-associated factor 11	9.41	1.12	9.75	21.87	10.11	15.56	1.24
KLMA_60401	CTA1	peroxisomal catalase A	927.65	779.70	1035.41	1307.85	1578.80	2035.55	0.84
KLMA_60402	PDAT9	pisatin demethylase	719.55	392.65	891.67	377.64	253.59	361.30	-1.01
KLMA_60403	CAM1	putative elongation factor 1 gamma homolog	758.34	567.16	756.46	1143.84	1068.26	1224.79	0.72
KLMA_60404		hypothetical protein	139.91	163.32	172.97	354.09	312.56	423.53	1.19
KLMA_60405		37S ribosomal protein YMR-31	171.66	213.66	142.52	393.62	390.07	359.57	1.11
KLMA_60406	PDE1	low-affinity cyclic AMP phosphodiesterase	98.76	145.42	114.50	802.37	513.07	610.23	2.42
KLMA_60407	PRE4	proteasome component PRE4	1206.30	1205.91	963.54	1555.12	1647.04	1561.02	0.50
KLMA_60408	RTG2	retrograde regulation protein 2	1127.52	949.73	1157.22	918.44	1006.76	1076.12	-0.11
KLMA_60409	ZIP2	protein ZIP2	9.41	11.19	6.09	941.99	386.70	496.14	6.09
KLMA_60410	RET2	coatomer subunit delta	1943.48	1943.10	1996.51	1203.55	1556.90	1425.32	-0.49
KLMA_60411	RPN12	26S proteasome regulatory subunit RPN12	688.98	760.68	645.61	1030.30	1319.32	1051.92	0.70
KLMA_60412	RAG5	hexokinase	46460.09	33350.33	40994.82	2251.51	3216.58	2788.40	-3.87
KLMA_60413		uncharacterized esterase/lipase C417.12	138.74	147.66	162.01	550.05	401.86	484.04	1.68
KLMA_60414	DAL5	allantoate permease	14.11	32.44	7.31	1054.69	433.03	527.26	5.22
KLMA_60415		uncharacterized esterase/lipase C417.12	96.41	114.10	107.20	412.12	299.92	307.71	1.68
KLMA_60416		GAL4	463.24	406.07	404.42	869.65	619.22	684.57	0.77
KLMA_60417	cyp524A1	cytochrome P450 61	9834.97	6124.61	10720.73	1545.02	2988.27	2689.00	-1.89
KLMA_60418	BUD22	bud site selection protein 22	504.39	359.09	573.74	444.08	449.88	424.40	-0.12
KLMA_60419	SEC59	dolichol kinase	3745.87	3050.56	3889.48	3079.96	2217.40	2121.99	-0.53

Locus_tag	UniProt_gene	Product	Unique exon reads						log$_2$ Fold Change
			KmWT.1	KmWT.2	KmWT.3	Kmmig1.1	Kmmig1.2	Kmmig1.3	
KLMA_60420	LPD1	dihydrolipoyl dehydrogenase	7388.28	8757.91	7017.63	3827.66	5268.85	4722.83	-0.75
KLMA_60421		conserved hypothetical membrane protein	231.62	196.88	135.21	460.06	264.54	285.24	0.84
KLMA_60422	GIM3	prefoldin subunit 4	136.38	118.58	115.72	84.11	150.80	151.26	0.06
KLMA_60423	ARO9	aromatic amino acid aminotransferase 2	194.00	255.05	230.23	763.68	560.25	575.66	1.48
KLMA_60424	RAG8	casein kinase I homolog RAG8	1168.67	1120.89	1043.93	844.42	978.12	915.35	-0.28
KLMA_60425	GRE2	NADPH-dependent methylglyoxal reductase GRE2	960.57	1104.11	1015.92	2124.51	2313.44	2498.85	1.17
KLMA_60426		putative lipase YJR107W	4.70	2.24	4.87	21.87	20.22	10.37	2.16
KLMA_60427	ADO1	adenosine kinase	11979.50	5117.83	8888.67	3382.74	7463.51	6779.99	-0.56
KLMA_60428	TDA4	uncharacterized TLC domain-containing protein YJR116W	679.57	696.92	661.44	576.97	654.60	676.79	-0.09
KLMA_60429	STE24	CAAX prenyl protease 1	1713.04	1572.82	1671.27	1376.81	1807.11	1875.65	0.03
KLMA_60430	RPN3	26S proteasome regulatory subunit RPN3	1595.46	1766.35	1583.56	1436.53	1584.70	1709.69	-0.06
KLMA_60431	SRB4	mediator of RNA polymerase II transcription subunit 17	553.77	534.71	572.52	654.34	700.94	607.64	0.24
KLMA_60432	ILM1	protein ILM1	346.84	328.88	313.06	276.71	286.44	297.34	-0.20
KLMA_60433	JHD2	histone demethylase JHD2	1148.69	1064.96	1037.84	648.46	411.97	467.62	-1.09
KLMA_60434	PRO3	pyrroline-5-carboxylate reductase	1511.99	1236.11	1618.89	1176.64	1155.04	1080.44	-0.36
KLMA_60435	ATP2	ATP synthase subunit beta	20264.86	21805.85	19569.20	15919.56	20268.32	17865.32	-0.19
KLMA_60437	CAF17	putative transferase CAF17	69.37	134.24	119.38	101.77	131.43	114.09	0.10
KLMA_60438	RPS5	40S ribosomal protein S5	25721.41	20268.83	23837.51	9718.43	15487.26	12646.36	-0.88
KLMA_60439	YAT2	carnitine O-acetyltransferase YAT2	505.56	345.66	375.18	594.63	409.44	458.97	0.25

Locus_tag	UniProt_gene	Product	Unique exon reads						log2 Fold Change
			KmWT.1	KmWT.2	KmWT.3	Kmmig1.1	Kmmig1.2	Kmmig1.3	
KLMA_60440		uncharacterized membrane protein YJR124C	372.71	213.66	300.88	206.06	174.39	210.04	-0.59
KLMA_60441	LEU3	regulatory protein LEU3	1317.99	1099.63	1442.26	378.48	329.41	411.43	-1.79
KLMA_60442	GCD11	eukaryotic translation initiation factor 2 subunit gamma	4978.03	4456.70	4781.15	2241.42	3218.26	3000.17	-0.75
KLMA_60443	SST2	protein SST2	236.32	232.68	230.23	1261.59	819.73	880.78	2.08
KLMA_60444	BUR1	serine/threonine-protein kinase BUR1	651.35	624.21	634.64	481.93	472.63	526.39	-0.37
KLMA_60445	ERG13	hydroxymethylglutaryl-CoA synthase	4166.78	2907.37	4158.68	1152.25	1588.07	1656.97	-1.35
KLMA_60446	RPM2	ribonuclease P protein component	1750.66	1598.55	1660.31	1859.58	2011.84	1761.55	0.17
KLMA_60447	RSC9	chromatin structure-remodeling complex subunit RSC9	1048.75	951.97	975.72	402.87	645.34	606.78	-0.85
KLMA_60448	KNH1	cell wall synthesis protein KNH1	811.25	906.11	744.28	1406.25	1264.56	1289.62	0.69
KLMA_60449	LHP1	la protein homolog	369.18	448.58	433.65	304.46	466.73	419.21	-0.07
KLMA_60450		platinum sensitivity protein 3	117.57	135.36	143.74	486.97	272.96	296.47	1.41
KLMA_60451	SLC1	probable 1-acyl-sn-glycerol-3-phosphate acyltransferase	881.80	991.12	909.94	753.59	905.66	827.19	-0.16
KLMA_60452	FBP1	fructose-1,6-bisphosphatas	63.49	55.93	71.87	650.14	350.47	401.92	2.88
KLMA_60453		hypothetical protein	79.95	64.88	79.18	82.42	83.41	133.97	0.42
KLMA_60454	ATG3	autophagy-related protein 3	171.66	250.58	183.94	947.03	610.80	757.17	1.93
KLMA_60455	LRO1	phospholipid:diacylglycerol acyltransferase	686.63	588.41	702.86	873.02	815.52	964.62	0.42
KLMA_60456	NRM1	transcription factor NRM1	458.53	289.73	450.71	192.60	379.96	375.99	-0.34
KLMA_60457	ERG2	c-8 sterol isomerase	833.59	656.65	800.31	427.26	767.50	752.85	-0.23
KLMA_60458	CSE2	mediator of RNA polymerase II transcription subunit	128.15	121.93	131.56	126.16	157.54	163.36	0.23
KLMA_60459	TOM40	mitochondrial import receptor subunit	2621.87	2340.22	2307.13	857.88	1656.31	1498.79	-0.86

Locus_tag	UniProt_gene	Product	Unique exon reads						log$_2$ Fold Change
			KmWT.1	KmWT.2	KmWT.3	Kmmig1.1	Kmmig1.2	Kmmig1.3	
		TOM40							
KLMA_60460	INP1	inheritance of peroxisomes protein 1	215.16	256.17	185.16	571.92	465.05	407.11	1.14
KLMA_60461	PFK2	6-phosphofructokinase subunit beta	48076.72	33478.97	41578.30	5874.79	9787.06	9006.56	-2.32
KLMA_60462	GLO1	lactoylglutathione lyase	1096.95	1271.91	940.39	1330.55	1556.90	1541.14	0.42
KLMA_60463	PRP2	pre-mRNA-splicing factor ATP-dependent RNA helicase-like protein PRP2	159.90	161.09	169.32	200.17	181.98	201.39	0.25
KLMA_60464	URK1	uridine kinase	258.66	281.90	259.46	195.97	184.50	207.44	-0.44
KLMA_60465	PHO91	uncharacterized transporter YNR013C	1995.21	1711.54	1852.77	1843.60	1479.39	1490.15	-0.21
KLMA_60466		uncharacterized protein YMR206W	920.60	819.97	780.82	666.96	615.01	548.00	-0.46
KLMA_60467		hypothetical protein	554.94	644.34	489.69	659.39	597.32	739.89	0.24
KLMA_60468	SMM1	tRNA-dihydrouridine synthase 2	673.69	479.90	645.61	232.13	258.64	254.98	-1.27
KLMA_60469	ACC1	acetyl-CoA carboxylase	10933.10	14342.22	12517.47	4612.37	7391.05	7627.92	-0.94
KLMA_60470	TIM23	mitochondrial import inner membrane translocase subunit TIM23	1661.30	1092.92	1327.76	682.10	1302.47	1135.76	-0.39
KLMA_60471	RRT12	putative subtilase-type proteinase YCR045C	29.39	23.49	31.67	46.26	32.01	34.57	0.42
KLMA_60472	RCF2	altered inheritance rate of mitochondria protein 38	801.85	1067.19	807.62	811.62	616.69	600.73	-0.40
KLMA_60473	IMG1	54S ribosomal protein IMG1	357.42	307.63	359.35	194.28	346.26	283.51	-0.31
KLMA_60474	BUD23	putative methyltransferase BUD23	389.17	312.10	403.20	316.24	264.54	297.34	-0.33
KLMA_60475		uncharacterized protein YIL077C	198.70	237.15	166.88	582.01	383.33	412.30	1.19

Locus_tag	UniProt_gene	Product	Unique exon reads						log2 Fold Change
			KmWT.1	KmWT.2	KmWT.3	Kmmig1.1	Kmmig1.2	Kmmig1.3	
KLMA_60476	THS1	threonyl-tRNA synthetase	3910.47	3289.95	3871.21	1478.58	2352.20	2147.05	-0.89
KLMA_60477	AIR1	protein AIR2	311.57	390.41	467.76	232.13	206.41	229.05	-0.81
KLMA_60478		protein SNA4	249.25	253.93	224.14	306.15	253.59	261.03	0.17
KLMA_60479		uncharacterized protein YDL176W	259.84	270.71	231.44	650.98	397.65	407.97	0.93
KLMA_60480		IMPACT family member YDL177C	119.92	93.97	98.67	206.90	144.06	174.60	0.75
KLMA_60481		NADPH-dependent alpha-keto amide reductase	563.17	711.46	622.46	1149.73	1160.09	1248.13	0.91
KLMA_60482	DLD2	D-lactate dehydrogenase [cytochrome] 2	2297.37	1698.11	2277.90	1063.94	1314.26	1355.31	-0.75
KLMA_60483	HNT1	hit family protein 1	539.66	586.17	571.30	395.30	451.57	399.33	-0.45
KLMA_60484	CDC48	cell division control protein 48	4592.40	6236.48	4589.90	3601.41	3800.41	4059.00	-0.43
KLMA_60486	PCL2	PHO85 cyclin-2	2188.03	2051.61	2199.94	1222.90	1447.38	1427.91	-0.65
KLMA_60487	VCX1	vacuolar calcium ion transporter	1355.61	1353.57	1254.67	5350.82	3595.69	3377.03	1.64
KLMA_60489		uncharacterized membrane protein YDL180W	142.26	154.37	132.78	296.05	277.18	239.43	0.92
KLMA_60490		uncharacterized protein YDL129W	727.78	544.78	613.94	668.64	443.14	481.44	-0.24
KLMA_60491	RPP1B	60S acidic ribosomal protein P1-beta	2299.73	1448.65	2236.48	741.81	1572.06	1264.55	-0.74
KLMA_60492		ATPase inhibitor	1299.18	1371.47	1494.64	1529.04	1398.51	1214.42	-0.01
KLMA_60493	LYS21	homocitrate synthase	6116.14	4792.30	5646.02	4784.78	6844.28	6405.72	0.12
KLMA_60494	CDC53	cell division control protein 53	1088.72	1076.14	933.08	767.89	791.93	829.78	-0.37
KLMA_60495	SRF1	hypothetical membrane protein	179.89	218.14	132.78	534.07	443.14	370.81	1.34
KLMA_60496		uncharacterized protein YDL183C	205.75	221.49	198.55	852.83	588.89	573.07	1.69
KLMA_60497	VMA1	v-type proton ATPase catalytic subunit A	5709.34	5137.96	5464.51	3753.64	5758.33	5278.61	-0.14
KLMA_60498		uncharacterized protein YDL186W	17.64	32.44	10.96	389.41	117.10	182.38	3.49
KLMA_60499	PPH21	serine/threonine-	1857.65	2078.45	1883.22	1977.33	1877.88	1916.27	-0.01

Locus_tag	UniProt_gene	Product	Unique exon reads						log$_2$ Fold Change
			KmWT.1	KmWT.2	KmWT.3	Kmmig1.1	Kmmig1.2	Kmmig1.3	
KLMA_60500		protein phosphatase PP2A-1 catalytic subunit probable allantoinase 1	65.84	76.07	51.16	316.24	215.67	263.63	2.04
KLMA_60501	RDI1	rho GDP-dissociation inhibitor	198.70	294.21	215.61	373.43	380.80	372.54	0.67
KLMA_60502	RBS1	r3H_encore_like protein	390.34	503.39	356.91	281.75	254.43	277.46	-0.62
KLMA_60503	UFD2	ubiquitin conjugation factor E4	1442.62	1724.96	1293.65	1678.75	1490.34	1612.02	0.10
KLMA_60504	RPL35B	60S ribosomal protein L35	21261.87	16362.50	20029.65	9397.15	17027.31	13116.57	-0.54
KLMA_60505	ARF1	ADP-ribosylation factor	2176.27	2213.81	2140.25	1321.30	1750.67	1495.33	-0.52
KLMA_60506	NUS1	probable undecaprenyl pyrophosphate synthetase	99.94	111.87	97.45	63.92	103.62	123.60	-0.09
KLMA_60507	SNF3	high-affinity glucose transporter SNF3	1303.88	1058.24	1383.79	633.32	659.66	638.76	-0.96
KLMA_60508	SEC31	protein transport protein SEC31	2814.69	3363.78	3062.37	1390.27	1767.52	1835.02	-0.89
KLMA_60509	COA4	coiled-coil-helix-coiled-coil-helix domain-containing	24.69	29.08	20.71	25.23	31.17	29.39	0.20
KLMA_60510	SCM3	protein YLR218C	64.67	60.41	63.34	114.38	105.31	97.67	0.75
KLMA_60511	CPR6	peptidyl-prolyl cis-trans isomerase D	667.81	594.00	674.84	661.07	978.96	762.36	0.31
KLMA_60512	RPB1	DNA-directed RNA polymerase II subunit RPB1	8204.23	7232.08	7898.33	2993.33	3745.65	4204.22	-1.09
KLMA_60514	BPL1	biotin--protein ligase	798.32	959.80	784.47	862.09	753.17	824.59	-0.06
KLMA_60515	CRD1	probable cardiolipin synthetase 1	236.32	214.78	257.02	301.94	300.76	247.21	0.26
KLMA_60516	CCT4	T-complex protein 1 subunit delta	2101.03	2354.76	2193.85	1276.73	1486.97	1628.44	-0.60
KLMA_60519	CDC123	cell division cycle protein 123	1002.90	986.65	913.59	905.82	711.05	766.68	-0.28
KLMA_60520	FRE1	ferric/cupric reductase transmembrane component 1	545.54	665.60	534.76	795.64	776.76	542.81	0.28

Locus_tag	UniProt_gene	Product	Unique exon reads						log$_2$ Fold Change
			KmWT.1	KmWT.2	KmWT.3	Kmmig1.1	Kmmig1.2	Kmmig1.3	
KLMA_60521		uncharacterized protein YDL144C	984.08	938.55	926.99	951.24	1172.73	1030.31	0.15
KLMA_60522	CRR1	probable glycosidase CRR1	130.51	117.46	77.96	611.45	302.45	286.10	1.88
KLMA_60523	COP1	coatomer subunit alpha	5311.94	5093.22	4935.85	2656.90	3135.70	3329.49	-0.75
KLMA_60524	SPC42	spindle pole body component SPC42	146.97	106.27	75.52	86.63	109.52	129.65	-0.01
KLMA_60525	SOK2	kilA-N super family protein	939.41	1127.60	996.43	813.30	702.63	758.04	-0.43
KLMA_60526	PRI2	DNA primase large subunit	449.13	484.38	366.66	1005.07	501.27	584.30	0.69
KLMA_60527	MSK1	lysyl-tRNA synthetase	211.63	198.00	242.41	194.28	207.25	235.97	-0.03
KLMA_60528	RNH201	ribonuclease H2 subunit A	186.94	220.37	148.61	171.58	165.97	182.38	-0.10
KLMA_60529	LAT1	dihydrolipoyllysine-residue acetyltransferase component of pyruvate dehydrogenase complex	2839.38	3565.14	2562.94	1099.26	1930.12	1867.00	-0.87
KLMA_60530		mitochondrial import receptor subunit TOM7	103.46	69.36	112.07	49.62	114.58	57.91	-0.36
KLMA_60531	FLX1	mitochondrial FAD carrier protein FLX1	189.29	208.07	182.72	227.93	191.24	210.90	0.12
KLMA_60532	RPL16A	60S ribosomal protein L16-A	21628.70	18385.02	21564.49	6679.69	13140.96	11033.47	-1.00
KLMA_60533	CSM2	chromosome segregation in meiosis protein 2	21.16	38.03	28.02	37.01	53.08	41.49	0.59
KLMA_60534	FKH2	fork head protein homolog 2	848.88	866.95	901.41	550.05	576.25	648.27	-0.56
KLMA_60535	ASG1	cutinase transcription factor 1 alpha	1436.74	1485.57	1465.41	931.05	869.44	761.50	-0.78
KLMA_60536	TAO3	cell morphogenesis protein PAG1	1066.39	1017.97	1096.31	880.59	804.57	885.96	-0.31
KLMA_60537	MET18	DNA repair/transcription protein MET18/MMS19	848.88	889.33	884.36	708.17	750.65	783.97	-0.23

Locus_tag	UniProt_gene	Product	Unique exon reads						log2 Fold Change
			KmWT.1	KmWT.2	KmWT.3	Kmmig1.1	Kmmig1.2	Kmmig1.3	
KLMA_60538	RRT14	uncharacterized protein YIL127C	363.30	276.31	348.38	202.70	186.19	184.11	-0.79
KLMA_60539	STH1	nuclear protein STH1/NPS1	1748.31	1740.62	1785.77	881.43	1115.44	939.55	-0.84
KLMA_60540	RPL9B	60S ribosomal protein L9-B	4299.64	3204.93	4453.47	1945.37	2465.93	1899.85	-0.92
KLMA_60541	KGD1	2-oxoglutarate dehydrogenase E1 component	3424.90	3427.55	3269.45	4159.88	4684.17	4849.89	0.44
KLMA_60542	AYR1	NADPH-dependent 1-acyldihydroxyacetone phosphate reductase	774.81	695.80	628.55	727.52	879.55	893.74	0.25
KLMA_60543	SIM1	septation protein SUN4	14867.09	10309.48	12843.92	15525.10	12939.61	11097.44	0.06
KLMA_60545		hypothetical protein	534.96	502.27	509.18	468.47	411.97	483.17	-0.18
KLMA_60546	REB1	DNA-binding protein REB1	1688.35	1628.76	1841.81	1641.75	1683.27	1494.47	-0.10
KLMA_60547	VPS54	vacuolar protein sorting-associated protein 54	291.58	393.76	286.26	390.25	382.48	406.25	0.28
KLMA_60548	REG1	protein HEX2	2506.65	2604.22	2305.91	1471.01	1609.13	1614.61	-0.66
KLMA_60549	PST2	protoplast secreted protein 2	4491.28	3426.43	3854.15	3208.64	3565.36	3423.70	-0.21
KLMA_60551		uncharacterized protein YBR053C	523.20	676.78	440.96	941.99	1293.20	1185.89	1.06
KLMA_60552	ERC1	uncharacterized transporter C4B3.13	343.31	345.66	331.33	882.27	792.77	818.54	1.29
KLMA_60553		putative agmatinase 1	663.11	1009.02	662.66	705.65	609.11	710.50	-0.21
KLMA_60554	LYS14	lysine biosynthesis regulatory protein LYS14	1047.57	990.01	1074.39	862.09	844.16	833.24	-0.29
KLMA_60555	PRP6	pre-mRNA-splicing factor 6	696.03	692.44	689.46	776.30	496.22	527.26	-0.21
KLMA_60556	PHO89	phosphate permease PHO89	268.07	244.98	314.28	449.97	377.43	406.25	0.58
KLMA_60557	mtr	N-amino acid transport system protein	792.44	824.45	870.96	1302.80	662.19	792.61	0.15
KLMA_60558		aminotriazole resistance protein	179.89	184.58	166.88	817.51	448.20	593.81	1.81

Locus_tag	UniProt_gene	Product	Unique exon reads						log_2 Fold Change
			KmWT.1	KmWT.2	KmWT.3	Kmmig1.1	Kmmig1.2	Kmmig1.3	
KLMA_70001		aminotriazole resistance protein	118.75	118.58	124.25	423.89	198.82	254.98	1.28
KLMA_70002		arylsulfatase	108.17	100.68	147.39	520.62	192.08	235.10	1.41
KLMA_70003	HXT15	hexose transporter HXT9	61.14	58.17	38.98	721.63	247.69	238.56	2.93
KLMA_70004		conserved hypothetical protein	238.67	212.54	210.74	1367.56	501.27	477.99	1.83
KLMA_70005		conserved hypothetical membrane protein	633.72	570.51	611.50	1100.95	790.24	840.15	0.59
KLMA_70006		uncharacterized transcriptional regulatory protein YNR063W	566.70	667.83	643.17	1147.20	846.69	905.84	0.63
KLMA_70007	FCY2	purine-cytosine permease FCY2	438.55	288.61	587.14	578.65	215.67	233.38	-0.35
KLMA_70008		conserved hypothetical protein	295.11	257.29	331.33	371.75	258.64	303.39	0.08
KLMA_70009		flocculation protein FLO9	82.30	42.51	60.91	95.04	42.12	55.32	0.05
KLMA_70011		uncharacterized protein YJL171C	1876.46	2568.42	1587.22	4752.82	3577.16	5154.14	1.16
KLMA_70012		1-aminocyclopropane-1-carboxylate oxidase	226.92	217.02	238.75	1046.28	662.19	697.53	1.82
KLMA_70013	VBA2	vacuolar basic amino acid transporter 2	289.23	247.22	314.28	591.26	462.52	584.30	0.95
KLMA_70014	AMD1	AMP deaminase	1220.41	1397.19	1333.85	839.38	882.07	827.19	-0.63
KLMA_70015	PFA5	palmitoyltransferase PFA5	484.40	457.53	443.40	851.99	616.69	662.09	0.62
KLMA_70016	ENV9	uncharacterized oxidoreductase YOR246C	1135.75	965.40	1048.81	763.68	689.15	646.54	-0.59
KLMA_70017	CDC60	leucyl-tRNA synthetase	8451.14	6595.56	8412.38	2524.86	3020.28	2754.69	-1.50
KLMA_70019	PET20	protein PET20	222.21	240.51	233.88	433.15	353.00	329.32	0.68
KLMA_70020		putative esterase YMR210W	323.33	378.10	363.00	421.37	385.01	358.71	0.13
KLMA_70021	AIM44	uncharacterized protein YPL158C	893.55	692.44	750.37	851.99	863.54	899.79	0.16
KLMA_70022	TGS1	trimethylguanosine synthase	212.81	206.95	236.32	404.55	280.54	343.15	0.65

Locus_tag	UniProt_gene	Product	Unique exon reads						log$_2$ Fold Change
			KmWT.1	KmWT.2	KmWT.3	Kmmig1.1	Kmmig1.2	Kmmig1.3	
KLMA_70023	PRM4	pheromone-regulated membrane protein 4	273.94	393.76	259.46	1270.00	794.46	878.18	1.67
KLMA_70024	KIP2	kinesin-like protein KIP2	250.43	233.80	241.19	1105.99	673.98	744.21	1.80
KLMA_70025	PEP4	saccharopepsin	6607.59	7468.11	6259.95	14680.68	11509.92	11660.99	0.90
KLMA_70026	SWP82	SWI/SNF global transcription activator complex subunit SWP82	873.57	822.21	772.29	2008.45	1432.21	1306.90	0.94
KLMA_70027	EMP47	protein EMP47	798.32	832.28	729.66	937.78	1069.95	995.74	0.35
KLMA_70028		conserved hypothetical protein	325.68	369.15	292.35	477.72	315.09	345.74	0.21
KLMA_70029	RGD2	rho-GTPase-activating protein RGD2	643.12	736.07	503.09	831.81	615.01	788.29	0.25
KLMA_70030		protein SIC1	151.67	209.19	153.48	348.20	385.01	318.95	1.03
KLMA_70031	BOS1	protein transport protein BOS1	377.41	299.80	327.68	600.52	544.24	574.80	0.78
KLMA_70032	FMP25	protein FMP25	705.44	851.29	734.53	573.60	651.23	602.45	-0.33
KLMA_70033	FMP32	protein FMP32	85.83	92.85	90.14	250.64	165.97	172.01	1.13
KLMA_70034	SEC53	phosphomannomutase	3380.22	2525.91	3427.81	1452.51	2738.89	2617.26	-0.46
KLMA_70035		hypothetical protein	242.20	202.48	229.01	142.98	131.43	149.53	-0.67
KLMA_70036		hypothetical protein	526.73	458.65	542.07	343.99	278.02	326.73	-0.69
KLMA_70037	RPL10	60S ribosomal protein L10	20421.23	16506.81	19380.39	8102.76	12752.58	10864.92	-0.83
KLMA_70039	BUD20	bud site selection protein 20	710.14	563.80	762.55	419.69	396.81	424.40	-0.71
KLMA_70040	OTU1	ubiquitin thioesterase OTU1	68.19	115.22	92.58	227.09	240.11	265.36	1.41
KLMA_70041	RFU1	regulator of free ubiquitin chains 1	79.95	85.02	110.85	65.60	85.93	46.68	-0.47
KLMA_70042		uncharacterized protein YFL042C	723.07	720.41	615.15	534.07	540.03	524.66	-0.37
KLMA_70043	RGR1	mediator of RNA polymerase II transcription subunit 14	947.64	1091.80	1000.08	721.63	874.49	860.03	-0.31
KLMA_70044	SOR1	sorbitol dehydrogenase 1	1211.00	1267.43	1018.35	11024.60	18283.44	18287.13	3.77

Locus_tag	UniProt_gene	Product	Unique exon reads						log$_2$ Fold Change
			KmWT.1	KmWT.2	KmWT.3	Kmmig1.1	Kmmig1.2	Kmmig1.3	
KLMA_70045	FET5	iron transport multicopper oxidase FET5	1086.37	1033.63	1054.90	897.41	965.48	948.20	-0.18
KLMA_70046	MEF1	elongation factor G	1601.34	1626.52	1387.45	781.34	1166.83	1109.83	-0.59
KLMA_70047	FYV7	rRNA-processing protein FYV7	182.24	143.19	149.83	213.63	152.49	125.33	0.05
KLMA_70048	PET309	pentatricopeptide repeat-containing protein PET309	1275.67	1134.31	1310.70	524.82	621.75	561.83	-1.12
KLMA_70049	SPC3	microsomal signal peptidase subunit 3	492.63	466.48	497.00	630.79	596.47	518.61	0.26
KLMA_70050		probable metabolite transport protein YFL040W	10.58	24.61	28.02	123.64	26.96	26.79	1.49
KLMA_70051	ACT	actin	28556.09	22858.50	26525.92	18419.18	24548.94	23965.07	-0.22
KLMA_70053		transmembrane protein 208 homolog	262.19	259.53	204.65	814.14	645.34	721.74	1.59
KLMA_70054	YPT1	GTP-binding protein YPT1	1080.49	1122.01	1024.44	1830.98	1901.47	1687.22	0.75
KLMA_70055	PER33	UPF0121 membrane protein YLR064W	560.82	387.05	523.79	1049.64	1043.83	1048.46	1.09
KLMA_70056	TUB2	tubulin beta chain	3197.98	2958.83	3039.23	1626.61	2568.71	2532.56	-0.45
KLMA_70058	RPO41	DNA-directed RNA polymerase	1305.06	1368.11	1315.58	847.79	846.69	785.70	-0.69
KLMA_70059	MOB2	maintenance of ploidy protein mob2	523.20	552.61	566.43	679.58	562.77	535.90	0.11
KLMA_70060		uncharacterized protein YLR063W	686.63	601.83	633.43	745.18	530.76	597.27	-0.04
KLMA_70061	ATP10	mitochondrial ATPase complex subunit ATP10	166.95	253.93	205.86	403.71	372.37	379.45	0.88
KLMA_70062		covalently-linked cell wall protein 14	8185.42	8203.06	6850.74	8401.33	5476.10	5770.42	-0.24
KLMA_70063	PEX19	peroxisomal membrane protein import receptor PEX19	328.03	341.19	278.95	478.56	492.85	547.14	0.68
KLMA_70064	UBC9	SUMO-conjugating enzyme UBC9	397.40	434.04	425.13	360.81	440.62	458.97	0.00
KLMA_70065		uncharacterized protein YDL063C	906.49	731.60	946.48	560.99	614.17	607.64	-0.54
KLMA_70066		conserved hypothetical	150.49	102.92	151.05	43.74	69.08	46.68	-1.34

Locus_tag	UniProt_gene	Product	Unique exon reads						$\log_2$ Fold Change
			KmWT.1	KmWT.2	KmWT.3	Kmmig1.1	Kmmig1.2	Kmmig1.3	
		membrane protein							
KLMA_70067	STE23	a-factor-processing enzyme	1036.99	1105.23	1070.73	973.95	908.19	901.52	-0.21
KLMA_70068	RPS29	40S ribosomal protein S29	2521.94	2019.16	2363.16	1269.16	1960.44	1503.98	-0.54
KLMA_70069	TSR1	ribosome biogenesis protein TSR1	2396.13	2144.45	2745.66	1035.34	1313.42	1339.75	-0.98
KLMA_70070	REH1	zinc finger protein REH1	577.28	464.24	598.10	388.57	464.20	430.45	-0.35
KLMA_70071	VAC14	vacuole morphology and inheritance protein 14	743.06	752.85	838.07	637.52	630.17	624.06	-0.30
KLMA_70072	SWC7	SWR1-complex protein 7	27.04	41.39	26.80	28.60	40.44	28.52	0.03
KLMA_70073	IKI3	elongator complex protein 1	1576.65	1361.40	1536.06	608.93	854.27	758.90	-1.01
KLMA_70074	RAD59	DNA repair protein RAD59	310.39	236.04	356.91	245.59	199.67	235.97	-0.41
KLMA_70075	USO1	intracellular protein transport protein USO1	1045.22	1180.18	981.81	752.75	939.36	885.96	-0.32
KLMA_70076	SMC6	structural maintenance of chromosomes protein 6	566.70	589.53	573.74	676.21	598.16	550.59	0.08
KLMA_70077	NAM2	leucyl-tRNA synthetase	410.33	501.16	466.54	608.09	492.01	512.56	0.23
KLMA_70078	CTF3	central kinetochore subunit CTF3	472.64	426.21	444.62	605.56	501.27	471.07	0.23
KLMA_70079	CSR1	phosphatidylinositol transfer protein CSR1	1750.66	1523.60	1668.83	1101.79	1327.74	1262.82	-0.42
KLMA_70080		conserved hypothetical protein	2767.66	2571.78	2477.67	1153.09	1083.43	1083.04	-1.24
KLMA_70081	MBP1	transcription factor MBP1	586.69	655.53	567.65	516.41	516.44	560.10	-0.18
KLMA_70082	MPG1	mannose-1-phosphate guanyltransferase	13217.54	11110.44	11547.84	4669.56	7610.10	7149.07	-0.88
KLMA_70083	FUI1	uridine permease	3958.68	2667.98	3537.44	883.95	637.76	636.16	-2.24
KLMA_70084	VPS25	vacuolar protein-sorting-associated protein 25	50.56	49.22	35.33	52.99	53.92	66.56	0.36

Locus_tag	UniProt_gene	Product	Unique exon reads						log2 Fold Change
			KmWT.1	KmWT.2	KmWT.3	Kmmig1.1	Kmmig1.2	Kmmig1.3	
KLMA_70085		central kinetochore subunit MCM16	17.64	38.03	14.62	33.64	54.76	44.08	0.91
KLMA_70086	URA7	CTP synthase	4552.42	3584.16	4430.33	1385.22	1630.19	1520.40	-1.47
KLMA_70087	SOD1	superoxide dismutase [Cu-Zn]	3467.22	3947.72	3623.93	4120.35	4662.27	4495.50	0.27
KLMA_70089		conserved hypothetical membrane protein	12.93	4.47	2.44	38.69	26.96	22.47	2.15
KLMA_70090		conserved hypothetical protein	497.33	384.82	483.60	717.42	666.40	745.94	0.64
KLMA_70091	ERD2	ER lumen protein retaining receptor	467.94	368.04	419.04	597.15	647.02	716.55	0.64
KLMA_70092	PRE7	proteasome component C5	779.51	907.23	772.29	932.73	1100.28	1004.38	0.30
KLMA_70093	CPS1	carboxypeptidase S	1290.95	1355.80	1308.27	1249.81	1371.55	1517.80	0.07
KLMA_70094		hypothetical protein	58.79	69.36	49.94	358.29	512.23	361.30	2.79
KLMA_70095	COR1	cytochrome b-c1 complex subunit 1	8402.93	7374.14	8210.17	4036.24	6315.21	5383.19	-0.61
KLMA_70096	PSY4	serine/threonine-protein phosphatase 4 regulatory subunit 2	316.27	313.22	391.02	302.78	350.47	325.00	-0.06
KLMA_70097	RSM7	37S ribosomal protein S7	617.26	578.34	551.81	333.90	459.15	433.91	-0.51
KLMA_70098		uncharacterized protein YJR112W-A	105.82	112.98	124.25	164.85	79.19	74.33	-0.11
KLMA_70099	EDE1	EH domain-containing and endocytosis protein 1	3058.07	3789.99	2810.22	3264.99	3470.16	3403.82	0.07
KLMA_70100	NNF1	kinetochore-associated protein NNF1	105.82	116.34	135.21	186.72	180.29	160.77	0.56
KLMA_70101		uncharacterized protein YJR111C	252.78	269.59	249.72	322.13	295.71	312.90	0.27
KLMA_70102	YMR1	phosphoinositide 3-phosphatase	297.46	422.85	364.22	849.47	667.24	701.86	1.03
KLMA_70103	MOH1	protein yippee-like MOH1	304.51	392.65	202.21	1894.06	834.89	853.12	1.99
KLMA_70104	CFL1	ferric reductase transmembrane component 3	322.15	299.80	276.51	2035.36	495.38	548.86	1.78
KLMA_70105	CFL1	probable ferric	3477.80	1411.74	2767.58	1121.13	1157.56	573.07	-1.42

Locus_tag	UniProt_gene	Product	Unique exon reads						log2 Fold Change
			KmWT.1	KmWT.2	KmWT.3	Kmmig1.1	Kmmig1.2	Kmmig1.3	
		reductase transmembrane component							
KLMA_70106	SEC17	vesicular-fusion protein SEC17	961.75	1048.18	974.50	1224.58	1130.60	1056.24	0.19
KLMA_70107		hypothetical protein	146.97	142.07	136.43	1015.16	486.11	587.76	2.30
KLMA_70108	FIP1	pre-mRNA polyadenylation factor FIP1	379.76	374.75	415.38	746.86	573.73	507.38	0.64
KLMA_70109	BUD4	bud site selection protein 4	576.11	611.90	702.86	560.99	862.70	852.25	0.27
KLMA_70110	JSN1	protein JSN1	1654.25	2077.33	1537.28	1935.28	1490.34	1735.62	-0.03
KLMA_70111	GRR1	SCF E3 ubiquitin ligase complex F-box protein GRR1	944.11	1058.24	818.58	527.34	435.56	599.86	-0.85
KLMA_70112		conserved hypothetical protein	258.66	253.93	296.00	344.83	277.18	274.86	0.15
KLMA_70113	TIF5	eukaryotic translation initiation factor 5	2319.71	2455.44	2310.79	947.03	1527.41	1425.32	-0.86
KLMA_70115	EMC2	ER membrane protein complex subunit 2	244.55	225.97	213.17	342.31	345.42	344.01	0.59
KLMA_70116	TIP41	type 2A phosphatase activator TIP41	275.12	333.36	336.20	442.40	416.18	430.45	0.45
KLMA_70117	ERV2	FAD-linked sulfhydryl oxidase ERV2	250.43	215.90	164.45	605.56	411.97	413.16	1.18
KLMA_70118		guanine nucleotide-binding protein subunit gamma	4.70	4.47	2.44	121.95	61.50	46.68	4.30
KLMA_70119		UPF0136 membrane protein YJR085C	325.68	336.71	336.20	448.28	407.76	390.69	0.32
KLMA_70120	CSN12	COP9 signalosome complex subunit 12	148.14	168.92	142.52	142.14	158.39	149.53	-0.03
KLMA_70121		assembly-complementing factor 4	98.76	82.78	93.80	168.21	163.44	140.89	0.78
KLMA_70122	VMA13	v-type proton ATPase subunit H	2133.95	2103.06	2264.50	2338.14	2652.96	2562.81	0.22
KLMA_70123	GLN1	glutamine synthetase	9840.85	5809.15	6468.25	4826.84	7321.13	7328.85	-0.18
KLMA_70124	ARP7	actin-related protein 7	781.86	781.94	846.60	619.02	640.28	649.13	-0.34

Locus_tag	UniProt_gene	Product	Unique exon reads						log$_2$ Fold Change
			KmWT.1	KmWT.2	KmWT.3	Kmmig1.1	Kmmig1.2	Kmmig1.3	
KLMA_70125	EAF6	chromatin modification-related protein EAF6	145.79	155.49	199.77	245.59	198.82	170.28	0.30
KLMA_70126	AIM24	uncharacterized protein YJR080C	1269.79	1092.92	1298.52	424.73	459.15	453.79	-1.45
KLMA_70127	HARS	histidyl-tRNA synthetase	2253.87	2007.98	2185.32	1237.20	1973.92	1939.61	-0.32
KLMA_70128		uncharacterized protein YBL107C	218.69	161.09	190.03	135.41	193.77	170.28	-0.19
KLMA_70129	SRO77	protein SNI2	905.31	943.02	855.12	811.62	695.89	870.40	-0.19
KLMA_70130	PKC1	protein kinase C-like 1	2411.42	2388.32	2553.19	1383.54	1390.09	1427.05	-0.81
KLMA_70131	SEA4	WD repeat-containing protein YBL104C	777.16	793.12	894.10	709.01	684.09	690.62	-0.24
KLMA_70132	NTO1	nuA3 HAT complex component NTO1	283.35	362.44	383.71	870.50	646.18	667.28	1.09
KLMA_70133	RTG3	retrograde regulation protein 3	592.57	574.99	594.45	523.14	517.28	526.39	-0.17
KLMA_70134	SFT2	protein transport protein SFT2	437.37	345.66	472.63	471.83	522.34	536.76	0.29
KLMA_70136	ECM21	protein ECM21	911.19	1111.94	1047.59	1571.10	1154.19	1386.42	0.42
KLMA_70137	APL4	AP-1 complex subunit gamma-1	978.21	1137.67	845.38	531.55	653.76	712.23	-0.64
KLMA_70138	YOP1	protein YOP1	1443.79	1393.84	1293.65	1334.76	1481.92	1393.34	0.03
KLMA_70139	ATH1	vacuolar acid trehalase	447.95	693.56	370.31	1506.34	823.10	965.48	1.12
KLMA_70140	ATP1	ATP synthase subunit alpha	24890.17	25486.21	23991.00	12114.61	19656.68	17752.09	-0.59
KLMA_70141	CCL1	cyclin CCL1	204.58	267.36	222.92	219.52	227.47	270.54	0.05
KLMA_70142	YME1	protein YME1	1904.68	2100.83	1760.19	2532.43	1912.42	1847.99	0.13
KLMA_70144	BRN1	condensin complex subunit 2	402.10	450.82	393.45	306.15	425.45	391.55	-0.15
KLMA_70145	ywtG	conserved hypothetical membrane protein	32.92	86.14	36.54	1026.09	318.46	403.65	3.49
KLMA_70146		uncharacterized protein YBL095W	215.16	229.32	210.74	551.73	394.28	426.13	1.07
KLMA_70147	GLO3	ADP-ribosylation factor GTPase-activating protein GLO3	971.15	1047.06	1026.88	1030.30	1096.91	1127.12	0.10

Locus_tag	UniProt_gene	Product	Unique exon reads						log2 Fold Change
			KmWT.1	KmWT.2	KmWT.3	Kmmig1.1	Kmmig1.2	Kmmig1.3	
KLMA_70148	ROX3	mediator of RNA polymerase II transcription subunit 19	1064.03	1130.96	1060.99	465.11	502.12	485.77	-1.16
KLMA_70149	RPL32	60S ribosomal protein L32	9382.31	8012.89	9738.92	3617.39	6932.74	5312.32	-0.77
KLMA_70150	MAP2	methionine aminopeptidase 2	1041.70	1073.90	1068.30	945.35	962.11	969.80	-0.15
KLMA_70151		37S ribosomal protein MRP21	190.47	181.22	170.54	86.63	149.12	121.01	-0.60
KLMA_70152	AVT6	vacuolar amino acid transporter 6	767.75	882.62	863.65	549.21	533.29	607.64	-0.57
KLMA_70153	SHO1	protein SSU81	517.32	446.34	481.16	507.16	464.20	706.18	0.22
KLMA_70154	TEL1	serine/threonine-protein kinase TEL1	377.41	453.05	459.23	1275.04	676.51	843.61	1.12
KLMA_70155	RPL23B	60S ribosomal protein L23	14678.97	11793.93	14492.05	5054.76	10032.22	7500.86	-0.86
KLMA_70156		conserved hypothetical protein	18.81	70.47	57.25	146.34	91.83	121.87	1.30
KLMA_70157		hypothetical protein	21.16	34.68	18.27	73.17	32.86	52.73	1.09
KLMA_70158	PFS2	polyadenylation factor subunit 2	246.90	288.61	283.82	230.45	287.28	332.78	0.05
KLMA_70159	PFK27	6-phosphofructo-2-kinase 2	783.04	930.72	894.10	472.67	657.97	515.15	-0.66
KLMA_70160	MED7	mediator of RNA polymerase II transcription subunit 7	296.28	332.24	321.59	203.54	160.07	185.84	-0.79
KLMA_70161	HRT1	RING-box protein HRT1	114.05	142.07	162.01	196.81	187.03	162.50	0.39
KLMA_70162	PHA2	putative prephenate dehydratase	355.07	277.43	292.35	502.95	444.83	516.88	0.66
KLMA_70163	ATP11	protein ATP11	415.03	505.63	400.76	395.30	525.71	501.33	0.11
KLMA_70164		protein DAL82	110.52	76.07	109.63	224.56	163.44	161.63	0.89
KLMA_70165	EMW1	uncharacterized protein YNL313C	845.35	756.21	775.95	418.85	396.81	420.08	-0.94
KLMA_70166	ALR1	magnesium transporter ALR1	1286.25	981.06	1036.63	677.89	699.26	845.34	-0.57
KLMA_70167	TDA2	uncharacterized protein YER071C	95.23	107.39	82.83	195.97	163.44	186.70	0.93
KLMA_70168	SEC6	exocyst complex component SEC6	532.61	643.22	582.26	600.52	765.81	717.41	0.24

Locus_tag	UniProt_gene	Product	Unique exon reads						log2 Fold Change
			KmWT.1	KmWT.2	KmWT.3	Kmmig1.1	Kmmig1.2	Kmmig1.3	
KLMA_70169		uncharacterized protein YIL067C	431.49	293.09	404.42	443.24	459.99	483.17	0.30
KLMA_70170	RNR1	ribonucleoside-diphosphate reductase large chain 1	9207.13	7189.57	8714.48	2377.67	2575.45	3259.48	-1.61
KLMA_70171	FIS1	mitochondria fission 1 protein	59.96	43.63	48.73	71.49	66.56	85.57	0.55
KLMA_70172	SEE1	uncharacterized methyltransferase YIL064W	211.63	157.73	182.72	389.41	369.01	356.98	1.02
KLMA_70173	YRB2	ran-specific GTPase-activating protein 2	343.31	346.78	266.77	522.30	511.38	513.43	0.69
KLMA_70174	ARC15	actin-related protein 2/3 complex subunit 5	532.61	536.95	455.58	692.19	910.72	796.07	0.65
KLMA_70175	ARG5,6	protein ARG5	3517.78	1975.54	3504.55	1554.28	1779.31	1955.17	-0.77
KLMA_70176	SNP1	U1 small nuclear ribonucleoprotein 70 kDa homolog	114.05	129.76	142.52	66.44	89.30	88.16	-0.66
KLMA_70177	MOT2	general negative regulator of transcription subunit 4	559.65	630.92	582.26	343.99	413.66	451.19	-0.55
KLMA_70178	RGI1	uncharacterized protein YIL057C	6953.26	6067.56	6521.85	3119.49	4054.84	3141.93	-0.92
KLMA_70179	ICL1	isocitrate lyase	16.46	71.59	12.18	195.13	102.78	135.70	2.11
KLMA_70180	VHR1	transcription factor VHR1	846.52	804.31	883.14	381.00	329.41	361.30	-1.24
KLMA_70181		hypothetical protein	279.82	434.04	271.64	343.15	275.49	290.42	-0.12
KLMA_70182	THO1	protein THO1	162.25	115.22	154.70	53.83	87.62	88.16	-0.91
KLMA_70183	RHR2	(DL)-glycerol-3-phosphatase 1	11558.59	7362.96	10639.11	1633.34	1697.59	1726.11	-2.55
KLMA_70184	CEM1	3-oxoacyl-[acyl-carrier-protein] synthase homolog	258.66	225.97	220.48	241.38	206.41	183.24	-0.16
KLMA_70185	DFG10	protein DFG10	190.47	143.19	155.92	177.46	205.56	242.88	0.35
KLMA_70186	FCY2	purine-cytosine permease FCY2	13094.09	10028.70	13309.25	3693.93	3406.14	2532.56	-1.92
KLMA_70187	PCL6	PHO85 cyclin-6	984.08	949.73	1086.57	462.58	420.40	448.60	-1.18
KLMA_70188	PET117	protein PET117	79.95	83.90	64.56	21.87	53.08	39.76	-0.99

Locus_tag	UniProt_gene	Product	Unique exon reads						$\log_2$ Fold Change
			KmWT.1	KmWT.2	KmWT.3	Kmmig1.1	Kmmig1.2	Kmmig1.3	
KLMA_70189	MMF1	protein MMF1	478.52	356.85	367.87	856.20	913.25	823.73	1.11
KLMA_70190	RPL34B	60S ribosomal protein L34-B	12786.05	10774.84	12243.39	4937.86	8740.70	6621.81	-0.82
KLMA_70191	NEO1	probable phospholipid-transporting ATPase NEO1	773.63	738.31	784.47	572.76	605.74	705.31	-0.29
KLMA_70192	SOL1	6-phosphogluconolactonase-like protein 1	1223.93	1047.06	1132.86	1471.01	1475.18	1413.22	0.36
KLMA_70193	ABZ1	aminodeoxychorismate synthase	224.56	218.14	260.68	184.19	224.10	197.07	-0.22
KLMA_70194	HUB1	ubiquitin-like modifier HUB1	14.11	7.83	35.33	31.96	26.12	15.56	0.37
KLMA_70195	PPG1	serine/threonine-protein phosphatase PP2A-like PPG1	296.28	265.12	238.75	334.74	346.26	384.64	0.41
KLMA_70196	SSK2	MAP kinase kinase kinase SSK2	504.39	530.24	515.27	529.87	442.30	458.11	-0.12
KLMA_70197	RSA4	WD repeat-containing protein YCR072C	1113.41	945.26	1213.25	416.32	573.73	534.17	-1.10
KLMA_70198	IMG2	54S ribosomal protein IMG2	372.71	337.83	367.87	210.26	379.96	309.44	-0.26
KLMA_70199	ALG12	dolichyl-P-Man:Man(7)GlcNAc(2)-PP-dolichyl-alpha-1	1366.20	1333.43	1344.81	2751.94	2930.98	2470.32	1.01
KLMA_70200		uncharacterized protein YNR029C	202.23	276.31	231.44	571.92	556.03	485.77	1.18
KLMA_70201	CPR4	peptidyl-prolyl cis-trans isomerase CPR4	1455.55	1153.33	1391.10	1022.73	1410.31	1270.60	-0.11
KLMA_70202	TOM20	mitochondrial import receptor subunit TOM20	1479.07	1158.92	1402.06	487.81	866.91	716.55	-0.96
KLMA_70203		hypothetical protein	600.80	605.19	516.49	306.99	385.01	367.35	-0.70
KLMA_70204	SYT1	conserved hypothetical protein	799.50	802.07	766.20	858.72	744.75	728.65	-0.02
KLMA_70205	RDS3	pre-mRNA-splicing factor RDS3	30.57	24.61	57.25	45.42	38.75	32.85	0.06
KLMA_70206	ASR1	alcohol-sensitive RING finger protein 1	82.30	120.81	92.58	201.01	165.13	179.79	0.88

Locus_tag	UniProt_gene	Product	Unique exon reads						log2 Fold Change
			KmWT.1	KmWT.2	KmWT.3	Kmmig1.1	Kmmig1.2	Kmmig1.3	
KLMA_70207	TWF1	twinfilin-1	339.79	304.27	246.06	392.77	414.50	483.17	0.54
KLMA_70208		hypothetical protein	379.76	287.49	436.09	571.92	248.53	254.98	-0.04
KLMA_70209		uncharacterized PH domain-containing protein YPR091C	123.45	191.29	118.16	518.09	655.45	660.37	2.08
KLMA_70210	MEC1	serine/threonine-protein kinase MEC1	364.48	379.22	395.89	533.23	456.62	446.01	0.33
KLMA_70211	CKS1	cyclin-dependent kinases regulatory subunit	58.79	50.34	47.51	45.42	32.86	41.49	-0.39
KLMA_70212	HSL7	protein arginine N-methyltransferase HSL7	479.70	459.77	537.19	177.46	260.33	281.78	-1.04
KLMA_70213		dilute domain-containing protein YPR089W	885.32	771.87	807.62	653.50	725.37	841.88	-0.15
KLMA_70214	AGP2	general amino acid permease AGP2	499.68	698.04	655.35	1997.51	1744.77	1658.69	1.54
KLMA_70215	RVB2	ruvB-like helicase 2	1240.39	1165.63	1290.00	548.37	870.28	773.60	-0.75
KLMA_70216	VMA11	v-type proton ATPase 16 kDa proteolipid subunit 2	1089.90	816.61	1079.26	1138.79	1194.63	1099.46	0.20
KLMA_70217	NSL1	kinetochore-associated protein NSL1	49.38	54.81	48.73	50.46	38.75	45.81	-0.18
KLMA_70218	SSO1	protein SSO2	1994.04	2144.45	1890.53	1369.24	1580.49	1304.31	-0.50
KLMA_70219	FAS2	fatty acid synthase subunit alpha	12060.62	17257.42	14260.60	3366.76	5061.60	5379.74	-1.66
KLMA_70220	USV1	conserved hypothetical protein	310.39	205.83	226.57	634.16	413.66	439.09	1.00
KLMA_70221		hypothetical protein	717.19	745.02	743.06	848.63	570.36	586.03	-0.14
KLMA_70222	CET1	mRNA-capping enzyme subunit beta	470.29	460.88	430.00	476.88	387.54	439.96	-0.06
KLMA_70223	ALG5	dolichyl-phosphate beta-glucosyltransferase	645.47	579.46	626.12	431.46	572.88	618.88	-0.19
KLMA_70224	SPT21	protein SPT21	315.10	417.26	333.77	158.12	151.65	202.26	-1.06
KLMA_70225	NEW1	[NU+] prion formation protein 1	3582.44	3600.94	3904.09	1263.27	1305.00	1527.31	-1.44
KLMA_70226		uncharacterized	744.24	700.28	672.41	926.01	1117.97	1059.70	0.55

Locus_tag	UniProt_gene	Product	Unique exon reads						log2 Fold Change
			KmWT.1	KmWT.2	KmWT.3	Kmmig1.1	Kmmig1.2	Kmmig1.3	
		protein YMR178W							
KLMA_70227		UPF0368 protein YPL225W	748.94	545.90	674.84	588.74	935.15	701.86	0.18
KLMA_70228	MMT1	mitochondrial metal transporter 1	520.85	644.34	581.05	901.61	881.23	793.48	0.56
KLMA_70229	ECM5	protein ECM5	311.57	416.14	350.82	547.53	395.12	439.09	0.36
KLMA_70230		hypothetical protein	102.29	147.66	86.49	9755.44	5266.33	7335.77	6.05
KLMA_70231	TUB4	tubulin gamma chain	412.68	489.97	430.00	847.79	626.80	569.61	0.62
KLMA_70232	LDB17	protein LDB17	317.45	382.58	417.82	339.79	394.28	411.43	0.04
KLMA_70233		uncharacterized protein YLR211C	190.47	201.36	148.61	418.01	393.44	348.33	1.10
KLMA_70234	RPN5	26S proteasome regulatory subunit RPN5	1006.42	1229.40	1019.57	1075.71	1226.65	1086.49	0.06
KLMA_70235	NOP14	probable nucleolar complex protein 14	907.66	853.53	1089.00	364.18	530.76	499.60	-1.03
KLMA_70236	ATG9	autophagy-related protein 9	189.29	315.46	246.06	650.98	483.58	573.93	1.19
KLMA_70237	RPC53	DNA-directed RNA polymerase III subunit RPC4	324.50	392.65	405.64	225.40	272.12	233.38	-0.62
KLMA_70238	SAS10	something about silencing protein 10	1060.51	845.70	1148.69	485.29	549.30	561.83	-0.94
KLMA_70239	MSH5	mutS protein homolog 5	49.38	55.93	57.25	121.95	89.30	89.03	0.89
KLMA_70240	CLB4	G2/mitotic-specific cyclin-4	640.77	692.44	556.68	285.96	402.70	383.77	-0.82
KLMA_70241	MNN5	alpha-1 2-mannosyltransferase	1403.82	1238.35	1099.97	744.34	1078.37	1132.30	-0.34
KLMA_70242		conserved hypothetical protein	30.57	88.37	42.63	213.63	220.73	241.15	2.06
KLMA_70243		uncharacterized protein YDR391C	39.97	55.93	48.73	47.10	58.13	56.18	0.16
KLMA_70244		WD repeat-containing protein KLLA0C16533g	152.84	156.61	126.69	220.36	231.68	200.53	0.58
KLMA_70245		uncharacterized protein YDL157C	125.80	77.19	123.03	54.67	71.61	56.18	-0.84
KLMA_70246	STE7	serine/threonine-protein kinase STE7	380.94	460.88	353.26	379.32	266.22	349.20	-0.27
KLMA_70248	DHH1	ATP-dependent RNA helicase DHH1	1727.15	1843.54	1681.01	990.77	1043.83	998.33	-0.79

Locus_tag	UniProt_gene	Product	Unique exon reads						log$_2$ Fold Change
			KmWT.1	KmWT.2	KmWT.3	Kmmig1.1	Kmmig1.2	Kmmig1.3	
KLMA_70249	PNP1	purine nucleoside phosphorylase	933.53	928.48	939.18	1285.98	1766.67	1772.79	0.78
KLMA_70250	SEC13	protein transport protein SEC13	1327.40	1258.48	1265.63	541.64	877.02	869.54	-0.75
KLMA_70251	HRD3	ERAD-associated E3 ubiquitin-protein ligase component HRD3	459.71	523.53	356.91	612.29	523.18	518.61	0.30
KLMA_70252		conserved hypothetical protein	206.93	228.20	208.30	375.11	310.87	331.91	0.66
KLMA_70253		conserved hypothetical protein	10.58	6.71	23.14	14.30	16.01	12.10	0.08
KLMA_70254	ENT2	epsin-2	870.04	992.24	896.54	1115.24	900.61	952.52	0.11
KLMA_70255	NOP10	H/ACA ribonucleoprotein complex subunit 3	42.33	17.90	19.49	1.68	22.75	17.29	-0.93
KLMA_70256	DAD4	DASH complex subunit DAD4	76.42	30.20	57.25	72.33	67.40	62.23	0.30
KLMA_70257	OSH3	oxysterol-binding protein homolog 3	1567.25	1523.60	1490.99	2114.42	1241.81	1376.92	0.05
KLMA_70258	PHO84	inorganic phosphate transporter	509.09	340.07	433.65	31067.02	8791.25	12907.39	5.36
KLMA_70260	TUB1	tubulin alpha-1 chain	2773.54	2054.96	2571.46	1479.42	3022.81	2622.45	-0.05
KLMA_70261	ALO1	D-arabinono-1	2035.19	1638.82	1896.62	1240.56	1726.24	1693.27	-0.26
KLMA_70262	PGA3	uncharacterized oxidoreductase YML125C	650.18	533.60	572.52	597.15	799.51	708.77	0.26
KLMA_70263	UFO1	ubiquitin ligase complex F-box protein UFO1	611.38	676.78	583.48	516.41	485.27	514.29	-0.30
KLMA_70264	ORC4	origin recognition complex subunit 4	268.07	298.68	297.22	231.29	325.20	312.03	0.01
KLMA_70265	TIF3	eukaryotic translation initiation factor 4B	1584.88	1530.31	1667.61	463.42	803.72	758.04	-1.24
KLMA_70266	FMP27	protein FMP27	672.52	870.31	761.33	2446.64	1251.92	1425.32	1.15
KLMA_70267		hypothetical protein	265.71	248.34	241.19	340.63	239.26	274.86	0.18
KLMA_70268	RHO1	GTP-binding protein Rho1	5957.42	4752.03	5493.75	5194.38	5310.13	5133.40	-0.05
KLMA_70269		40S ribosomal protein MRP2	189.29	152.14	219.26	137.93	235.89	167.68	-0.05

Locus_tag	UniProt_gene	Product	Unique exon reads						log$_2$ Fold Change
			KmWT.1	KmWT.2	KmWT.3	Kmmig1.1	Kmmig1.2	Kmmig1.3	
KLMA_70270		uncharacterized protein C1F7.10	64.67	46.98	69.43	143.82	170.18	150.40	1.36
KLMA_70271	MET16	phosphoadenosine phosphosulfate reductase	2671.26	1531.43	2333.93	638.36	905.66	1012.16	-1.35
KLMA_70272	NUT2	mediator of RNA polymerase II transcription subunit 10	59.96	62.64	40.20	37.01	58.97	57.05	-0.09
KLMA_70273	JIP5	WD repeat-containing protein JIP5	660.76	566.04	719.91	339.79	363.11	383.77	-0.84
KLMA_70275		hypothetical protein	353.89	419.49	405.64	525.66	379.96	400.20	0.15
KLMA_70277		PWWP domain-containing protein YLR455W	377.41	378.10	386.15	426.42	350.47	389.82	0.03
KLMA_70278		pyridoxamine 5'-phosphate oxidase homolog	566.70	470.95	442.18	350.72	390.07	312.90	-0.49
KLMA_70279	VPS4	vacuolar protein sorting-associated protein 4	768.93	901.63	685.81	660.23	755.70	688.03	-0.16
KLMA_70281	ASN1	asparagine synthetase [glutamine-hydrolyzing] 1	8883.80	5608.91	8195.55	4788.15	6601.65	6785.18	-0.32
KLMA_70282	NOC4	nucleolar complex protein 4	1094.60	1086.21	1338.72	456.69	552.67	537.63	-1.19
KLMA_70283	PPT1	serine/threonine-protein phosphatase T	545.54	482.14	548.16	318.76	500.43	500.46	-0.26
KLMA_70284	RRP15	ribosomal RNA-processing protein 15	506.74	415.02	506.74	211.11	272.12	280.05	-0.90
KLMA_70285	KAR3	kinesin-like protein KAR3	182.24	144.31	171.76	436.51	288.13	266.22	0.99
KLMA_70286	TAZ1	lysophosphatidylcholine acyltransferase	666.64	711.46	729.66	596.31	722.00	708.77	-0.06
KLMA_70287	LOA1	vacuolar protein sorting-associated protein 66	485.58	410.54	372.75	328.85	375.74	383.77	-0.22
KLMA_70288		uncharacterized protein YGR122W	701.91	737.19	688.24	343.99	482.74	425.26	-0.76
KLMA_70289	MEP3	ammonium	576.11	486.61	616.37	405.39	402.70	464.16	-0.40

Locus_tag	UniProt_gene	Product	Unique exon reads						log2 Fold Change
			KmWT.1	KmWT.2	KmWT.3	Kmmig1.1	Kmmig1.2	Kmmig1.3	
		transporter MEP3							
KLMA_70290	RRP9	ribosomal RNA-processing protein 9	734.83	628.68	851.47	287.64	375.74	373.40	-1.09
KLMA_70291	CTF4	DNA polymerase alpha-binding protein	546.71	510.10	476.29	282.60	315.09	385.50	-0.64
KLMA_70292		conserved oligomeric Golgi complex subunit 2	126.98	128.64	130.34	217.83	212.30	172.87	0.64
KLMA_70293	NUP57	nucleoporin NUP57	683.10	647.70	708.95	333.90	552.67	471.94	-0.59
KLMA_70294		hypothetical protein	232.79	307.63	232.66	274.19	228.31	292.15	0.04
KLMA_70295		mitochondrial import receptor subunit TOM5	7.05	13.42	6.09	8.41	12.64	7.78	0.11
KLMA_70296	IWS1	transcription factor IWS1	737.18	718.17	610.28	466.79	673.14	706.18	-0.16
KLMA_70297	rps12	40S ribosomal protein S23	20418.88	16257.35	19086.82	8050.61	15647.33	12129.47	-0.64
KLMA_70298		uncharacterized protein YGR117C	162.25	184.58	160.79	251.48	288.97	280.91	0.69
KLMA_70299	NAT3	N-terminal acetyltransferase B complex catalytic subunit NAT3	194.00	203.59	196.12	263.25	278.86	267.95	0.45
KLMA_70300	SCD6	protein SCD6	851.23	879.26	817.36	484.45	464.20	490.09	-0.82
KLMA_70301	ANT1	peroxisomal adenine nucleotide transporter 1	166.95	258.41	176.63	854.52	992.44	1071.80	2.28
KLMA_70302	YLH47	LETM1 domain-containing protein YLH47	210.46	341.19	294.79	279.23	256.11	268.81	-0.07
KLMA_70303		probable 6-phosphofructo-2-kinase/fructose-2,6-biphosphatase	124.63	64.88	107.20	341.47	338.68	338.83	1.78
KLMA_70304	SPT6	transcription elongation factor SPT6	2220.95	2349.17	2247.44	1470.17	1878.72	1678.57	-0.44
KLMA_70305	CTR1	copper transport protein CTR1	505.56	486.61	476.29	444.08	406.07	293.88	-0.36
KLMA_70306	DAM1	DASH complex subunit DAM1	168.13	155.49	191.25	123.64	162.60	92.49	-0.44

Locus_tag	UniProt_gene	Product	Unique exon reads						log $_2$ Fold Change
			KmWT.1	KmWT.2	KmWT.3	Kmmig1.1	Kmmig1.2	Kmmig1.3	
KLMA_70307	SHY1	cytochrome oxidase assembly protein SHY1	1154.57	1176.82	1120.68	583.69	649.55	527.26	-0.97
KLMA_70308	MRPS5	37S ribosomal protein S5	691.33	827.80	685.81	317.92	488.64	454.65	-0.81
KLMA_70309		conserved hypothetical protein	631.37	851.29	641.95	571.92	342.05	324.13	-0.78
KLMA_70310	AFG3	mitochondrial respiratory chain complexes assembly protein AFG3	1499.05	2006.86	1467.84	1407.09	1356.39	1292.21	-0.29
KLMA_70311	BIM1	microtubule integrity protein mal3	238.67	246.10	199.77	132.89	162.60	177.19	-0.54
KLMA_70312	FAA2	long-chain-fatty-acid--CoA ligase 2	238.67	321.05	263.12	451.65	467.57	548.86	0.83
KLMA_70313	HEM14	protoporphyrinogen oxidase	885.32	776.34	841.73	340.63	359.74	358.71	-1.24
KLMA_70314	PRP22	pre-mRNA-splicing factor ATP-dependent RNA helicase PRP22	330.38	449.70	364.22	536.60	371.53	382.04	0.17
KLMA_70315	PRE1	proteasome component C11	502.04	535.83	498.21	645.09	736.33	739.89	0.47
KLMA_70316	PRI1	DNA primase small subunit	231.62	269.59	239.97	198.49	194.61	170.28	-0.40
KLMA_70317		uncharacterized glycosyl hydrolase YIR007W	151.67	255.05	138.87	831.81	603.21	642.21	1.93
KLMA_70318	MDM38	mitochondrial distribution and morphology protein 38	699.56	1142.14	740.62	897.41	679.88	707.04	-0.18
KLMA_70320	RPR2	ribonuclease P protein subunit RPR2	459.71	501.16	447.05	966.38	609.95	616.28	0.64
KLMA_70321		mitochondrial import protein 1	716.02	637.63	695.55	553.42	411.13	431.31	-0.55
KLMA_70322	PRE6	proteasome component PRE6	1687.17	1679.09	1532.40	1599.69	1787.74	1745.99	0.07
KLMA_70323		uncharacterized protein YOL036W	949.99	949.73	886.80	430.62	438.93	494.41	-1.03
KLMA_70324		transcriptional activator of sulfur metabolism	671.34	661.12	744.28	396.98	271.28	338.83	-1.04

Locus_tag	UniProt_gene	Product	Unique exon reads						log2 Fold Change
			KmWT.1	KmWT.2	KmWT.3	Kmmig1.1	Kmmig1.2	Kmmig1.3	
KLMA_70325	SLT2	mitogen-activated protein kinase SLT2/MPK1	1076.97	1530.31	855.12	774.62	641.13	914.49	-0.57
KLMA_70326	VPS15	serine/threonine-protein kinase VPS15	511.44	645.46	587.14	667.80	582.99	542.81	0.04
KLMA_70327	SMK1	sporulation-specific mitogen-activated protein kinase SMK1	90.53	143.19	125.47	206.90	167.65	190.16	0.65
KLMA_70328		uncharacterized protein YBR096W	237.50	270.71	239.97	370.07	318.46	258.44	0.34
KLMA_70329	RXT2	transcriptional regulatory protein RXT2	303.34	333.36	280.17	352.40	414.50	355.25	0.29
KLMA_70330	PBY1	TTL domain-containing protein YBR094W	832.42	807.67	790.56	783.87	771.71	794.34	-0.05
KLMA_70331	TIM12	mitochondrial import inner membrane translocase subunit TIM12	64.67	87.25	88.92	144.66	108.68	108.91	0.59
KLMA_70332	NHP6	non-histone chromosomal protein 6	1700.10	1694.76	1872.26	1154.77	1446.53	1133.17	-0.50
KLMA_70334	POL30	proliferating cell nuclear antigen	670.17	875.90	650.48	383.52	426.29	406.25	-0.85
KLMA_70335	MAK3	N-alpha-acetyltransferase 30	98.76	72.71	88.92	95.04	105.31	116.69	0.28
KLMA_70337	ATG11	autophagy-related protein 11	238.67	340.07	261.90	658.55	378.27	433.04	0.81
KLMA_70338	TAH18	probable NADPH reductase TAH18	330.38	372.51	359.35	491.18	529.92	431.31	0.45
KLMA_70339	pheS	phenylalanyl-tRNA synthetase	558.47	597.36	527.45	382.68	377.43	372.54	-0.57
KLMA_70340		54S ribosomal protein L16	538.48	429.56	465.32	487.81	667.24	632.71	0.32
KLMA_70341	RFC5	replication factor C subunit 5	319.80	387.05	309.40	234.66	273.81	272.27	-0.38
KLMA_70342	APL3	AP-2 complex subunit alpha	779.51	814.38	889.23	893.20	852.59	803.85	0.04
KLMA_70343		UPF0001 protein YBL036C	630.19	670.07	585.92	306.99	411.97	390.69	-0.77
KLMA_70344	IST2	hypothetical protein	3173.29	3571.85	3187.84	3086.69	3299.14	2946.58	-0.09

Locus_tag	UniProt_gene	Product	Unique exon reads						$\log_2$ Fold Change
			KmWT.1	KmWT.2	KmWT.3	Kmmig1.1	Kmmig1.2	Kmmig1.3	
KLMA_70346	POL12	DNA polymerase alpha subunit B	325.68	268.48	293.57	197.65	264.54	283.51	-0.25
KLMA_70347	STU1	protein STU1	399.75	488.85	451.92	320.44	291.50	284.37	-0.58
KLMA_70348		uncharacterized protein YBR085C-A	65.84	82.78	45.07	280.07	194.61	227.32	1.85
KLMA_70349	RIB1	GTP cyclohydrolase-2	579.63	346.78	542.07	809.10	684.09	974.99	0.75
KLMA_70351	HEK2	KH domain-containing protein YBL032W	452.66	349.02	347.17	267.46	358.90	406.25	-0.15
KLMA_70352	SHE1	mitotic spindle-associated protein SHE1	139.91	165.56	190.03	125.32	113.73	152.13	-0.34
KLMA_70354	AAC	ADP,ATP carrier protein	16451.97	14599.51	16813.80	20360.35	25870.79	22403.18	0.52
KLMA_70355		uncharacterized protein YBL029C-A	132.86	134.24	129.12	89.15	52.23	55.32	-1.01
KLMA_70356		hypothetical protein	172.83	153.26	159.57	121.95	56.45	79.52	-0.91
KLMA_70357		UPF0642 protein YBL028C	269.24	237.15	265.55	107.66	111.21	130.52	-1.14
KLMA_70358	RPL19B	60S ribosomal protein L19	19702.86	17764.17	18960.14	6306.26	11610.18	9549.38	-1.04
KLMA_70359	MIS1	c-1-tetrahydrofolate synthase	1504.93	1427.40	1626.20	488.66	550.98	592.95	-1.48
KLMA_70361	TEC1	conserved hypothetical protein	48.20	54.81	40.20	250.64	179.45	261.03	2.27
KLMA_70362	MAK21	ribosome biogenesis protein MAK21	1317.99	1354.69	1478.81	400.34	443.14	421.80	-1.71
KLMA_70364		conserved hypothetical protein	69.37	73.83	74.31	84.11	76.67	82.98	0.16
KLMA_70365	UBC4	ubiquitin-conjugating enzyme E2 4	2392.61	1953.16	2213.34	1639.22	2051.43	2168.66	-0.16
KLMA_70366	SPT7	transcriptional activator SPT7	630.19	652.17	560.34	576.13	573.73	609.37	-0.07
KLMA_70367	YOS9	protein OS-9 homolog	415.03	365.80	380.06	379.32	492.01	486.63	0.23
KLMA_70368		uncharacterized protein YDR056C	431.49	360.21	416.60	229.61	294.87	239.43	-0.66
KLMA_70369	HTD2	hydroxyacyl-thioester dehydratase type 2	163.43	189.05	168.10	104.29	122.16	137.43	-0.52
KLMA_70370	IPK1	inositol-pentakisphosphate 2-	310.39	234.92	333.77	193.44	171.87	165.96	-0.73

Locus_tag	UniProt_gene	Product	Unique exon reads						log_2 Fold Change
			KmWT.1	KmWT.2	KmWT.3	Kmmig1.1	Kmmig1.2	Kmmig1.3	
		kinase							
KLMA_70371	DYS1	deoxyhypusine synthase	4373.71	2870.46	4375.51	1241.40	1855.98	1620.66	-1.30
KLMA_70372	OMS1	methyltransferase OMS1	522.02	533.60	521.36	234.66	278.02	311.17	-0.94
KLMA_70373	RRP4	exosome complex component RRP4	483.22	451.93	509.18	311.19	453.25	410.57	-0.30
KLMA_70374	TRM5	tRNA (guanine-N(1)-)-methyltransferase	366.83	343.43	380.06	260.73	212.30	216.09	-0.66
KLMA_70375	PCL5	PHO85 cyclin-5	1856.48	1192.48	1607.93	1679.59	1365.66	1368.27	-0.08
KLMA_70376	ARL3	ADP-ribosylation factor-like protein 3	177.54	155.49	177.85	211.95	187.03	226.46	0.29
KLMA_70377	PHO8	repressible alkaline phosphatase	1830.61	1538.14	1657.87	1326.35	1564.48	1473.72	-0.20
KLMA_70378	MNN9	mannan polymerase complexes subunit MNN9	792.44	931.84	839.29	925.16	1036.25	1195.40	0.30
KLMA_70379		nucleus export protein BRR6	144.61	204.71	198.55	228.77	132.27	132.25	-0.15
KLMA_70380	RAI1	protein RAI1	276.30	259.53	313.06	235.50	282.23	271.41	-0.10
KLMA_70381	PTR2	peptide transporter PTR2	34.10	42.51	67.00	356.61	197.98	203.12	2.40
KLMA_70382	PTR2	peptide transporter PTR2	19.99	62.64	59.69	141.30	65.71	69.15	0.96
KLMA_70383	PTR2	peptide transporter PTR2	2733.57	3072.93	1992.85	1121.97	547.61	787.43	-1.67
KLMA_70384	UGA4	GABA-specific permease	426.79	502.27	370.31	142.14	72.45	107.18	-2.01
KLMA_70385	PAN6	pantoate--beta-alanine ligase	339.79	241.63	356.91	241.38	286.44	291.29	-0.20
KLMA_70386	NDC80	probable kinetochore protein NDC80	587.86	751.73	710.17	645.93	609.11	560.97	-0.17
KLMA_70387	SSL2	DNA repair helicase RAD25	1698.93	1953.16	1733.39	1809.12	1330.27	1242.94	-0.30
KLMA_70390	SRP40	suppressor protein SRP40	645.47	549.26	620.03	138.77	188.71	185.84	-1.82
KLMA_70391		hypothetical protein	1029.94	1011.26	1130.42	1222.90	1327.74	1144.40	0.22
KLMA_70392	LPX1	peroxisomal membrane protein LPX1	99.94	130.88	152.27	666.12	506.33	662.96	2.26
KLMA_70393		hypothetical protein	208.10	204.71	158.36	273.34	232.52	362.16	0.60

Locus_tag	UniProt_gene	Product	Unique exon reads						log2 Fold Change
			KmWT.1	KmWT.2	KmWT.3	Kmmig1.1	Kmmig1.2	Kmmig1.3	
KLMA_70394	PXL1	paxillin-like protein 1	54.08	99.56	38.98	26.07	37.07	52.73	-0.74
KLMA_70395	PUS4	tRNA pseudouridine synthase 4	364.48	321.05	366.66	147.19	141.54	121.01	-1.36
KLMA_70396	MID1	stretch-activated cation channel MID1	778.33	629.80	655.35	932.73	850.90	1085.63	0.48
KLMA_70397	RFC3	replication factor C subunit 3	51.73	58.17	52.38	48.78	54.76	60.50	0.01
KLMA_70398	PCL1	PHO85 cyclin-1	2110.43	2399.51	2290.08	1081.60	842.48	847.07	-1.30
KLMA_70399	CAF40	protein CAF40	618.43	463.12	537.19	411.28	317.61	408.84	-0.51
KLMA_70400	SEC21	coatomer subunit gamma	4717.02	4169.21	4228.12	4016.06	5244.42	5178.34	0.14
KLMA_70401	NPC2	phosphatidylglycerol /phosphatidylinositol transfer protein	116.40	109.63	148.61	91.68	127.21	159.91	0.02
KLMA_70402	EMI5	succinate dehydrogenase assembly factor 2	325.68	205.83	277.73	358.29	405.23	482.31	0.62
KLMA_70403	MRP10	40S ribosomal protein MRP10	111.69	107.39	127.90	117.75	112.05	87.30	-0.13
KLMA_70404	FAD1	FAD synthetase	241.02	305.39	272.86	940.30	476.00	806.44	1.44
KLMA_70405	MTF2	mitochondrial transcription factor 2	457.36	390.41	442.18	333.06	304.13	313.76	-0.44
KLMA_70406	NBA1	protein NBA1	70.54	51.46	77.96	80.74	145.75	150.40	0.92
KLMA_70407	AGC1	mitochondrial aspartate-glutamate transporter AGC1	509.09	609.66	427.56	650.14	675.67	731.24	0.41
KLMA_70408		zinc finger protein YPR022C	1382.66	1313.30	1506.82	549.21	406.92	492.68	-1.54
KLMA_70409	EAF3	chromatin modification-related protein EAF3	166.95	163.32	162.01	70.65	108.68	130.52	-0.67
KLMA_70410	YCK3	casein kinase I homolog 3	873.57	879.26	760.11	740.13	796.98	755.45	-0.13
KLMA_70411	DSE1	protein DSE1	820.66	935.19	775.95	1062.26	942.73	854.85	0.18
KLMA_70412	RSP5	e3 ubiquitin-protein ligase RSP5	3799.95	3721.75	3438.77	2141.34	2574.61	2542.06	-0.59
KLMA_70413	MPT5	multicopy suppressor of pop two	1196.89	1241.70	1191.33	643.41	631.86	739.02	-0.85
KLMA_70414	NSA2	ribosome biogenesis protein NSA2	845.35	751.73	821.02	398.66	409.44	488.36	-0.90

Locus_tag	UniProt_gene	Product	Unique exon reads						log2 Fold Change
			KmWT.1	KmWT.2	KmWT.3	Kmmig1.1	Kmmig1.2	Kmmig1.3	
KLMA_70415	LCP5	U3 small nucleolar ribonucleoprotein protein LCP5	198.70	161.09	136.43	99.24	85.09	99.40	-0.81
KLMA_70416	VFA1	uncharacterized protein YER128W	51.73	44.75	46.29	116.07	78.35	121.01	1.14
KLMA_70417	NOP9	pumilio domain-containing protein YJL010C	912.37	760.68	900.20	312.03	288.97	353.52	-1.43
KLMA_70418	MRP8	uncharacterized protein MRP8	136.38	195.76	180.28	254.00	265.38	279.19	0.64
KLMA_70419	LTV1	protein LTV1	513.79	496.68	477.51	158.12	185.34	206.58	-1.44
KLMA_70420		conserved hypothetical membrane protein	289.23	280.78	287.48	273.34	231.68	262.76	-0.16
KLMA_70421	RPC25	DNA-directed RNA polymerase III subunit RPC8	22.34	41.39	25.58	20.19	22.75	12.97	-0.68
KLMA_70422	RPT1	26S protease regulatory subunit 7 homolog	630.19	619.73	553.03	664.44	534.13	589.49	-0.01
KLMA_70424	AVT3	vacuolar amino acid transporter 3	427.97	450.82	406.85	536.60	389.22	472.80	0.12
KLMA_70425	MBB1	uncharacterized protein MBB1	16.46	30.20	34.11	16.82	7.58	10.37	-1.21
KLMA_70426	FOX2	peroxisomal hydratase-dehydrogenase-epimerase	90.53	138.71	126.69	528.18	455.78	546.27	2.10
KLMA_70427	KTR1	alpha-1,2 mannosyltransferase KTR1	63.49	78.31	103.54	95.88	125.53	127.92	0.51
KLMA_70428		conserved hypothetical protein	96.41	40.27	90.14	219.52	168.50	191.89	1.36
KLMA_70429	ICL2	mitochondrial 2-methylisocitrate lyase	103.46	61.53	81.61	756.11	1535.84	1855.77	4.07
KLMA_70430		hypothetical protein	114.05	154.37	109.63	609.77	252.74	286.10	1.60
KLMA_70431		hypothetical protein	431.49	431.80	331.33	2717.46	1180.31	1708.83	2.23
KLMA_70432	YFH7	uncharacterized protein YFR007W	91.71	133.12	129.12	160.64	161.76	159.91	0.45
KLMA_70433		conserved hypothetical protein	189.29	163.32	157.14	407.91	246.85	251.53	0.83
KLMA_70434		uncharacterized	16.46	13.42	17.05	67.28	37.07	36.30	1.59

Locus_tag	UniProt_gene	Product	Unique exon reads						$\log_2$ Fold Change
			KmWT.1	KmWT.2	KmWT.3	Kmmig1.1	Kmmig1.2	Kmmig1.3	
		protein YDL144C							
KLMA_70435	AIM45	probable electron transfer flavoprotein subunit alpha	3384.92	2857.03	3040.44	2245.63	2753.22	2538.61	-0.30
KLMA_70436		putative sulfate transporter YPR003C	657.23	553.73	679.71	1059.73	1032.03	1160.83	0.78
KLMA_70438		uncharacterized peptidase YFR006W	592.57	585.05	553.03	666.96	517.28	618.88	0.06
KLMA_70439	SAD1	pre-mRNA-splicing factor SAD1	144.61	121.93	119.38	357.45	240.11	302.52	1.22
KLMA_70440	RPN11	26S proteasome regulatory subunit RPN11	831.24	904.99	723.57	1142.16	1071.63	1120.20	0.44
KLMA_70441		type 1 phosphatases regulator YPI1	91.71	136.48	70.65	185.87	187.03	114.09	0.70
KLMA_70442	NIC96	nucleoporin NIC96	1414.40	1334.55	1472.71	876.38	1101.12	995.74	-0.51
KLMA_70443	PDH1	probable 2-methylcitrate dehydratase	143.44	105.15	197.34	1785.57	1847.55	2260.28	3.73
KLMA_70444	CIT3	citrate synthase 3	17.64	22.37	17.05	890.68	666.40	928.32	5.44
KLMA_70445	LOC1	60S ribosomal subunit assembly/export protein LOC1	384.46	354.61	426.34	254.00	312.56	254.12	-0.51
KLMA_70446	HAT1	histone acetyltransferase type B catalytic subunit	513.79	464.24	503.09	608.93	619.22	616.28	0.32
KLMA_70447	SNF8	vacuolar-sorting protein SNF8	81.13	101.80	85.27	459.22	294.87	308.57	1.99
KLMA_70448	DEG1	tRNA pseudouridine synthase 3	452.66	473.19	411.73	310.35	298.24	343.15	-0.49
KLMA_70449	SPB4	ATP-dependent rRNA helicase SPB4	577.28	514.58	553.03	588.74	373.22	464.16	-0.21
KLMA_70450	ULA1	NEDD8-activating enzyme E1 regulatory subunit	238.67	239.39	252.15	627.43	455.78	478.85	1.10
KLMA_70451	LSP1	sphingolipid long chain base-responsive protein LSP1	1823.55	2485.64	1949.00	3054.73	3634.45	3809.21	0.75
KLMA_70453		ice-structuring	928.83	1070.55	926.99	1005.91	1243.50	982.77	0.14

Locus_tag	UniProt_gene	Product	Unique exon reads						log2 Fold Change
			KmWT.1	KmWT.2	KmWT.3	Kmmig1.1	Kmmig1.2	Kmmig1.3	
		glycoprotein							
KLMA_70455	ADY2	hypothetical protein	383.29	396.00	371.53	1054.69	1556.06	1223.06	1.74
KLMA_70456	FRE3	ferric reductase transmembrane component 4	621.96	488.85	565.21	814.14	518.12	490.95	0.12
KLMA_70457	yxeK	putative monooxygenase yxeK	447.95	163.32	328.89	327.17	248.53	255.85	-0.18
KLMA_70458	ywnB	uncharacterized protein ywnB	262.19	284.14	241.19	1082.44	893.03	783.10	1.81
KLMA_70459	MCH2	probable transporter MCH2	131.68	143.19	126.69	399.50	245.16	235.97	1.13
KLMA_70460		hypothetical protein	372.71	376.99	431.22	659.39	485.27	474.53	0.46
KLMA_70461		YKL222C	641.95	484.38	570.08	984.88	668.93	736.43	0.49
KLMA_70462	FCY2	purine-cytosine permease FCY2	19.99	11.19	12.18	415.48	147.43	176.33	4.09
KLMA_80001		hypothetical protein	438.55	365.80	430.00	252.32	222.41	185.84	-0.90
KLMA_80002	TY2B-C	transposon Ty2-LR1 Gag-Pol polyprotein	4.70	2.24	0.00	0.00	0.00	3.46	-1.02
KLMA_80003	TY2B-GR1	transposon Ty2-DR2 Gag-Pol polyprotein	0.00	2.24	13.40	22.71	6.74	3.46	1.08
KLMA_80004		putative uncharacterized oxidoreductase YGL039W	119.92	155.49	132.78	423.89	258.64	274.00	1.23
KLMA_80005	LAC12	high-affinity glucose transporter	68.19	63.76	43.85	479.40	171.87	203.99	2.28
KLMA_80006	UGA4	GABA-specific permease	212.81	189.05	248.50	116.07	104.47	95.94	-1.04
KLMA_80007	UGA4	GABA-specific permease	191.64	279.66	216.83	291.85	165.97	182.38	-0.10
KLMA_80008		conserved hypothetical protein	84.65	119.70	92.58	839.38	979.80	858.30	3.17
KLMA_80009		peroxisomal biogenesis factor 2	343.31	355.73	321.59	314.56	223.26	262.76	-0.35
KLMA_80010	NUC1	nuclease 1	741.88	544.78	638.30	506.32	480.21	422.67	-0.45
KLMA_80011	PHR1	deoxyribodipyrimidine photo-lyase	142.26	104.03	112.07	368.38	286.44	342.28	1.48
KLMA_80012		conserved hypothetical protein	652.53	627.56	641.95	891.52	690.83	669.01	0.23
KLMA_80013		uncharacterized	65.84	17.90	40.20	84.11	40.44	89.03	0.79

Locus_tag	UniProt_gene	Product	Unique exon reads						log2 Fold Change
			KmWT.1	KmWT.2	KmWT.3	Kmmig1.1	Kmmig1.2	Kmmig1.3	
		dipeptidase C965.12							
KLMA_80014	hyuA	conserved hypothetical protein	202.23	250.58	187.59	422.21	485.27	519.48	1.15
KLMA_80015	THI72	thiamine transporter THI72	3094.52	2511.37	3113.53	1571.94	1533.31	2207.56	-0.71
KLMA_80016	DIM1	dimethyladenosine transferase	1256.85	677.90	1118.24	597.99	427.98	564.42	-0.94
KLMA_80017		APC/C-CDH1 modulator 1	583.16	548.14	505.52	821.71	655.45	870.40	0.52
KLMA_80019	HSP31	probable chaperone protein HSP31	129.33	213.66	99.89	1067.30	217.36	229.92	1.77
KLMA_80020	KAR9	karyogamy protein KAR9	99.94	125.29	119.38	95.88	92.67	91.62	-0.30
KLMA_80021	MDL2	ATP-dependent permease MDL2	1105.18	959.80	975.72	894.05	821.42	891.15	-0.22
KLMA_80022		ATP synthase subunit epsilon	282.17	323.29	308.19	227.93	387.54	240.29	-0.09
KLMA_80023	POL5	DNA polymerase V	1128.70	985.53	1169.40	998.34	650.39	684.57	-0.49
KLMA_80024	HAT2	histone acetyltransferase type B subunit 2	418.56	428.44	412.94	205.22	356.37	350.06	-0.47
KLMA_80025	KEL3	kelch repeat-containing protein 3	446.78	413.90	453.14	294.37	331.09	363.03	-0.41
KLMA_80026	PCM1	phosphoacetylglucosamine mutase	391.52	409.43	310.62	229.61	309.19	425.26	-0.21
KLMA_80027	SOM1	protein SOM1	25.87	13.42	31.67	43.74	32.01	26.79	0.54
KLMA_80028	FUM1	fumarate hydratase	4570.06	4416.43	4626.44	4156.51	6011.92	5550.88	0.21
KLMA_80029	PRB1	proteinase YSCB	5186.14	6575.43	5124.66	11900.14	6652.20	8143.07	0.66
KLMA_80030	UTP23	rRNA-processing protein UTP23	266.89	283.02	254.59	112.70	159.23	165.09	-0.88
KLMA_80031	CIN8	kinesin-like protein CIN8	536.13	532.48	578.61	528.18	596.47	611.10	0.08
KLMA_80032	NPR2	nitrogen permease regulator 2	332.73	322.17	277.73	381.00	332.78	324.13	0.15
KLMA_80033	LIG4	DNA ligase 4	383.29	374.75	467.76	568.56	311.72	363.03	0.02
KLMA_80034	TSR3	UPF0293 protein YOR006C	358.60	272.95	300.88	418.85	285.60	331.91	0.15
KLMA_80035		UPF0507 protein KLLA0D01133g	465.59	448.58	460.45	859.56	500.43	615.42	0.52
KLMA_80036	SGT2	small glutamine-rich tetratricopeptide	1750.66	1994.55	1772.37	1738.47	1727.92	1636.22	-0.11

Locus_tag	UniProt_gene	Product	Unique exon reads						$\log_2$ Fold Change
			KmWT.1	KmWT.2	KmWT.3	Kmmig1.1	Kmmig1.2	Kmmig1.3	
		repeat-containing protein 2							
KLMA_80037	ALG6	dolichyl pyrophosphate Man9GlcNAc2 alpha-1	1580.18	1377.06	1600.62	1812.48	1840.81	1609.43	0.21
KLMA_80038	Pir	pirin-like protein	305.69	353.49	296.00	1465.97	812.15	877.32	1.72
KLMA_80039	YPT7	GTP-binding protein YPT7	684.27	774.11	699.20	805.73	832.37	834.97	0.20
KLMA_80040	PHO80	aminoglycoside antibiotic sensitivity protein 3	728.95	655.53	772.29	915.91	656.29	693.21	0.07
KLMA_80041	RRP6	exosome complex exonuclease RRP6	1169.85	1312.18	1404.50	1415.50	913.25	946.47	-0.25
KLMA_80042	IZH2	ADIPOR-like receptor IZH2	862.98	1294.28	1029.32	1890.70	1108.70	1166.88	0.39
KLMA_80043	RIT1	tRNA A64-2'-O-ribosylphosphate transferase	194.00	269.59	239.97	309.51	265.38	298.20	0.31
KLMA_80044	AEP2	hypothetical protein	413.86	434.04	461.67	394.46	378.27	433.91	-0.12
KLMA_80045	GPI12	N-acetylglucosaminyl-phosphatidylinositol de-N-acetylase	386.81	359.09	381.27	821.71	497.90	558.37	0.74
KLMA_80046	CAT8	regulatory protein CAT8	1102.83	1233.87	1227.87	836.85	932.62	940.42	-0.40
KLMA_80047	NAB6	RNA-binding protein NAB6	663.11	1005.67	743.06	655.18	743.06	790.88	-0.14
KLMA_80048	CTSD	plasmepsin-2	1211.00	1200.31	1321.67	857.04	1039.62	1025.99	-0.35
KLMA_80049	VAN1	mannan polymerase I complex VAN1 subunit	892.38	918.41	965.97	882.27	941.89	921.40	-0.02
KLMA_80050	SAM4	homocysteine S-methyltransferase 2	1843.54	1482.21	2031.83	1004.22	1286.46	1281.84	-0.58
KLMA_80051	PGM3	phosphoglucomutase YMR278W	386.81	523.53	414.16	400.34	295.71	405.38	-0.27
KLMA_80052	FPR1	FK506-binding protein 1	1749.48	1652.25	1644.47	829.28	1188.74	904.98	-0.79
KLMA_80053		DNA-directed RNA polymerase II subunit RPB1	9.41	13.42	12.18	88.31	101.10	160.77	3.32
KLMA_80054	HIP1	histidine permease	3944.57	2174.66	3848.06	640.05	563.62	427.86	-2.61

Locus_tag	UniProt_gene	Product	Unique exon reads						log_2 Fold Change
			KmWT.1	KmWT.2	KmWT.3	Kmmig1.1	Kmmig1.2	Kmmig1.3	
KLMA_80056	tsc2	conserved hypothetical protein	94.06	143.19	151.05	262.41	302.45	331.91	1.21
KLMA_80057	SUI2	eukaryotic translation initiation factor 2 subunit alpha	2099.85	1880.45	1995.29	1084.97	1653.78	1536.82	-0.48
KLMA_80058		UPF0103 protein YJR008W	104.64	259.53	143.74	901.61	541.71	689.75	2.07
KLMA_80059	GAP3	glyceraldehyde-3-phosphate dehydrogenase 3	280993.24	164705.63	249313.91	24882.72	60886.67	52281.29	-2.33
KLMA_80060		putative uncharacterized oxidoreductase YGL039W	29.39	22.37	18.27	85.79	28.64	46.68	1.20
KLMA_80061	MET3	sulfate adenylyltransferase	5844.55	3407.41	4937.07	1058.89	2197.18	2458.22	-1.31
KLMA_80062	MNN1	alpha-1,3-mannosyltransferase	208.10	242.75	209.52	213.63	133.95	184.97	-0.31
KLMA_80063	MNN1	alpha-1,3-mannosyltransferase	994.67	1137.67	852.69	160.64	200.51	230.78	-2.33
KLMA_80064	MNN1	alpha-1,3-mannosyltransferase	1573.12	1431.87	1627.42	385.20	712.74	720.01	-1.35
KLMA_80065	PDX1	pyruvate dehydrogenase complex protein X component	939.41	1128.72	834.42	386.05	612.48	716.55	-0.76
KLMA_80066	XKS1	xylulose kinase	335.08	418.38	347.17	201.85	245.16	329.32	-0.50
KLMA_80067		signal peptidase complex subunit SPC1	105.82	72.71	151.05	126.16	104.47	94.21	-0.02
KLMA_80068	mcfL	mitochondrial substrate carrier family protein L	166.95	81.66	120.59	386.89	395.96	433.91	1.72
KLMA_80069	SAG1	alpha-agglutinin	45.85	76.07	82.83	53.83	50.55	44.08	-0.46
KLMA_80071	HOL1	protein HOL1	1444.97	1054.89	1364.30	810.78	646.18	741.62	-0.81
KLMA_80072	SSL1	suppressor of stem-loop protein 1	198.70	177.87	225.35	173.26	153.33	153.85	-0.32
KLMA_80073		conserved hypothetical protein	79.95	112.98	97.45	143.82	79.19	86.44	0.09
KLMA_80074	THI73	thiamine pathway transporter THI73	25.87	8.95	29.24	20.19	15.16	9.51	-0.51
KLMA_80075	CMS1	protein CMS1	197.52	199.12	254.59	231.29	154.17	149.53	-0.28

Locus_tag	UniProt_gene	Product	Unique exon reads						log2 Fold Change
			KmWT.1	KmWT.2	KmWT.3	Kmmig1.1	Kmmig1.2	Kmmig1.3	
KLMA_80077	APL1	AP-2 complex subunit beta	596.09	623.09	640.73	528.18	625.96	560.97	-0.12
KLMA_80078	SSK1	conserved hypothetical protein	139.91	130.88	114.50	331.38	250.22	292.15	1.18
KLMA_80079	NSE1	non-structural maintenance of chromosomes element 1	72.90	124.17	80.40	151.39	139.01	140.03	0.63
KLMA_80080	PAM18	mitochondrial import inner membrane translocase subunit TIM14	336.26	294.21	325.24	364.18	315.93	262.76	-0.02
KLMA_80081	RLP24	ribosome biogenesis protein RLP24	932.35	827.80	847.82	361.66	441.46	401.92	-1.11
KLMA_80082		hypothetical protein	8.23	20.14	15.84	31.12	12.64	23.34	0.60
KLMA_80083	AHC1	protein AHC1	270.42	444.10	317.93	586.22	368.16	428.72	0.42
KLMA_80084		probable phospholipase YOR022C	393.87	573.87	450.71	411.28	411.97	436.50	-0.17
KLMA_80085		uncharacterized protein YOR021C	182.24	152.14	166.88	258.21	220.73	229.92	0.50
KLMA_80086		uncharacterized protein YOR020W-A	231.62	315.46	252.15	350.72	377.43	293.88	0.35
KLMA_80087	HSP10	10 kDa heat shock protein	420.91	487.73	421.47	436.51	706.84	508.24	0.31
KLMA_80088	PRP3	U4/U6 small nuclear ribonucleoprotein PRP3	119.92	104.03	142.52	163.17	123.84	112.37	0.13
KLMA_80089	TMA17	translation machinery-associated protein 17	111.69	107.39	105.98	351.56	255.27	263.63	1.42
KLMA_80090		uncharacterized protein YGL146C	882.97	493.32	783.26	250.64	223.26	171.14	-1.74
KLMA_80091	RRP42	exosome complex component RRP42	814.78	693.56	831.98	317.92	414.50	396.74	-1.05
KLMA_80092	TRM3	tRNA guanosine-2'-O-methyltransferase TRM3	570.23	614.14	534.76	618.18	601.53	678.52	0.14
KLMA_80093	ATG20	autophagy-related protein 20	277.47	492.21	320.37	1206.92	724.53	866.95	1.36
KLMA_80094		uncharacterized oxidoreductase SSP0419	11.76	16.78	7.31	137.09	39.60	59.64	2.71

Locus_tag	UniProt_gene	Product	Unique exon reads						log$_2$ Fold Change
			KmWT.1	KmWT.2	KmWT.3	Kmmig1.1	Kmmig1.2	Kmmig1.3	
KLMA_80095	IWR1	putative transcription factor IWR1	243.38	281.90	243.63	316.24	330.25	273.14	0.26
KLMA_80096	RPL9B	60S ribosomal protein L9-B	11231.73	8861.95	10818.18	3335.64	8254.59	6275.21	-0.79
KLMA_80098	NUP84	nucleoporin NUP84	911.19	951.97	967.19	466.79	668.93	687.16	-0.63
KLMA_80099	ARO2	chorismate synthase	1994.04	1455.36	1723.65	1127.86	1534.99	1544.60	-0.30
KLMA_80100	CYK3	cytokinesis protein 3	330.38	390.41	416.60	213.63	212.30	275.73	-0.70
KLMA_80101	ywtG	conserved hypothetical protein containing the Major Facilitator Superfamily (MFS) domain	29.39	44.75	34.11	2228.81	2396.85	2251.64	5.98
KLMA_80102	INO80	putative DNA helicase INO80	1165.15	1457.60	1152.35	1077.40	1026.14	950.79	-0.31
KLMA_80103	NUT1	mediator of RNA polymerase II transcription subunit 5	831.24	1007.90	973.28	610.61	566.99	638.76	-0.63
KLMA_80104	PEX14	peroxisomal membrane protein PEX14	262.19	270.71	324.02	349.88	297.39	317.22	0.17
KLMA_80105	LYS5	L-aminoadipate-semialdehyde dehydrogenase-phosphopantetheinyl transferase	36.45	52.58	36.54	132.89	92.67	96.81	1.36
KLMA_80106		solute carrier family 25 member 38 homolog	99.94	138.71	104.76	107.66	104.47	102.86	-0.13
KLMA_80107	CDC43	geranylgeranyl transferase type-1 subunit beta	172.83	272.95	213.17	333.06	273.81	365.62	0.56
KLMA_80108		protease B inhibitors 2 and 1	84.65	253.93	107.20	2194.32	1384.19	1275.79	3.44
KLMA_80109	YFH1	frataxin homolog	75.25	126.41	85.27	118.59	96.04	95.08	0.11
KLMA_80110		uncharacterized protein YDL121C	59.96	129.76	118.16	265.77	204.72	254.98	1.24
KLMA_80111	UBP1	ubiquitin carboxyl-terminal hydrolase 1	1028.76	1166.75	1040.28	712.38	622.59	704.45	-0.67
KLMA_80112	PUB1	nuclear and cytoplasmic polyadenylated	1851.77	2022.52	1722.43	691.35	1086.80	999.19	-1.01

Locus_tag	UniProt_gene	Product	Unique exon reads						log$_2$ Fold Change
			KmWT.1	KmWT.2	KmWT.3	Kmmig1.1	Kmmig1.2	Kmmig1.3	
KLMA_80113	CAB2	RNA-binding protein PUB1 uncharacterized protein YIL083C	380.94	421.73	375.18	430.62	353.00	370.81	-0.03
KLMA_80114	SAM50	sorting assembly machinery 50 kDa subunit	413.86	463.12	405.64	320.44	356.37	334.50	-0.34
KLMA_80115	CRZ1	transcriptional regulator CRZ1	96.41	121.93	97.45	156.44	145.75	153.85	0.53
KLMA_80116	SDS3	transcriptional regulatory protein SDS3	92.88	132.00	95.01	158.96	147.43	122.74	0.42
KLMA_80117	KTR5	probable mannosyltransferase KTR5	67.02	76.07	87.71	244.75	167.65	248.93	1.52
KLMA_80118	HHF1	histone H4	3849.34	4523.82	4471.74	3270.04	4430.59	3676.96	-0.18
KLMA_80119	HHT1	histone H3	8071.38	6674.99	9323.54	3724.21	5171.97	4358.94	-0.86
KLMA_80120	AIM19	loss of respiratory capacity protein 2	57.61	76.07	51.16	243.07	249.37	361.30	2.20
KLMA_80121	SIW14	tyrosine-protein phosphatase SIW14	171.66	222.61	185.16	804.05	402.70	442.55	1.51
KLMA_80122	AVT7	vacuolar amino acid transporter 7	703.09	649.94	574.96	2205.26	1333.64	1397.66	1.36
KLMA_80123	lag1	sphingosine N-acyltransferase lac1	159.90	105.15	126.69	627.43	440.62	570.47	2.07
KLMA_80124	SSN8	RNA polymerase II holoenzyme cyclin-like subunit	78.77	87.25	88.92	126.16	137.32	151.26	0.70
KLMA_80125	ICE2	protein ICE2	1109.89	844.58	1060.99	848.63	806.25	803.85	-0.29
KLMA_80126	UTP25	uncharacterized protein YIL091C	482.05	494.44	492.12	282.60	199.67	229.92	-1.04
KLMA_80127	RSM25	37S ribosomal protein S25	493.81	608.55	515.27	311.19	675.67	520.34	-0.10
KLMA_80128		uncharacterized protein YNL024C	223.39	324.41	275.30	286.80	137.32	135.70	-0.56
KLMA_80129	FAP1	FKBP12-associated protein 1	336.26	321.05	393.45	243.91	210.62	247.21	-0.58
KLMA_80130	LYS12	homoisocitrate dehydrogenase	3510.73	2056.08	3002.68	1789.77	2743.11	2417.60	-0.30
KLMA_80131		uncharacterized protein YNL022C	197.52	192.41	221.70	153.91	189.56	171.14	-0.25
KLMA_80132	HDA1	histone deacetylase HDA1	1069.91	1044.82	1184.02	317.08	483.58	458.11	-1.39

Locus_tag	UniProt_gene	Product	Unique exon reads						log2 Fold Change
			KmWT.1	KmWT.2	KmWT.3	Kmmig1.1	Kmmig1.2	Kmmig1.3	
KLMA_80133	PRK1	actin-regulating kinase PRK1	505.56	519.05	393.45	773.77	652.92	740.75	0.61
KLMA_80134		uncharacterized WD repeat-containing protein YNL035C	182.24	243.87	181.50	173.26	103.62	121.01	-0.61
KLMA_80135	BDP1	transcription factor TFIIIB component B"	485.58	439.63	432.43	465.95	391.75	458.11	-0.05
KLMA_80136		conserved hypothetical protein containing PIG-H super family	9.41	21.25	13.40	40.37	36.23	29.39	1.26
KLMA_80137		UPF0617 protein YIL096C	119.92	109.63	112.07	227.93	221.57	219.55	0.97
KLMA_80138	FYV10	protein FYV10	276.30	346.78	248.50	557.62	513.07	498.73	0.85
KLMA_80139	IDH1	isocitrate dehydrogenase [NAD] subunit 1	1340.33	1374.82	1337.50	1860.42	2624.32	2235.22	0.73
KLMA_80140		ATP synthase assembly factor FMC1	137.56	123.05	124.25	183.35	162.60	178.06	0.45
KLMA_80141	SGA1	glucoamylase	134.03	162.20	135.21	968.90	572.04	710.50	2.38
KLMA_80142	NCE103	carbonic anhydrase	335.08	494.44	332.55	4357.52	2609.15	3067.59	3.11
KLMA_80143	XBP1	hypothetical protein	4898.09	6034.00	4973.61	5249.05	2665.60	2738.27	-0.58
KLMA_80144	COG6	conserved oligomeric Golgi complex subunit 6	278.65	309.87	303.31	267.46	350.47	369.08	0.15
KLMA_80145	DPH1	diphthamide biosynthesis protein 1	282.17	270.71	306.97	308.67	268.75	253.26	-0.05
KLMA_80146	SHQ1	protein SHQ1	215.16	236.04	236.32	325.49	206.41	232.51	0.15
KLMA_80147	BOP3	protein BOP3	131.68	170.03	142.52	334.74	272.96	216.09	0.89
KLMA_80148	YIP3	prenylated Rab acceptor 1	544.36	479.90	553.03	502.95	644.50	619.74	0.16
KLMA_80149		leukotriene A-4 hydrolase	2941.67	2707.13	2476.45	1623.24	2136.52	2077.91	-0.48
KLMA_80150	SLM1	phosphatidylinositol 4	3215.62	3628.90	3516.73	2275.06	1953.70	1937.02	-0.75
KLMA_80151	ALG11	alpha-1,2-mannosyltransferase	357.42	341.19	376.40	420.53	546.77	503.92	0.45
KLMA_80152	MOB1	DBF2 kinase	313.92	404.95	345.95	644.25	623.43	600.73	0.81

Locus_tag	UniProt_gene	Product	Unique exon reads						log$_2$ Fold Change
			KmWT.1	KmWT.2	KmWT.3	Kmmig1.1	Kmmig1.2	Kmmig1.3	
		activator protein MOB1							
KLMA_80153	PFK26	6-phosphofructo-2-kinase/fructose-2	1248.62	1230.52	1276.60	710.69	582.15	607.64	-0.98
KLMA_80154		putative zinc metalloproteinase YIL108W	161.07	214.78	129.12	355.77	264.54	312.03	0.88
KLMA_80155	SEC24	protein transport protein SEC24	3286.16	3322.39	3410.75	1397.84	2008.47	2084.82	-0.87
KLMA_80156		uncharacterized protein YNL050C	268.07	228.20	216.83	256.52	227.47	204.85	-0.05
KLMA_80157	COG5	conserved oligomeric Golgi complex subunit 5	225.74	204.71	243.63	275.87	294.87	297.34	0.37
KLMA_80158	HPM1	mitotic exit network interactor 1	571.40	431.80	546.94	629.11	422.92	586.03	0.08
KLMA_80159	COX5A	cytochrome c oxidase polypeptide 5A	3141.55	2723.91	2904.01	1857.06	1979.82	1658.69	-0.67
KLMA_80160	HOS4	protein HOS4	409.15	521.29	462.89	300.26	333.62	372.54	-0.47
KLMA_80161	MSG5	tyrosine-protein phosphatase MSG5	908.84	1313.30	902.63	2649.33	1659.68	2254.23	1.07
KLMA_80162	VAC7	vacuolar segregation protein 7	881.80	1085.09	920.90	481.93	543.40	560.97	-0.86
KLMA_80163	POR1	mitochondrial outer membrane protein porin 1	6892.12	4652.47	5966.38	1478.58	3786.94	4188.66	-0.89
KLMA_80164	NUP159	nucleoporin NUP159	1595.46	1698.11	1586.00	1303.64	1469.28	1421.86	-0.22
KLMA_80165	HIS5	histidinol-phosphate aminotransferase	1634.26	1149.97	1606.71	1174.96	1391.77	1370.00	-0.16
KLMA_80166	OCA2	tyrosine-protein phosphatase-like protein OCA2	284.53	189.05	239.97	116.91	139.01	121.01	-0.92
KLMA_80167		uncharacterized protein YNL058C	363.30	378.10	348.38	209.42	210.62	254.12	-0.69
KLMA_80168	RHO3	GTP-binding protein RHO3	772.45	749.50	808.84	542.48	689.15	724.33	-0.25
KLMA_80169	ARP5	actin-related protein 5	798.32	806.55	800.31	508.84	528.23	523.80	-0.62
KLMA_80170	NOP2	putative ribosomal RNA methyltransferase Nop2	1389.71	1509.06	1639.60	503.79	592.26	602.45	-1.42

Locus_tag	UniProt_gene	Product	Unique exon reads						$\log_2$ Fold Change
			KmWT.1	KmWT.2	KmWT.3	Kmmig1.1	Kmmig1.2	Kmmig1.3	
KLMA_80171	TRM6	tRNA (adenine-N(1)-)-methyltransferase non-catalytic subunit TRM6	524.37	559.33	468.98	206.06	279.70	280.05	-1.02
KLMA_80172	MTQ1	mitochondrial N(5)-glutamine methyltransferase MTQ1	65.84	48.10	63.34	98.40	57.29	52.73	0.24
KLMA_80173	DNAJA1	mitochondrial protein import protein MAS5	1816.50	1957.64	1908.80	996.65	1139.87	1154.78	-0.79
KLMA_80174	AQR1	probable transporter AQR1	5092.08	2386.08	4366.98	650.14	467.57	528.12	-2.85
KLMA_80175	QDR2	quinidine resistance protein 2	920.60	601.83	769.86	346.52	273.81	351.79	-1.24
KLMA_80176		uncharacterized oxidoreductase YJR096W	31.74	40.27	29.24	727.52	539.19	667.28	4.25
KLMA_80177	SFC1	succinate/fumarate mitochondrial transporter	831.24	666.72	774.73	1149.73	2292.38	1994.06	1.26
KLMA_80178	CPA2	carbamoyl-phosphate synthase arginine-specific large chain	3728.24	2154.52	3883.39	2455.05	3102.84	3530.88	-0.10
KLMA_80179	MCH4	probable transporter MCH4	1287.42	730.48	1108.49	1096.74	1140.71	1193.67	0.13
KLMA_80180		uncharacterized sulfatase PB10D8.02c	313.92	242.75	250.93	182.51	92.67	115.82	-1.05
KLMA_80181	ZUO1	zuotin	2096.32	2014.69	2146.34	977.31	1413.68	1288.75	-0.77
KLMA_80182	ERV29	ER-derived vesicles protein ERV29	1921.14	1571.70	1799.17	1141.32	1773.41	1646.59	-0.21
KLMA_80183	GLC8	protein GLC8	384.46	372.51	330.11	623.22	539.19	605.05	0.70
KLMA_80184		uncharacterized protein YMR310C	402.10	482.14	437.31	349.04	321.83	322.40	-0.41
KLMA_80185	BGL2	glucan 1	9409.36	9617.04	7803.32	2862.12	3452.47	4711.59	-1.28
KLMA_80186	NIP1	eukaryotic translation initiation factor 3 subunit C	4611.21	4948.91	4921.23	1982.38	2690.03	2412.41	-1.03
KLMA_80187		conserved hypothetical protein	3481.33	3868.29	3135.46	2107.69	1706.86	1626.71	-0.95

Locus_tag	UniProt_gene	Product	Unique exon reads						log2 Fold Change
			KmWT.1	KmWT.2	KmWT.3	Kmmig1.1	Kmmig1.2	Kmmig1.3	
		containing HMG-box super family							
KLMA_80188	YOR1	oligomycin resistance ATP-dependent permease YOR1	958.22	1022.45	812.49	565.19	447.36	572.20	-0.82
KLMA_80189	RDS1	uncharacterized transcriptional regulatory protein YCR106W	1241.57	1166.75	1180.36	770.41	591.42	681.97	-0.81
KLMA_80190	RDS1	uncharacterized transcriptional regulatory protein YCR106W	253.96	251.70	337.42	407.91	246.00	283.51	0.15
KLMA_80191	mug70	meiotically up-regulated gene 70 protein	38.80	36.92	56.03	100.09	93.51	119.28	1.25
KLMA_80192		conserved hypothetical membrane brotien	23.51	21.25	24.36	60.56	48.86	87.30	1.51
KLMA_80193	PSE1	importin subunit beta-3	2247.99	1873.74	2073.25	1797.34	2302.49	2308.69	0.05
KLMA_80194	PXR1	protein PXR1	302.16	242.75	310.62	174.94	153.33	149.53	-0.84
KLMA_80195	GAS1	beta-1,3-glucanosyltransferas e	377.41	1454.25	403.20	1889.02	1363.97	1875.65	1.20
KLMA_80196	GAS1	beta-1,3-glucanosyltransferas e	20943.25	17754.10	17573.91	28773.46	26619.75	24804.35	0.51
KLMA_80197	FKS3	1,3-beta-glucan synthase component FKS3	184.59	156.61	142.52	406.23	266.22	247.21	0.93
KLMA_80198	SCW4	probable family 17 glucosidase SCW4	26747.82	18480.11	23737.63	18759.81	20254.84	17933.61	-0.28
KLMA_80199	CWC22	pre-mRNA-splicing factor CWC22	233.97	186.81	211.95	225.40	273.81	263.63	0.27
KLMA_80200	CAB4	uncharacterized protein YGR277C	263.36	177.87	231.44	305.30	296.55	301.66	0.43
KLMA_80201	UBP15	ubiquitin carboxyl-terminal hydrolase 15	778.33	1048.18	867.31	1216.17	917.46	883.37	0.16
KLMA_80202	YME2	mitochondrial escape protein 2	984.08	1210.38	965.97	1018.52	1058.99	1102.92	0.01
KLMA_80203	MKT1	protein MKT1	2166.87	2251.84	2083.00	1463.44	1590.60	1721.79	-0.45

Locus_tag	UniProt_gene	Product	Unique exon reads						log2 Fold Change
			KmWT.1	KmWT.2	KmWT.3	Kmmig1.1	Kmmig1.2	Kmmig1.3	
KLMA_80204	OST3	dolichyl-diphosphooligosaccharide--protein glycosyltransferase subunit 3	781.86	832.28	736.97	1070.67	1405.25	1344.93	0.70
KLMA_80205	SNN1	uncharacterized protein YNL086W	19.99	6.71	7.31	15.14	19.38	10.37	0.40
KLMA_80206	TCB1	tricalbin-1	3754.10	3849.28	3781.06	4587.97	4842.56	4836.92	0.33
KLMA_80207	TOP2	DNA topoisomerase 2	1266.26	1592.96	1371.61	745.18	893.87	879.05	-0.75
KLMA_80208	RHO2	GTP-binding protein RHO2	109.34	132.00	79.18	172.42	160.91	189.29	0.70
KLMA_80209	NST1	stress response protein NST1	1039.34	1119.77	1171.84	950.40	821.42	766.68	-0.39
KLMA_80210	YVC1	calcium channel YVC1	516.14	617.50	550.59	839.38	800.35	820.27	0.55
KLMA_80211		UPF0586 protein YNL092W	110.52	144.31	127.90	217.83	152.49	201.39	0.58
KLMA_80212	VPS21	vacuolar protein sorting-associated protein 21	539.66	520.17	505.52	644.25	694.20	678.52	0.37
KLMA_80213	PTC5	protein phosphatase 2C homolog 5	714.84	1010.14	816.14	894.89	807.94	960.30	0.07
KLMA_80214	TMA46	translation machinery-associated protein 46	746.59	576.11	726.00	412.96	535.82	529.85	-0.47
KLMA_80216	APP1	actin patch protein 1	297.46	286.37	282.61	242.22	197.14	210.90	-0.41
KLMA_80217	ECM3	protein ECM3	1408.52	1220.45	1277.81	552.58	534.13	595.54	-1.22
KLMA_80219		uncharacterized protein YOR093C	618.43	724.89	518.92	956.28	829.84	998.33	0.58
KLMA_80220	ARF3	ADP-ribosylation factor 3	932.35	751.73	958.67	627.43	922.51	873.86	-0.12
KLMA_80221	RKI1	ribose-5-phosphate isomerase	1229.81	1022.45	1057.33	1370.93	1262.87	1262.82	0.24
KLMA_80222	RPS7A	40S ribosomal protein S7-A	18695.26	15558.19	18426.60	5581.27	11792.15	9704.96	-0.96
KLMA_80223		hypothetical protein	19.99	22.37	9.75	26.91	16.85	30.25	0.50
KLMA_80224		nucleoporin NUP1	303.34	445.22	409.29	161.48	166.81	248.93	-1.00
KLMA_80225	KTR1	alpha-1,2-mannosyltransferase	3240.31	3312.32	3024.61	2573.64	2573.77	3016.59	-0.23
KLMA_80226	CRC1	mitochondrial carnitine carrier	14.11	13.42	26.80	216.15	422.08	547.14	4.45

Locus_tag	UniProt_gene	Product	Unique exon reads						log$_2$ Fold Change
			KmWT.1	KmWT.2	KmWT.3	Kmmig1.1	Kmmig1.2	Kmmig1.3	
KLMA_80227	PHO23	transcriptional regulatory protein PHO23	317.45	346.78	321.59	182.51	186.19	230.78	-0.72
KLMA_80228	RAS2	hypothetical protein	360.95	322.17	421.47	282.60	358.90	325.86	-0.19
KLMA_80229	OST2	dolichyl-diphosphooligosaccharide protein glycosyltransferase subunit OST2	283.35	269.59	230.23	408.75	461.68	447.74	0.75
KLMA_80230	OCA1	putative tyrosine-protein phosphatase OCA1	218.69	233.80	214.39	313.71	278.02	292.15	0.41
KLMA_80231		[PSI+] induction protein 2	1007.60	938.55	964.76	2931.09	2437.29	2669.12	1.47
KLMA_80232	AIM37	uncharacterized protein YNL100W	506.74	548.14	518.92	497.07	593.10	538.49	0.05
KLMA_80233	AVT4	vacuolar amino acid transporter 4	1189.84	1039.23	1186.46	1484.47	961.27	983.63	0.01
KLMA_80234	POL1	DNA polymerase alpha catalytic subunit A	874.74	981.06	845.38	412.12	435.56	519.48	-0.98
KLMA_80235	VAM3	syntaxin VAM3	95.23	83.90	92.58	212.79	221.57	178.06	1.17
KLMA_80236	MET4	transcriptional activator of sulfur metabolism MET4	329.20	333.36	419.04	167.37	184.50	182.38	-1.02
KLMA_80237		uncharacterized protein YOR352W	460.89	425.09	381.27	509.68	463.36	485.77	0.20
KLMA_80238	CYC3	cytochrome c heme lyase	739.53	579.46	650.48	1092.54	747.28	731.24	0.38
KLMA_80239	MSC6	meiotic sister-chromatid recombination protein 6	1242.75	1249.53	1320.45	512.20	775.92	783.10	-0.88
KLMA_80240	CLN3	G1/S-specific cyclin CLN3	2728.87	2161.23	2738.35	2629.15	2434.76	2410.68	-0.03
KLMA_80241		jmjC domain-containing protein 4	2228.01	1832.35	2080.56	346.52	494.53	491.82	-2.20
KLMA_80242	GDS1	protein GDS1	2541.93	2096.35	2583.65	886.48	857.64	833.24	-1.49
KLMA_80243	CIR2	probable electron transfer flavoprotein-ubiquinone oxidoreductase	3341.42	2751.88	3145.20	985.72	1503.82	1535.96	-1.20
KLMA_80244	CDC24	cell division control	1113.41	1045.94	1221.78	735.93	835.74	834.10	-0.49

Locus_tag	UniProt_gene	Product	Unique exon reads						log2 Fold Change
			KmWT.1	KmWT.2	KmWT.3	Kmmig1.1	Kmmig1.2	Kmmig1.3	
		protein 24							
KLMA_80245	ERV46	ER-derived vesicles protein ERV46	827.71	690.21	749.15	523.14	684.93	675.92	-0.27
KLMA_80246	HRP1	nuclear polyadenylated RNA-binding protein 4	1387.36	1231.63	1410.59	939.46	1187.05	1217.01	-0.27
KLMA_80247	TRM11	tRNA guanosine-2'-O-methyltransferase TRM11	539.66	503.39	645.61	322.13	316.77	356.11	-0.76
KLMA_80248	YPT11	GTP-binding protein YPT11	297.46	284.14	366.66	202.70	314.24	295.61	-0.22
KLMA_80249		UPF0364 protein YMR027W	2480.79	2223.88	2341.24	890.68	1098.59	1202.32	-1.14
KLMA_80250	MPE1	protein MPE1	159.90	203.59	174.19	248.11	209.78	230.78	0.36
KLMA_80252	TOA2	transcription initiation factor IIA small subunit	653.71	569.39	588.35	535.75	641.13	548.86	-0.07
KLMA_80253	TAP42	type 2A phosphatase-associated protein 42	306.87	353.49	321.59	461.74	403.55	440.82	0.41
KLMA_80254	NUP120	nucleoporin NUP120	1191.01	1224.92	1164.53	648.46	895.55	816.81	-0.60
KLMA_80255	FAR8	factor arrest protein 8	435.02	592.88	512.83	363.34	450.73	484.90	-0.25
KLMA_80256		translationally-controlled tumor protein homolog	13804.23	10284.87	12154.46	4714.97	6819.01	5481.73	-1.09
KLMA_80257	DEF1	uncharacterized protein YKL054C	3028.68	4028.26	3176.87	1366.72	1925.90	1771.92	-1.01
KLMA_80258	MDM35	mitochondrial distribution and morphology protein 35	91.71	107.39	92.58	165.69	147.43	117.55	0.56
KLMA_80259	ASK1	DASH complex subunit ASK1	44.68	25.73	29.24	28.60	26.96	42.35	-0.03
KLMA_80260	SFK1	protein SFK1	174.01	153.26	215.61	481.09	417.87	479.72	1.35
KLMA_80261	EIS1	uncharacterized protein YMR031C	2062.23	2326.79	1930.73	1780.52	1706.02	1822.06	-0.25
KLMA_80262	HOF1	cytokinesis protein 2	270.42	227.09	308.19	575.28	643.65	570.47	1.15
KLMA_80263	CSE4	histone H3-like centromeric protein CSE4	88.18	106.27	85.27	122.79	106.99	124.47	0.34

Locus_tag	UniProt_gene	Product	Unique exon reads						log$_2$ Fold Change
			KmWT.1	KmWT.2	KmWT.3	Kmmig1.1	Kmmig1.2	Kmmig1.3	
KLMA_80264	ARP9	actin-like protein ARP9	962.92	896.04	963.54	602.20	851.74	798.66	-0.33
KLMA_80265	PKH1	probable serine/threonine-protein kinase YOL100W	1068.74	1157.80	1186.46	905.82	722.00	841.88	-0.47
KLMA_80266		hypothetical protein	32.92	31.32	37.76	88.31	34.54	33.71	0.62
KLMA_80267	IZH1	ADIPOR-like receptor IZH1	1930.55	1919.60	2319.31	4509.76	1929.27	1797.86	0.42
KLMA_80268	MZM1	protein FMP36	289.23	308.75	249.72	162.32	235.05	217.82	-0.46
KLMA_80269	TPT1	tRNA 2'-phosphotransferase	231.62	151.02	165.67	248.11	190.40	183.24	0.18
KLMA_80270	RSM28	37S ribosomal protein RSM28	914.72	953.09	934.30	576.13	887.13	720.87	-0.36
KLMA_80271	VPS3	vacuolar protein sorting-associated protein 3	579.63	642.11	655.35	675.37	750.65	708.77	0.19
KLMA_80272	PUF6	pumilio homology domain family member 6	1724.79	1529.20	1824.75	632.48	785.19	750.26	-1.23
KLMA_80273	ITR2	myo-inositol transporter 2	7605.79	2534.86	7043.21	6185.15	6034.66	6223.35	0.10
KLMA_80274	SEC20	conserved hypothetical protein	292.76	211.42	258.24	195.13	259.48	305.98	0.00
KLMA_80275		non-disjunction protein 1	30.57	13.42	31.67	47.94	21.90	8.64	0.06
KLMA_80276	ERG24	delta(14)-sterol reductase	857.11	689.09	861.22	340.63	567.83	549.73	-0.72
KLMA_80277	SLI1	N-acetyltransferase SLI1	278.65	222.61	294.79	529.03	502.12	506.51	0.95
KLMA_80278	HCH1	hsp90 co-chaperone HCH1	315.10	246.10	285.04	278.39	358.05	332.78	0.20
KLMA_80279		ribonucleases P/MRP protein subunit POP3	82.30	57.05	77.96	47.94	51.39	63.10	-0.42
KLMA_80280		cell wall integrity and stress response component 2	4473.65	3700.50	3744.52	2926.88	2738.05	2735.68	-0.50
KLMA_80281	rplO	54S ribosomal protein L10	748.94	752.85	816.14	477.72	663.87	531.58	-0.47
KLMA_80282		uncharacterized membrane protein YOL107W	244.55	248.34	280.17	271.66	316.77	308.57	0.21

Locus_tag	UniProt_gene	Product	Unique exon reads						log2 Fold Change
			KmWT.1	KmWT.2	KmWT.3	Kmmig1.1	Kmmig1.2	Kmmig1.3	
KLMA_80284	VID24	vacuolar import and degradation protein 24	2493.72	1553.81	2299.82	403.71	427.14	426.13	-2.34
KLMA_80285	PHO88	inorganic phosphate transport protein PHO88	1492.00	1198.07	1302.18	1398.68	1421.26	1476.32	0.11
KLMA_80286	UBA3	NEDD8-activating enzyme E1 catalytic subunit	14.11	22.37	21.93	69.81	62.34	72.61	1.81
KLMA_80287	ISA2	iron-sulfur assembly protein 2	150.49	156.61	135.21	256.52	243.48	196.21	0.65
KLMA_80288		hypothetical protein	92.88	157.73	92.58	412.96	363.11	360.44	1.72
KLMA_80289	AIM3	uncharacterized protein YBR108W	161.07	246.10	155.92	343.99	294.02	363.03	0.83
KLMA_80290	CMD1	calmodulin	828.89	993.36	877.05	1227.10	1399.35	1353.58	0.56
KLMA_80292	ALG1	chitobiosyldiphosphodolichol beta-mannosyltransferase	1194.54	974.34	1152.35	1440.73	1516.46	1542.01	0.44
KLMA_80293	HOS1	histone deacetylase HOS1	69.37	132.00	127.90	75.70	82.56	93.35	-0.39
KLMA_80294	SPE3	spermidine synthase	1895.27	1406.14	1627.42	922.64	1280.57	1367.41	-0.47
KLMA_80295	MED1	mediator of RNA polymerase II transcription subunit 1	239.85	262.88	261.90	256.52	186.19	249.80	-0.14
KLMA_80296	YSA1	ADP-ribose pyrophosphatase	315.10	409.43	281.39	396.98	536.66	502.19	0.51
KLMA_80297	CYC8	general transcriptional corepressor CYC8	1711.86	1458.72	1550.67	700.60	1048.04	919.67	-0.82
KLMA_80298	RAD16	DNA repair protein RAD16	451.48	393.76	501.87	321.28	192.08	271.41	-0.78
KLMA_80299	LYS2	L-aminoadipate-semialdehyde dehydrogenase	2026.96	1553.81	1952.66	1468.49	2109.56	2242.13	0.07
KLMA_80300	NOT5	general negative regulator of transcription subunit 5	988.79	931.84	1022.01	491.18	696.73	697.53	-0.64
KLMA_80301	LTP1	low molecular weight phosphotyrosine protein phosphatase	79.95	110.75	103.54	63.08	96.04	78.66	-0.31

Locus_tag	UniProt_gene	Product	Unique exon reads						log2 Fold Change
			KmWT.1	KmWT.2	KmWT.3	Kmmig1.1	Kmmig1.2	Kmmig1.3	
KLMA_80302	TKL1	transketolase	9372.91	8000.59	8306.40	3828.50	7487.09	8430.90	-0.38
KLMA_80303		protein OPY2	1305.06	1402.79	1337.50	710.69	590.58	654.32	-1.05
KLMA_80304		hypothetical protein	4.70	2.24	1.22	25.23	7.58	1.73	2.08
KLMA_80305		conserved hypothetical protein	286.88	276.31	324.02	408.75	197.14	172.87	-0.19
KLMA_80306	ADH3	alcohol dehydrogenase 3	654.88	645.46	537.19	9119.60	5999.28	7112.77	3.60
KLMA_80307		hypothetical protein	2.35	0.00	0.00	5.89	2.53	0.00	1.84
KLMA_80308	MRL1	mannose 6-phosphate receptor-like protein 1	358.60	407.19	415.38	236.34	237.58	274.00	-0.66
KLMA_80309	TEF	elongation factor 1-alpha	146875.54	131368.72	137571.53	55714.25	82674.82	75753.68	-0.96
KLMA_80310	MUD1	U1 small nuclear ribonucleoprotein A	79.95	89.49	131.56	88.31	127.21	114.96	0.14
KLMA_80311	CBP6	cytochrome B pre-mRNA-processing protein 6	362.12	308.75	344.73	194.28	357.21	271.41	-0.30
KLMA_80312	GRS1	glycyl-tRNA synthetase 1	4713.50	4414.19	4803.07	1983.22	3108.74	2956.95	-0.79
KLMA_80313	DIB1	spliceosomal protein DIB1	22.34	49.22	42.63	61.40	39.60	57.91	0.48
KLMA_80314	MRPL36	54S ribosomal protein L36	245.73	204.71	227.79	145.50	282.23	206.58	-0.10
KLMA_80315	TFC1	transcription factor tau 95 kDa subunit	406.80	476.55	389.80	454.17	452.41	421.80	0.06
KLMA_80316	PTC4	protein phosphatase 2C homolog 4	506.74	483.26	482.38	287.64	303.29	306.85	-0.71
KLMA_80317	TPS1	trehalose-6-phosphate synthase	6196.09	6059.73	5844.57	1525.68	1361.44	1461.62	-2.06
KLMA_80318	amdS	putative amidase C550.07	241.02	206.95	219.26	533.23	392.59	485.77	1.08
KLMA_80319		uncharacterized protein YMR252C	124.63	189.05	137.65	220.36	218.20	146.94	0.37
KLMA_80320		conserved hypothetical protein	277.47	338.95	261.90	337.26	299.08	274.86	0.05
KLMA_80321	AIM39	uncharacterized protein YOL053W	306.87	334.48	226.57	449.13	386.70	354.38	0.45
KLMA_80322		cytochrome c oxidase subunit 7	450.30	415.02	486.03	212.79	350.47	208.31	-0.81
KLMA_80323	PSH1	RING finger protein PSH1	239.85	242.75	221.70	254.84	211.46	272.27	0.07

Locus_tag	UniProt_gene	Product	Unique exon reads						log2 Fold Change
			KmWT.1	KmWT.2	KmWT.3	Kmmig1.1	Kmmig1.2	Kmmig1.3	
KLMA_80324	PET111	protein PET111	513.79	466.48	507.96	294.37	321.83	333.64	-0.65
KLMA_80325	ROY1	uncharacterized protein YMR258C	1506.11	1222.69	1452.01	359.13	478.53	436.50	-1.71
KLMA_80326	TY2B-LR1	hypothetical protein	7.05	0.00	3.65	3.36	6.74	5.19	0.52
KLMA_80327		uncharacterized protein YMR259C	743.06	784.17	712.60	692.19	622.59	596.40	-0.23
KLMA_80328	TFB4	RNA polymerase II transcription factor B subunit 4	566.70	500.04	571.30	489.50	458.31	458.11	-0.22
KLMA_80329		zinc finger protein 511	117.57	51.46	76.74	111.86	53.92	69.15	-0.06
KLMA_80330	BRR1	pre-mRNA-splicing factor BRR1	136.38	104.03	108.41	186.72	167.65	163.36	0.57
KLMA_80331	YMC1	carrier protein YMC1	1250.98	797.60	1165.75	820.87	1219.06	1095.14	-0.04
KLMA_80332	ARO7	chorismate mutase	218.69	229.32	218.04	672.01	715.26	645.67	1.61
KLMA_80333	JID1	J domain-containing protein 1	122.28	136.48	127.90	317.92	184.50	210.90	0.88
KLMA_80334	FCY1	cytosine deaminase	357.42	357.97	309.40	374.27	328.57	380.32	0.08
KLMA_80335		uncharacterized protein YPR063C	604.32	387.05	518.92	350.72	486.11	487.50	-0.19
KLMA_80336	AIM10	probable prolyl-tRNA synthetase	409.15	496.68	433.65	256.52	304.98	248.93	-0.73
KLMA_80337	SBH1	protein transport protein Sec61 subunit beta	629.01	465.36	583.48	490.34	599.84	452.06	-0.12
KLMA_80338	PIN4	RNA-binding protein PIN4	1661.30	1463.19	1554.33	581.17	828.16	863.49	-1.04
KLMA_80339	ADH6	NADP-dependent alcohol dehydrogenase 6	376.23	340.07	281.39	2408.79	1001.71	1013.89	2.15
KLMA_80340	SAS3	histone acetyltransferase SAS3	119.92	112.98	127.90	220.36	288.13	246.34	1.07
KLMA_80341	DOT6	transcriptional regulatory protein DOT6	2169.22	2296.59	2218.21	1141.32	1027.82	1162.56	-1.00
KLMA_80342		deoxyribonuclease Tat-D	645.47	740.55	702.86	337.26	400.18	423.53	-0.85
KLMA_80343	PTC2	protein phosphatase 2C homolog 3	3096.87	2577.37	2729.82	2151.43	2354.72	2367.46	-0.29
KLMA_80344	TRP2	anthranilate synthase component 1	1474.36	1219.33	1454.44	736.77	1020.24	936.96	-0.62

Locus_tag	UniProt_gene	Product	Unique exon reads						log$_2$ Fold Change
			KmWT.1	KmWT.2	KmWT.3	Kmmig1.1	Kmmig1.2	Kmmig1.3	
KLMA_80345	PTH2	peptidyl-tRNA hydrolase 2	805.37	611.90	872.18	295.21	358.90	344.01	-1.20
KLMA_80346	SHP1	UBX domain-containing protein 1	430.32	525.77	473.85	427.26	390.91	424.40	-0.20
KLMA_80348	MET6	5-methyltetrahydropteroyltriglutamate homocysteine methyltransferase	62695.72	39094.60	52909.32	10500.62	20868.16	22983.16	-1.51
KLMA_80349		ino eighty subunit 5	63.49	54.81	76.74	23.55	34.54	39.76	-0.99
KLMA_80350	TSC11	target of rapamycin complex 2 subunit TSC11	767.75	940.79	827.11	882.27	691.67	815.09	-0.09
KLMA_80351	AIM11	hypothetical protein	97.59	110.75	107.20	106.81	136.48	105.45	0.14
KLMA_80353	CMC2	coiled-coil-helix-coiled-coil-helix domain-containing protein YBL059C-A	91.71	136.48	87.71	105.13	113.73	126.20	0.12
KLMA_80354	PUP3	proteasome component PUP3	596.09	696.92	629.77	751.91	850.06	858.30	0.36
KLMA_80355	YEL1	PH and SEC7 domain-containing protein YBL060W	319.80	326.65	271.64	402.87	354.68	476.26	0.43
KLMA_80356	RAD51	DNA repair protein RAD51	1041.70	847.94	1040.28	453.33	411.97	696.67	-0.91
KLMA_80357	SKT5	protein SKT5	438.55	498.92	440.96	719.95	470.94	630.11	0.40
KLMA_80358	PRX1	mitochondrial peroxiredoxin PRX1	2031.66	1623.16	1879.57	2378.51	2085.13	2545.52	0.34
KLMA_80359	SEF1	putative transcription factor SEF1	1606.05	1366.99	1616.45	1848.65	1178.63	1197.13	-0.12
KLMA_80360	UBP13	ubiquitin carboxyl-terminal hydrolase 13	904.14	1162.28	1019.57	985.72	800.35	853.12	-0.23
KLMA_80361	PRS4	ribose-phosphate pyrophosphokinase 2	945.29	965.40	1014.70	553.42	588.89	567.88	-0.77
KLMA_80362	UBC6	ubiquitin-conjugating enzyme E2 6	443.25	478.78	455.58	637.52	726.22	701.86	0.58
KLMA_80363	AST1	protein AST1	933.53	997.84	1058.55	985.72	977.27	968.08	-0.03
KLMA_80364	DPH3	diphthamide biosynthesis protein 3	64.67	62.64	46.29	44.58	40.44	29.39	-0.60

Locus_tag	UniProt_gene	Product	Unique exon reads						log2 Fold Change
			KmWT.1	KmWT.2	KmWT.3	Kmmig1.1	Kmmig1.2	Kmmig1.3	
KLMA_80365	RPS8B	40S ribosomal protein S8	22918.48	20301.27	21904.35	8181.82	16082.05	13311.91	-0.79
KLMA_80366	AAR2	A1 cistron-splicing factor AAR2	123.45	130.88	82.83	63.92	57.29	51.00	-0.97
KLMA_80367	SSA3	heat shock protein SSA3	5273.14	8903.34	5285.45	4958.88	3917.52	3882.68	-0.61
KLMA_80369	ILS1	isoleucyl-tRNA synthetase	5077.97	4570.81	4976.05	1550.91	2628.53	2574.04	-1.11
KLMA_80371		hypothetical protein	64.67	44.75	35.33	89.99	78.35	70.88	0.72
KLMA_80372	ATG8	autophagy-related protein 8	83.48	120.81	93.80	652.66	379.96	375.13	2.24
KLMA_80373	NUP170	nucleoporin NUP170	2590.13	2515.84	2581.21	1232.15	1674.00	1553.24	-0.79
KLMA_80374		conserved hypothetical protein	0.00	0.00	2.44	58.03	28.64	46.68	5.83
KLMA_80375	PET112	glutamyl-tRNA(Gln) amidotransferase subunit B	272.77	276.31	294.79	235.50	299.08	229.92	-0.14
KLMA_80376		uncharacterized protein YBL081W	202.23	128.64	154.70	119.43	131.43	80.38	-0.55
KLMA_80377	GLE2	nucleoporin GLE2	644.30	653.29	545.72	471.83	628.49	500.46	-0.20
KLMA_80378	FLO8	hypothetical protein	1176.90	1465.43	1332.63	755.27	846.69	834.10	-0.71
KLMA_80379	ALG3	dolichyl-P-Man:Man(5)GlcNAc(2)-PP-dolichyl mannosyltransferase	498.51	492.21	472.63	431.46	380.80	384.64	-0.29
KLMA_80380	KAP123	importin subunit beta-4	8096.07	5745.39	6939.67	2612.33	3880.45	3454.82	-1.06
KLMA_80381	SWI4	regulatory protein SWI4	436.20	587.29	433.65	328.01	341.20	399.33	-0.45
KLMA_80382	LSM4	U6 snRNA-associated Sm-like protein LSm4	498.51	422.85	461.67	381.84	540.87	440.82	-0.02
KLMA_80383	TMN3	transmembrane 9 superfamily member 3	4467.77	3965.62	4084.38	2517.29	2657.17	2560.22	-0.69
KLMA_80384	CDC27	anaphase-promoting complex subunit CDC27	823.01	909.46	835.63	698.92	671.45	651.72	-0.35
KLMA_80385	BOI1	protein BOI2	1327.40	1614.21	1416.68	795.64	1017.71	966.35	-0.65
KLMA_80386		hypothetical protein	177.54	182.34	175.41	273.34	236.74	257.58	0.52
KLMA_80387		uncharacterized	359.77	322.17	360.57	861.24	411.97	534.17	0.79

Locus_tag	UniProt_gene	Product	Unique exon reads						log2 Fold Change
			KmWT.1	KmWT.2	KmWT.3	Kmmig1.1	Kmmig1.2	Kmmig1.3	
		protein YBL086C							
KLMA_80389	HXT14	hexose transporter HXT14	186.94	146.54	172.97	3413.02	1731.29	2506.63	3.92
KLMA_80390	RTC1	uncharacterized WD repeat-containing protein YOL138C	1787.11	1646.65	1605.49	2179.18	1315.11	1357.90	-0.05
KLMA_80391		uncharacterized membrane protein YNL320W	1055.80	872.55	1064.64	1419.71	743.91	680.25	-0.07
KLMA_80392	TIF45	eukaryotic translation initiation factor 4E	1255.68	1286.45	1319.23	645.93	964.64	859.17	-0.64
KLMA_80393	VNX1	low affinity vacuolar monovalent cation/H(+) antiporter	919.42	944.14	1018.35	1068.14	965.48	1039.82	0.09
KLMA_80394		protein KRE1	1359.14	2013.57	1315.58	1736.79	1294.89	1488.42	-0.05
KLMA_80395	ARG8	acetylornithine aminotransferase	1535.50	1149.97	1667.61	1341.49	1764.15	1934.42	0.21
KLMA_80396	KEI1	UPF0596 Golgi apparatus membrane protein YDR367W	629.01	538.07	711.39	437.35	465.89	418.35	-0.51
KLMA_80397	RIO1	serine/threonine-protein kinase RIO1	293.93	356.85	344.73	319.60	245.16	290.42	-0.22
KLMA_80398	RTC5	uncharacterized protein YOR118W	510.27	583.94	504.30	454.17	380.80	390.69	-0.38
KLMA_80399	ESF1	pre-rRNA-processing protein ESF1	1215.70	1042.58	1192.55	716.58	695.04	670.74	-0.73
KLMA_80400	CDC40	pre-mRNA-processing factor 17	210.46	203.59	230.23	299.42	225.78	215.22	0.20
KLMA_80401	RPT5	26S protease regulatory subunit 6A	808.90	962.04	708.95	829.28	1058.99	915.35	0.18
KLMA_80402		probable 26S proteasome complex subunit SEM1	64.67	83.90	69.43	78.22	83.41	70.88	0.09
KLMA_80403	RPO31	DNA-directed RNA polymerase III subunit RPC1	1947.01	1774.18	1900.28	1137.95	1112.07	1153.05	-0.72
KLMA_80404	ESC2	protein ESC2	185.77	145.42	148.61	201.85	233.37	188.43	0.38
KLMA_80405	APE4	putative aspartyl aminopeptidase	937.06	1173.46	964.76	718.26	771.71	834.97	-0.40

Locus_tag	UniProt_gene	Product	Unique exon reads						log2 Fold Change
			KmWT.1	KmWT.2	KmWT.3	Kmmig1.1	Kmmig1.2	Kmmig1.3	
KLMA_80406		uncharacterized trans-sulfuration enzyme YHR112C	1410.87	981.06	1163.31	830.97	824.79	905.84	-0.47
KLMA_80407	VPS13	vacuolar protein sorting-associated protein 13	680.75	1110.82	874.61	1086.65	896.40	906.71	0.12
KLMA_80408	SDH2	succinate dehydrogenase [ubiquinone] iron-sulfur subunit	1850.60	2052.72	2019.65	2501.31	3475.22	2994.12	0.60
KLMA_80409	ATG10	autophagy-related protein 10	52.91	64.88	57.25	68.97	57.29	63.96	0.12
KLMA_80410	GOS1	protein transport protein GOS1	209.28	182.34	193.68	212.79	242.63	245.48	0.26
KLMA_80411	FPS1	glycerol uptake/efflux facilitator protein	585.51	530.24	644.39	1121.97	664.71	745.94	0.53
KLMA_80412	GUT1	glycerol kinase	298.64	349.02	339.86	1190.94	1299.10	1363.09	1.96
KLMA_80413	RPL8B	60S ribosomal protein L8-B	17173.87	12723.53	16420.34	4732.64	8508.18	7176.73	-1.18
KLMA_80414	SBP1	single-stranded nucleic acid-binding protein	2083.39	1874.86	1935.60	1201.03	1611.66	1464.21	-0.46
KLMA_80415	YBT1	ATP-dependent bile acid permease	2894.64	2525.91	2684.75	1871.36	2391.79	2586.15	-0.24
KLMA_80416	MUP3	low-affinity methionine permease	450.30	413.90	481.16	570.24	495.38	513.43	0.23
KLMA_80417	COF1	actin-depolymerizing factor 1	1543.73	1646.65	1534.84	1160.66	1733.82	1491.01	-0.11
KLMA_80418	EFM1	uncharacterized protein YHL039W	892.38	753.97	801.53	917.59	917.46	873.00	0.15
KLMA_80419	isp4	sexual differentiation process protein isp4	529.08	492.21	627.34	404.55	212.30	227.32	-0.97
KLMA_80421		uncharacterized oligopeptide transporter C1840.12	395.04	363.56	398.33	229.61	152.49	212.63	-0.96
KLMA_80423		flocculation protein FLO9	2.35	0.00	3.65	3.36	0.00	1.73	-0.23
KLMA_80426		flocculation protein FLO5	1135.75	986.65	1063.42	1831.83	1289.83	1491.01	0.53
KLMA_80427	FCY2	purine-cytosine permease FCY2	47.03	34.68	77.96	1350.74	450.73	477.99	3.84
KLMA_80428		hypothetical protein	99.94	114.10	204.65	143.82	89.30	177.19	-0.03
